# Supplementary material for: Green oxidation of indoles using halide catalysis
Source: Nat Commun. 2019 Oct 18;10:4754. doi: 10.1038/s41467-019-12768-4 (PMC6802211; doi:10.1038/s41467-019-12768-4)
Supplement: Supplementary file 2 — Supplementary Information [file 41467_2019_12768_MOESM2_ESM.pdf]

Supplementary Information

Green Oxidation of Indoles using Halide Catalysis

Jun Xu[#], Lixin Liang[#], Haohao Zheng, Yonggui Robin Chi, & Rongbiao Tong*

#These authors contributed equally to this work.

Supplementary Methods

General Information

Oxone-halide oxidation of indoles was carried out in glassware without exclusion of air and moisture (open flask reaction) and the solvents were used as received from commercial suppliers without prior purification. Other reactions were carried out in oven or flame-dried glassware under a nitrogen atmosphere, unless otherwise noted. Tetrahydrofuran (THF) was freshly distilled before use from sodium using benzophenone as indicator. Dichloromethane was freshly distilled before use from calcium hydride (CaH_2). All other solvents were dried over 3Å or 4Å molecular sieves. Solvents were used as received from commercial suppliers without prior purification for workup, extraction and column chromatography. Reactions were monitored by thin-layer chromatography (TLC, 0.25 mm) on pre-coated silica gel plates. Flash chromatography was performed with silica gel 60 (particle size 0.040−0.062 mm). ^{1}H- and ^{13}C-NMR spectra were recorded on a 400 MHz spectrometer (400 MHz for ^{1}H, 100 MHz for ^{13}C). Chemical shifts are reported in parts per million (ppm) as values relative to the internal chloroform. Abbreviations for signal coupling are as follows: s, singlet; d, doublet; t, triplet; q, quartet; m, multiplet. HRMS spectrometry was detected by Agilent CI-TOF or 6540 Q-TOF. Infrared spectrometry was recorded on ALPHA FTIR (Bruker) or Tensor-27 FTIR (Bruker).

Preparation methods of tetrahydro-β-carbolines (THCs)

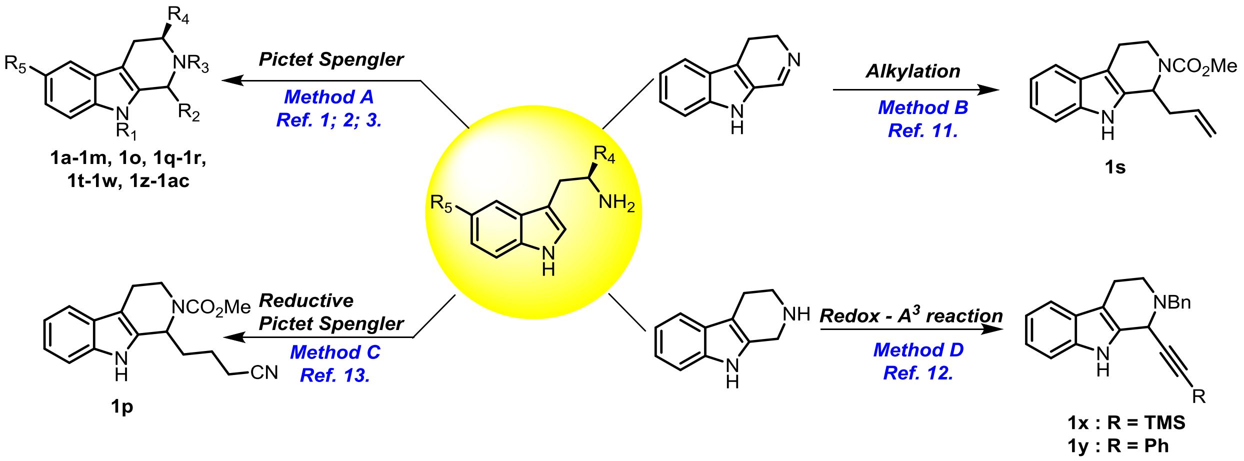

Pictet-Spengler reaction[1,2,3] for the preparation of compounds 1a[4], 1b[5], 1c – 1d, 1e, 1f[1], 1g[6], 1h[1], 1i[Error! Bookmark not defined.], 1j[7], 1k, 1l, 1n[8],1m[9], 1o, 1q, 1r, 1t[2], 1u[10], 1v, 1w, 1z, 1aa[11], 1ab to 1ac. Alkylation of 3,4 – dihydro – β – carboline[9] for the synthesis of compound **1s**. CuI-catalyzed Redox – A^3 reaction for the synthesis of compounds 1x and 1y. Reductive Pictet-Spengler cyclization[13] for the synthesis of compound **1p**. The physical data for new compounds were provided below.

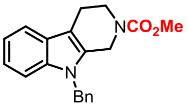

1c

1c (362 mg, 87% yield) was prepared using Method A. ^{1}H-NMR (400 MHz, $CDCl_3$) δ: 7.59 (d, *J* = 7.2 Hz, 1H), 7.44 – 7.15 (m, 6H), 7.06 (d, *J* = 7.0 Hz, 2H), 5.24 (s, 2H), 4.62 (d, *J* = 19.4 Hz, 2H), 3.84 (m, 5H), 2.91 (s, 2H). ^{13}C-NMR (100 MHz, $CDCl_3$) δ: 156.2, 137.2, 136.8, 128.7, 128.3, 127.3, 126.7, 126.5, 126.0, 121.4, 119.2, 117.9, 109.1, 52.6, 46.5, 41.9, 41.3,

21.3, 20.9. IR (neat) 2956.3, 2923.7, 2850.3, 1703.7, 1466.2, 1448.6, 1407.3, 1234.5, 739.8 cm^{-1}; HRMS (CI^+) (m/z) *calcd.* for $C_{20}H_{20}N_2O_2$ $[M]^+$320.1525; found 320.1518.

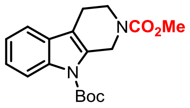

1d

1d (185 mg, 92% yield) was prepared using Method A. ^{1}H-NMR (400 MHz, $CDCl_3$) δ 8.16/8.12 (brs, 1H), 7.36 (s, 1H), 7.24 m, 2H), 4.87 (s, 2H), 3.77 (s, 5H), 2.72 (s, 2H), 1.69 (s, 9H). ^{13}C-NMR 1 (100 MHz, $CDCl_3$) δ 156.1, 149.9, 135.4, 131.2, 128.6, 123.8, 122.5, 117.6, 115.2, 115.1, 114.6, 83.8, 52.6, 44.2, 43.9, 40.9, 28.0, 21.2, 20.8. IR (neat) 2977.2, 1698.5,1617.1, 1447.3, 1362.7, 1314.5, 1226.2, 1143.1, 1109.0, 741.4 cm^{-1}; HRMS (CI^+) (m/z) *calcd.* for $C_{18}H_{22}N_2O_4$ $[M]^+$330.1580; found 330.1584.

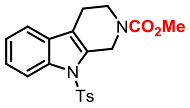

1d'

1d' (285 mg, 89% yield) was prepared using Method A. ^{1}H-NMR (400 MHz, $CDCl_3$) δ 8.12 (d, J = 8.3 Hz, 1H), 7.70 (brs, 2H), 7.38 – 7.27 (m, 2H), 7.22 (dd, J = 16.7, 7.6 Hz, 3H), 4.94 (s, 2H), 3.77 (s, 5H), 2.69 (s, 2H), 2.32 (s, 3H). ^{13}C-NMR (100 MHz, $CDCl_3$) δ156.1, 144.9, 136.0, 135.4, 131.0, 129.9, 129.4, 126.4, 124.5, 123.5, 118.3, 114.20 108.6, 52.82, 43.4, 41.0, 21.5, 21.0. IR (neat) 2915.2, 1695.9, 1600.7, 1444.1, 1403.5, 1367.2, 1227.0, 1172.7, 1103.5, 962.4, 808.5, 732.7, 666.1, 576.2 cm^{-1}; HRMS (CI^+) (m/z) *calcd.* for $C_{20}H_{20}N_2O_4S$ $[M]^+$384.1141; found 384.1136.

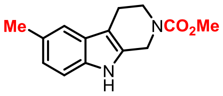

1k

1k (311 mg, 93% yield) was prepared using Method A. ^{1}H-NMR (400 MHz, $CDCl_3$) δ 8.16/ 7.91 (brs, 1H), 7.29 (s, 1H), 7.18 (d, J = 8.2 Hz, 1H), 7.00 (d, J = 8.2 Hz, 1H), 4.64 (d, J = 13.0 Hz, 2H), 3.81 (s, 5H), 2.79 (s, 2H), 2.47 (s, 3H). ^{13}C-NMR (100 MHz, CDC_{l3}) δ 156.5, 134.4, 130.3, 120.0, 128.6, 127.1, 123.1 117.6, 110.5, 107.7, 52.8, 42.2, 21.4, 20.9. IR (neat) 1692.8, 1447.2, 1409.5, 1317.2, 1230.9, 1194.4, 1129.0, 1102.1, 983.9, 916.2, 797.9, 752.6 cm^{-1}; HRMS (CI^+) (m/z) *calcd.* for $C_{14}H_{16}N_2O_4$ $[M]^+$244.1212; found 244.1216.

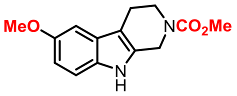

1l

1l (112 mg, 86% yield) was prepared using Method A. ^{1}H-NMR (400 MHz, $CDCl_3$) δ 8.40/ 8.13 (brs, 1H), 7.17 (d, J = 8.7 Hz, 1H), 6.95 (s, 1H), 6.82 (dd, J = 8.7, 2.4 Hz, 1H), 4.66/4.61 (s, 2H), 3.87 (s, 3H), 3.79 (m, 5H), 2.78 (s, 2H). ^{13}C-NMR (100 MHz, $CDCl_3$) δ 156.5, 156.3, 153.9, 131.2, 130.7, 127.2, 111.5, 111.2, 107.9, 100.2, 55.8, 52.8, 42.2, 21.3, 21.0. IR (neat) 2954.5, 2848.4, 2360.4, 2341.5, 1688.2, 1596.7, 1482.2, 1448.4, 1409.5, 1268.7, 1227.8, 1137.7, 1102.5, 1032.1, 800.2, 766.0 cm^{-1}; HRMS (CI^+) (m/z) *calcd.* for $C_{14}H_{16}N_2O_3$ $[M]^+$260.1161; found260.1170.

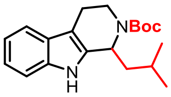

1o

1o (1.408 g, 86%) was prepared using Method A. ^{1}H-NMR (400 MHz, $CDCl_3$) δ 8.23/ 8.10 (brs, 1H), 7.50 (d, J = 7.2 Hz, 1H), 7.40 – 7.23 (m, 1H), 7.23 – 7.06 (m, 2H), 5.46/5.29 (brs, 1H), 4.40 (brs, 1H), 3.18 (m, 1H), 2.87 (m, 1H), 2.69 (dd, J = 15.2, 3.9 Hz, 1H), 1.80 (d, J = 13.0 Hz, 2H), 1.55 (d, J = 8.8 Hz, 10H), 1.18 – 0.95 (m, 6H). ^{13}C-NMR (100 MHz, $CDCl_3$) δ 155.4, 155.0, 146.8, 135.9, 135.5, 134.9, 127.0, 121.8, 121.6, 119.6, 119.4, 118.2, 117.9, 110.8, 108.8, 108.1, 85.3, 80.2, 79.7, 49.9, 49.3, 44.4, 43.9, 38.5, 37.2, 28.5, 27.4, 25.2, 24.9, 23.4, 22.6, 21.5, 21.1. IR (neat) 2963.5,

1804.1, 1762.0, 1664.9, 1460.7, 1414.7, 1365.2, 1313.9, 1220.8, 1163.3, 1116.3, 1066.5, 738.5 cm^{-1}; HRMS (CI^+) (m/z) *calcd.* for $C_{20}H_{28}N_2O_2$ $[M+H]^+$ 328.2151; found 328.2150.

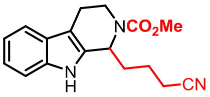

1p

1p (310 mg, 21% yield) was prepared using Method C. ^{1}H-NMR (400 MHz, $CDCl_3$) δ 8.56/ 8.38 (s, 1H), 7.49 (d, *J* = 7.7 Hz, 1H), 7.32 (d, *J* = 8.0 Hz, 1H), 7.25 – 7.07 (m, 2H), 5.47 – 5.17 (m, 1H), 4.61 – 4.29 (m, 1H), 3.81 (s, 3H), 3.22 –3.16 (m, 1H), 2.94 – 2.80 (m, 1H), 2.72 (dd, *J* = 15.4, 4.0 Hz, 1H), 2.55 – 2.32 (m, 2H), 2.03 (ddt, *J* = 10.1, 6.2, 3.8 Hz, 1H), 1.93 (dtd, *J* = 13.8, 9.2, 5.2 Hz, 1H), 1.80 (brs, 2H).^{13}C-NMR (100 MHz, $CDCl_3$) δ 156.9, 156.2, 136.0, 133.4, 132.9, 126.5, 121.8, 119.5, 119.4, 118.0, 110.9, 108.1, 52.9, 50.5, 38.4, 33.4, 33.0, 21.8, 21.3, 20.9, 16.6. IR (neat) 2918.1, 1148.1, 1343.0, 1163.0, 1116.2, 745.8, 600.2 cm^{-1}; HRMS (CI^+) (m/z) *calcd.* for $C_{17}H_{19}N_3O_2$ $[M+H]^+$ 298.1556; found 298.1568.

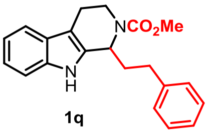

1q

1q (420 mg, 20% yield) was prepared using Method A. ^{1}H-NMR (400 MHz, $CDCl_3$) δ 7.98 – 7.73 (m, 1H), 7.48 (m, 1H), 7.29 m, 3H), 7.22 (m, 3H), 7.19 – 7.08 (m, 2H), 5.50 – 5.20 (m, 1H), 4.64 – 4.29 (m, 1H), 3.80 (s, 3H), 3.24 (s, 1H), 2.95 – 2.67 (m, 4H), 2.15 (d, *J* = 7.8 Hz, 2H). ^{13}C-NMR (100 MHz, $CDCl_3$) δ 156.7, 141.6, 135.9, 128.7, 128.5, 128.3, 126.8, 126.0, 121.8, 119.5, 118.0, 110.8, 108.9, 52.8, 51.4, 38.6, 36.6, 36.4, 32.5, 21.4. IR (neat) 2922.4, 2852.3, 1675.6, 1446.0, 1224.8, 1105.9, 1005.8, 736.4, 695.5 cm^{-1}; HRMS (CI^+) (m/z) *calcd.* for $C_{21}H_{22}N_2O_2$ $[M+H]^+$335.1760; found 335.1752.

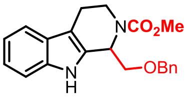

1r

1r (230 mg, 48% yield) was prepared using Method A ^{1}H-NMR (400 MHz, $CDCl_3$) δ 8.37 (brs, 1H), 7.51 (d, *J* = 7.8 Hz, 1H), 7.46 – 7.28 (m, 6H), 7.22 – 7.14 (m, 1H), 7.14 – 7.06 (m, 1H), 5.36 (brs, 1H), 4.59 (m, 3H), 3.86 (brs, 1H), 3.77 (s, 3H), 3.66 (dd, *J* = 9.7, 8.2 Hz, 1H), 3.11 (brs, 1H), 2.89 – 2.69 (m, 2H). ^{13}C-NMR (100 MHz, $CDCl_3$) δ 156.0, 137.7, 136.0, 132.5, 128.6, 128.0, 127.9, 126.4, 121.8, 119.4, 118.2, 110.9, 109.5, 73.8, 71.5, 71.2, 52.9, 50.4, 40.3, 21.6. IR (neat) 2943.5, 2857.8, 1682.2, 1450.0, 1405.0, 1365.4, 1309.7, 1232.1, 1103.1, 1011.2, 737.0, 696.5 cm^{-1}; HRMS (CI^+) (m/z) *calcd.* for $C_{21}H_{22}N_2O_3$ $[M]^+$350.1630; found 350.1642.

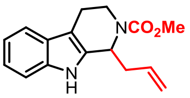

1s

1s (398 mg, 79% yield) was prepared using Method B. ^{1}H-NMR (400 MHz, $CDCl_3$) δ 8.42,/8.28 (s, 1H), 7.52 (d, *J* = 7.5 Hz, 1H), 7.33 (d, *J* = 7.9 Hz, 1H), 7.25 – 7.11 (m, 2H), 6.00 – 5.90 (m, 1H), 5.52 – 5.11 (m, 3H), 4.62– 4.37 (m, 1H), 3.83 (brs, 3H), 3.26 (m, 1H), 2.94 – 2.71 (m, 2H), 2.65 (dt, *J* = 10.8, 5.0 Hz, 2H). ^{13}C-NMR (100 MHz, $CDCl_3$) δ 156.3, 156.1, 135.9, 134.2, 133.6, 133.3, 126.5, 121.6, 119.3, 118.4, 118.2, 117.9, 110.9, 108.9, 108.2, 52.7, 51.1, 39.3, 38.9, 38.8, 38.5, 21.4, 21.1. IR (neat) 2916.7, 1676.3, 1450.3, 1408.0, 1368.8, 1229.7, 1106.2, 997.0, 918.7, 743.5 cm^{-1}; HRMS (CI^+) (m/z) *calcd.* for $C_{16}H_{18}N_2O_2$ $[M]^+$270.1368; found 270.1367.

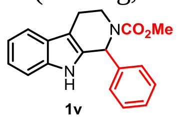

1v

1v (852 mg, 89% yield) was prepared using Method A. ^{1}H-NMR (400 MHz, $CDCl_3$) δ 8.21 (s, 1H), 7.60 (s, 1H), 7.31 (dd, *J* = 17.9, 5.2 Hz, 6H), 7.25 – 7.15 (m, 2H), 6.46 (brs, 1H), 4.36 (brs, 1H), 3.78 (s, 3H), 3.22 (t, *J* = 10.8 Hz, 1H), 2.98 (td, *J* = 13.8, 12.0, 5.1 Hz, 1H), 2.86 (dd, *J* = 15.4, 3.6 Hz, 1H).^{13}C-NMR (100 MHz, $CDCl_3$) δ 156.1, 139.9, 136.2, 131.4, 128.4, 128.0, 126.6, 121.9, 119.4, 118.1, 111.0, 110.1, 54.4, 52.7, 38.1, 21.3. IR (neat)1677.9, 1451.6, 1403.2, 1224.1, 1102.3, 741.2, 697.0 cm^{-1}; HRMS (CI$^+$) (m/z) *calcd.* for $C_{19}H_{18}N_2O_2$ [M]$^+$306.1368; found 306.1367.

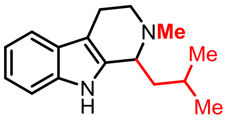

1w

1w (968 mg, 80% yield) was prepared using Method A. ^{1}H-NMR (400 MHz, $CDCl_3$) δ 7.71 (brs, 1H), 7.49 (d, *J* = 7.6 Hz, 1H), 7.32 (d, *J* = 7.7 Hz, 1H), 7.12 (m, 2H), 3.63 (t, *J* = 6.6 Hz, 1H), 3.20 (qd, *J* = 7.9, 3.7 Hz, 1H), 2.95 – 2.80 (m, 2H), 2.72 – 2.61 (m, 1H), 2.48 (s, 3H), 1.93 (dq, *J* = 7.9, 6.4 Hz, 1H), 1.74 (ddd, *J* = 13.8, 7.6, 6.0 Hz, 1H), 1.63 – 1.51 (m, 1H), 1.02 (d, *J* = 6.5 Hz, 3H), 0.97 (d, *J* = 6.7 Hz, 3H).^{13}C-NMR (100 MHz, $CDCl_3$) δ 135.7, 135.4, 127.3, 121.2, 119.22, 118.0, 111.6, 107.3, 57.6, 47.5, 43.2, 41.3, 25.2, 23.2 , 22.6, 17.6. IR (neat) 3407.6, 2944.2, 2865.0, 2794.3, 1455.4, 1358.0, 1308.3, 1155.6, 737.5 cm^{-1}; HRMS (CI$^+$) (m/z) *calcd.* for $C_{16}H_{22}N_2$ [M]$^+$242.1783; found 242.1790.

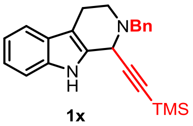

1x

1x (455 mg, 44% yield) was prepared using Method D. ^{1}H-NMR (400 MHz, $CDCl_3$) δ 7.85 (s, 1H), 7.53 (dd, *J* = 7.1, 4.8 Hz, 3H), 7.45 – 7.28 (m, 4H), 7.23 – 7.10 (m, 2H), 4.61 (s, 1H), 3.98 (dd, *J* = 73.5, 13.0 Hz, 2H), 3.20 – 3.09 (m, 1H), 2.96 – 2.75 (m, 3H), 0.28 (s, 9H). ^{13}C-NMR (100 MHz, $CDCl_3$) δ 138.1, 136.0, 131.1, 129.4, 128.3, 127.2, 127.0, 121.7, 119.4, 118.3, 110.9, 108.6, 101.5, 91.4, 58.8, 50.1, 46.9, 21.4, 0.1. IR (neat) 3404.4, 2955.1, 2903.3, 2823.4, 2157.7, 1453.9, 1304.0, 1250.5, 1017.8, 950.8, 842.9, 739.0, 698.1 cm^{-1}; HRMS (CI$^+$) (m/z) *calcd.* for $C_{23}H_{26}N_2Si$ [M]$^+$358.1865; found 358.1860.

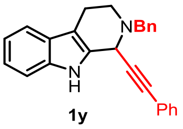

1y

1y (580 mg, 55% yield) was prepared using Method D**Error! Bookmark not defined.**. ^{1}H-NMR (400 MHz, $CDCl_3$) δ 7.89 (s, 1H), 7.62 – 7.48 (m, 5H), 7.48 – 7.30 (m, 7H), 7.18 (m, 2H), 4.83 (s, 1H), 4.06 (dd, *J* = 70.0, 13.1 Hz, 2H), 3.28 – 3.16 (m, 1H), 3.01 – 2.80 (m, 3H). ^{13}C-NMR (100 MHz, $CDCl_3$) δ 138.2, 136.1, 131.8, 131.3, 129.3, 128.4, 128.3, 128.3, 127.2, 127.0, 122.6, 121.8, 119.4, 118.36, 110.9, 108.7 ,86.7, 85.5, 58.9, 50.1, 47.1, 21.5. IR (neat) 3405.0, 2904.1, 2828.8, 1451.2, 1305.4, 1156.4, 1113.3, 914.0, 694.6 cm^{-1}; HRMS (CI$^+$) (m/z) *calcd.* for $C_{26}H_{22}N_2$ [M]$^+$362.1783; found 362.1780.

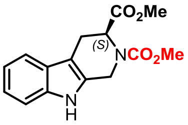

1z

1z (769 mg, 89% yield) was prepared using Method A. ^{1}H-NMR (400 MHz, $CDCl_3$) δ 8.76/ 8.55 (s, 1H), 7.58 (d, *J* = 7.0 Hz, 1H), 7.34 – 7.27 (m, 1H), 7.25 – 7.15 (m, 2H), 5.48 (dd, *J* = 77.4, 5.3 Hz, 1H), 4.96 (dd, *J* = 46.7, 16.0 Hz, 1H), 4.67 (t, *J* = 17.1 Hz, 1H), 3.90 (d, *J* = 6.4 Hz, 3H), 3.63 (d, *J* = 7.6 Hz, 3H), 3.54 (d, *J* = 15.6 Hz, 1H), 3.20 (m, 1H). ^{13}C-NMR (100 MHz, $CDCl_3$) δ 171.8, 171.6, 157.1, 156.6, 136.2, 136.2, 129.2, 129.1, 126.3, 126.3, 121.6, 121.5, 119.2, 119.1, 117.8, 117.7, 110.8, 110.7, 105.3, 104.7, 53.2, 53.0, 53.0, 52.3, 52.2, 40.5, 40.2, 23.2, 22.9. IR (neat) 2289.9,

2953.6, 1734.6, 1689.1, 1629.5, 1446.6, 1400.6, 1328.9, 1281.4, 1230.9, 1193.0, 1105.0, 1024.1, 951.4, 742.0 cm^{-1}; HRMS (CI^+) (m/z) *calcd.* for $C_{15}H_{16}N_2O_4$ $[M]^+$288.1110; found 288.1100. $[\alpha]_D^{25}$= +84.7 (*c* 1.0, $CHCl_3$)

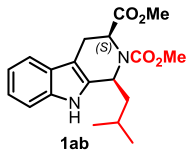

1ab

1ab (613 mg, 39% yield) was prepared using Method A. ^{1}H-NMR (400 MHz, $CDCl_3$) δ 8.31 (s, 1H), 7.54 (d, *J* = 7.3 Hz, 1H), 7.29 (d, *J* = 7.3 Hz, 1H), 7.20– 1.72 (m, 2H), 5.54 (m, 1H), 5.42 – 5.26 (m, 1H), 3.87/3.84 (s, 3H), 3.73/ 3.69 (d, 3H), 3.48 (m, 1H),3.11– 3.06 (m, 1H), 1.94 (m, 1H), 1.85 – 1.71 (m, 1H), 1.61 (m, 1H), 1.09 (d, *J* = 6.2 Hz, 3H), 1.05 (d, *J* = 6.6 Hz, 3H).^{13}C-NMR (100 MHz, $CDCl_3$) δ 172.4, 157.0, 156.3, 136.0, 135.9, 133.8, 133.1, 126.3, 121.7, 121.6, 119.3, 118.1, 117.9, 110.7, 105.5, 104.8, 53.2, 53.0, 52.2, 51.8, 49.8, 45.6, 45.2, 25.1, 22.7, 22.3, 21.9, 21.4. IR (neat) 3333.6, 3305.1, 1738.3, 1679.6, 1445.0, 1399.1, 1323.0, 1237.5, 1198.6, 1105.9, 1038.2, 734.3 cm^{-1}; HRMS (CI^+) (m/z) *calcd.* for $C_{19}H_{24}N_2O_4$ $[M]^+$344.1736; found 344.1750. $[\alpha]_D^{25}$= +49.1 (*c* 1.0, $CHCl_3$)

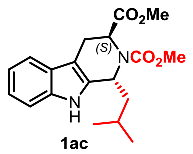

1ac

1ac (166 mg, 11% yield) was prepared using Method A. ^{1}H-NMR (400 MHz, $CDCl_3$) δ 9.33 (brs, 1H), 7.28 – 7.17 (m, 2H), 7.00 (t, *J* = 7.5 Hz, 1H), 6.92 (d, *J* = 7.7 Hz, 1H), 4.90 (t, *J* = 8.5 Hz, 1H), 4.26 (brs, 1H), 3.77 (s, 3H), 3.71 (s, 3H), 2.56 (dd, *J* = 13.3, 8.7 Hz, 1H), 2.44 (dd, *J* = 13.3, 9.0 Hz, 1H), 1.85 – 1.65 (m, 1H), 1.65 – 1.34 (m, 2H), 0.91 (d, *J* = 6.4 Hz, 3H), 0.81 (s, 3H). ^{13}C-NMR (100 MHz, $CDCl_3$) δ 171.6, 171.3, 157.2, 156.9, 1356.0, 135.1, 128.9, 126.4, 121.6, 119.5, 117.8, 110.9, 107.0, 106.2, 54.3, 52.9, 52.4, 52.2, 44.3, 43.8, 24.7, 23.0, 22.8, 22.7, 22.4. IR (neat) 3353.9, 2953.4, 1739.4, 1687.1, 1446.9, 1337.4, 1273.1, 1227.5, 738.1 cm^{-1}; HRMS (CI^+) (m/z) *calcd.* for $C_{19}H_{24}N_2O_4$ $[M]^+$344.1736; found 344.1732. $[\alpha]_D^{25}$= –4.4 (*c* 1.0, $CHCl_3$)

Oxidative Rearrangement of THCs to Spirooxindoles

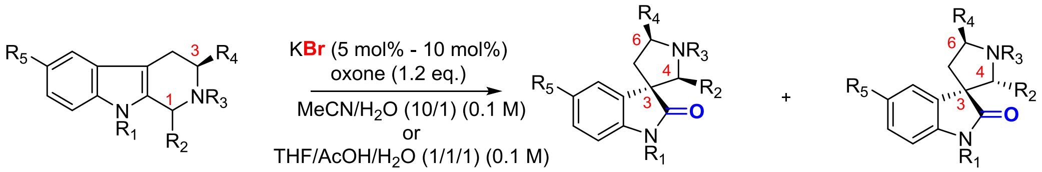

General Procedure-1: A 0.01 M or 0.005 M stock solution of KBr in $MeCN/H_2O$ (v/v 10:1) was prepared as follows: KBr (11.9 mg, 0.1 mmol for 0.01 M stock solution or 6 mg, 0.05 mmol for 0.005 M stock solution) was dissolved in 10.0 mL of $MeCN/H_2O$ (v/v, 10:1). To a solution of THCs (1.0 equiv.) and KBr (0.05 – 0.1equiv.) in $MeCN/H_2O$ (10/1) (0.1 M) at 0 ºC was added oxone (1.2 equiv., MW 307) in one batch. The resulting solution was allowed to warm to rt and stirred for additional 0.5 to 16 h. After the reaction was completed as determined by TLC analysis, the reaction was quenched by addition of *aq. sat.* $NaHCO_3$ and *aq. sat.* Na_2SO_3 and then diluted with EtOAc. The organic fractions were collected, and the aqueous phase was extracted with ethyl acetate three times. The combined organic fractions were washed with brine, dried over Na_2SO_4, filtered, and concentrated under reduced pressure. The resulting residue was purified by flash column

chromatography (EtOAc/hexane = 1:10 to 1:1) to provide the spirooxindole. **General procedure-1** was used for the synthesis of spirooxindoles **2a**–**2h**, **2k**–**2t**, **2w**, and **2y**–**2ab**.

General Procedure-2: A 0.0 1 M or 0.005 M stock solution of KBr in THF/H_2O/AcOH (v/v 1:1:1) was prepared as follows: KBr (11.9 mg, 0.1 mmol for 0.01M stock solution or 6 mg, 0.05 mmol for 0.005 M stock solution) was dissolved in 10.0 mL of THF/H_2O/AcOH (v/v 1:1:1). To a solution of THCs (1.0 equiv.) and KBr (0.05 – 0.1equiv.) in THF/H_2O/AcOH(1/1/1) (0.1M) at 0 °C was added oxone (1.2 equiv., MW 307) at one batch. The resulting reaction mixture was allowed to warm to rt and stirred for additional 0.5 to 16 h. After the reaction was completed as determined by TLC analysis, the reaction was quenched by addition of *aq. sat.* $NaHCO_3$ and *aq. sat.* Na_2SO_3 and then diluted with EtOAc. The organic fractions were collected, and the aqueous phase was extracted with ethyl acetate three times. The combined organic fractions were washed with brine, dried over Na_2SO_4, filtered, and concentrated under reduced pressure. The resulting residue was purified by flash column chromatography (EtOAc/hexane 1:10 to 1:1 or MeOH/CH_2Cl_2 100:1 to 10:1) to provide the desired spirooxindole. **General procedure-2** was used for the synthesis of spirooxindoles **2i**, **2j**, **2v** and **2x**.

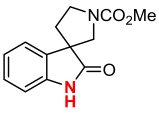

2a

2a[4]**:** Following **General procedure-1**, reaction of **1a** (46 mg, 0.2 mmol) with KBr (2.0 mL, 0.005 M, 0.01 mmol) and oxone (74 mg, 0.24 mmol) provided spirooxindole **2a (**45.4 mg, 93% yield). ^{1}H-NMR(400 MHz, $CDCl_3$) δ: 9.79/9.74 (s, 1H), 7.19 (t, *J* = 7.7 Hz, 1H), 7.12 (dd, *J* = 7.5, 2.5 Hz, 1H), 7.05 – 6.89 (m, 2H), 3.95 – 3.67 (m, 6H), 3.60 (dd, *J* = 35.2, 11.1 Hz, 1H), 2.38 (m, 1H), 2.07 (m, 1H). ^{13}C-NMR (100 MHz, $CDCl_3$) δ 179.9, 179.6, 154.9, 154.8, 140.1, 140.0, 131.9, 131.6, 127.9, 122.2, 122.0, 109.7, 53.6, 53.3, 52.7, 51.8, 45.1, 44.6, 35.7, 34.8. IR (neat) 2956.9, 2881.5, 1680.4, 1617.2, 1455.8, 1388.2, 1337.7, 1190.1, 1125.6, 743.5 cm^{-1}; HRMS (CI+) (m/z) *calcd.* for $C_{13}H_{14}N_2O_3$ [M]+ 246.1004; found 246.0992.

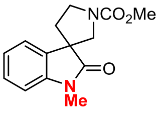

2b

2b: Following the **General Procudure-1**, reaction of **1b** (70 mg, 0.29 mmol) with KBr (2.9 mL, 0.005 M, 0.0145 mmol) and oxone (106 mg, 0.35 mmol, MW 307) provided spirooxindole **2b** (71 mg, 95% yield). ^{1}H-NMR (400 MHz, $CDCl_3$) δ 7.33 – 7.24 (m, 1H), 7.15 (d, *J* = 7.7 Hz, 1H), 7.04 (t, *J* = 7.5 Hz, 1H), 6.85 (d, *J* = 7.8 Hz, 1H), 3.92 – 3.66 (m, 6H), 3.57 (dd, *J* = 32.2, 10.9 Hz, 1H), 3.20 (s, 3H), 2.38 (dt, *J* = 12.6, 8.3 Hz, 1H), 2.13 – 1.96 (m, 1H). ^{13}C-NMR (100 MHz, $CDCl_3$) δ 177.3, 177.0, 155.2, 155.2, 142.8, 142.7, 132.2, 131.8, 131.2, 128.4, 125.5, 122.9, 122.9, 122.2, 109.6, 108.2, 54.3, 52.9, 52.7, 52.4, 51.7, 45.6, 45.1, 36.2, 35.3, 26.3 IR (neat) 1712.9, 1613.6, 1494.2, 1455.1, 1375.9, 1351.9,1137.1, 752.7 cm^{-1}; HRMS (CI$^+$) (m/z) *calcd.* for $C_{14}H_{16}N_2O_3$ $[M+H]^+$ 260.1239; found 261.1227.

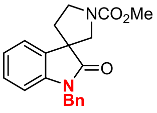

2c

2c: Following the **General Procudure-1**, reaction of **1c** (55 mg, 0.17 mmol) with KBr (1.7 mL, 0.005 M, 0.0085 mmol) and oxone (63 mg, 0.20 mmol, MW 307) provided spirooxindole **2c** (55mg, 95% yield). ^{1}H-NMR (400 MHz, $CDCl_3$) δ 7.34 – 7.23 (m, 5H), 7.22 – 7.15 (m, 2H), 7.03 (t, *J* = 7.5 Hz, 1H), 6.76 (d, *J* = 7.5 Hz, 1H), 4.92 (s, 2H), 3.96 – 3.58 (m, 7H), 2.48 (dt, *J* = 12.6, 8.1 Hz, 1H), 2.12 (brs, 1H). ^{13}C-NMR (100 MHz, $CDCl_3$) δ 177.3, 155.3, 141.9, 135.6, 132.2, 131.7, 128.8, 128.4, 127.7, 127.1, 123.0, 122.4, 109.3, 54.5, 54.1, 52.8, 52.6, 51.8, 45.7, 45.2, 43.8, 36.3, 35.5. IR (neat)

1710.4, 1613.4, 1488.7, 1454.8, 1364.4, 1194.5, 1132.2, 967.72, 698.5 cm^{-1}; HRMS (CI^+) (m/z) *calcd.* for $C_{20}H_{20}N_2O_3$ $[M+H]^+$ 337.1552; found 337.1541.

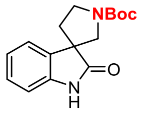

2e

2e[14]**:** Following the **General Procudure-1**, reaction of **1e** (50 mg, 0.185 mmol) with KBr (1.85 mL, 0.005 M, 0.009 mmol) and oxone (68 mg, 0.22 mmol, MW 307) provided spirooxindole **2e** (48.5 mg, 91% yield). ^{1}H-NMR (400 MHz, $CDCl_3$) δ 9.19/9.17 (s, 1H), 7.24 – 7.11 (m, 2H), 7.07 – 6.98 (m, 1H), 6.94 (d, *J* = 7.7 Hz, 1H), 3.90 – 3.66 (m, 3H), 3.58 (dd, *J* = 29.7, 11.0 Hz, 1H), 2.40 (dt, *J* = 12.8, 8.4 Hz, 1H), 2.12 – 1.99 (m, 1H), 1.47 (d, *J* = 24.4 Hz, 9H). ^{13}C-NMR (100 MHz, $CDCl_3$) δ 180.4, 180.1, 154.4, 140.3, 140.2, 133.1, 132.6, 128.4, 122.9, 122.8, 122.7, 110.1, 79.9, 79.8, 54.3, 53.8, 53.3, 52.4, 45.4, 45.2, 36.3, 35.5, 28.5, 28.4. IR (neat) 2975.9, 1687.6, 1472.2, 1401.4, 1167.0, 1127.2, 732.6 cm^{-1}; HRMS (CI^+) (m/z) *calcd.* for $C_{16}H_{20}N_2O_3$ $[M+Na]^+$ 311.1372; found 311.1254.

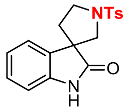

2f

2f: Following the **General Procudure-1**, reaction of **1f** (50 mg, 0.15 mmol) with KBr (1.5 mL, 0.005 M, 0.0075 mmol) and oxone (56 mg, 0.18 mmol, MW 307) provided spirooxindole **2f** (48.3 mg, 94% yield). ^{1}H-NMR (400 MHz, $CDCl_3$) δ 8.28 (s, 1H), 7.77 (d, *J* = 8.2 Hz, 2H), 7.37 (d, *J* = 8.0 Hz, 2H), 7.22 (td, *J* = 7.7, 1.3 Hz, 1H), 7.05 (d, *J* = 7.2 Hz, 1H), 6.98 (t, *J* = 7.5 Hz, 1H), 6.87 (d, *J* = 7.8 Hz, 1H), 3.73 (dt, *J* = 9.7, 7.3 Hz, 1H), 3.58 (d, *J* = 9.6 Hz, 1H), 3.56 – 3.50 (m, 1H), 3.46 (d, *J* = 9.7 Hz, 1H), 2.47 (s, 3H), 2.30 (ddd, *J* = 12.8, 8.6, 7.1 Hz, 1H), 2.06 (ddd, *J* = 12.7, 7.6, 5.2 Hz, 1H).^{13}C-NMR (100 MHz, $CDCl_3$) δ 179.0, 143.9, 139.7, 133.4, 132.7, 129.9, 128.6, 127.8, 123.2, 109.9, 56.0, 52.9, 47.3, 36.3, 21.6. IR (neat) 3308.9, 1715.5,1618.0, 1472.1, 1341.4, 1223.6, 1162.0, 1116.5, 754.2, 665.0 cm^{-1}; HRMS (CI^+) (m/z) *calcd.* for $C_{18}H_{18}N_2O_3S$ $[M+H]^+$ 343.1116; found 343.1111.

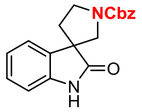

2g

2g[15]**:** Following the **General Procudure-1**, reaction of **1g** (40 mg, 0.13 mmol) with KBr (1.3 mL, 0.005 M, 0.0065 mmol) and oxone (48 mg, 0.157 mmol, MW 307) provided spirooxindole **2g** (37.0 mg, 90% yield). ^{1}H-NMR (400 MHz, $CDCl_3$) δ 8.86 (brs, 1H), 7.46 – 7.21 (m, 6H), 7.15 (dd, *J* = 12.0, 7.4 Hz, 1H), 7.03 (t, *J* = 7.4, 3.0 Hz, 1H), 6.94 (d, *J* = 7.7 Hz, 1H), 5.21 (d, *J* = 18.8 Hz, 2H), 4.00 – 3.89 (m, 1H), 3.88 – 3.77 (m, 2H), 3.69 (dd, *J* = 18.6, 11.1 Hz, 1H), 2.49 – 2.38 (m, 1H), 2.12 (m, 1H). ^{13}C-NMR (100 MHz, $CDCl_3$) δ 180.1, 179.9, 154.7, 140.1, 136.5, 132.5, 128.5, 128.4, 128.0, 127.9, 127.9, 127.8, 123.0, 122.7, 110.2, 67.0, 54.3, 55.0, 53.2, 52.3, 45.7, 45.2, 36.3, 35.4. IR (neat) 2959.5, 2881.4, 1694.6, 1617.2, 1416.9, 1336.6, 1118.0, 740.4, 694.5 cm^{-1}; HRMS (CI^+) (m/z) *calcd.* for $C_{19}H_{18}N_2O_3$ $[M]^+$ 322.1317; found 322.1322.

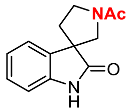

2h

2h: Following the **General Procudure-1**, reaction of **1h** (50 mg, 0.23 mmol) with KBr (2.3 mL, 0.005 M, 0.0115 mmol) and oxone (86 mg, 0.28 mmol, MW 307) provided spirooxindole **2h** (52.4 mg, 99% yield). ^{1}H-NMR (400 MHz, $CDCl_3$) δ 9.73 /9.44 (s, 1H), 7.22 (q, *J* = 7.9 Hz, 1H), 7.13 (dd, *J* = 7.4, 4.9 Hz, 1H), 7.06 – 6.92 (m, 2H), 3.97 (tt, *J* = 8.6, 6.0 Hz, 1H), 3.84 (ddt, *J* = 16.1, 10.1, 7.6 Hz, 2H), 3.67 (dd, *J* = 53.3, 11.3 Hz, 1H), 2.50 – 2.36 (m, 1H), 2.28 – 2.17 (m, 1H), 2.12 (d, *J* = 40.3 Hz, 3H).^{13}C-NMR (100 MHz, $CDCl_3$) δ 180.4, 179.3, 169.6, 169.5, 140.7, 140.4, 132.4, 131.5, 128.6, 128.5,

122.9, 122.7, 122.4, 110.4, 110.2, 55.3, 53.5, 53.4, 51.8, 46.6, 45.1, 36.3, 34.9, 22.5, 22.3. IR (neat) 3191.9, 1711.1, 1614.7, 1460.1, 1419.5, 1339.2, 728.9 cm^{-1}; HRMS (CI^+) (m/z) *calcd.* for $C_{13}H_{14}N_2O_2$ $[M+H]^+$ 231.1134; found 231.1137.

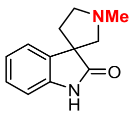

2i

2i[4]**:** Following the **General Procudure-2**, reaction of **1i** (37 mg, 0.2 mmol) with KBr (2.0 mL, 0.005 M, 0.01 mmol) and oxone (74 mg, 0.24 mmol, MW 307) provided spirooxindole **2i** (24.2 mg, 60% yield). ^{1}H-NMR (400 MHz, $CDCl_3$) δ 8.94 (brs, 1H), 7.28 (d, J = 7.4 Hz, 1H), 7.08 (td, J = 7.7, 1.0 Hz, 1H), 6.92 (t, J = 7.5 Hz, 1H), 6.80 (d, J = 7.7 Hz, 1H), 2.90 (ddd, J = 9.0, 7.5, 5.0 Hz, 1H), 2.83 – 2.67 (m, 3H), 2.36 (s, 3H), 2.34 – 2.27 (m, 1H), 2.00 (dt, J = 12.8, 7.3 Hz, 1H). ^{13}C-NMR (100 MHz, $CDCl_3$) δ 182.7, 140.0, 136.3, 127.8, 123.4, 122.9, 109.4, 66.4, 56.8, 53.6, 41.8, 38.0 .IR (neat) 2976.5, 2928.7, 1686.9, 1407.9, 1370.1, 1249.2, 1158.4, 1105.7, 1004.2, 920.0, 843.2, 761.4 cm^{-1}; HRMS (CI^+) (m/z) *calcd.* for $C_{12}H_{14}N_2O$ $[M]^+$ 202.1106; found 202.1126.

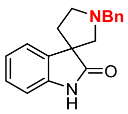

2j

2j[16]**:** Following the **General Procudure-2**, reaction of **1j** (52 mg, 0.2 mmol) with KBr (2.0 mL, 0.005 M, 0.01 mmol) and oxone (74 mg, 0.24mmol, MW 307) provided spirooxindole **2j** (41.2 mg, 74% yield). ^{1}H-NMR (400 MHz, $CDCl_3$) δ 8.84 (s, 1H), 7.50 (d, J = 7.3 Hz, 1H), 7.42 (d, J = 7.2 Hz, 2H), 7.32 (t, J = 7.4 Hz, 2H), 7.27 – 7.22 (M, 1H), 7.19 (t, J = 7.6 Hz, 1H), 7.06 (t, J = 7.5 Hz, 1H), 6.90 (d, J = 7.1 Hz, 1H), 3.82 (s, 2H), 3.20 (brs, 1H), 2.99 (s, J = 7.8 Hz, 1H), 2.85 (dd, J = 21.4, 9.1 Hz, 2H), 2.42 (ddd, J = 12.3, 8.1, 3.9 Hz, 1H), 2.16 (m, 1H). ^{13}C-NMR (100 MHz, $CDCl_3$) δ 182.1, 139.6, 137.9, 135.4, 128.3, 127.8, 127.2, 126.6, 123.0, 122.3, 108.8, 62.9, 59.4, 53.4, 52.5, 36.1. IR (neat) 1705.3, 1617.1, 1470.2, 476.5, 699.2 cm^{-1}; HRMS (CI^+) (m/z) *calcd.* for $C_{18}H_{18}N_2O$ $[M+H]^+$ 279.1497; found 279.1487.

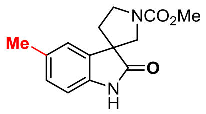

2k

2k: Following the **General Procudure-1**, reaction of **1k** (95 mg, 0.39 mmol) with KBr (2.0 mL, 0.005 M, 0.01 mmol) and oxone (143 mg, 0.47 mmol, MW 307) provided spirooxindole **2k** (96.1 mg, 95% yield). ^{1}H-NMR (400 MHz, $CDCl_3$) δ 9.44/9.37 (s,1H), 7.02 (d, J = 8.0 Hz, 1H), 6.95 (d, J = 9.0 Hz, 1H), 6.84 (dd, J = 8.0, 3.2 Hz, 1H), 3.93 – 3.70 (m, 6H), 3.60 (dd, J = 31.9, 11.0 Hz, 1H), 2.43 – 2.34 (m, 1H), 2.30 (s, 3H), 2.14 – 1.99 (m, 1H). ^{13}C-NMR (100 MHz, $CDCl_3$) δ 180.4, 180.0, 155.4, 155.4, 137.9, 137.8, 132.7, 132.4, 132.3, 128.7, 123.3, 109.9, 54.2, 53.8, 53.3, 52.5, 52.4, 45.7, 45.2, 36.3, 35.4, 21.0. IR (neat) 1712.5, 1626.7, 1493.4, 1455.6, 1394.1, 1324.4, 1195.4, 1129.2, 750.6c m^{-1}; HRMS (CI^+) (m/z) *calcd.* for $C_{14}H_{16}N_2O_3$ $[M]^+$ 260.1161; found 260.1158.

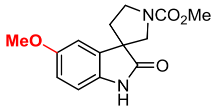

2l

2l: Following the **General Procudure-1**, reaction of **1l** (34 mg, 0.13 mmol) with KBr (1.3 mL, 0.005 M, 0.0065 mmol) and oxone (59 mg, 0.16 mmol, MW 307) provided spirooxindole **2l** (29.1 mg, 80% yield). ^{1}H-NMR (400 MHz, $CDCl_3$) δ 8.67/8.60 (s, 1H), 6.85 (d, J = 8.9 Hz, 1H), 6.77 (d, J = 6.1 Hz, 2H), 4.02 – 3.70 (m, 9H), 3.62 (dd, J = 33.2, 11.0 Hz, 1H), 2.42 (td, J = 11.6, 9.6, 5.3 Hz, 1H), 2.09 (m, 1H). ^{13}C-NMR (100 MHz, $CDCl_3$) δ 179.9, 179.7, 156.2, 155.4, 134.0, 133.7, 133.5, 133.4, 112.7, 112.5, 110.4, 110.2, 55.9, 54.4, 54.0, 53.8, 52.8, 52.6, 45.7,

45.2, 36.4, 35.5. IR (neat) 1693.6, 1452.3, 1389.3, 1194.9, 1128.09, 1034.32, 730.8 cm^{-1}; HRMS (CI$^+$) (m/z) *calcd.* for $C_{14}H_{16}N_2O_4$ [M+H]$^+$ 277.1180; found 277.1200

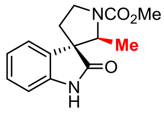

2m

2m: Following the **General Procudure-1**, reaction of **1m** (50 mg, 0.22 mmol) with KBr (2.2 mL, 0.01 M, 0.022 mmol) and oxone (80 mg, 0.26 mmol, MW 307) provided spirooxindole **2m** (44.1 mg, 77.0% yield). ^{1}H-NMR (400 MHz, $CDCl_3$) δ 9.17 (brs, 1H), 7.22 (t, J = 7.7 Hz, 1H), 7.10 (d, J = 7.5 Hz, 1H), 7.02 (t, J = 7.5 Hz, 1H), 6.93 (d, J = 7.7 Hz, 1H), 4.16 – 3.96 (m, 1H), 3.96 – 3.78 (m, 2H), 3.74 (s, 3H), 2.44 – 2.37 (m, 1H), 2.11(brs, 1H), 1.31 (d, J = 6.5 Hz, 3H).^{13}C-NMR (100 MHz, $CDCl_3$) δ 179.3, 178.8, 155.4, 140.5, 140.1, 132.9, 131.8, 128.4, 122.8, 122.6, 109.8, 61.3, 60.7, 56.5, 52.4, 45.1, 34.1, 33.2, 16.5, 15.2. IR (neat) 1681.0, 1616.9, 1454.2, 1382.5, 1336.3, 1189.8, 1108.9, 739.0 cm^{-1}; HRMS (CI$^+$) (m/z) *calcd.* for $C_{14}H_{16}N_2O_3$ [M]$^+$ 260.1161; found 260.1163.

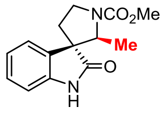

2m'

2m': Following the **General Procudure-1**, reaction of **1m** (50 mg, 0.22 mmol) with KBr (2.2 mL, 0.01 M, 0.022 mmol) and oxone (80 mg, 0.26 mmol, MW 307) provided spirooxindole **2m'** (11.5 mg, 20.1% yield). ^{1}H-NMR (400 MHz, $CDCl_3$) δ 8.39 (s, 1H), 7.28 – 7.21 (m, 2H), 7.05 (t, J = 7.3 Hz, 1H), 6.93 (d, J = 7.8 Hz, 1H), 4.17 (d, J = 7.0 Hz, 1H), 3.98 (s, 1H), 3.85 – 3.61 (m, 4H), 2.32 (ddd, J = 12.7, 8.3, 7.0 Hz, 1H), 2.15 (ddd, J = 13.0, 7.6, 5.6 Hz, 1H), 1.15 (d, J = 6.4 Hz, 3H). ^{13}C-NMR (100 MHz, $CDCl_3$) δ 180.1, 155.9, 140.7, 129.4, 128.5, 125.1, 122.4, 110.0, 59.2, 57.0, 52.4, 45.6, 33.8, 17.5. IR (neat) 3234.5, 1706.3, 1621.0, 1459.3, 1384.9, 753.8 cm^{-1}; HRMS (CI$^+$) (m/z) *calcd.* for $C_{14}H_{16}N_2O_3$ [M+H]$^+$ 260.1161; found 260.1165.

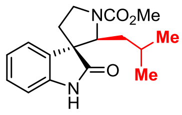

2n

2n[17]**:** Following the **General Procudure-1**, reaction of **1n** (100 mg, 0.35 mmol) with KBr (4.2 mg, 0.035 mmol) and oxone (129 mg, 0.42 mmol, MW 307) provided spirooxindole **2n** (72.1 mg, 68% yield). ^{1}H-NMR (400 MHz, $CDCl_3$) δ 8.47 (s, 1H), 7.23 (t, J = 8.3 Hz, 1H), 7.03 (t, J = 7.0 Hz, 2H), 6.91 (d, J = 7.7 Hz, 1H), 4.06 (d, J = 63.4 Hz, 1H), 3.94 – 3.58 (m, 5H), 2.49 (d, J = 32.5 Hz, 1H), 2.17 – 1.99 (m, 1H), 1.83 (brs, 2H), 1.30 (brs, 1H), 0.81 (d, J = 6.7 Hz, 6H). ^{13}C-NMR (100 MHz, $CDCl_3$) δ 179.3, 178.5, 155.6, 139.7, 134.3, 133.4, 128.1, 122.9, 122.3, 109.9, 63.5, 55.7, 52.4, 44.5, 40.1, 39.7, 35.4, 33.8, 25.1, 23.1, 22.3, 21.9. IR (neat) 2954.8, 1693.0, 1456.3, 1384.9, 1335.3, 1119.3, 749.5 cm^{-1}; HRMS (CI$^+$) (m/z) *calcd.* for $C_{17}H_{22}N_2O_3$ [M]$^+$ 302.1630; found 302.1634.

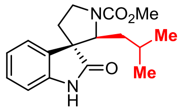

2n'

2n'[17]**:** Following the **General Procudure-1**, reaction of **1n** (100 mg, 0.35 mmol) with KBr (4.2 mg, 0.035 mmol) and oxone (129 mg, 0.42 mmol, MW 307) provided spirooxindole **2n'** (29.6 mg, 28.0% yield). ^{1}H-NMR (400 MHz, $CDCl_3$) δ 9.18 (s, 1H), 7.25 – 7.16 (m, 2H), 7.03 (t, J = 7.6 Hz, 1H), 6.95 (d, J = 7.7 Hz, 1H), 4.24 (brs, 1H), 4.01 (brs, 1H), 3.73 (s, 3H), 3.61 (dt, J = 11.1, 7.5 Hz, 1H), 2.30 (dt, J = 12.6, 8.0 Hz, 1H), 2.08 (ddd, J = 12.5, 7.3, 4.9 Hz, 1H), 1.34 (m, 1H), 1.12 – 1.01 (m, 1H), 0.94 – 0.86 (m, 1H), 0.83 (d, J = 6.5 Hz, 3H), 0.61 (d, J = 6.5 Hz, 3H). ^{13}C-NMR (100 MHz, $CDCl_3$) δ 181.0, 156.3, 140.8, 129.5, 128.4, 125.0, 122.1, 110.3, 61.3, 56.7, 52.4, 45.4, 40.8, 35.3, 24.6,

22.7, 22.0. IR (neat) 2954.4, 1681.5, 1453.7,1379.2, 1331.7, 1210.4, 1109.9, 732.1 cm^{-1}; HRMS (CI$^+$) (m/z) *calcd.* for $C_{17}H_{22}N_2O_3$ [M]$^+$ 302.1630; found 302.1622.

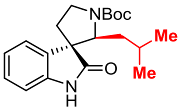

2o

(3*R**, 4*S**)-**2o:** Following the **General Procudure-1**, reaction of **1o** (66 mg, 0.2 mmol) with KBr (2.0 mL, 0.01 M, 0.02 mmol) and oxone (74 mg, 0.24 mmol, MW 307) provided spirooxindole **2o** (35.5 mg, 51.6%). ^{1}H-NMR (400 MHz, $CDCl_3$) δ 9.15 (s, 1H), 7.22 (t, *J* = 8.3 Hz, 1H), 7.02 (d, *J* = 6.5 Hz, 2H), 6.93 (d, *J* = 7.7 Hz, 1H), 4.25 – 3.57 (m, 3H), 2.67 – 2.32 (m, 1H), 2.06 – 1.96 (m, 1H), 1.87 (d, *J* = 15.7 Hz, 1H), 1.47 (s, 11H), 0.82 (d, *J* = 6.5 Hz, 6H). ^{13}C-NMR (100 MHz, $CDCl_3$) δ 178.4, 154.7, 139.4, 134.7, 128.1, 123.0, 122.6, 109.8, 80.2, 63.6, 55.7, 43.8, 40.1, 35.6, 34.0, 28.6, 25.1, 23.0, 22.7. IR (neat) 2960.6, 1696.2, 1620.1, 1470.7, 1397.5, 1335.9, 1240.8, 1169.1, 1119.5, 747.9 cm^{-1}; HRMS (CI$^+$) (m/z) *calcd.* for $C_{20}H_{28}N_2O_3$ [M]$^+$ 344.2100; found 344.2093. The relative configuration was confirmed by X-ray diffraction analysis (The Cambridge Crystallographic Data Centre, Deposition number 1935503) (Supplementary Figure 243, page 278).

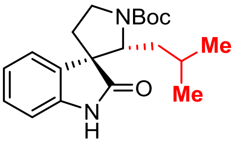

2o'

(3*R**, 4*R**)-**2o':** Following the **General Procudure-1**, reaction of **1o** (66 mg, 0.2 mmol) with KBr (2.0 mL, 0.01 M, 0.02 mmol) and oxone (74 mg, 0.24mmol, MW 307) provided spirooxindole **2o'** (24.1 mg, 35%). ^{1}H-NMR (400 MHz, $CDCl_3$) δ 8.53 (s, 1H), 7.30 – 7.26 (m, 2H), 7.11 – 7.04 (m, 1H), 6.96 (dd, *J* = 8.0, 1.1 Hz, 1H), 4.25 (s, 1H), 4.05 (s, 1H), 3.61 (ddd, *J* = 11.3, 8.6, 7.0 Hz, 1H), 2.35 (dt, *J* = 12.5, 8.5 Hz, 1H), 2.04 (ddd, *J* = 11.9, 6.9, 4.0 Hz, 1H), 1.74 (s, 2H), 1.54 (s, 9H), 1.45 – 1.32 (m, 1H), 1.03 (s, 1H), 0.87 (d, *J* = 6.5 Hz, 3H), 0.62 (d, *J* = 6.5 Hz, 3H). ^{13}C-NMR (100 MHz, $CDCl_3$) δ 180.5, 155.2, 140.4, 130.0, 128.3, 125.2, 122.2, 110.0, 61.1, 57.1, 45.7, 40.9, 35.8, 29.7, 28.5, 24.6, 23.3, 21.9. IR (neat) 2957.3, 2926.1, 1693.8, 1619.6, 1465.7, 1391.9, 1336.1, 1241.5, 1166.9, 1107.2, 1030.0, 744.6 cm^{-1}; HRMS (CI$^+$) (m/z) *calcd.* for $C_{20}H_{28}N_2O_3$ [M]$^+$ 344.2100; found 344.2103. The relative configuration was confirmed by X-ray diffraction analysis (The Cambridge Crystallographic Data Centre, Deposition number 1935504) (Supplementary Figure 244, page 279).

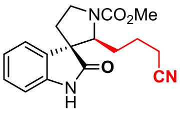

2p

2p: Following the **General Procudure-1**, reaction of **1p** (90 mg, 0.3 mmol) with KBr (3.6 mg, 0.03 mmol) and oxone (111 mg, 0.36 mmol, MW 307) provided spirooxindole **2p** (62.1 mg, 65.9% yield). ^{1}H-NMR (400 MHz, $CDCl_3$) δ 9.23 (s, 1H), 7.25 – 7.19 (m, 1H), 6.98 (m, 3H), 4.20 – 3.60 (m, 6H), 2.60 – 2.41 (m, 1H), 2.29 (t, *J* = 7.2 Hz, 2H), 2.13 – 1.79 (m, 3H), 1.71 – 1.43 (m, 2H). ^{13}C-NMR (100 MHz, $CDCl_3$) δ 178.4, 177.9, 155.7, 139.6, 133.8, 133.1, 128.4, 123.1, 122.2, 119.5, 110.1, 64.3, 64.0, 55.6, 55.3, 52.7, 44.6, 35.1, 33.8, 30.2, 22.4, 16.8. IR (neat) 2954.3, 1686.5, 1618.6, 1448.6, 1384.0, 1124.3, 728.4 cm^{-1}; HRMS (CI$^+$) (m/z) *calcd.* for $C_{17}H_{19}N_3O_3$ [M]$^+$ 313.1426; found 313.1435.

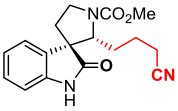

2p'

2p': Following the **General Procudure-1**, reaction of **1p** (90 mg, 0.3 mmol) with KBr (3.6 mg, 0.03 mmol) and oxone (111 mg, 0.36mmol, MW 307) provided spirooxindole **2p'** (18.3 mg, 19.5% yield). ^{1}H-NMR (400 MHz, $CDCl_3$) δ 8.74 (s, 1H), 7.33 – 7.19 (m, 2H), 7.07 (t, *J*

= 7.5 Hz, 1H), 6.96 (d, *J* = 7.8 Hz, 1H), 4.13 (brs, 1H), 4.02 (brs, 1H), 3.75 (s, 3H), 3.63 (m, 1H), 2.41 – 1.92 (m, 6H), 1.68 – 1.45 (m, 2H).^{13}C-NMR (100 MHz, $CDCl_3$) δ 180.3 , 180.3, 156.9, 141.0, 128.9 , 128.5, 125.0, 122.6, 119.5, 110.6, 62.3, 52.3, 45.4, 35.0, 31.5, 29.7, 21.9, 17.1. IR (neat) 2926.1, 1700.2, 1620.2, 1455.6, 1383.0, 755.5 cm^{-1}; HRMS (CI$^+$) (m/z) *calcd.* for $C_{17}H_{19}N_3O_3$ [M]$^+$ 313.1426; found 313.1428.

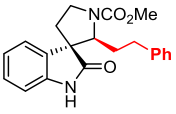

2q

2q: Following the **General Procudure-1**, reaction of **1q** (50 mg, 0.15 mmol) with KBr (1.5 mL, 0.01 M, 0.015 mmol) and oxone (83 mg, 0.27 mmol, MW 307) provided spirooxindole **2q** (18.2 mg, 34.7% yield). ^{1}H-NMR (400 MHz, $CDCl_3$) δ 8.49 (s, 1H), 7.25 – 7.10 (m, 4H), 7.05 (dd, *J* = 11.4, 6.2 Hz, 4H), 6.91 (d, *J* = 7.7 Hz, 1H), 4.14 – 3.69 (m, 6H), 2.84 (dt, *J* = 116.7, 7.8 Hz, 1H), 2.45 (s, 3H), 2.09 (dt, *J* = 13.1, 6.9 Hz, 2H). ^{13}C-NMR (100 MHz, $CDCl_3$) δ 178.7, 155.7, 141.6, 139.5, 128.6, 128.4, 128.3, 128.2, 125.8, 123.2, 122.6, 109.9, 66.0, 56.0, 52.6, 44.9, 35.7, 32.6, 30.8. IR (neat) 1694.9, 1615.6, 1454.2, 1384.4, 1336.7, 1186.6, 1123.1, 734.2, 696.7 cm^{-1}; HRMS (CI$^+$) (m/z) *calcd.* for $C_{21}H_{22}N_2O_3$ [M]$^+$ 350.1630; found 350.1628.

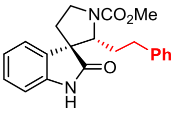

2q'

2q': Following the **General Procudure-1**, reaction of **1q** (50mg, 0.15mmol) with KBr (1.5 mL, 0.01 M, 0.015 mmol) and oxone (83 mg, 0.27 mmol, MW 307) provided spirooxindole **2q'** (11.8 mg, 22.5% yield). ^{1}H-NMR (400 MHz, $CDCl_3$) δ 8.10 (s, 1H), 7.35 – 7.26 (m, 2H), 7.16 (t, *J* = 7.5 Hz, 3H), 7.09 (q, *J* = 7.5, 7.0 Hz, 2H), 6.97 (d, *J* = 7.8 Hz, 1H), 6.89 – 6.78 (m, 2H), 4.23 (brs, 1H), 4.05 (brs, 1H), 3.74 (s, 3H), 3.66 (m, 2H), 2.35 (m, 3H), 2.13 – 2.02 (m, 2H). ^{13}C-NMR (100 MHz, $CDCl_3$) δ 179.7, 156.8, 140.9, 140.0, 128.7, 128.0, 127.7, 127.6, 125.3, 124.7, 121.9, 109.7, 62.6, 55.9, 51.9, 45.2, 35.0, 33.4, 31.5. IR (neat) 1681.0, 1614.6, 1449.9, 1380.8, 1326.5, 1114.9, 736.5, 695.9 cm^{-1}; HRMS (CI$^+$) (m/z) *calcd.* for $C_{21}H_{22}N_2O_3$ [M]$^+$ 350.1630; found 350.1635.

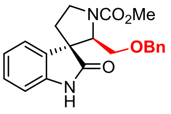

2r

2r: Following the **General Procudure-1**, reaction of **1r** (83 mg, 0.24 mmol) with KBr (2.4 mL, 0.01 M, 0.024 mmol) and oxone (133 mg, 0.43 mmol, MW 307) provided spirooxindole **2r** (46.2 mg, 53.2% yield). ^{1}H-NMR (400 MHz, $CDCl_3$) δ 8.07/8.00 (s, 1H), 7.25 – 7.16 (m, 4H), 7.11 (s, 2H), 7.03 (td, *J* = 7.5, 1.1 Hz, 2H), 6.80 (d, *J* = 7.7 Hz, 1H), 4.34 (m, 2H), 4.10 (brd, *J* = 49.5 Hz, 1H), 3.96 – 3.69 (m, 7H), 2.59 – 2.36 (m, 1H), 2.09 (m, 1H). ^{13}C-NMR (100 MHz, $CDCl_3$) δ 178.7, 155.4, 139.9, 138.2, 134.0, 133.6, 128.0, 127.2, 122.8, 122.3, 109.7, 72.7, 68.2, 64.1, 54.8, 52.6, 45.6, 35.9, 34.7. IR (neat) 1689.01, 1616.5, 1452.5, 1382.3, 1196.7, 1010.0, 734.5 cm^{-1}; HRMS (CI$^+$) (m/z) *calcd.* for $C_{21}H_{22}N_2O_4$ [M]$^+$366.1580; found 366.1587.

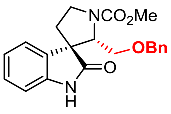

2r'

2r': Following the **General Procudure-1**, reaction of **1r** (83 mg, 0.24 mmol) with KBr (2.4 mL, 0.01 M, 0.024 mmol) and oxone (133 mg, 0.43 mmol, MW 307) provided spirooxindole **2r'** (25.9 mg, 29.8% yield). ^{1}H-NMR (400 MHz, $CDCl_3$) δ 8.34 (brs, 1H), 7.15 – 7.06 (m, 2H), 7.00 (t, *J* = 7.6 Hz, 1H), 6.88 (d, *J* = 7.8 Hz, 1H), 4.38 (d, *J* = 12.1 Hz, 1H), 4.21 (s, 2H), 3.96 (s, 1H), 3.83 (s, 1H), 3.72 (s, 4H), 3.54 (s, 1H), 2.26 (t, *J* = 7.4 Hz, 2H). ^{13}C-NMR (100 MHz, $CDCl_3$) δ 180.5, 155.8, 141.2, 137.9, 131.3, 129.2, 128.4, 128.2, 127.4, 127.2, 124.9, 122.2, 109.9, 73.2, 69.1, 62.3, 56.0, 52.5,

46.0, 34.9. IR (neat) 2922.4, 1705.2, 1455.6, 1383.6, 1198.3, 1102.3 cm^{-1}; HRMS (CI^+) (m/z) *calcd.* for $C_{21}H_{22}N_2O_4$ $[M]^+$366.1580; found 366.1593.

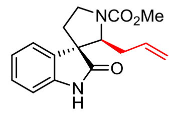

2s

2s: Following the **General Procudure-1**, reaction of **1s** (54 mg, 0.2 mmol) with KBr (2.0 mL, 0.01 M, 0.02 mmol) and oxone (74 mg, 0.24 mmol, MW 307) provided spirooxindole **2s** (40.7 mg, 71.1% yield). ^{1}H-NMR (400 MHz, $CDCl_3$) δ 8.72 (brs, 1H), 7.21 (t, *J* = 7.6 Hz, 1H), 7.03 (m, 2H), 6.88 (d, *J* = 7.7 Hz, 1H), 5.57 (brs, 1H), 4.98 (dd, *J* = 17.1, 1.9 Hz, 1H), 4.83 (brd, *J* = 31.9 Hz, 1H), 4.23 – 3.98 (m, 1H), 3.84 (brs, 2H), 3.75 (s, 3H), 2.95 – 2.31 (m, 3H), 2.14 – 2.02 (m, 1H), 1.89 (s, 1H). ^{13}C-NMR (100 MHz, $CDCl_3$) δ 179.0, 155.5, 139.8, 134.1, 133.0, 128.3, 122.9, 122.4, 117.1, 109.8, 64.4, 55.7, 52.5, 44.9, 35.7, 35.0. IR (neat) 2953.32, 1685.7, 1620.6, 1454.6, 1385.5, 1340.2, 1192.4, 1124.2, 743.0 cm^{-1}; HRMS (CI^+) (m/z) *calcd.* for $C_{16}H_{18}N_2O_3$ $[M]^+$286.1317; found 286.1313.

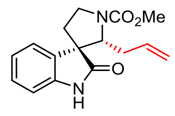

2s'

2s': Following the **General Procudure-1**, reaction of **1s** (54 mg, 0.2 mmol) with KBr (2.0 mL, 0.01 M, 0.02 mmol) and oxone (74 mg, 0.24 mmol, MW 307) provided spirooxindole **2s'** (13.2 mg, 23.1% yield). ^{1}H-NMR (400 MHz, $CDCl_3$) δ 8.40 (s, 1H), 7.30 – 7.22 (m, 2H), 7.06 (t, *J* = 7.5 Hz, 1H), 6.91 (d, *J* = 7.7 Hz, 1H), 5.46 – 5.37 (m, 1H), 4.73 (d, *J* = 10.1 Hz, 1H), 4.48 (d, *J* = 17.0 Hz, 1H), 4.22 (dd, *J* = 10.0, 5.0 Hz, 1H), 4.02 (brs, 1H), 3.75 (s, 3H), 3.67 (m, 1H), 2.40 – 2.27 (m, 1H), 2.15 – 2.02 (m, 1H), 1.97 (ddd, *J* = 12.4, 6.7, 3.1 Hz, 1H), 1.82 (brs, 1H). ^{13}C-NMR (100 MHz, $CDCl_3$) δ 180.1, 156.2, 140.9, 133.0, 129.8, 128.5, 124.9, 122.2, 117.7, 110.2, 62.5, 61.8, 57.0, 52.5, 46.2, 35.7, 29.7. IR (neat) 2924.9, 1705.5, 1456.3, 1384.3, 1215.9, 1118.7, 754.6 cm^{-1}; HRMS (CI^+) (m/z) *calcd.* for $C_{16}H_{18}N_2O_3$ $[M]^+$286.1317; found 286.1310.

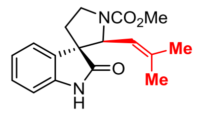

2t

2t: Following the **General Procudure-1**, reaction of **1t** (100 mg, 0.35 mmol) with KBr (4.2 mg, 0.035 mmol) and oxone (130 mg, 0.42 mmol, MW 307) provided spirooxindole **2t** (47.3 mg, 45.0% yield). ^{1}H-NMR (400 MHz, $CDCl_3$) δ 8.91 (s, 1H), 7.19 (q, *J* = 7.3, 6.9 Hz, 2H), 7.02 (t, *J* = 7.5 Hz, 1H), 6.87 (d, *J* = 7.7 Hz, 1H), 5.24 (d, *J* = 9.4 Hz, 1H), 4.72 (brs, 1H), 4.00 (q, *J* = 8.7, 8.2 Hz, 1H), 3.87 (s, 1H), 3.69 (s, 3H), 2.34 (s, 1H), 2.18 (s, 1H), 1.59 (s, 3H), 1.27 (s, 3H). ^{13}C-NMR (100 MHz, $CDCl_3$) δ 178.5, 154.9, 140.2, 135.2, 130.6, 127.8, 122.1, 122.0, 120.4, 109.2, 62.8, 57.2, 51.8, 45.1, 33.6, 25.2, 17.2. IR (neat) 1688.1, 1618.7, 1454.5, 1383.5,1339.6,1188.2, 1125.2, 911.1, 730.0 cm^{-1}; HRMS (CI^+) (m/z) *calcd.* for $C_{17}H_{20}N_2O_3$ $[M]^+$300.1474; found 300.1467.

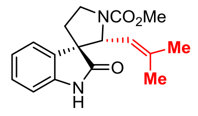

2t'

2t': Following the **General Procudure-1**, reaction of **1t** (100 mg, 0.35 mmol) with KBr (4.2 mg, 0.035 mmol) and oxone (130 mg, 0.42 mmol, MW 307) provided spirooxindole **2t'** (15.8 mg, 15.0% yield). ^{1}H-NMR (400 MHz, $CDCl_3$) δ 8.41 (s, 1H), 7.21 (t, *J* = 7.7 Hz, 1H), 7.13 (d, *J* = 7.5 Hz, 1H), 6.99 (t, *J* = 7.6 Hz, 1H), 6.87 (d, *J* = 7.8 Hz, 1H), 5.00 (d, *J* = 8.6 Hz, 1H), 4.79 (d, *J* = 8.0 Hz, 1H), 3.97 (m, 1H), 3.77 – 3.64 (m, 4H), 2.31 (ddd, *J* = 13.5, 8.1, 5.8 Hz, 1H), 2.17 (dt, *J* = 12.8, 7.4 Hz, 1H), 1.58 (s, 3H), 1.32 (s, 3H). ^{13}C-NMR (100 MHz, $CDCl_3$) δ 179.6, 155.2, 140.1, 135.7, 128.5, 127.6, 124,7, 121.3, 121.4, 109.0, 60.8, 56.6, 51.8, 44.7, 33.2, 29.1, 25.0, 17.2. IR (neat) 2922.6, 1700.4,

1457.2, 1385.1, 1195.3, 1120.1. 748.8 cm^{-1}; HRMS (CI$^+$) (m/z) *calcd.* for $C_{17}H_{20}N_2O_3$ [M]$^+$300.1474; found 300.1480.

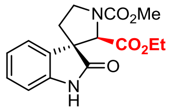

2u

2u: Following the **General Procudure-1**, reaction of **1u** (30 mg, 0.1 mmol) with KBr (1.0 mL, 0.01 M, 0.01 mmol) and oxone (37 mg, 0.12 mmol, MW 307) provided spirooxindole **2s** (25.5 mg, 80% yield, *dr* > 20:1). ^{1}H-NMR (400 MHz, $CDCl_3$) δ 8.28 (s, 1H), 7.56 (d, *J* = 7.9 Hz, 1H), 7.35 (d, *J* = 8.2 Hz, 1H), 7.23 (t, *J* = 7.6 Hz, 1H), 7.14 (t, *J* = 7.5 Hz, 1H), 5.26 (s, 1H), 4.37 – 4.16 (m, 2H), 4.09 (m, 1H), 3.81 (s, 3H), 3.51 (s, 1H), 2.97 – 2.83 (m, 2H), 1.63 (s, 1H), 1.17 (t, *J* = 7.1 Hz, 3H).^{13}C-NMR (100 MHz, $CDCl_3$) δ 170.7, 156.3, 136.1, 128.1, 125.4, 122.6, 119.2, 118.4, 111.9, 111.0, 81.9, 62.3, 52.8, 40.3, 20.2, 13.9. IR (neat) 3355.2, 2947.5, 1690.6, 1523.9, 1445.6, 1378.4, 1297.1, 1213.1, 1074.1, 1012.0, 739.8 cm^{-1}; HRMS (CI$^+$) (m/z) *calcd.* for $C_{16}H_{18}N_2O_5$ [M]$^+$318.1216; found 318.1212.

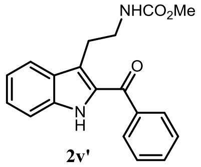

2v'

2v': Following the **General Procudure-1**, reaction of **1v** (200 mg, 0.65 mmol) with KBr (6.5 mL, 0.01 M, 0.01 mmol) and oxone (167 mg, 0.54 mmol, MW 307) provided spirooxindole **2v** (0% yield) but C-2 aroyl indole **2v'** (74mg, 59.7% yield) and **1v** (84 mg) was recovered. ^{1}H-NMR (400 MHz, $CDCl_3$) δ 9.35/ 8.92 (s, 1H), 7.82 – 7.31 (m, 8H), 7.24 – 7.12 (m, 1H), 5.29 (d, J = 4.5 Hz, 1H), 3.59 (s, 3H), 3.42 (q, J = 6.4 Hz, 2H), 3.13 (t, J = 6.8 Hz, 2H).^{13}C-NMR (100 MHz, $CDCl_3$) δ 188.99 , 156.96 , 138.68 , 136.55 , 132.29 , 131.58 , 128.88 , 128.53 , 127.90 , 126.38 , 122.23 , 121.00 , 120.67 , 112.15 , 51.81 , 41.95 , 25.01 . IR (neat) 3305.8, 2924.2, 1696.6, 1625.5, 1523.6, 1440.2, 1322.9, 1252.0, 1144.8, 1076.3, 735.4, 694.7 cm^{-1}; HRMS (CI$^+$) (m/z) *calcd.* for $C_{19}H_{18}N_2O_3$ [M]$^+$ 322.1317; found 322.1322.

Possible mechanisms for the formation of 2v'

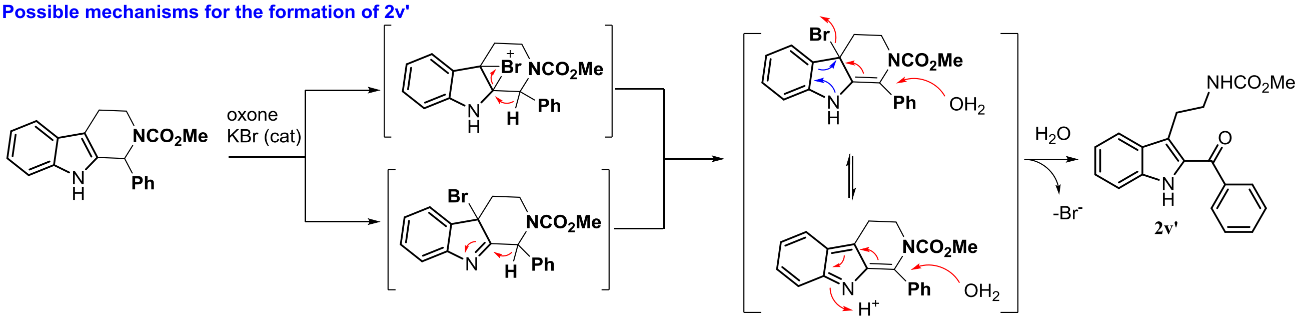

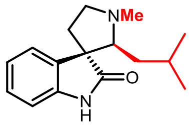

2w

2w: Following the **General Procudure-2**, reaction of **1w** (49 mg, 0.2 mmol) with KBr (2.0 mL, 0.01 M, 0.02 mmol) and oxone (111 mg, 0.36 mmol, MW 307) provided spirooxindole **2w** (11.2 mg, 32.1% yield) and **1w** (16 mg) was recovered; only the major diastereomer was obtained and ^{1}H-NMR of crude reaction mixture determined *dr* = 3.5:1) as light yellow solid. ^{1}H-NMR (400 MHz, $CDCl_3$) δ 8.57 (s, 1H), 7.51 (d, *J* = 7.5 Hz, 1H), 7.20 (t, *J* = 7.3 Hz, 1H), 7.05 (t, *J* = 7.5 Hz, 1H), 6.89 (d, *J* = 7.7 Hz, 1H), 3.50 – 3.36 (m, 1H), 2.84 (s, 1H), 2.71 (q, *J* = 9.3 Hz, 1H), 2.50 (s, 3H), 2.34 (ddd, *J* = 10.7, 6.9, 2.0 Hz, 1H), 2.13 (m, 1H), 1.32 – 1.26 (m, 2H), 0.93 (m, 1H), 0.74 (d, *J* = 6.4 Hz, 3H), 0.52 (d, *J* = 6.5 Hz, 3H). ^{13}C-NMR (100 MHz, $CDCl_3$) δ 182.3, 140.1, 132.5, 127.9, 125.7, 122.7, 109.6, 71.6, 57.0, 55.9, 40.3, 38.2, 36.6, 24.7, 23.7, 21.7. IR (neat) 2950.9, 1702.2, 1616.8, 1465.8, 1340.1, 1187.6, 741.8, 674.0, 616.5 cm^{-1}; HRMS (CI$^+$) (m/z) *calcd.* for $C_{16}H_{26}N_2O$ [M]$^+$258.1732; found 258.1738.

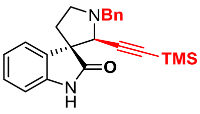

2x

2x: Following the **General Procudure-2**, reaction of **1x** (100mg, 0.28mmol) with KBr (2.8 mL, 0.01 M, 0.028 mmol) and oxone (155 mg, 0.50 mmol, MW 307) provided spirooxindole **2x** (33.8 mg, 45.1% yield, **1x** (27.5 mg) was recovered; only the major diastereomer was obtained and ^{1}H-NMR of crude reaction mixture determined *dr* = 3.6:1). ^{1}H-NMR (400 MHz, $CDCl_3$) δ 7.88 (s, 1H), 7.52 (d, *J* = 7.4 Hz, 1H), 7.46 – 7.31 (m, 4H), 7.29 (d, *J* = 7.3 Hz, 1H), 7.21 (t, *J* = 7.7 Hz, 1H), 7.07 (t, *J* = 7.6 Hz, 1H), 6.86 (d, *J* = 7.7 Hz, 1H), 4.34 (d, *J* = 13.1 Hz, 1H), 3.61 (s, 1H), 3.35 (d, *J* = 13.1 Hz, 1H), 3.11 (d, *J* = 9.2 Hz, 1H), 2.57 – 2.44 (m, 1H), 2.41 – 2.31 (m, 1H), 2.07 (q, *J* = 9.1 Hz, 1H), -0.10 (s, 9H). ^{13}C-NMR (100 MHz, $CDCl_3$) δ 181.0, 140.4, 138.7, 133.9, 128.9, 128.2, 127.8, 127.0, 125.3, 122.3, 109.2, 101.5, 92.7, 91.8, 64.7, 57.4, 57.2, 51.4, 34.4, 34.3. -0.4. IR (neat) 2958.4, 1710.0, 1617.8, 1470.1, 1337.6, 1247.2, 847.18, 751.0 cm^{-1}; HRMS (CI^+) (m/z) *calcd.* for $C_{22}H_{26}N_2OSi$ $[M+H]^+$375.1893; found 375.1886.

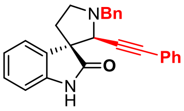

2y

2y: Following the **General Procudure-2**, reaction of **1y** (111 mg, 0.31 mmol) with KBr (3.1 mL, 0.01 M, 0.031 mmol) and oxone (171 mg, 0.56 mmol, MW 307) provided spirooxindole **2y** (55 mg, 47% yield; only the major diastereomer was obtained and ^{1}H-NMR of crude reaction mixture determined *dr* = 2.2:1). ^{1}H-NMR (400 MHz, $CDCl_3$) δ 8.80 (s, 1H), 7.63 (d, *J* = 7.4 Hz, 1H), 7.46 (d, *J* = 7.5 Hz, 2H), 7.37 (t, *J* = 7.4 Hz, 2H), 7.33 – 7.27 (m, 1H), 7.16 (tt, *J* = 13.9, 7.3 Hz, 5H), 6.97 (dd, *J* = 21.4, 7.5 Hz, 3H), 4.44 (d, *J* = 13.0 Hz, 1H), 3.87 (s, 1H), 3.43 (d, *J* = 13.1 Hz, 1H), 3.18 (d, *J* = 9.0 Hz, 1H), 2.58 (m, 1H), 2.44 (ddd, *J* = 12.5, 9.1, 2.9 Hz, 1H), 2.13 (m, 1H). ^{13}C-NMR (100 MHz, $CDCl_3$) δ 180.8, 140.4, 138.6, 134.1, 131.7, 128.9, 128.3, 128.1, 128.0, 127.9, 127.0, 125.3, 122.5, 109.3, 86.9, 85.0, 64.8, 57.6, 57.4, 51.5, 34.4. IR (neat) 2923.1, 2856.1, 2354.5, 1710.0, 1618.1, 1469.3,1341.8, 751.0, 692.6 cm^{-1}; HRMS (CI^+) (m/z) *calcd.* for $C_{26}H_{22}N_2O$ $[M]^+$378.1732; found 378.1723.

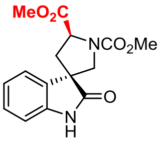

2z

2z: Following the **General Procudure-1**, reaction of **1z** (82 mg, 0.28 mmol) with KBr (2.8 mL, 0.01 M, 0.028 mmol) and oxone (105 mg, 0.34 mmol, MW 307) provided spirooxindole **2z** (51.1 mg, 60.0% yield). ^{1}H-NMR (400 MHz, $CDCl_3$) δ 9.54/9.51 (s, 1H), 7.27 (td, *J* = 7.5, 6.5, 3.0 Hz, 1H), 7.16 – 6.95 (m, 3H), 4.75 (dt, *J* = 31.2, 8.4 Hz, 1H), 3.90 – 3.73 (m, 8H), 2.59 (ddd, *J* = 15.9, 12.8, 8.6 Hz, 1H), 2.44 (td, *J* = 13.4, 8.1 Hz, 1H). ^{13}C-NMR (100 MHz, $CDCl_3$) δ 178.2, 177.9, 171.9, 171.8, 155.3, 154.6, 140.0, 139.2, 132.7, 132.7, 128.9, 128.7, 125.4, 123.0, 122.2, 110.4, 110.2, 58.9, 58.5, 55.3, 54.9, 53.0, 53.0, 52.9, 52.5, 52.1, 40.4, 39.4. IR (neat) 706.9, 1619.6, 1456.7, 1389.3, 1204.0, 754.3 cm^{-1}; HRMS (CI^+) (m/z) *calcd.* for $C_{15}H_{16}N_2O_5$ $[M]^+$304.1059; found 304.1054.

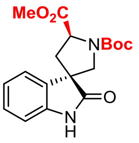

2aa

2aa[18]**:** Following the **General Procudure-1**, reaction of **1aa** (160 mg, 0.48 mmol) with KBr (4.8 mL, 0.005 M, 0.024 mmol) and oxone (177 mg, 0.58mmol, MW 307) provided spirooxindole **2aa** (136.0 mg, 81.8% yield). ^{1}H-NMR (400 MHz, $CDCl_3$) δ 8.81 (dd, *J* = 38.5, 13.4 Hz, 1H), 7.28–7.22 (m, 1H), 7.15 – 7.08 (m, 1H), 7.04 (tdd, *J* = 7.4, 2.2, 1.0 Hz, 1H), 6.95 (dd, *J* = 7.8, 4.3 Hz, 1H), 4.68 (dt, *J* = 44.3, 8.7 Hz, 1H), 3.84 (dd, *J* = 10.6, 5.2 Hz, 1H), 3.81 – 3.67 (m, 4H), 2.56 (ddd, *J* = 14.9, 12.8, 9.1 Hz, 1H), 2.38 (m, 1H), 1.46 (d, *J* = 11.5 Hz, 9H). ^{13}C-NMR (100 MHz, $CDCl_3$) δ 177.9, 172.4, 172.1,

154.2, 153.3, 139.7, 133.2, 133.0, 128.7, 123.2, 123.1, 122.4, 110.6, 80.9, 59.0, 58.6, 55.3, 54.7, 53.0, 52.5, 52.3, 52.2, 40.5, 39.6, 28.3. IR (neat) 1712.1, 1620.9, 1472.6, 1402.4, 1367.7, 1345.2, 1166.3, 1134.2, 751.8 cm^{-1}; HRMS (CI$^+$) (m/z) *calcd.* for $C_{18}H_{22}N_2O_5$ [M]$^+$346.1529; found 346.1527.

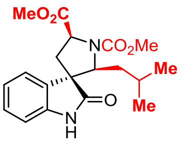

2ab

2ab: Following the **General Procudure-1**, reaction of **1ab** (76 mg, 0.22 mmol) with KBr (2.2 mL, 0.01 M, 0.022 mmol) and oxone (81 mg, 0.26 mmol, MW 307) provided spirooxindole **2ab** (71.2 mg, 99.5% yield, *dr* > 20:1). ^{1}H-NMR (400 MHz, $CDCl_3$) δ 9.34 (brs, 1H), 7.26 – 7.18 (m, 2H), 7.00 (t, J = 7.5 Hz, 1H), 6.92 (d, J = 7.7 Hz, 1H), 4.90 (t, J = 8.7 Hz, 1H), 4.25 (s, 1H), 3.77 (s, 3H), 3.71 (s, 3H), 2.56 (dd, J = 13.4, 8.7 Hz, 1H), 2.44 (dd, J = 13.4, 8.9 Hz, 1H), 1.72 (d, J = 9.4 Hz, 1H), 1.67 – 1.34 (m, 2H), 0.91 (d, J = 6.4 Hz, 3H), 0.81 (s, 3H).^{13}C-NMR (100 MHz, $CDCl_3$) δ 181.4, 173.3, 156.4, 141.4, 128.8, 126.8, 125.5, 122.0, 110.3, 61.4, 58.2, 55.6, 52.8, 52.4, 41.7, 37.3, 24.4, 23.3, 21.5. IR (neat) 2953.2, 1704.2, 1619.5, 1447.6, 1377.3, 1260.9, 1182.8, 1117.4, 1035.9, 743.2 cm^{-1}; HRMS (CI$^+$) (m/z) *calcd.* for $C_{19}H_{24}N_2O_5$ [M]$^+$360.1685; found 360.1684. $[\alpha]_D^{25}$= + 17.0 (*c* 1.0, $CHCl_3$)

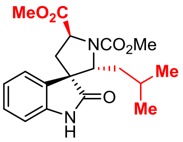

2ac

2ac Following the **General Procudure-1**, reaction of **1ac** (35 mg, 0.1mmol) with KBr (1.0 mL, 0.01 M, 0.01 mmol) and oxone (55 mg, 0.18 mmol, MW 307) provided spirooxindole **2ac** (36 mg, 98.3% yield, *dr* = 7:1). ^{1}H-NMR (400 MHz, CDCl3) δ 9.05 (d, J = 23.0 Hz, 1H), 7.57 – 7.30 (m, 1H), 7.22 (dd, J = 7.8, 1.2 Hz, 1H), 7.05 (td, J = 7.6, 1.0 Hz, 1H), 6.92 (d, J = 7.7 Hz, 1H), 4.82 (t, J = 8.3 Hz, 1H), 4.22 (m, 1H), 3.77-3.66 (m, 7H), 2.74 – 2.52 (m, 1H), 2.22 (dd, J = 13.2, 8.4 Hz, 1H), 2.06 (m 2H), 0.71 – 0.57 (m, 6H).^{13}C-NMR (100 MHz, $CDCl_3$) δ 179.5, 179.1, 173.1, 172.5, 155.0, 154.4, 140.4, 140.1, 130.0, 129.9, 128.8, 128.7, 124.3, 123.4, 123.0, 122.7, 110.5, 110.1, 64.6, 60.4, 59.3, 59.0, 56.4, 52.4, 39.4, 38.3, 25.2, 23.3, 21.7, 14.1. IR (neat) 2955.4, 1702.8, 1618.2, 1447.9, 1350.2, 1262.4, 1194.3, 1110.2, 953.8, 732.8 cm^{-1}; HRMS (CI$^+$) (m/z) *calcd.* for $C_{19}H_{24}N_2O_5$ [M]$^+$360.1685; found 360.1686.

Total Synthesis of (±)-Coerusecine and (±)-Horsfiline and One-pot Bromination

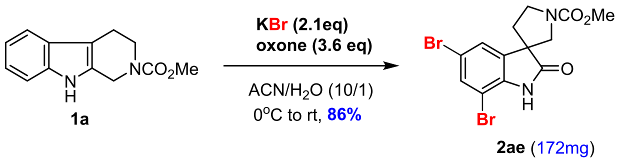

Oxidative rearrangement and Dibromination: To a solution of THCs **1a** (115 mg, 0.5 mmol) in MeCN/H_2O (1/1) (5.0 mL, 0.1 M) at 0 °C were added KBr (6 mg, 0.05 mmol) and oxone (184 mg, 0.6 mmol). The resulting solution was stirred at rt for 30 min. To the reaction mixture were added KBr (50 mg, 0.5mmol) and oxone (184 mg, 0.6 mmol). The resulting reaction mixture was stirred for 1h and then another batch of KBr (50 mg, 0.5 mmol) and oxone (184 mg, 0.6 mmol) were added. After stirring for 12h, the reaction was quenched by addition of *aq. sat.* $NaHCO_3$ (10 mL) and *aq. sat.* Na_2SO_3 (10 mL) and then diluted with EtOAc (20 mL). The organic fractions were collected, and the aqueous phase was extracted with EtOAc (3 × 20 mL). The combined organic fractions were washed with brine, dried over Na_2SO_4, filtered, and concentrated under reduced pressure. The

resulting residue was purified by flash column chromatography with gradient eluents (MeOH/CH_2Cl_2 1:100 to 1:10) to provide dibromospirooxindole **2ae (**172 mg) in 86% l. ^{1}H-NMR (400 MHz, $CDCl_3$) δ 9.34/9.29 (s, 1H), 7.51 (d, *J* = 1.7 Hz, 1H), 7.19 (d, *J* = 10.2 Hz, 1H), 3.90 – 3.53 (m, 7H), 2.43 (m, 1H), 2.09 (m, 1H).^{13}C-NMR (100 MHz, $CDCl_3$) δ 178.4, 178.1, 155.2, 139.1, 139.0, 135.2, 135.0, 133.4, 124.7, 115.6, 103.6, 54.7, 54.2, 53.7, 52.9, 45.4, 44.9, 36.3, 35.4. IR (neat): 1772.4, 1460.2, 1392.9, 754.1; HRMS (CI^+) (m/z) *calcd.* for $C_{13}H_{12}Br_2N_2O_3$ $[M]^+$ 401.9215; found 401.9199.

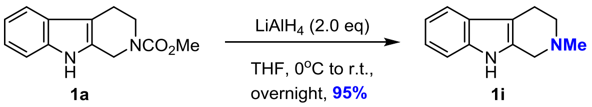

Preparation of THC 1i ($LiAlH_4$ Reduction) Error! Bookmark not defined.**:** To a solution of THC **1a** (270 mg, 1.17 mmol) in THF (12 ml, 0.1M) at 0 °C was added LiAlH4 powder (88 mg, 2.34 mmol) in three portions. The reaction mixture was warmed to rt and stirred for overnight. The reaction was quenched by dropwise addition of H2O (0.5 mL) at 0 °C followed by sequential addition of aq. NaOH (3 N, 1.0 mL), H2O (1.0 mL) and MgSO4 (~ 300 mg). The resulting mixture was stirred for 1.5 h and then added H2O (10 mL) and diluted with EtOAc (10 mL). The organic fractions were collected, and the aqueous phase was extracted with EtOAc (3 × 10 mL). The combined organic fractions were washed with brine, dried over Na_2SO_4, filtered, and concentrated under reduced pressure. The resulting residue was purified by flash column chromatography with gradient eluents (MeOH/ CH_2Cl_2= 1:50 to 1:10) to provide THC **1i**[4] (208 mg) in 95% yield.

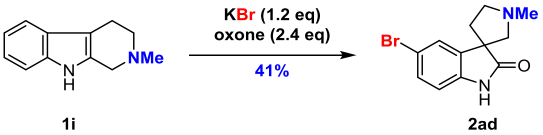

Preparation of compound 2ad (Oxidative rearrangement and Bromination)

To a solution of THCs **1i** (100 mg, 0.54 mmol) in THF/H_2O/AcOH(1/1/1) (5.4 mL, 0.1 M) at 0 °C were added KBr (3 mg, 0.05 mmol) and oxone (198 mg, 0.65 mmol, MW 307). The resulting solution was stirred at rt for overnight. To the reaction mixture were added KBr (70 mg, 0.60 mmol) and Oxone (198 mg, 0.65 mmol). After stirring for 12h, the reaction was quenched by addition of *aq. sat.* $NaHCO_3$ (10 mL) and *aq. sat.* Na_2SO_3 (10 mL) and then diluted with EtOAc (20 mL). The organic fractions were collected, and the aqueous phase was extracted with EtOAc (3 × 20 mL). The combined organic fractions were washed with brine, dried over Na_2SO_4, filtered, and concentrated under reduced pressure. The resulting residue was purified by flash column chromatography with gradient eluents (MeOH/CH_2Cl_2 1:100 to 1:10) to give bromospirooxindole **2ad**[18] **(**62 mg) in 41% yield. ^{1}H-NMR (400 MHz, $CDCl_3$) δ 9.42 (s, 1H), 7.53 (s, 1H), 7.34 – 7.27 (m, 1H), 6.80 (d, *J* = 8.2 Hz, 1H), 3.03 (td, *J* = 8.1, 4.7 Hz, 1H), 2.85 (s, 2H), 2.76 (q, *J* = 8.0 Hz, 1H), 2.46 (s, 3H), 2.40 (ddd, *J* = 12.8, 7.9, 4.8 Hz, 1H), 2.09 (dt, *J* = 13.0, 7.4 Hz, 1H).^{13}C-NMR (100 MHz, $CDCl_3$) δ 182.7 , 139.2, 138.3, 130.6, 126.5, 115.4, 111.2, 66.1, 56.5, 53.9, 41.6, 37.7. IR (neat) 2941.8, 2840.4, 2786.7, 1702.8, 1614.9, 1469.7, 1312.1, 1235.6, 1193.2, 1159.1, 907.0, 812.7, 728.6 cm^{-1}; HRMS (CI+) (m/z) *calcd.* for $C_{12}H_{13}BrN_2O$ [M]+ 280.0211; found 280.0210.

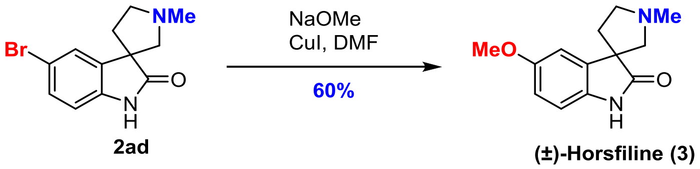

Synthesis of (±) - Horsfiline 4 (Ullmann ether synthesis)[18]**:**To a solution of **2ad** (28 mg, 0.1 mmol) and CuI (38 mg, 0.2 mmol, 2.0 equiv) in DMF (0.5 ml) were added NaOMe solution (4.0 mL, 0.75 M MeOH, 3.0 mmol). The resulting mixture was heated to 120 °C. After the reaction was completed as determined by TLC analysis, the reaction mixture was cooled to rt and the insoluble precipitates were filtered off through celite. The filtrate was collected and concentrated under reduced pressure. The residue was dissolved in CH_2Cl_2 (5.0 mL) and H_2O (5.0 mL). The organic fractions were collected, and the aqueous phase was extracted with CH_2Cl_2 (3 × 10 mL). The combined organic fractions were washed with brine, dried over Na_2SO_4, filtered, and concentrated under reduced pressure. The resulting residue was purified by flash column chromatography with gradient eluents ($MeOH/CH_2Cl_2$ 1: 100 to 1:10) to provide (±)-horsfiline **3**[18,19] **(**13.9 mg) in 60% yield. ^{1}H-NMR (400 MHz, $CDCl_3$) δ8.01 (brs, 1H), 7.16 (s, 1H), 6.78 (d, *J* = 8.5 Hz, 1H), 6.74 (dd, *J* = 8.4, 2.5 Hz, 1H), 3.80 (s, 3H), 3.21 (brs, 1H), 3.11 (d, *J* = 9.7 Hz, 1H), 2.94 (d, *J* = 9.8 Hz, 1H), 2.90 (m, 1H), 2.57 (s, 3H), 2.41 (ddd, *J* = 13.0, 7.3, 3.8 Hz, 1H), 2.22 (m, 1H). ^{13}C-NMR (100 MHz, $CDCl_3$) δ 182.8, 156.2, 136.9, 133.5, 112.7, 110.3, 110.0, 65.9, 56.6, 55.9, 54.1, 41.7, 38.0. IR (neat) 2929.5, 2843.3, 2787.8, 1698.9, 1604.6, 1482.5, 1304.3, 1200.9, 1031.9, 810.3, 732.5, 680.4 cm-1; HRMS (CI+) (m/z) *calcd.* for $C_{13}H_{16}N_2O_2$ [M]+ 232.1212; found 232.1216.

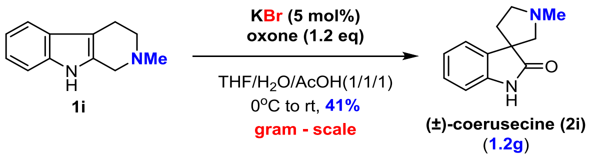

Synthesis of (±) - coerusecine (2i) in gram scale: To a solution of THCs **1i** (2.7 g, 14.5 mmol) in $THF/H_2O/AcOH$(1/1/1) (70 mL) at 0° C were added KBr (86 mg, 0.73 mmol) and oxone (5.3 g, 17.4 mmol) in three portions. After completion of the addition, the resulting reaction mixture was warmed to rt and stirred for overnight. The reaction was quenched by addition of *aq. sat.* $NaHCO_3$ (70mL) and *aq. sat.* Na_2SO_3 (70mL) and then diluted with EtOAc (100 mL). The organic fractions were collected, and the aqueous phase was extracted with EtOAc (3 × 70mL). The combined organic fractions were washed with brine, dried over Na_2SO_4, filtered, and concentrated under reduced pressure. The resulting residue was purified by flash column chromatography with gradient eluents ($MeOH/CH_2Cl_2$ 1:100 to 1:10) to give (±)-coerusecine[4,18,19] (**2i** 1.2 g) in 41% yield.

1.2.4. Oxidative rearrangement of yohimbine to β-yohimbine oxindoles

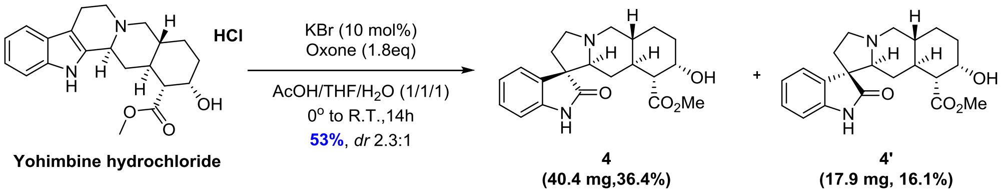

To a solution of yohimbine hydrochloride (117 mg, 0.3 mmol.) and KBr (0.03 mmol, 0.01M) in THF/H_2O/AcOH (v/v 1:1:1) (3.0 mL, 0.1M) at 0 ºC was added oxone (111 mg, 0.36 mmol, MW 307) in three portions. After completion of the addition, the resulting reaction mixture was stirred at rt for 12 h and then another batch of oxone (55 mg, 0.18 mmol) was added. After stirring for 1h, the reaction was quenched by addition of *aq. sat.* $NaHCO_3$ (10 mL) and *aq. sat.* Na_2CO_3 (10 mL) and diluted with EtOEt (20 mL). The organic fractions were collected, and the aqueous phase was extracted with EtOAc (4×20 mL). The combined organic fractions were washed with brine, dried over Na_2SO_4, filtered, and concentrated under reduced pressure. The resulting residue was purified by flash column chromatography with gradient eluents (MeOH/ CH_2Cl_2 1:100 to 1:20) to provide a diastereomeric mixture of β-yohimbine oxindoles (62 mg, *dr* 2.3:1) in 56% yield. The diastereomers were separated through flash column chromatography (EA/Hexane = 4:1) to give **4**[20] (40.4 mg, 36.4% yield) and **4'** (17.9mg, 16.1%).

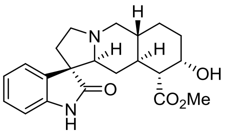

4

4 (40.4 mg, 36.4% yield). ^{1}H-NMR (400 MHz, $CDCl_3$) δ 8.75 (s, 1H), 7.38 (d, *J* = 7.2 Hz, 1H), 7.19 (td, *J* = 7.7, 1.2 Hz, 1H), 7.02 (t, *J* = 7.5 Hz, 1H), 6.90 (d, *J* = 7.7 Hz, 1H), 4.09 (s, 1H), 3.57 (s, 3H), 3.28 (s, 1H), 3.11 (d, *J* = 10.2 Hz, 2H), 2.52 (d, *J* = 11.0 Hz, 2H), 2.36 (ddd, *J* = 12.0, 9.3, 2.3 Hz, 1H), 2.12 (d, *J* = 11.6 Hz, 1H), 2.07 – 1.86 (m, 3H), 1.75 (t, *J* = 11.3 Hz, 1H), 1.54 – 1.25 (m, 4H), 1.09 – 1.00 (m, 1H), 0.69 (d, *J* = 11.8 Hz, 1H).^{13}C-NMR (100 MHz, $CDCl_3$) δ 181.8, 175.4, 140.2, 133.7, 127.6, 125.0, 122.4, 109.6, 71.4, 66.7, 58.7, 56.7, 53.4, 52.3, 51.7, 40.3, 36.1, 35.3, 31.3, 30.3, 23.3. IR (neat) 2924.5, 1697.9, 1617.9, 1469.9, 1210.7, 1156.6, 1018.3, 751.9 cm^{-1}; HRMS (CI^+) (m/z) *calcd.* for $C_{21}H_{26}N_2O_4$ $[M+H]^+$ 370.1893; found 370.1902.

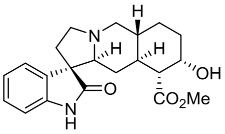

4'

4' (17.9 mg, 16.1% yield). ^{1}H-NMR (400 MHz, $CDCl_3$) δ 7.89 (s, 1H), 7.22 – 7.14 (m, 2H), 7.05 (t, *J* = 7.5 Hz, 1H), 6.85 (d, *J* = 7.6 Hz, 1H), 4.10 (s, 1H), 3.59 (s, 3H), 3.36 (q, *J* = 8.9, 7.2 Hz, 1H), 3.19 (dd, *J* = 10.8, 2.7 Hz, 1H), 3.08 (s, 1H), 2.48 – 2.41 (m, 2H), 2.33 – 2.23 (m, 2H), 1.99 (d, *J* = 2.8 Hz, 1H), 1.91 (d, *J* = 3.0 Hz, 1H), 1.82 (d, *J* = 10.2 Hz, 1H), 1.66 (dd, *J* = 11.5, 8.3 Hz, 1H), 1.45 (d, *J* = 12.8 Hz, 1H), 1.42 – 1.35 (m, 1H), 1.35 – 1.27 (m, 1H), 1.17 – 1.11 (m, 1H), 0.88 (t, *J* = 6.7 Hz, 1H). ^{13}C-NMR (100 MHz, $CDCl_3$) δ 181.1, 175.9, 140.5, 133.4, 128.0, 123.2, 122.8, 109.3, 74.3, 66.6, 58.7, 55.8, 54.5, 52.3, 51.6, 40.0, 36.6, 35.0, 31.2, 29.6, 23.4. IR (neat) 2929.8, 1705.4, 1619.4, 1471.7, 1440.0, 1213.5, 1158.8, 1113.7, 911.2, 728.4 cm^{-1}; HRMS (CI^+) (m/z) *calcd.* for $C_{21}H_{26}N_2O_4$ $[M]^+$ 370.1893; found 370.1908.

Preparation of Tetrahydropyrano[2,3-b]indoles (THPIs) 5a-5e

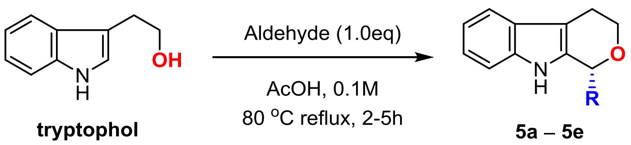

General Procedure-3: To a solution of tryptophol (1.0 equiv) and aldehyde (1.0 equiv) in AcOH (0.2 M) was heated to reflux at 80 °C for 2 – 5h. Then the resulting reaction mixture was cooled to rt. The reaction mixture was basified to pH 9 –10 by addition of *aq. sat.* Na_2CO_3 and then diluted with CH_2Cl_2. The organic fractions were collected, and the aqueous phase was extracted with CH_2Cl_2 three times. The combined organic fractions were washed with brine, dried over Na_2SO_4, filtered and concentrated under reduced pressure. The resulting residue was purified by flash column chromatography with gradient eluents (EtOAc/hexane 1:50 to 1:10) to give THPIs **5a**[21], **5b - 5e**. The physical data for new compounds were provided below.

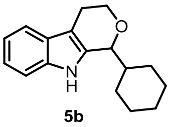

5b

5b: reaction of tryptophol (500 mg, 3.1 mmol) with Cyclohexanaldehyde (0.34 mL, 3.1 mmol) provided THPI **5b** (360 mg, 46% yield). ^{1}H-NMR (400 MHz, $CDCl_3$) δ 7.88 (s, 1H), 7.55 (d, *J* = 7.5 Hz, 1H), 7.36 (d, *J* = 7.8 Hz, 1H), 7.23 – 7.13 (m, 2H), 4.73 (s 1H), 4.32 (ddd, *J* = 11.1, 5.4, 2.2 Hz, 1H), 3.81 (td, *J* = 10.7, 3.7 Hz, 1H), 2.98 (dddd, *J* = 15.5, 10.3, 5.4, 2.2 Hz, 1H), 2.72 (ddt, *J* = 15.3, 4.0, 2.0 Hz, 1H), 1.86 – 1.68 (m, 6H), 1.56 – 1.49 (m, 1H), 1.41 – 1.19 (m, 5H).^{13}C-NMR (100 MHz, $CDCl_3$) δ 135.6 , 133.8, 126.9, 121.3, 119.2, 117.8, 110.6, 108.7, 77.5, 64.6, 42.3, 29.3, 26.5, 26.4, 26.2, 26.1, 22.2. IR (neat) 2921.6, 2850.0, 1714.1, 1450.7, 1315.3, 1098.5, 1046.3, 736.9 cm^{-1}; HRMS (CI$^+$) (m/z) *calcd.* for $C_{17}H_{21}NO$ [M]$^+$255.1623; found 255.1625.

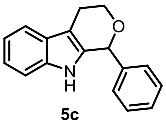

5c

5c: reaction of tryptophol (300 mg, 1.86 mmol) with benzaldehyde (0.19 mL, 1.86 mmol) provided THPI **5c** (250 mg, 54% yield). ^{1}H-NMR (400 MHz, $CDCl_3$) δ 7.74 – 7.54 (m, 2H), 7.43 (td, *J* = 5.9, 4.9, 3.5 Hz, 5H), 7.24 – 7.16 (m, 3H), 5.80 (s, 1H), 4.35 (ddd, *J* = 11.3, 5.3, 3.2 Hz, 1H), 4.02 (ddd, *J* = 11.3, 9.3, 4.1 Hz, 1H), 3.15 (dddd, *J* = 14.9, 9.3, 5.4, 2.0 Hz, 1H), 2.89 (dtd, *J* = 15.4, 3.9, 1.8 Hz, 1H). ^{13}C-NMR (100 MHz, $CDCl_3$) δ 139.3, 135.9, 133.4, 128.8, 128.7, 128.4, 126.8, 121.8, 119.5, 118.2, 110.9, 108.6, 75.9, 64.5, 22.2. IR (neat) 3395.4, 2908.9, 2846.6, 1705.2, 1451.3, 1298.9,1258.4, 1043.6, 972.8, 734.2, 696.2 cm^{-1}; HRMS (CI$^+$) (m/z) *calcd.* for $C_{17}H_{15}NO$ [M]$^+$ 249.1154; found 249.1152.

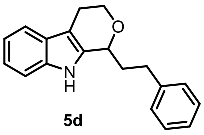

5d

5d: reaction of tryptophol (300 mg, 1.86 mmol) with 3-phenylpropanal (0.25 mL, 1.86 mmol) provided THPI **5d** (152 mg, 30% yield). ^{1}H-NMR (400 MHz, $CDCl_3$) δ 7.61 (brs, 1H), 7.51 (d, *J* = 7.5 Hz, 1H), 7.30 (q, *J* = 6.1, 4.9 Hz, 4H), 7.25 – 7.10 (m, 5H), 4.87 (ddd, *J* = 8.2, 4.0, 1.8 Hz, 1H), 4.32 (ddd, *J* = 11.9, 5.8, 2.8 Hz, 1H), 3.86 (td, *J* = 10.5, 4.0 Hz, 1H), 3.07 – 2.93 (m, 1H), 2.85 (t, *J* = 7.8 Hz, 2H), 2.74 (brd, *J* = 15.5 Hz, 1H), 2.25 – 2.07 (m, 2H).^{13}C-NMR (100 MHz, $CDCl_3$) δ 141.9, 135.8, 134.5, 128.5, 128.5, 128.3, 128.2, 127.1, 125.9, 121.7, 119.6, 118.1, 110.9, 108.3, 72.0, 64.5, 36.4, 31.2, 22.4. IR (neat) 3407.6, 3310.0, 2915.9, 2849.1, 1712.5, 1598.2, 1449.7, 1312.6, 1260.1, 1147.8, 1087.0, 1043.6, 1015.2,737.1, 698.0 cm^{-1}; HRMS (CI$^+$) (m/z) *calcd.* for $C_{19}H_{19}NO$ [M]$^+$277.1467; found 277.1462.

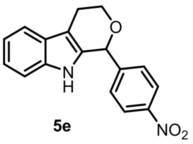

5e

5e: reaction of tryptophol (300 mg, 1.86 mmol) with 4-nitrobenzaldehyde (0.19 mL, 1.86 mmol) provided THPI **5e** (270 mg, 49% yield). ^{1}H-NMR (400 MHz, $CDCl_3$) δ 8.23 (d, *J* = 8.7 Hz, 2H), 7.58 (d, *J* = 8.6 Hz, 3H), 7.48 (brs, 1H), 7.24 – 7.12 (m, 2H), 5.91 (s, 1H), 4.32 (ddt, *J* = 11.3, 5.4, 2.9 Hz, 1H), 4.01 (ddd, *J* = 11.3, 9.5, 4.1 Hz, 1H), 3.11 (m, 1H), 2.92 – 2.82 (m, 1H).^{13}C-NMR (100 MHz, $CDCl_3$) δ 148.1, 146.7, 136.2, 131.9, 129.1, 126.8, 124.0, 122.5, 120.0, 118.5, 111.1, 109.3, 75.0, 64.9, 22.1. IR (neat) 3405.6, 1721.3,1521.2, 1347.1, 1241.0, 1083.5, 1040.4, 742.1 cm^{-1}; HRMS (CI^+) (m/z) *calcd.* for $C_{17}H_{14}N_2O_3$ $[M+H]^+$ 295.1083; found 295.1073.

Oxidative Rearrangement of THPIs to oxa-Spirooxindoles 6a-6e.

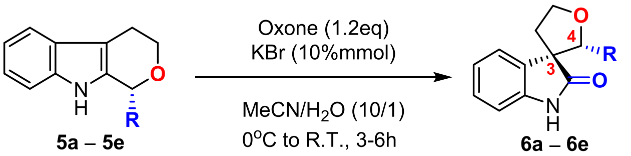

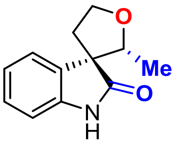

6a

6a[22]**:** Following the **General Procedure-1**, reaction of THPI **5a** (50 mg, 0.27 mmol) with KBr (2.7 mL, 0.01 M, 0.027 mmol) and oxone (98 mg, 0.32 mmol, MW 307) provided oxa-spirooxindole **6a** (39.0 mg, 72% yield, *d.r.* >20:1). ^{1}H-NMR (400 MHz, $CDCl_3$) δ 9.50 (s, 1H), 7.25 – 7.19 (m, 2H), 7.05 (t, *J* = 7.5 Hz, 1H), 6.97 (d, *J* = 8.0 Hz, 1H), 4.32 – 4.08 (m, 3H), 2.70 (ddd, *J* = 12.7, 9.4, 6.6 Hz, 1H), 2.31 – 2.14 (m, 1H), 0.91 (d, *J* = 6.2 Hz, 3H).^{13}C-NMR (100 MHz, $CDCl_3$) δ 180.3, 140.3, 131.6, 127.9, 124.5, 122.6, 110.0, 82.2, 67.1, 58.3, 38.0, 15.0. IR (neat) 2924.8, 1701.6, 1617.5, 1466.4, 1106.0, 1045.1, 862.4, 748.9 cm^{-1}; HRMS (CI^+) (m/z) *calcd.* for $C_{12}H_{13}NO_2$ $[M+H]^+$ 204.1025; found 204.1030. The relative configuration was confirmed by X-ray diffraction analysis (The Cambridge Crystallographic Data Centre, Deposition number 1935506) (Supplementary Figure 245, page 280).

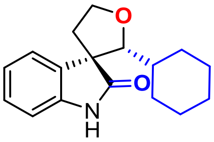

6b

6b: Following the **General Procedure-1**, reaction of THPI **5b** (57 mg, 0.22 mmol) with KBr (2.2 mL, 0.01 M, 0.022 mmol) and oxone (82 mg, 0.27 mmol, MW 307) provided oxa-spirooxindole **6b** (42.3 mg, 70% yield, *d.r.* >20:1). ^{1}H-NMR (400 MHz, $CDCl_3$) δ 9.20 (s, 1H), 7.27 (d, *J* = 6.9 Hz, 1H), 7.23 (td, *J* = 7.7, 1.3 Hz, 1H), 7.05 (td, *J* = 7.5, 1.0 Hz, 1H), 6.96 (d, *J* = 7.9 Hz, 1H), 4.29 – 4.05 (m, 2H), 3.82 (d, *J* = 9.8 Hz, 1H), 2.64 (ddd, *J* = 12.4, 9.3, 7.4 Hz, 1H), 2.09 (ddd, *J* = 12.6, 7.8, 5.1 Hz, 1H), 2.05 – 1.95 (m, 1H), 1.71 – 1.60 (m, 1H), 1.51 – 1.42 (m, 1H), 1.42 – 1.30 (m, 2H), 1.15 – 0.94 (m, 3H), 0.91 – 0.70 (m, 3H).^{13}C-NMR (100 MHz, $CDCl_3$) δ 181.0, 139.9, 131.9, 127.8, 124.5, 122.6, 110.1, 89.9, 66.2, 56.6, 40.4, 39.3, 30.9, 27.2, 26.0, 25.3. IR (neat) 2923.6, 2851.5, 1701.5, 1616.9, 1462.2, 1220.2, 1071.4, 745.5 cm^{-1}; HRMS (CI^+) (m/z) *calcd.* for $C_{17}H_{21}NO_2$ $[M]^+$271.1572; found 271.1576. The relative configuration was confirmed by X-ray diffraction analysis (The Cambridge Crystallographic Data Centre, Deposition number 1935507) (Supplementary Figure 246, page 281).

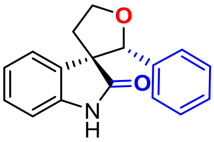

6c

6c: Following the **General Procedure-1**, reaction of THPI **5c** (25 mg, 0.1 mmol) with KBr (1.0 mL, 0.01 M, 0.01 mmol) and oxone (37 mg, 0.12 mmol, MW 307) provided oxa-spirooxindole **6c** (24.4 mg, 92% yield, *d.r.* >20:1). ^{1}H-NMR (400 MHz, $CDCl_3$) δ 8.89 (s, 1H), 7.16 – 7.10 (m, 3H), 7.10 – 7.00 (m, 4H), 6.88 (t, *J* = 7.5 Hz, 1H), 6.70 (d, *J* = 7.7 Hz, 1H), 5.25 (s, 1H), 4.60 – 4.35 (m, 2H), 2.86 (ddd, *J* = 12.6, 9.4, 6.9 Hz, 1H), 2.35 (ddd, *J* = 13.0, 8.1, 5.4 Hz, 1H).^{13}C-NMR (100 MHz, $CDCl_3$) δ 179.8, 139.7, 136.5, 131.2, 127.7, 127.6, 127.4, 125.4, 124.8, 122.2, 109.5, 87.5, 67.3, 59.7, 38.1 IR (neat) 2926.3, 2880.5, 1700.2, 1615.9, 1462.7, 1340.8, 1218.4, 1060.6, 746.7, 703.4 cm^{-1}; HRMS (CI^{+}) (m/z) *calcd.* for $C_{17}H_{15}NO_2$ $[M+H]^+$ 266.1181; found 266.1190. The relative configuration was confirmed by X-ray diffraction analysis (The Cambridge Crystallographic Data Centre, Deposition number 1935508) (Supplementary Figure 247, page 282).

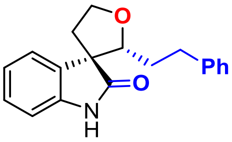

6d

6d[23]**:** Following the **General Procedure-1**, reaction of THPI **5d** (56 mg, 0.2 mmol) with KBr (2.0 mL, 0.01 M, 0.02 mmol) and oxone (74 mg, 0.24 mmol, MW 307) provided oxa-spirooxindole **6d** (55.1 mg, 93% yield, *d.r.* >20:1). ^{1}H-NMR (400 MHz, $CDCl_3$) δ 8.44 (s, 1H), 7.31 – 6.91 (m, 9H), 4.31 (q, *J* = 7.9 Hz, 1H), 4.21 (m, 1H), 4.14 (dd, *J* = 8.6, 4.6 Hz, 1H), 2.83 – 2.65 (m, 2H), 2.51 – 2.39 (m, 1H), 2.23 (ddd, *J* = 13.2, 8.0, 5.6 Hz, 1H), 1.80 – 1.60 (m, 2H), 1.41 (m, 1H).^{13}C-NMR (100 MHz, $CDCl_3$) δ 179.7, 141.5, 139.9, 131.7, 128.3, 128.2, 128.0, 125.8, 124.6, 122.8, 109.9, 85.9, 67.1, 57.6, 38.7, 32.7, 32.7. IR (neat) 2938.1, 2867.0, 1701.4, 1614.2, 1462.6, 1222.3, 1065.4, 746.3, 691.9, 568.3 cm^{-1}; HRMS (CI^{+}) (m/z) *calcd.* for $C_{19}H_{19}NO_2$ $[M+H]^+$294.1494; found 294.1491.

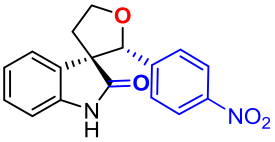

6e

6e: Following the **General Procedure-1**, reaction of THPI **5e** (33 mg, 0.11 mmol) with KBr (1.1 mL, 0.01 M, 0.011 mmol) and oxone (41 mg, 0.13 mmol, MW 307) provided oxa-spirooxindole **6e** (32.4 mg, 95% yield, *dr* >20:1). ^{1}H-NMR (400 MHz, $CDCl_3$) δ 8.26 (s, 1H), 7.99 – 7.92 (m, 2H), 7.31 (d, *J* = 8.4 Hz, 2H), 7.12 – 7.01 (m, 2H), 6.89 (td, *J* = 7.6, 1.0 Hz, 1H), 6.70 (d, *J* = 7.7 Hz, 1H), 5.29 (s, 1H), 4.51 (td, *J* = 8.3, 7.2 Hz, 1H), 4.42 (ddd, *J* = 9.4, 8.7, 5.2 Hz, 1H), 2.90 (ddd, *J* = 12.6, 9.5, 7.2 Hz, 1H), 2.37 (ddd, *J* = 12.9, 8.0, 5.2 Hz, 1H).^{13}C-NMR (100 MHz, $CDCl_3$) δ 178.6, 147.2, 144.2, 139.3, 130.3, 128.3, 126.2, 124.5, 123.0, 122.7, 109.7, 86.5, 67.7, 59.6, 38.2. IR (neat) 2887.8, 1705.0, 1612.1, 1519.7, 1472.9, 1344.4, 1220.9, 1110.4, 1076.3, 911.3, 852.3, 747.9, 630.4 cm^{-1}; HRMS (CI^{+}) (m/z) *calcd.* for $C_{17}H_{14}N_2O_4$ $[M+H]^+$ 311.1032; found 311.1045.

Preparation of indole derivatives 7 from indole

Substrates **7a**, **7t**, **7r** and **7w** were acquired commercially.**7b**, **7c** , **7e** , **7m**[24]; **7d**[25]; **7f**[26]; **7h**[27]; **7i**, **7g**[28]; **7k**, **7n**, **7o**, **7p**[29]; **7j**[30]; **7l**[31]; **7u**, **7v**[32]; **7q**[33]; **7x** ,**7y**[34] are known compounds and they are synthesized according to related literature.

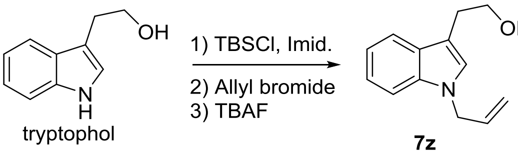

To a solution of tryptophol (10.0 mmol) and imidazole (1.36 g, 20.0 mmol) in DMF (50 mL) at 0 °C was

added *tert*-butyldimethylsilyl chloride (TBSCl, 1.66 g, 11 mmol). Then the resulting reaction mixture was warmed to rt and stirred for 3 h. The mixture was quenched by addition of water (30 mL) and then diluted with EtOAc (30 mL). The organic fractions were collected, and the aqueous phase was extracted with EtOAc (30 mL × 3). The combined organic layers were washed with water, brine, dried over Na_2SO_4, filtered and concentrated under reduced pressure. The resulting residue was used directly for the next step without further purification. To a solution of the resulting TBS ether in THF (50 mL) at 0 °C was added NaH (400 mg, 10.0 mmol) in three portions. The resulting reaction mixture was stirred at 0 °C for 15 min and then was warmed to rt. After stirring at rt for 1 h, the reaction mixture was cooled to 0 °C and then allyl bromide (11.0 mmol) was added. The reaction mixture was stirred at rt for 6-12 h. After the reaction was completed as determined by TLC analysis, the reaction was quenched by slow addition of *aq. sat.* $NaHCO_3$ (30 mL). The organic fractions were collected, and the aqueous phase was extracted with EtOAc (30 mL × 3). The combined organic layers were washed with brine, dried over Na_2SO_4, filtered and concentrated under reduced pressure to provide the crude products. To a solution of the crude product obtained above in THF (50 mL) was added tetra-butylammonium fluoride (TBAF, 15 mmol) dropwise at rt and then the resulting reaction mixture was stirred at rt for 24 h. The reaction mixture was concentrated under reduced pressure and the resulting residue was purified by ash column chromatography with gradient eluents (EtOAc/hexane 1:10 to 1:2) to provide the desired product **7z** (1.48 g, 38% yield). ^{1}H-NMR (400 MHz, $CDCl_3$) δ: 7.68 (d, J = 7.9 Hz, 1H), 7.37 (d, J = 8.2 Hz, 1H), 7.31 – 7.25 (m, 1H), 7.21 – 7.16 (m, 1H), 7.03 (s, 1H), 6.04 (m, 1H), 5.25 (ddd, J = 10.2, 2.8, 1.4 Hz, 1H), 5.16 (ddd, J = 17.1, 3.0, 1.6 Hz, 1H), 4.73 (dt, J = 5.5, 1.6 Hz, 2H), 3.94 (t, J = 6.5 Hz, 2H), 3.08 (td, J = 6.4, 0.4 Hz, 2H).^{13}C-NMR (100 MHz, $CDCl_3$) δ: 136.7, 133.6, 128.2, 126.3, 121.8, 119.1, 119.1, 117.4, 111.2, 109.8, 62.8, 48.8. 28.8. IR (KBr, neat) 3363.0, 2876.2, 1467.3, 1375.2, 1333.7, 1048.2, 743.7 cm^{-1}; HRMS (ESI) (*m/z*) *calcd.* for $C_{13}H_{16}NO$ $[M+H]^+$ 202.1226; found 202.1228.

Oxidation of indoles to 2-oxindoles

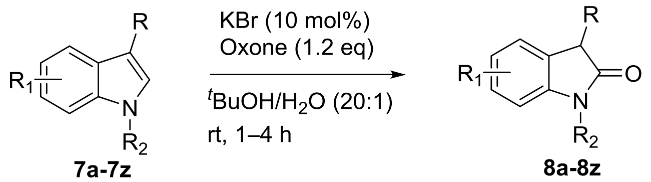

General Procedure-4: To a solution of indole derivatives **7a-7z** (0.50 mmol) and KBr (6.0 mg, 0.05 mmol) in ^{t}BuOH/H_2O (v/v 20:1) (5 mL, 0.1 M) at rt was added oxone (368.4 mg, 0.60 mmol, MW 307). and was stirred for 1–4 h. The reaction mixture was quenched by addition of *aq. sat.* Na_2SO_3 (5.0 mL) and then diluted with EtOAc (30 mL). The organic fractions were collected, and the aqueous phase was extracted with EtOAc (10 mL × 3). The combined organic layers were washed with brine, dried over Na_2SO_4, filtered and concentrated under reduced pressure. The resulting residue was purified by silica gel column chromatography to give 2-oxindoles **8a–8z**.

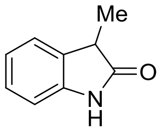

8a

8a (66.9 mg, 91% yield): ^{1}H-NMR (400 MHz, $CDCl_3$) δ: 9.67 (s, 1H), 7.20 (t, *J* = 7.5 Hz, 2H), 7.07 – 6.98 (m, 1H), 6.94 (d, *J* = 7.6 Hz, 1H), 3.48 (q, *J* = 7.6 Hz, 1H), 1.51 (d, *J* = 7.7 Hz, 3H). ^{13}C-NMR (100 MHz, $CDCl_3$) δ: 182.2, 141.6, 131.4, 127.9, 123.8, 122.4, 110.0, 41.3, 15.3. IR (KBr, neat) 3182.8, 2931.7, 1714.3, 1472.5, 1232.2, 1218.1, 744.6 cm^{-1}; HRMS (ESI) (*m/z*) *calcd.* for $C_9H_{10}NO$ $[M + H]^+$ 148.0757; found 148.0759.

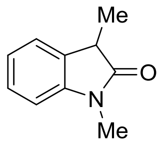

8b

8b (74.1 mg, 92% yield): ^{1}H-NMR (400 MHz, $CDCl_3$) δ: 7.25-7.18 (m, 2H), 7.01 (t, *J* = 7.5 Hz, 1H), 6.78 (d, *J* = 7.7 Hz, 1H), 3.38 (q, *J* = 7.6 Hz, 1H), 3.16 (s, 3H), 1.44 (d, *J* = 7.6 Hz, 3H). ^{13}C-NMR (100 MHz, $CDCl_3$) δ: 178.7, 144.1, 130.7, 127.9, 123.5, 122.4, 108.0, 40.6, 26.2, 15.4. IR (KBr) 1724.2, 1611.5, 1468.1, 1377.1, 1124.2, 751.8 cm^{-1}; HRMS (ESI) (*m/z*) *calcd.* for $C_{10}H_{12}NO$ $[M+H]^+$162.0913; found 162.0918.

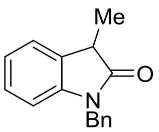

8c

8c (106.6 mg, 90% yield): ^{1}H-NMR (400 MHz, $CDCl_3$) δ: 7.34 – 7.20 (m, 6H), 7.15 (t, *J* = 7.7 Hz, 1H), 7.02 (dd, *J* = 10.9, 4.0 Hz, 1H), 6.71 (d, *J* = 7.7 Hz, 1H), 4.91 (s, 2H), 3.53 (q, *J* = 7.6 Hz, 1H), 1.54 (d, *J* = 7.6 Hz, 3H). ^{13}C-NMR (100 MHz, $CDCl_3$) δ: 178.9, 143.2, 136.1, 130.8, 128.9, 127.9, 127.7, 127.4, 123.7, 122.5, 109.1, 43.8, 40.7, 15.7. IR (KBr, neat) 3056.7, 2970.3, 1713.3, 1487.5, 1359.3, 979.6, 749.1 cm^{-1}; HRMS (ESI) (*m/z*) *calcd.* for $C_{16}H_{15}NONa$ $[M + Na]^+$ 260.1046; found 260.1050.

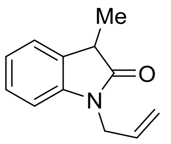

8d

8d (85.1 mg, 91% yield): ^{1}H-NMR (400 MHz, $CDCl_3$) δ: 7.23 (t, *J* = 7.4 Hz, 2H), 7.04 (t, *J* = 7.5 Hz, 1H), 6.82 (d, *J* = 7.8 Hz, 1H), 5.83 (m, 1H), 5.30 – 5.05 (m, 2H), 4.43 – 4.20 (m, 2H), 3.46 (q, *J* = 7.6 Hz, 1H), 1.49 (d, *J* = 7.7 Hz, 3H). ^{13}C-NMR (100 MHz, $CDCl_3$) δ: ^{13}C NMR (100 MHz, $cdcl_3$) δ 178.4, 143.1, 131.6, 130.6, 127.8, 123.6, 122.4, 117.4, 108.9, 42.3, 40.5, 15.6. IR (KBr, neat) 1713.4, 1612.9, 1488.5, 1354.1, 1205.8, 752.0 cm^{-1}; HRMS (ESI) (*m/z*) *calcd.* for $C_{12}H_{13}NONa$ $[M+Na]^+$ 210.0889; found 210.0886.

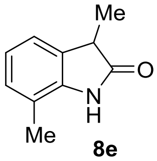

8e

8e (70.8 mg, 88% yield): ^{1}H-NMR (400 MHz, $CDCl_3$) δ: 9.87 (s, 1H), 7.05 (dd, *J* = 12.2, 7.5 Hz, 2H), 6.96 (t, *J* = 7.5 Hz, 1H), 3.49 (q, *J* = 7.7 Hz, 1H), 2.34 (s, 3H), 1.51 (d, *J* = 7.7 Hz, 3H). ^{13}C-NMR (100 MHz, $CDCl_3$) δ: 182.5, 140.4, 131.0, 129.3, 122.4, 121.1, 119.6, 41.7, 16.6, 15.5. IR (KBr, neat) 3154.4, 3056.4, 1700.0, 1626.2, 1334.7, 1218.1, 735.6 cm^{-1}; HRMS (ESI) (*m/z*) *calcd.* for $C_{10}H_{11}NONa$ $[M+Na]^+$184.0733; found 184.0737.

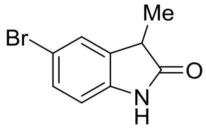

8f

8f (101.9 mg, 91% yield): ^{1}H-NMR (400 MHz, $CDCl_3$) ^{1}H NMR (400 MHz, $cdcl_3$) δ 9.30 (s, 1H), 7.37 – 7.30 (m, 2H), 6.81 (d, *J* = 8.8 Hz, 1H), 3.47 (q, *J* = 7.6 Hz, 1H), 1.49 (d, *J* = 7.7 Hz, 3H). ^{13}C-NMR (100 MHz, $CDCl_3$) δ: 181.3, 140.4, 133.4, 130.9, 127.2, 115.2, 111.4, 41.4, 15.2. IR (KBr, neat) 3200.4, 1718.0, 1475.5, 1212.7, 1170.8, 814.8, 714.0 cm^{-1}; HRMS (ESI) (*m/z*) *calcd.* for $C_9H_8BrNONa$$[M+Na]^+$ 247.9681; found 247.9682.

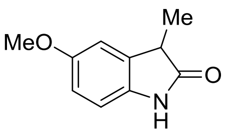

8g

8g (78.8 mg, 89% yield): ^{1}H-NMR (400 MHz, $CDCl_3$) δ: 8.92 (s, 1H), 6.87 – 6.76 (m, 2H), 6.73 (dd, *J* = 8.4, 2.4 Hz, 1H), 3.78 (s, 3H), 3.44 (q, *J* = 7.7 Hz, 1H), 1.49 (d, *J* = 7.7 Hz, 3H).^{13}C-NMR (100 MHz, $CDCl_3$) δ:181.6, 155.9, 134.8, 132.8, 112.4, 111.2, 110.2, 55.9, 41.7, 15.4. IR (KBr, neat) 3161.6, 3050.7, 2936.3, 1699.0, 1485.7, 1209.4, 1033.9 cm^{-1}; HRMS (ESI) (*m/z*) *calcd.* for $C_{10}H_{12}NO_2$ $[M+H]^+$178.0863; found 178.0868.

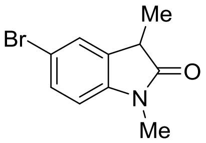

8h

8h (106.4 mg, 89% yield): ^{1}H-NMR (400 MHz, $CDCl_3$) δ: 7.41 – 7.37 (m, 1H), 7.35 – 7.32 (m, 1H), 6.69 (d, *J* = 8.2 Hz, 1H), 3.43 (q, *J* = 7.7 Hz, 1H), 3.18 (s, 3H), 1.46 (d, *J* = 7.7 Hz, 3H).^{13}C-NMR (100 MHz, $CDCl_3$) δ: 178.1, 143.2, 132.8, 130.8, 127.0, 115.2, 109.5, 40.7, 26.4, 15.4. IR (KBr, neat) 2973.4, 1717.4, 1608.8, 1488.4, 1342.8, 1053.8, 808.8 cm^{-1}; HRMS (ESI) (*m/z*) *calcd.* for $C_{10}H_{11}BrNO$ $[M+H]^+$ 240.0019; found 240.0011.

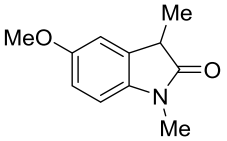

8i

8i (84.0 mg, 88% yield): ^{1}H-NMR (400 MHz, $CDCl_3$) δ: 6.81 (d, *J* = 1.0 Hz, 1H), 6.74 (d, *J* = 8.4 Hz, 1H), 6.66 (d, *J* = 8.4 Hz, 1H), 3.74 (s, 3H), 3.34 (q, *J* = 7.6 Hz, 1H), 3.12 (s, 3H), 1.41 (d, *J* = 7.7 Hz, 3H). ^{13}C-NMR (100 MHz, $CDCl_3$) δ: 178.2, 155.9, 137.5, 131.9, 111.8, 111.1, 108.1, 55.7, 40.9, 26.1, 15.3. IR (KBr, neat) 2933.5, 1704.3, 1501.0, 1367.8, 1148.7, 1024.9, 804.7 cm^{-1}; HRMS (ESI) (*m/z*) *calcd.* for $C_{11}H_{13}NO_2Na$ $[M+Na]^+$ 214.0838; found 214.0844.

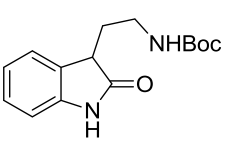

8j

8j (124.2 mg, 90% yield): ^{1}H NMR (400 MHz, $CDCl_3$) δ 8.96 (s, 1H), 7.29 (d, *J* = 6.9 Hz, 1H), 7.20 (t, *J* = 7.7 Hz, 1H), 7.02 (t, *J* = 7.2 Hz, 1H), 6.89 (d, *J* = 7.7 Hz, 1H), 5.00 (t, *J* = 5.5 Hz, 1H), 3.50 (t, *J* = 6.5 Hz, 1H), 3.45 – 3.24 (m, 2H), 2.17 – 2.05 (m, 2H), 1.42 (s, 9H).^{13}C-NMR (100 MHz, $CDCl_3$) δ: ^{13}C NMR (100 MHz, $cdcl_3$) δ 180.90, 156.09, 141.65, 129.32, 128.0, 124.1, 122.4, 110.0, 79.2, 44.0, 37.8, 30.7, 28.4. IR (KBr, neat) 2976.3, 1698.1, 1694.2, 1471.2, 1365.3, 1163.9, 748.7 cm^{-1}; HRMS (ESI) (*m/z*) *calcd.* for $C_{15}H_{20}N_2O_3Na$ $[M+Na]^+$ 299.1366; found 299.1371.

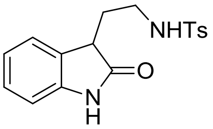

8k

8k (155.1 mg, 94% yield): ^{1}H-NMR (400 MHz, $CDCl_3$) δ: 8.37 (s, 1H), 7.73 (d, *J* = 8.2 Hz, 2H), 7.27 (d, *J* = 8.2 Hz, 2H), 7.21 (t, *J* = 7.7 Hz, 1H), 7.14 (d, *J* = 7.4 Hz, 1H), 7.02 (t, *J* = 7.5 Hz, 1H), 6.87 (d, *J* = 7.8 Hz, 1H), 5.84 – 5.72 (m, 1H), 3.53 – 3.44 (m, 1H), 3.22 – 3.08 (m, 2H), 2.40 (s, 3H), 2.18 (m, 1H), 2.00 (m, 1H).^{13}C-NMR (100 MHz, $CDCl_3$) δ: 180.7, 143.4, 141.4, 137.0, 129.8, 128.8, 128.3, 127.1, 124.1, 122.7, 110.3, 43.9, 40.6, 31.3, 21.6. IR (KBr, neat) 3279.1, 1695.7, 1470.5, 1320.0, 1158.0, 749.5, 552.2 cm^{-1}; HRMS (ESI) (*m/z*) *calcd.* for $C_{17}H_{18}N_2O_3SNa$$[M+Na]^+$ 353.0930; found 353.0939.

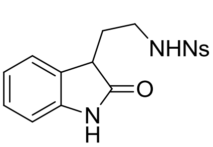

8l

8l (157.0 mg, 87% yield): ^{1}H-NMR (400 MHz, CD_3OD) δ: 8.29 (d, *J* = 9.0 Hz, 2H), 7.94 (d, *J* = 8.9 Hz, 2H), 7.10 (dd, *J* = 16.5, 7.7 Hz, 2H), 6.89 (t, *J* = 7.5 Hz, 1H), 6.75 (d, *J* = 7.7 Hz, 1H), 3.39 (t, *J* = 6.4 Hz, 1H), 2.95 (t, *J* = 7.3 Hz, 2H), 1.92 (m, 2H).^{13}C-NMR (100 MHz, CD_3OD) δ: 181.8, 151.4, 147.6, 143.5, 130.4, 129.4, 129.2, 125.4, 125.1, 123.4,

110.9, 44.6, 41.1, 31.8. IR (KBr, neat) 3308.9, 1699.3, 1528.5, 1468.7, 1350.2, 1163.5, 756.3,562.4 cm^{-1}; HRMS (ESI) (*m/z*) *calcd.* for $C_{16}H_{15}N_3O_5SNa$ [M+Na]$^+$ 384.0625; found 384.0633.

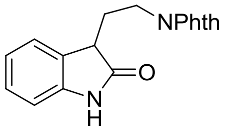

8m

8m (137.7 mg, 90% yield): ^{1}H-NMR (400 MHz, $CDCl_3$) δ: 8.59 (s, 1H), 7.76 (dd, *J* = 5.3, 3.0 Hz, 2H), 7.65 (dd, *J* = 5.2, 3.1 Hz, 2H), 7.29 (d, *J* = 7.3 Hz, 1H), 7.08 (t, *J* = 7.7 Hz, 1H), 6.88 (t, *J* = 7.5 Hz, 1H), 6.82 (d, *J* = 7.8 Hz, 1H), 4.04 – 3.91 (m, 1H), 3.90 – 3.70 (m, 1H), 3.53 (t, *J* = 5.9 Hz, 1H), 2.49 (dt, *J* = 13.4, 6.9 Hz, 1H), 2.40 – 2.23 (m, 1H).^{13}C-NMR (100 MHz, $CDCl_3$) δ: 179.5, 168.3, 141.6, 133.9, 132.1, 128.7, 128.1, 124.1, 123.2, 122.5, 110.1, 44.1, 35.2, 28.5. IR (KBr, neat) 3181.0, 3058.7, 1700.0, 1463.0, 1402.1, 1230.7, 1106.5, cm^{-1}; HRMS (ESI) (*m/z*) *calcd.* for $C_{18}H_{14}N_2O_3Na$ [M+Na]$^+$ 329.0897; found 329.0896.

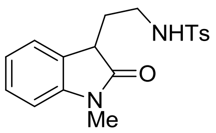

8n

8n (158.2 mg, 92% yield): ^{1}H-NMR (400 MHz, $CDCl_3$) δ: 9.13 (s, 1H), 7.63 (d, *J* = 8.1 Hz, 2H), 7.35 (d, *J* = 7.4 Hz, 1H), 7.29 (d, *J* = 8.1 Hz, 2H), 7.21 (t, *J* = 7.7 Hz, 1H), 7.04 (t, *J* = 7.5 Hz, 1H), 6.91 (d, *J* = 7.7 Hz, 1H), 3.55 (t, *J* = 6.4 Hz, 1H), 3.39 – 3.22 (m, 1H), 3.08 (m, 1H), 2.20 (m, 1H), 2.11 (m, 1H).]^{13}C-NMR (100 MHz, $CDCl_3$) δ: 180.3, 143.5, 141.7, 134.0, 129.8, 128.9, 128.1, 127.5, 124.3, 122.5, 110.1, 47.2, 43.2, 35.0, 28.8, 21.5. IR (KBr, neat) 3213.6, 1705.6, 1471.4, 1337.1, 1185.5, 753.4, 718.9, 549.4 cm^{-1}; HRMS (ESI) (*m/z*) *calcd.* for $C_{18}H_{20}N_2O_3SNa$ [M+Na]$^+$ 367.1087; found 367.1097.

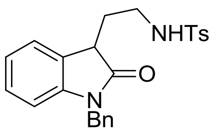

8o

8o (191.1 mg, 91% yield): ^{1}H-NMR (400 MHz, $CDCl_3$) δ: 8.65 (s, 1H), 7.69 (d, *J* = 8.2 Hz, 2H), 7.32 – 7.24 (m, 7H), 7.20 – 7.15 (m, 1H), 6.97 (d, *J* = 4.4 Hz, 2H), 6.83 (d, *J* = 7.7 Hz, 1H), 4.33 (dd, *J* = 44.6, 14.6 Hz, 2H), 3.30 (t, *J* = 6.3 Hz, 1H), 3.24 (t, *J* = 7.9 Hz, 2H), 2.42 (s, 3H), 2.10 – 1.90 (m, 2H).^{13}C-NMR (100 MHz, $CDCl_3$) δ: 179.7, 143.5, 141.4, 136.7, 136.3, 129.9, 128.7, 128.7, 128.7, 128.1, 128.0, 127.4, 124.1, 122.5, 109.8, 52.5, 45.1, 43.5, 29.3, 21.6. IR (KBr, neat) 3056.1, 2921.4, 1708.3, 1471.0, 1158.9, 941.8, 729.4, 659.4 cm^{-1}; HRMS (ESI) (*m/z*) *calcd.* for $C_{24}H_{24}N_2O_3SNa$ [M+Na]$^+$ 443.1400; found 443.1409.

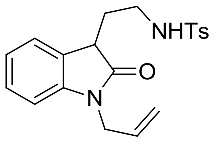

8p

8p (162.8 mg, 88% yield): ^{1}H-NMR (400 MHz, $CDCl_3$) δ: ^{1} 9.10 (s, 1H), 7.61 (d, *J* = 8.3 Hz, 2H), 7.26 – 7.19 (m, 3H), 7.16 (t, *J* = 7.7 Hz, 1H), 6.99 (td, *J* = 7.6, 0.7 Hz, 1H), 6.86 (d, *J* = 7.7 Hz, 1H), 5.56 (m, 1H), 5.14 (dd, *J* = 17.1, 1.3 Hz, 1H), 5.08 (dd, *J* = 10.0, 1.1 Hz, 1H), 3.78 (d, *J* = 6.5 Hz, 2H), 3.44 (t, *J* = 6.3 Hz, 1H), 3.34 – 3.14 (m, 2H), 2.36 (s, 3H), 2.13 (dd, *J* = 14.4, 7.4 Hz, 2H).^{13}C-NMR (100 MHz, $CDCl_3$) δ: 180.2, 143.4, 141.6, 136.6, 132.8, 129.8, 128.9, 128.1, 127.2, 124.2, 122.5, 119.4, 110.0, 51.0, 44.2, 43.5, 29.4, 21.6. IR (KBr,neat) 3246.9, 1706.3, 1471.5, 1335.9, 1184.7, 1092.2, 752.4 cm^{-1}; HRMS (ESI) (*m/z*) *calcd.* for $C_{20}H_{22}N_2O_3SNa$ [M+Na]$^+$ 393.1243; found 393.1253.

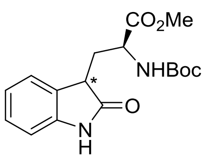

8q/8q'

8q/8q' (153.7 mg, 92%, *dr* 1.6:1.0): $[\alpha]^{25}_{D}$ = - 42 (*c* 0.71, $CHCl_3$). ^{1}H-NMR (400 MHz, $CDCl_3$) δ: 8.87 (s, 0.8H), 8.84 (s, 1.3H), 7.45 (d, *J* = 7.2 Hz, 1.3H), 7.25 – 7.18 (m, 3.8H), 7.05 (dd, *J* = 13.4, 6.5 Hz, 3.0H), 6.90 (dd, *J* = 13.0, 7.7 Hz, 2.9H), 5.81 (d, *J* = 8.1 Hz, 1.0H), 5.56 (d, *J* = 8.6 Hz, 1.6H), 4.69 (dd, *J* = 14.2, 8.5 Hz, 1.4H), 4.54 (d, *J* = 5.0 Hz, 0.9H), 3.73 (s, 3.1H), 3.69 (s, 4.0H), 3.55 (d, *J* = 5.9 Hz, 2.4H), 2.47 – 2.35 (m, 2.3H), 2.31 – 2.27 (m, 2.7H), 1.44 (s, 11.5H), 1.40 (s, 8.5H). ^{13}C-NMR (100 MHz, $CDCl_3$) δ: 180.4, 180.2, 172.9, 172.8, 155.8, 155.4, 141.5, 129.2, 128.5, 128.3, 124.9, 124.2, 122.8, 110.1, 80.3, 80.0, 52.6, 51.7, 43.30, 42.8, 33.6, 32.2, 28.4. IR (KBr, neat) 3295.8, 2954.4, 1705.4, 1472.0, 1366.5, 1248.2, 1163.3 cm^{-1}; HRMS (ESI) (*m/z*) *calcd.* for $C_{17}H_{22}N_2O_5Na$ $[M+Na]^+$ 357.1421; found 357.1428.

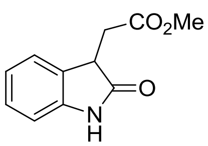

8r

8r (92.2 mg, 90% yield): ^{1}H-NMR (400 MHz, $CDCl_3$) δ: 9.29 (s, 1H), 7.20 (t, *J* = 7.2 Hz, 2H), 6.99 (t, *J* = 7.5 Hz, 1H), 6.91 (d, *J* = 7.7 Hz, 1H), 3.82 (dd, *J* = 8.0, 4.5 Hz, 1H), 3.69 (s, 3H), 3.08 (dd, *J* = 16.9, 4.5 Hz, 1H), 2.83 (dd, *J* = 16.9, 8.1 Hz, 1H). ^{13}C-NMR (100 MHz, $CDCl_3$) δ: 179.7, 171.7, 141.8, 128.8, 128.4, 124.1, 122.5, 110.1, 52.2, 42.5, 34.6. IR (KBr, neat) 3142.5, 2947.5, 1725.0, 1699.4, 1621.6, 1470.5, 1226.2, 763.7 cm^{-1}; HRMS (ESI) (*m/z*) *calcd.* for $C_{11}H_{11}NO_3Na$ $[M]^+$ 228.0631; found 228.0634.

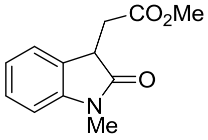

8s

8s (100.7 mg, 92% yield): ^{1}H-NMR (400 MHz, $CDCl_3$) δ: 7.29 – 7.16 (m, 2H), 6.99 (t, *J* = 7.5 Hz, 1H), 6.80 (d, *J* = 7.8 Hz, 1H), 3.75 (dd, *J* = 8.3, 4.4 Hz, 1H), 3.65 (s, 3H), 3.18 (s, 3H), 3.04 (dd, *J* = 16.9, 4.4 Hz, 1H), 2.73 (dd, *J* = 16.9, 8.4 Hz, 1H). ^{13}C-NMR (100 MHz, $CDCl_3$) δ: 176.8, 171.7, 144.4, 128.4, 128.2, 123.9, 122.6, 108.2, 77.5, 77.2, 76.8, 52.1, 41.9, 34.8, 26.4. IR (KBr, neat) 2952.5, 1713.5, 1614.9, 1351.9, 1208.4, 1090.8, 1022.4, 755.6 cm^{-1}; HRMS (ESI) (*m/z*) *calcd.* for $C_{12}H_{13}NO_3Na$ $[M+Na]^+$ 242.0788; found 242.0789.

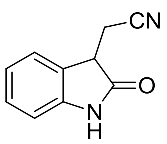

8t

8t (78.3 mg, 91% yield): ^{1}H-NMR (400 MHz, $CDCl_3$) δ: 8.56 (s, 1H), 7.49 (d, *J* = 7.5 Hz, 1H), 7.33 – 7.28 (m, 1H), 7.11 (td, *J* = 7.6, 0.9 Hz, 1H), 6.95 (d, *J* = 7.9 Hz, 1H), 3.71 (dd, *J* = 8.9, 4.8 Hz, 1H), 3.10 (dd, *J* = 16.8, 4.8 Hz, 1H), 2.76 (dd, *J* = 16.8, 8.9 Hz, 1H).δ: 176.6, 141.6, 129.6, 126.3, 124.6, 123.3, 117.4, 110.5, 42.0, 19.0. IR (KBr, neat) 3135.6, 3088.1, 2851.6, 1705.8, 1472.3, 1342.9, 1236.8, 749.5, cm^{-1}; HRMS (ESI) (*m/z*) *calcd.* for $C_{10}H_8N_2ONa$ $[M+Na]^+$195.0529; found 195.0529.

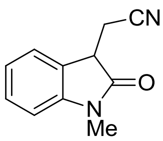

8u

8u (86.5 mg, 93% yield): ^{1}H-NMR (400 MHz, $CDCl_3$) δ: 7.49 (d, *J* = 7.4 Hz, 1H), 7.35 (t, *J* = 7.8 Hz, 1H), 7.11 (t, *J* = 7.6 Hz, 1H), 6.87 (d, *J* = 7.8 Hz, 1H), 3.65 (dd, *J* = 9.0, 4.7 Hz, 1H), 3.21 (s, 3H), 3.09 (dd, *J* = 16.9, 4.7 Hz, 1H), 2.68 (dd, *J* = 16.9, 9.0 Hz, 1H). ^{13}C-NMR (100 MHz, $CDCl_3$) δ: 174.2, 144.1, 129.4, 125.7, 124.2, 123.2, 117.3, 108.7, 41.4, 26.5, 19.0. IR (KBr, neat) 2967.7, 2934.5, 2256.8, 1709.0, 1611.5, 1470.9, 1376.2, 1022.7, 754.9 cm^{-1}; HRMS (ESI) (*m/z*) *calcd.* for $C_{11}H_{10}N_2ONa$ $[M+Na]^+$ 209.0685; found 209.0687

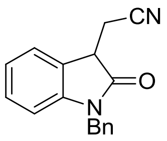

8v

8v (123.8 mg, 91% yield): ^{1}H-NMR (400 MHz, $CDCl_3$) δ: 7.41 (d, *J* = 7.4 Hz, 1H), 7.31 – 7.08 (m, 6H), 7.00 (t, *J* = 7.6 Hz, 1H), 6.68 (d, *J* = 7.9 Hz, 1H), 4.90 – 4.72 (m, 2H), 3.67 (dd, *J* = 8.7, 4.6 Hz, 1H), 3.06 (dd, *J* = 16.8, 4.6 Hz, 1H), 2.69 (dd, *J* = 16.8, 8.8 Hz, 1H). ^{13}C-NMR (100 MHz, $CDCl_3$) δ: 174.5, 143.3, 135.2, 129.4, 129.0, 127.9, 127.4, 125.7, 124.36, 123.3, 117.2, 109.8, 44.1, 41.5, 19.2. IR (KBr, neat) 2959.4, 1709.3, 1613.2, 1469.6, 1372.3, 1183.6, 1105.0, 737.8 cm^{-1}; HRMS (ESI) (*m/z) calcd.* for $C_{17}H_{14}N_2ONa$ $[M+Na]^+$ 285.0998; found 295.1006.

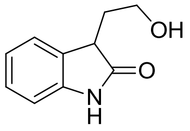

8w

8w (80.5 mg, 91% yield): ^{1}H-NMR (400 MHz, $CDCl_3$) δ: 9.06 (s, 1H), 7.16 (t, *J* = 7.9 Hz, 2H), 6.99 (t, *J* = 7.4 Hz, 1H), 6.86 (d, *J* = 7.6 Hz, 1H), 3.84 (t, *J* = 5.8 Hz, 2H), 3.57 (dd, *J* = 8.2, 5.2 Hz, 1H), 2.20 (dq, *J* = 14.4, 5.5 Hz, 1H), 2.13 – 1.96 (m, 1H). ^{13}C-NMR (100 MHz, $CDCl_3$) δ: 181.7, 141.5, 129.5, 128.2, 124.1, 122.7, 110.2, 60.7, 44.8, 33.2. IR (KBr, neat) 3343.7, 3155.6, 1687.9, 1619.2, 1472.0, 1349.7, 1212.7, 1038.7, 759.8 cm^{-1}; HRMS (ESI) (*m/z*) *calcd.* for $C_{10}H_{11}NO_2Na$ $[M+Na]^+$ 200.0682; found 200.0682.

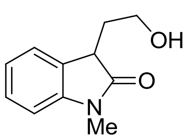

8x

8x (86.0 mg, 90% yield): ^{1}H-NMR (400 MHz, $CDCl_3$) δ: 7.27 – 7.13 (m, 2H), 7.02 (t, *J* = 7.5 Hz, 1H), 6.79 (d, *J* = 7.8 Hz, 1H), 3.84 (t, *J* = 5.8 Hz, 2H), 3.54 (dd, *J* = 8.5, 5.4 Hz, 1H), 2.24 – 2.06 (m, 1H), 1.98 (m, 1H). ^{13}C-NMR (100 MHz, $CDCl_3$) δ: 179.0, 144.0, 128.9, 128.1, 123.8, 122.8, 108.3, 60.8, 44.4, 33.4, 26.4. IR (KBr, neat) 3393.4, 1705.4, 1613.4, 1471.1, 1378.1, 1352.6, 1050.9, 755.0 cm^{-1}; HRMS (ESI) (*m/z*) *calcd.* for $C_{11}H_{13}NO_2Na$ $[M+Na]^+$ 214.0838; found 214.0844.

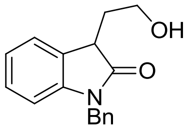

8y

8y (117.5 mg, 88% yield): ^{1}H-NMR (400 MHz, $CDCl_3$) δ: 7.32 – 7.21 (m, 6H), 7.18 (t, *J* = 7.8 Hz, 1H), 7.04 (t, *J* = 7.5 Hz, 1H), 6.74 (d, *J* = 7.8 Hz, 1H), 4.92 (q, *J* = 15.6 Hz, 2H), 3.94 (t, *J* = 5.8 Hz, 2H), 3.70 (dd, *J* = 8.7, 5.1 Hz, 1H), 2.29 (dq, *J* = 14.5, 5.3 Hz, 1H), 2.10 (m, 1H). ^{13}C-NMR (100 MHz, $CDCl_3$) δ: 179.1, 143.2, 135.8, 128.9, 128.1, 127.8, 127.9, 123.9, 122.8, 109.4, 61.0, 44.5, 43.9, 33.5. IR (KBr, neat) 3390.5, 1705.4, 1466.7, 1363.3, 1171.6, 1049.6, 752.7 cm^{-1}; HRMS (ESI) (*m/z*) *calcd.* for $C_{17}H_{17}NO_2Na$ $[M+Na]^+$ 290.1151; found 290.1157.

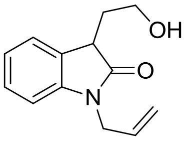

8z

8z (94.4 mg, 87% yield): ^{1}H-NMR (400 MHz, $CDCl_3$) δ: 7.18 (dd, *J* = 7.6, 4.7 Hz, 2H), 7.00 (t, *J* = 7.5 Hz, 1H), 6.77 (d, *J* = 8.1 Hz, 1H), 5.90 – 5.63 (m, 1H), 5.23 – 5.06 (m, 2H), 4.40 – 4.16 (m, 2H), 3.92 – 3.75 (m, 2H), 3.56 (dd, *J* = 8.8, 5.0 Hz, 1H), 2.18 (ddd, *J* = 14.6, 10.7, 5.1 Hz, 1H), 2.06 – 1.85 (m, 1H). ^{13}C-NMR (100 MHz, $CDCl_3$) δ: 178.7, 143.3, 131.3, 128.9, 128.1, 123.9, 122.8, 117.8, 109.2, 61.1, 44.6, 42.5, 33.5. IR (KBr, neat) 3404.8, 1709.2, 1613.7, 1489.3, 1467.3, 1051.6, 756.5 cm^{-1}; HRMS (ESI) (*m/z*) *calcd.* for $C_{13}H_{15}NO_2Na$ $[M+Na]^+$ 240.0995; found 240.0994.

Comparison of NBS and *m*-CPBA with our oxone-halide for oxidation of indole and N-Ts skatole.

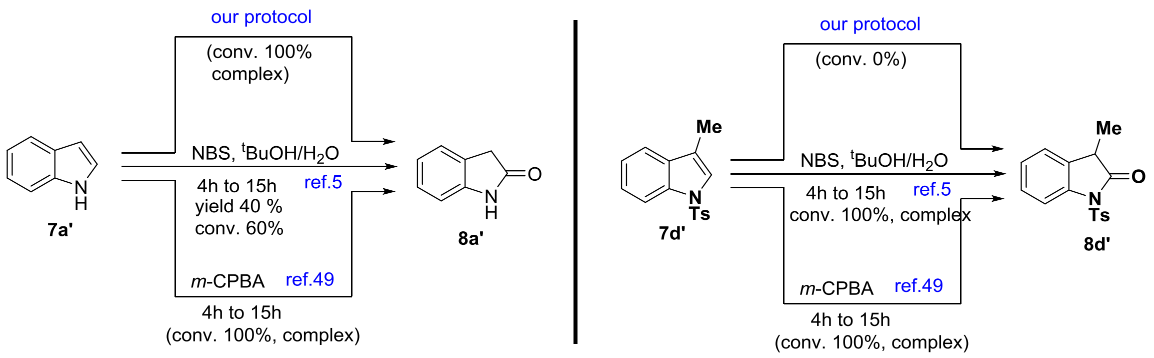

Deuterium experiments

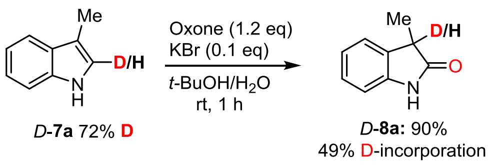

Control 1: Following the **General Procedure-4** for oxidation of C3 substituted indoles to 2-oxindoles, 2-deuterated 3-methyindole[35] (D-**7a**,72% D) (20 mg, 0.13 mmol) was used to provide the 2-oxindole D-**8a** (11.7 mg, 90% yield) with **49%** deuterium incorporation by ^{1}H-NMR integration.

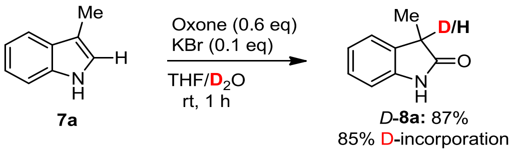

Control 2: Following the **General Procedure-4** for oxidation of C3 substituted indoles to 2-oxindoles, THF/D_2O (5 mL/0.25 mL) was used as the solvent system to replace ^{t}BuOH/H_2O. Reaction of C3-methyl indole (**7a**, 66 mg, 0.50 mmol) provided the 2-oxindole D-**8a** (61.8 mg, 87% yield) with **85 %** deuterium incorporation by ^{1}H-NMR integration.

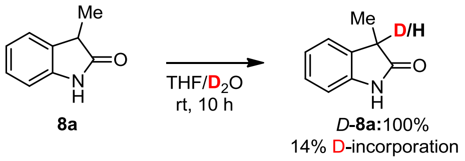

Control 3: A solution of 3-methyindole **8a** (37 mg, 0.25 mmol) in THF/D_2O (3 mL/0.15 mL) was stirred at rt for 10 h. The organic phase was concentrated under reduced pressure and provided the 2-oxindole D-**8a** (100% yield) with **14 %** deuterium incorporation by ^{1}H-NMR integration.

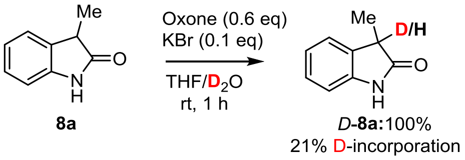

Control 4: under the standard condition of oxidation with THF/D_2O (v/v 20/1), 3-methyindole **8a** (55 mg, 0.37 mmol) was used to replace **7a**. **21%** deuterated 2-oxindole was observed (D-**8a**, ^{1}H-NMR integration).

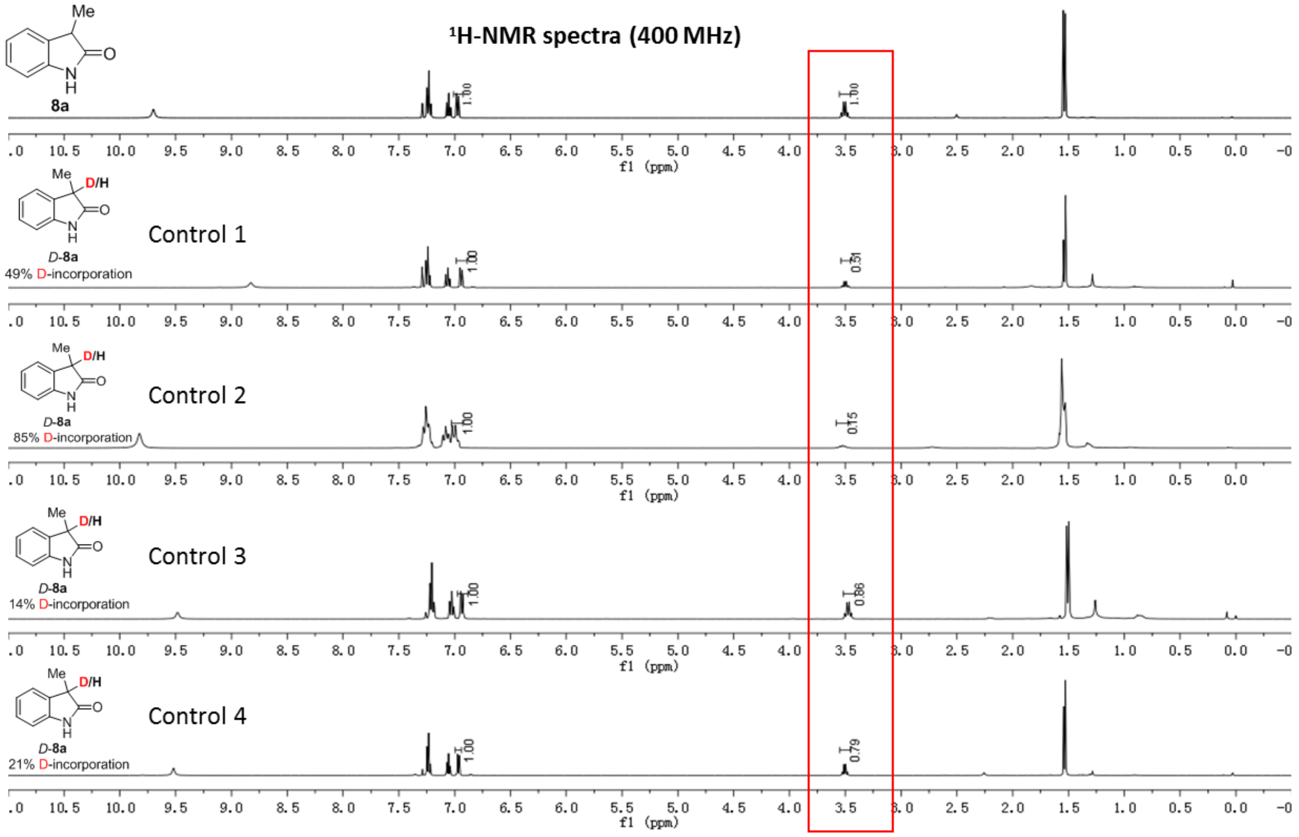

Supplementary Figure 1: H-NMR spectra of deuterium experiments

Total syntheses of (±)-desoxyeseroline, (±)-physovenol methyl ether and (±)-esermethole

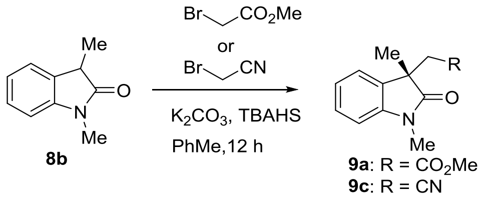

To a solution of **8b** (175 mg, 1.09 mmol) in toluene (10.0 mL) were added BuN_4HSO_4 (36.9 mg, 0.11 mmol) and K_2CO_3 (301 mg, 2.18 mmol). After the reaction mixture was stirred for 10 min, methyl bromoacetate (333.5 mg, 2.18 mmol) or bromoacetonitrile (259.4 mg, 2.18 mmol) in toluene (1.0 mL) were added dropwise via syringe over 30 min. The resulting reaction mixture was stirred at rt for 12 h. The reaction was quenched by addition of water (20 mL) and then diluted with EtOAc (20 mL). The organic fractions were collected, and the aqueous phase was extracted with EtOAc (20 mL × 3). The combined organic layers were washed with brine, dried over Na_2SO_4, filtered and concentrated under reduced pressure. The resulting residue was purified by flash column chromatography with gradient eluents (Hexane/ EtOAc 10:1 to 4:1) to provide 2-oxindole **9a** (177.8 mg, 70% yield) or **9c** (157.0 mg, 72% yield).

9a: ^{1}H-NMR (400 MHz, $CDCl_3$) δ: 7.26 (ddd, J = 7.7, 6.9, 1.2 Hz, 1H), 7.19 (dd, J = 7.3, 0.6 Hz, 1H), 7.03 (td, J = 7.6, 0.9 Hz, 1H), 6.85 (d, J = 7.8 Hz, 1H), 3.44 (s, 3H), 3.25 (s, 3H), 3.00 (d, J = 16.4 Hz, 1H), 2.85 (d, J = 16.4 Hz, 1H), 1.37 (s, 3H).^{13}C-NMR (100 MHz, $CDCl_3$) δ: 180.0, 170.4, 143.7, 133.1, 128.3, 122.5, 122.4,

108.3, 51.7, 45.6, 41.5, 26.5, 24.3. IR (KBr, neat) 2967.6, 1739.8, 1472.0, 1352.7, 1205.3, 1127.2, 756.4 cm^{-1}; HRMS (ESI) (m/z) *calcd.* for $C_{13}H_{15}NO_3Na$ $[M+Na]^+$ 256.0944; found 256.0952.

9c: ^{1}H-NMR (400 MHz, $CDCl_3$) δ: 7.46 (d, *J* = 7.4 Hz, 1H), 7.34 (t, *J* = 7.8 Hz, 1H), 7.12 (t, *J* = 7.6 Hz, 1H), 6.90 (d, *J* = 7.8 Hz, 1H), 3.23 (s, 3H), 2.83 (dd, *J* = 16.7, 0.8 Hz, 1H), 2.56 (d, *J* = 16.7 Hz, 1H), 1.51 (s, 3H).^{13}C-NMR (100 MHz, $CDCl_3$) δ: 177.5, 142.7, 131.0, 129.2, 123.3, 123.1, 116.7, 108.7, 44.9, 26.5, 26.3, 22.2. IR (KBr, neat) 2971.9, 1716.4, 1615.1, 1472.0， 1380.7， 1127.9, 757.7 cm^{-1}; HRMS (ESI) (*m/z*) *calcd.* for $C_{12}H_{12}N_2ONa$ $[M+Na]^+$ 223.0842; found 223.0845.

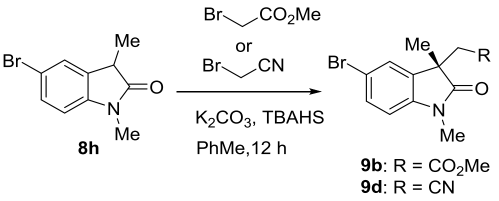

Following the procedure for the synthesis of **9a**/**9c**, reaction of **8h** (110 mg, 0.49 mmol) with methyl bromoacetate (116.6 mg, 0.98 mmol) or bromoacetonitrile (259.4 mg, 2.18 mmol) provided **9b** (111.2 mg, 73% yield) or **9d** (95.4 mg, 70% yield).

9b: ^{1}H-NMR (400 MHz, $CDCl_3$) δ: 7.39 (dd, *J* = 8.2, 1.9 Hz, 1H), 7.29 (d, *J* = 1.9 Hz, 1H), 6.73 (d, *J* = 8.3 Hz, 1H), 3.49 (s, 3H), 3.23 (s, 3H), 3.01 (d, *J* = 16.8 Hz, 1H), 2.83 (d, *J* = 16.8 Hz, 1H), 1.35 (s, 3H).^{13}C-NMR (100 MHz, $CDCl_3$) δ: 179.4, 170.2, 142.9, 135.2, 131.1, 125.7, 115.1, 109.7, 51.8, 45.7, 41.3, 26.6, 24.3. IR (KBr, neat) 2951.9, 1717.6, 1607.9, 1491.0,1346.8, 810.6 cm^{-1}; HRMS (ESI) (*m/z*) *calcd.* for $C_{13}H_{14}BrNO_3$ $[M+Na]^+$ 334.0049; found 334.0041.

9d: ^{1}H-NMR (400 MHz, $CDCl_3$) δ: 7.55 (s, 1H), 7.46 (dd, *J* = 8.3, 0.8 Hz, 1H), 6.78 (d, *J* = 8.3 Hz, 1H), 3.21 (s, 3H), 2.82 (d, *J* = 16.7 Hz, 1H), 2.59 (d, *J* = 16.7 Hz, 1H), 1.50 (s, 3H).^{13}C-NMR (100 MHz, $CDCl_3$) δ: 176.9, 141.9, 132.9, 132.2, 126.4, 116.2, 115.9, 110.2, 45.1, 26.7, 26.2, 22.2. IR (KBr) 2928.7, 1708.7, 1489.6, 1347.9, 1102.0, 824.8, 614.2 cm^{-1}; HRMS (ESI) (*m/z*) *calcd.* for $C_{12}H_{11}BrN_2ONa$ $[M+Na]^+$ 300.9947 ; found 300.9950.

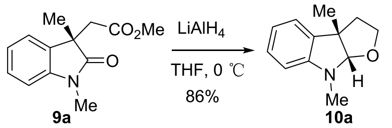

To a solution of **9a** (60 mg, 0.26 mmol) in THF (3 mL) at 0 °C was added $LiAlH_4$ (39.5 mg, 1.04 mmol) in small portions under nitrogen atmosphere. The reaction mixture was stirred at 0 °C for 2 h and then the reaction was quenched with addition of *aq. sat.* brine (10 mL) and diluted with EtOAc (10 mL). The organic fractions were collected, and the aqueous phase was extracted with EtOAc (3 × 10 mL). The combined organic layers were washed with brine, dried over Na_2SO_4, filtered and concentrated under reduced pressure. The resulting residue was purified by flash column chromatography with gradient eluents (Hexane/ EtOAc 20:1 to 5:1) to provide **10a** (42.3 mg, 86% yield). ^{1}H-NMR (400 MHz, $CDCl_3$) δ: 7.10 (td, *J* = 7.7, 1.2 Hz, 1H), 7.04 (dd, *J* = 7.2, 0.7 Hz, 1H), 6.77 – 6.56 (m, 1H), 6.37 (d, *J* = 7.8 Hz, 1H), 5.07 (s, 1H), 3.95 (ddd, *J* = 8.7, 7.3, 1.4 Hz, 1H), 3.46 (ddd, *J* = 11.2, 8.7, 5.2 Hz, 1H), 2.92 (s, 3H), 2.13 (ddd, *J* = 11.8, 5.1, 1.1 Hz, 1H), 2.05 (td, *J* = 11.5,

7.3 Hz, 1H), 1.46 (s, 3H).^{13}C-NMR (100 MHz, $CDCl_3$) δ: 150.6, 134.6, 128.2, 122.6, 117.4, 105.1, 105.0, 77.4, 77.2, 77.0, 67.46, 52.4, 41.8, 31.0, 24.9. IR (KBr, neat) 2959.3, 2867.2, 1608.6, 1494.8, 1388.9, 1301.9, 1033.3, 742.1 cm^{-1}; HRMS (ESI) (*m/z*) *calcd.* for $C_{12}H_{16}NO$ $[M+H]^+$190.1226; found 190.1225.

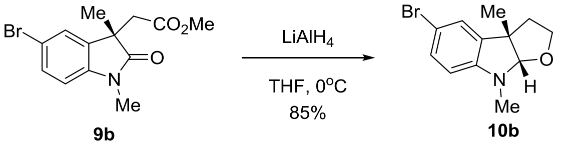

Following the procedure for the synthesis of **10a**, $LiAlH_4$ reduction of **9b** (50 mg, 0.18 mmol) provided **10b** (41.0 mg, 85%). ^{1}H-NMR (400 MHz, $CDCl_3$) δ: 7.17 (dd, *J* = 8.3, 2.0 Hz, 1H), 7.11 (d, *J* = 2.0 Hz, 1H), 6.23 (d, *J* = 8.3 Hz, 1H), 5.05 (s, 1H), 3.95 (ddd, *J* = 8.7, 7.2, 1.5 Hz, 1H), 3.44 (ddd, *J* = 11.2, 8.8, 5.2 Hz, 1H), 2.89 (s, 3H), 2.11 (ddd, *J* = 12.0, 5.1, 1.2 Hz, 1H), 2.07 – 2.01 (m, 1H), 1.44 (s, 3H). ^{13}C-NMR (100 MHz, $CDCl_3$) δ: 149.6, 137.0, 130.8, 125.8, 108.9, 106.4, 105.1, 67.5, 52.5, 41.7, 31.0, 24.7. IR (KBr, neat) 2959.7, 2868.3, 1601.8, 1495.2, 1272.2, 1033.9, 803.0 cm^{-1}; HRMS (ESI) (*m/z*) *calcd.* for $C_{12}H_{15}BrNO$ $[M+H]^+$ 268.0332; found 268.0341.

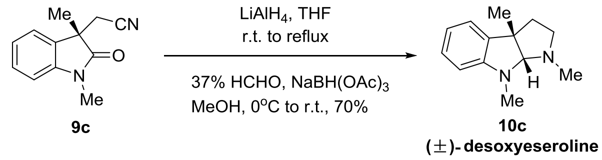

To a solution of **9c** (50 mg, 0.25 mmol) in THF (2.5 mL) at rt was added $LiAlH_4$ (47.5 mg, 1.25 mmol). The reaction mixture was stirred under nitrogen atmosphere for 1 h and then was heated to reflux. After heating at reflux for 10 min, the mixture was quenched at 0 °C by addition of water (20 mL) and then diluted with EtOAc (20 mL). The organic fractions were collected, and the aqueous phase was extracted with EtOAc (30 mL × 3). The combined organic layers were washed with water, brine, dried over Na_2SO_4, filtered and concentrated under reduced pressure. The resulting crude residue was used directly for the next step without further purification.

To a solution of the crude product obtained above in MeOH (5.0 mL) at 0 °C was added *aq.* HCHO (37 wt%, 0.1 mL, 1.0 mmol) under nitrogen atmosphere. After stirring for 5 min, $NaBH(OAc)_3$ (212.0 mg, 1.0 mmol) was added to the reaction mixture. The resulting solution was stirred at rt for 3 h, and then the reaction was quenched at 0 °C by addition of *aq. sat.* $NaHCO_3$ (15 mL) and then diluted with CH_2Cl_2 (15 mL). The organic fractions were collected, and the aqueous phase was extracted with CH_2Cl_2 (3 × 15 mL). The combined organic layers were washed with brine, dried over Na_2SO_4, filtered and concentrated under reduced pressure. The resulting residue was purified by flash column chromatography with gradient eluents (CH_2Cl_2/ MeOH 100:1 to 20:1) to provide (±)-desoxyeseroline (**10c**, 35.4 mg, 70%). ^{1}H-NMR (400 MHz, $CDCl_3$) δ: 7.09 (td, *J* = 7.7, 1.2 Hz, 1H), 7.00 (dd, *J* = 7.2, 0.7 Hz, 1H), 6.68 (td, *J* = 7.4, 0.5 Hz, 1H), 6.42 (d, *J* = 7.8 Hz, 1H), 4.13 (s, 1H), 2.95 (s, 3H), 2.78 – 2.68 (m, 1H), 2.64 (td, *J* = 8.6, 6.7 Hz, 1H), 2.56 (s, 3H), 2.05 – 1.82 (m, 2H), 1.45 (s, 3H). ^{13}C-NMR (100 MHz, $CDCl_3$) δ: 152.0, 136.7, 127.8, 122.4, 117.7, 106.7, 97.5, 53.3, 52.8, 40.9, 38.5, 36.7, 27.5. IR (KBr, neat) 2957.4, 2864.1, 1492.0, 1299.5, 1255.5 1124.6, 1034.6, 958.3 cm^{-1}; HRMS (ESI) (*m/z*) *calcd.* for $C_{13}H_{19}N_2$ $[M+H]^+$ 203.1543; found 203.1546.

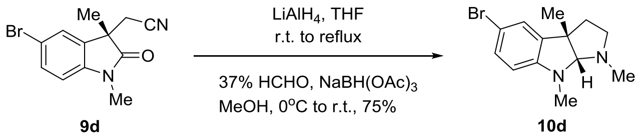

Following the procedure for the synthesis of **10c**, reductive cyclization of **9d** (100 mg, 0.36 mmol) provided **10d** (75.6 mg, 75% yield). ^{1}H-NMR (400 MHz, $CDCl_3$) δ: ^{1}H NMR (600 MHz, $CDCl_3$) δ 7.16 (dd, *J* = 8.3, 2.0 Hz, 1H), 7.06 (d, *J* = 2.0 Hz, 1H), 6.27 (d, *J* = 8.3 Hz, 1H), 4.17 (s, 1H), 2.93 (s, 3H), 2.82 – 2.71 (m, 1H), 2.63 (td, *J* = 9.1, 6.3 Hz, 1H), 2.55 (s, 3H), 1.98 (m, 2H), 1.42 (s, 3H).^{13}C-NMR (100 MHz, $CDCl_3$) δ: 150.8, 138.9, 130.6, 125.5, 109.4, 108.1, 97.4, 53.2, 52.9, 40.5, 38.3, 36.5, 27.2. IR (KBr) 2929.5, 2866.3, 1598.9, 1493.0, 1268.0, 1128.5, 1035.5 cm^{-1}; HRMS (ESI) (*m/z*) *calcd.* for $C_{13}H_{18}BrN_2$ $[M+H]^+$281.0648; found 281.0656.

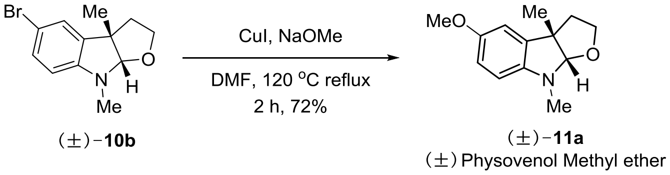

Following the Ullmann procedure for the synthesis of (±)-horsfiline (**4**) (Page S-19), reaction of **10b** (0.14 mmol, 38.0 mg) provided **11a** (±)-physovenol methyl ether (22.1 mg, 72%). ^{1}H-NMR (400 MHz, $CDCl_3$) δ: 6.70 (d, *J* = 2.4 Hz, 1H), 6.66 (dd, *J* = 8.3, 2.3 Hz, 1H), 6.30 (d, *J* = 8.1 Hz, 1H), 5.04 (s, 1H), 4.03 – 3.87 (m, 1H), 3.75 (s, 3H), 3.47 (ddd, *J* = 11.0, 8.6, 5.3 Hz, 1H), 2.88 (s, 3H), 2.19 – 2.09 (m, 1H), 2.04 (m, 1H), 1.45 (s, 3H). ^{13}C-NMR (100 MHz, $CDCl_3$) δ:152.9, 145.0, 136.2, 112.3, 110.6, 105.7, 105.5, 67.6, 56.2, 52.6, 41.6, 31.8, 24.6. IR (KBr, neat) 2931.3, 2866.5, 1498.8, 1448.6, 1425.8, 1281.1, 1033.9 cm^{-1}; HRMS (ESI) (*m/z*) *calcd.* for $C_{13}H_{18}NO_2$ $[M+H]^+$ 220.1332; found 220.1338.

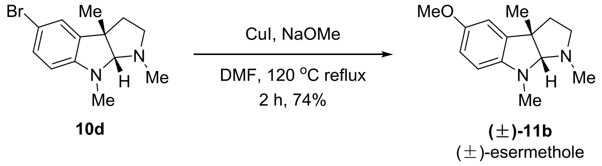

Following the Ullmann procedure for the synthesis of (±)-horsfiline (**4**) (Page S-19), reaction of **10d** (28.2 mg, 0.10 mmol) provided (±)-esermethole (**11b**, 17.2 mg,74%). ^{1}H-NMR (400 MHz, $CDCl_3$) δ: 6.65 (dd, *J* = 8.4, 2.5 Hz, 1H), 6.62 (d, *J* = 2.4 Hz, 1H), 6.37 (d, *J* = 8.4 Hz, 1H), 4.18 (s, 1H), 3.73 (s, 3H), 2.90 (s, 3H), 2.85 – 2.79 (m, 1H), 2.61 (td, *J* = 9.4, 6.4 Hz, 1H), 2.54 (s, 3H), 2.03 (m, 1H), 1.97 (m, 1H), 1.44 (s, 3H).^{13}C-NMR (100 MHz, $CDCl_3$) δ: 153.4, 146.2, 137.8, 112.5, 109.8, 107.9, 97.9, 56.1, 53.1, 53.0, 40.4, 38.2, 37.5, 27.2. IR (KBr, neat) 2955.8, 2865.6, 1498.5, 1281.0, 1220.8, 1121.5, 1032.5 cm^{-1}; HRMS (ESI) (*m/z*) *calcd.* for $C_{14}H_{21}N_2O$ $[M+H]^+$ 233.1648; found 233.1654.

Preparation of indole derivatives

Substrates **12a**, **12f**, **12g**, **12h**, **12i** and **12m** are commercially available. **12b**[36], **12c-2**[37], and **12n**[38], **12c-1**[39], **12j**, **12k**[40], **12l**[41], **12o**[42] and **12p**[43] are known compounds and reported in the related literatures. **12d**[44] and **12e**[45]were prepared by Fischer indole synthesis[46].

1.2.12 Oxidative cleavage (Witkop oxidation) of indoles to 2-keto acetanilides.

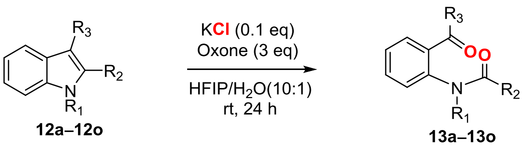

General Procedure-5 (Witkop Oxidation): A 0.01 M stock solution of KCl in HFIP/H_2O (v/v 10:1) was prepared as follows: KCl (14.9 mg, 0.2 mmol) was dissolved in 20.0 mL of HFIP/H_2O (v/v 10:1). To a solution of indole derivatives (**12a-12n**) (0.2 mmol) and KCl (2.4 mg, 0.02 mol) in HFIP/H_2O (v/v 10:1) (2.0 mL, 0.1 M) at rt was added oxone (184 mg, 0.6 mmol, MW 307) in one batch. The reaction mixture was stirred at rt for 24 h and then diluted with EtOAc (4 – 6 mL). The reaction mixture passed through a short pad of silica gel and washed with EtOAc. The resulting EtOAc/HFIP solution was concentrated under reduced pressure and the residue was purified by flash column chromatography to give 2-keto acetanilides **13a-13n**.

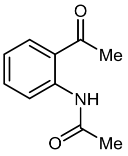

13a

13a47 (76.1 mg, 76% yield): ^{1}H-NMR(400 MHz, $CDCl_3$) δ 11.67 (s, 1H), 8.71 (d, *J* = 8.4 Hz, 1H), 7.87 (dd, *J* = 8.0, 1.6 Hz, 1H), 7.56 – 7.49 (m, 1H), 7.13 – 7.05 (m, 1H), 2.64 (s, 3H), 2.21 (s, 3H). ^{13}C-NMR (100 MHz, $CDCl_3$) δ 202.8, 169.2, 140.9, 135.005, 131.5, 122.2, 121.6, 120.6, 28.5, 25.5. IR (neat) 1647.1, 1583.0, 1514.6, 1441.2, 1360.7, 1302/7, 1235.7, 956.7, 763.5, 602.9 cm^{-1}; HRMS (CI+) (m/z) *calcd.* for $C_{10}H_{11}NO_2$ $[M]^+$ 177.0790; found 177.0793.

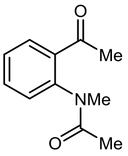

13b

13b (20.2 mg, 53% yield): ^{1}H-NMR (400 MHz, $CDCl_3$) δ 7.68 (ddd, *J* = 45.1, 7.7, 1.5 Hz, 1H), 7.53 (dtd, *J* = 24.8, 7.7, 1.6 Hz, 1H), 7.48 – 7.31 (m, 1H), 7.25 – 7.16 (m, 1H), 3.27 (d, *J* = 67.3 Hz, 3H), 2.53 (s, 3H), 1.98 (d, *J* = 164 Hz 3H). ^{13}C-NMR (100 MHz, $CDCl_3$) δ 200.3, 199.2, 171.4,170.5, 142.3, 141.3, 137.1, 136.7, 133.1, 132.4, 130.0, 129.4, 128.5, 128.4, 127.9, 127.2, 40.0, 37.1, 29.4, 28.7, 22.2. IR (neat) 1687.8, 1653.7, 1597.7, 1484.3, 1433.4, 1382.9, 1286.3, 1248.2, 1146.0, 768.8, 599.9 cm^{-1}; HRMS (CI+) (m/z) *calcd.* for $C_{11}H_{13}NO_2$ $[M+H]^+$ 192.1025; found 192.1028.

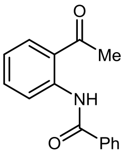

13d

13d[45] (30.8 mg, 64% yield): ^{1}H-NMR (400 MHz, $CDCl_3$) δ 12.71 (s, 1H), 8.99 (dd, *J* = 8.5, 1.2 Hz, 1H), 8.10 – 8.05 (m, 2H), 7.96 (dd, *J* = 8.0, 1.6 Hz, 1H), 7.68 – 7.48 (m, 4H), 7.19 – 7.12 (m, 1H), 2.72 (s, 3H). ^{13}C-NMR (100 MHz, $CDCl_3$) δ 203.3, 166.1, 141.4, 135.4, 134.8, 132.0, 131.8, 128.8, 127.5, 122.5, 121.9, 120.8, 28.6. IR (neat) 1648.8, 1583.0, 1523.7, 1444.2, 1358.1, 1307.2, 1249.4, 756.5, 702.4 cm^{-1}; HRMS (CI^+) (m/z) *calcd.* for $C_{15}H_{13}NO_2$ $[M]^+$ 239.0946; found 239.0934.

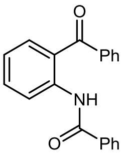

13e

13e[48] (43.2 mg, 72% yield): ^{1}H-NMR (400 MHz, $CDCl_3$) δ 12.71 (s, 1H), 8.99 (dd, *J* = 8.5, 1.2 Hz, 1H), 8.10 – 8.05 (m, 2H), 7.96 (dd, *J* = 8.0, 1.6 Hz, 1H), 7.68 – 7.48 (m, 4H), 7.19 – 7.12 (m, 1H), 2.72 (s, 3H). ^{13}C-NMR (100 MHz, $CDCl_3$) δ 200.3, 165,9, 141.1, 138.8, 134.6, 134.6, 134.0, 132.4, 132.0, 129.8, 128.8, 128.3, 127.4, 123.1, 122.1, 121.4. IR (neat) 1677.8, 1583.4, 1522.3, 1441.7, 1258.8, 932.4, 756.1, 696.5 cm^{-1}; HRMS (CI^+) (m/z) *calcd.* for $C_{20}H_{15}NO_2$ $[M]^+$ 301.1103; found 301.1100.

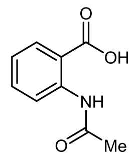
13f

13f[49] (21.5 mg, 60% yield): ^{1}H-NMR (400 MHz, MeOD) δ 8.49 (d, *J* = 8.4 Hz, 1H), 8.04 (dd, *J* = 7.9, 1.6 Hz, 1H), 7.54 – 7.44 (m, 1H), 7.09 (t, *J* = 7.6 Hz, 1H), 2.16 (s, 3H). ^{13}C-NMR (100 MHz, MeOD) δ 170.3, 170.0, 140.8, 133.4, 131.1, 122.5, 119.9, 117.0, 23.6. IR (neat) 2921.1, 1586.4, 1524.3, 1237.8, 756.9 cm^{-1}; HRMS (CI^{+}) (m/z) *calcd.* for $C_9H_9NO_3$ [M]$^{+}$ 179.0582; found 179.0594.

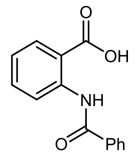
13g

13g[50] (31.7 mg, 66% yield): ^{1}H-NMR (400 MHz, MeOD) δ 8.71 (d, *J* = 8.4 Hz, 1H), 8.06 (dd, *J* = 8.0, 1.6 Hz, 1H), 7.98 – 7.87 (m, 2H), 7.48 (dt, *J* = 26.7, 7.6 Hz, 4H), 7.08 (t, *J* = 7.6 Hz, 1H).^{13}C-NMR (100 MHz, MeOD) δ 170.5, 166.0, 141.4, 134.6, 133.9, 131.9, 131.3, 128.5, 127.0, 122.7, 119.8, 116.2. IR (neat) 3060.9, 1658.9, 1586.4, 1524.0, 1446.3, 1303.3, 1223.5, 758.0, 699.1 cm^{-1}; HRMS (CI^{+}) (m/z) *calcd.* for $C_{14}H_{11}NO_3$ [M]$^{+}$ 241.0739; found 241.0735.

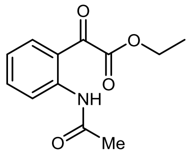
13h

13h (33.3 mg, 70% yield): ^{1}H-NMR (400 MHz, $CDCl_3$) δ 11.10 (s, 1H), 8.78 (d, *J* = 8.5 Hz, 1H), 7.72 – 7.60 (m, 2H), 7.19 – 7.10 (m, 1H), 4.46 (q, *J* = 7.1 Hz, 2H), 2.25 (s, 3H), 1.43 (t, *J* = 7.1 Hz, 3H). ^{13}C-NMR (100 MHz, $CDCl_3$) δ 190.6, 168.5, 163.5, 142.7, 137.1, 132.5, 122.5, 120.7, 116.9, 62.6, 25.5, 14.1. IR (neat) 3314.4, 2982.2, 2932.2, 1735.0, 1651.5, 1581.9, 1523.1, 1447.8, 1286.9, 1191.3, 1011.8, 751.9 cm^{-1}; HRMS (CI+) (m/z) *calcd.* for $C_{12}H_{13}NO_4$ [M]$^{+}$ 235.0845; found 235.0848.

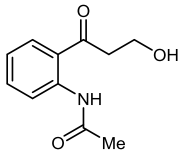
13j

13j (19.2 mg, 46% yield): ^{1}H-NMR (400 MHz, $CDCl_3$) δ 11.61 (s, 1H), 8.71 (d, *J* = 8.4 Hz, 1H), 7.89 (dd, *J* = 8.1, 1.6 Hz, 1H), 7.63 – 7.48 (m, 1H), 7.14 – 7.05 (m, 1H), 4.02 (t, *J* = 5.4 Hz, 2H), 3.28 (t, *J* = 5.4 Hz, 2H), 2.22 (s, 3H). ^{13}C-NMR (100 MHz, $CDCl_3$) δ 204.4, 169.5, 141.0, 135.4, 130.8, 122.4, 121.3, 120.8, 57.9, 41.8, 25.5. IR (neat) 3267.3, 2926.6, 1649.3, 1582.8, 1521.1, 1450.0, 1366.9, 1302.0, 1200.4, 1036.5, 756.1 cm^{-1}; HRMS (CI+) (m/z) *calcd.* for $C_{11}H_{13}NO_3$ [M]$^{+}$ 207.0895; found 207.0995.

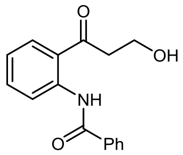
13k

13k (31.4 mg, 46% yield): ^{1}H-NMR (400 MHz, $CDCl_3$) δ 12.58 (s, 1H), 8.95 (d, *J* = 8.3 Hz, 1H), 8.06 – 8.01 (m, 2H), 7.94 (dd, *J* = 8.0, 1.6 Hz, 1H), 7.64 – 7.47 (m, 4H), 7.17 – 7.10 (m, 1H), 4.05 (t, *J* = 5.4 Hz, 2H), 3.32 (t, *J* = 5.4 Hz, 2H).^{13}C-NMR (100 MHz, $CDCl_3$) δ 204.8, 166.1, 141.4, 135.6, 134.6, 132.0, 131.0, 128.8, 127.4, 122.6, 121.6, 120.9, 57.9, 41.8. IR (neat) 3249.4, 2924.1, 1648.5, 1582.8, 1524.3, 1446.8, 1305.7, 1255.2, 1036.7, 757.0, 703.9 cm^{-1}; HRMS (CI+) (m/z) *calcd.* for $C_{16}H_{15}NO_3$ [M]$^{+}$ 269.1052; found 269.1044.

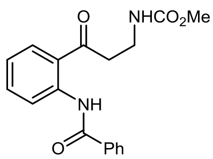
13l

13l (43.4 mg, 66% yield): 1H-NMR (400 MHz, $CDCl_3$) δ 12.61 (s, 1H), 8.98 (d, *J* = 8.5 Hz, 1H), 8.06 (d, *J* = 7.2 Hz, 2H), 7.96 (d, *J* = 8.0 Hz, 1H), 7.59 (dq, *J* = 23.0, 7.1, 6.4 Hz, 4H), 7.16 (t, *J* = 7.6 Hz, 1H), 5.29 (s, 1H), 3.69 – 3.55 (m, 5H), 3.34 (t, *J* = 5.4 Hz, 2H).^{13}C-NMR (100 MHz, $CDCl_3$) δ 203.82, 166.13, 157.0, 141.5, 135.6, 134.8, 132.1, 132.0, 128.8,

127.5, 122.6, 121.5, 121.0, 52.1, 39.9, 36.0. IR (neat) 3328.0, 2949.1, 2922.3, 1709.6, 1649.9, 1582.6, 1522.8, 1445.9, 1300.7, 1199.3, 757.7, 704.2 cm^{-1}; HRMS (CI+) (m/z) *calcd.* for $C_{18}H_{18}N_2O_4$ $[M]^+$ 326.1267; found 326.1257.

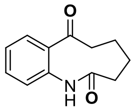

13m

13m[51] (72.2 mg, 71% yield): ^{1}H-NMR (400 MHz, $CDCl_3$) δ 8.31 (s, 1H), 7.58 (d, *J* = 7.6 Hz, 1H), 7.49 (t, *J* = 6.9 Hz, 1H), 7.38 (t, *J* = 7.6 Hz, 1H), 7.23 (d, *J* = 7.8 Hz, 1H), 2.87 (s, 2H), 2.21 (s, 2H), 1.85 (s, 4H).^{13}C-NMR (100 MHz, $CDCl_3$) δ 206.0, 176.7, 138.8, 134.5, 132.1, 128.6, 128.3, 127.9, 41.2, 32.2, 24.6, 24.4. IR (neat) 2928.3, 2860.7, 1655.7, 1599.4, 1446.1, 1386.0, 1333.4, 1278.7, 1237.9, 758.1 cm-1; HRMS (CI+) (m/z) *calcd.* for $C_{12}H_{13}NO_2$ $[M]^+$ 203.0946; found 203.0926.

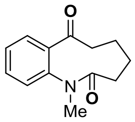

13n

13n (28.6 mg, 66% yield): ^{1}H-NMR (400 MHz, $CDCl_3$) δ 7.55 – 7.49 (m, 1H), 7.48-7.4 (m, 2H), 7.25 (d, *J* = 7.9 Hz, 1H), 3.17 (s, 3H), 2.89 (ddd, *J* = 15.7, 9.1, 2.9 Hz, 1H), 2.66 (ddd, *J* = 15.8, 9.3, 2.8 Hz, 1H), 2.27 (ddd, *J* = 13.3, 6.9, 3.2 Hz, 1H), 2.13 (ddd, *J* = 13.4, 11.3, 3.1 Hz, 1H), 1.98 (dddd, *J* = 16.3, 9.2, 5.6, 3.0 Hz, 1H), 1.87 – 1.76 (m, 1H), 1.72 (ddt, *J* = 14.7, 7.5, 3.2 Hz, 1H), 1.60 – 1.47 (m, 1H).^{13}C-NMR (100 MHz, $CDCl_3$) δ 206.5, 173.7, 141.0, 140.1, 131.9, 128.4, 128.2, 127.7, 42.5, 37.8, 34.0, 25.7, 24.8. IR (neat) 2932.2, 2863.1, 1645.6, 1448.0, 1380.5, 1284.6, 1103.6, 771.2 cm^{-1}; HRMS (CI+) (m/z) *calcd.* for $C_{13}H_{15}NO_2$ $[M]^+$ 217.1103; found 217.1111.

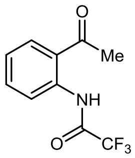

13o

13o: Following the **General Procedure-5**, reaction of indole **12o** (22 mg, 0.0951 mmol) with KCl (0.95 mL, 0.01 M, 0.01 mmol) and oxone (87 mg, 0.29 mmol, MW 307) provided 2-keto acetanilides **13o** (11 mg, 46% yield). ^{1}H-NMR (400 MHz, $CDCl_3$) δ 12.92 (s, 1H), 8.69 (dd, J = 8.4, 1.1 Hz, 1H), 7.99 (dd, J = 8.0, 1.6 Hz, 1H), 7.64 (ddd, J = 8.7, 7.4, 1.6 Hz, 1H), 7.29 (ddd, J = 8.5, 7.5, 1.2 Hz, 1H), 2.71 (s, 3H).^{13}C-NMR (100 MHz, $CDCl_3$) δ 203.16, 156.20, 155.83, 155.45, 155.08, 138.43, 135.44, 131.87, 124.64, 122.58, 121.05, 119.96, 117.09, 114.22, 111.35, 28.30. ^{19}F-NMR (376.5 MHz, $CDCl_3$) δ -76.27. IR (neat) 1721.7, 1661.9, 1591.8, 1536.1, 1455.7, 1362.9, 1286.6, 1253.6, 1158.8, 1142.3, 962.9, 901.0, 771.1, 746.4cm^{-1}; HRMS (CI+) (m/z) *calcd.* for $C_{10}H_8F_3NO_2$ $[M+H]^+$ 232.0580; found 232.0595.

Copies of NMR spectra

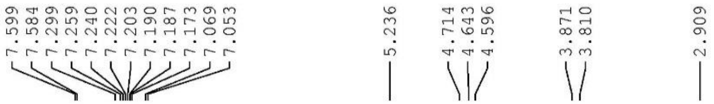

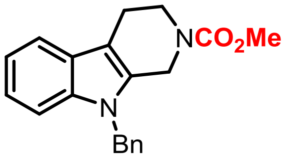

1c

NAME	xt2096
EXPNO	1
PROCNO	1
Date_	20170518
Time	16.42
INSTRUM	spect
PROBHD	5 mm PABBO BB/
PULPROG	zg30
TD	65536
SOLVENT	CDCl3
NS	15
DS	2
SWH	8012.820 Hz
FIDRES	0.122266 Hz
AQ	4.0894966 sec
RG	25.32
DW	62.400 usec
DE	6.50 usec
TE	296.3 K
D1	1.00000000 sec
TD0	1
======== CHANNEL f1 ========	
SFO1	400.1324710 MHz
NUC1	1H
P1	14.50 usec
SI	65536
SF	400.1300099 MHz
WDW	EM
SSB	0
LB	0.30 Hz
GB	0
PC	1.00

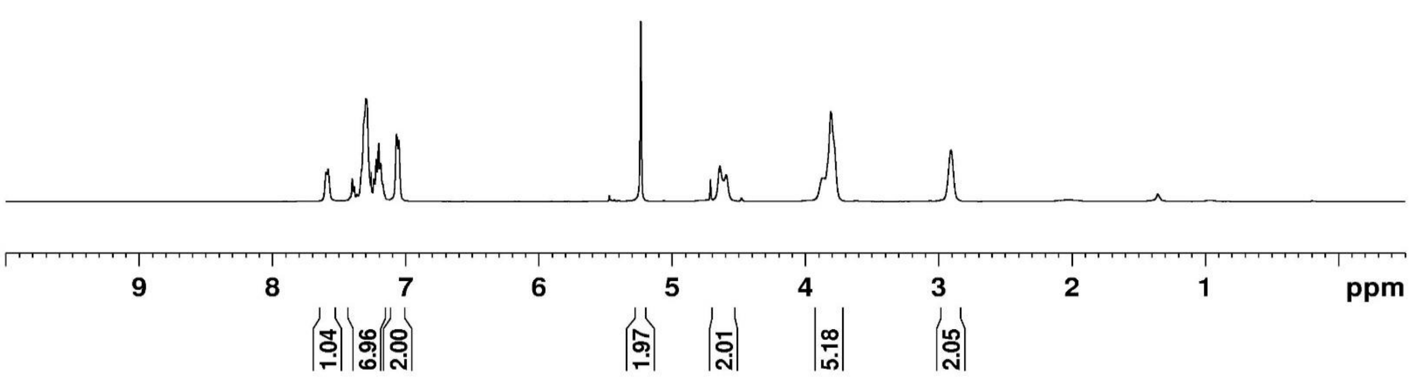

Supplementary Figure 2. ^{1}**H-NMR of 1c**

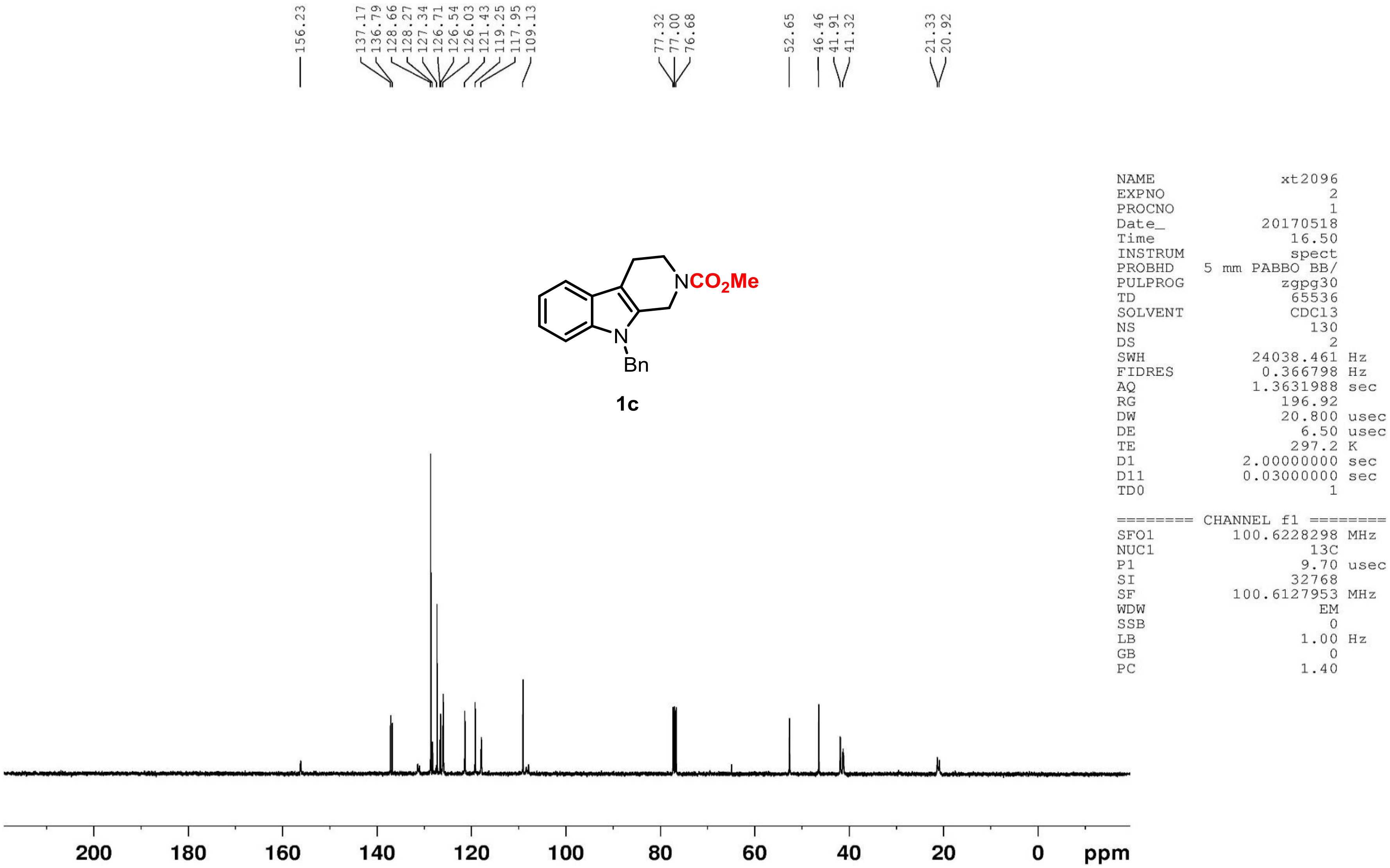

NAME	xt2096	
EXPNO	2	
PROCNO	1	
Date_	20170518	
Time	16.50	
INSTRUM	spect	
PROBHD	5 mm PABBO BB/	
PULPROG	zgpg30	
TD	65536	
SOLVENT	CDCl3	
NS	130	
DS	2	
SWH	24038.461	Hz
FIDRES	0.366798	Hz
AQ	1.3631988	sec
RG	196.92	
DW	20.800	usec
DE	6.50	usec
TE	297.2	K
D1	2.00000000	sec
D11	0.03000000	sec
TD0	1	
======== CHANNEL f1 ========		
SFO1	100.6228298	MHz
NUC1	13C	
P1	9.70	usec
SI	32768	
SF	100.6127953	MHz
WDW	EM	
SSB	0	
LB	1.00	Hz
GB	0	
PC	1.40	

Supplementary Figure 3. ^{13}C-NMR of 1c

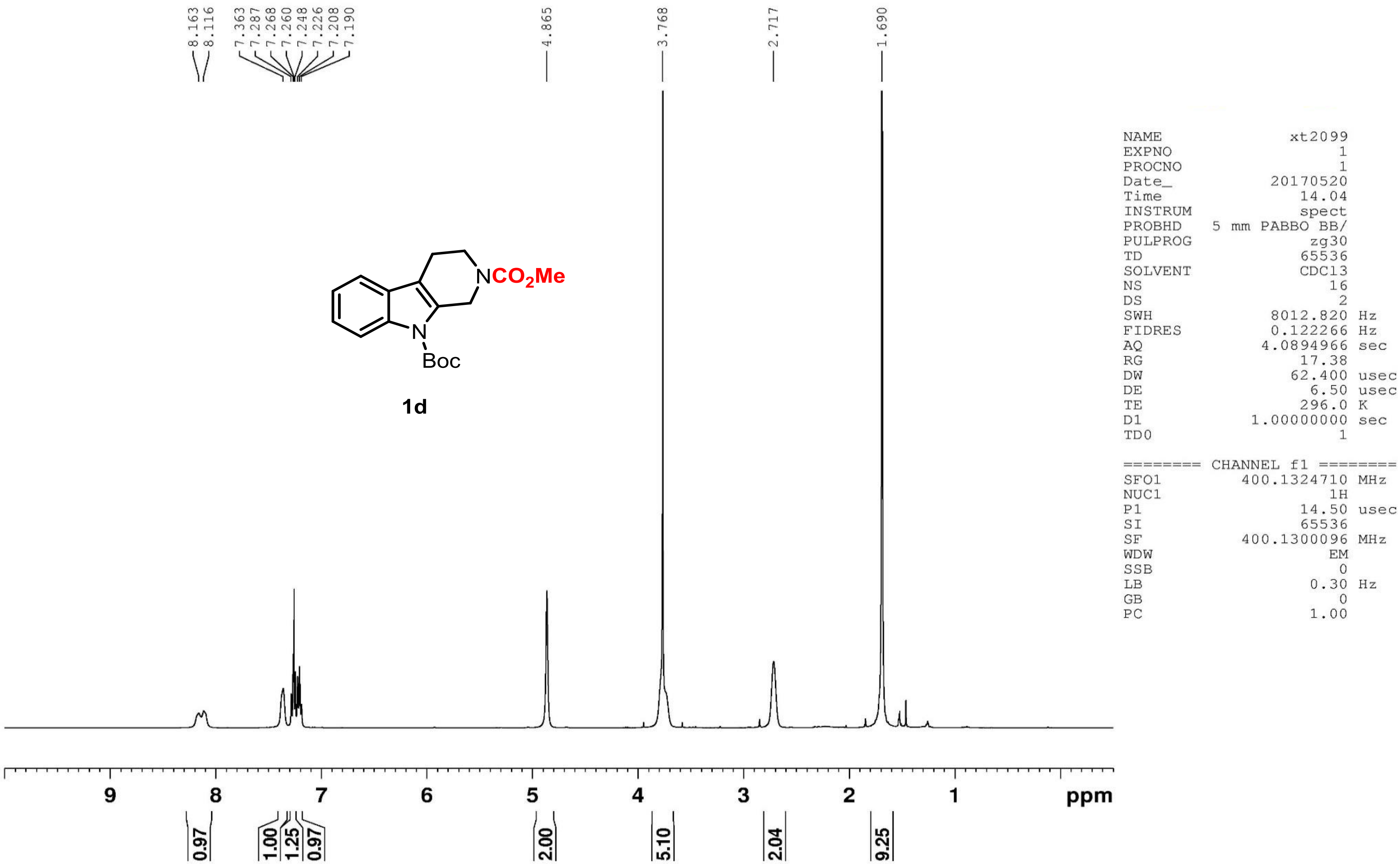

Supplementary Figure 4. ^{1}H-NMR of 1d

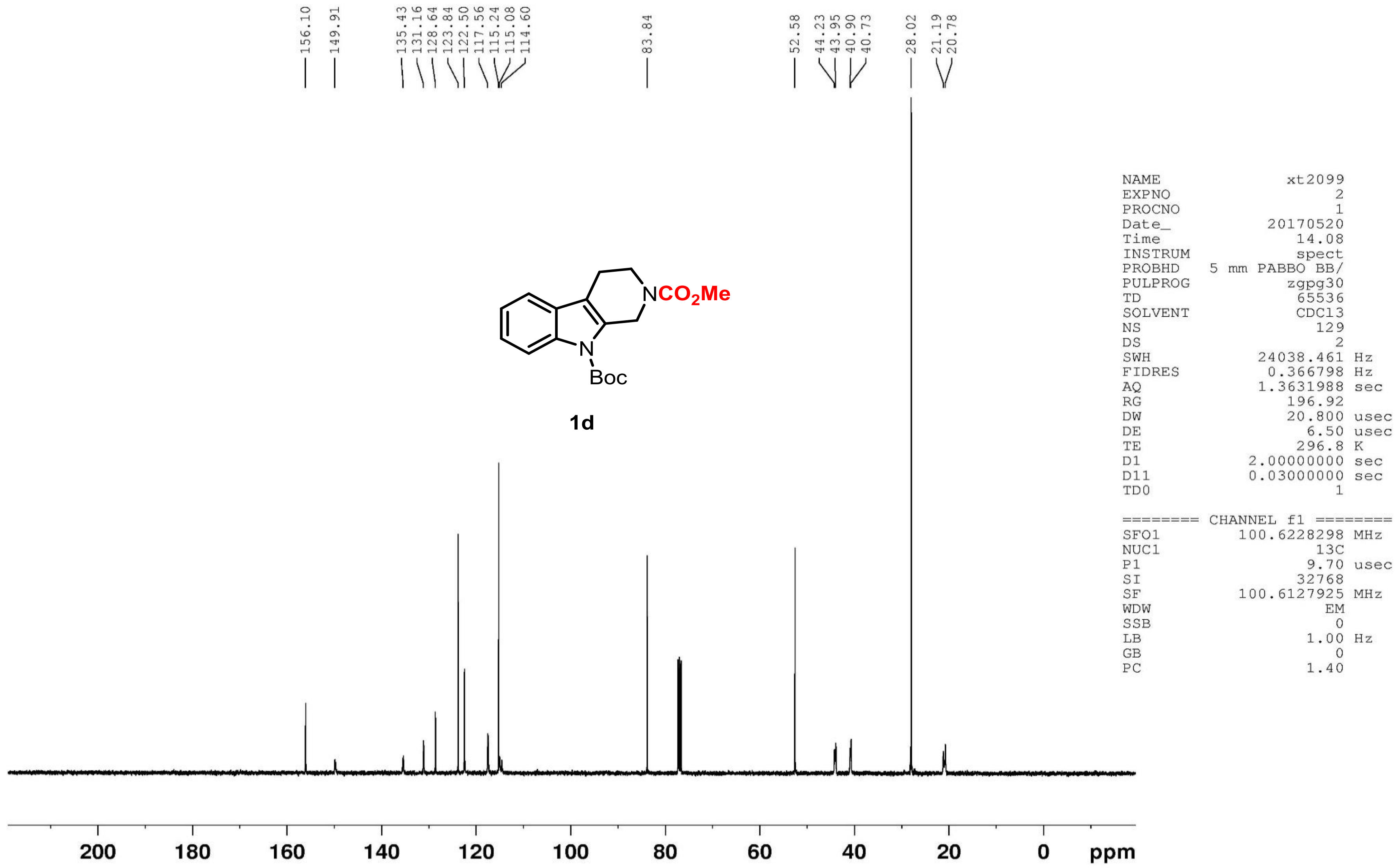

Supplementary Figure 5. ^{13}C-NMR of 1d

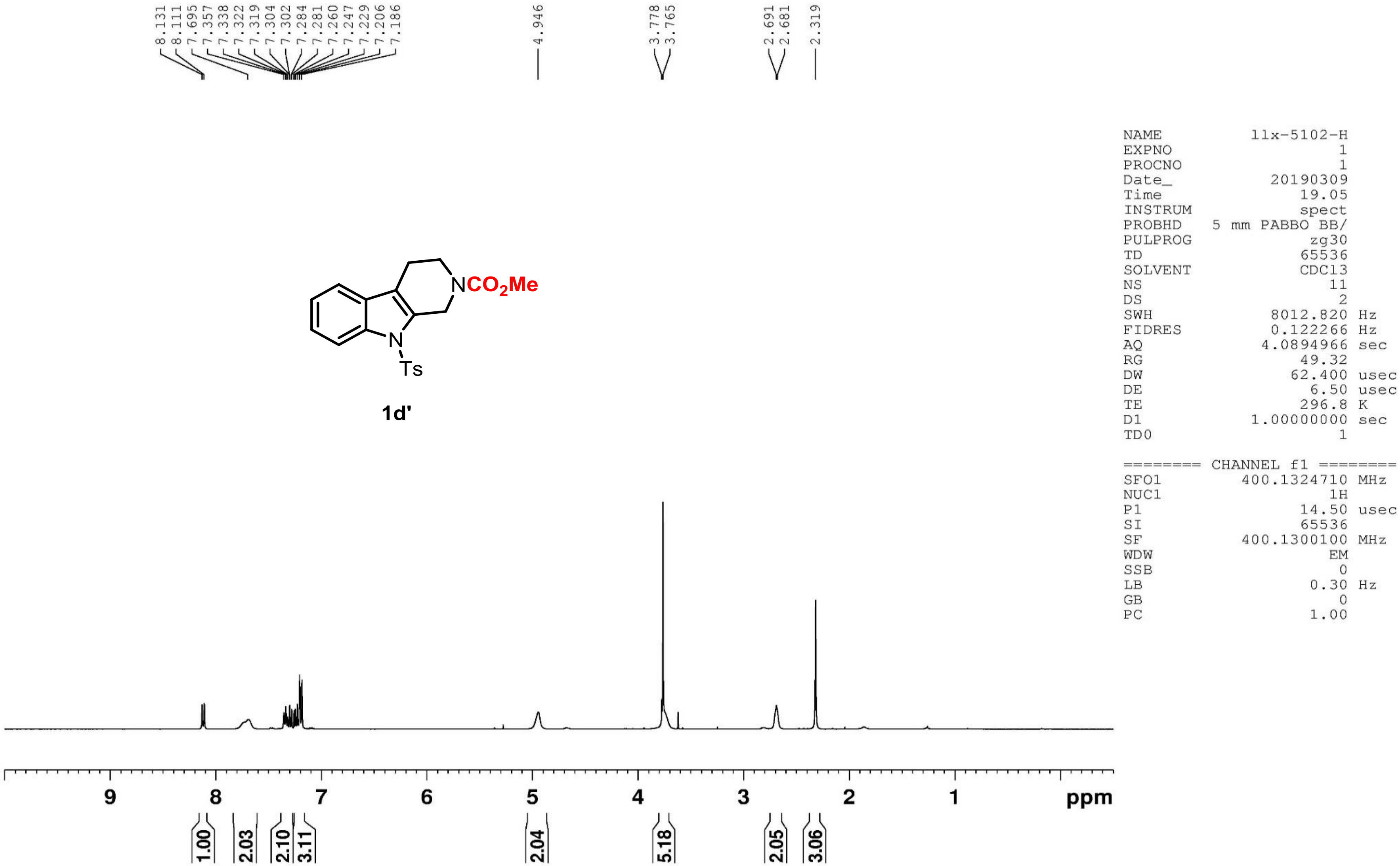

Supplementary Figure 6. ^{1}H-NMR of 1d'

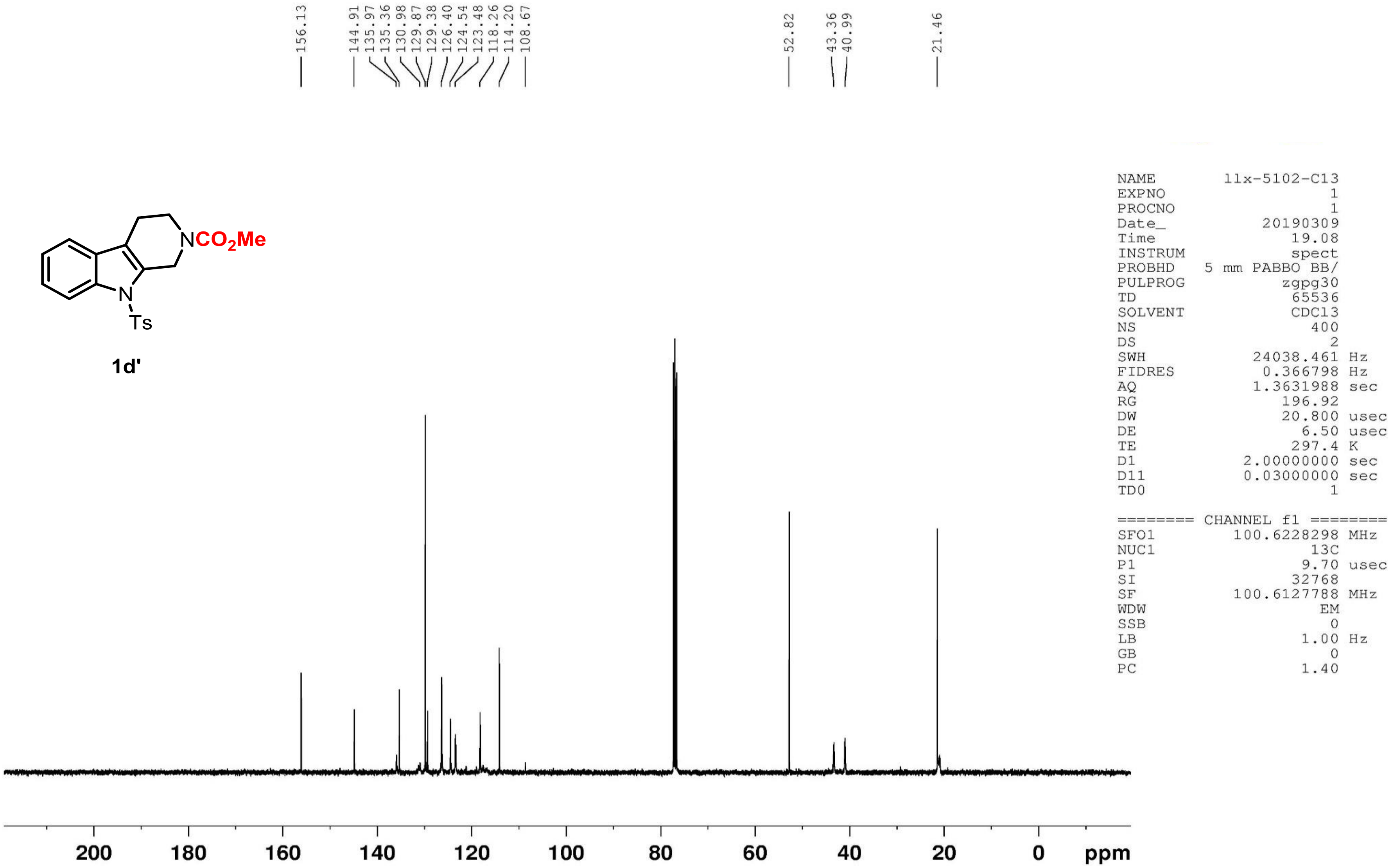

Supplementary Figure 7. ^{13}C-NMR of 1d'

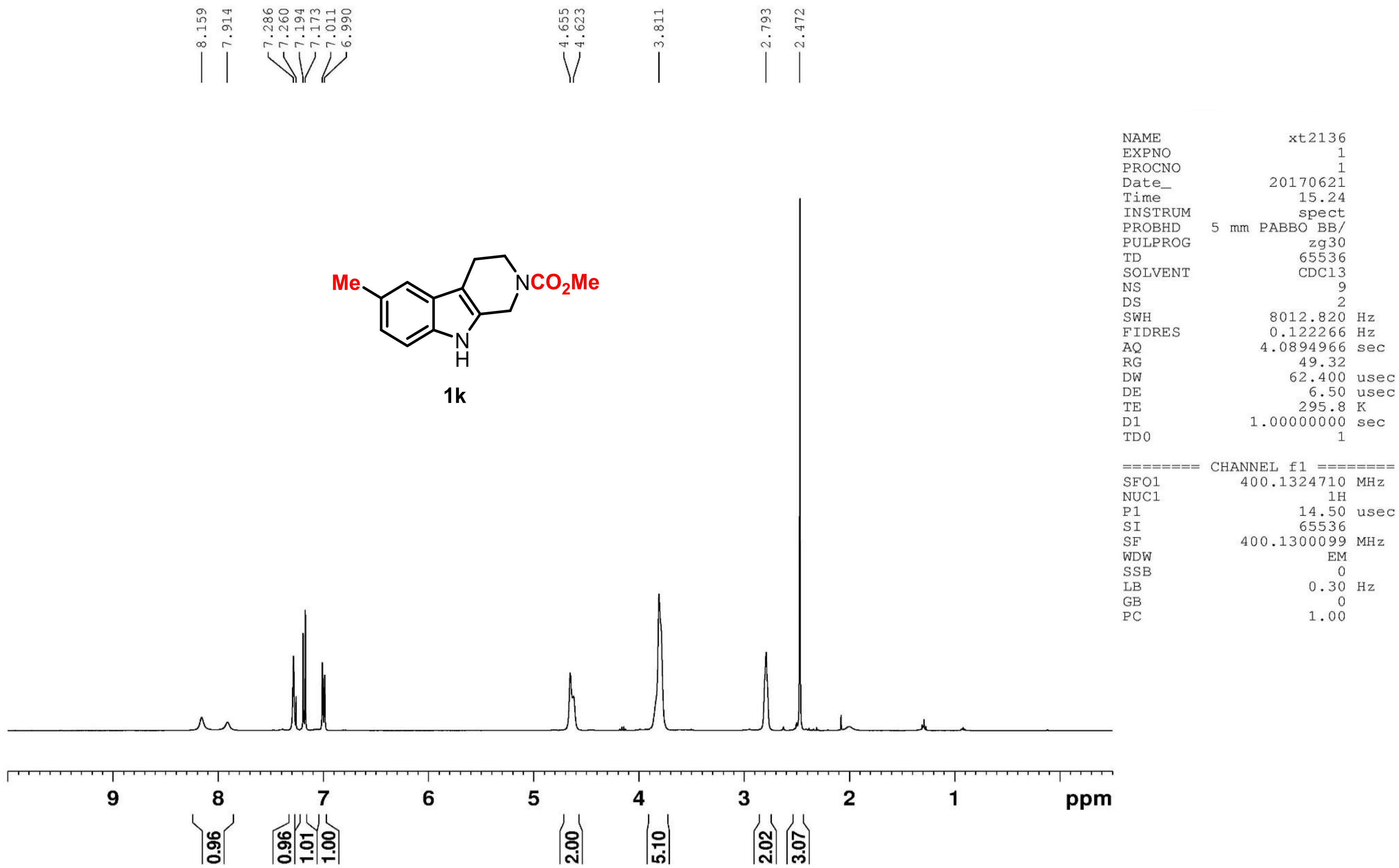

Supplementary Figure 8. ^{1}H-NMR of 1k

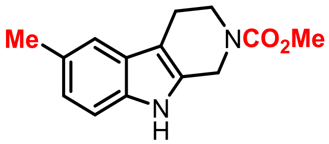

1k

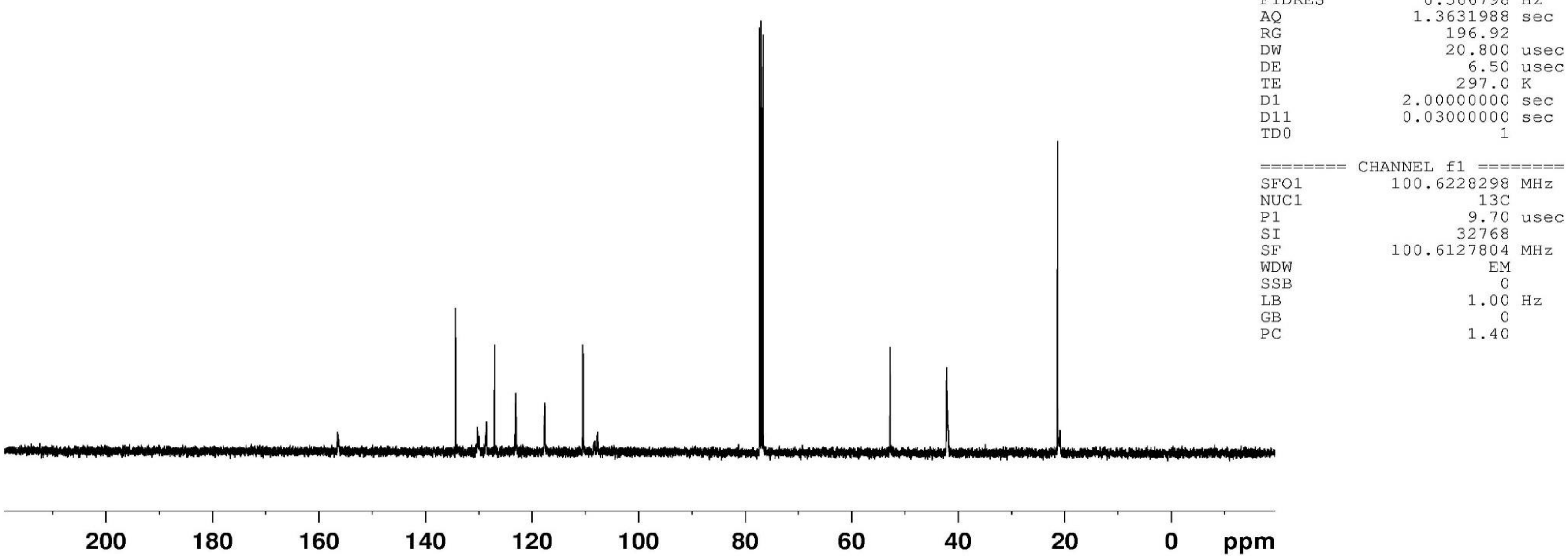

Supplementary Figure 9. ^{13}C-NMR of 1k

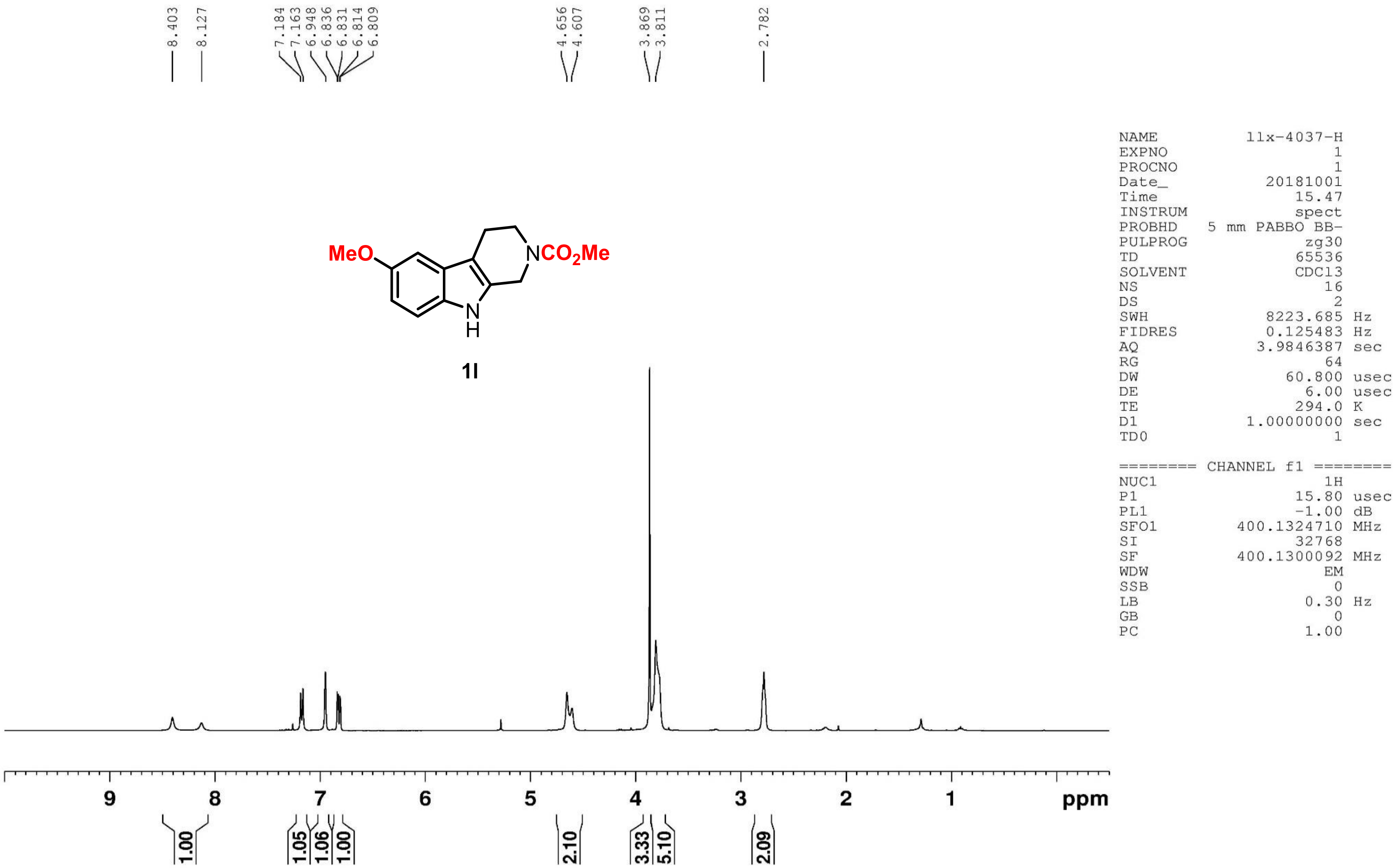

Supplementary Figure 10. ^{1}H-NMR of 1l

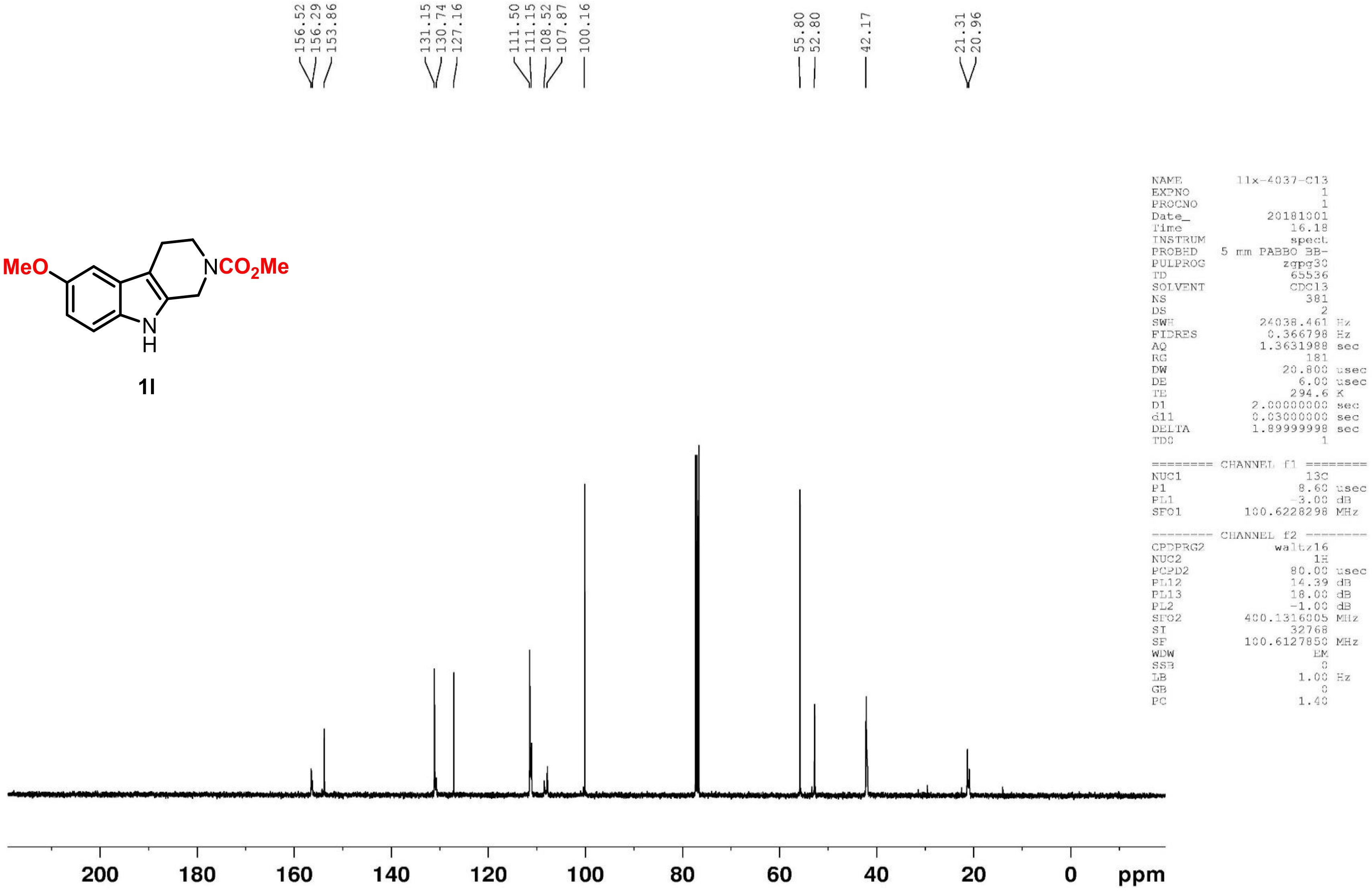

Supplementary Figure 11. ^{13}**C-NMR of 1l**

\

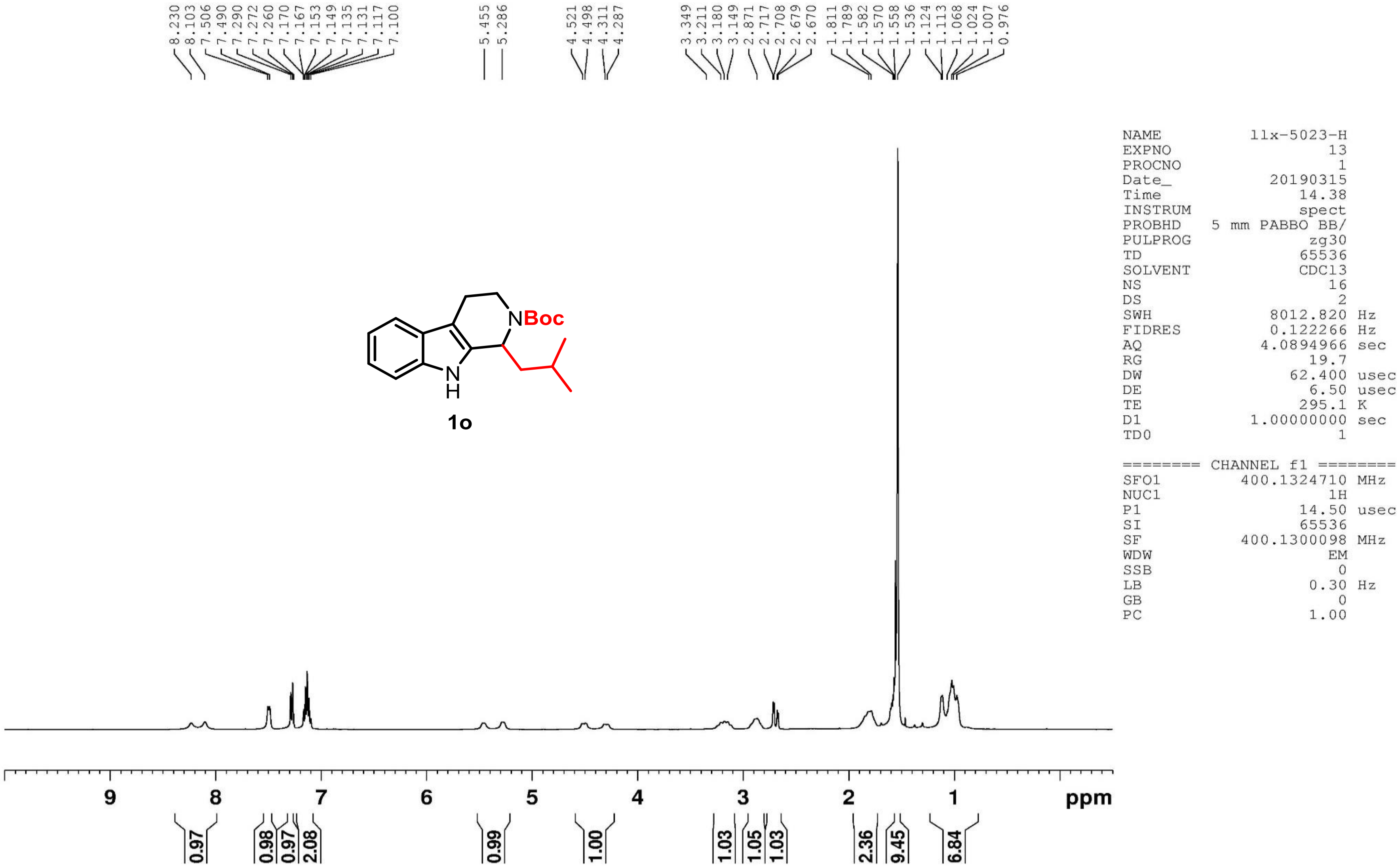

Supplementary Figure 12. ^{1}H-NMR of 1o

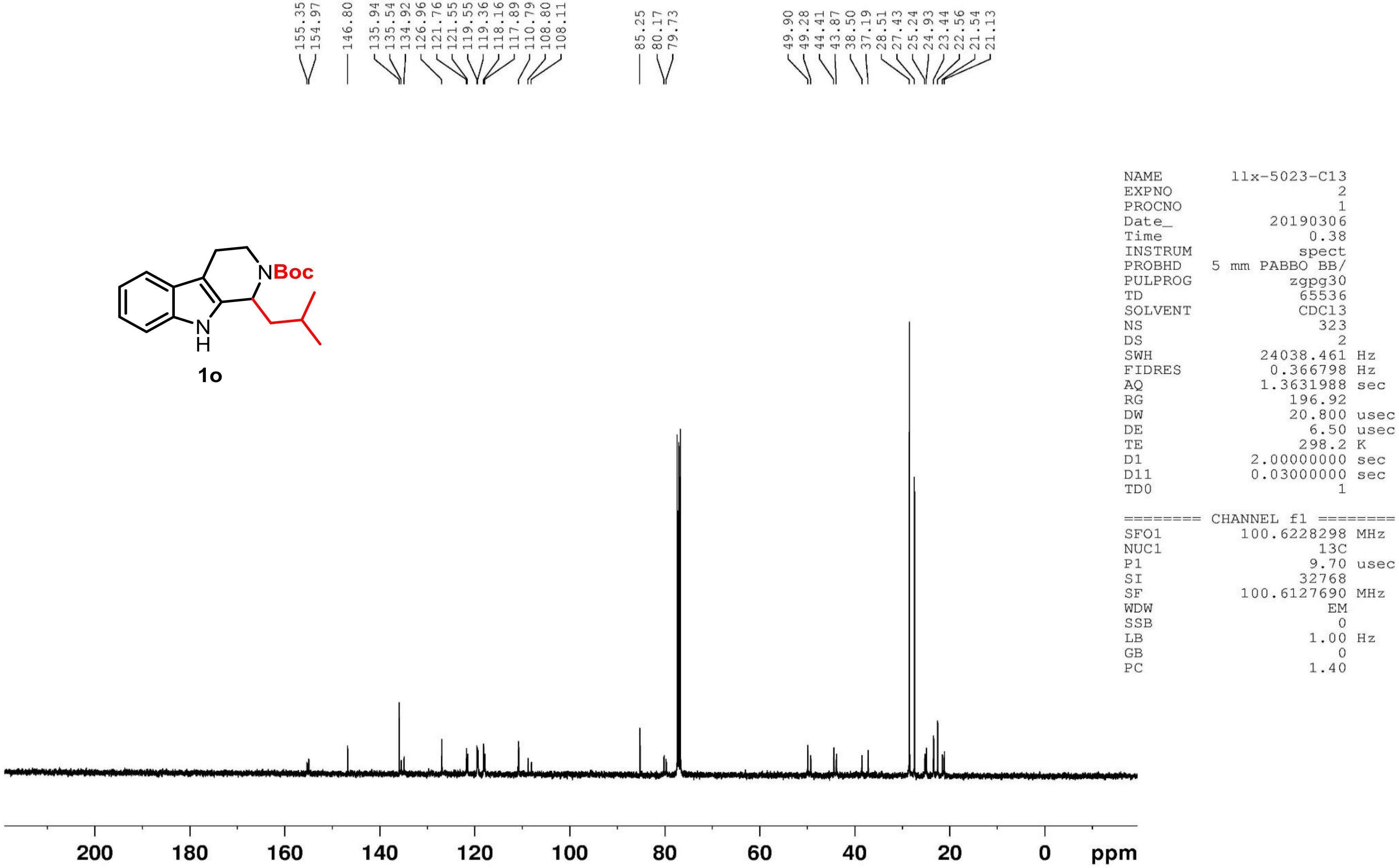

NAME	llx-5023-C13	
EXPNO	2	
PROCNO	1	
Date_	20190306	
Time	0.38	
INSTRUM	spect	
PROBHD	5 mm PABBO BB/	
PULPROG	zgpg30	
TD	65536	
SOLVENT	CDCl3	
NS	323	
DS	2	
SWH	24038.461	Hz
FIDRES	0.366798	Hz
AQ	1.3631988	sec
RG	196.92	
DW	20.800	usec
DE	6.50	usec
TE	298.2	K
D1	2.00000000	sec
D11	0.03000000	sec
TD0	1	
======== CHANNEL f1 ========		
SFO1	100.6228298	MHz
NUC1	13C	
P1	9.70	usec
SI	32768	
SF	100.6127690	MHz
WDW	EM	
SSB	0	
LB	1.00	Hz
GB	0	
PC	1.40	

Supplementary Figure 13. ^{13}C-NMR of 1o

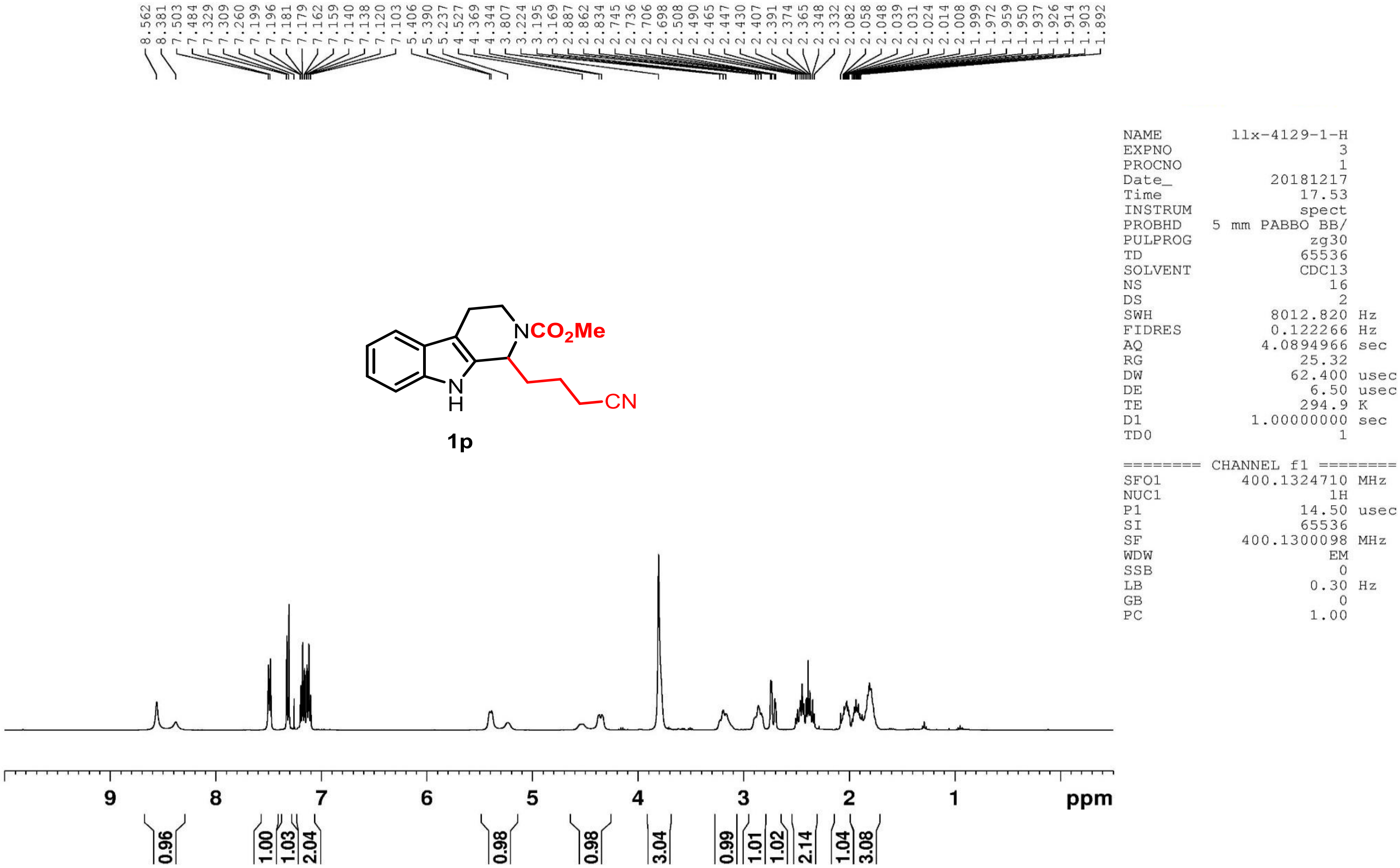

Supplementary Figure 14. ^{1}H-NMR of 1p

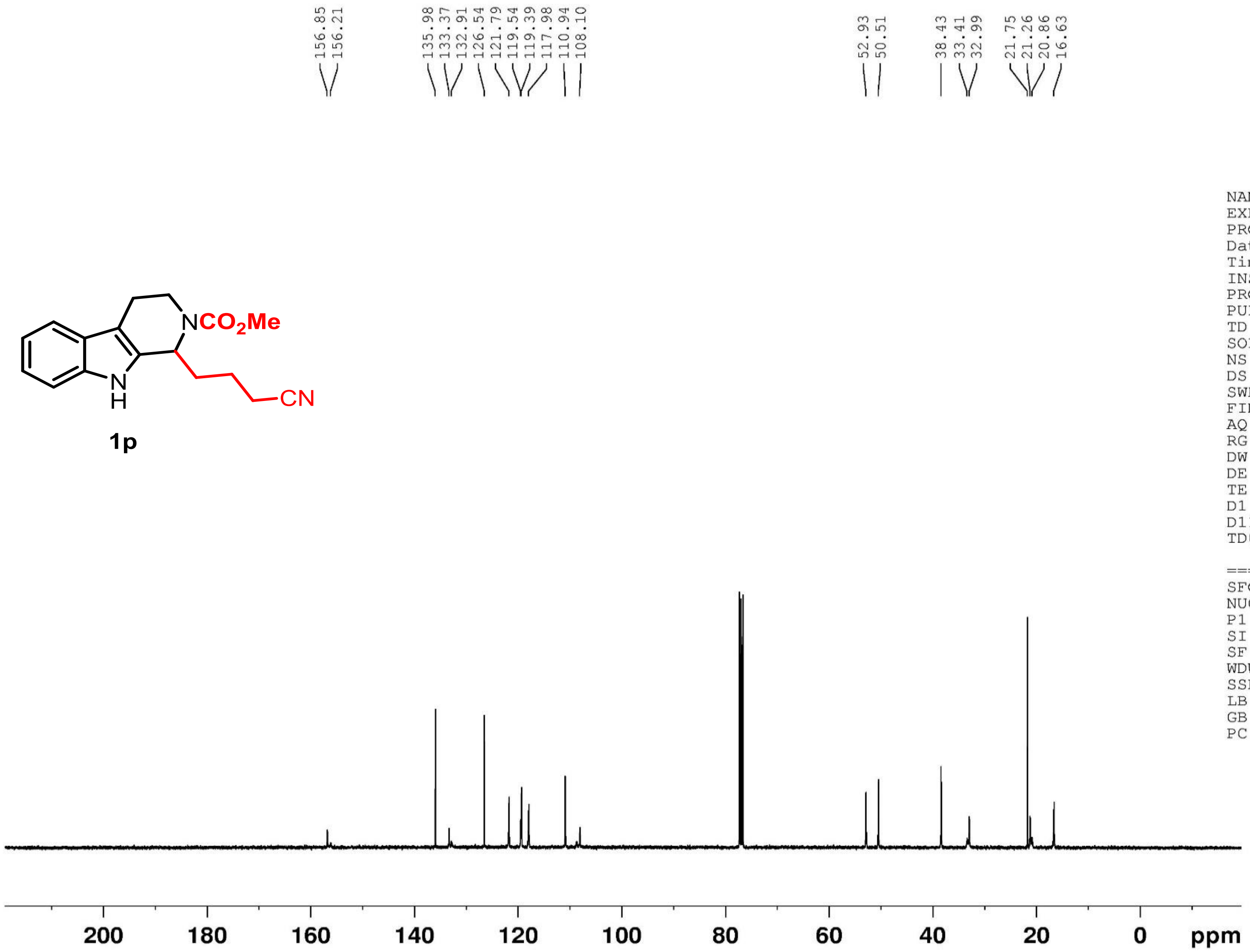

NAME	llx-4129-1-C13
EXPNO	2
PROCNO	1
Date_	20181217
Time	18.17
INSTRUM	spect
PROBHD	5 mm PABBO BB/
PULPROG	zgpg30
TD	65536
SOLVENT	CDCl3
NS	400
DS	2
SWH	24038.461 Hz
FIDRES	0.366798 Hz
AQ	1.3631988 sec
RG	196.92
DW	20.800 usec
DE	6.50 usec
TE	295.9 K
D1	2.00000000 sec
D11	0.03000000 sec
TD0	1

======== CHANNEL f1 ========

SFO1	100.6228298 MHz
NUC1	13C
P1	9.70 usec
SI	32768
SF	100.6127862 MHz
WDW	EM
SSB	0
LB	1.00 Hz
GB	0
PC	1.40

Supplementary Figure 15. ^{13}C-NMR of 1p

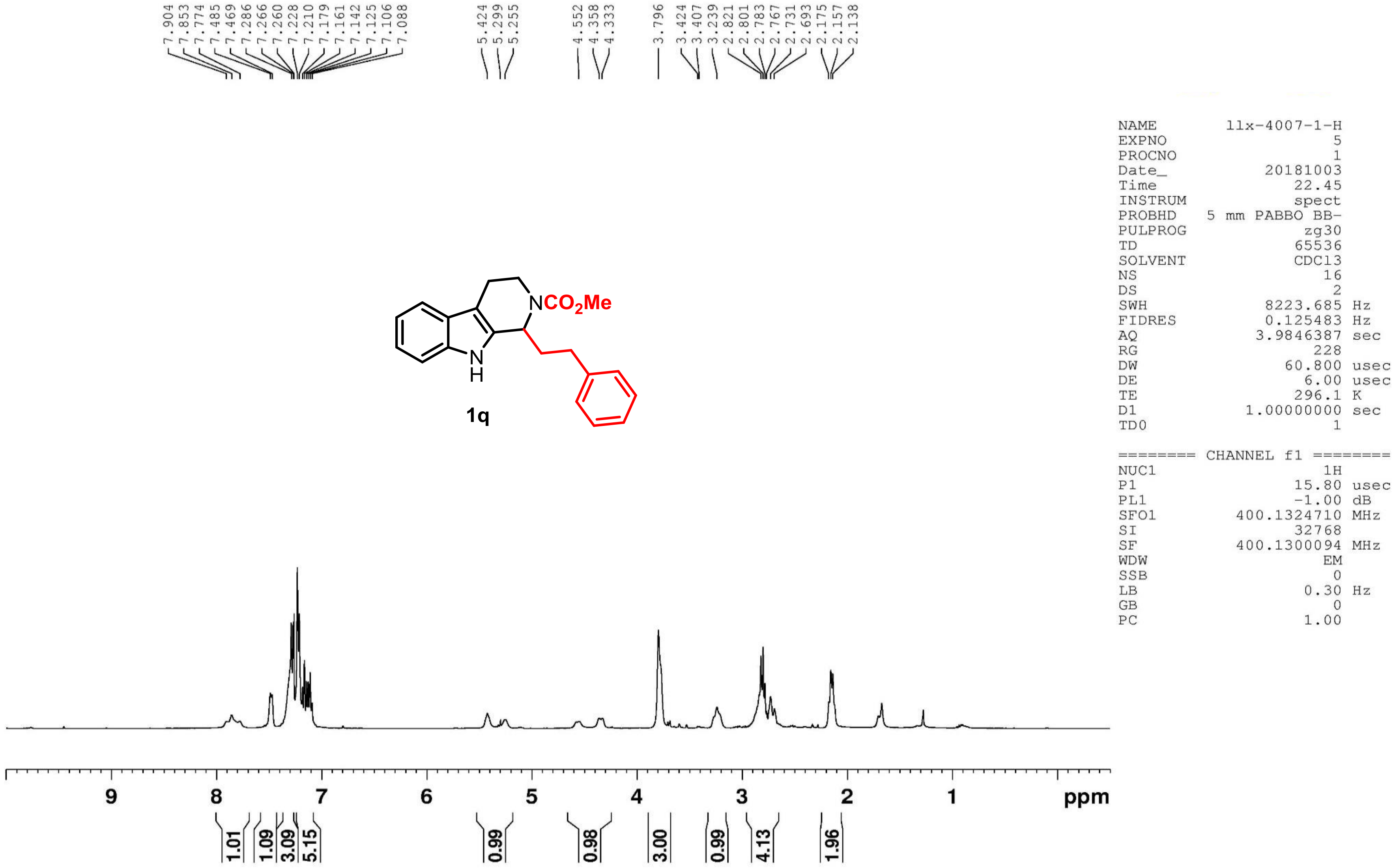

Supplementary Figure 16. ^{1}H-NMR of 1q

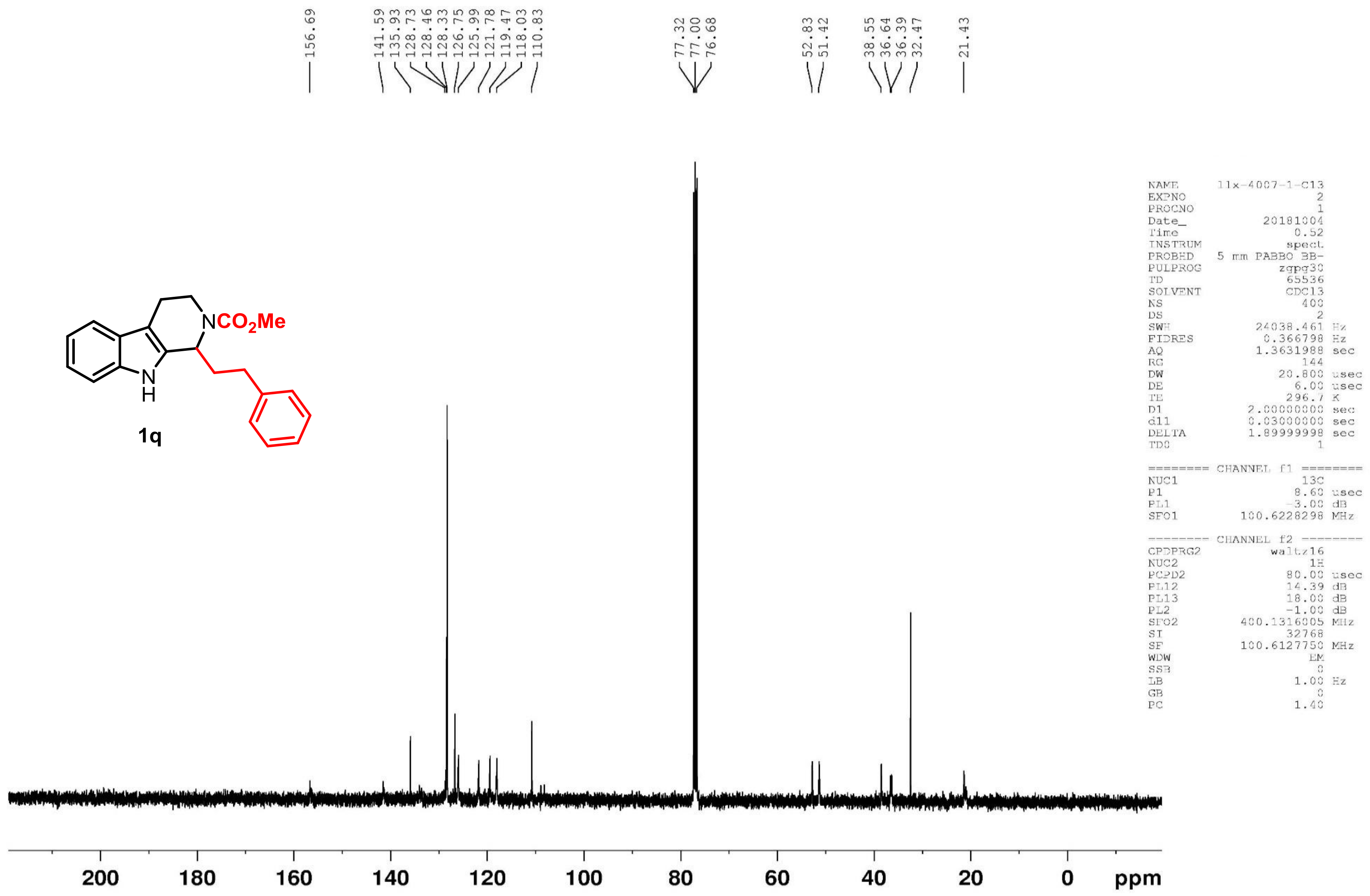

Supplementary Figure 17. ^{13}C**-NMR of 1q**

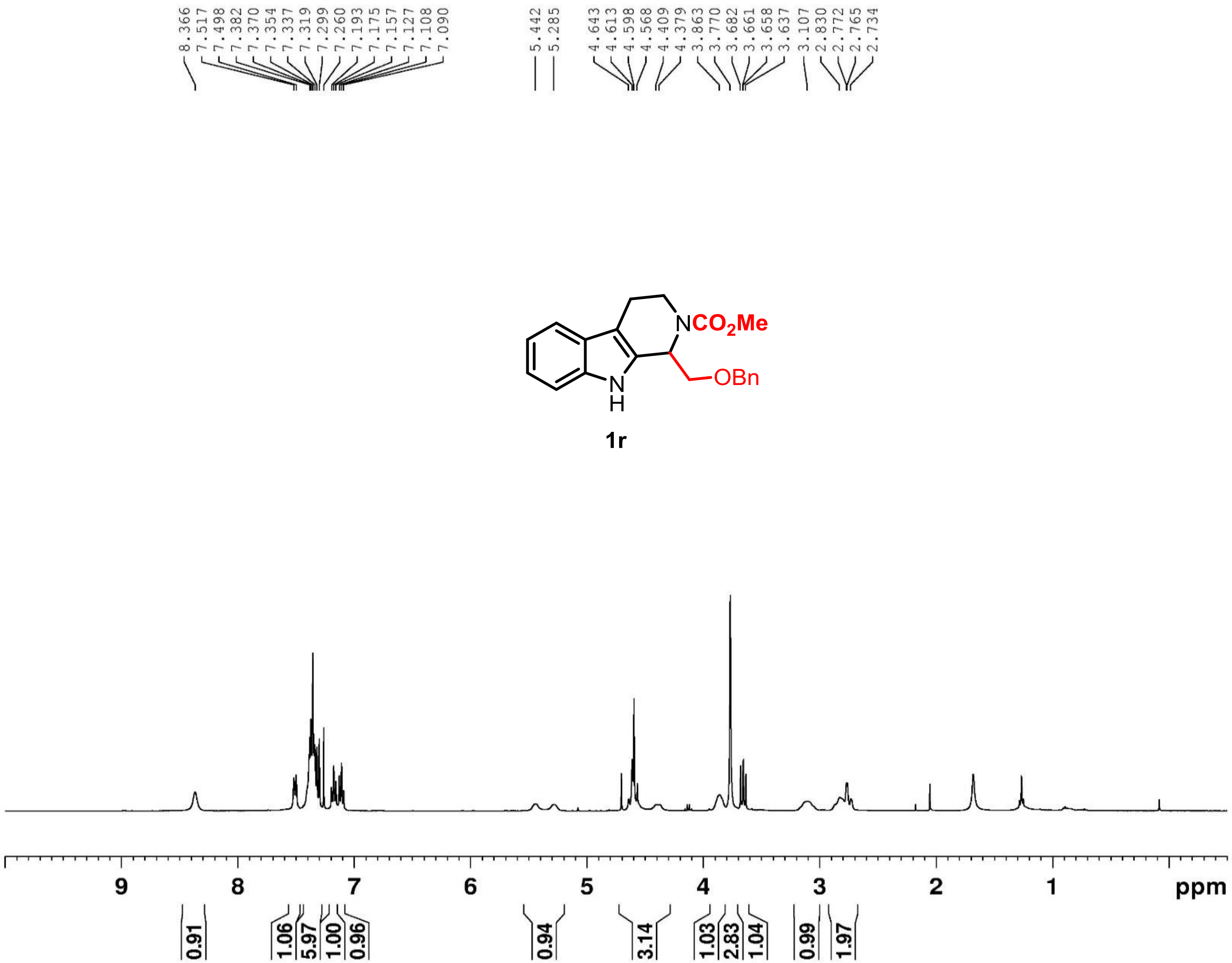

1r

NAME	llx-4025-2-2-H-NMR
EXPNO	1
PROCNO	1
Date_	20190412
Time	17.39
INSTRUM	spect
PROBHD	5 mm PABBO BB-
PULPROG	zg30
TD	65536
SOLVENT	CDCl3
NS	16
DS	2
SWH	8223.685 Hz
FIDRES	0.125483 Hz
AQ	3.9846387 sec
RG	287
DW	60.800 usec
DE	6.00 usec
TE	294.4 K
D1	1.00000000 sec
TD0	1
======== CHANNEL f1 ========	
NUC1	1H
P1	15.80 usec
PL1	-1.00 dB
PL1W	12.17476940 W
SFO1	400.1324710 MHz
SI	32768
SF	400.1300091 MHz
WDW	EM
SSB	0
LB	0.30 Hz
GB	0
PC	1.00

Supplementary Figure 18. ^{1}H-NMR of 1r

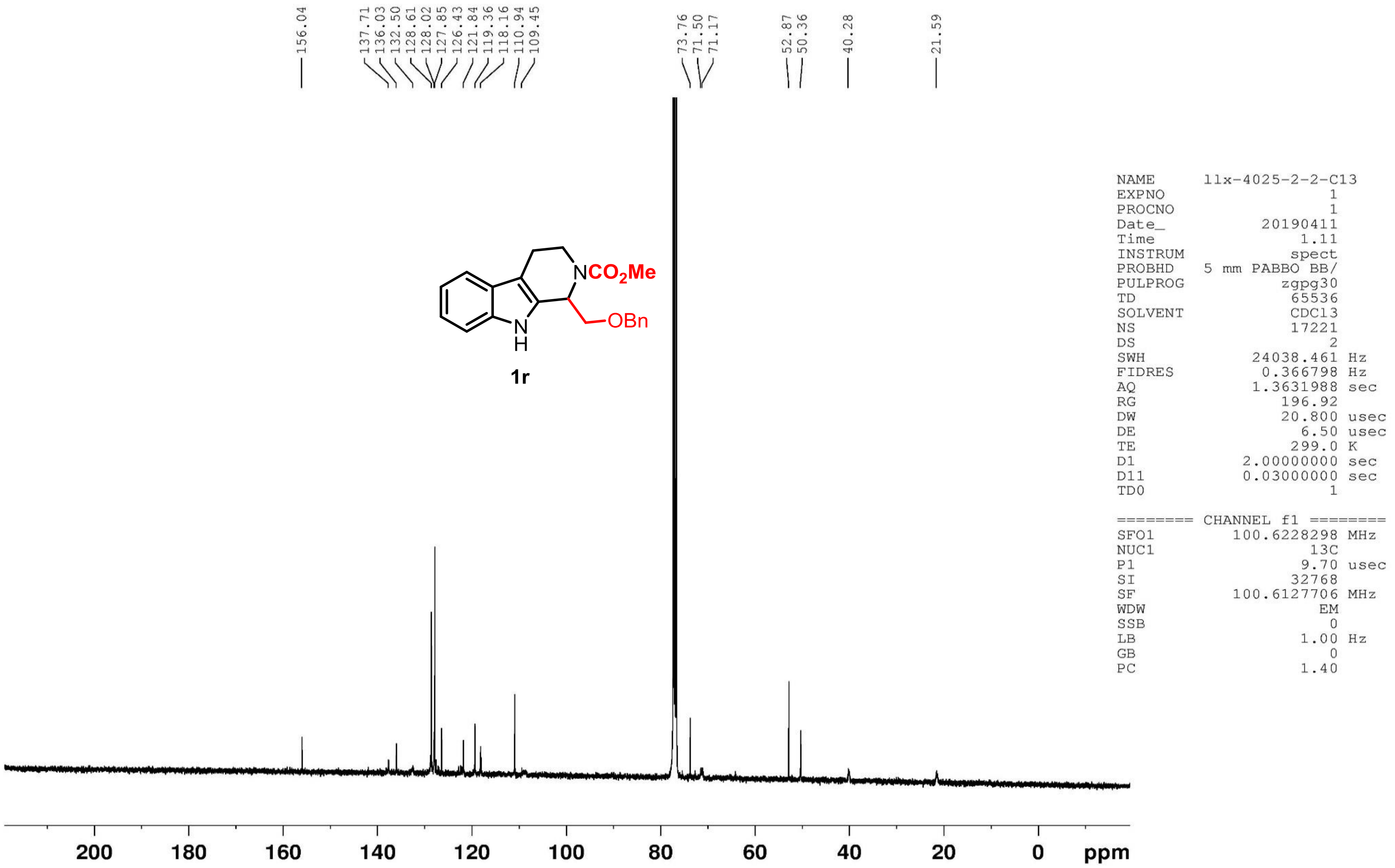

Supplementary Figure 19. ^{13}C-NMR of 1r

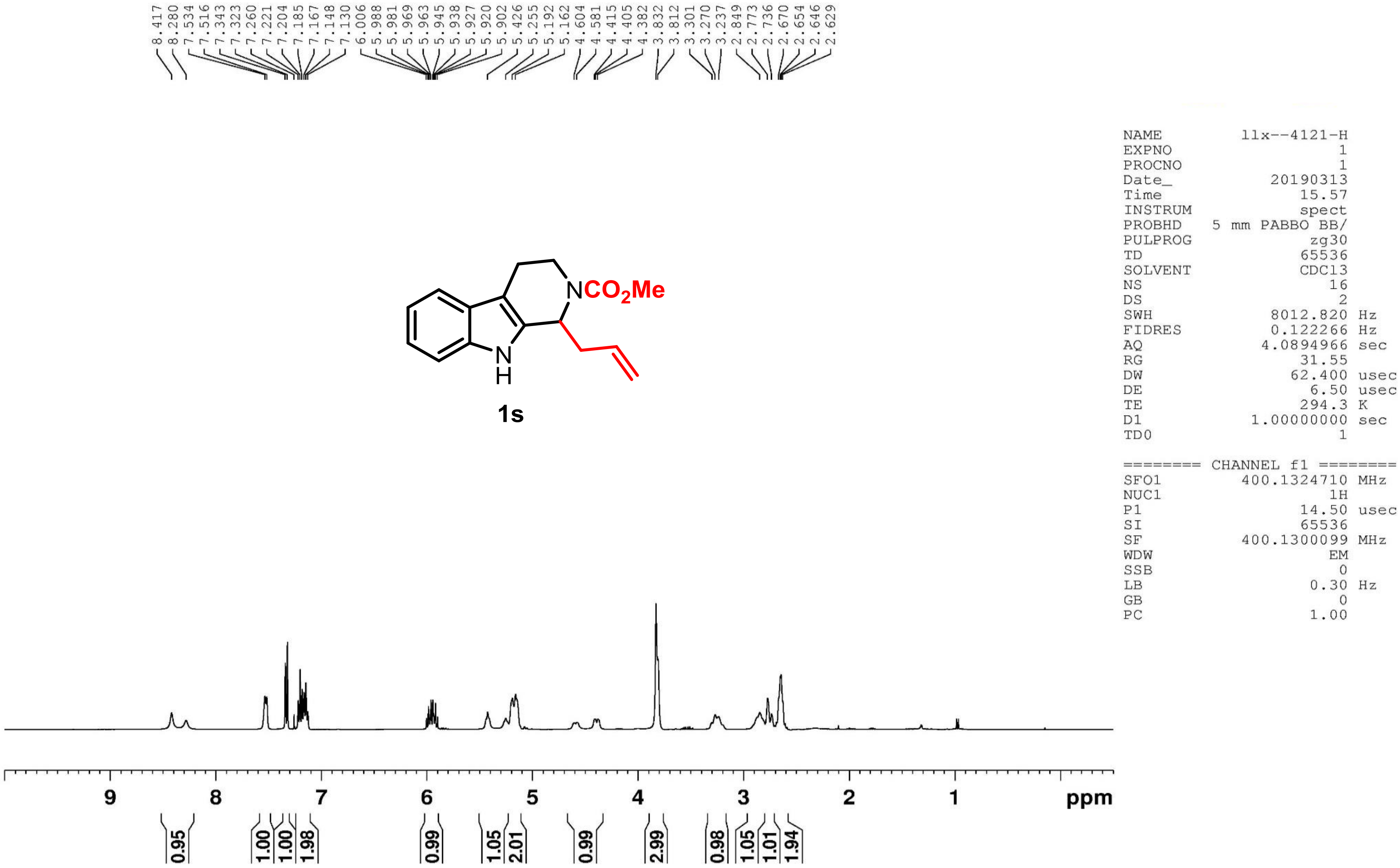

Supplementary Figure 20. ^{1}H-NMR of 1s

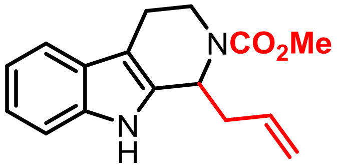

1s

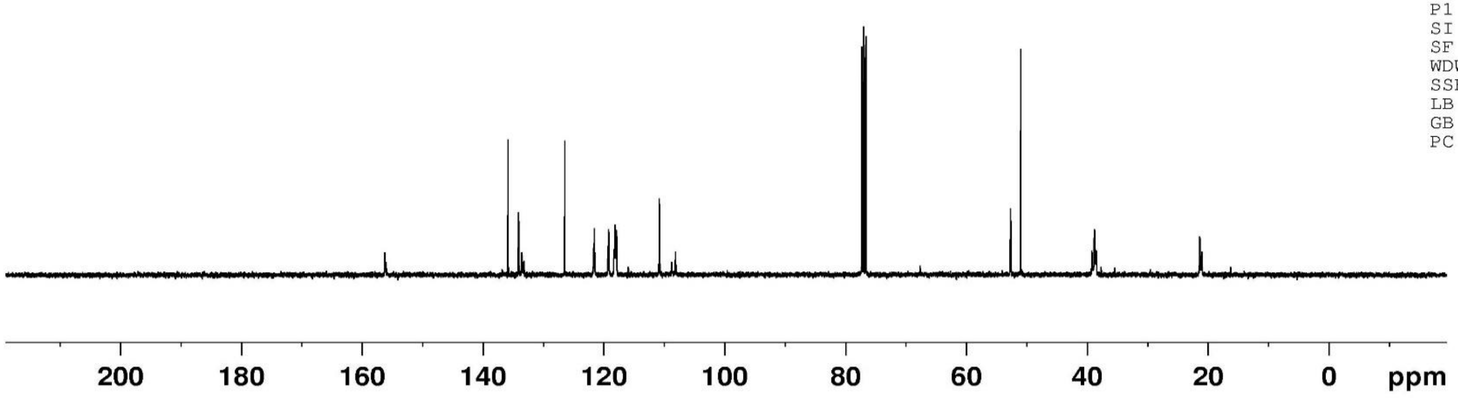

Supplementary Figure 21. ^{13}**C-NMR of 1S**

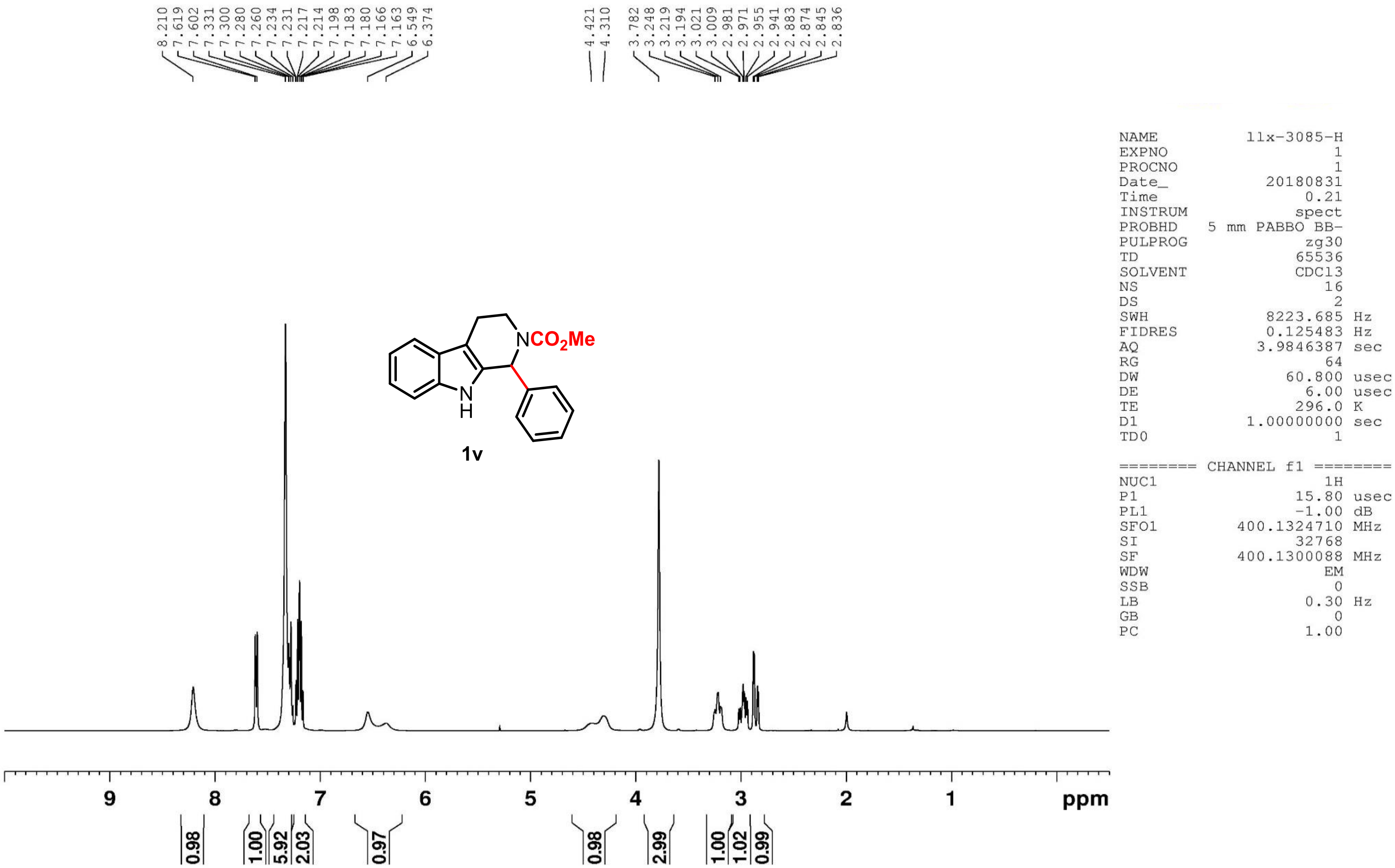

Supplementary Figure 22. ^{1}H-NMR of 1v

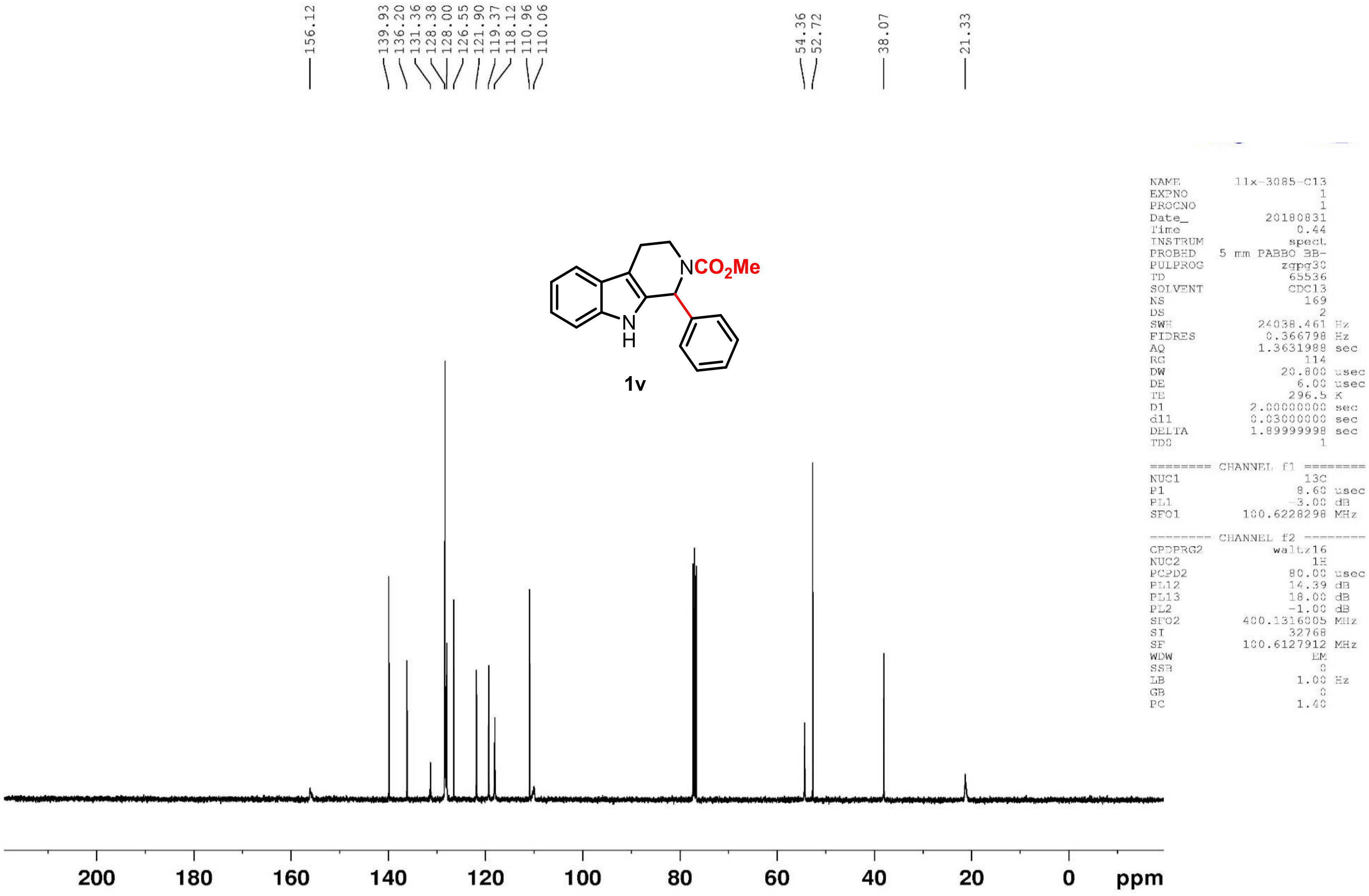

Supplementary Figure 23. ^{13}**C-NMR of 1v**

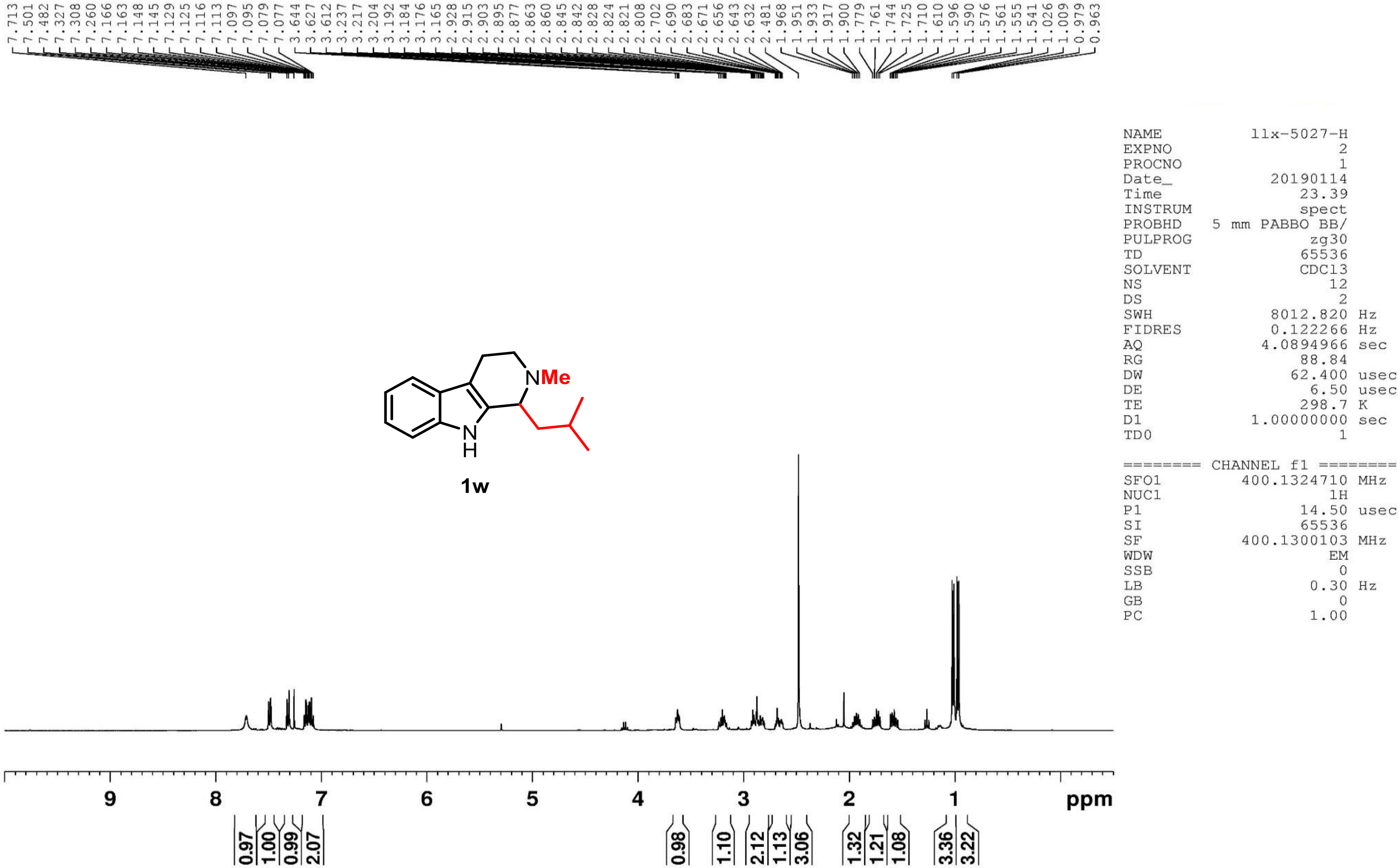

Supplementary Figure 24. ^{1}H-NMR of 1w

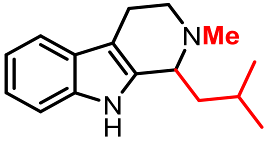

1w

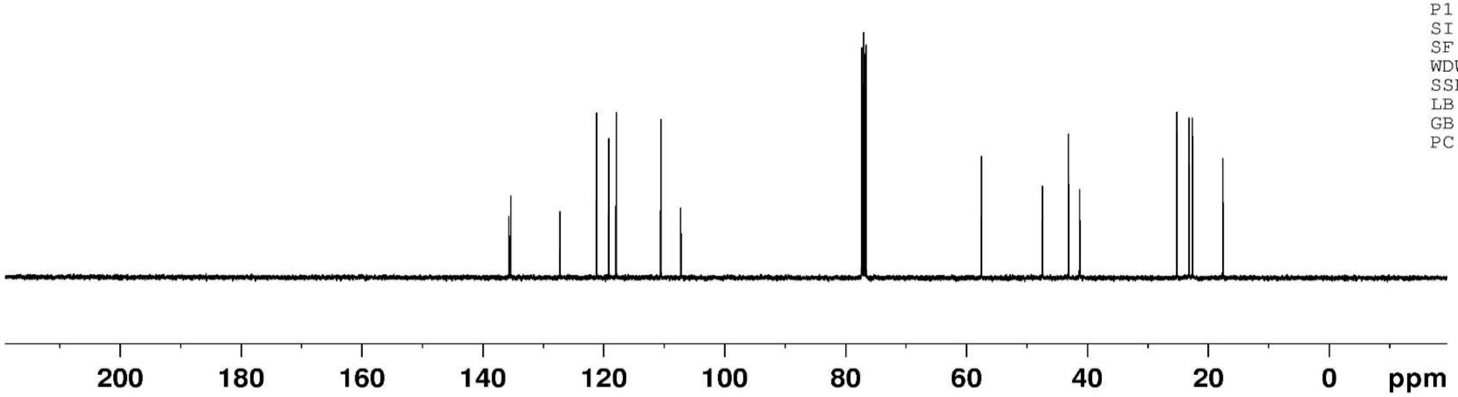


```
NAME          llx-5027-C13
EXPNO                    1
PROCNO                   1
Date_             20190305
Time                 11.06
INSTRUM              spect
PROBHD     5 mm PABBO BB/
PULPROG            zgpg30
TD                  65536
SOLVENT             CDCl3
NS                    141
DS                      2
SWH             24038.461 Hz
FIDRES           0.366798 Hz
AQ              1.3631988 sec
RG                 196.92
DW                 20.800 usec
DE                   6.50 usec
TE                  297.3 K
D1             2.00000000 sec
D11            0.03000000 sec
TD0                     1

======== CHANNEL f1 ========
SFO1         100.6228298 MHz
NUC1                 13C
P1                  9.70 usec
SI                 32768
SF           100.6127802 MHz
WDW                   EM
SSB                    0
LB                  1.00 Hz
GB                     0
PC                  1.40
```

Supplementary Figure 25. ^{13}C-NMR of 1w

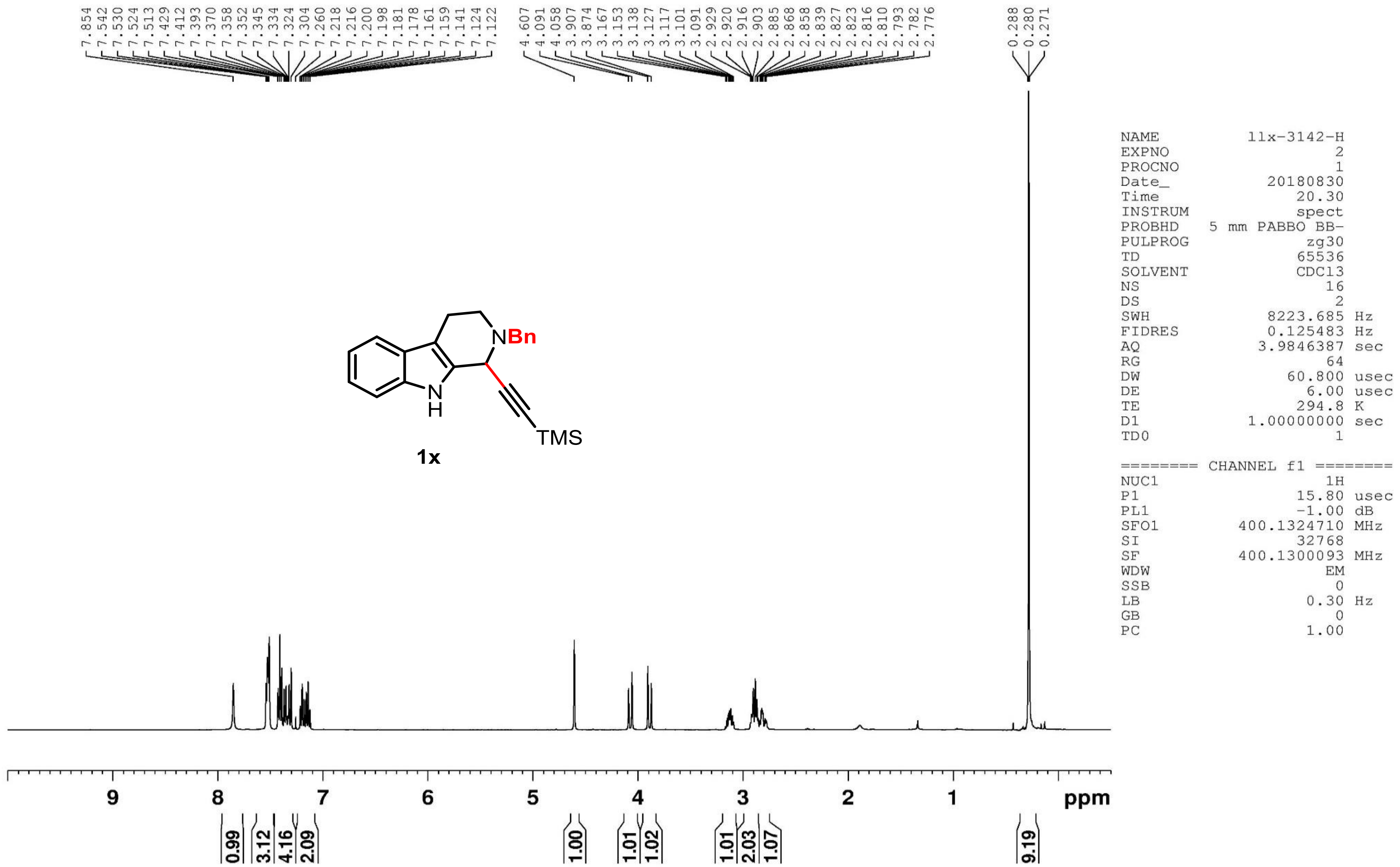

Supplementary Figure 26. ^{1}H-NMR of 1x

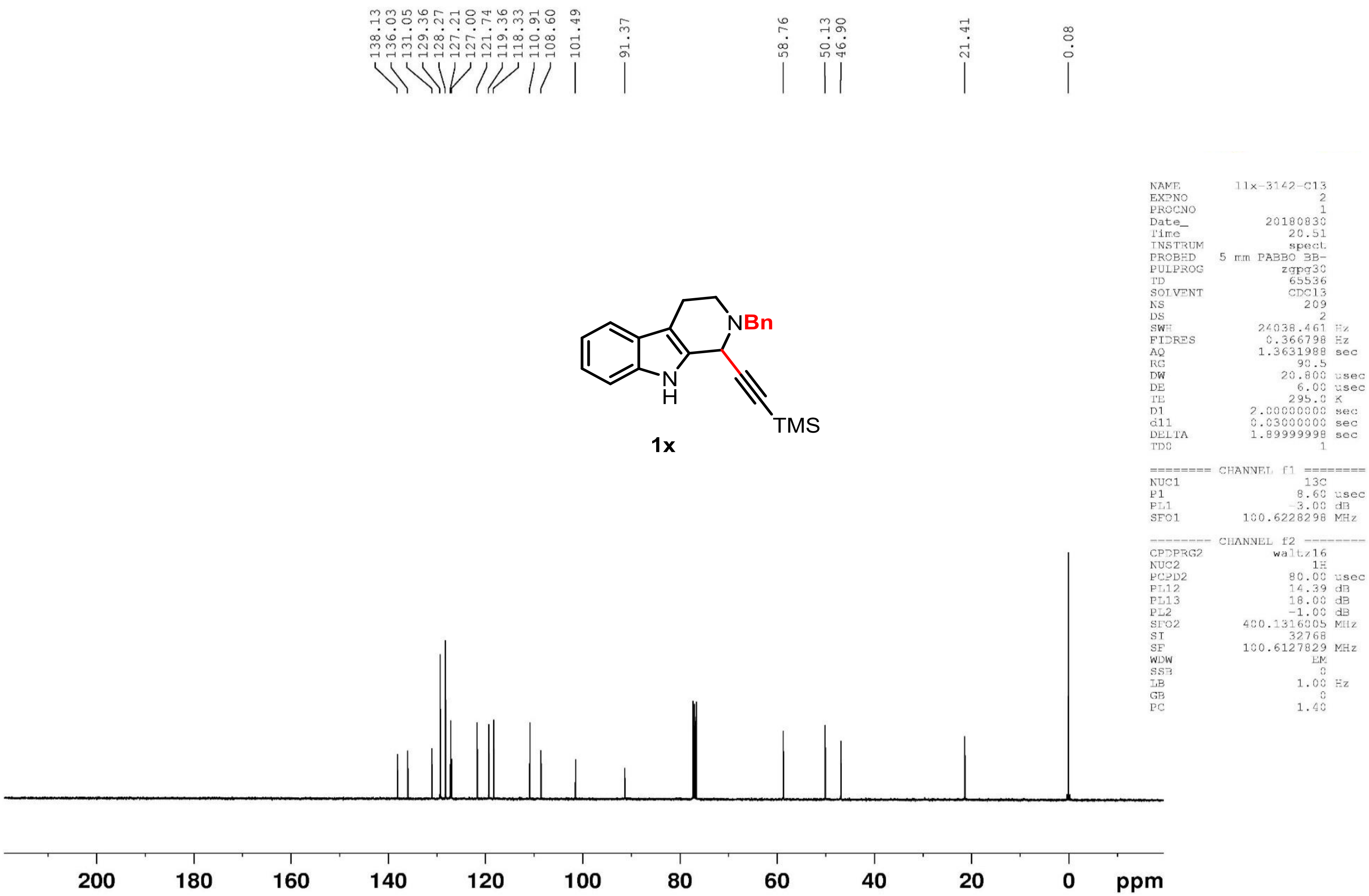

Supplementary Figure 27. ^{13}C-NMR of 1x

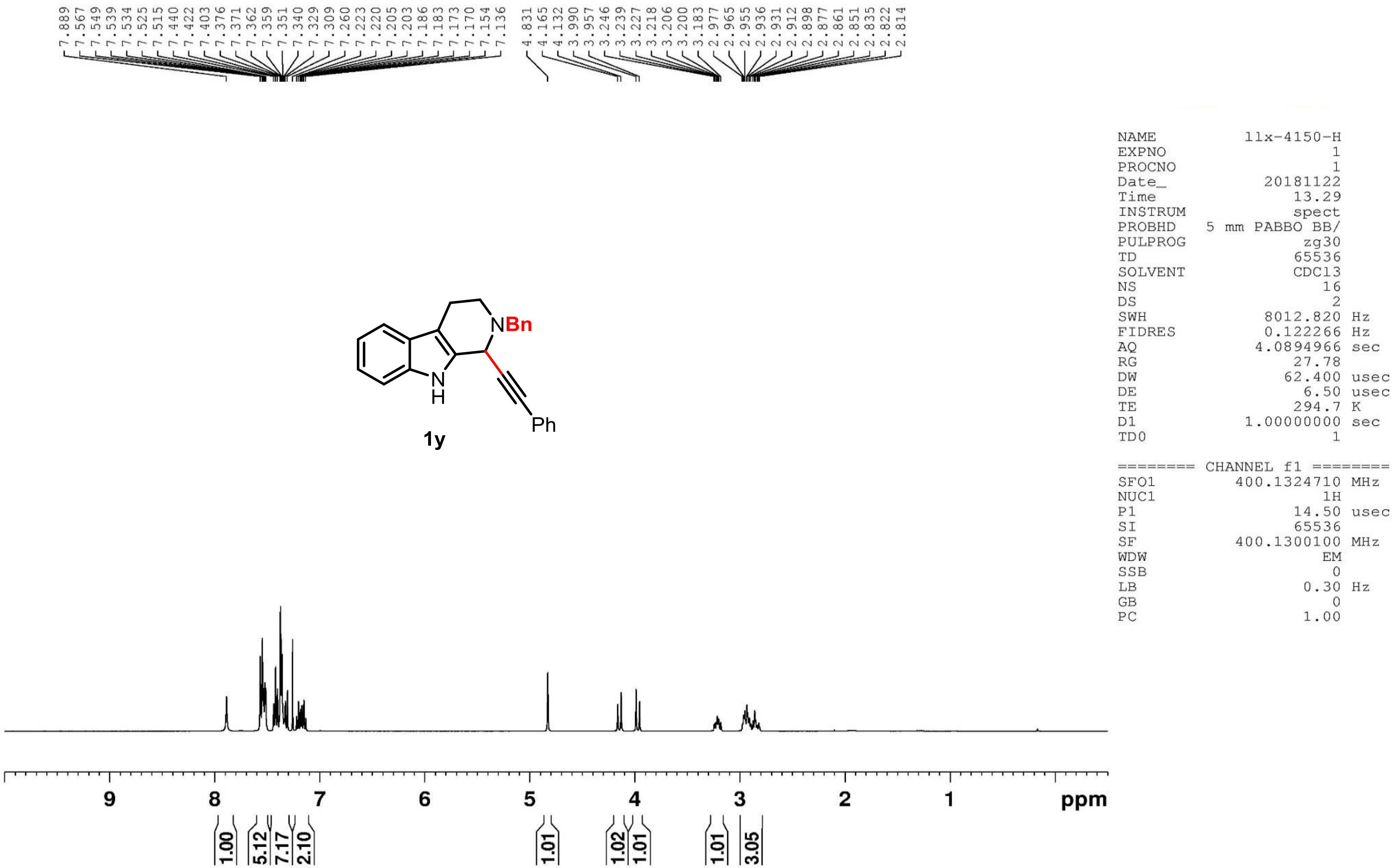

Supplementary Figure 28. ^{1}**H-NMR of 1y**

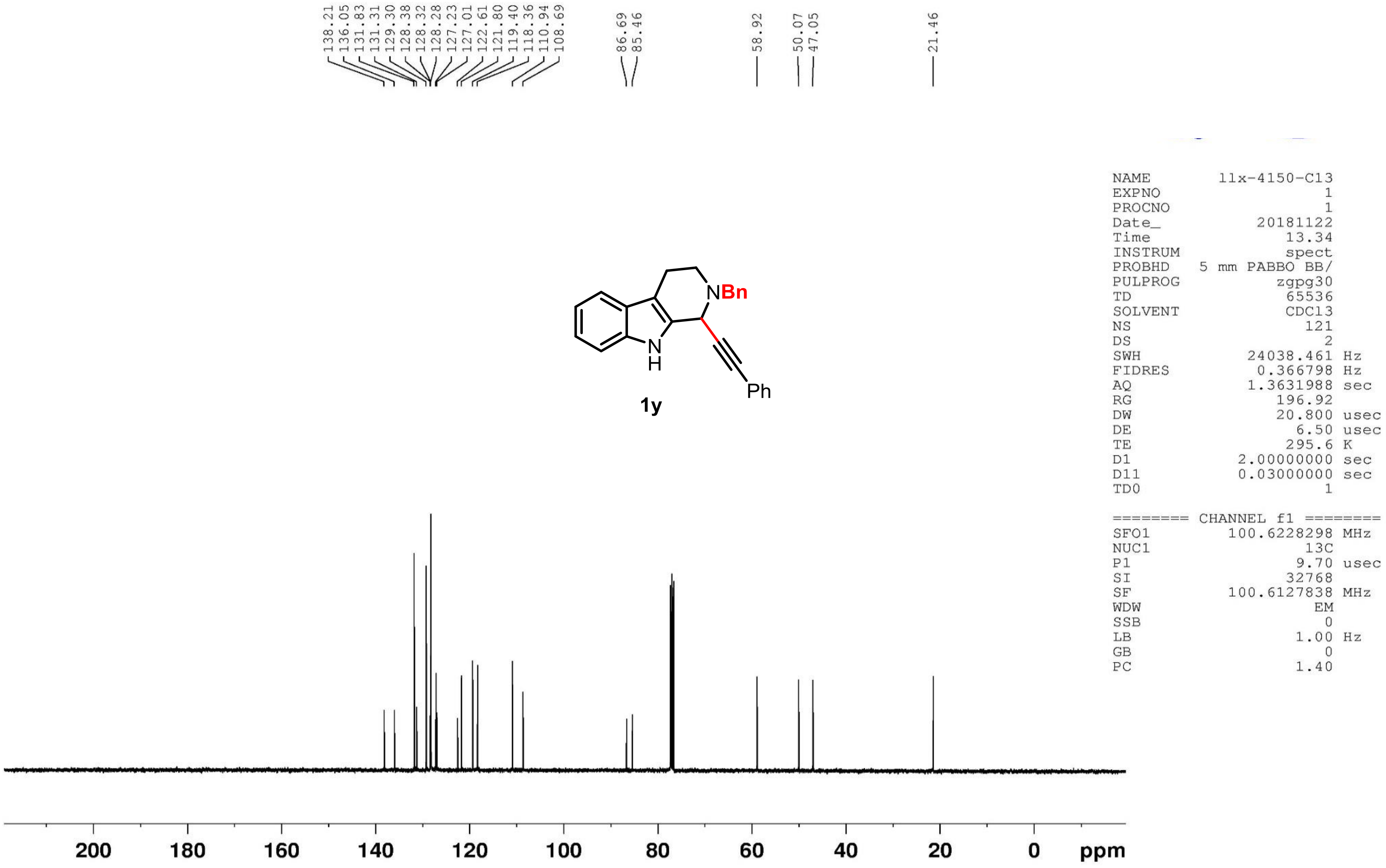

Supplementary Figure 29. ^{13}C-NMR of 1y

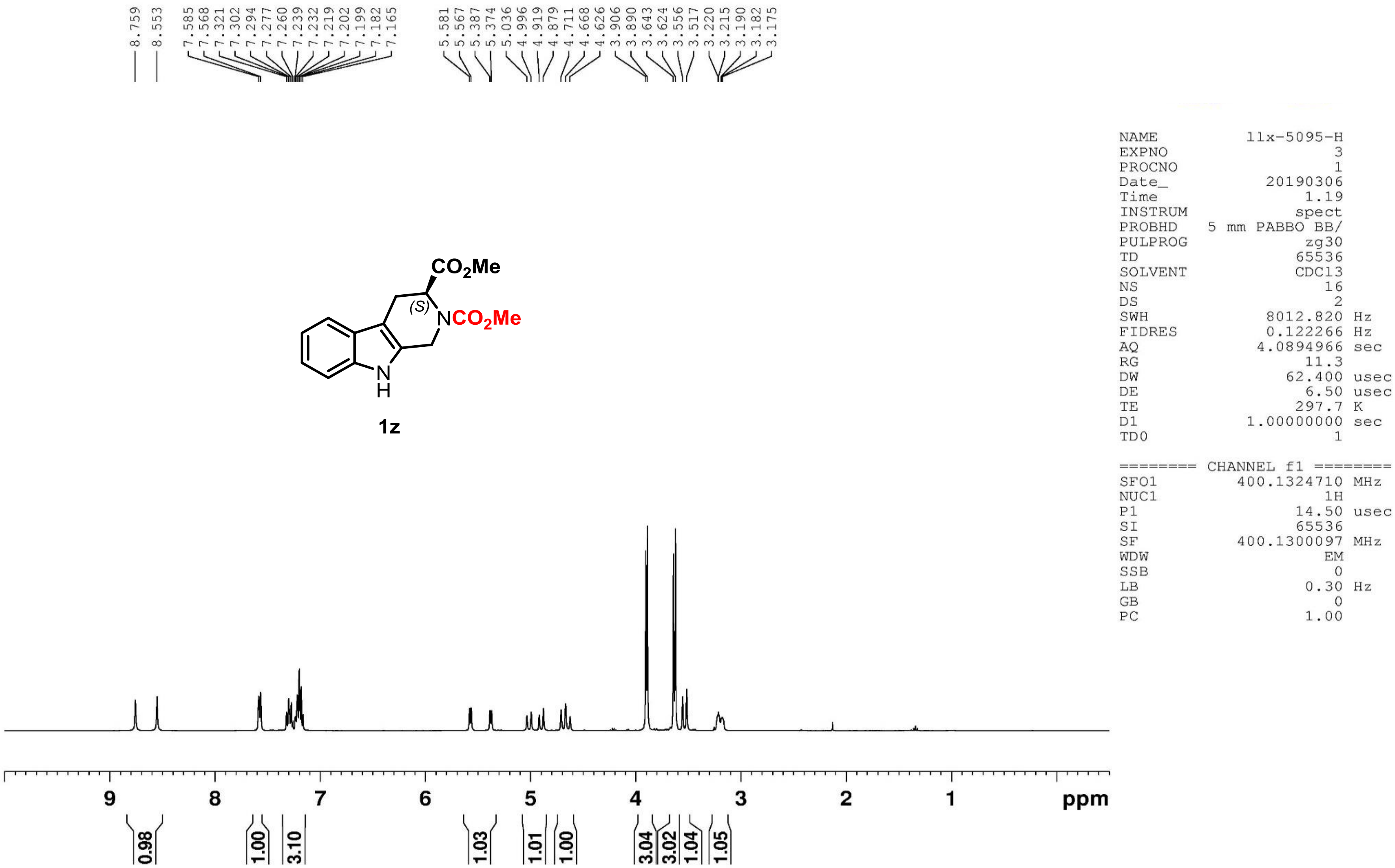

Supplementary Figure 30. ^{1}H-NMR of 1z

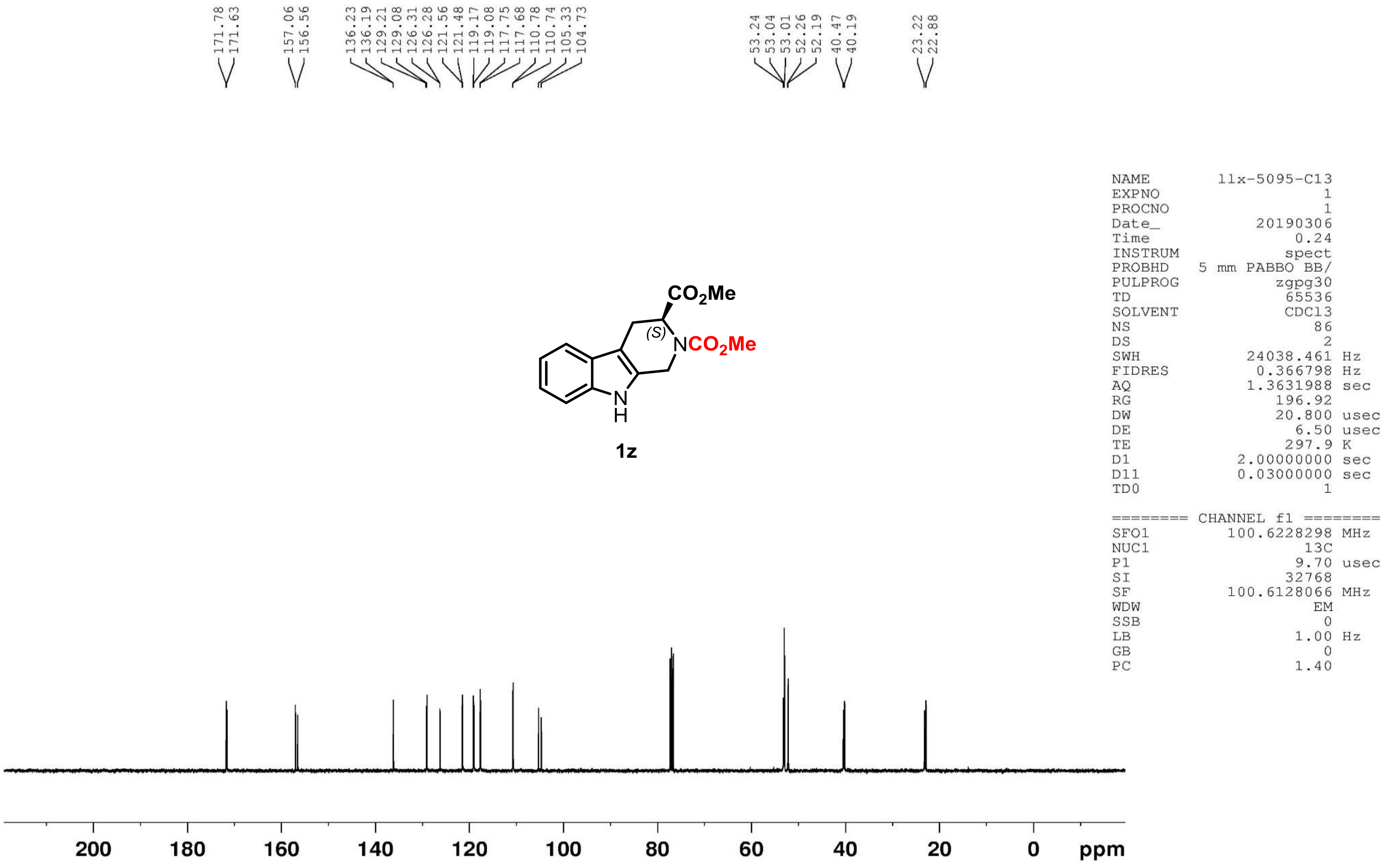

NAME	llx-5095-C13
EXPNO	1
PROCNO	1
Date_	20190306
Time	0.24
INSTRUM	spect
PROBHD	5 mm PABBO BB/
PULPROG	zgpg30
TD	65536
SOLVENT	CDCl3
NS	86
DS	2
SWH	24038.461 Hz
FIDRES	0.366798 Hz
AQ	1.3631988 sec
RG	196.92
DW	20.800 usec
DE	6.50 usec
TE	297.9 K
D1	2.00000000 sec
D11	0.03000000 sec
TD0	1

======== CHANNEL f1 ========

SFO1	100.6228298 MHz
NUC1	13C
P1	9.70 usec
SI	32768
SF	100.6128066 MHz
WDW	EM
SSB	0
LB	1.00 Hz
GB	0
PC	1.40

Supplementary Figure 31. ^{13}C-NMR of 1z

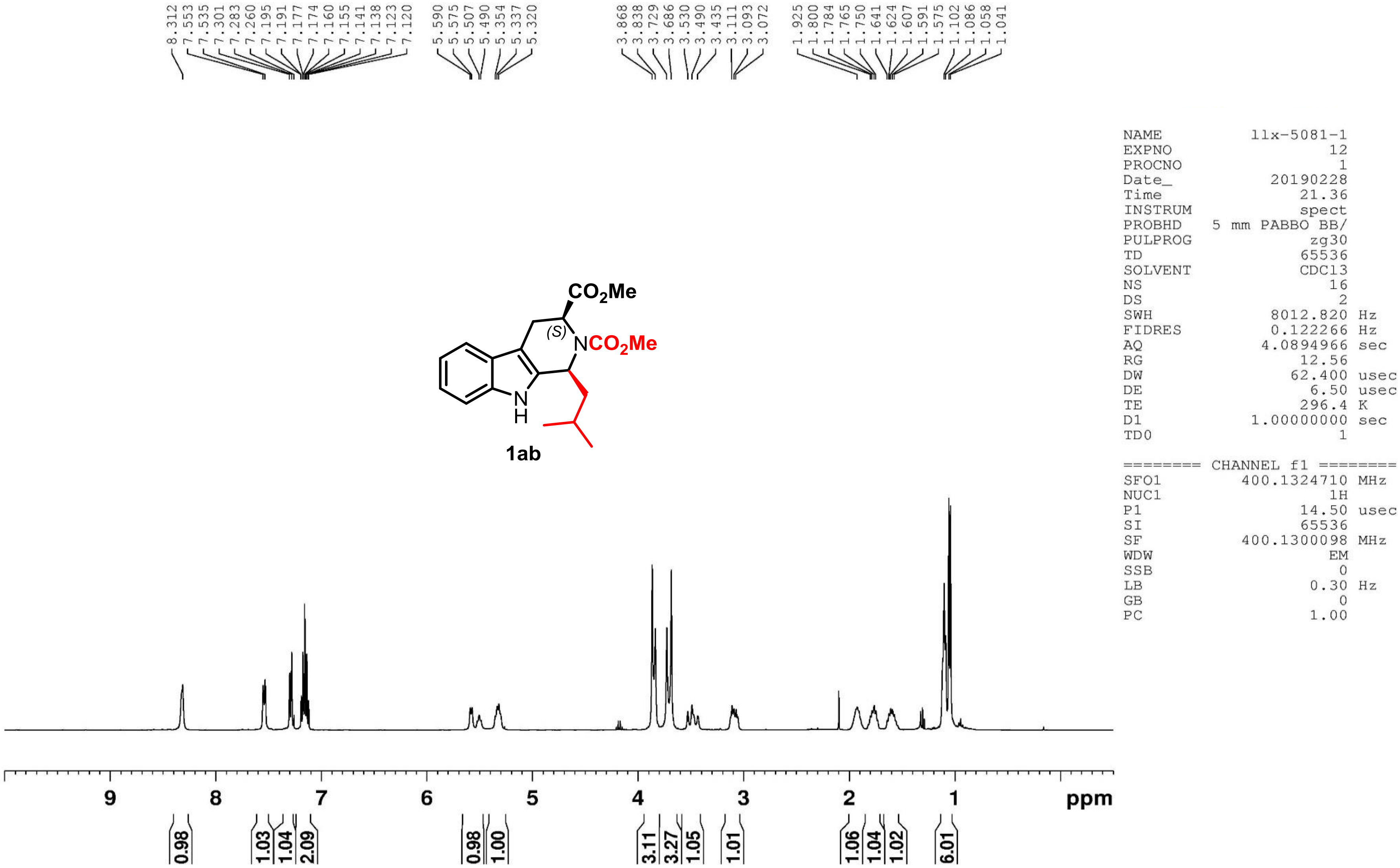

Supplementary Figure 32. ^{1}H-NMR of 1ab

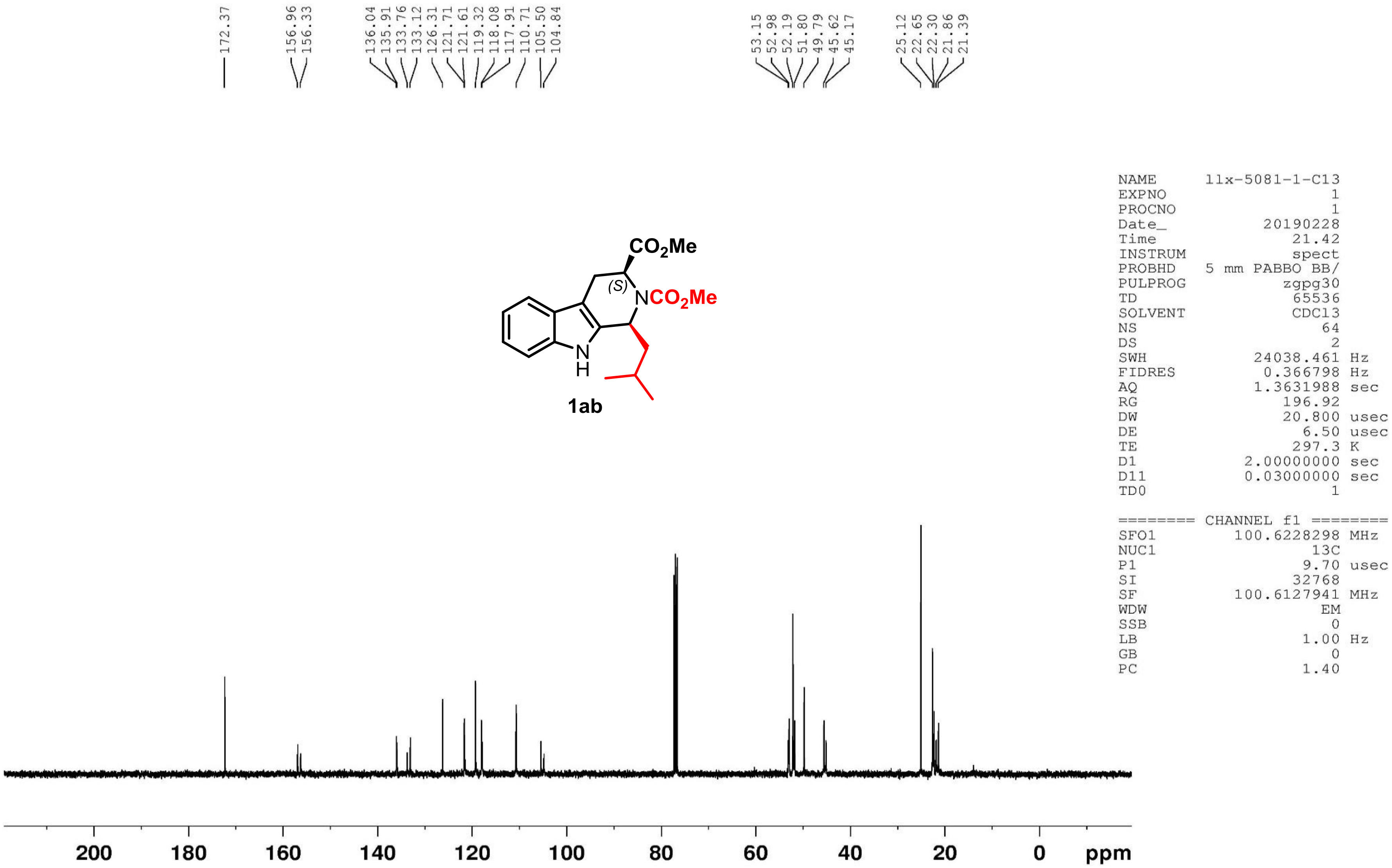

Supplementary Figure 33. ^{13}C-NMR of 1ab

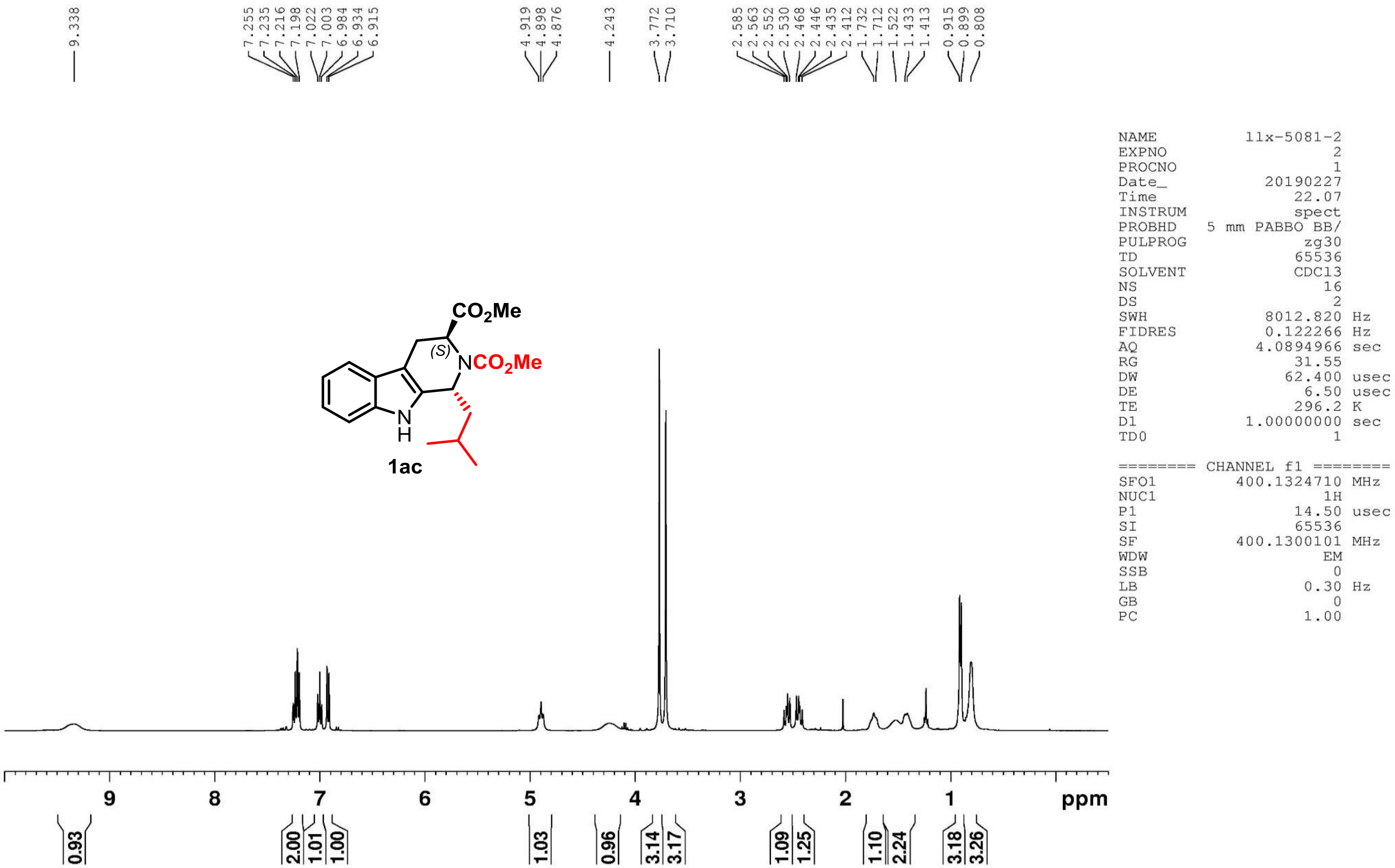

Supplementary Figure 34. ^{1}**H-NMR of 1ac**

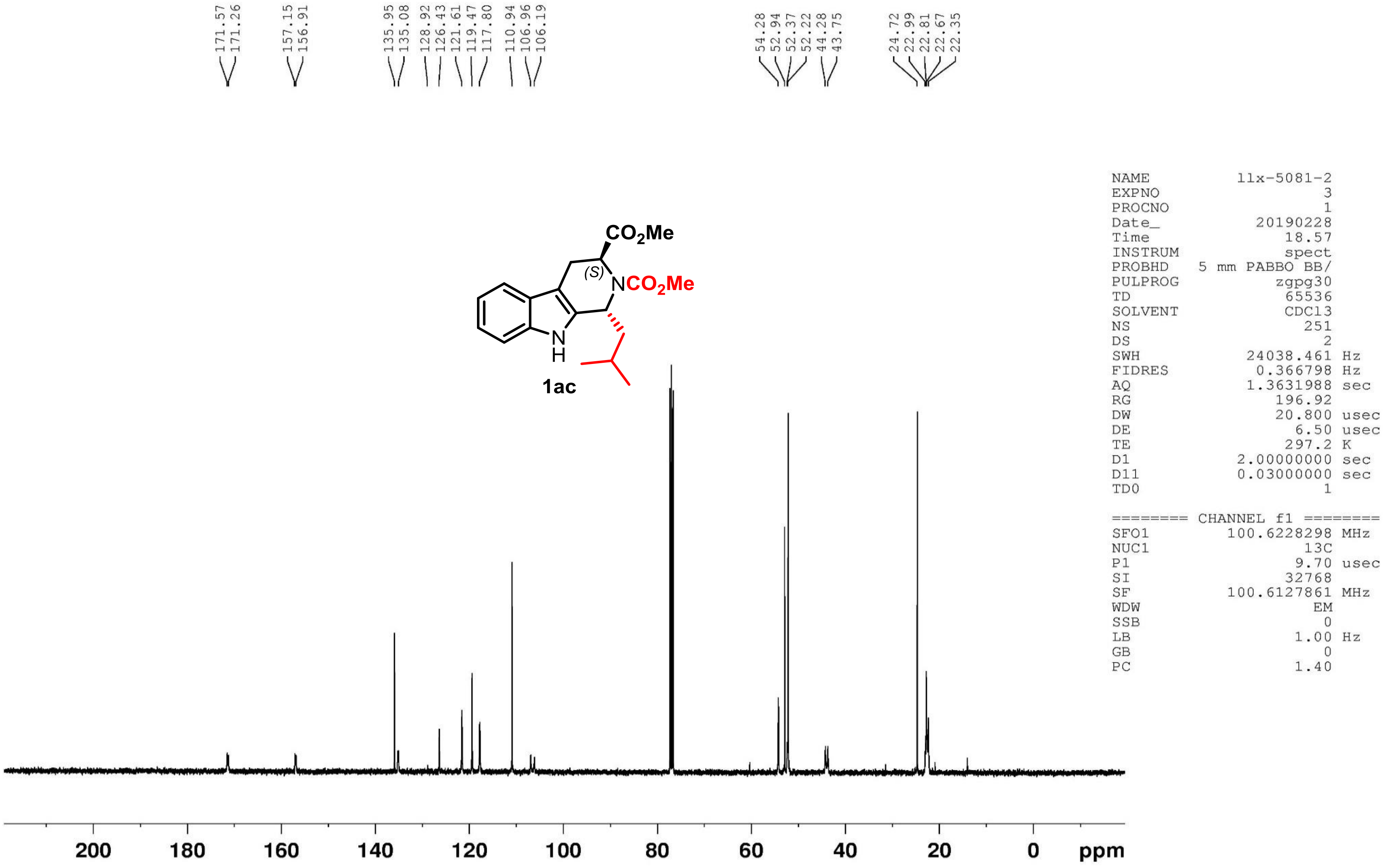

Supplementary Figure 35. ^{13}C-NMR of 1ac

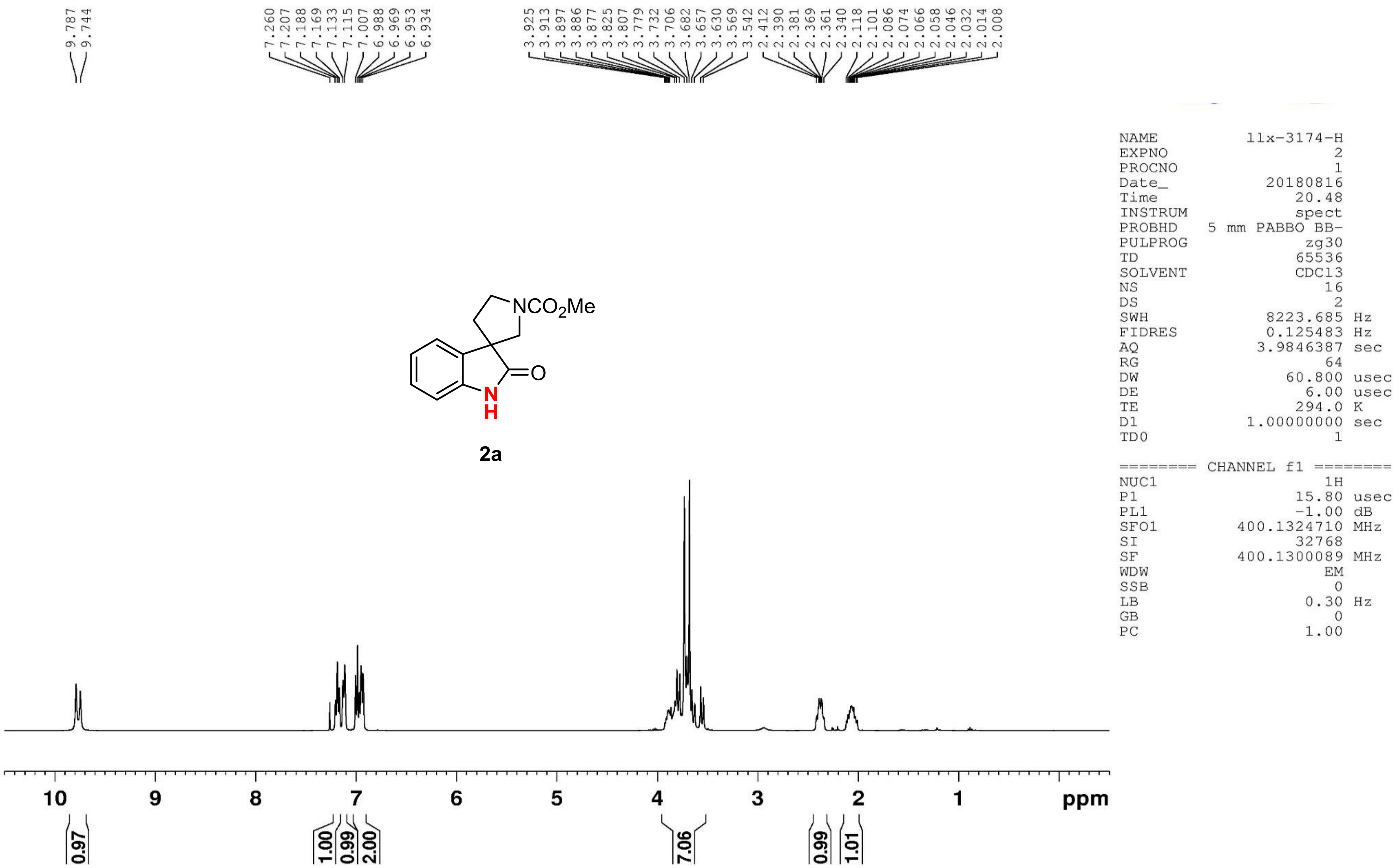

Supplementary Figure 36. ^{1}**H-NMR of 2a**

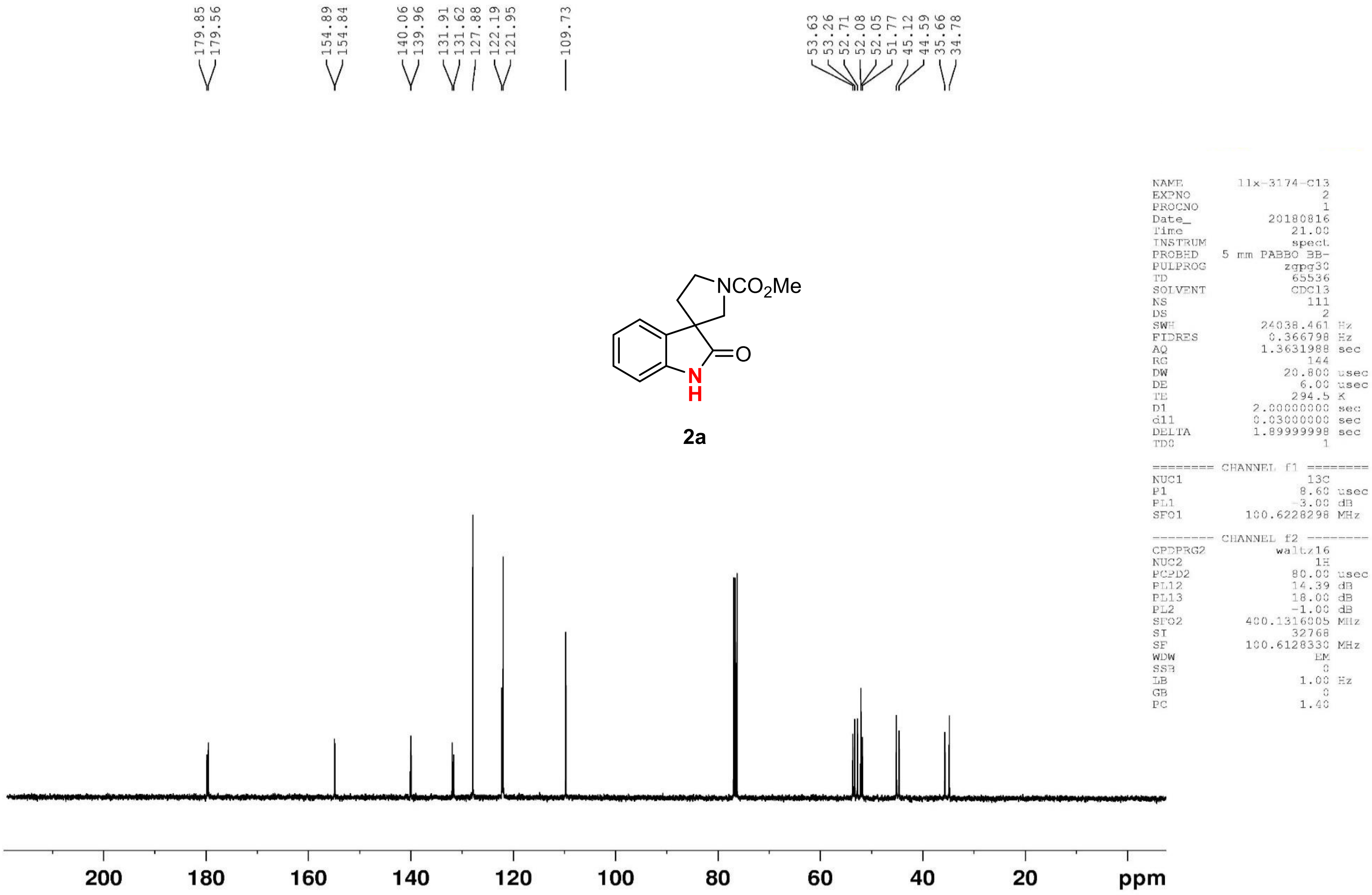

Supplementary Figure 37. ^{13}C-NMR of 2a

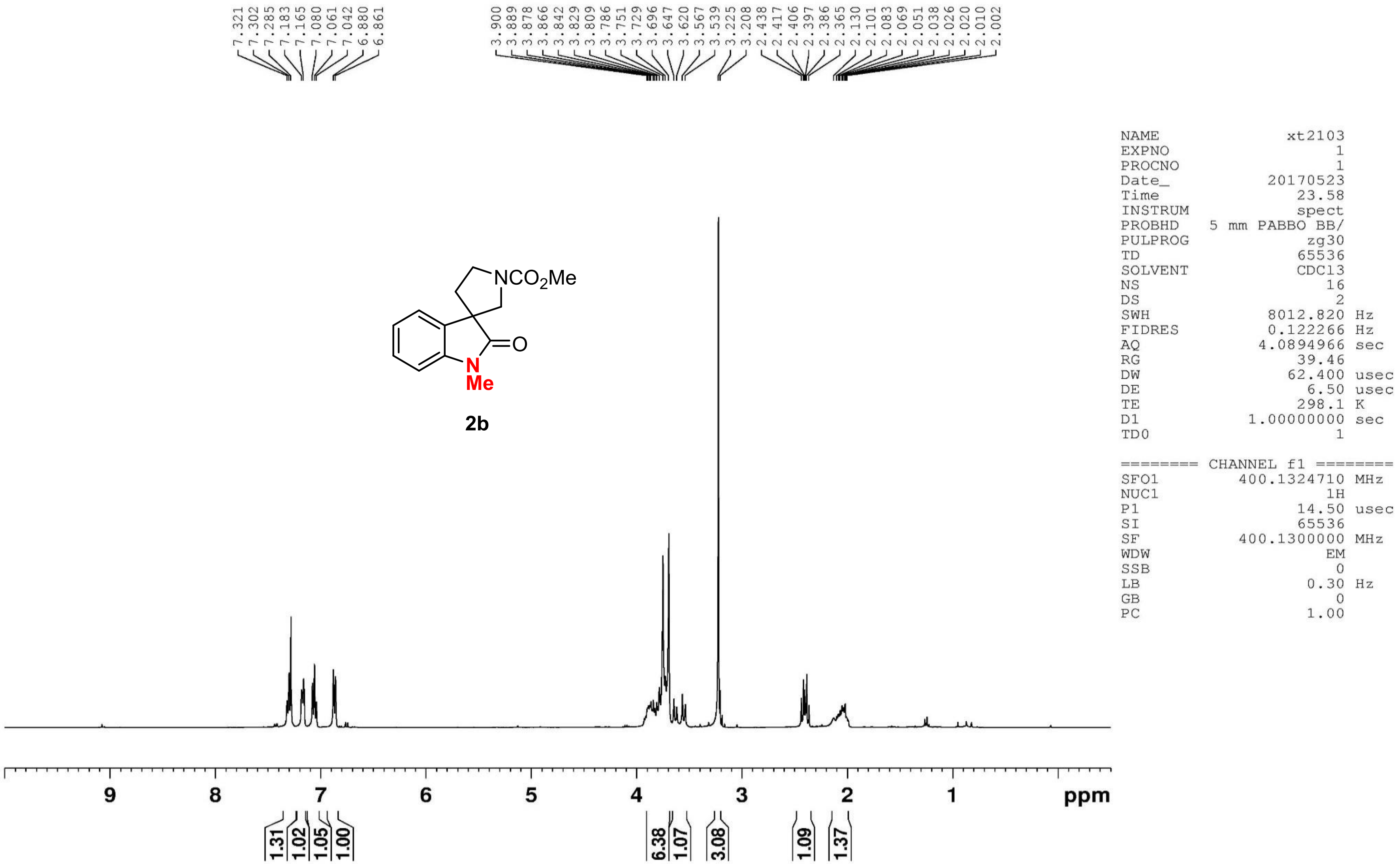

Supplementary Figure 38. ^{1}H-NMR of 2b

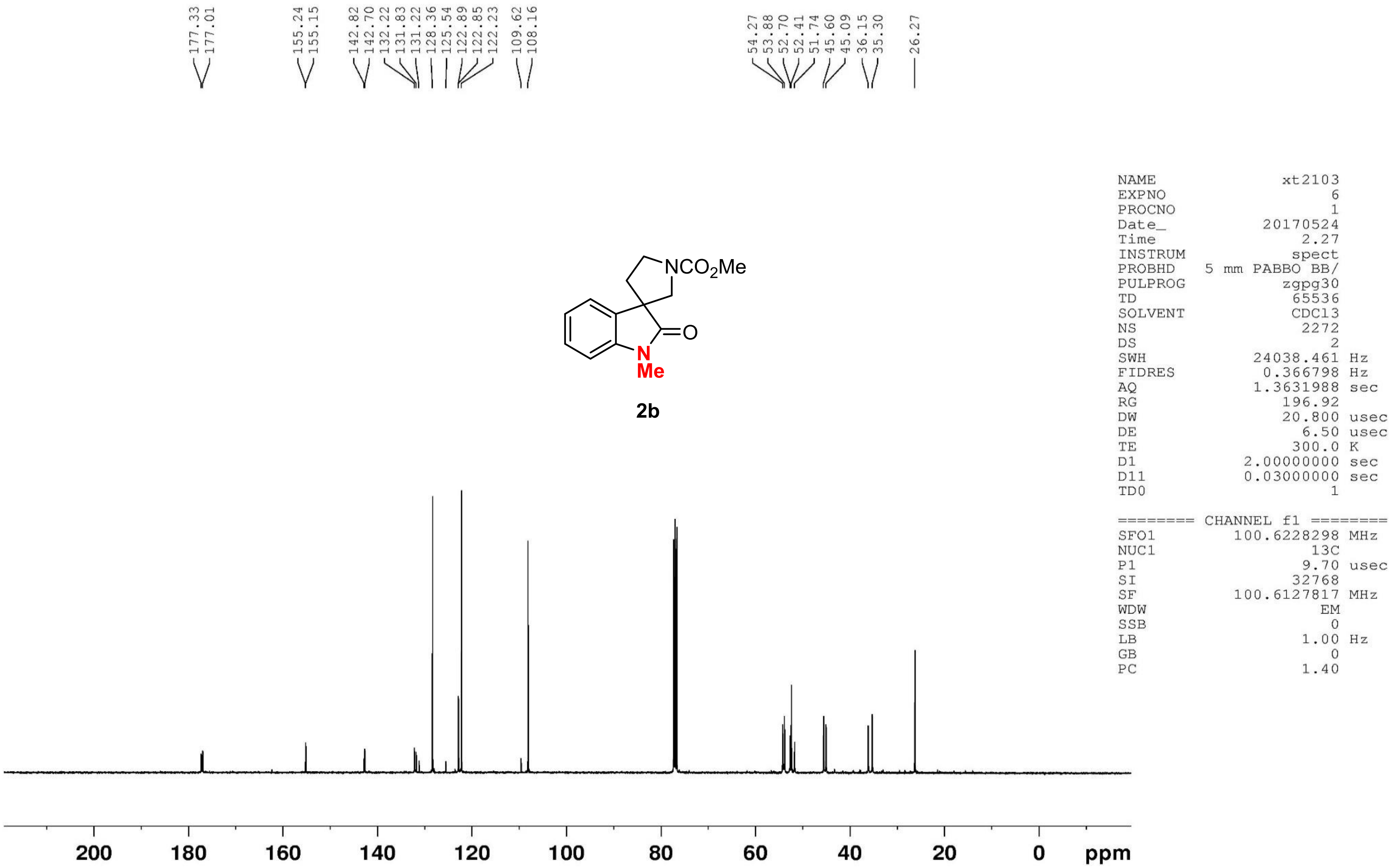

NAME	xt2103
EXPNO	6
PROCNO	1
Date_	20170524
Time	2.27
INSTRUM	spect
PROBHD	5 mm PABBO BB/
PULPROG	zgpg30
TD	65536
SOLVENT	CDCl3
NS	2272
DS	2
SWH	24038.461 Hz
FIDRES	0.366798 Hz
AQ	1.3631988 sec
RG	196.92
DW	20.800 usec
DE	6.50 usec
TE	300.0 K
D1	2.00000000 sec
D11	0.03000000 sec
TD0	1
======== CHANNEL f1 ========	
SFO1	100.6228298 MHz
NUC1	13C
P1	9.70 usec
SI	32768
SF	100.6127817 MHz
WDW	EM
SSB	0
LB	1.00 Hz
GB	0
PC	1.40

Supplementary Figure 39. ^{13}C-NMR of 2b

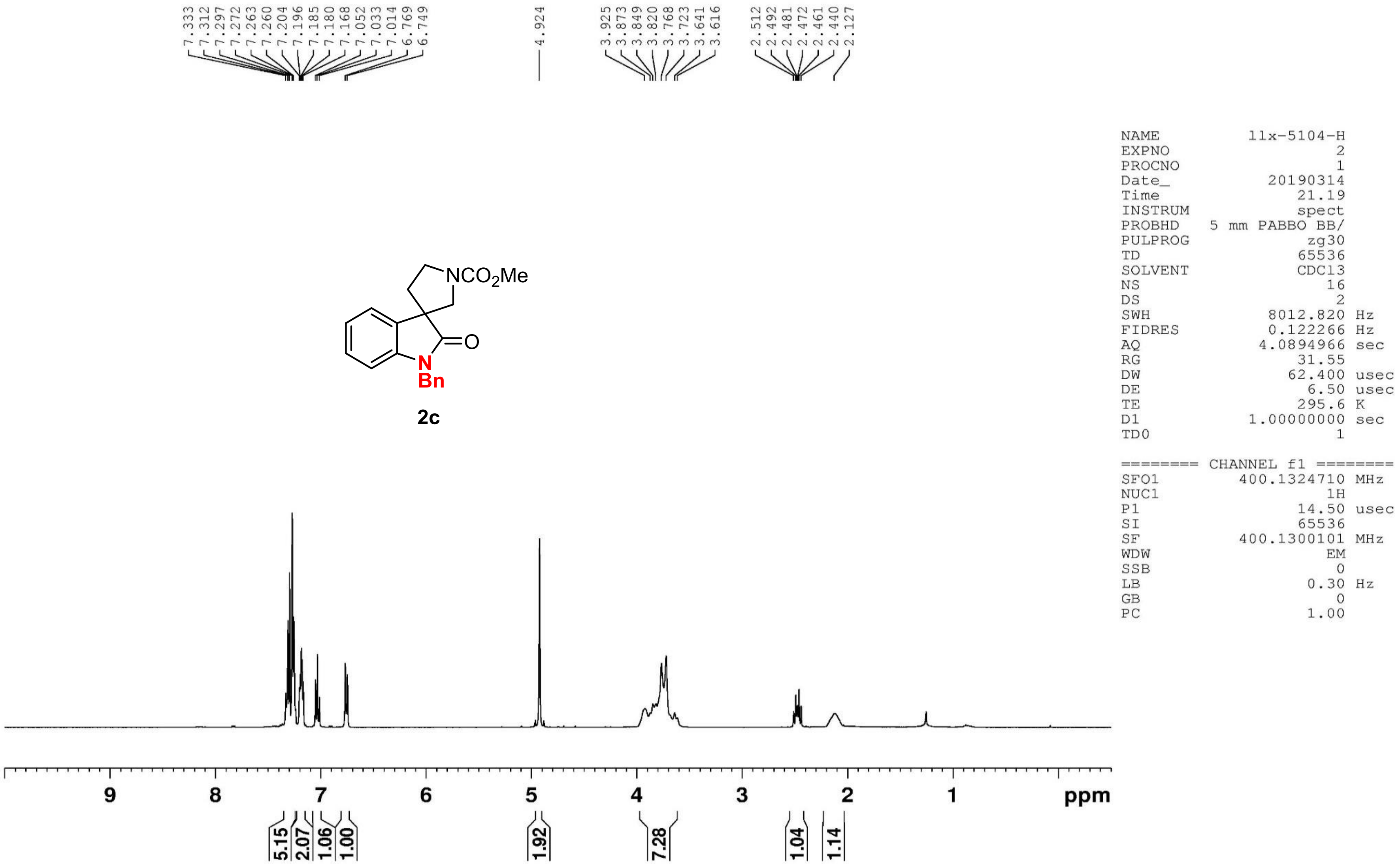

Supplementary Figure 40. ^{1}H-NMR of 2c

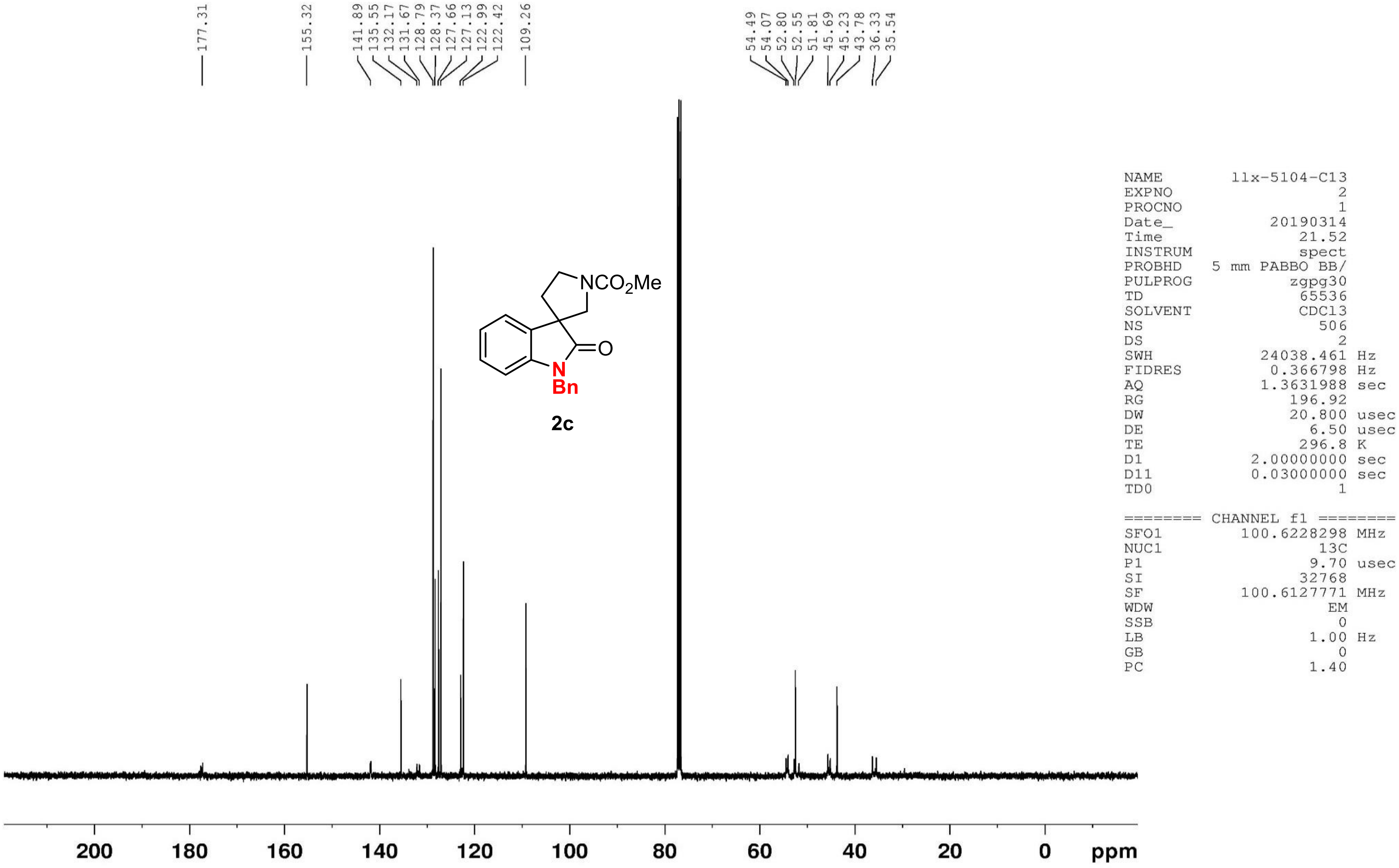

NAME	llx-5104-C13
EXPNO	2
PROCNO	1
Date_	20190314
Time	21.52
INSTRUM	spect
PROBHD	5 mm PABBO BB/
PULPROG	zgpg30
TD	65536
SOLVENT	CDCl3
NS	506
DS	2
SWH	24038.461 Hz
FIDRES	0.366798 Hz
AQ	1.3631988 sec
RG	196.92
DW	20.800 usec
DE	6.50 usec
TE	296.8 K
D1	2.00000000 sec
D11	0.03000000 sec
TD0	1
======== CHANNEL f1 ========	
SFO1	100.6228298 MHz
NUC1	13C
P1	9.70 usec
SI	32768
SF	100.6127771 MHz
WDW	EM
SSB	0
LB	1.00 Hz
GB	0
PC	1.40

Supplementary Figure 41. ^{13}C-NMR of 2c

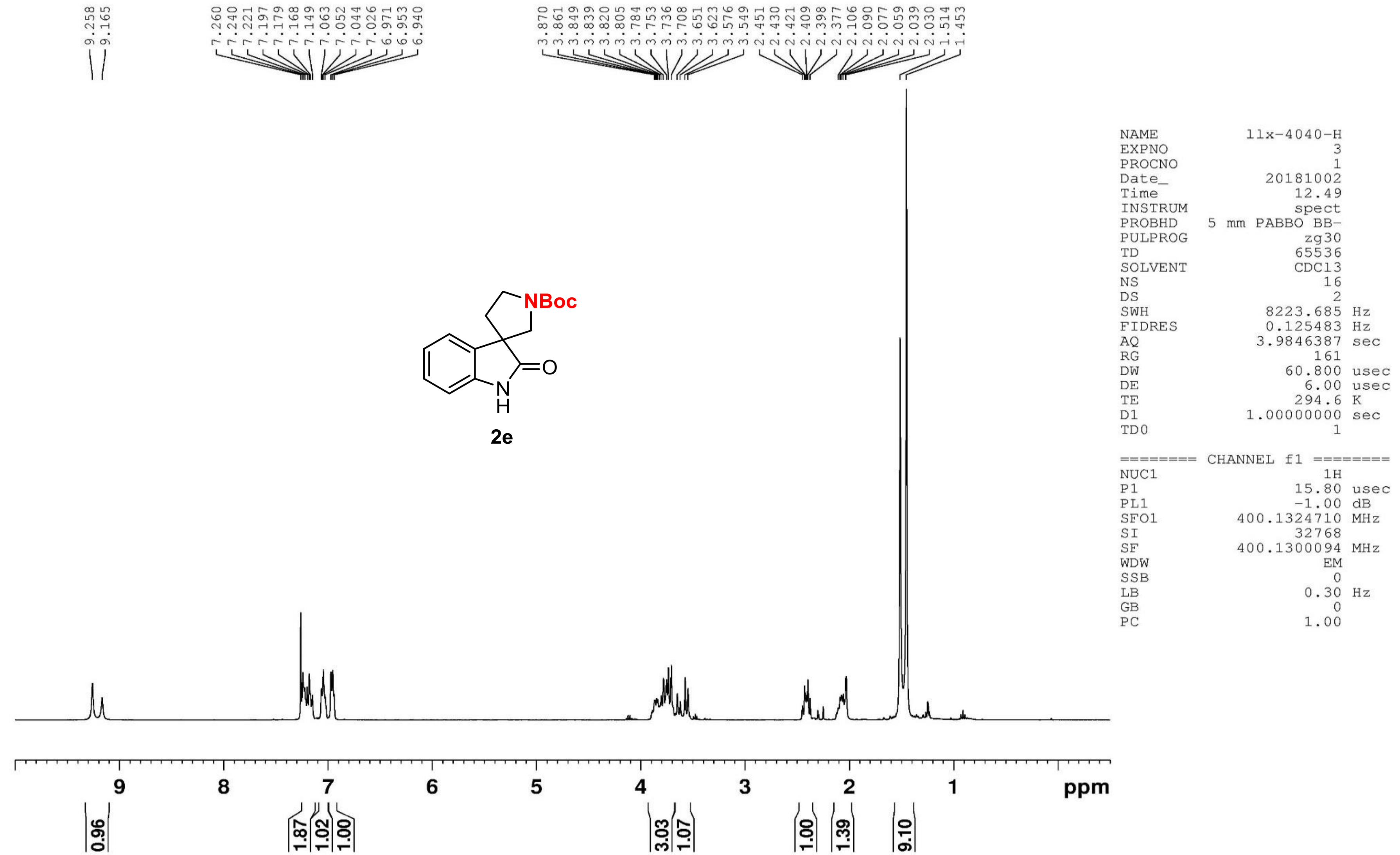

Supplementary Figure 42. ^{1}H-NMR of 2e

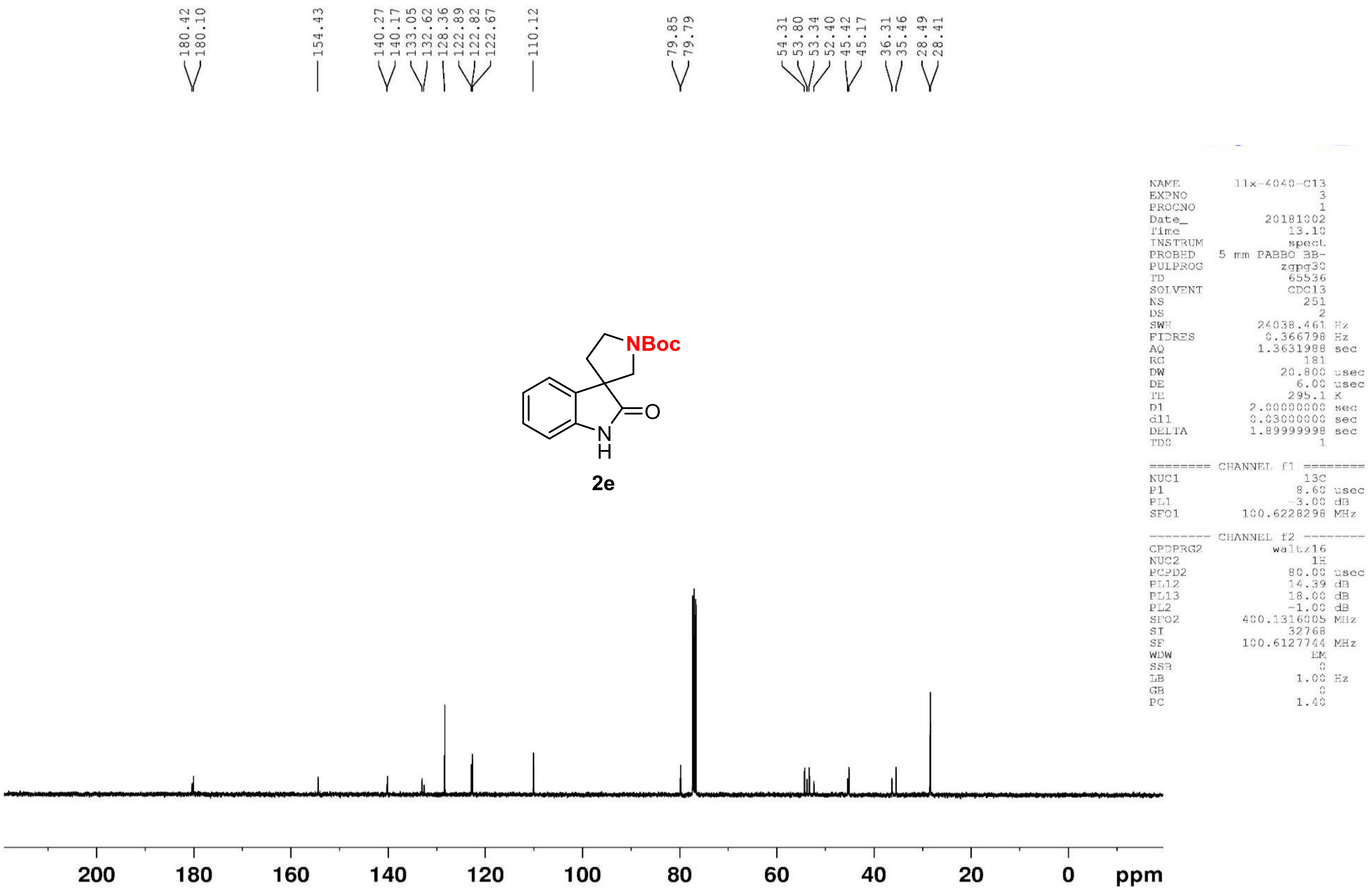

Supplementary Figure 43. ^{13}C-NMR of 2e

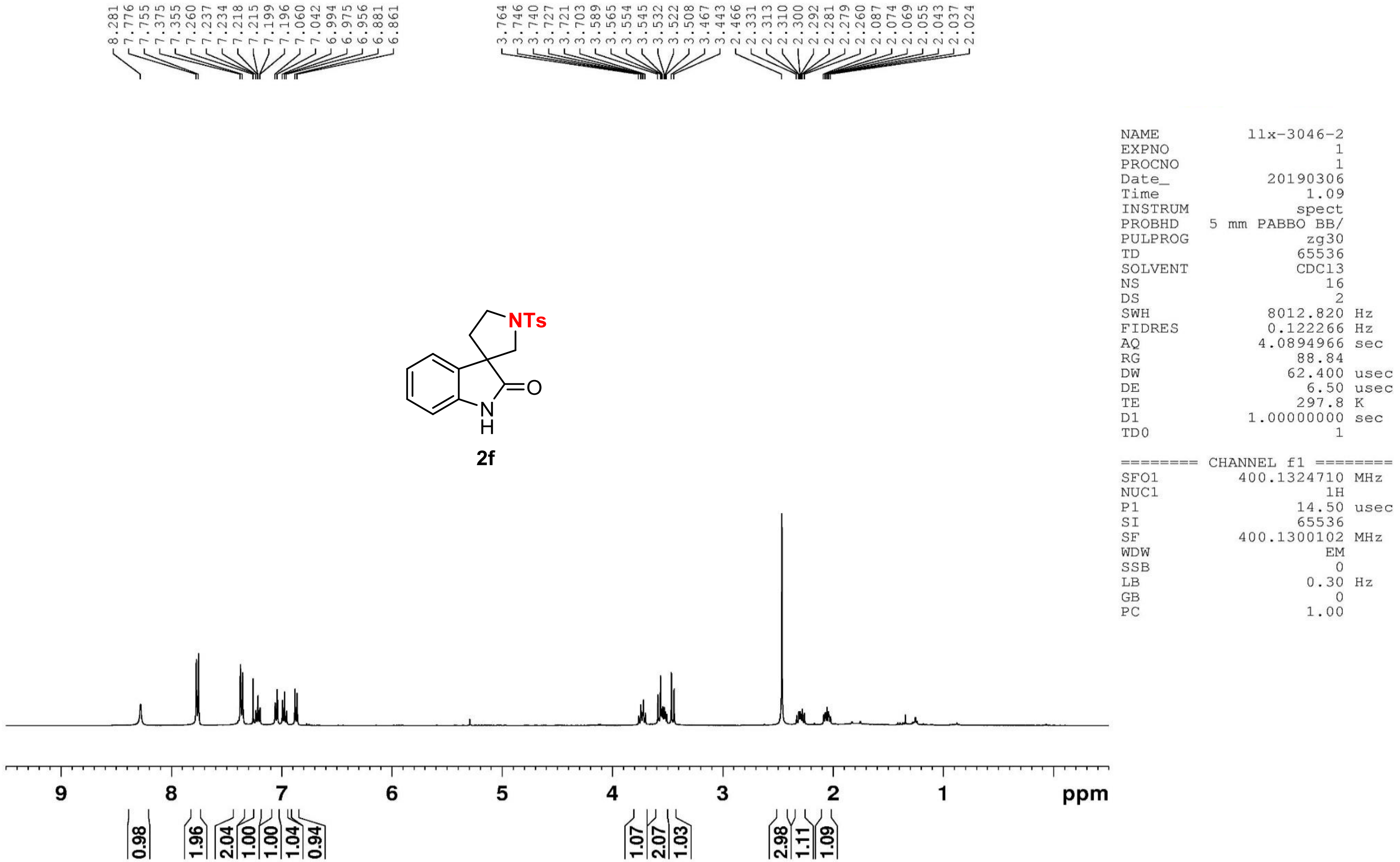

Supplementary Figure 44. ^{1}H-NMR of 2f

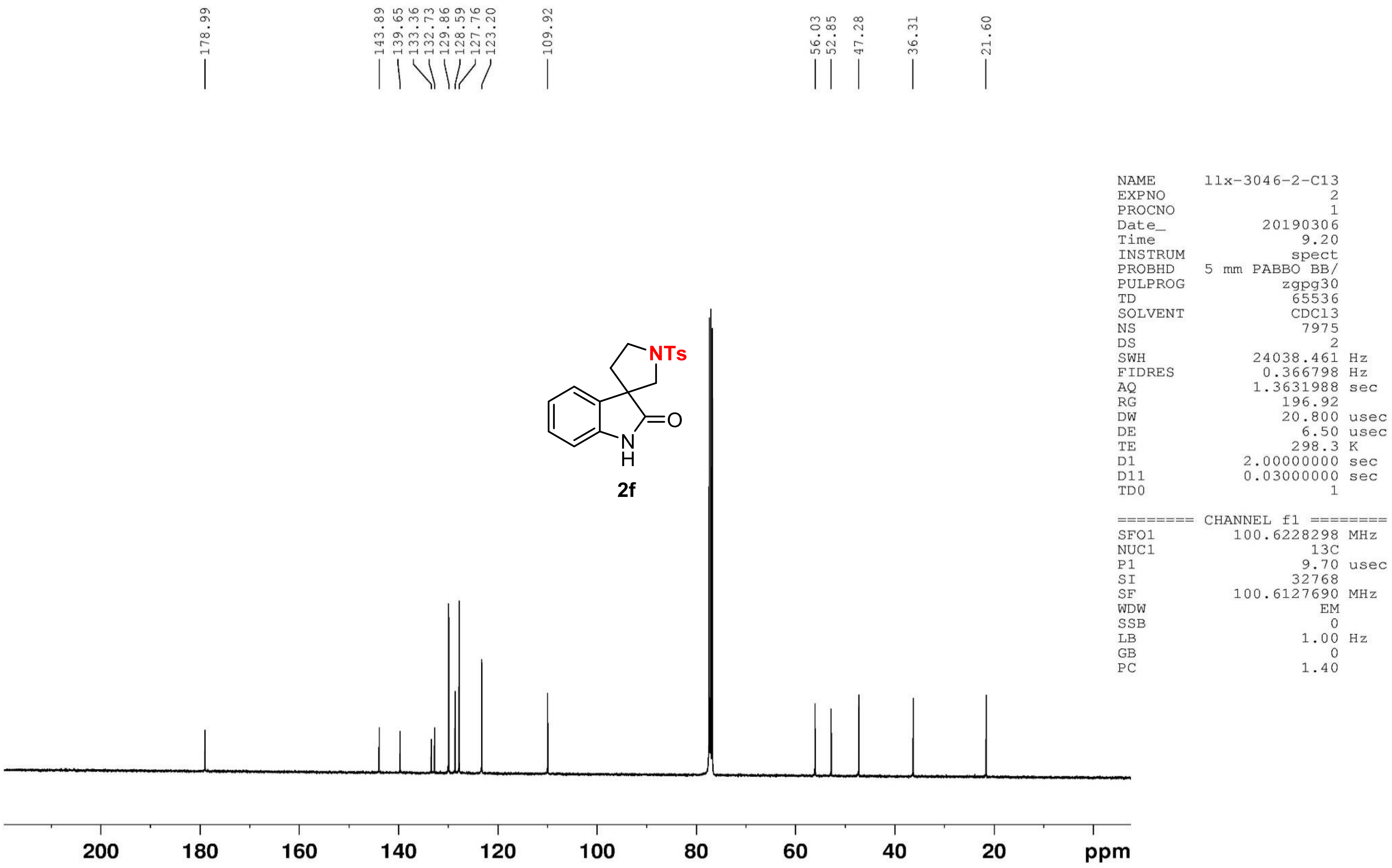

Supplementary Figure 45. ^{13}C-NMR of 2f

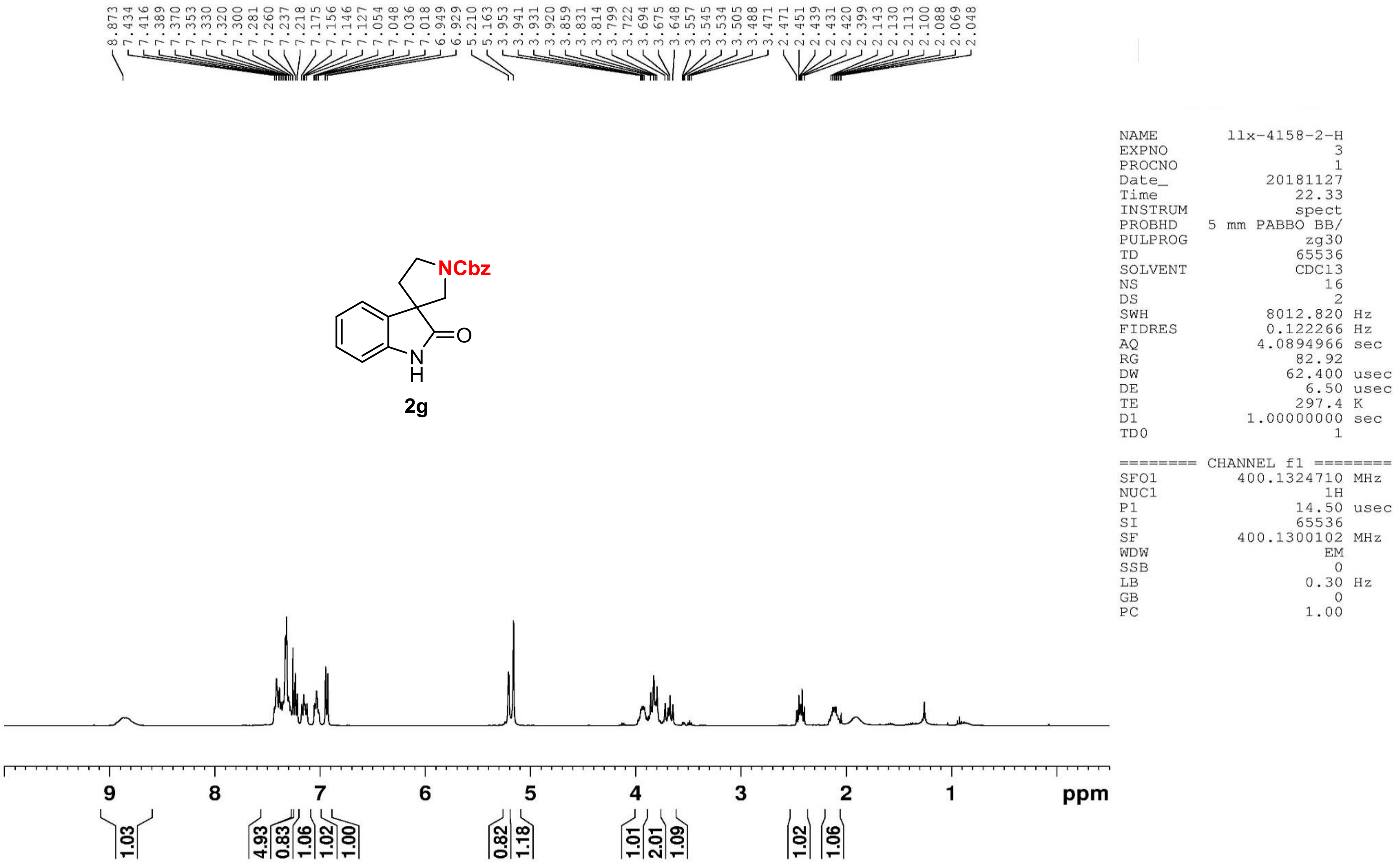

Supplementary Figure 46. ^{1}H-NMR of 2g

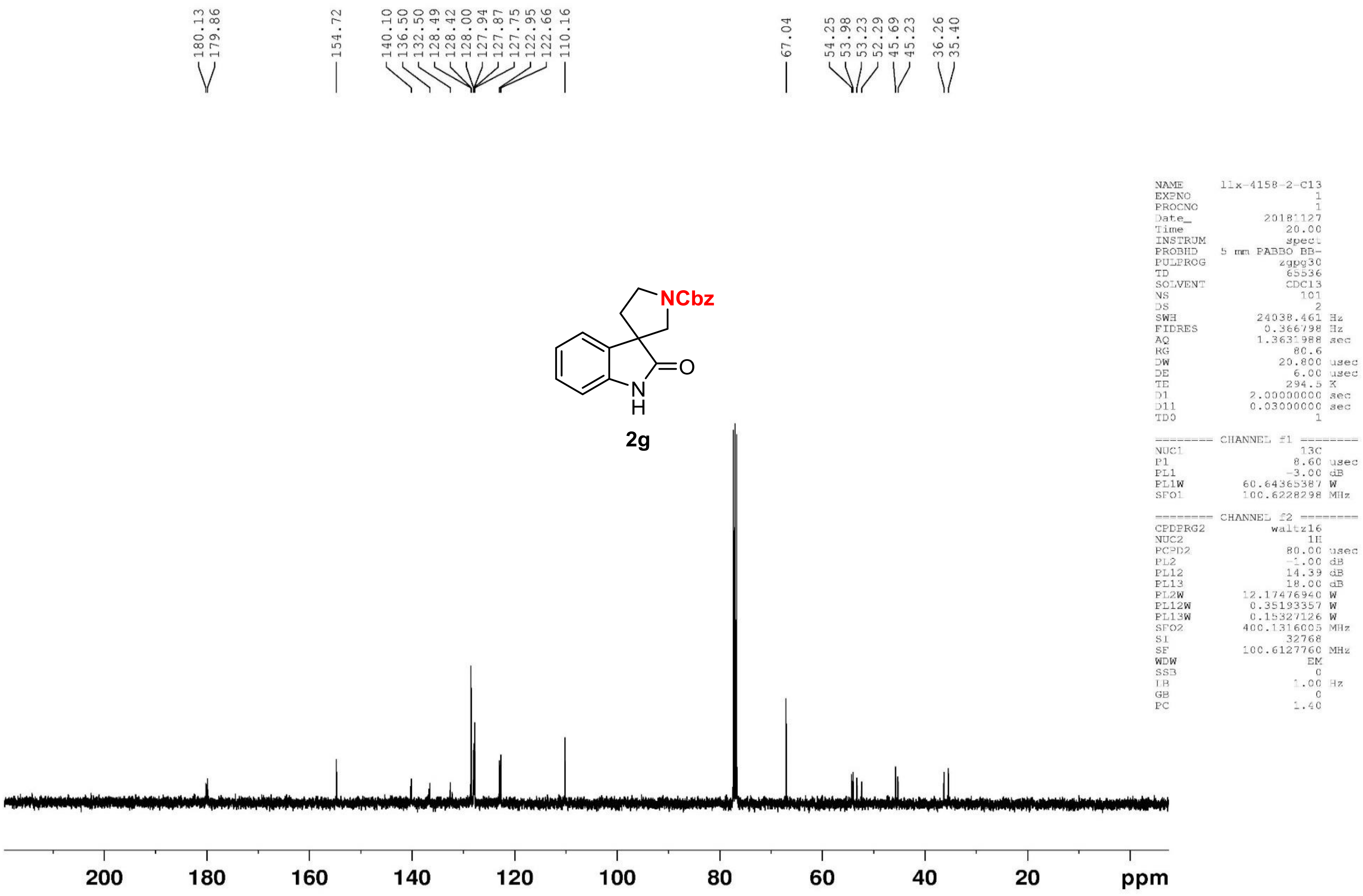

Supplementary Figure 47. ^{13}C-NMR of 2g

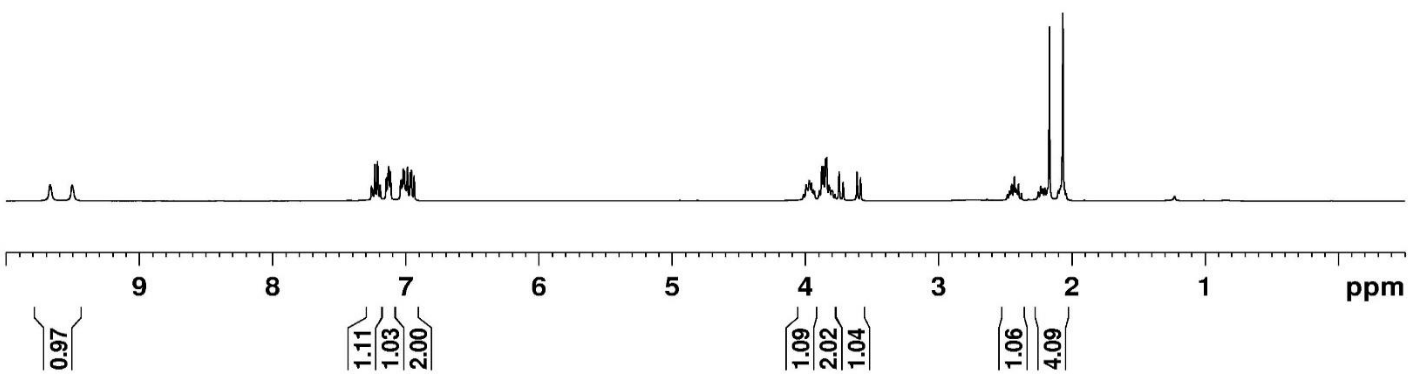

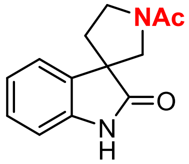

2h

```
NAME          llx-4147-H
EXPNO                  2
PROCNO                 1
Date_           20181122
Time               13.21
INSTRUM            spect
PROBHD   5 mm PABBO BB/
PULPROG             zg30
TD                 65536
SOLVENT            CDCl3
NS                    16
DS                     2
SWH             8012.820 Hz
FIDRES          0.122266 Hz
AQ             4.0894966 sec
RG                 34.77
DW                62.400 usec
DE                  6.50 usec
TE                 294.8 K
D1            1.00000000 sec
TD0                    1

======== CHANNEL f1 ========
SFO1         400.1324710 MHz
NUC1                  1H
P1                 14.50 usec
SI                 65536
SF           400.1300102 MHz
WDW                   EM
SSB                    0
LB                  0.30 Hz
GB                     0
PC                  1.00
```

Supplementary Figure 48. ^{1}H-NMR of 2h

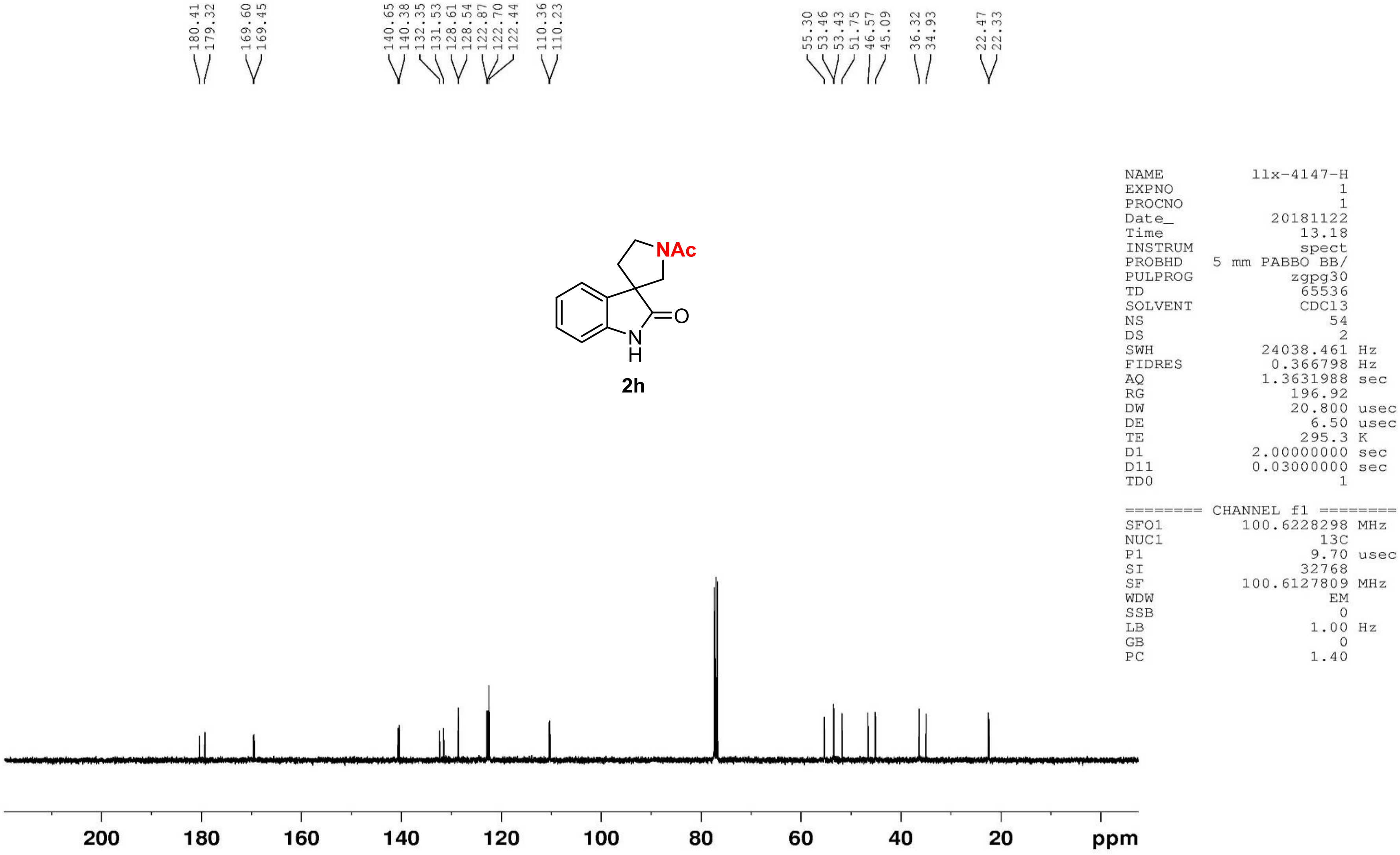

Supplementary Figure 49. ^{13}**C-NMR of 2h**

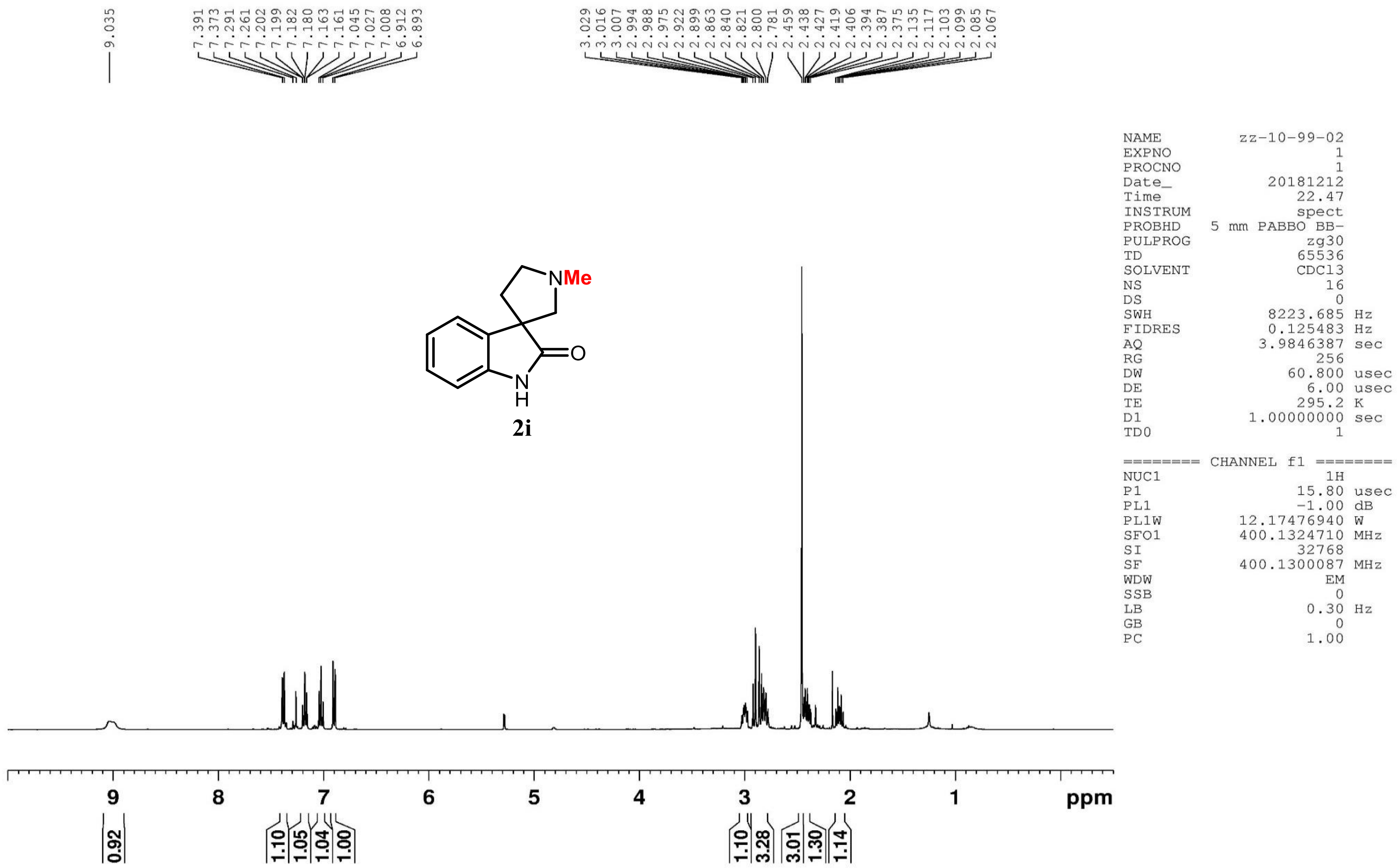

Supplementary Figure 50. ^{1}**H-NMR of 2i**

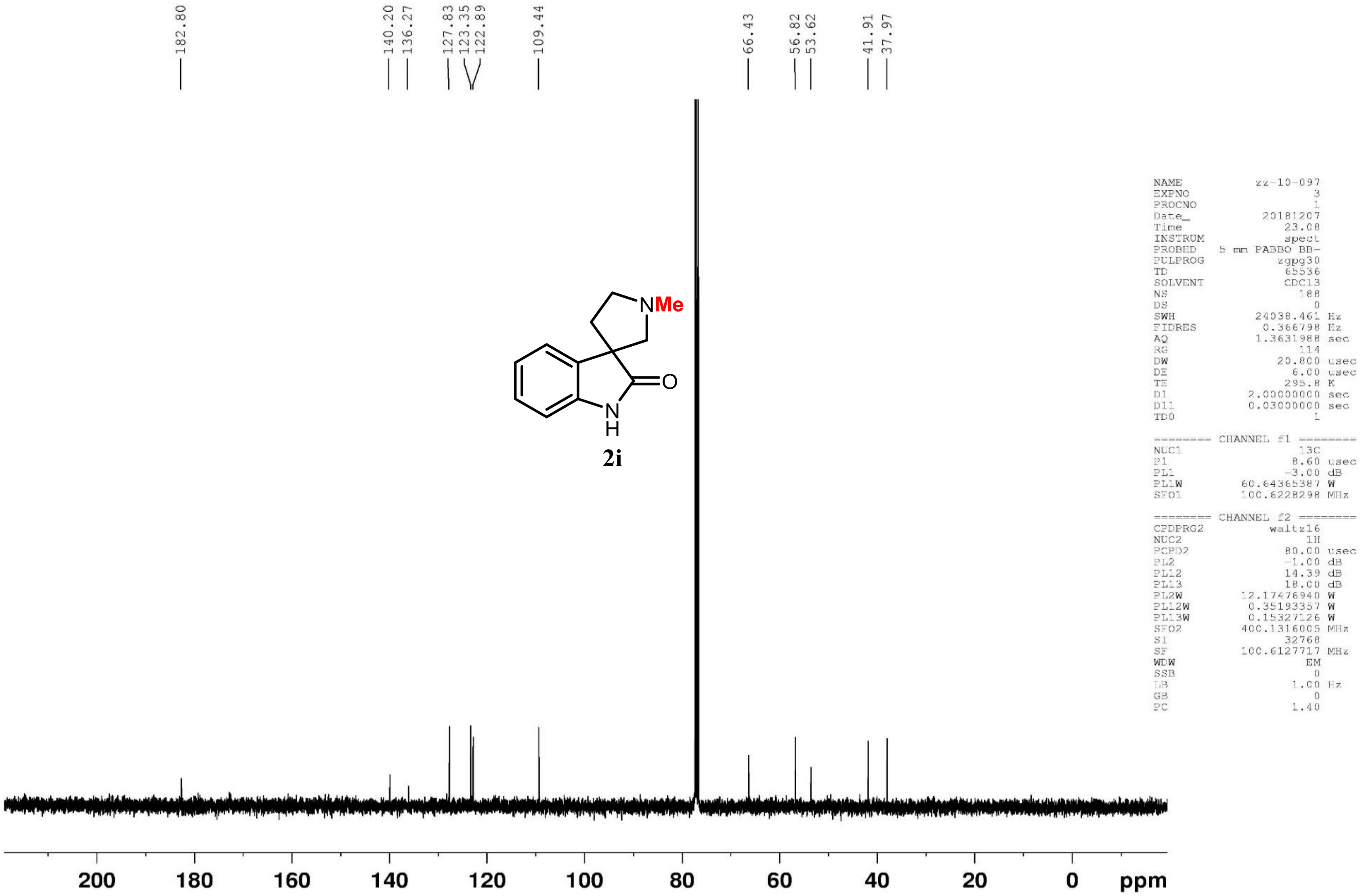

Supplementary Figure 51. ^{13}C-NMR of 2i

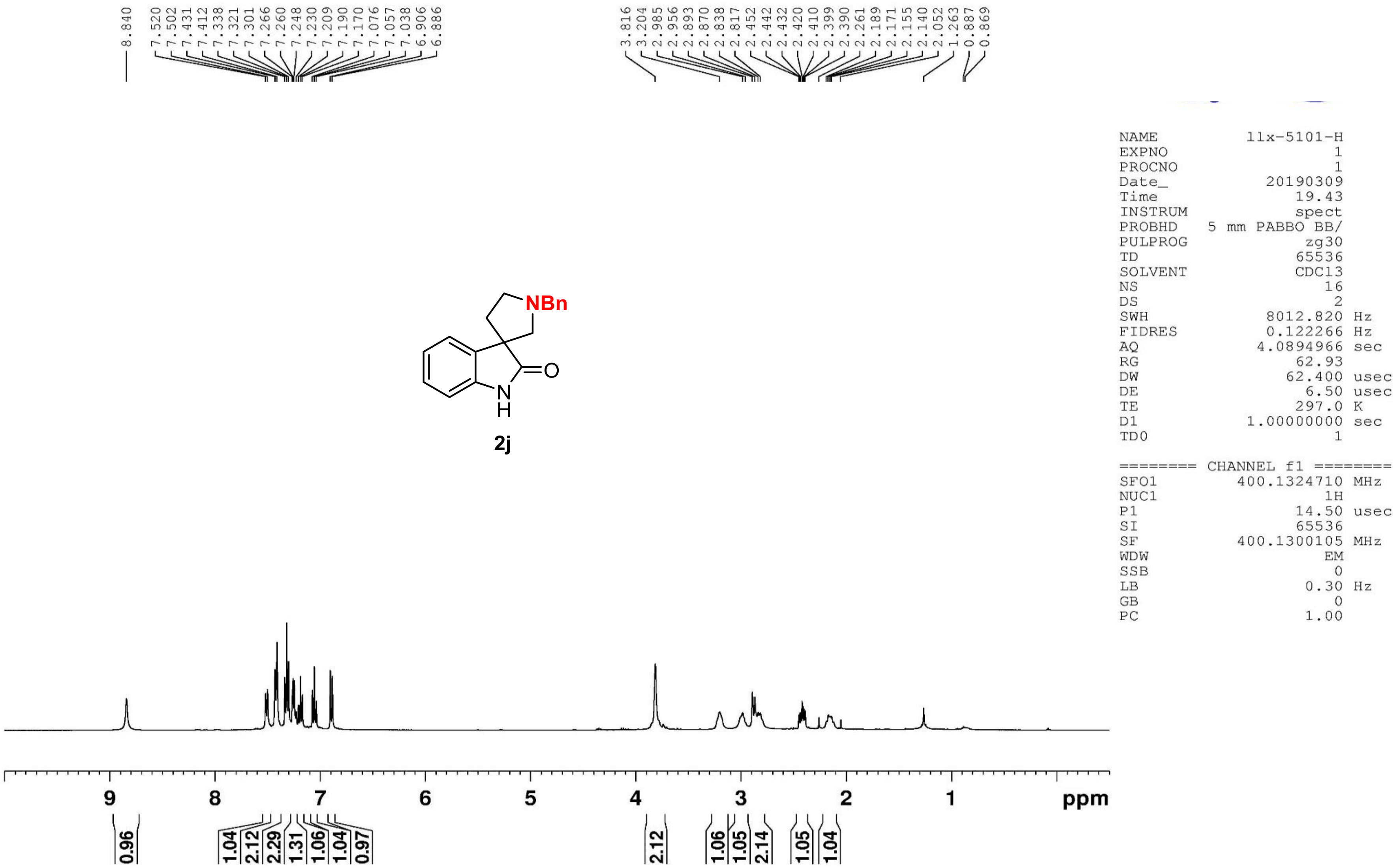

Supplementary Figure 52. ^{1}H-NMR of 2j

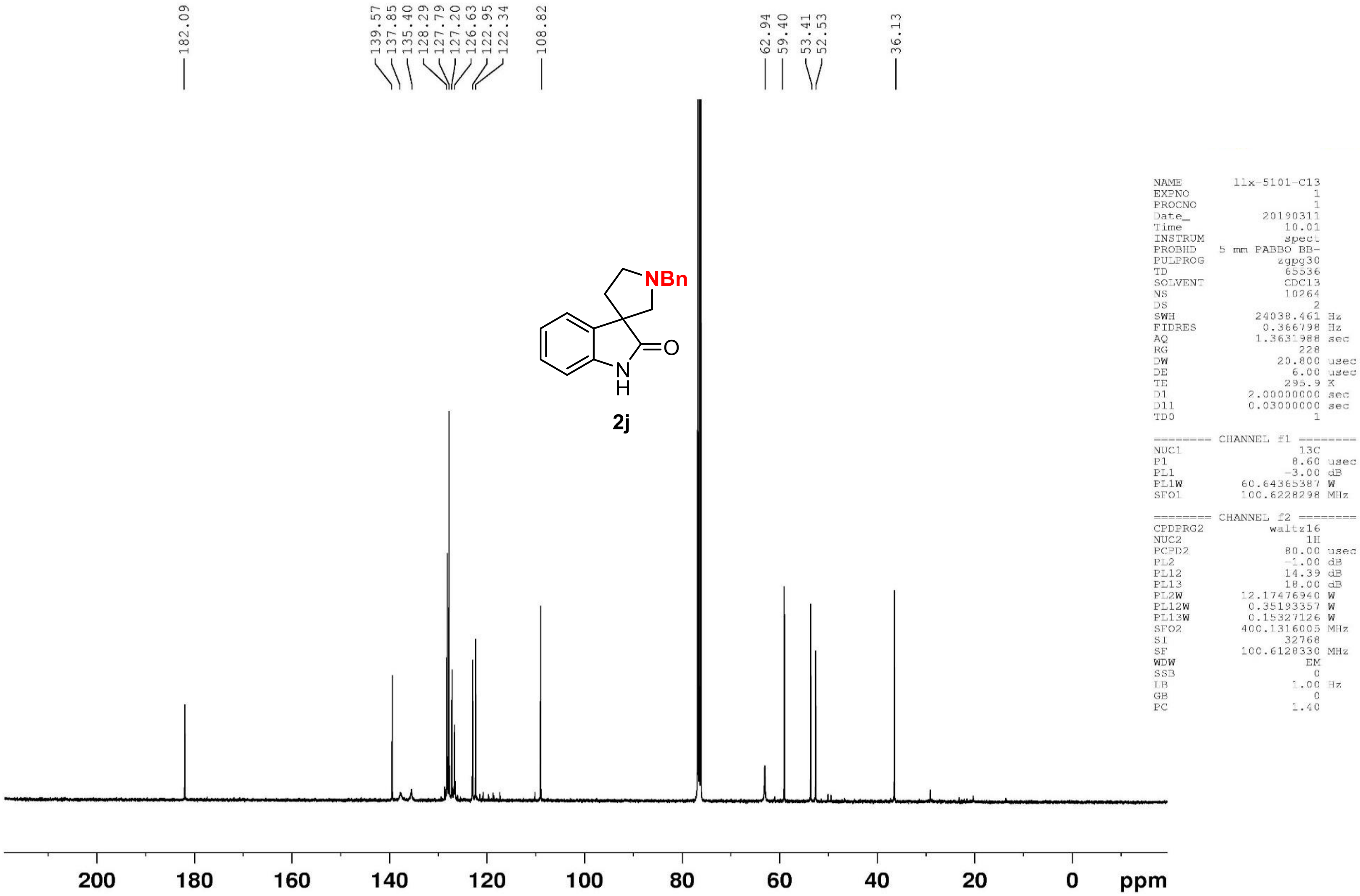

Supplementary Figure 53. ^{13}C-NMR of 2j

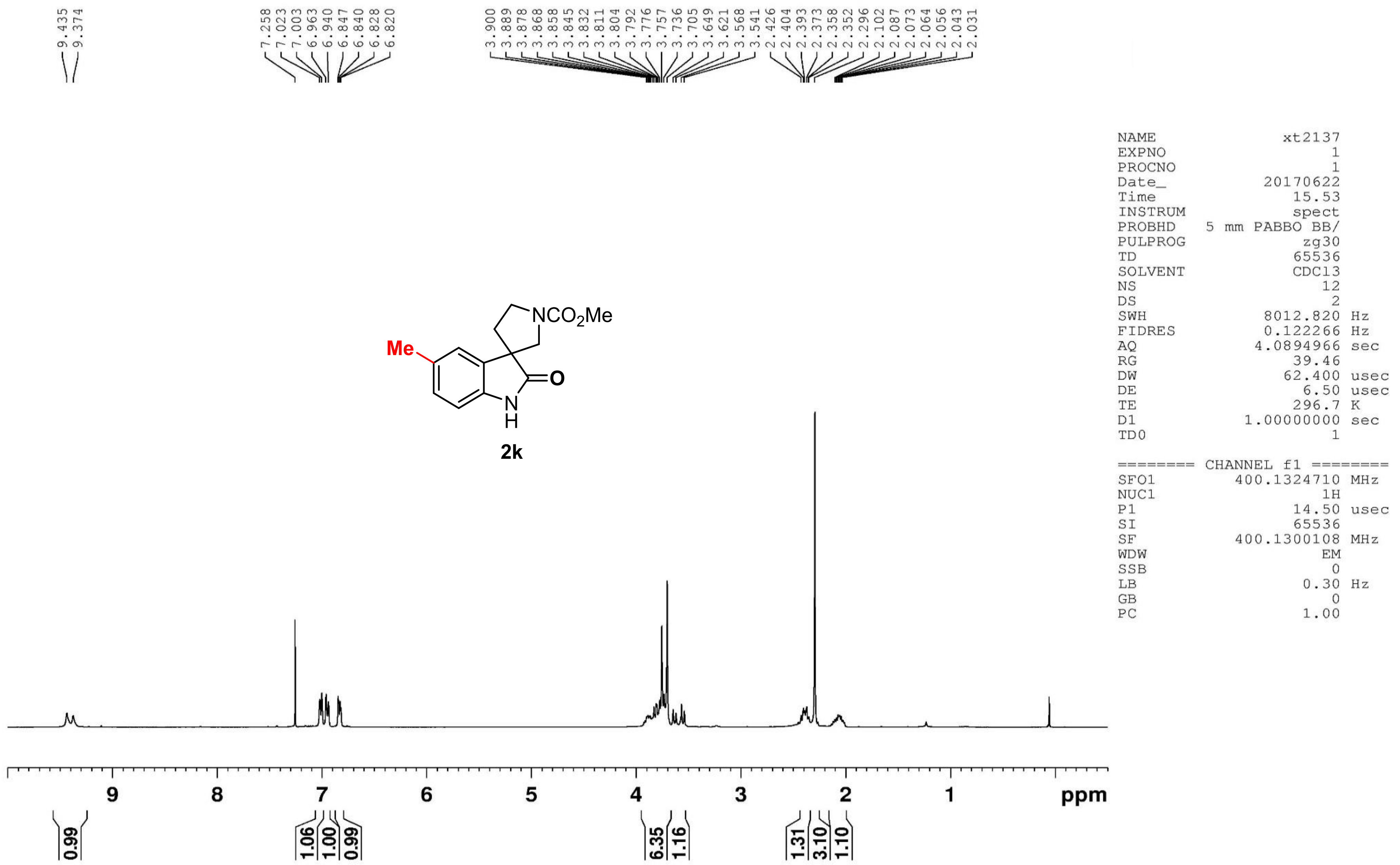

Supplementary Figure 54. ^{1}**H-NMR of 2k**

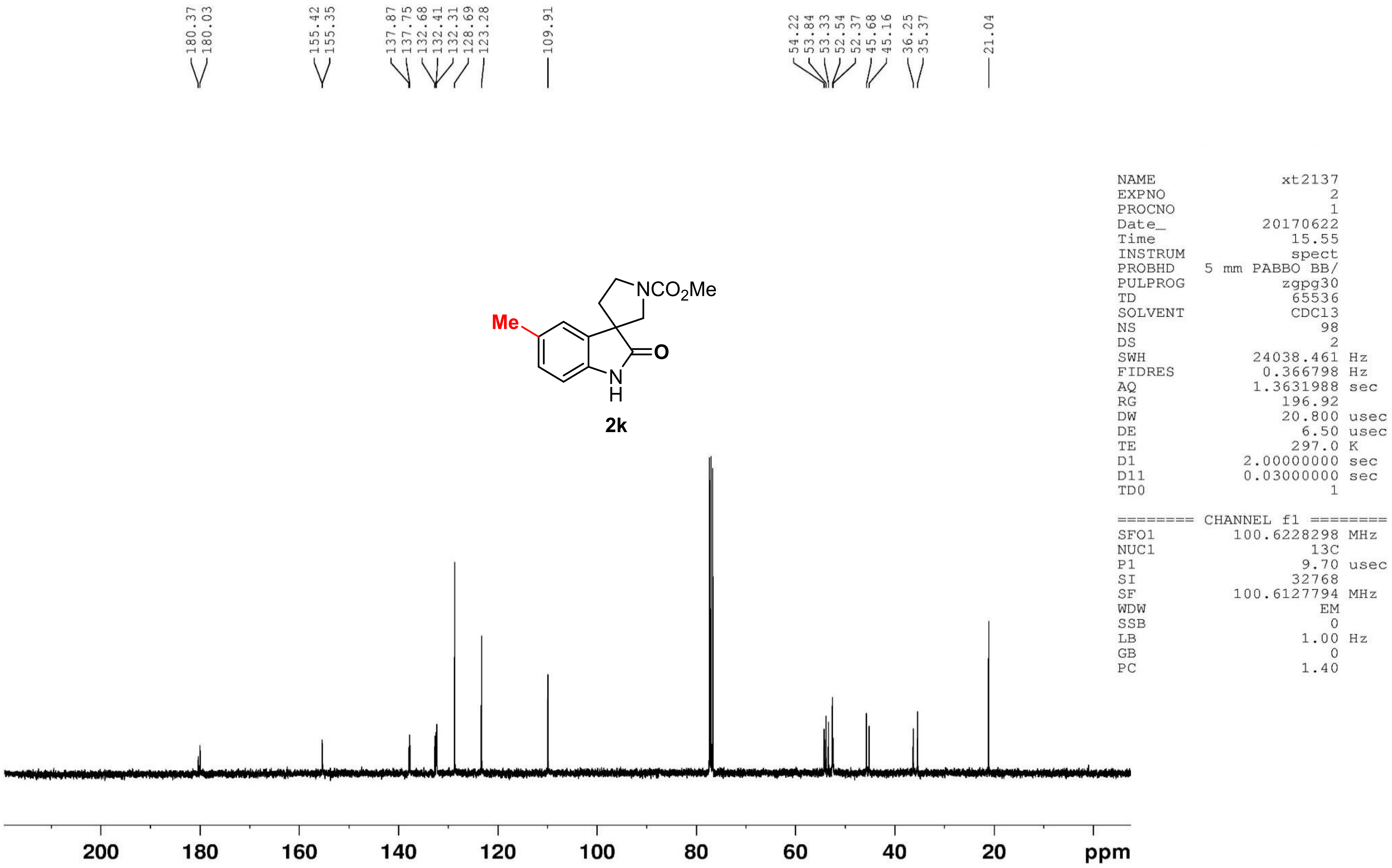

Supplementary Figure 55. ^{13}C-NMR of 2k

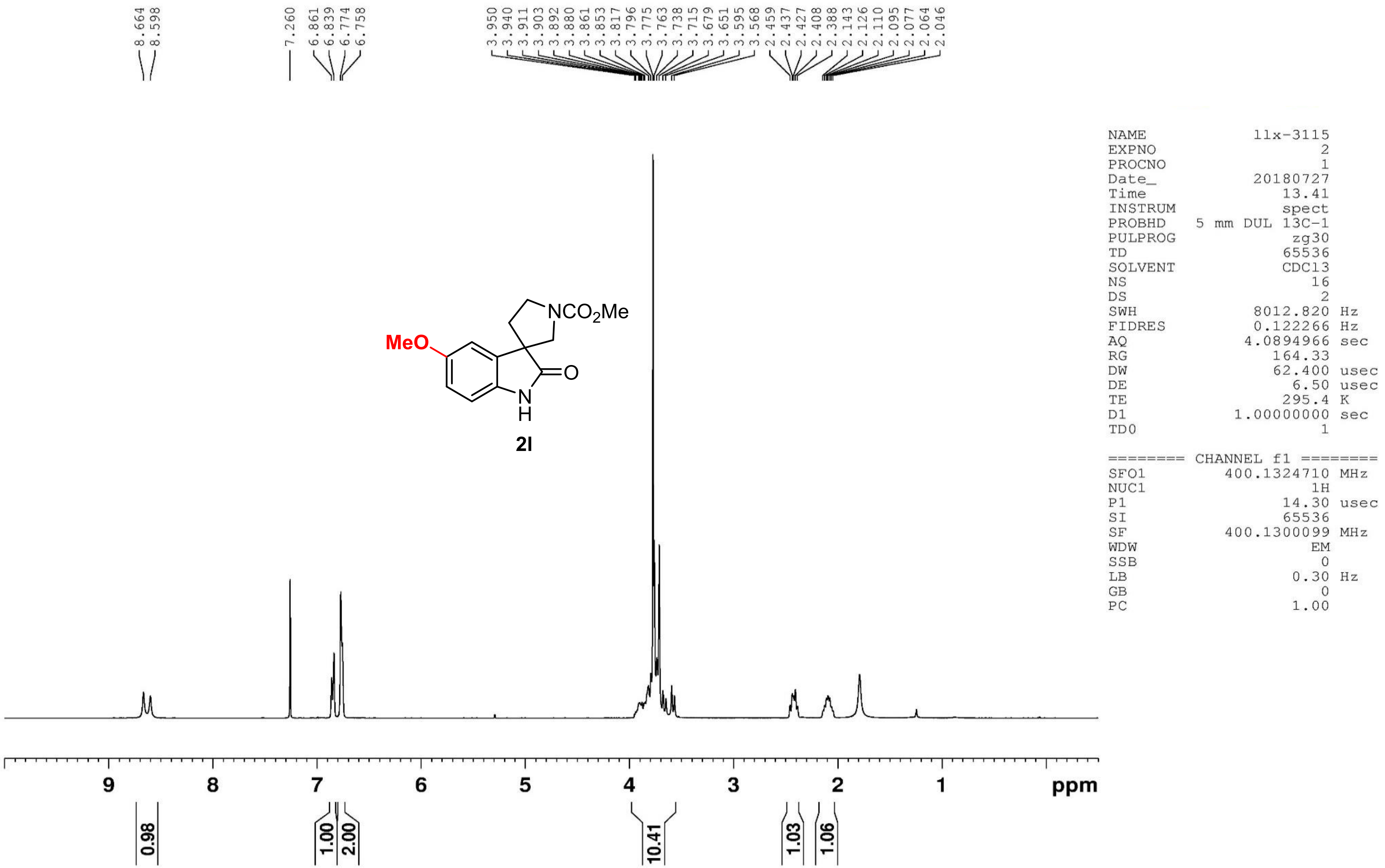

Supplementary Figure 56. ^{1}H-NMR of 2l

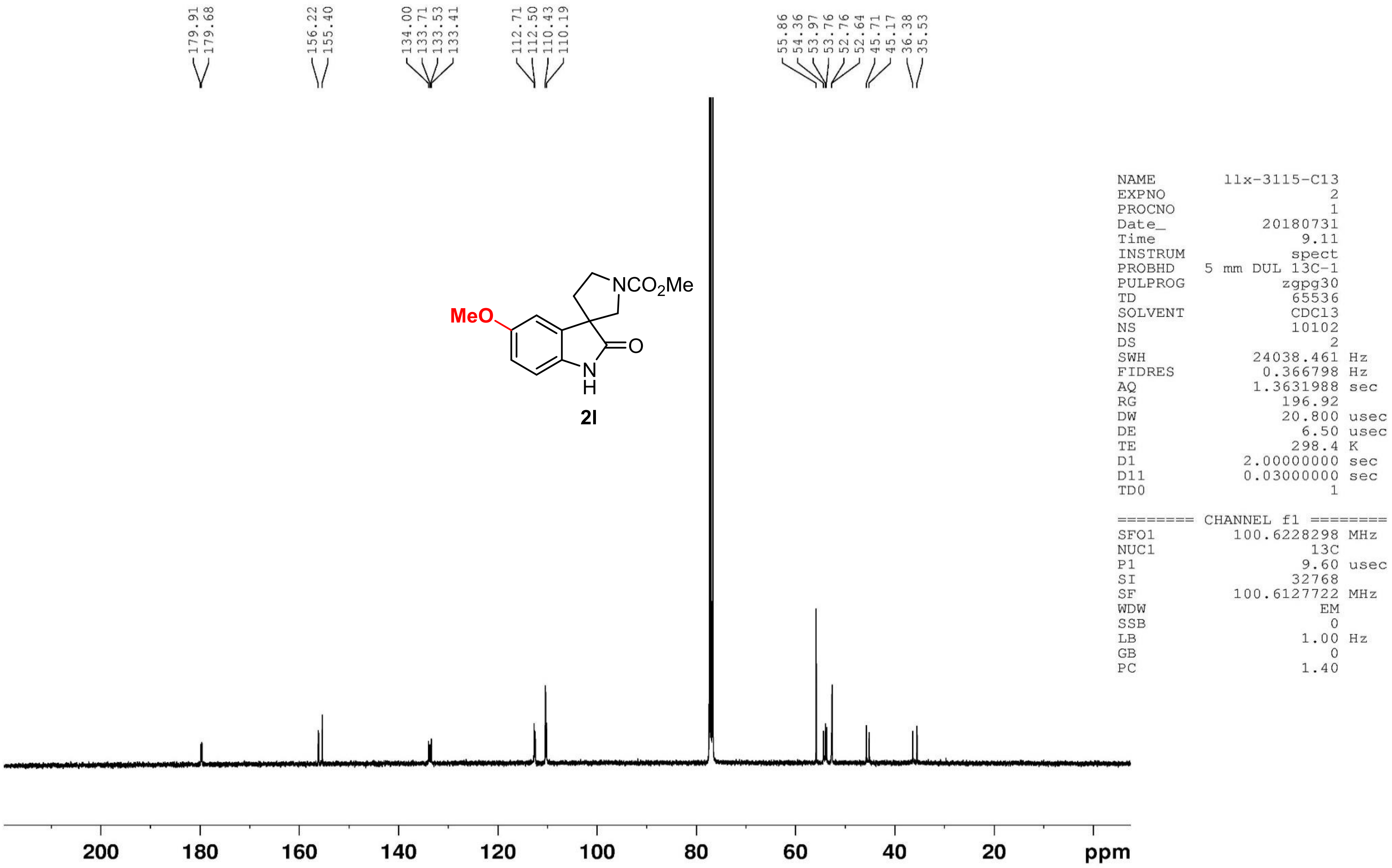

Supplementary Figure 57. ^{13}C-NMR of 2l

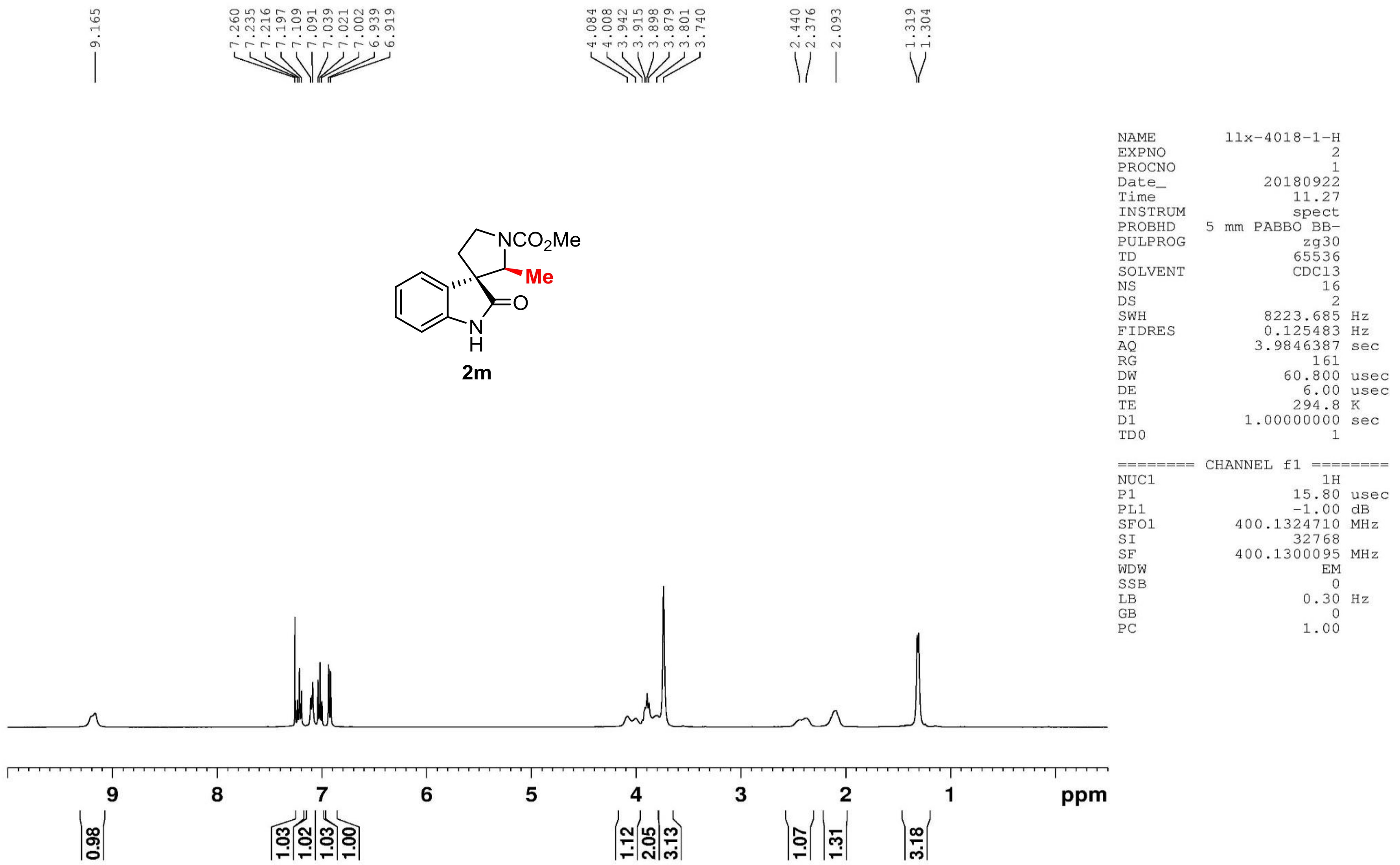

Supplementary Figure 58. ^{1}H-NMR of 2m

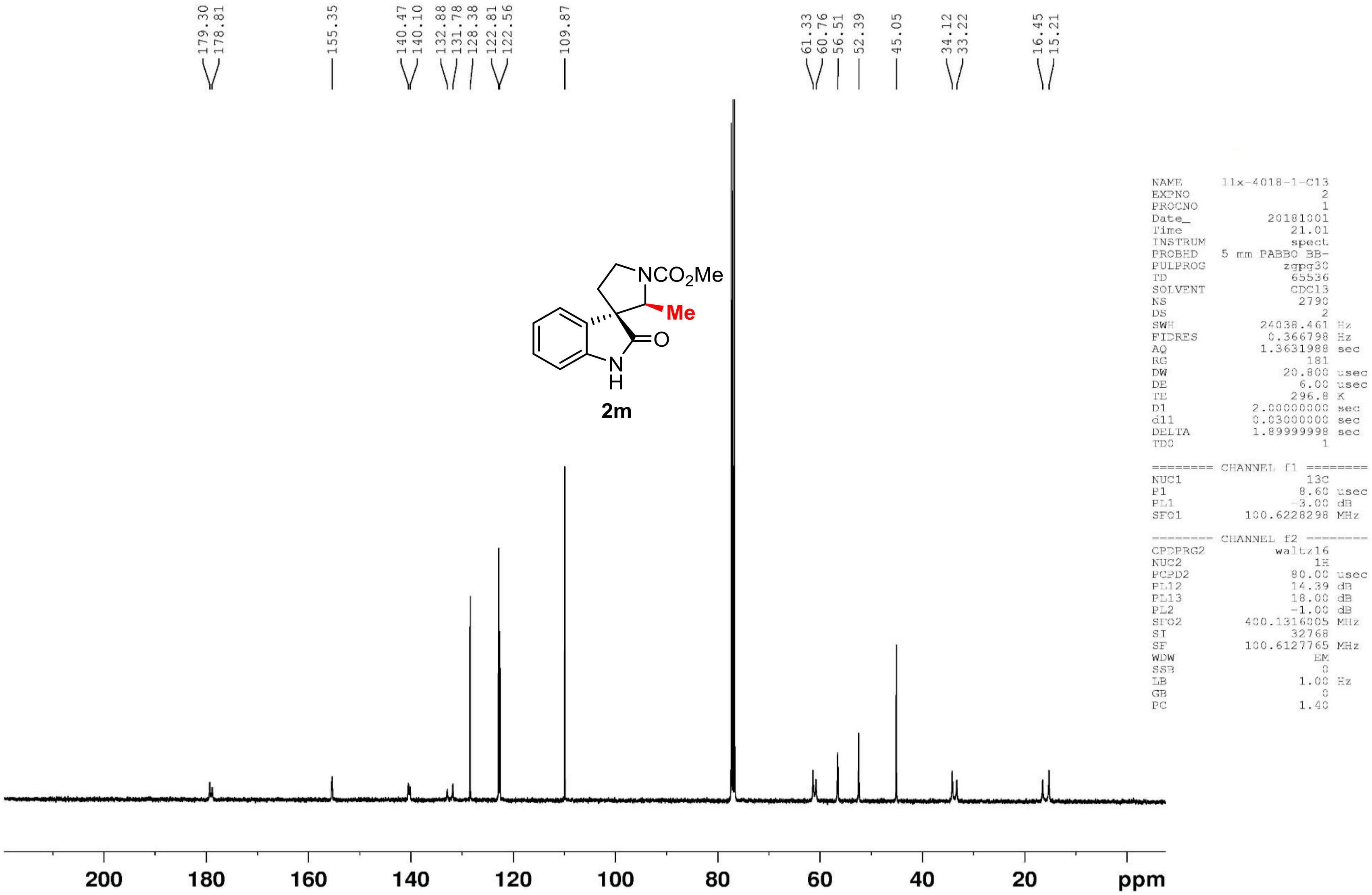

Supplementary Figure 59. ^{13}C-NMR of 2m

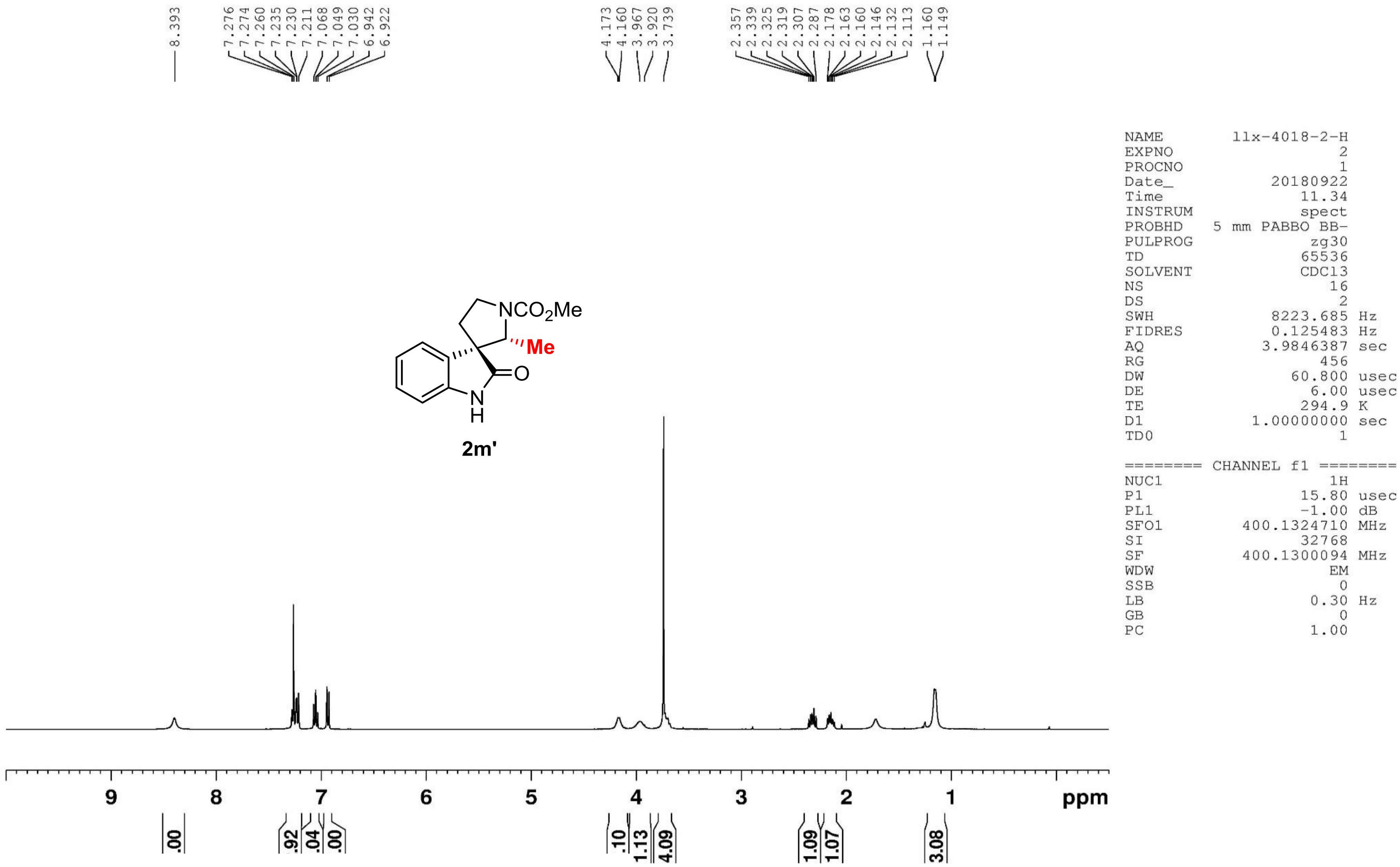

Supplementary Figure 60. ^{1}H-NMR of 2m'

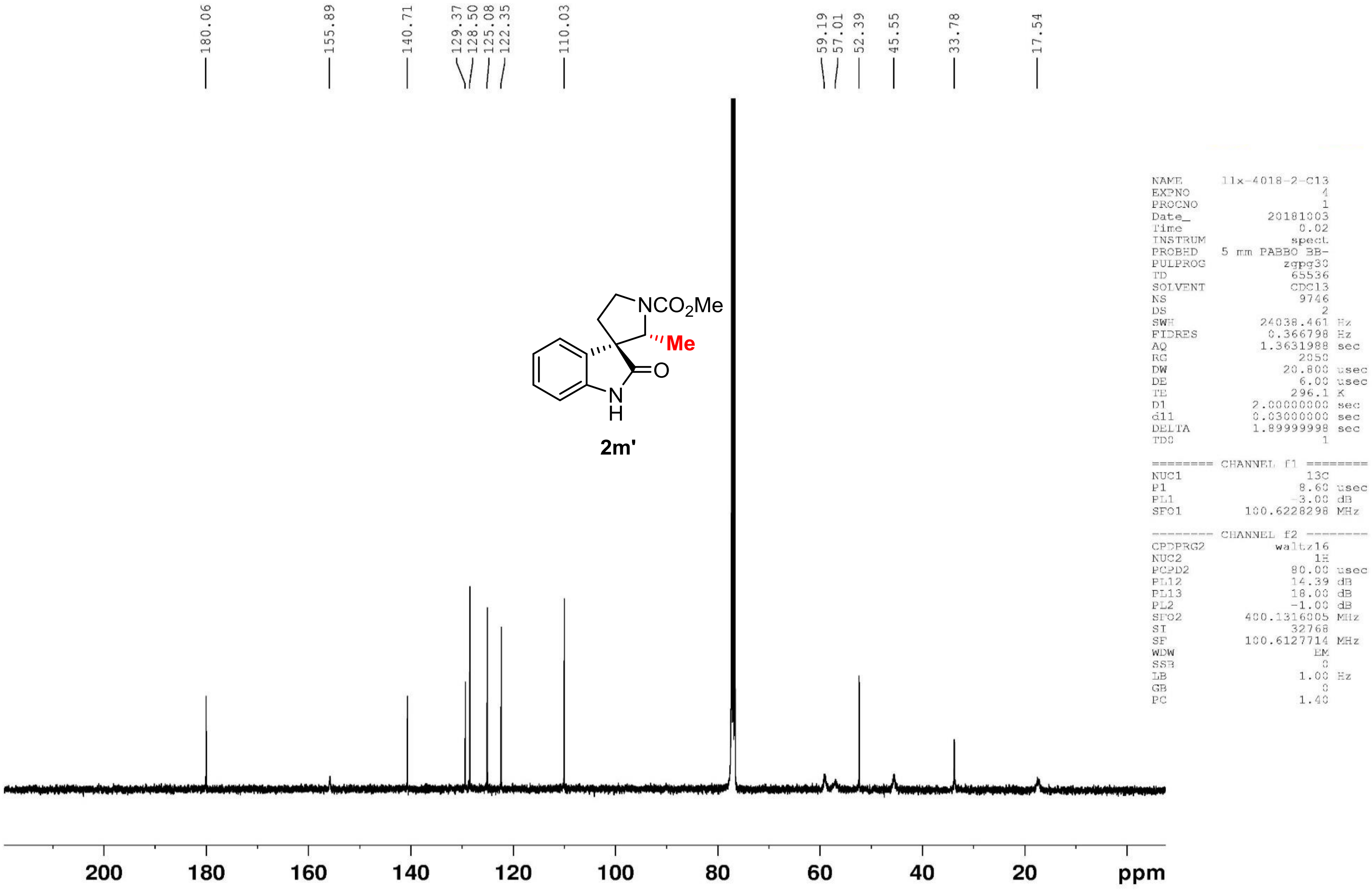

Supplementary Figure 61. ^{13}C-NMR of 2m'

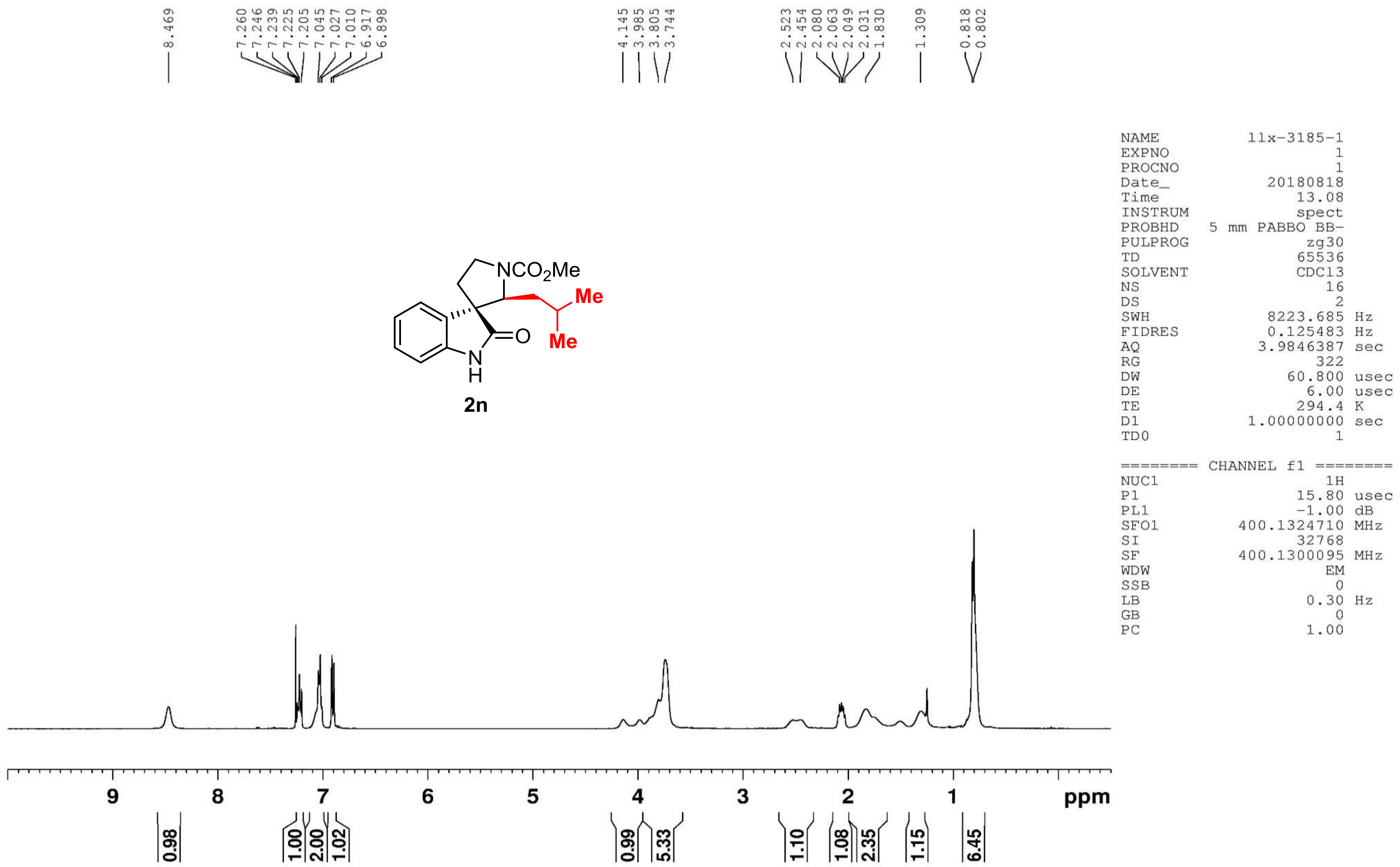

Supplementary Figure 62. ^{1}H-NMR of 2n

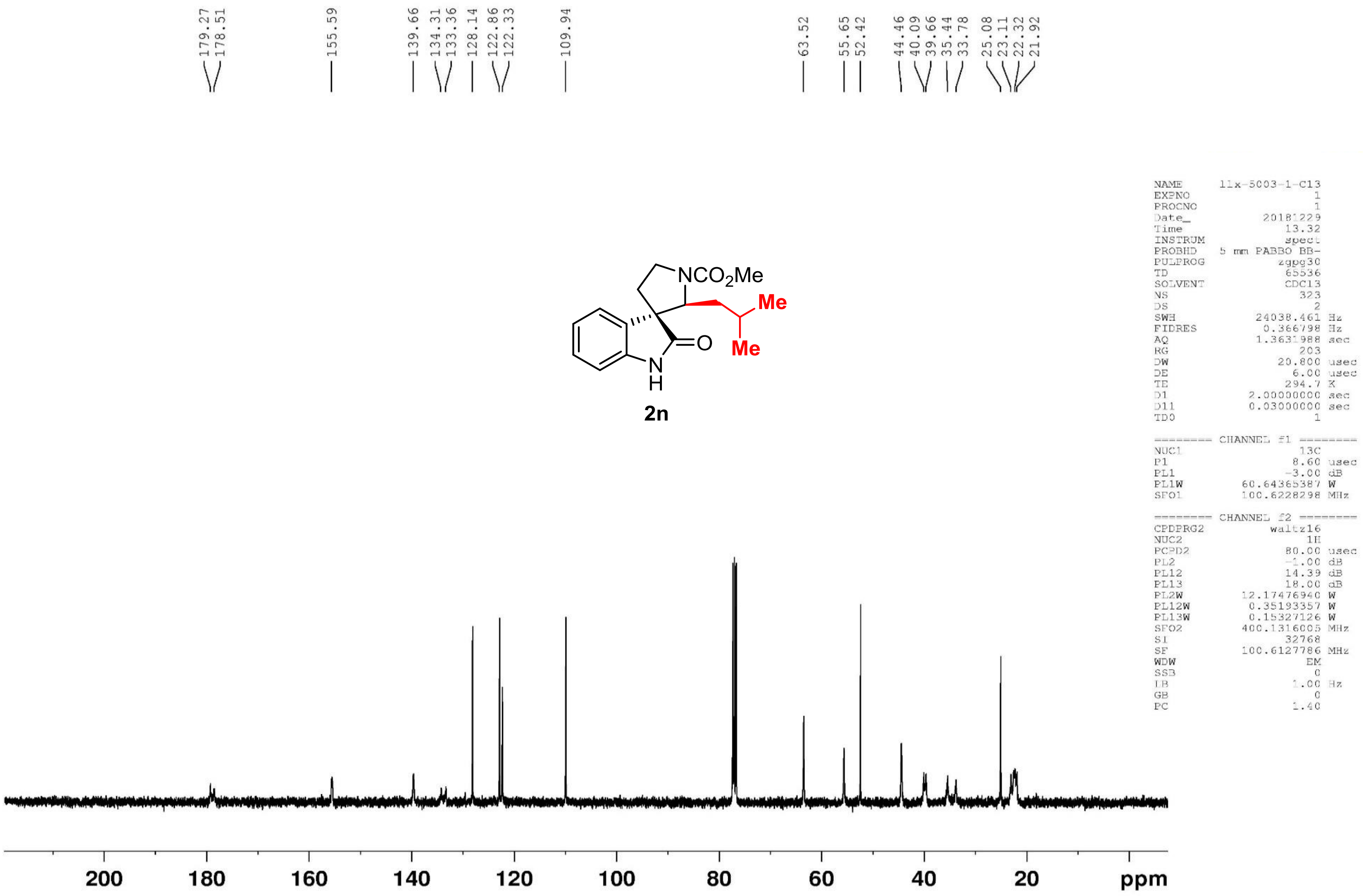

Supplementary Figure 63. ^{13}**C-NMR of 2n**

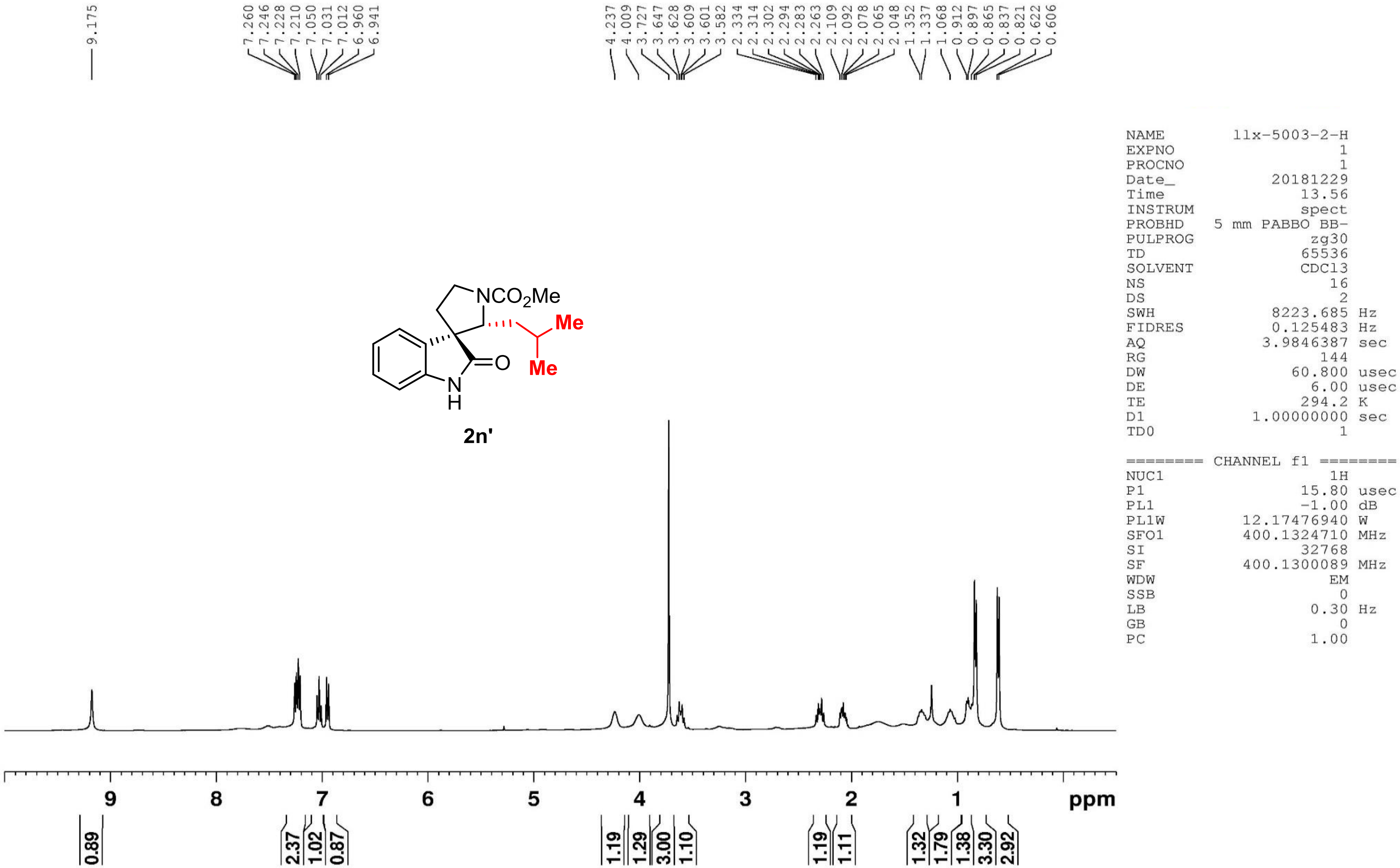

Supplementary Figure 64. ^{1}**H-NMR of 2n'**

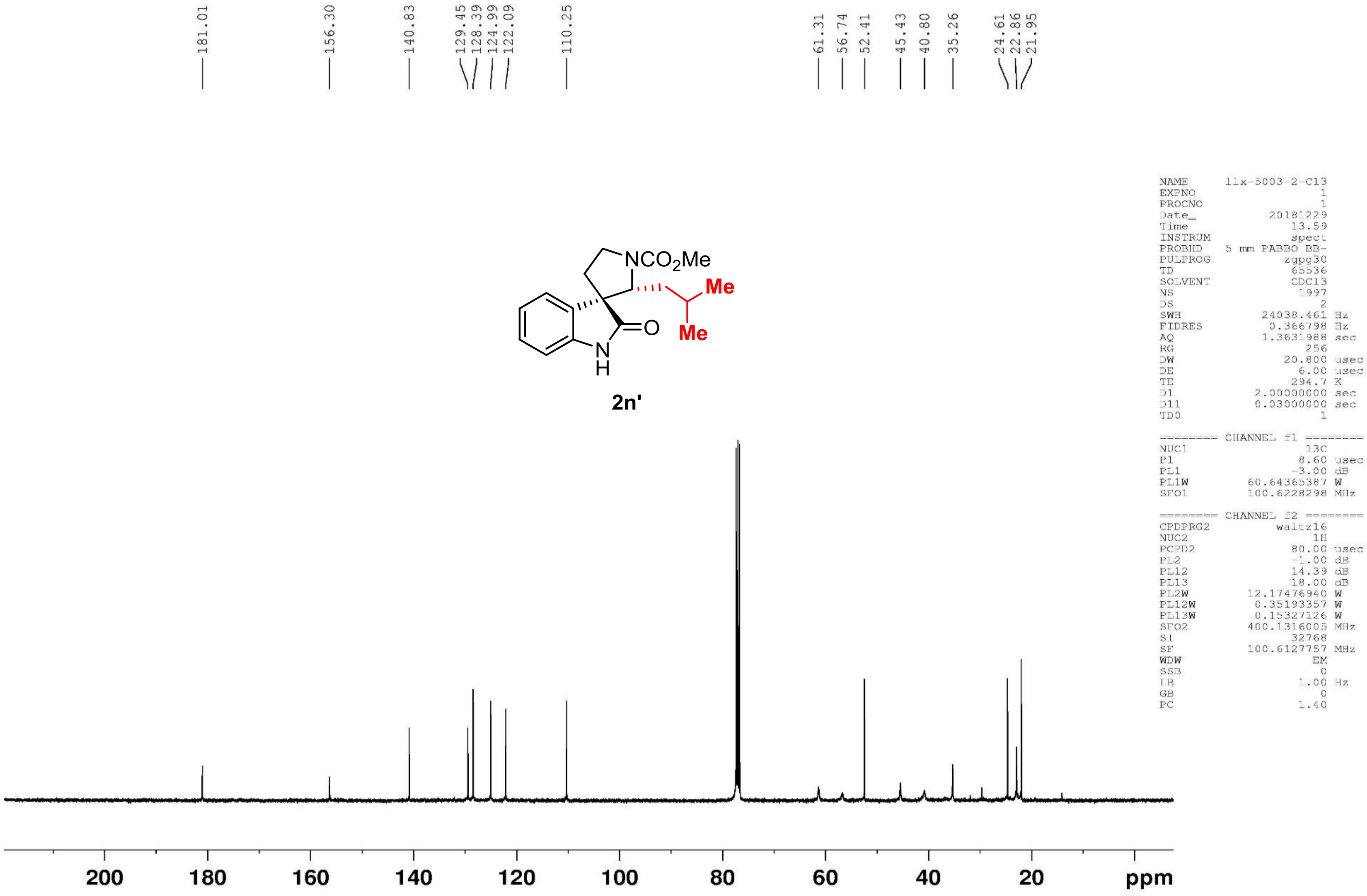

Supplementary Figure 65. ^{13}C-NMR of 2n'

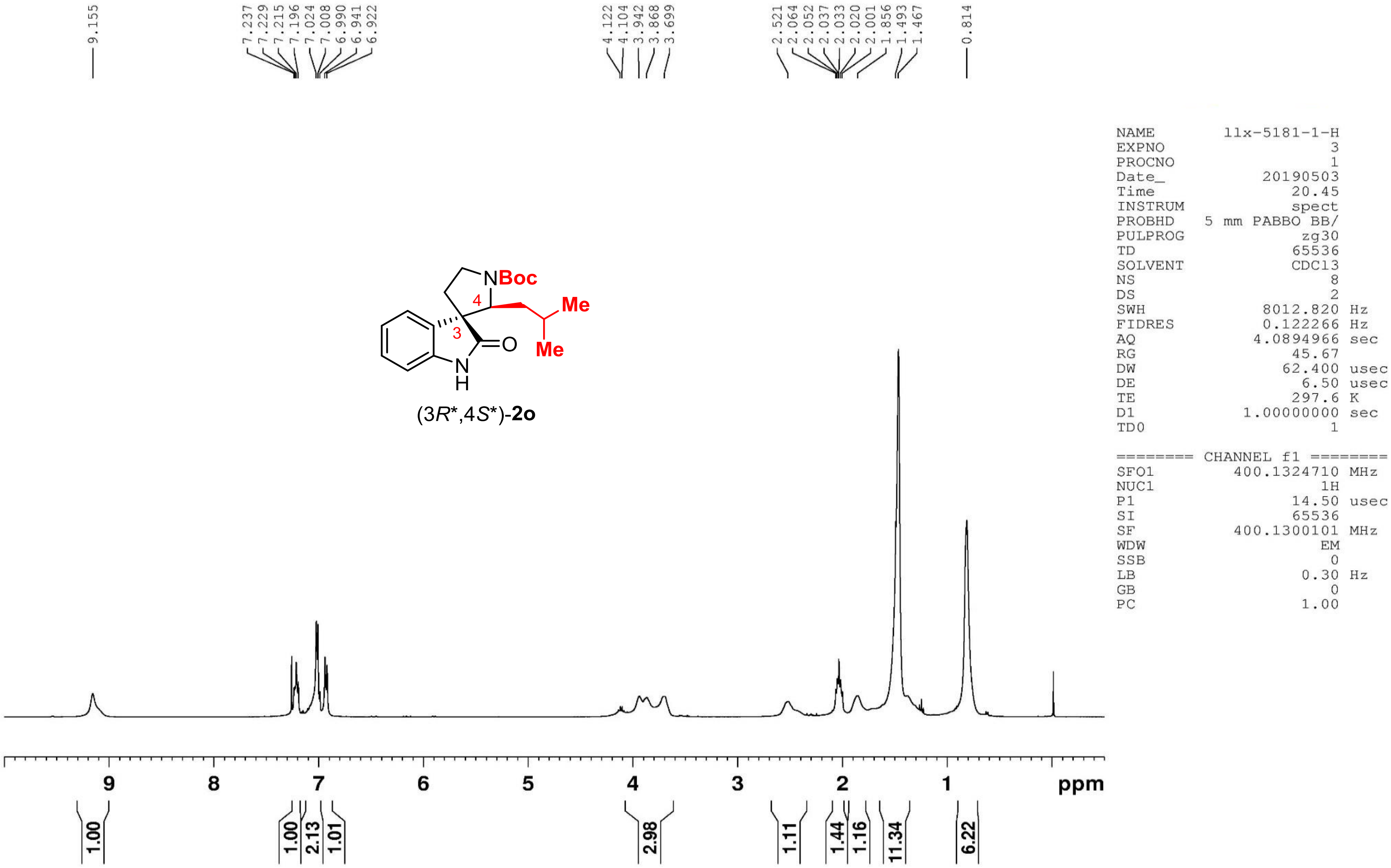

Supplementary Figure 66. ^{1}H-NMR of 2o

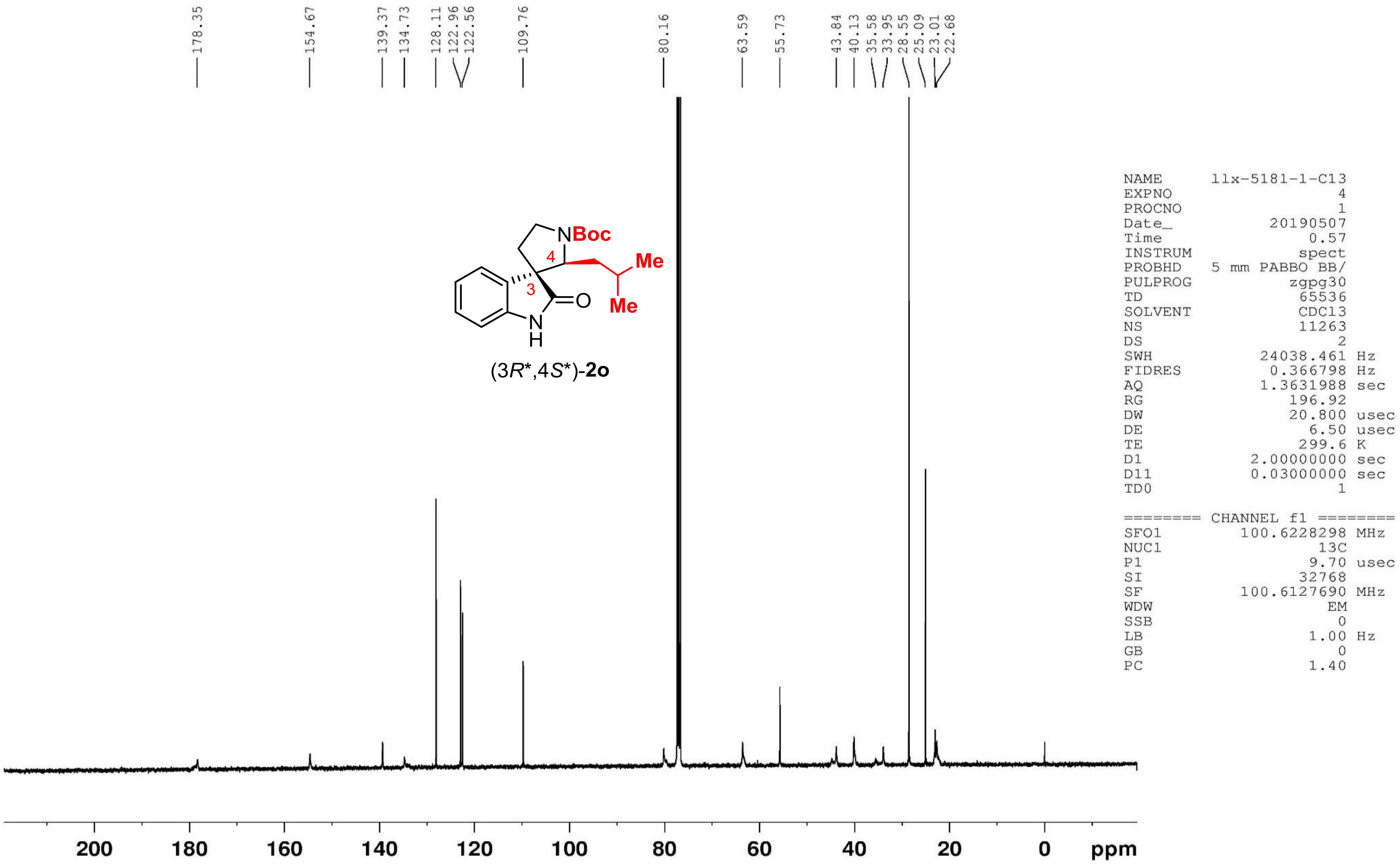

Supplementary Figure 67. ^{13}C-NMR of 2o

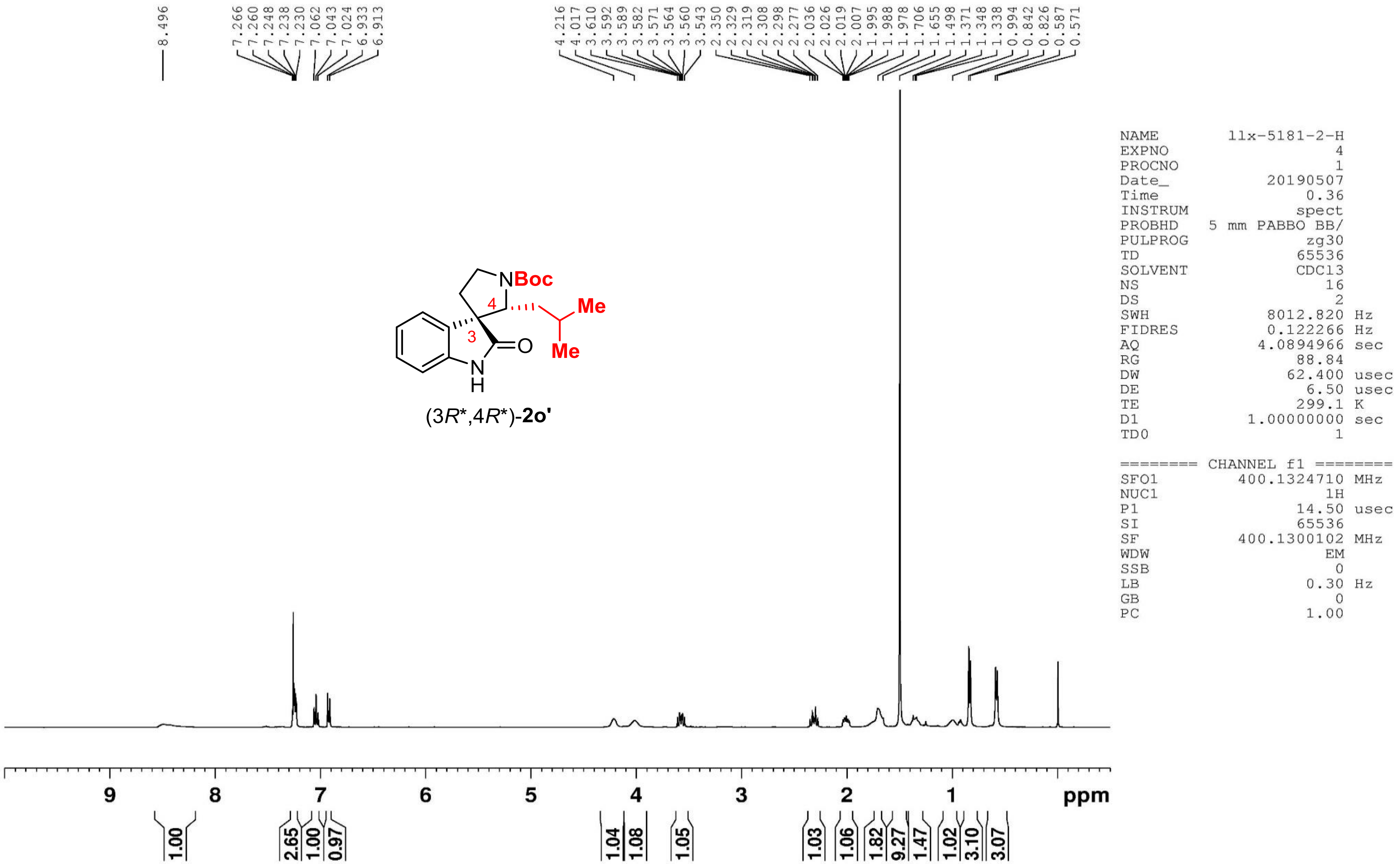

Supplementary Figure 68. ^{1}H-NMR of 2o'

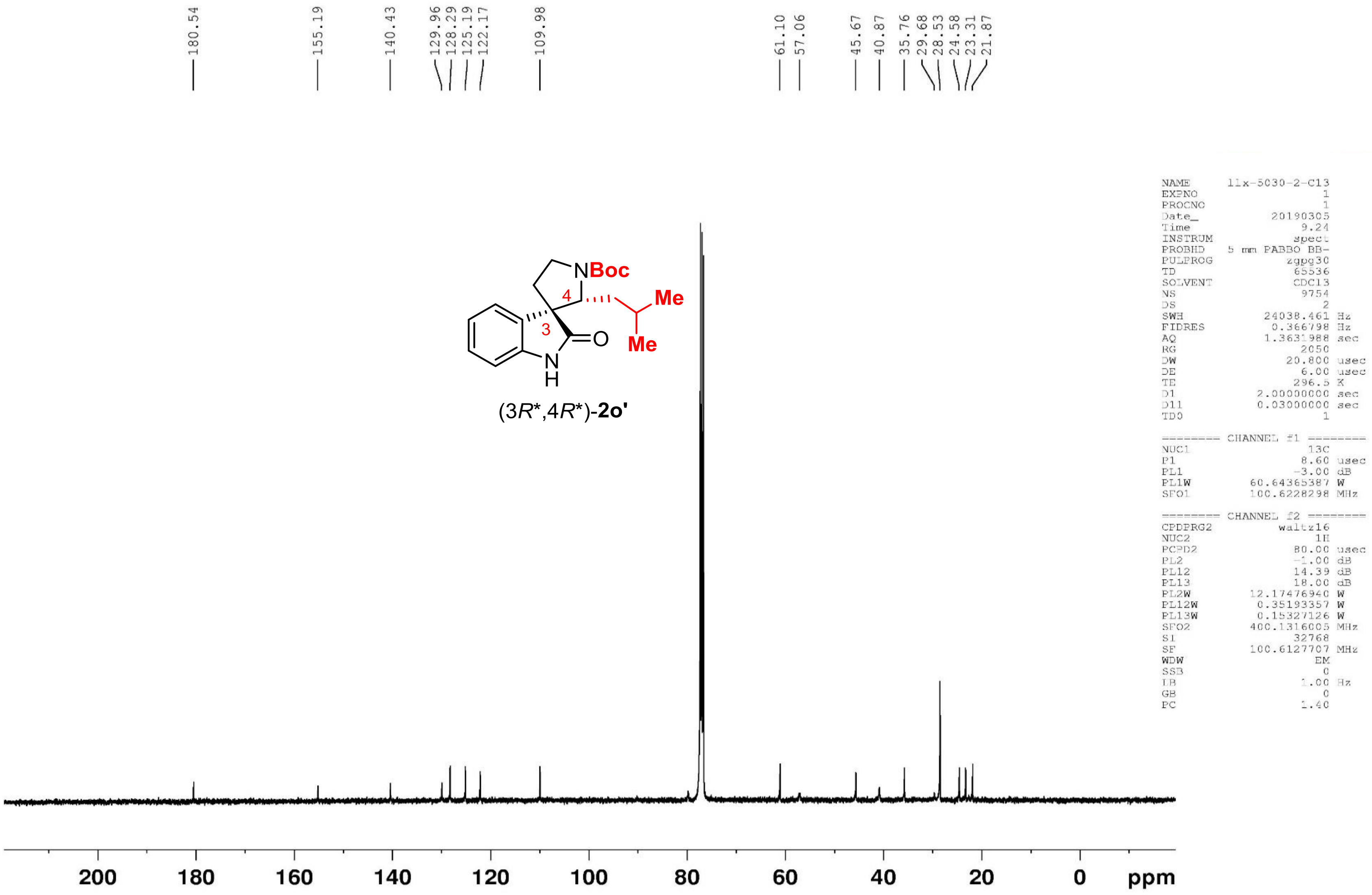

Supplementary Figure 69. ^{13}C-NMR of 2o'

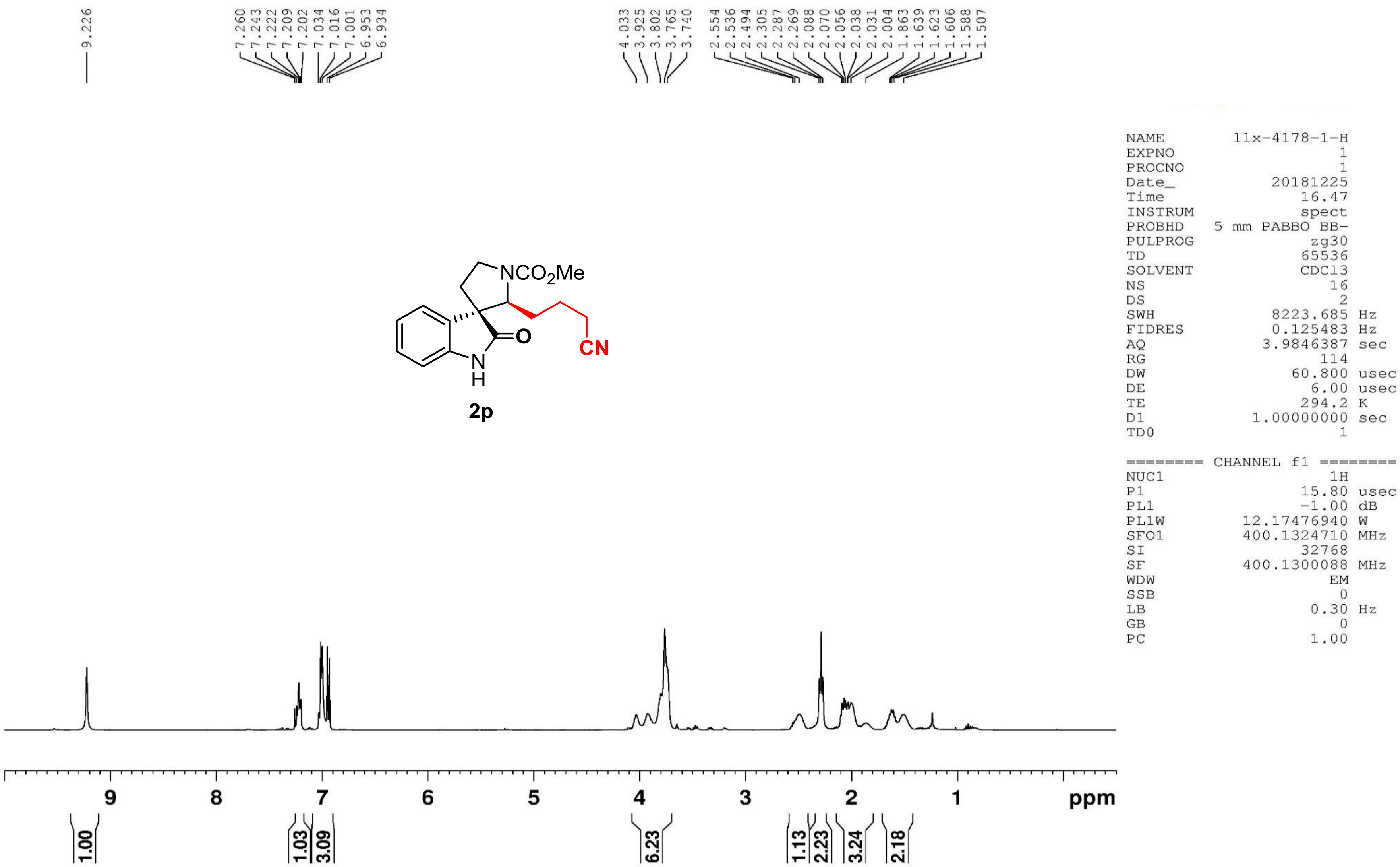

Supplementary Figure 70. ^{1}H-NMR of 2p

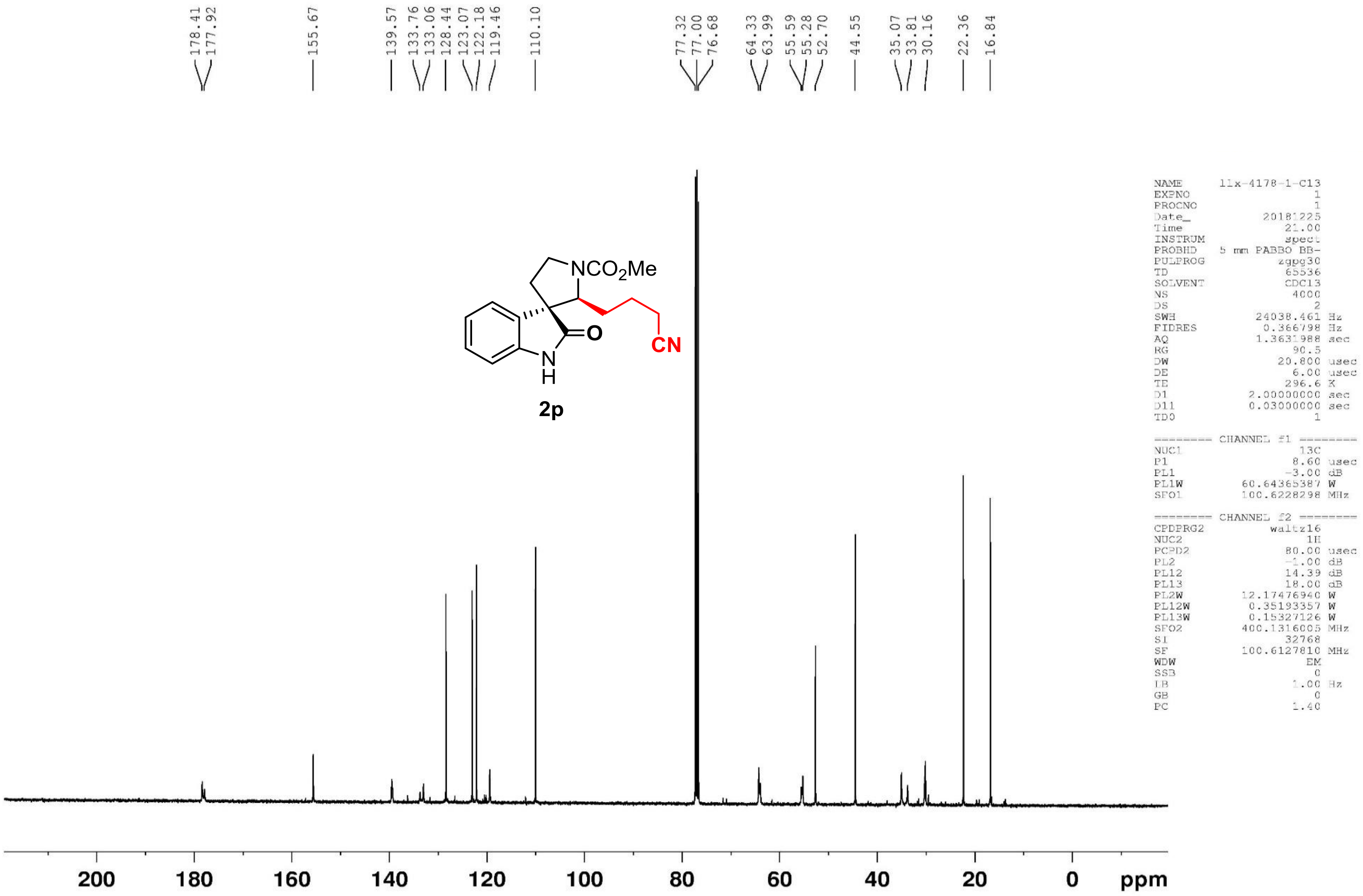

Supplementary Figure 71. ^{13}**C-NMR of 2p**

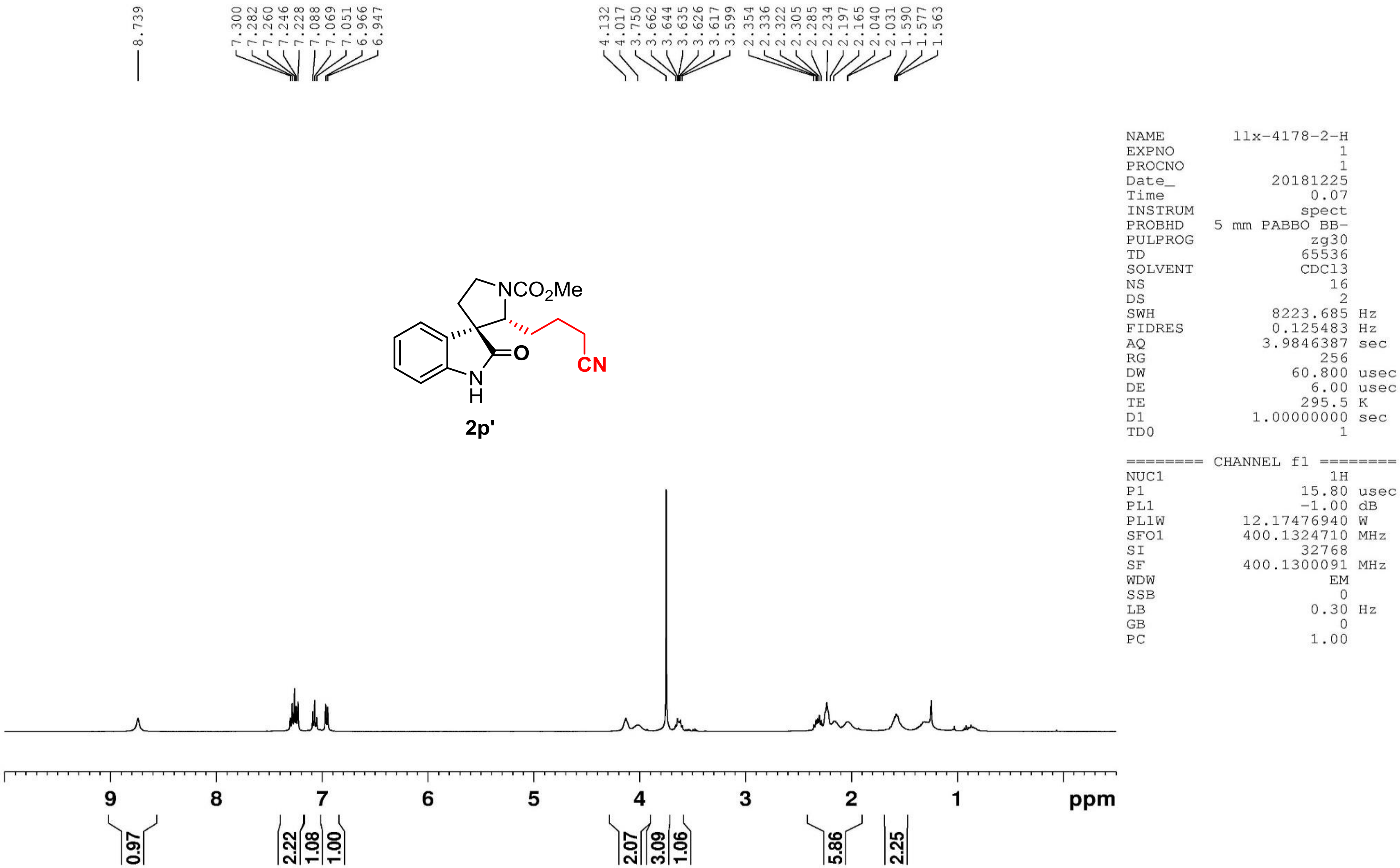

Supplementary Figure 72. ^{1}H-NMR of 2p'

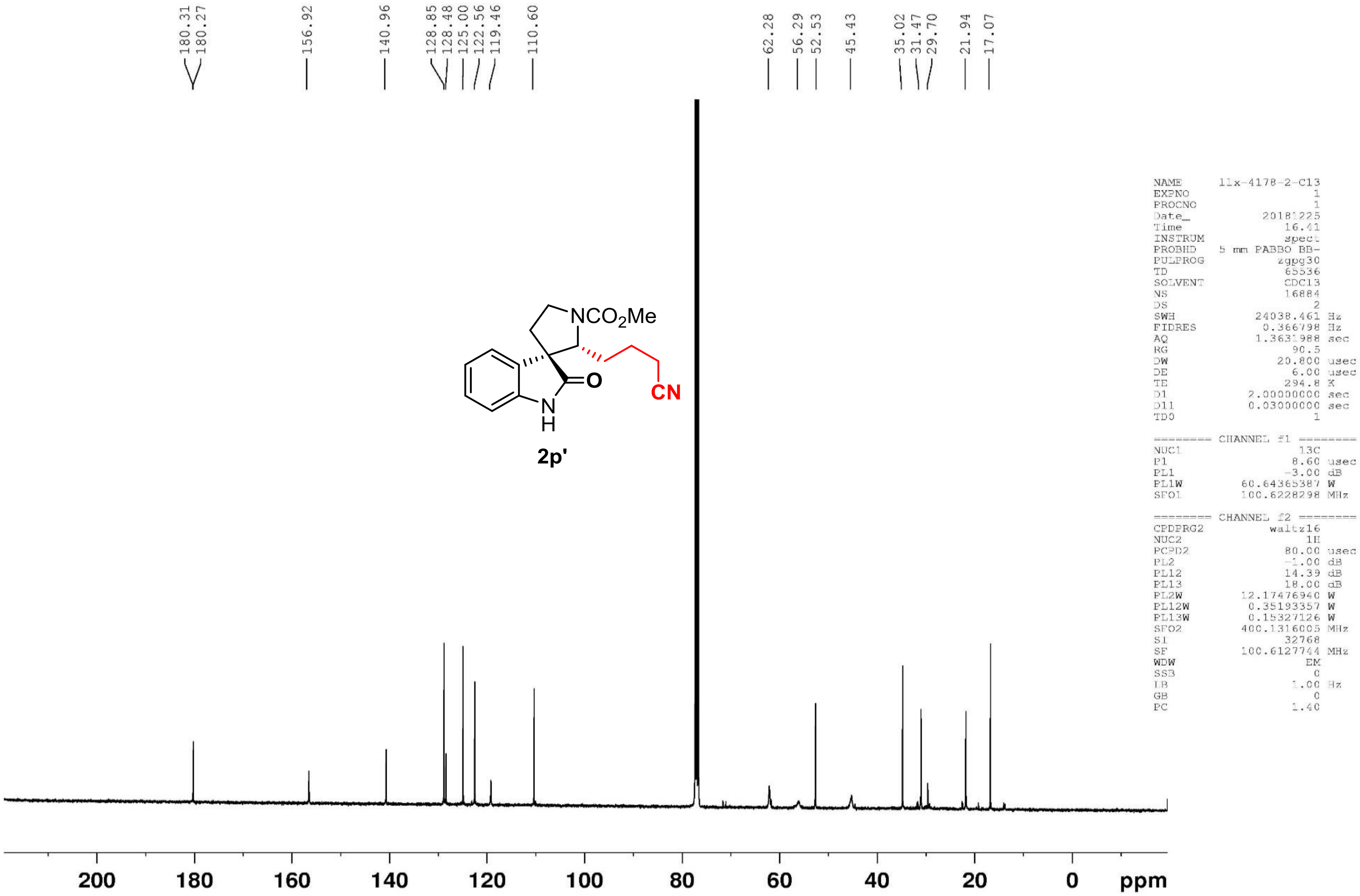

Supplementary Figure 73. ^{13}**C-NMR of 2p'**

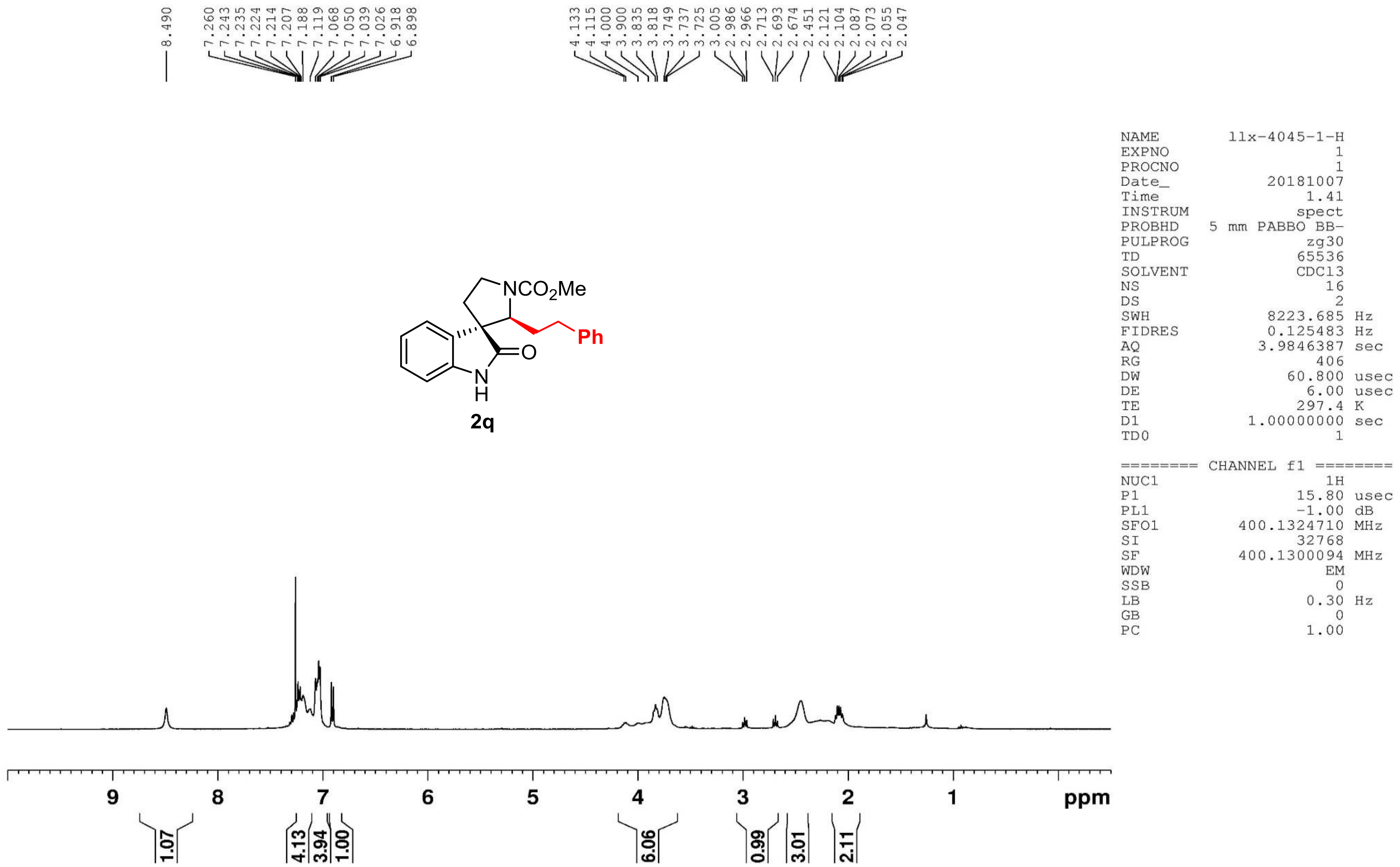

Supplementary Figure 74. ^{1}H-NMR of 2q

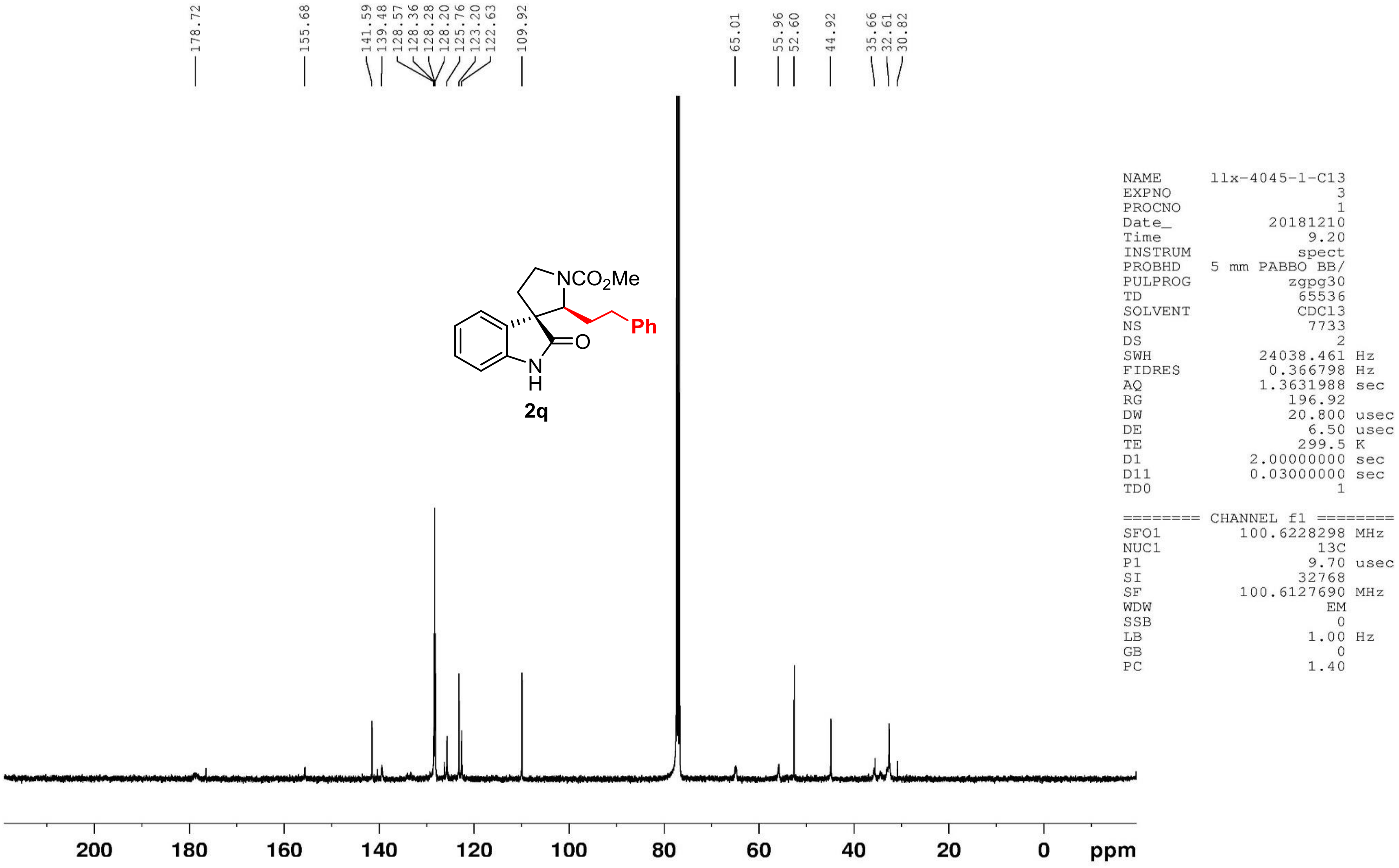

Supplementary Figure 75. ^{13}C-NMR of 2q

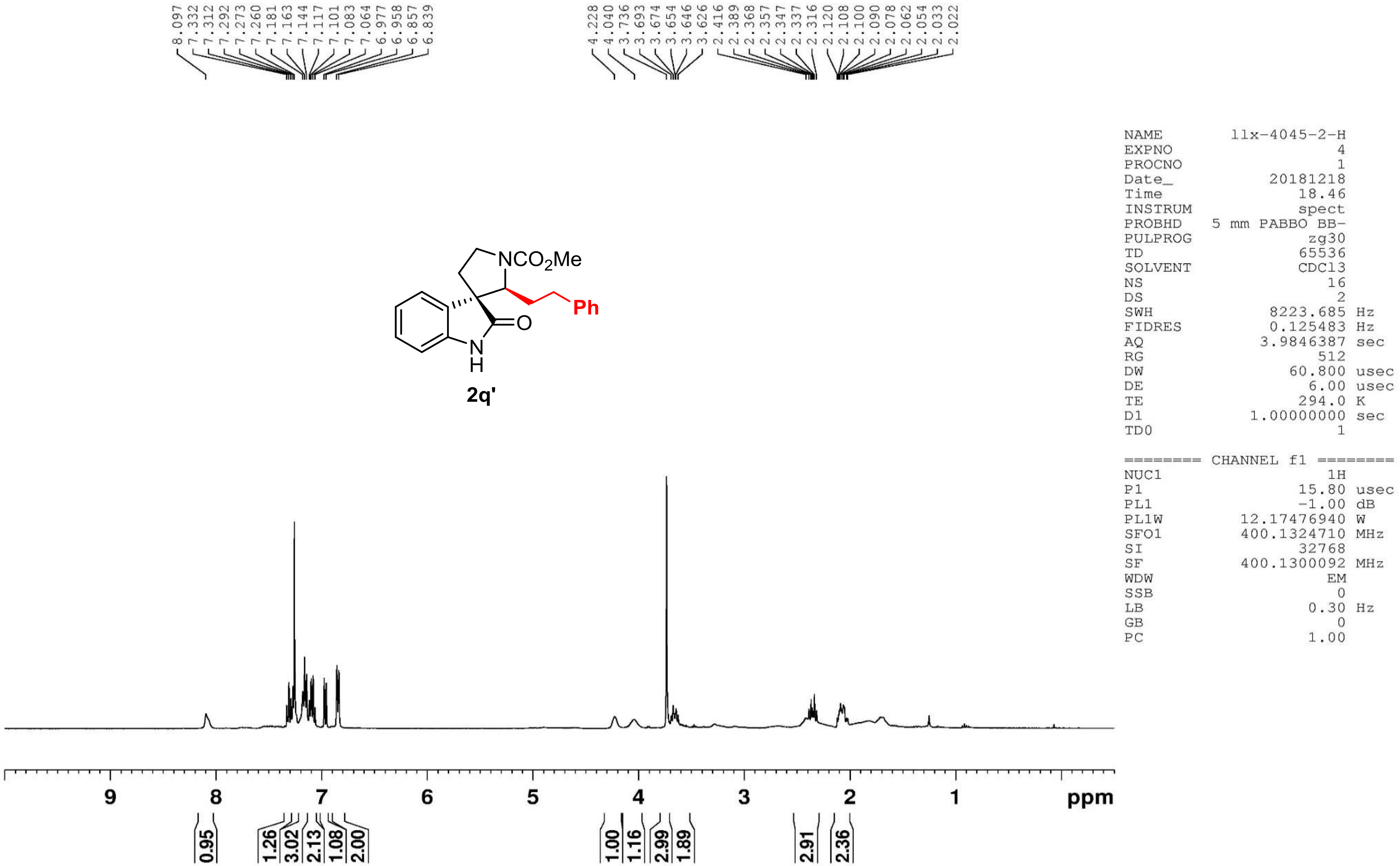

Supplementary Figure 76. ^{1}H-NMR of 2q'

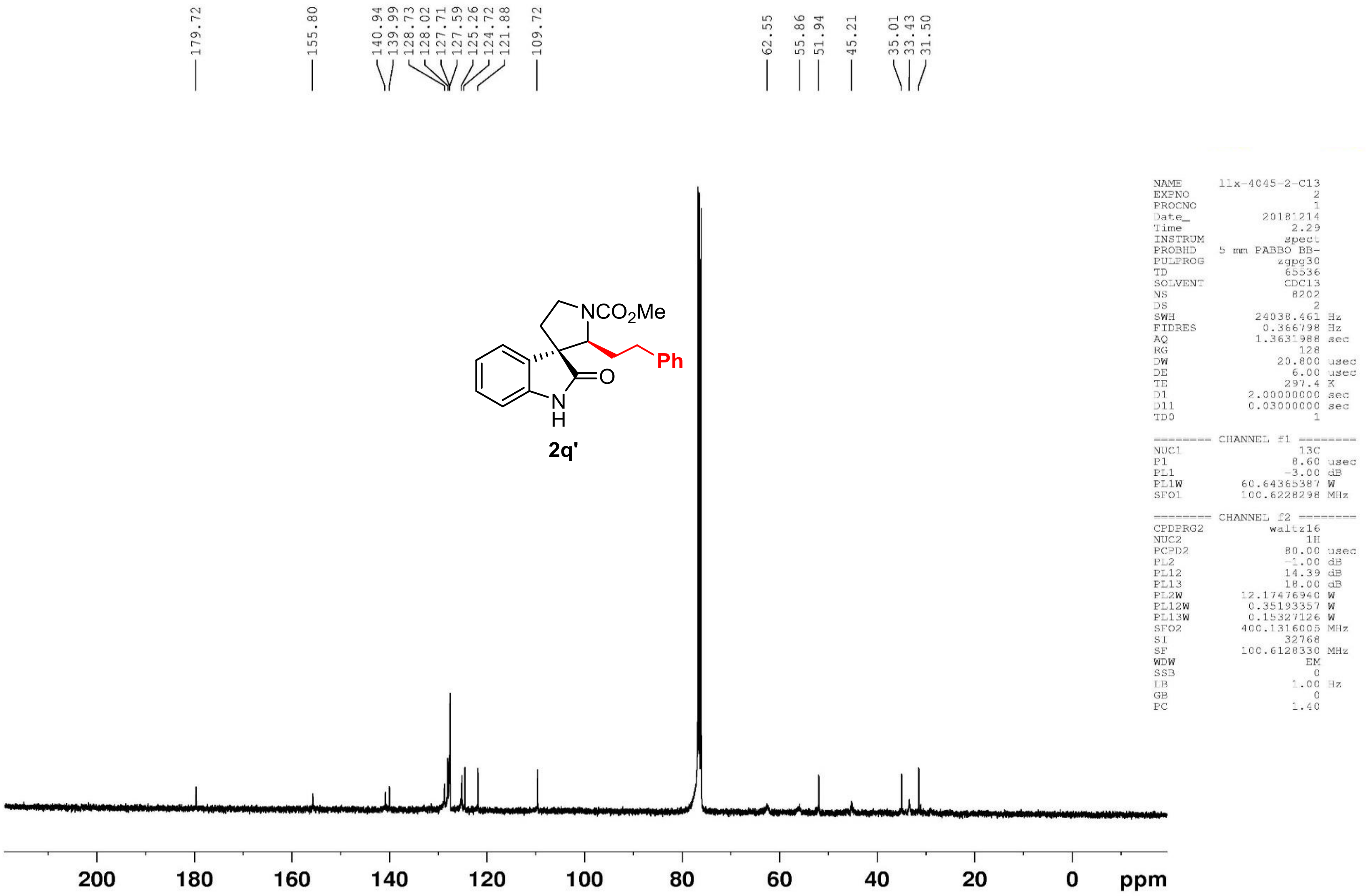

Supplementary Figure 77. ^{13}**C-NMR of 2q'**

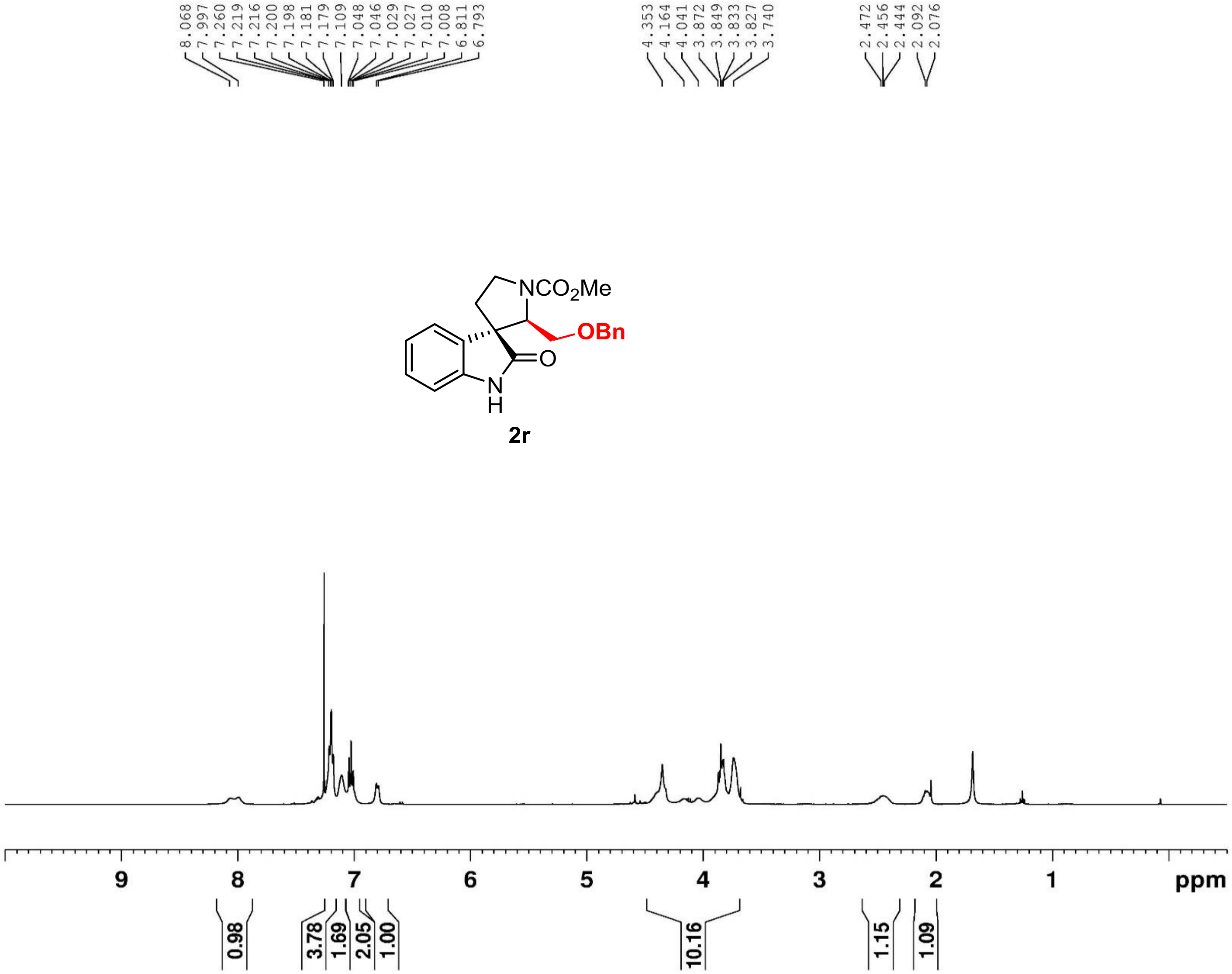

2r

```
NAME       llx-4078-1-H-purified
EXPNO                   1
PROCNO                  1
Date_            20181027
Time                19.42
INSTRUM             spect
PROBHD     5 mm PABBO BB/
PULPROG              zg30
TD                  65536
SOLVENT             CDCl3
NS                     16
DS                      2
SWH              8012.820 Hz
FIDRES           0.122266 Hz
AQ              4.0894966 sec
RG                 112.31
DW                 62.400 usec
DE                   6.50 usec
TE                  298.2 K
D1             1.00000000 sec
TD0                     1

======== CHANNEL f1 ========
SFO1          400.1324710 MHz
NUC1                   1H
P1                  14.50 usec
SI                  65536
SF            400.1300101 MHz
WDW                    EM
SSB                     0
LB                   0.30 Hz
GB                      0
PC                   1.00
```

Supplementary Figure 78. ^{1}H-NMR of 2r

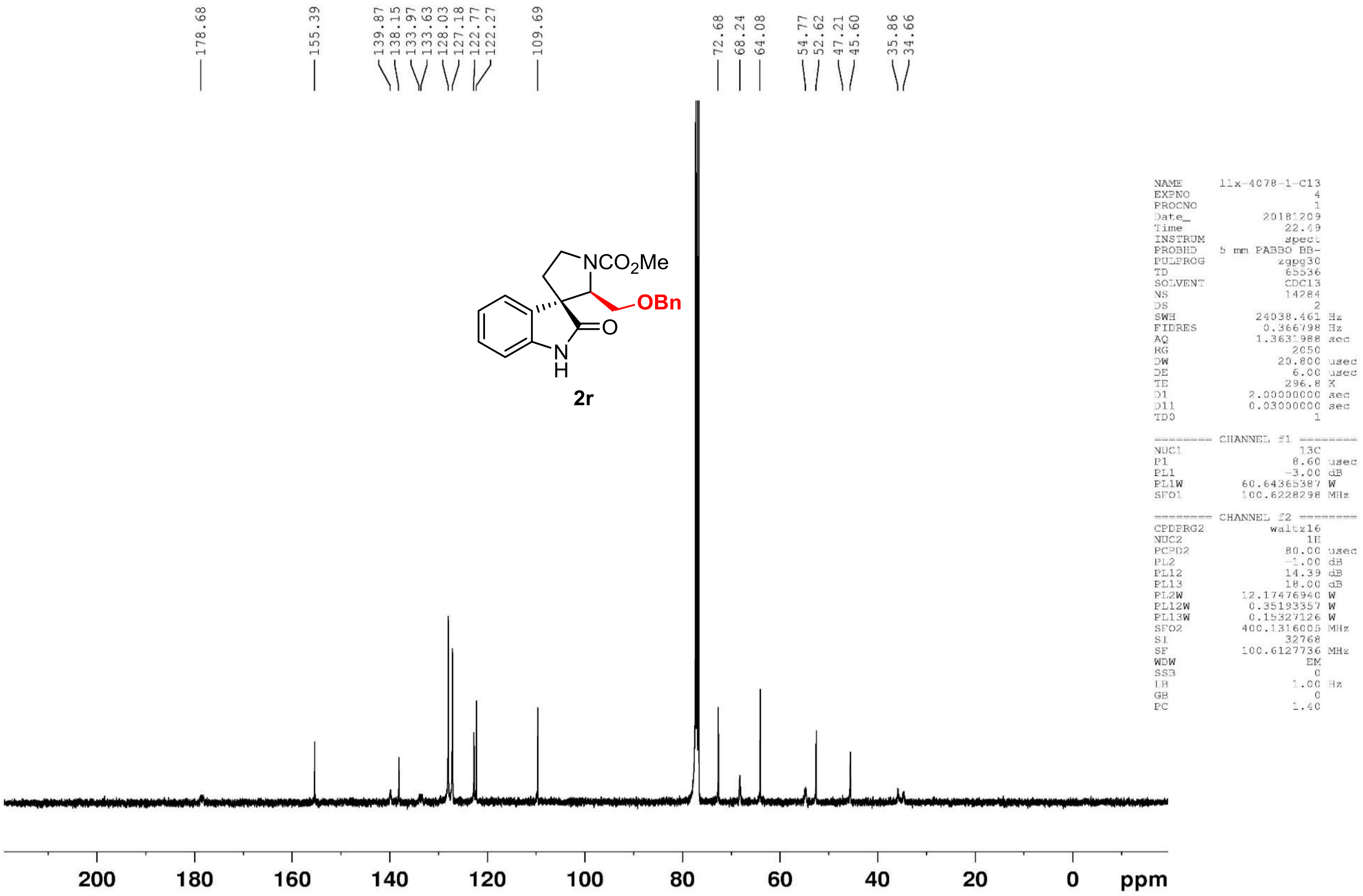

Supplementary Figure 79. ^{13}C-NMR of 2r

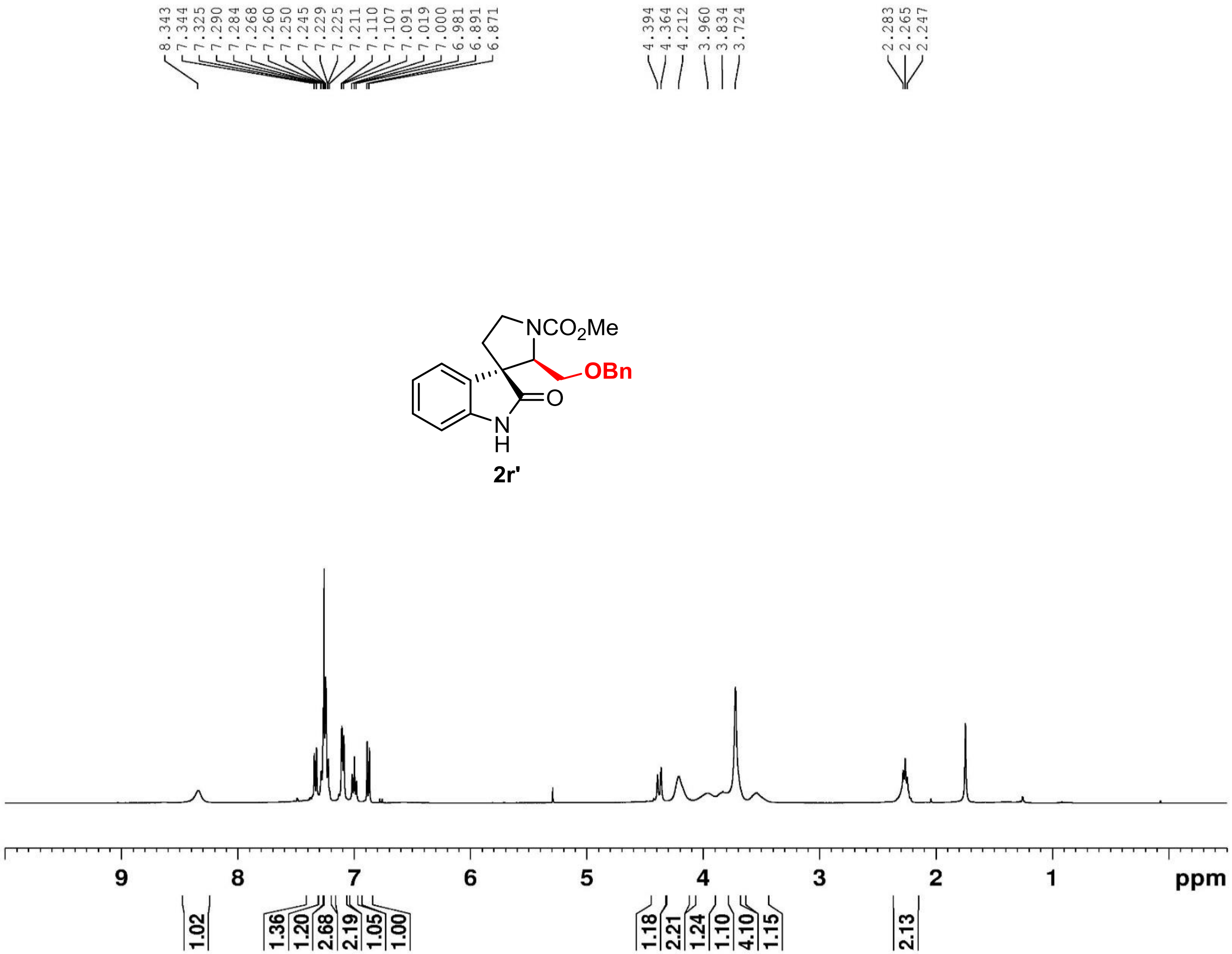

NAME	llx-4078-2-H
EXPNO	1
PROCNO	1
Date_	20181027
Time	14.06
INSTRUM	spect
PROBHD	5 mm PABBO BB/
PULPROG	zg30
TD	65536
SOLVENT	CDCl3
NS	16
DS	2
SWH	8012.820 Hz
FIDRES	0.122266 Hz
AQ	4.0894966 sec
RG	103.52
DW	62.400 usec
DE	6.50 usec
TE	298.1 K
D1	1.00000000 sec
TD0	1

======== CHANNEL f1 ========

SFO1	400.1324710 MHz
NUC1	1H
P1	14.50 usec
SI	65536
SF	400.1300102 MHz
WDW	EM
SSB	0
LB	0.30 Hz
GB	0
PC	1.00

Supplementary Figure 80. ^{1}H-NMR of 2r'

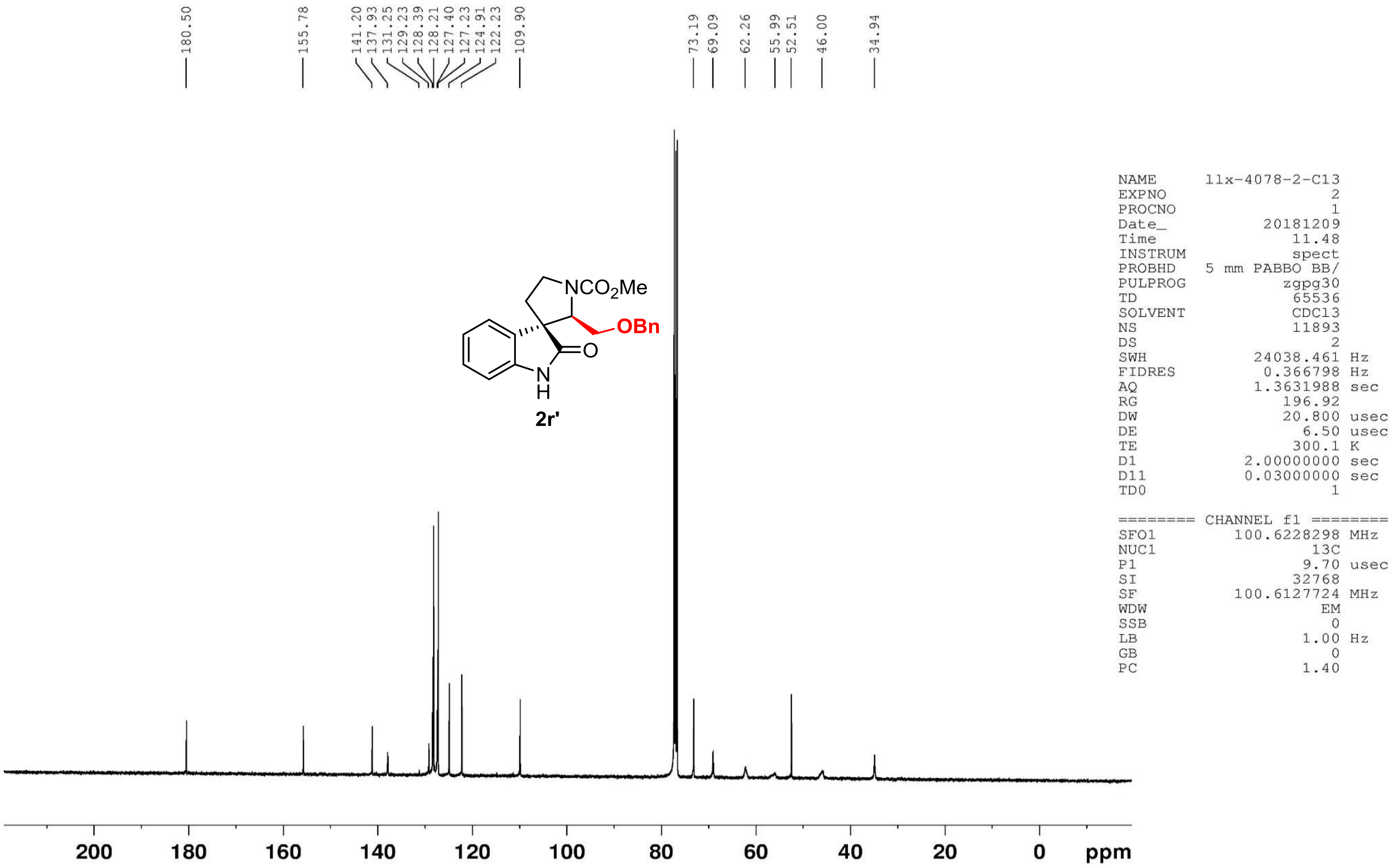

Supplementary Figure 81. ^{13}C-NMR of 2r'

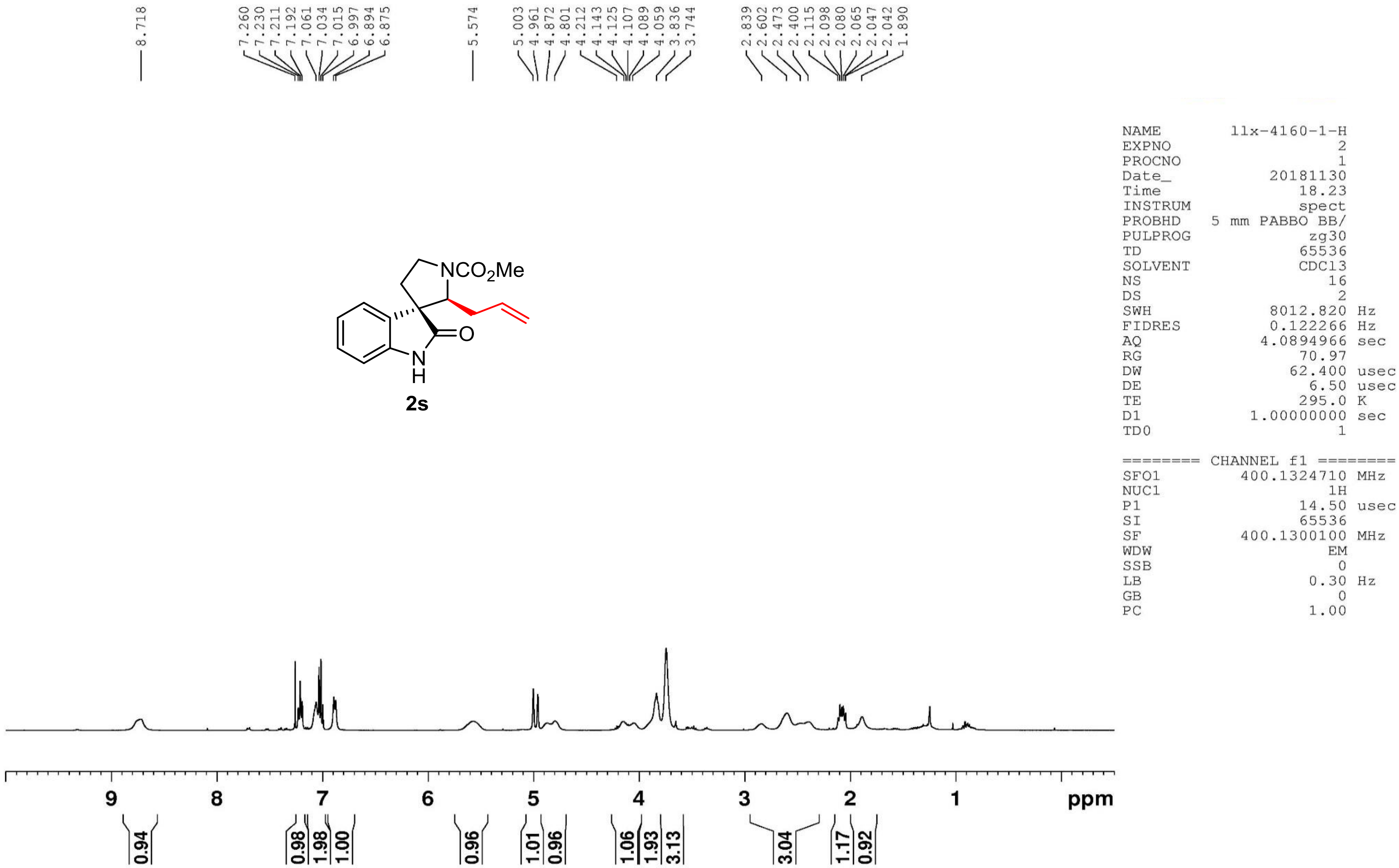

Supplementary Figure 82. ¹H-NMR of 2s

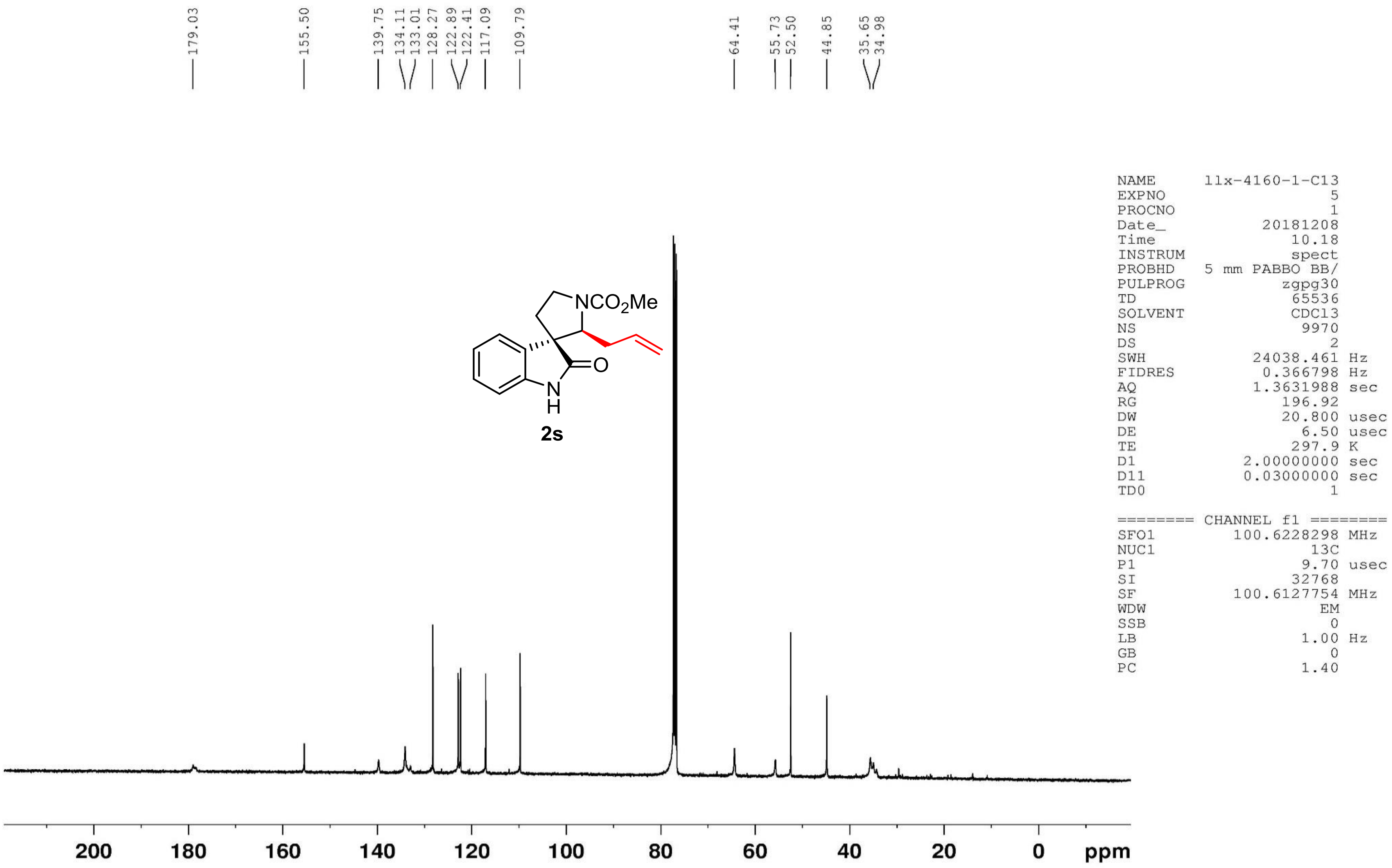

Supplementary Figure 83. ^{13}**C-NMR of 2s**

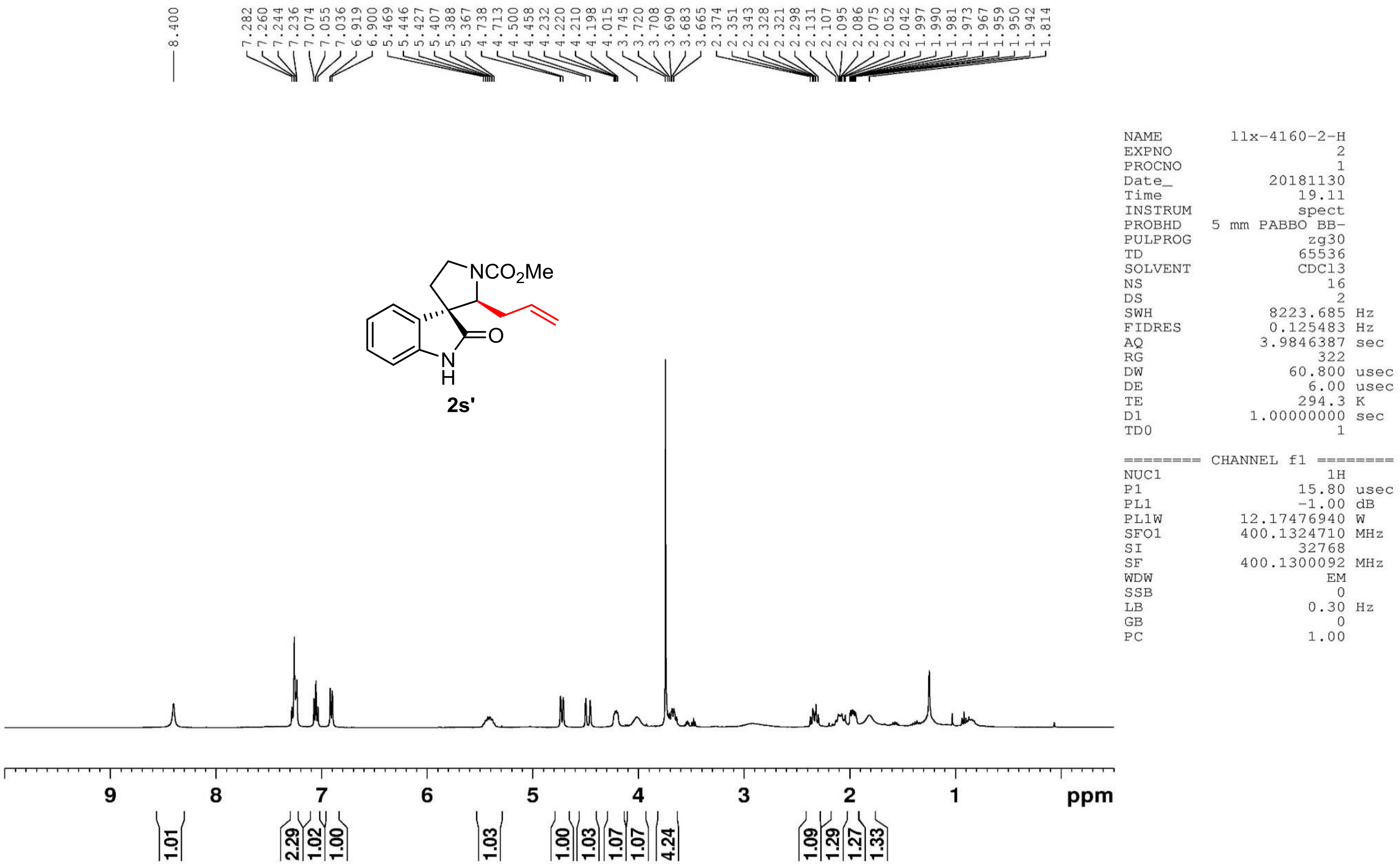

Supplementary Figure 84. ^{1}H-NMR of 2s'

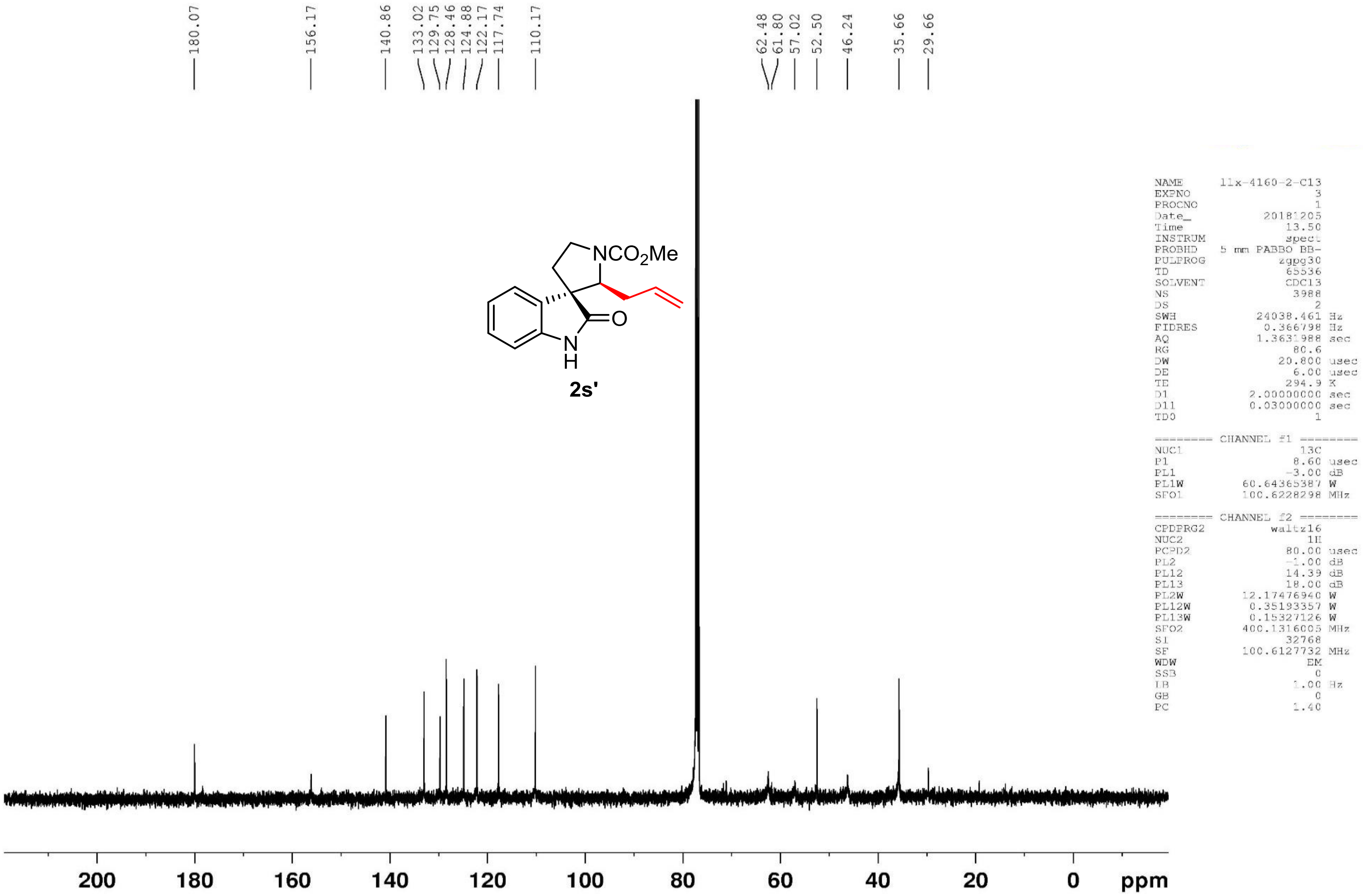

Supplementary Figure 85. ^{13}C-NMR of 2s'

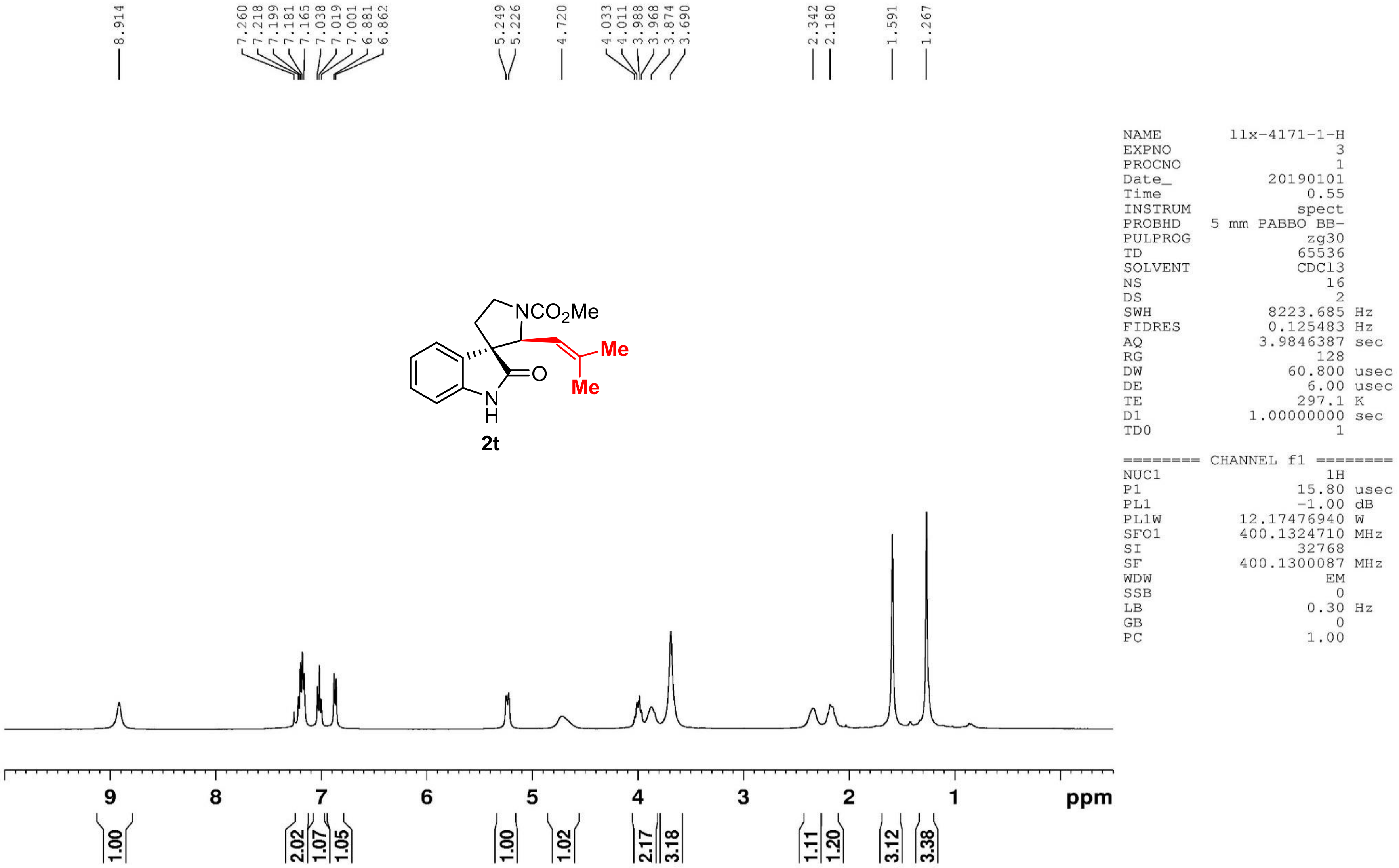

Supplementary Figure 86. ^{1}**H-NMR of 2t**

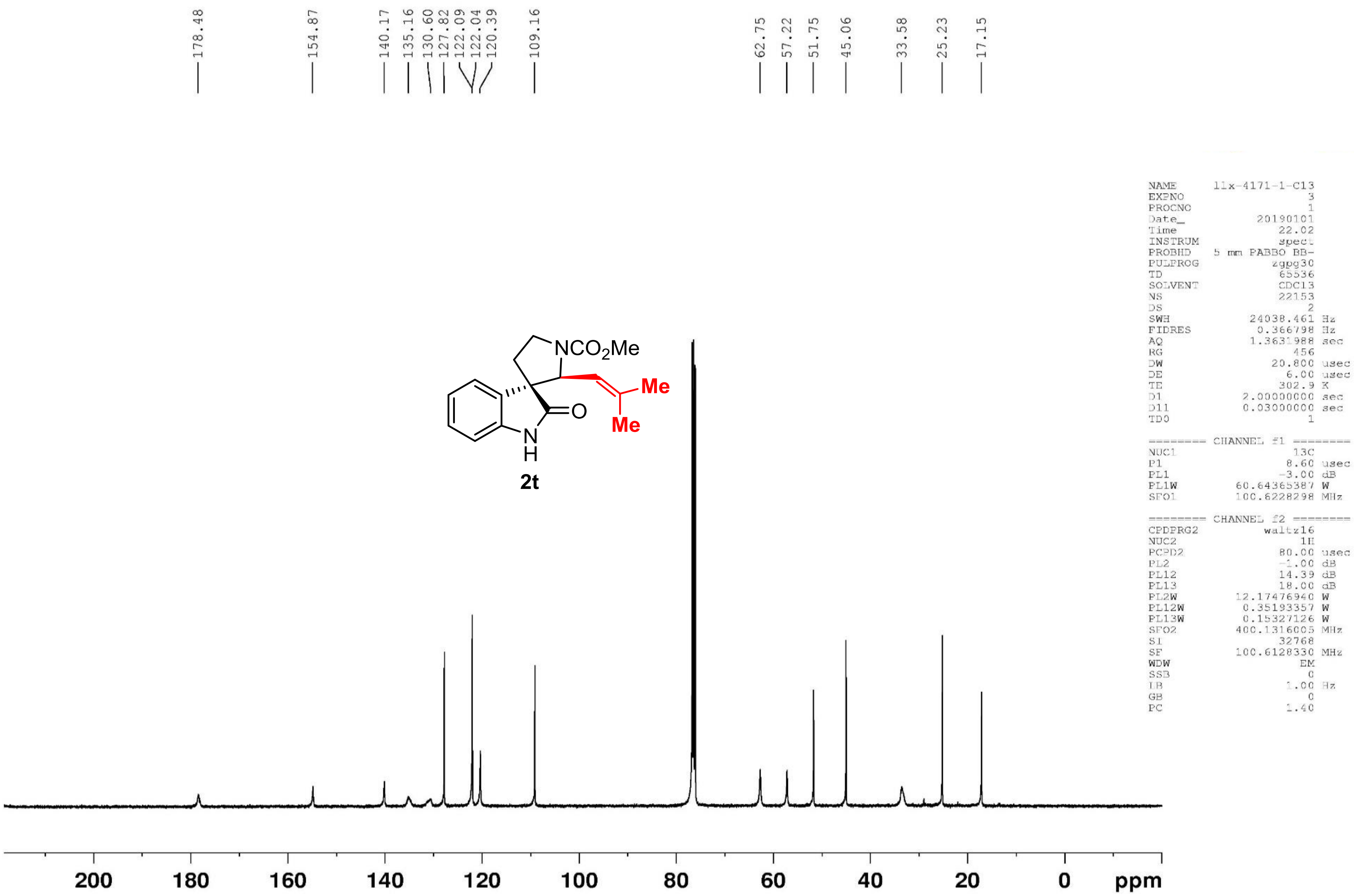

Supplementary Figure 87. ^{13}C-NMR of 2t

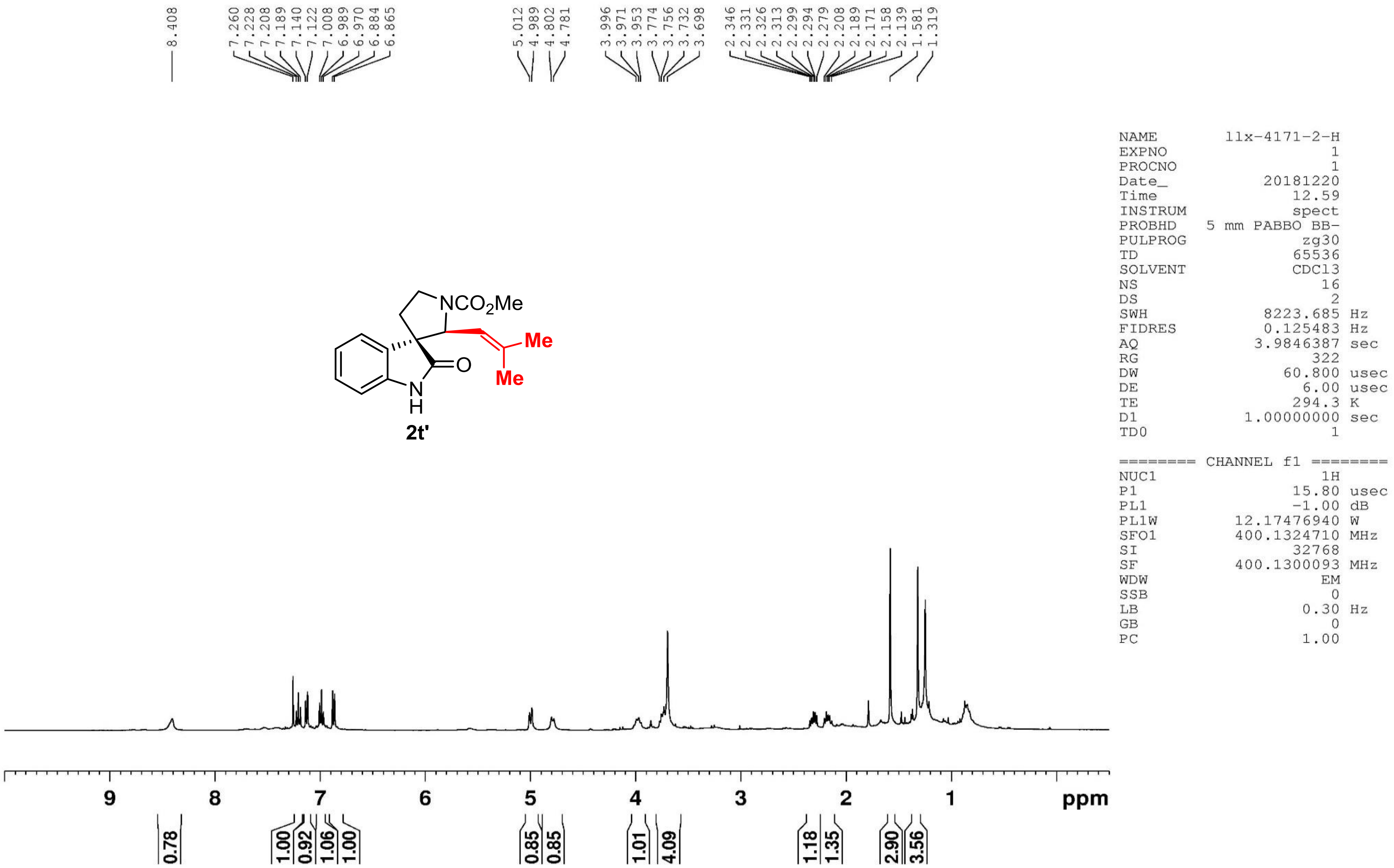

Supplementary Figure 88. ^{1}H-NMR of 2t'

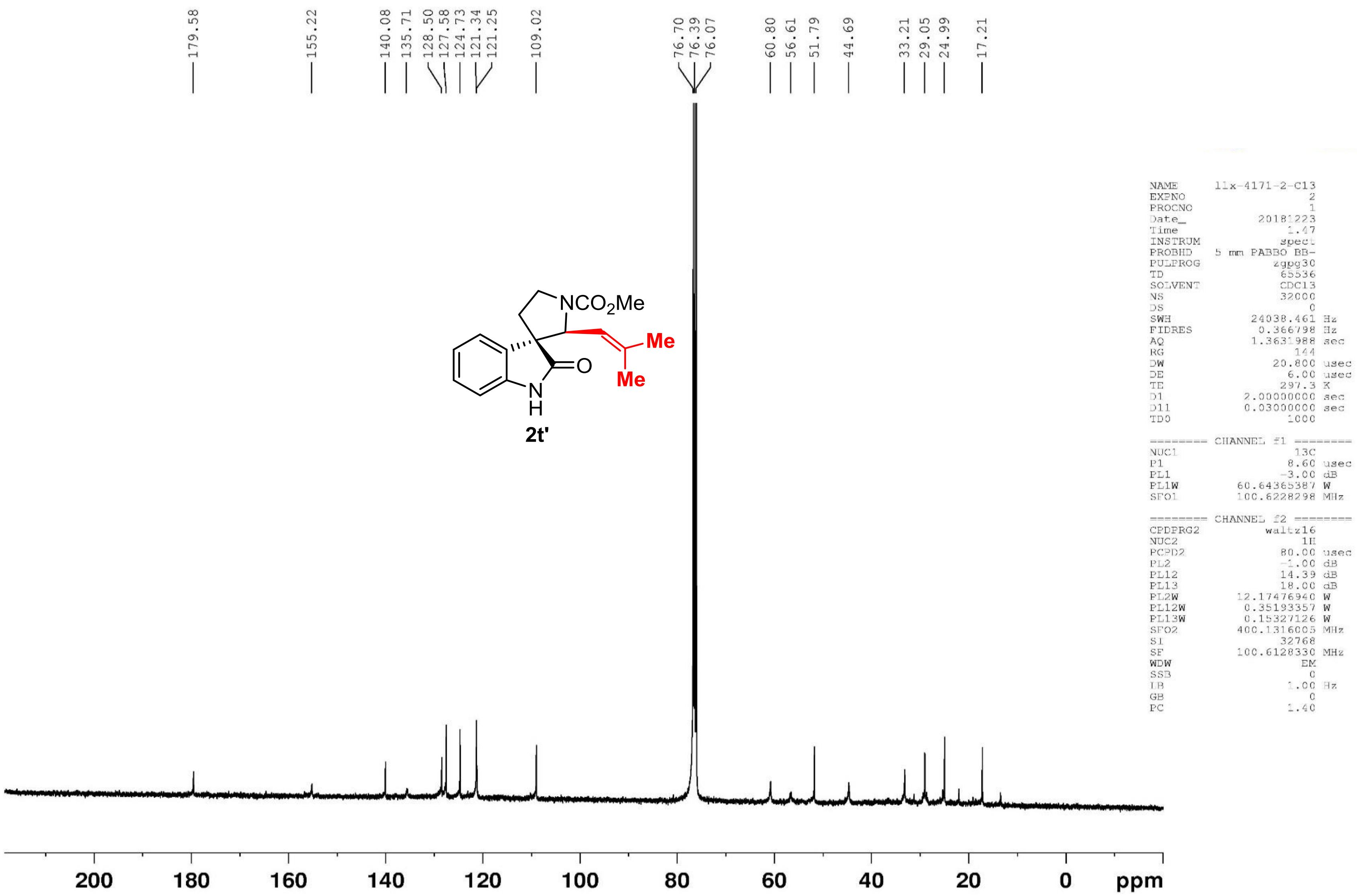

Supplementary Figure 89. ^{13}C-NMR of 2t'

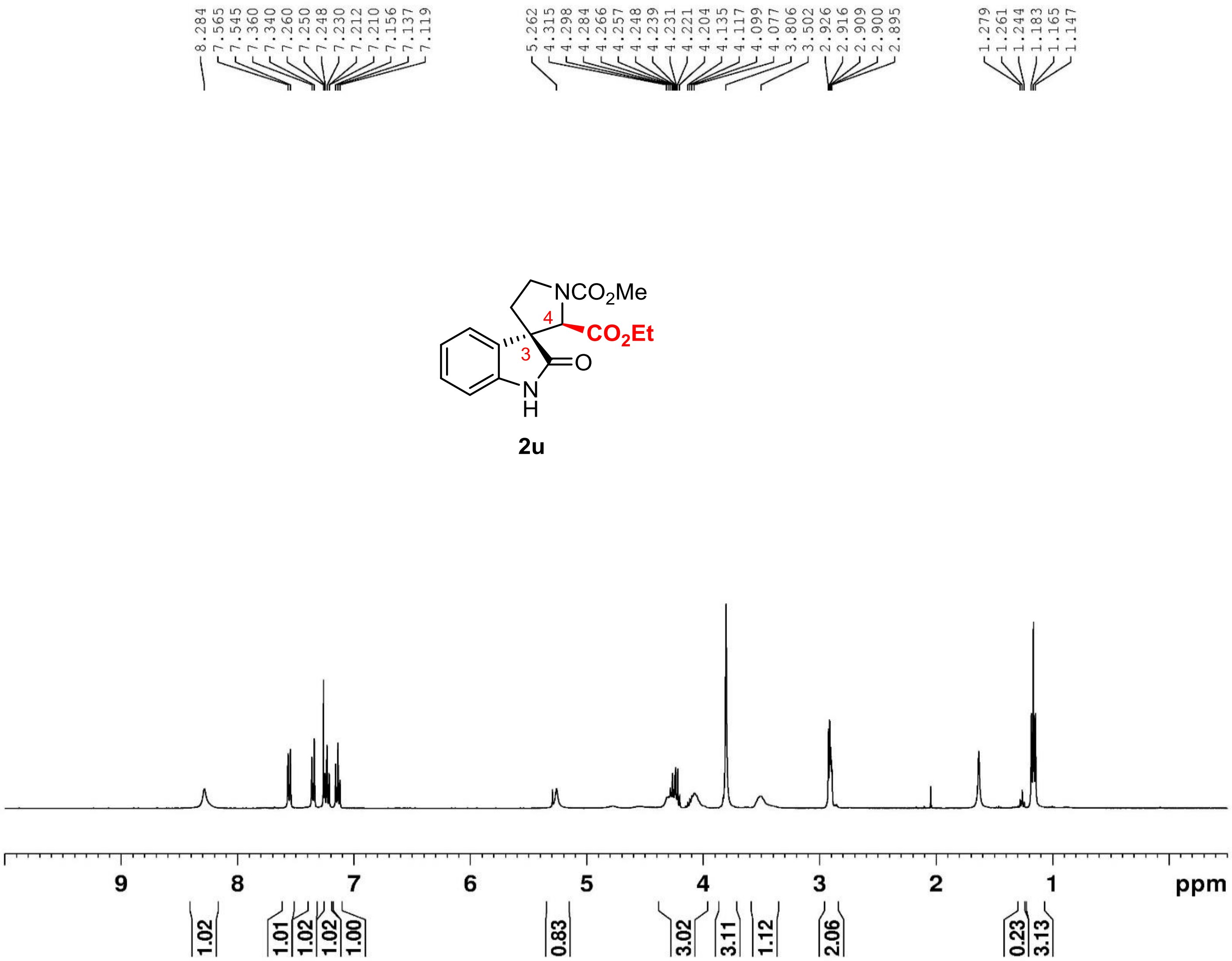

NAME	llx-4164-2-H
EXPNO	1
PROCNO	1
Date_	20181209
Time	1.33
INSTRUM	spect
PROBHD	5 mm PABBO BB-
PULPROG	zg30
TD	65536
SOLVENT	CDCl3
NS	16
DS	2
SWH	8223.685 Hz
FIDRES	0.125483 Hz
AQ	3.9846387 sec
RG	456
DW	60.800 usec
DE	6.00 usec
TE	300.5 K
D1	1.00000000 sec
TD0	1

======== CHANNEL f1 ========

NUC1	1H
P1	15.80 usec
PL1	-1.00 dB
PL1W	12.17476940 W
SFO1	400.1324710 MHz
SI	32768
SF	400.1300091 MHz
WDW	EM
SSB	0
LB	0.30 Hz
GB	0
PC	1.00

Supplementary Figure 90. ^{1}H-NMR of 2u

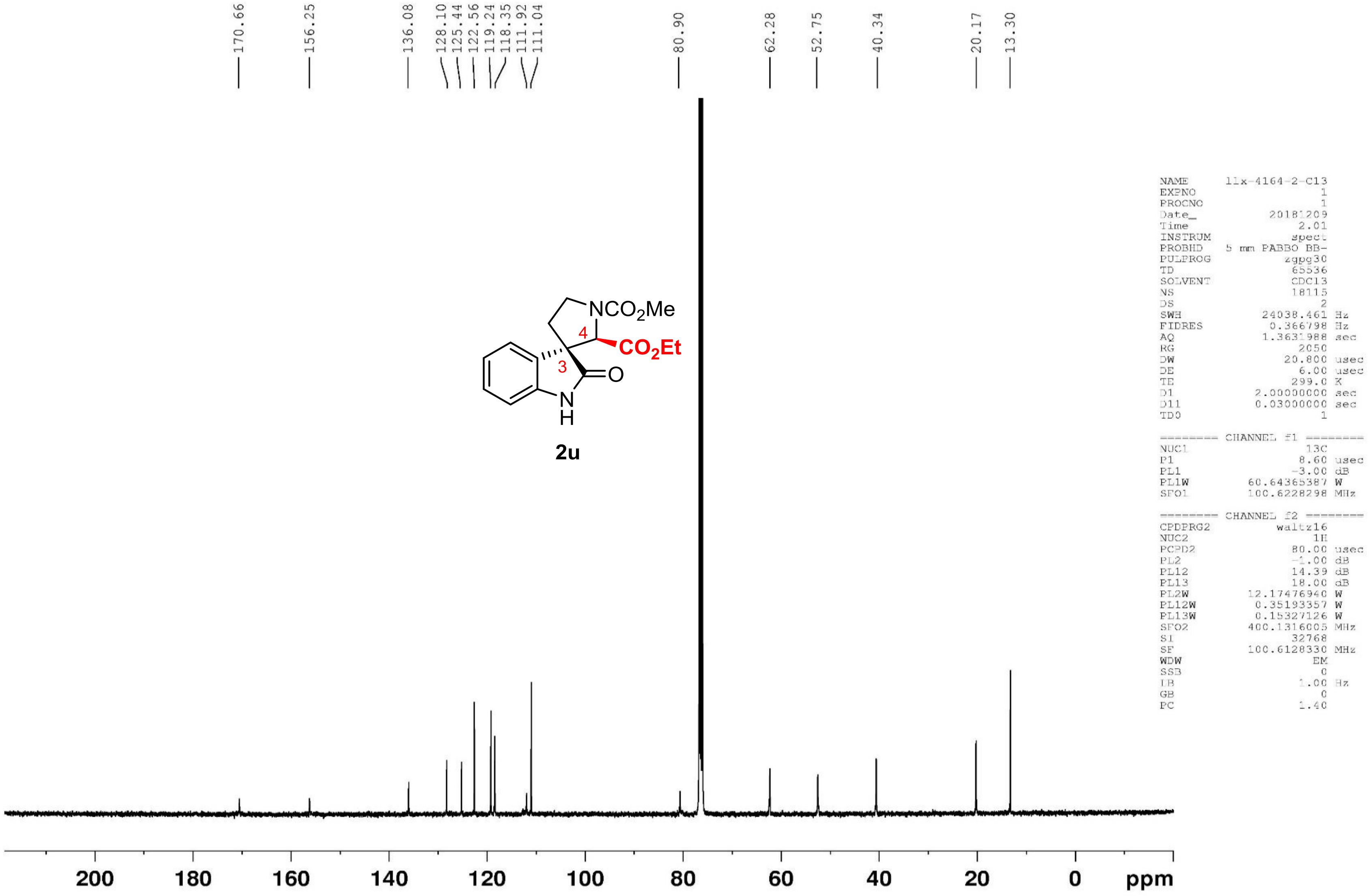

Supplementary Figure 91. ^{13}C-NMR of 2u

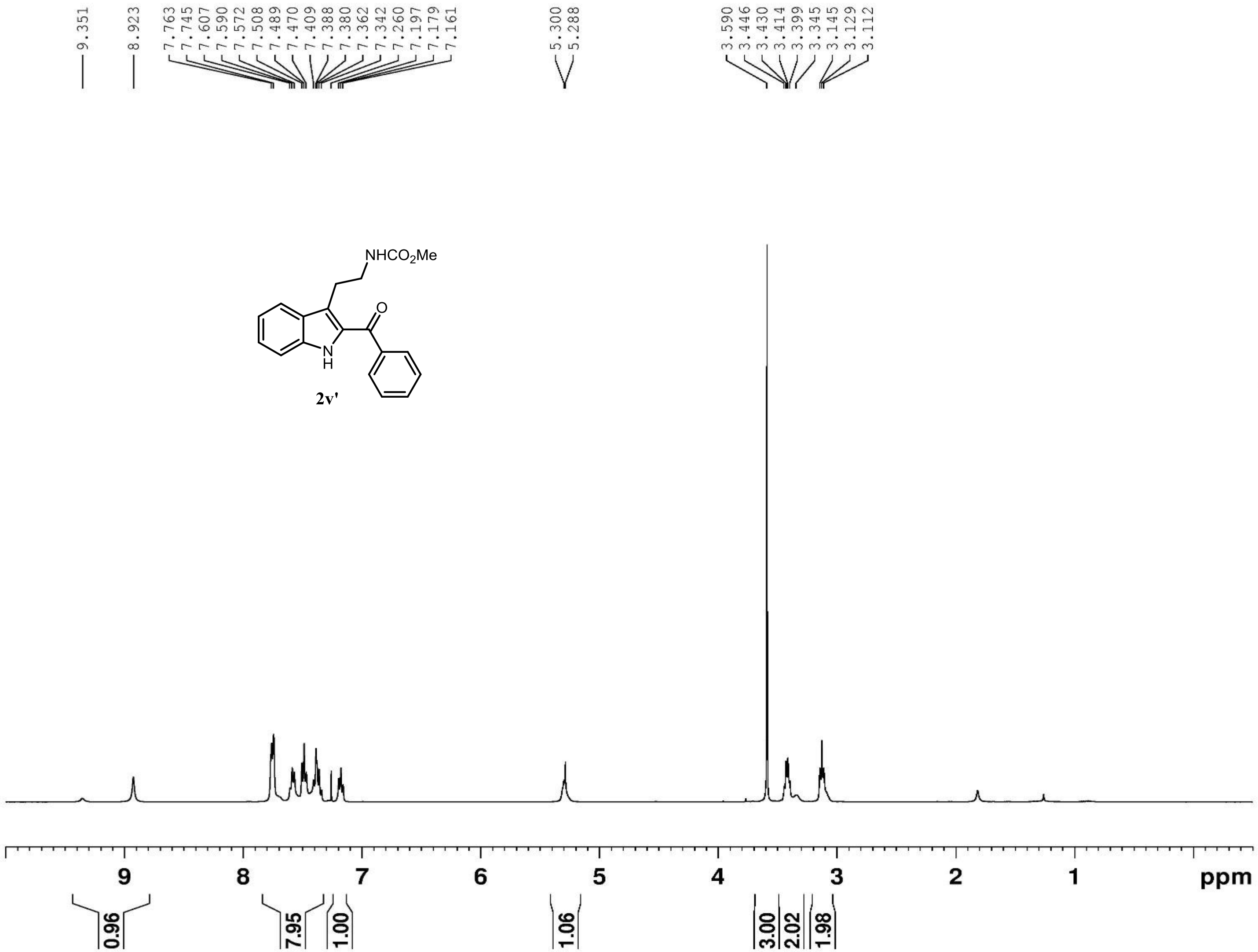


```
NAME          llx-3196-1
EXPNO                  1
PROCNO                 1
Date_           20190812
Time               13.22
INSTRUM            spect
PROBHD   5 mm PABBO BB/
PULPROG             zg30
TD                 65536
SOLVENT            CDCl3
NS                     9
DS                     2
SWH             8012.820 Hz
FIDRES          0.122266 Hz
AQ             4.0894966 sec
RG                 62.93
DW                62.400 usec
DE                  6.50 usec
TE                 296.0 K
D1            1.00000000 sec
TD0                    1

======== CHANNEL f1 ========
SFO1         400.1324710 MHz
NUC1                  1H
P1                 14.50 usec
SI                 65536
SF           400.1300098 MHz
WDW                   EM
SSB                    0
LB                  0.30 Hz
GB                     0
PC                  1.00
```

Supplementary Figure 92. ^{1}H-NMR of 2v'

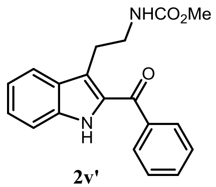

2v'

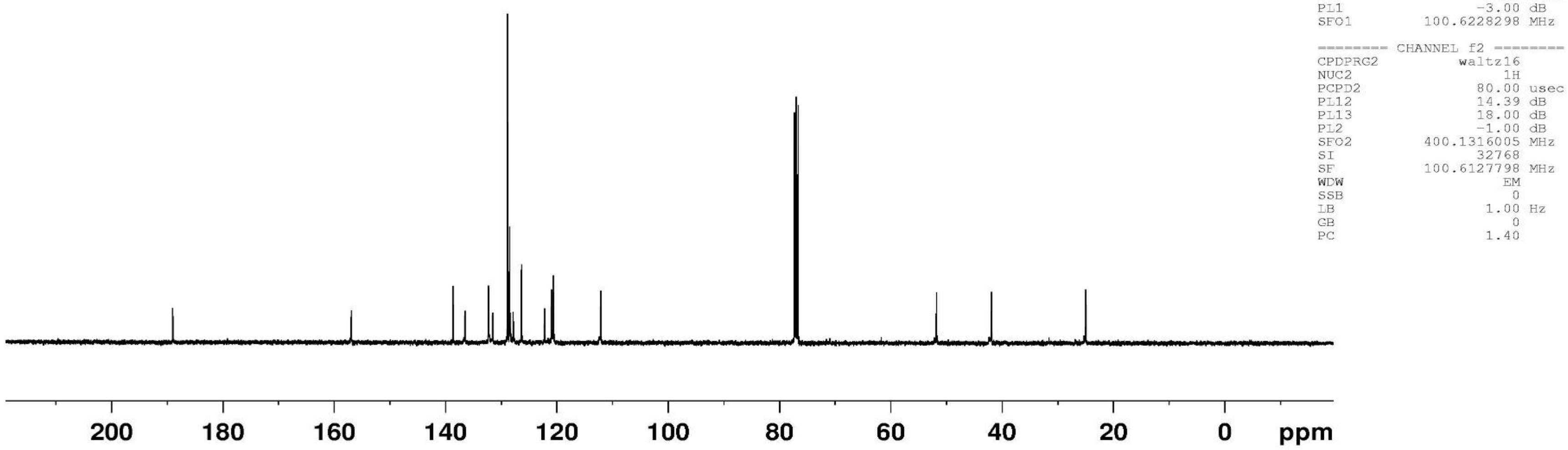

Supplementary Figure 93. ^{13}C-NMR of 2v'

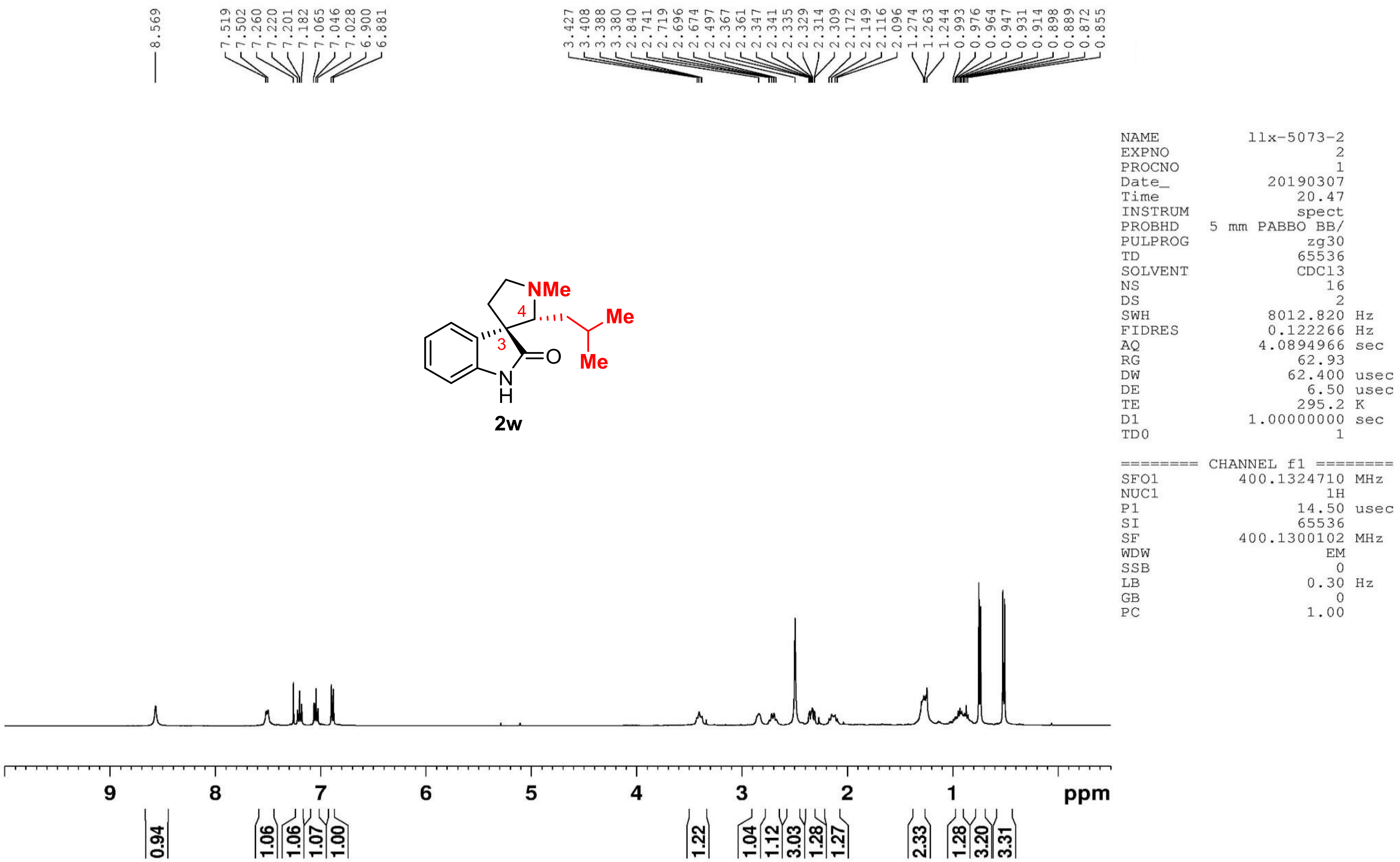

Supplementary Figure 94. ^{1}H-NMR of 2w

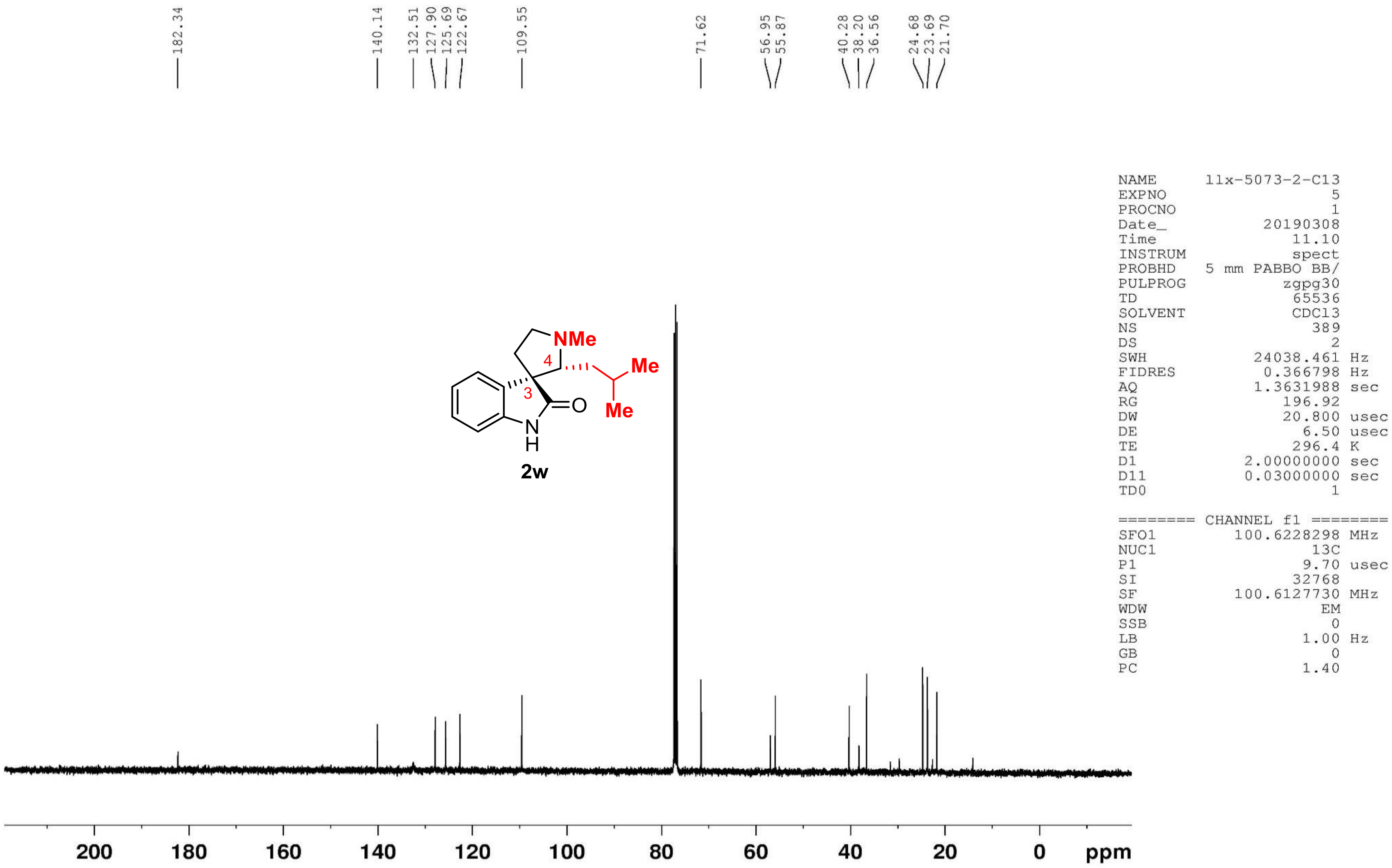

NAME	llx-5073-2-C13
EXPNO	5
PROCNO	1
Date_	20190308
Time	11.10
INSTRUM	spect
PROBHD	5 mm PABBO BB/
PULPROG	zgpg30
TD	65536
SOLVENT	CDCl3
NS	389
DS	2
SWH	24038.461 Hz
FIDRES	0.366798 Hz
AQ	1.3631988 sec
RG	196.92
DW	20.800 usec
DE	6.50 usec
TE	296.4 K
D1	2.00000000 sec
D11	0.03000000 sec
TD0	1
======== CHANNEL f1 ========	
SFO1	100.6228298 MHz
NUC1	13C
P1	9.70 usec
SI	32768
SF	100.6127730 MHz
WDW	EM
SSB	0
LB	1.00 Hz
GB	0
PC	1.40

Supplementary Figure 95. ^{13}C-NMR of 2w

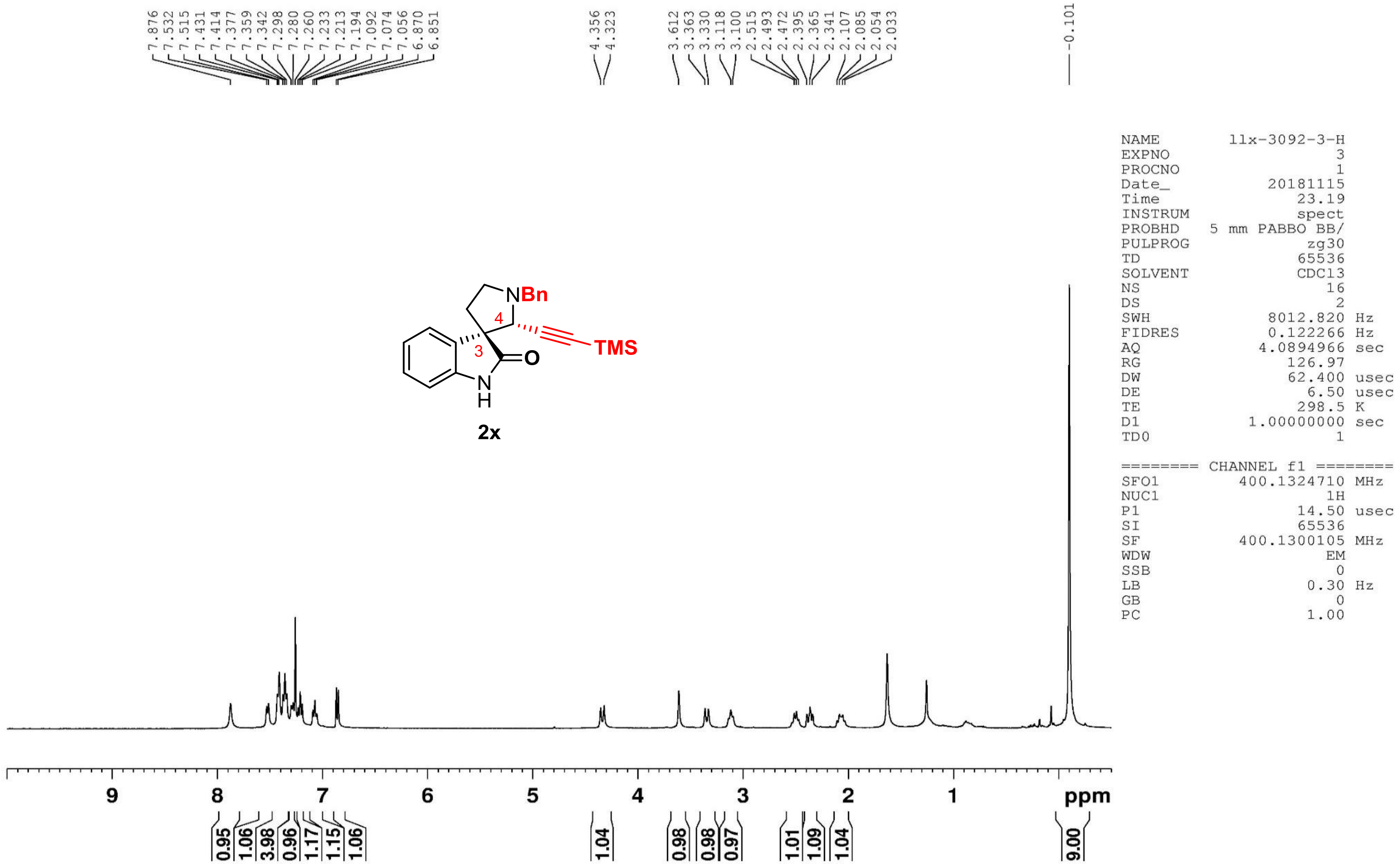

Supplementary Figure 96. ^{1}H-NMR of 2x

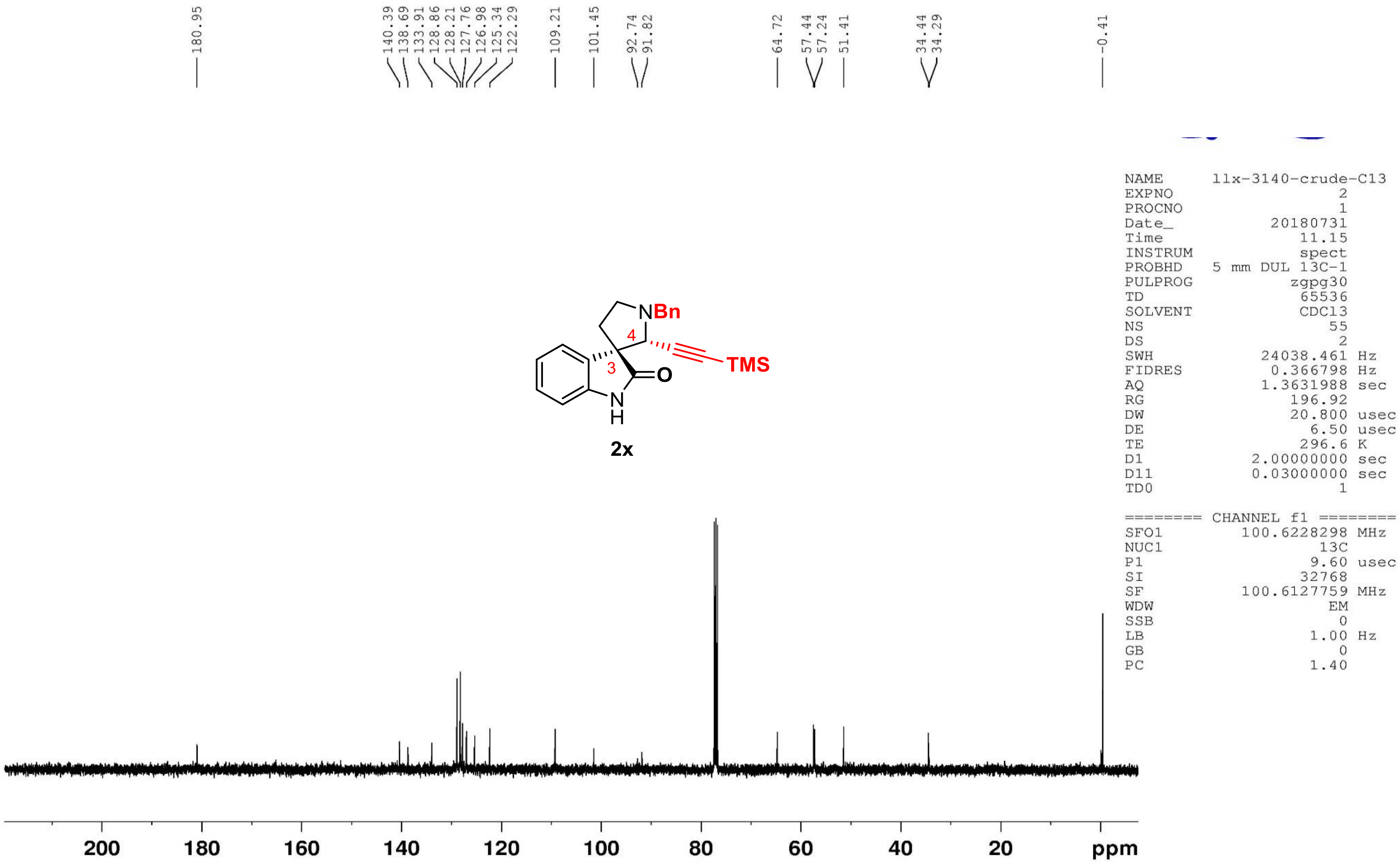

NAME llx-3140-crude-C13
EXPNO 2
PROCNO 1
Date_ 20180731
Time 11.15
INSTRUM spect
PROBHD 5 mm DUL 13C-1
PULPROG zgpg30
TD 65536
SOLVENT CDCl3
NS 55
DS 2
SWH 24038.461 Hz
FIDRES 0.366798 Hz
AQ 1.3631988 sec
RG 196.92
DW 20.800 usec
DE 6.50 usec
TE 296.6 K
D1 2.00000000 sec
D11 0.03000000 sec
TD0 1

======== CHANNEL f1 ========
SFO1 100.6228298 MHz
NUC1 13C
P1 9.60 usec
SI 32768
SF 100.6127759 MHz
WDW EM
SSB 0
LB 1.00 Hz
GB 0
PC 1.40

Supplementary Figure 97. ^{13}C-NMR of 2x

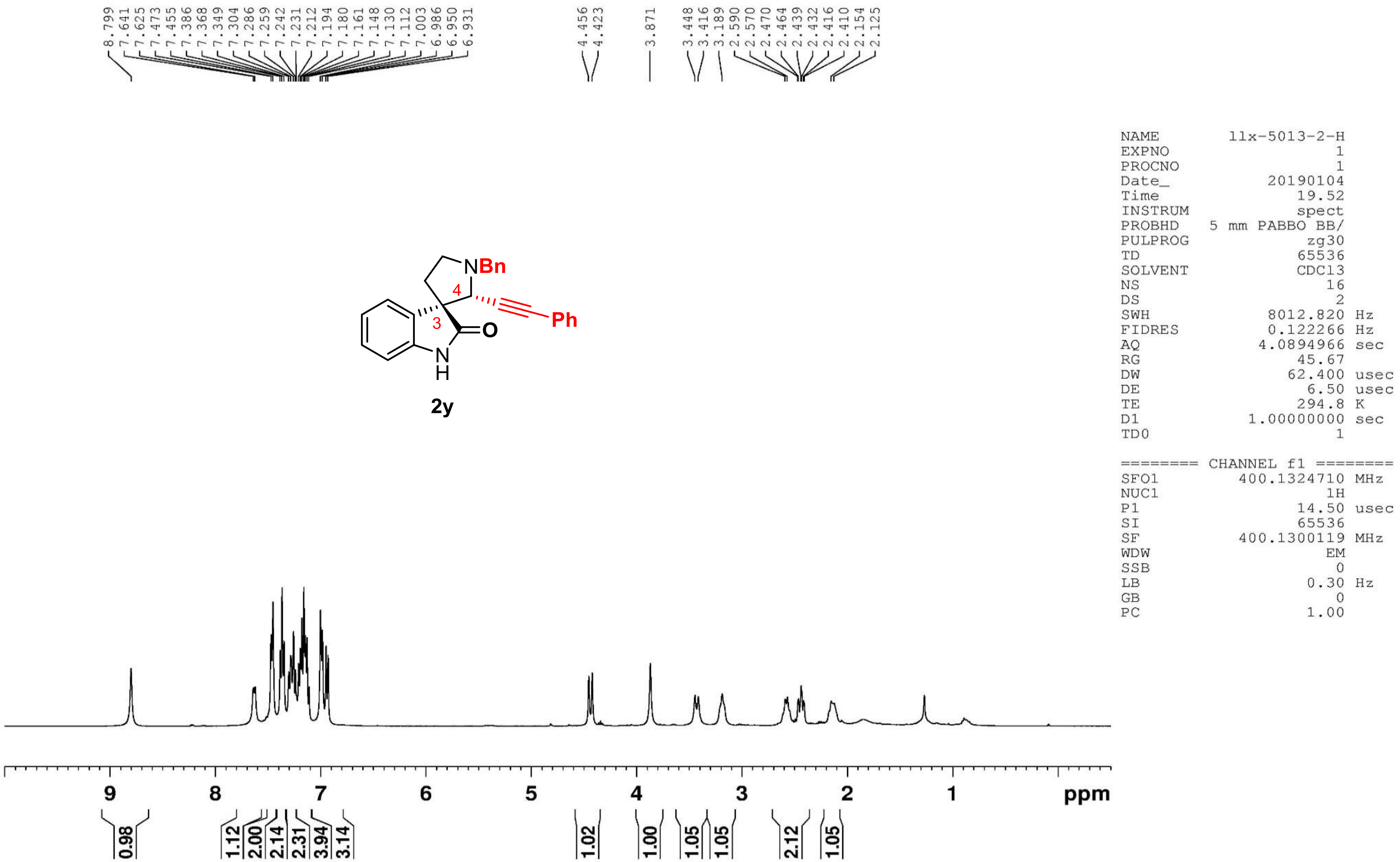

Supplementary Figure 98. ¹H-NMR of 2y

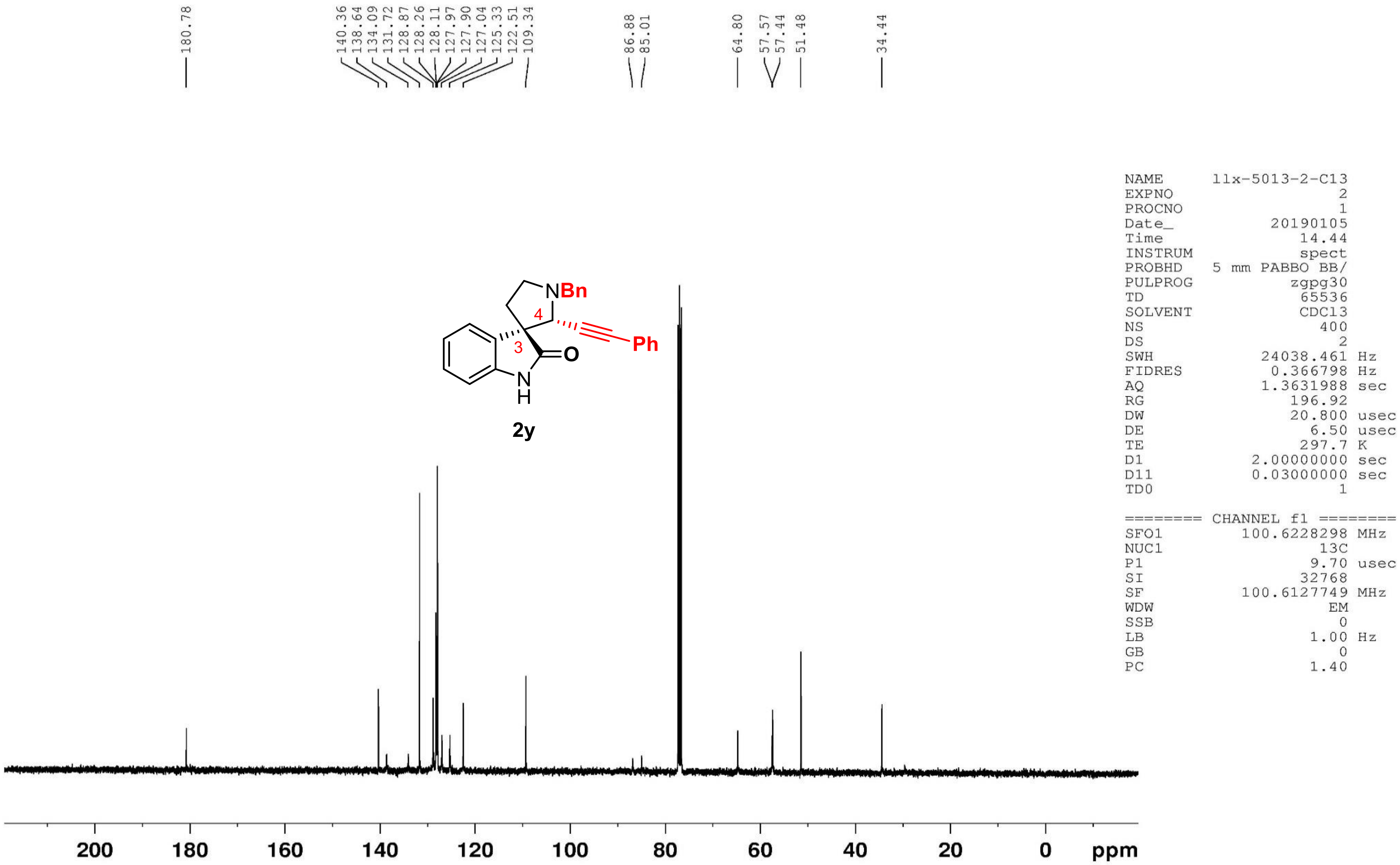

NAME	llx-5013-2-C13
EXPNO	2
PROCNO	1
Date_	20190105
Time	14.44
INSTRUM	spect
PROBHD	5 mm PABBO BB/
PULPROG	zgpg30
TD	65536
SOLVENT	CDCl3
NS	400
DS	2
SWH	24038.461 Hz
FIDRES	0.366798 Hz
AQ	1.3631988 sec
RG	196.92
DW	20.800 usec
DE	6.50 usec
TE	297.7 K
D1	2.00000000 sec
D11	0.03000000 sec
TD0	1
======== CHANNEL f1 ========	
SFO1	100.6228298 MHz
NUC1	13C
P1	9.70 usec
SI	32768
SF	100.6127749 MHz
WDW	EM
SSB	0
LB	1.00 Hz
GB	0
PC	1.40

Supplementary Figure 99. ^{13}C-NMR of 2y

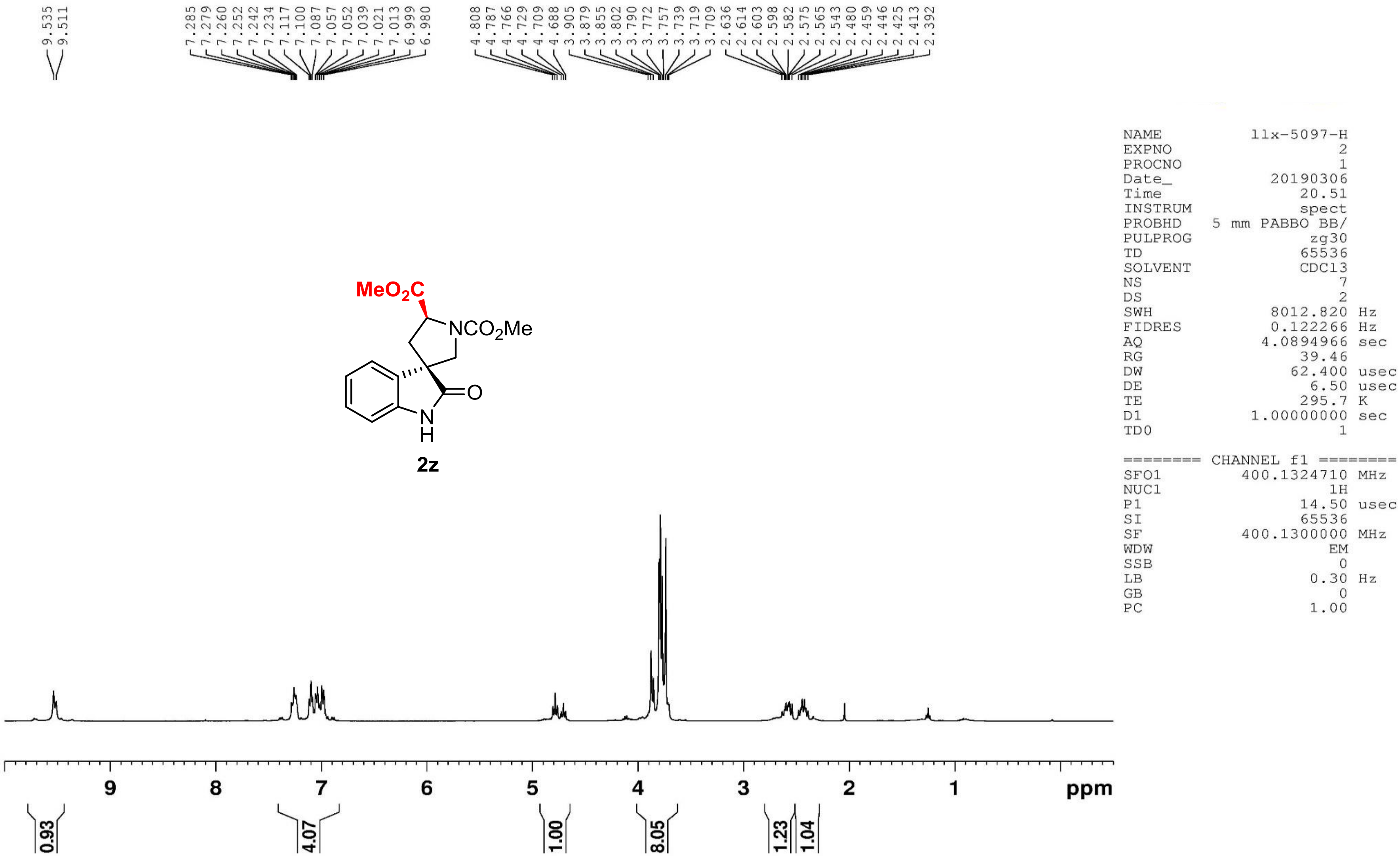

Supplementary Figure 100. ^{1}H-NMR of 2z

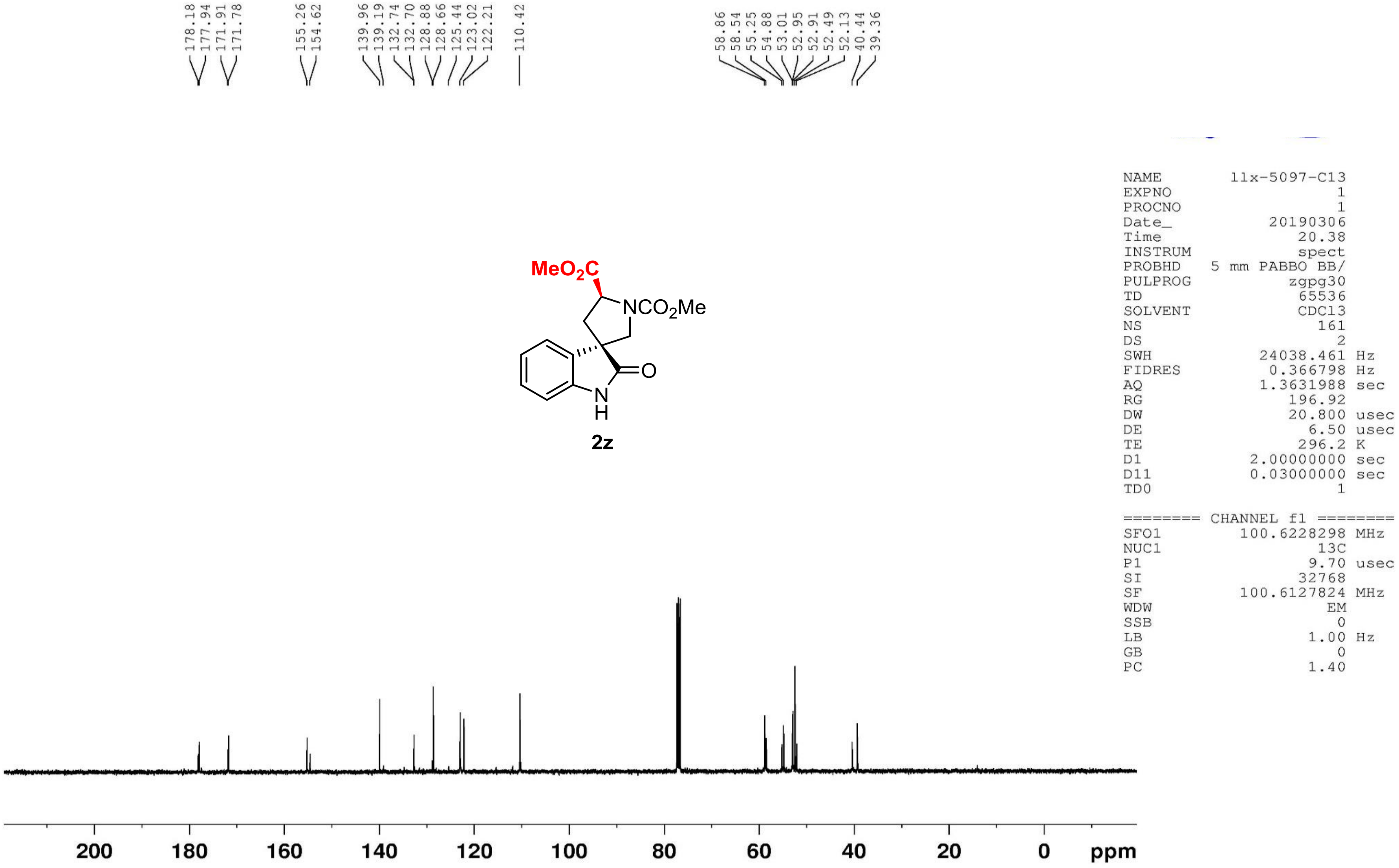

Supplementary Figure 101. ^{13}C-NMR of 2z

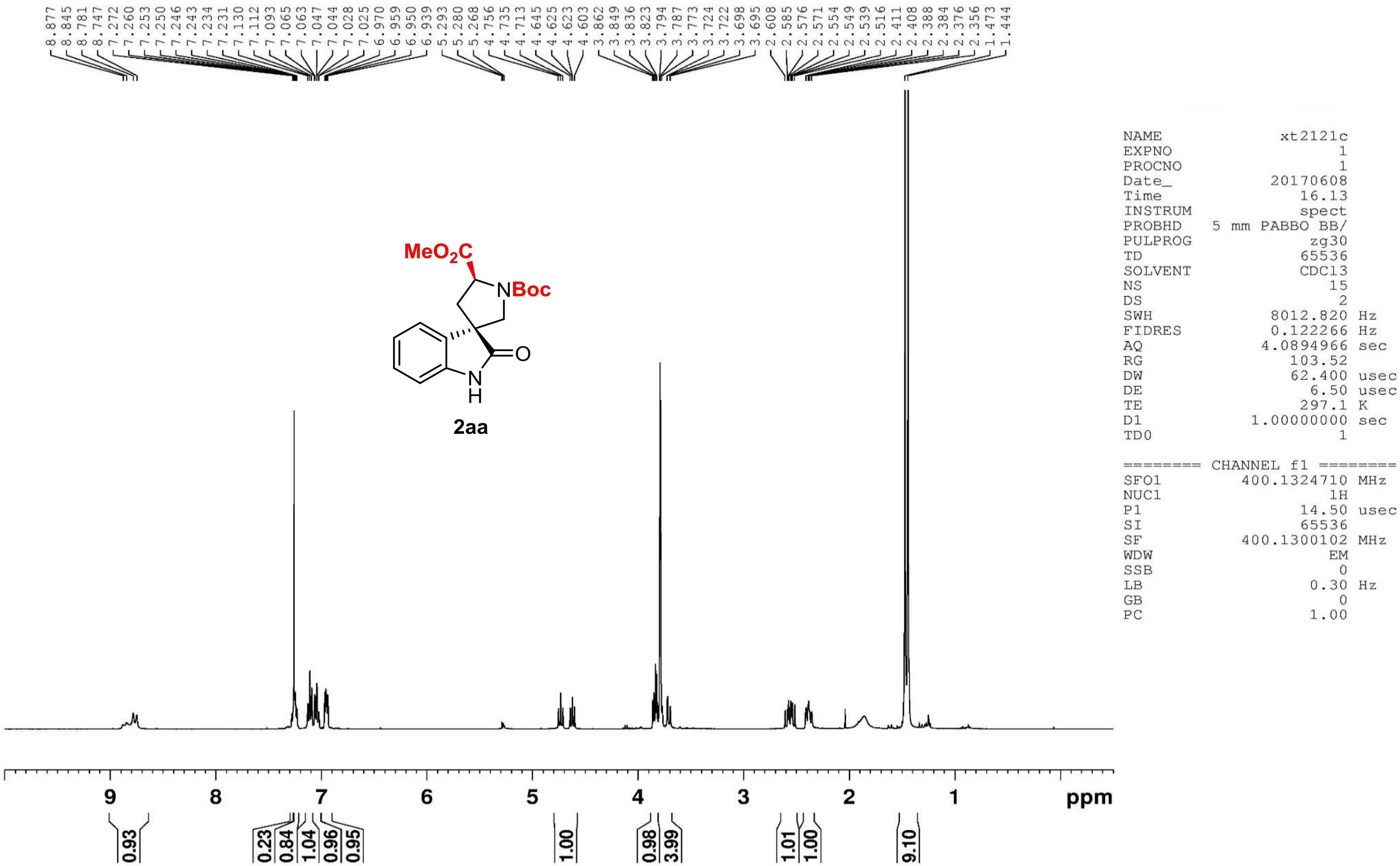

Supplementary Figure 102. ^{1}H-NMR of 2aa

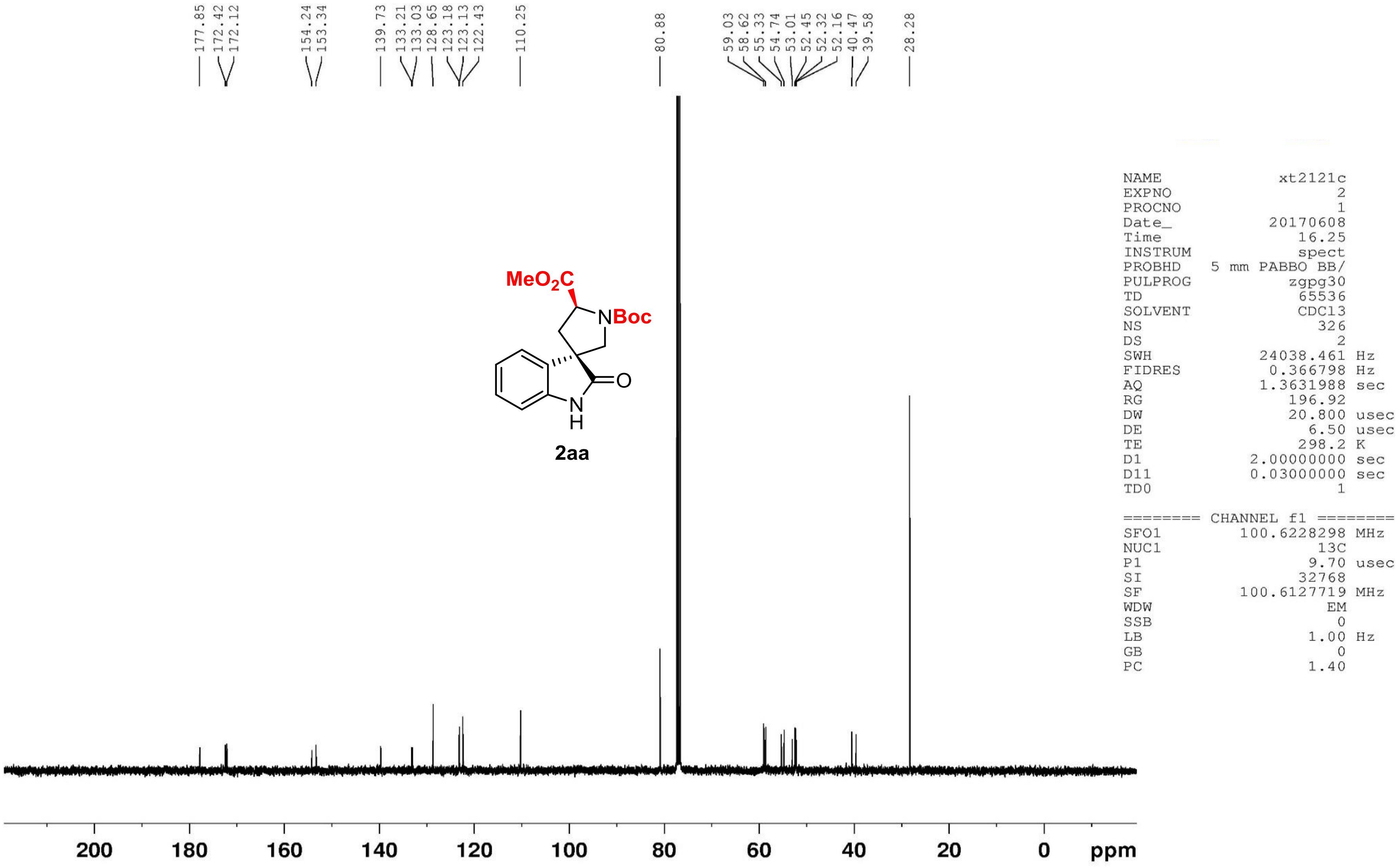

Supplementary Figure 103. ^{13}C-NMR of 2aa

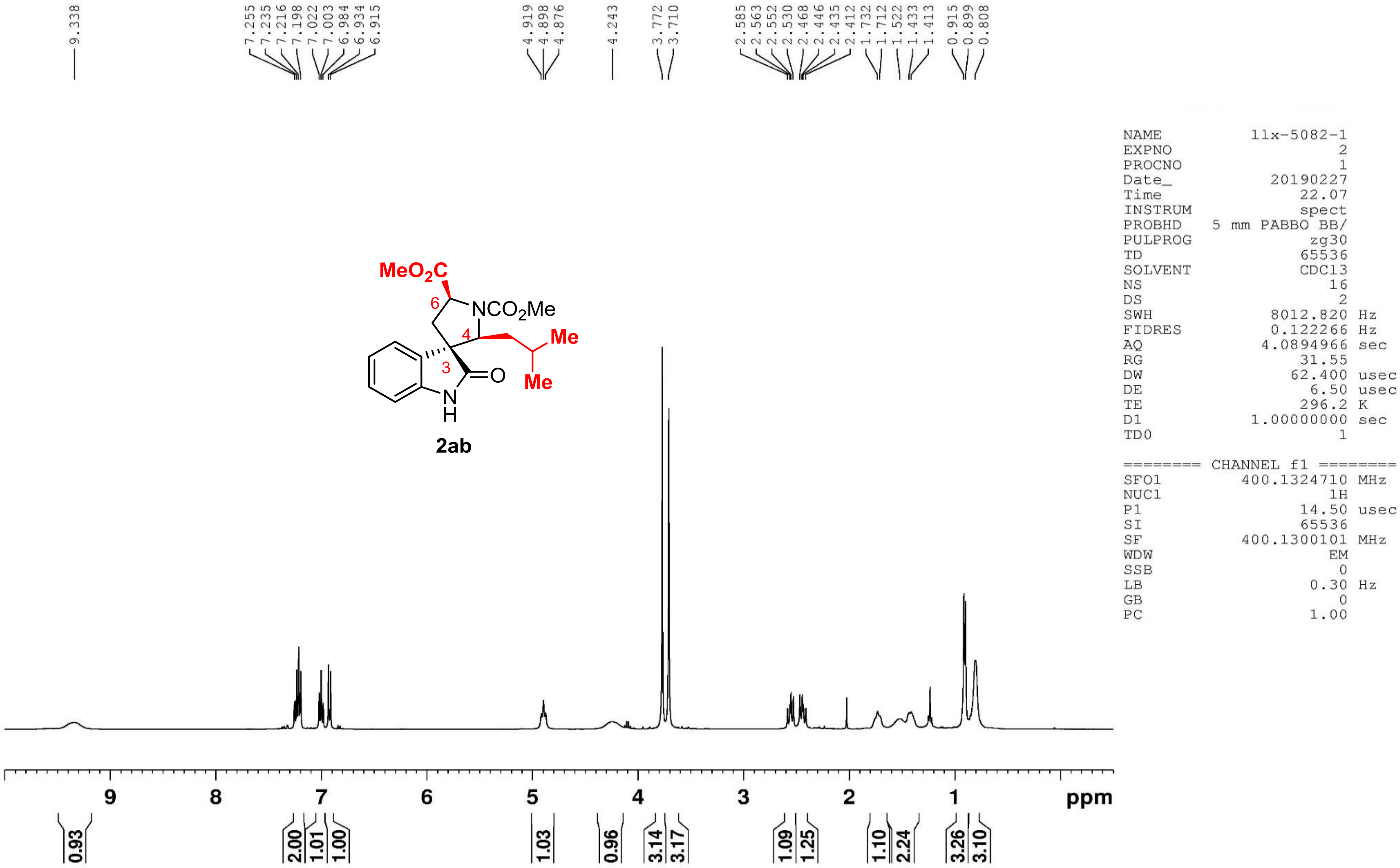

Supplementary Figure 104. ^{1}H-NMR of 2ab

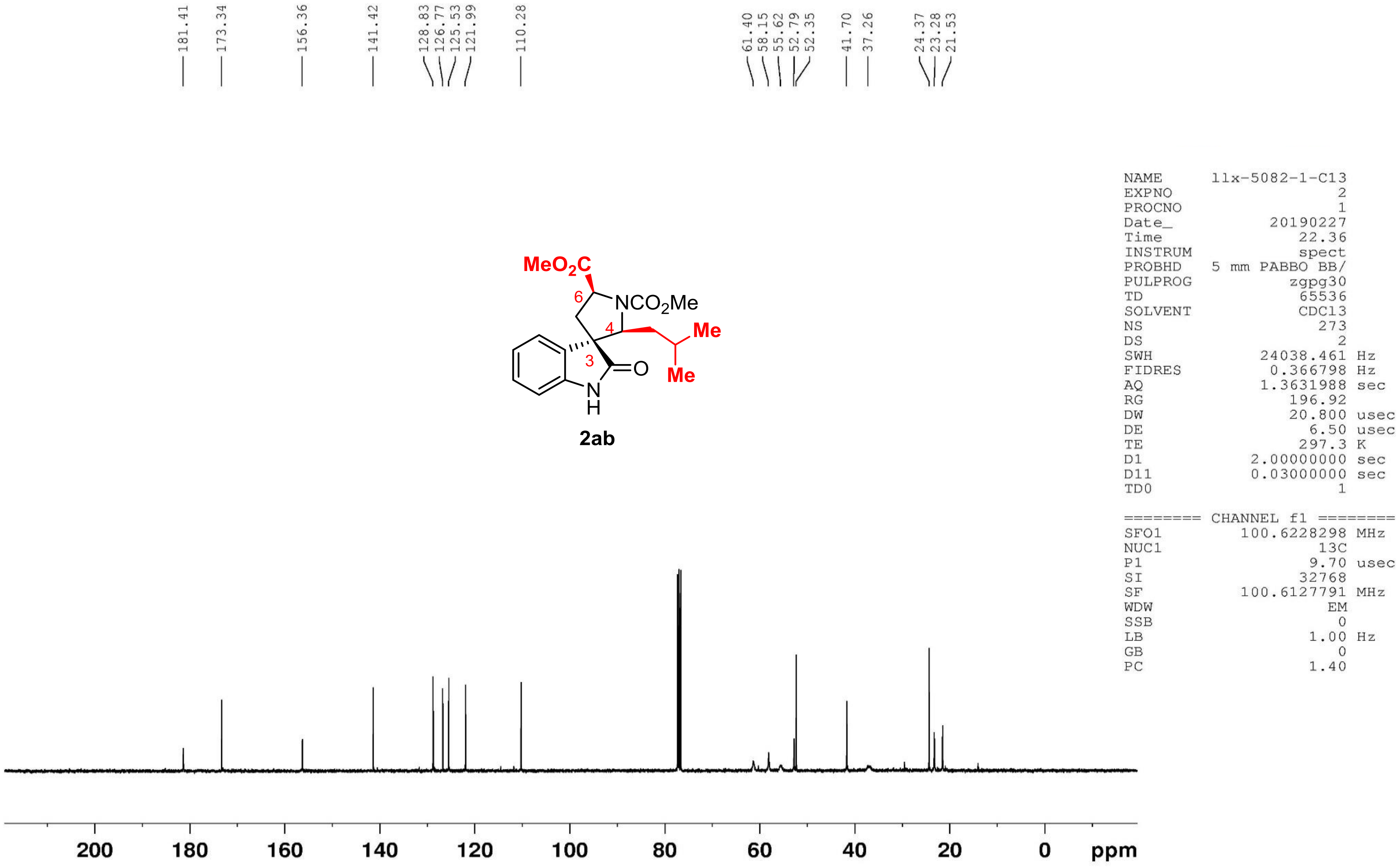


```
NAME         llx-5082-1-C13
EXPNO                     2
PROCNO                    1
Date_              20190227
Time                  22.36
INSTRUM               spect
PROBHD       5 mm PABBO BB/
PULPROG             zgpg30
TD                   65536
SOLVENT              CDCl3
NS                     273
DS                       2
SWH              24038.461 Hz
FIDRES            0.366798 Hz
AQ               1.3631988 sec
RG                  196.92
DW                  20.800 usec
DE                    6.50 usec
TE                   297.3 K
D1              2.00000000 sec
D11             0.03000000 sec
TD0                      1

======== CHANNEL f1 ========
SFO1          100.6228298 MHz
NUC1                  13C
P1                   9.70 usec
SI                  32768
SF            100.6127791 MHz
WDW                    EM
SSB                     0
LB                   1.00 Hz
GB                      0
PC                   1.40
```

Supplementary Figure 105. ^{13}C-NMR of 2ab

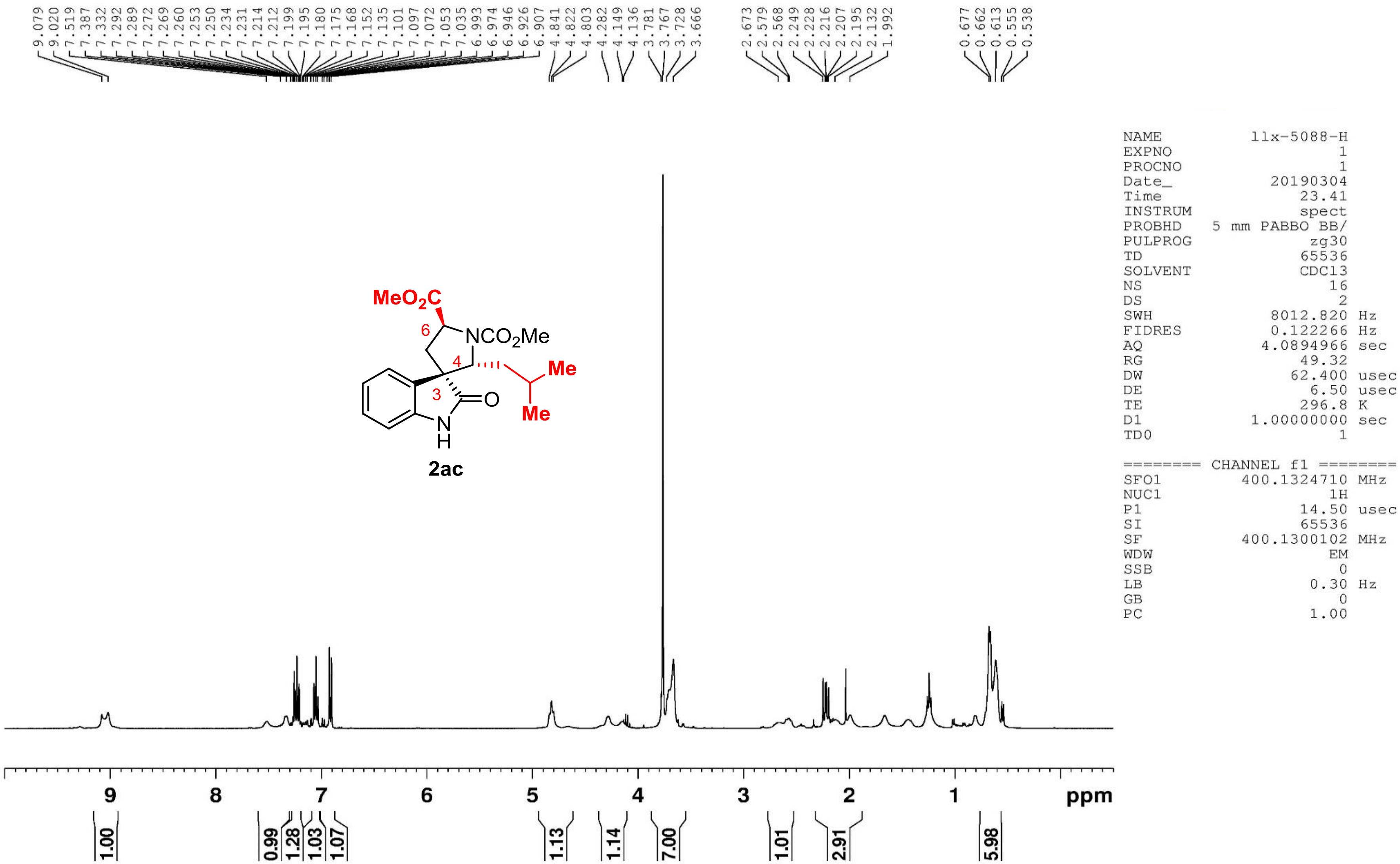

Supplementary Figure 106. ^{1}H-NMR of 2ac

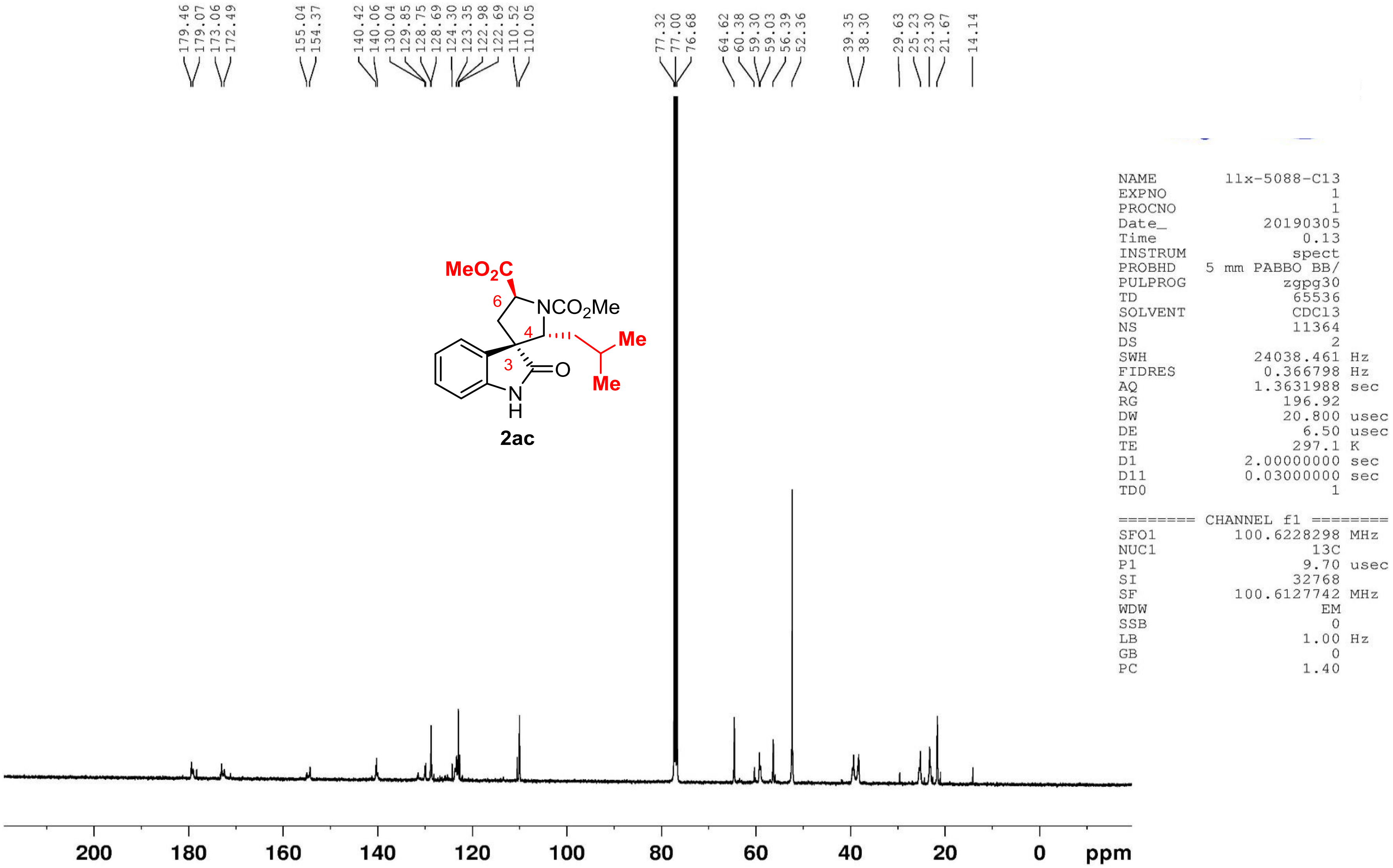

Supplementary Figure 107. ^{13}**C-NMR of 2ac**

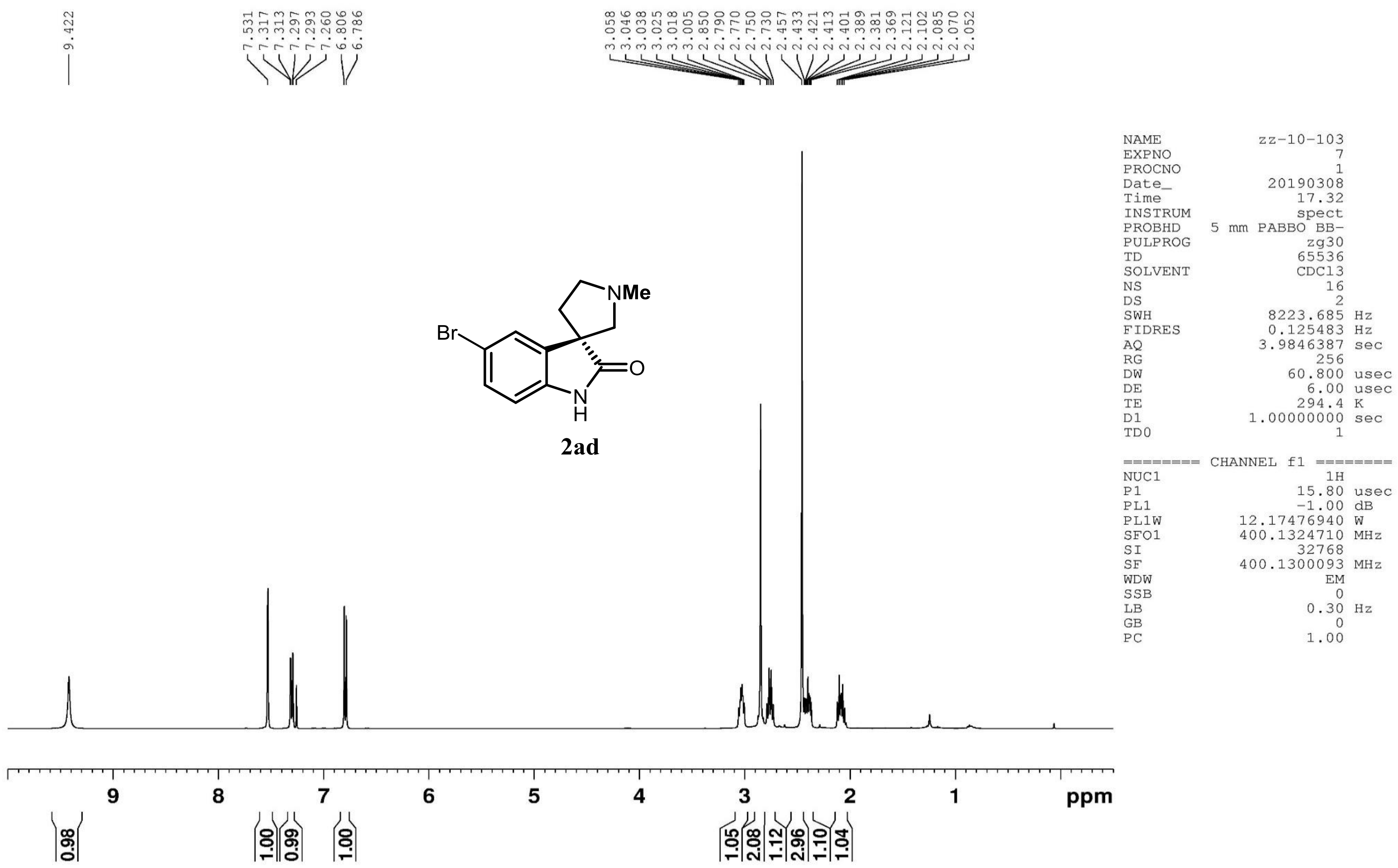

Supplementary Figure 108. ^{1}H-NMR of 2ad

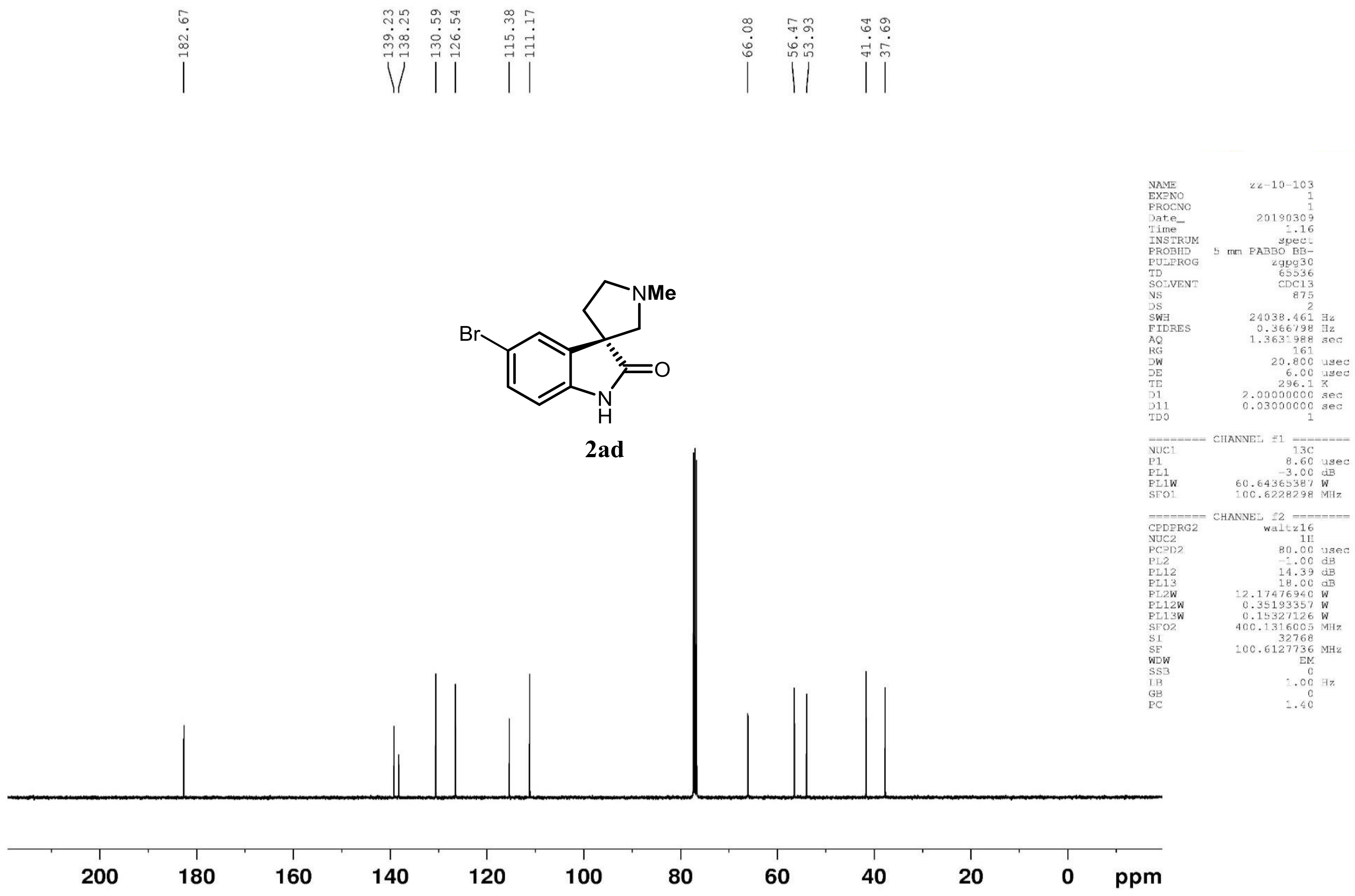

Supplementary Figure 109. ^{13}C-NMR of 2ad

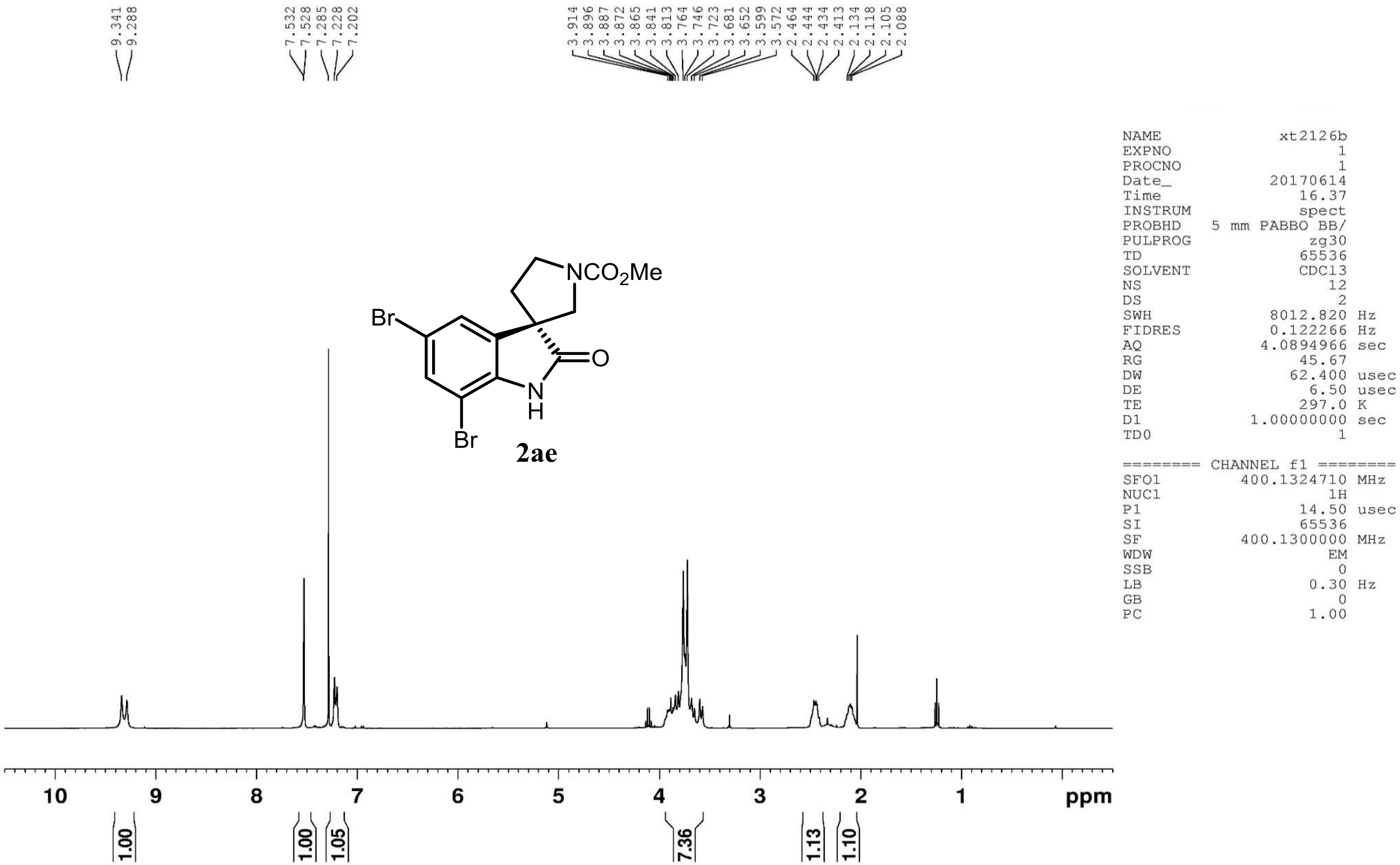

Supplementary Figure 110. ^{1}H-NMR of 2ae

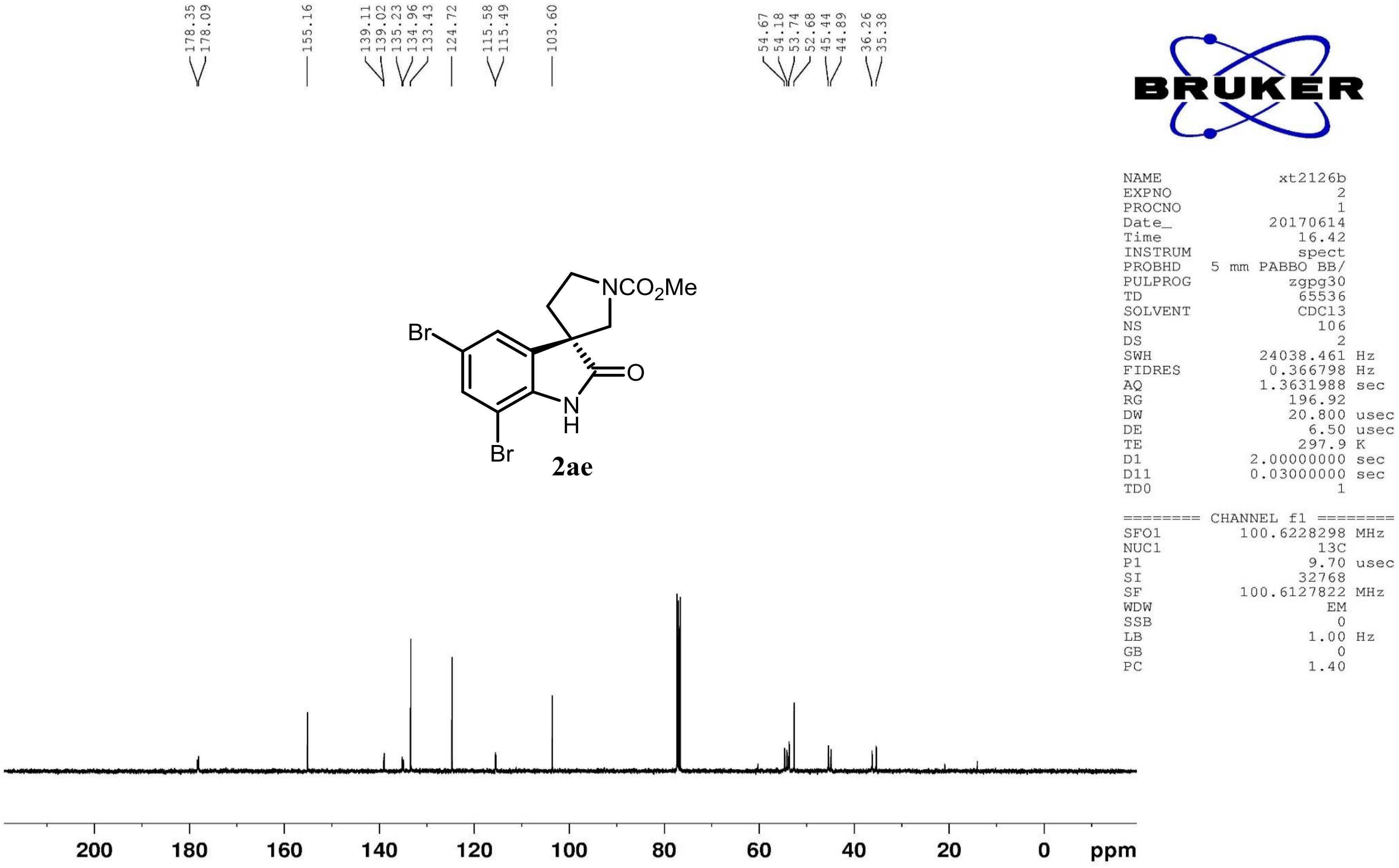

Supplementary Figure 111. ^{13}C-NMR of 2ae

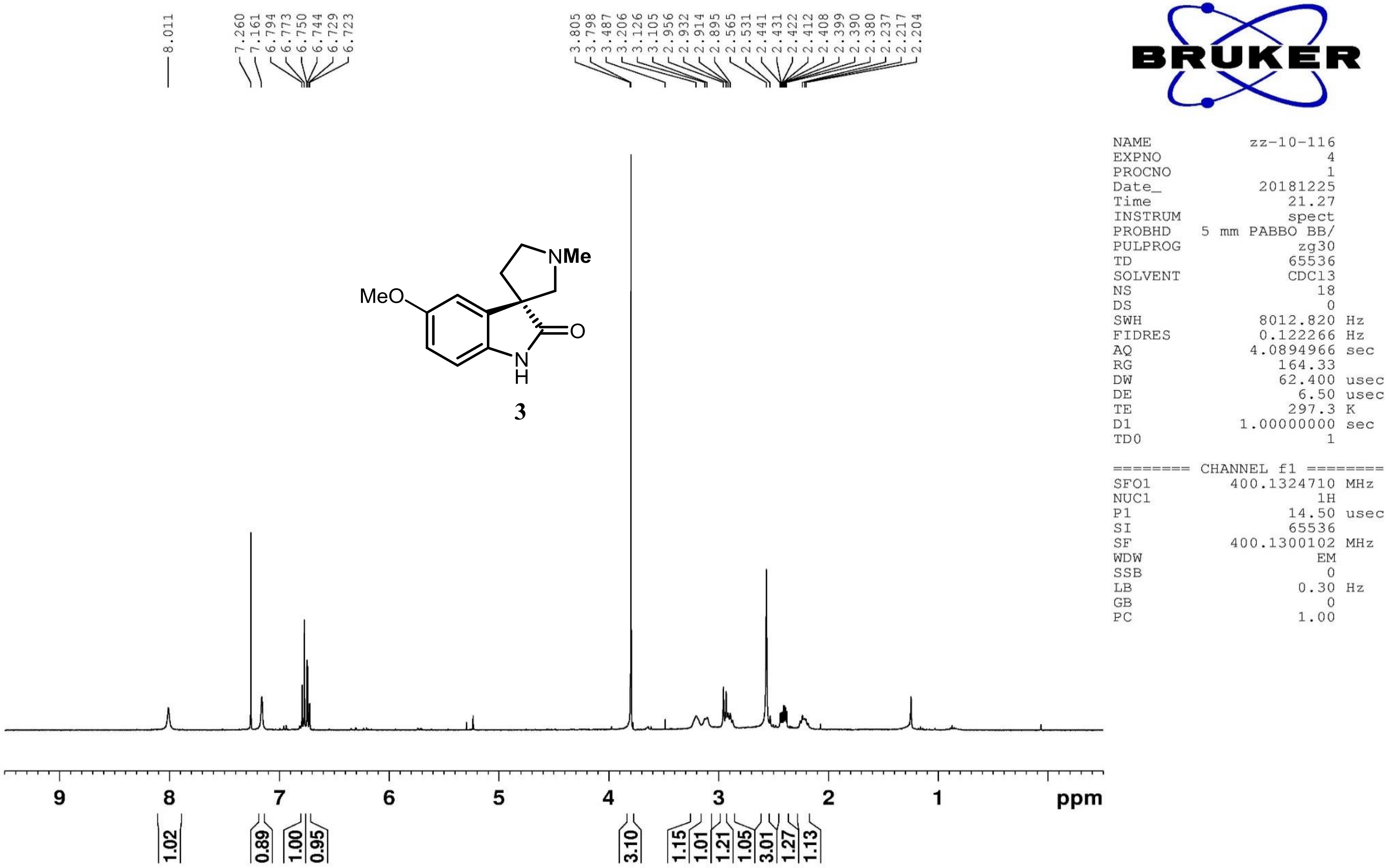

Supplementary Figure 112. ^{1}H-NMR of 3

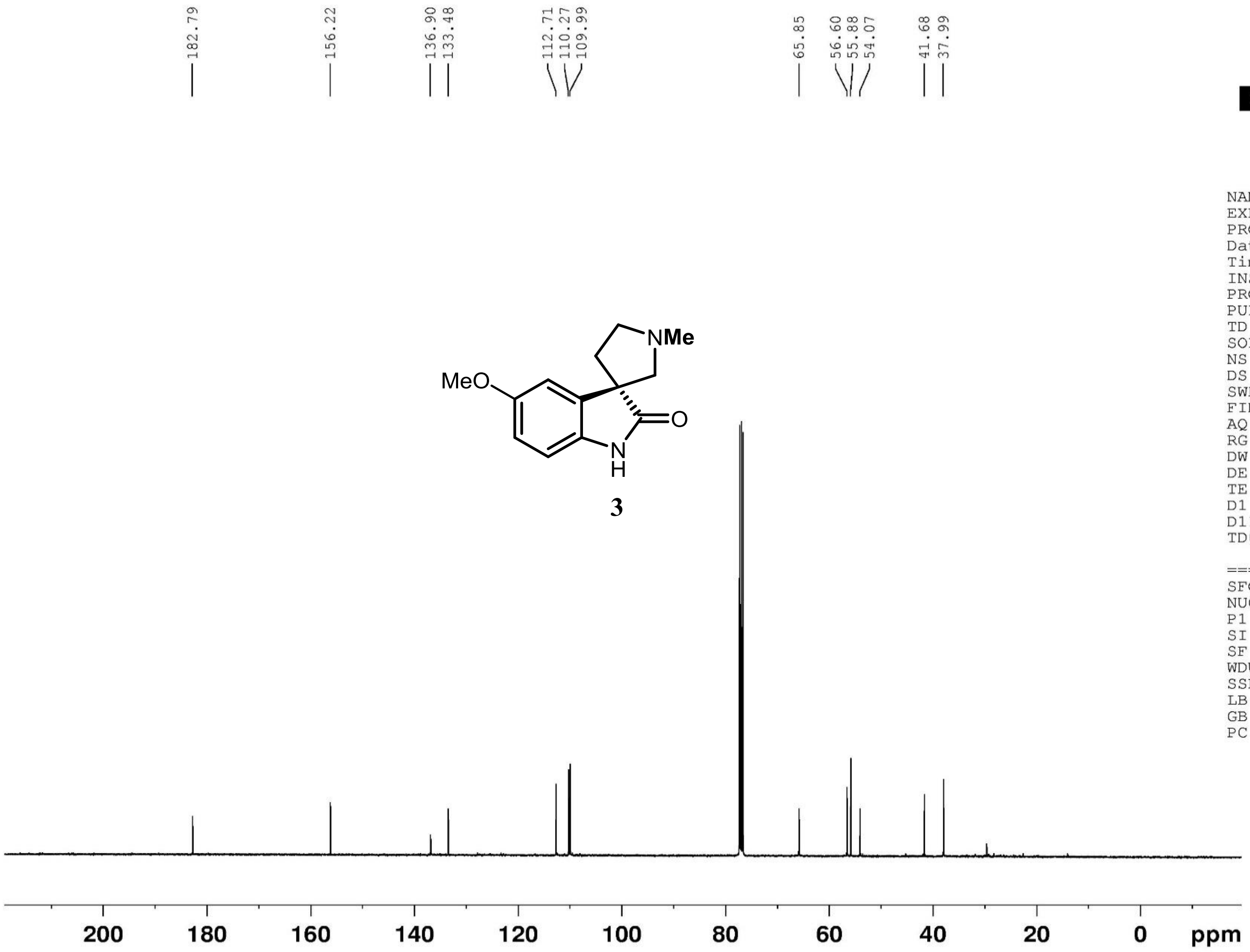


```
NAME            zz-10-128p
EXPNO                    3
PROCNO                   1
Date_             20190104
Time                  6.18
INSTRUM              spect
PROBHD    5 mm PABBO BB/
PULPROG            zgpg30
TD                   65536
SOLVENT              CDCl3
NS                    4000
DS                       2
SWH              24038.461 Hz
FIDRES            0.366798 Hz
AQ               1.3631988 sec
RG                  196.92
DW                  20.800 usec
DE                    6.50 usec
TE                   298.5 K
D1              2.00000000 sec
D11             0.03000000 sec
TD0                      1

======== CHANNEL f1 ========
SFO1          100.6228298 MHz
NUC1                  13C
P1                   9.70 usec
SI                  32768
SF            100.6127730 MHz
WDW                    EM
SSB                     0
LB                   1.00 Hz
GB                      0
PC                   1.40
```

Supplementary Figure 113. ^{13}C-NMR of 3

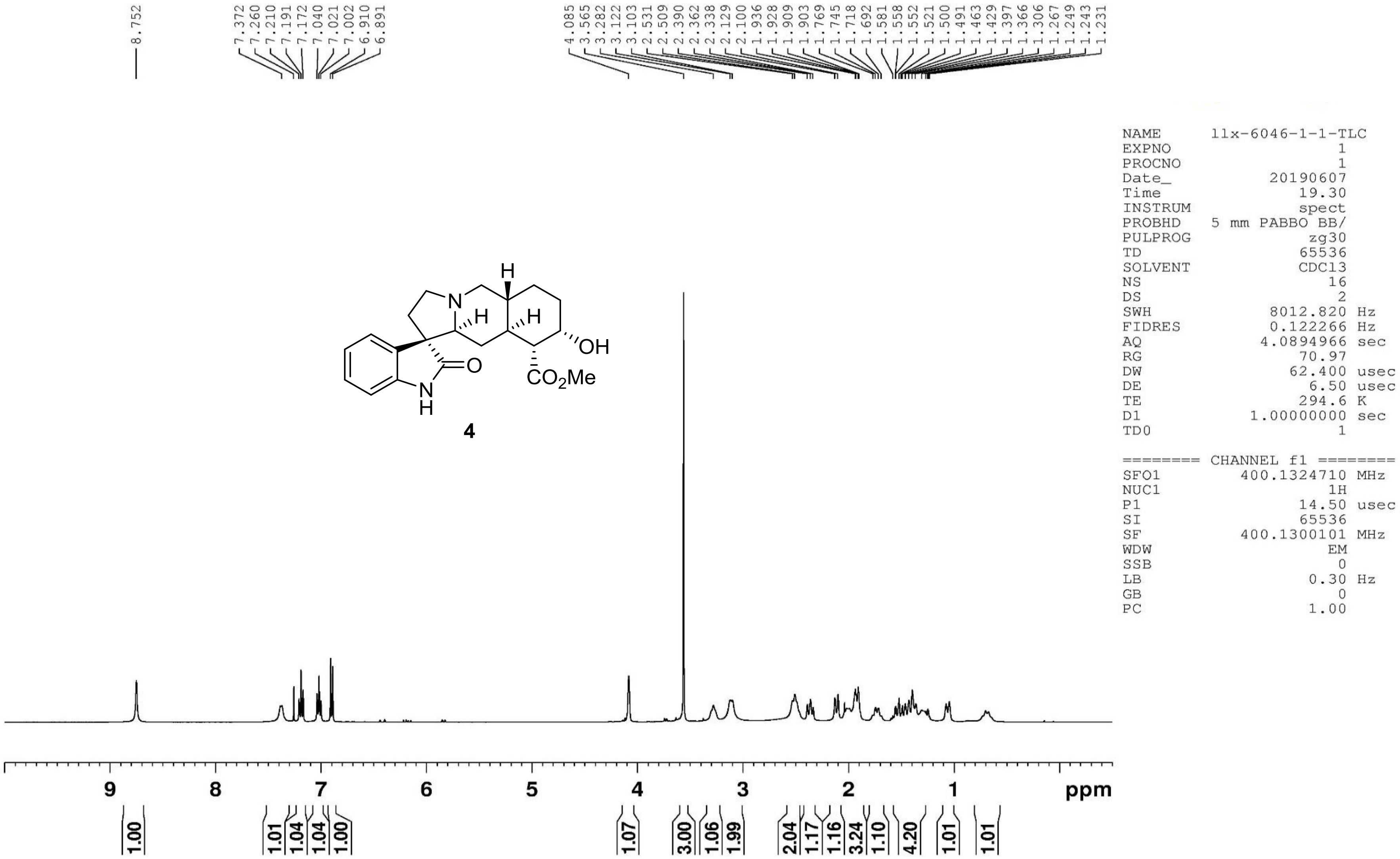

Supplementary Figure 114. ^{1}**H-NMR of 4**

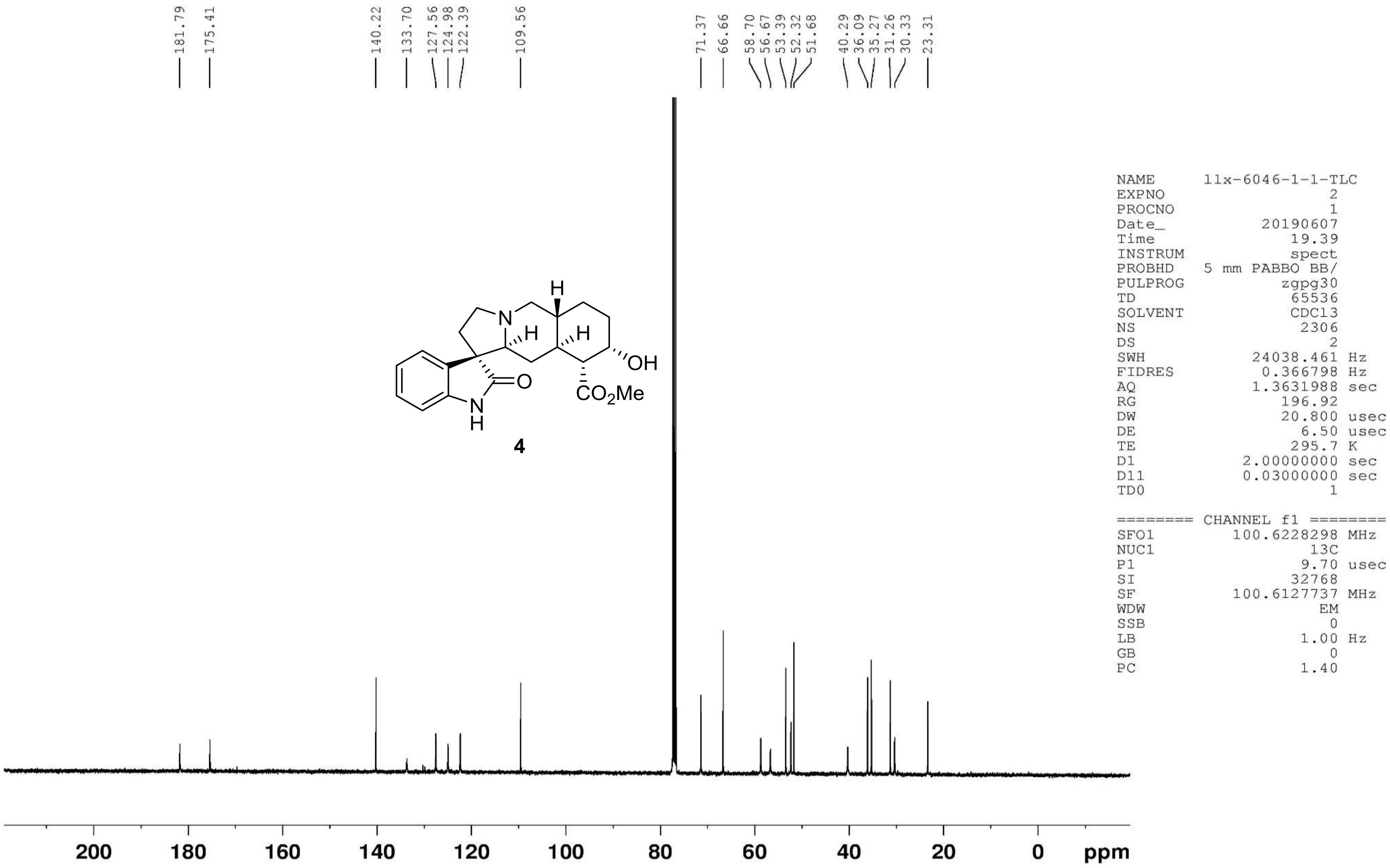

Supplementary Figure 115. ^{13}C-NMR of 4

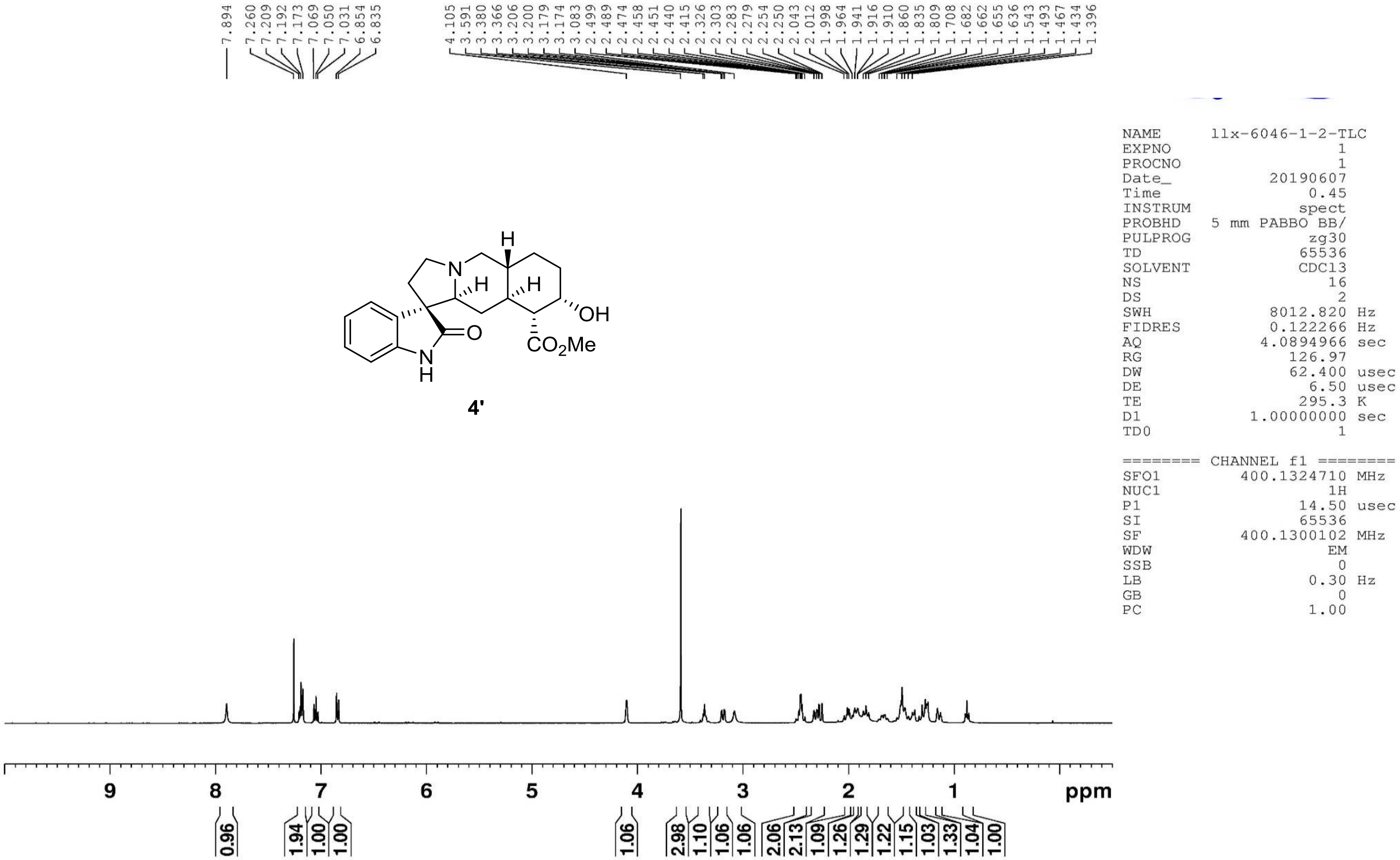

Supplementary Figure 116. ^{1}H-NMR of 4'

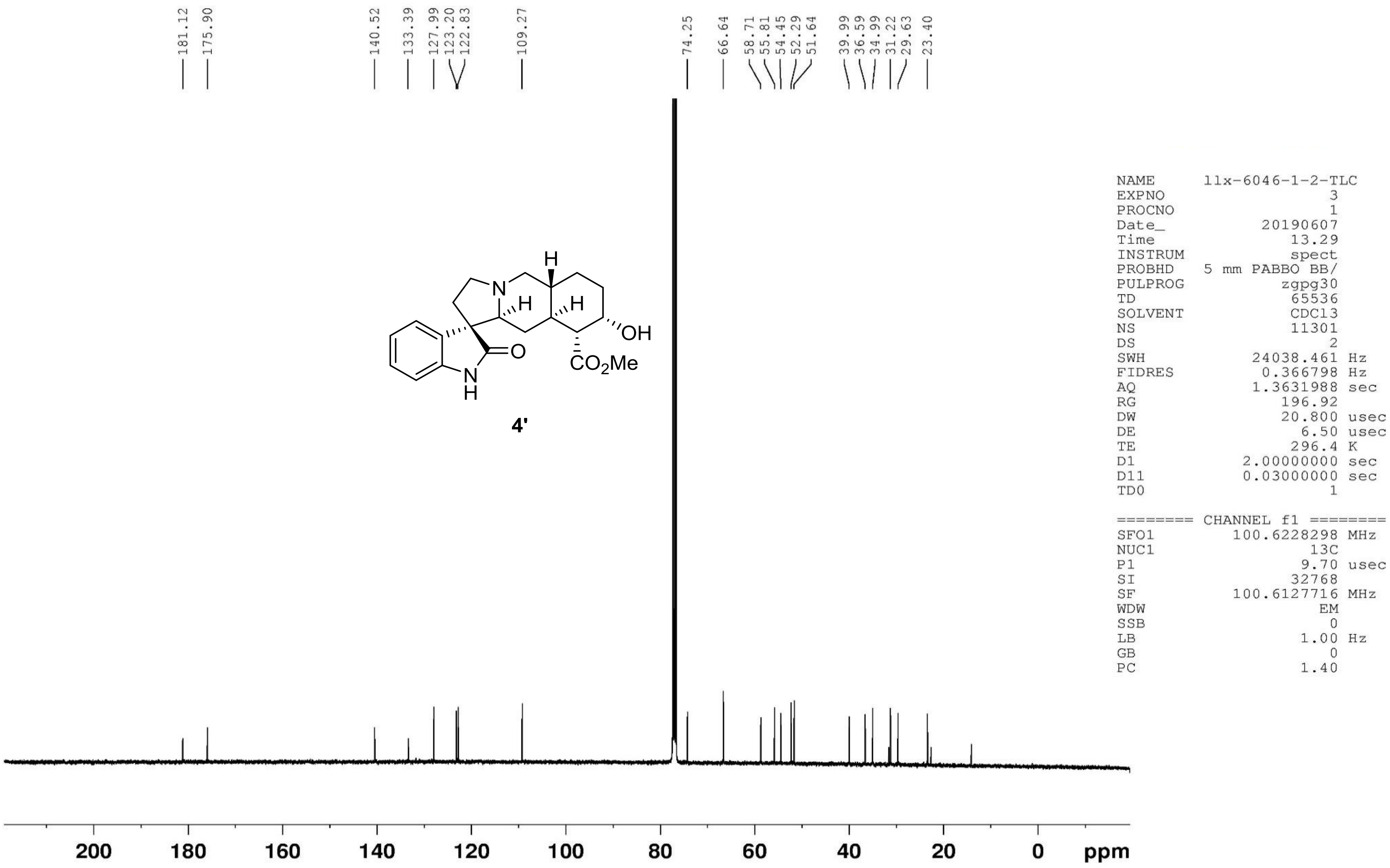

Supplementary Figure 117. ^{13}C-NMR of 4’

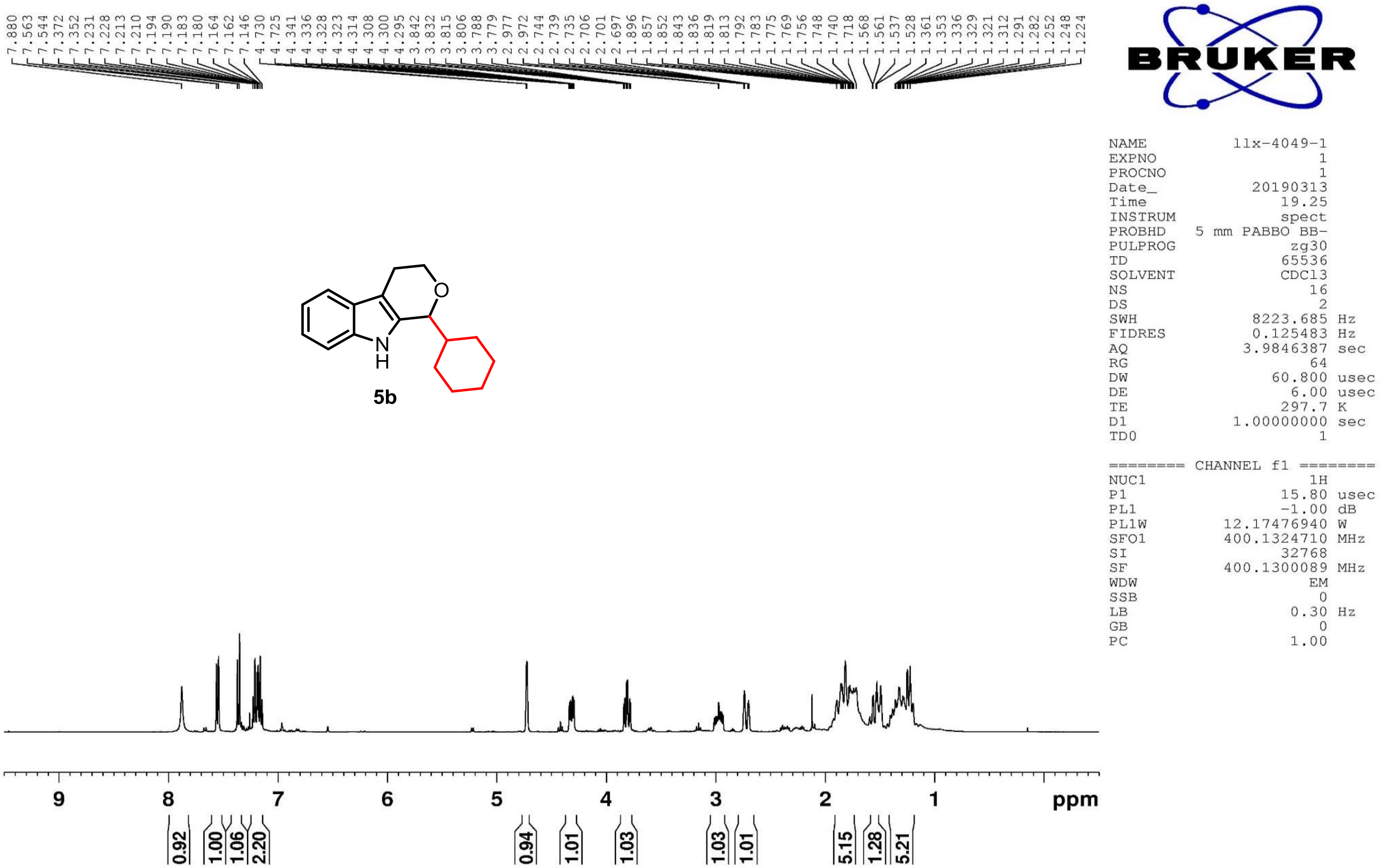

Supplementary Figure 118. ^{1}**H-NMR of 5b**

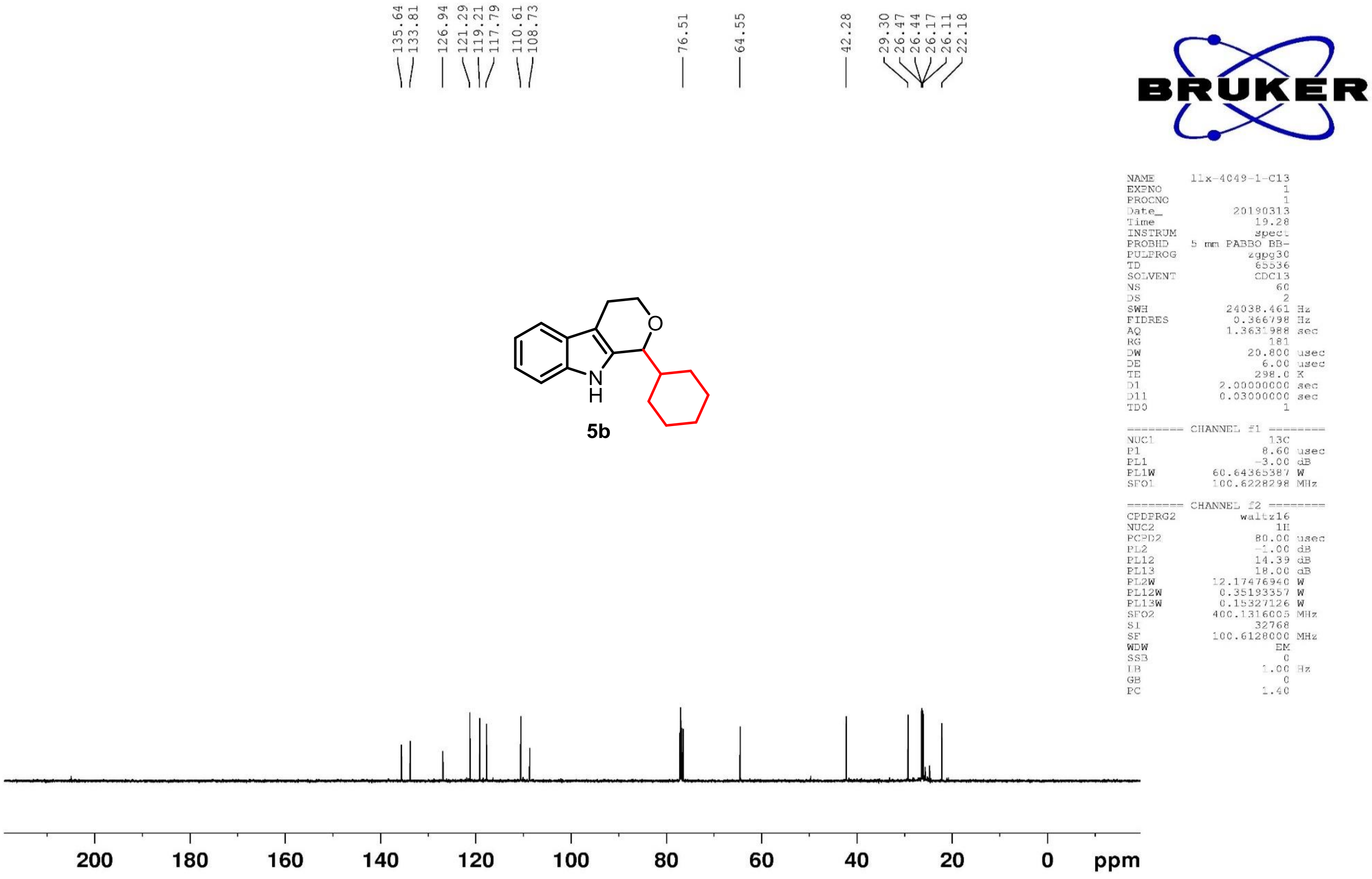

Supplementary Figure 119. ^{13}C-NMR of 5b

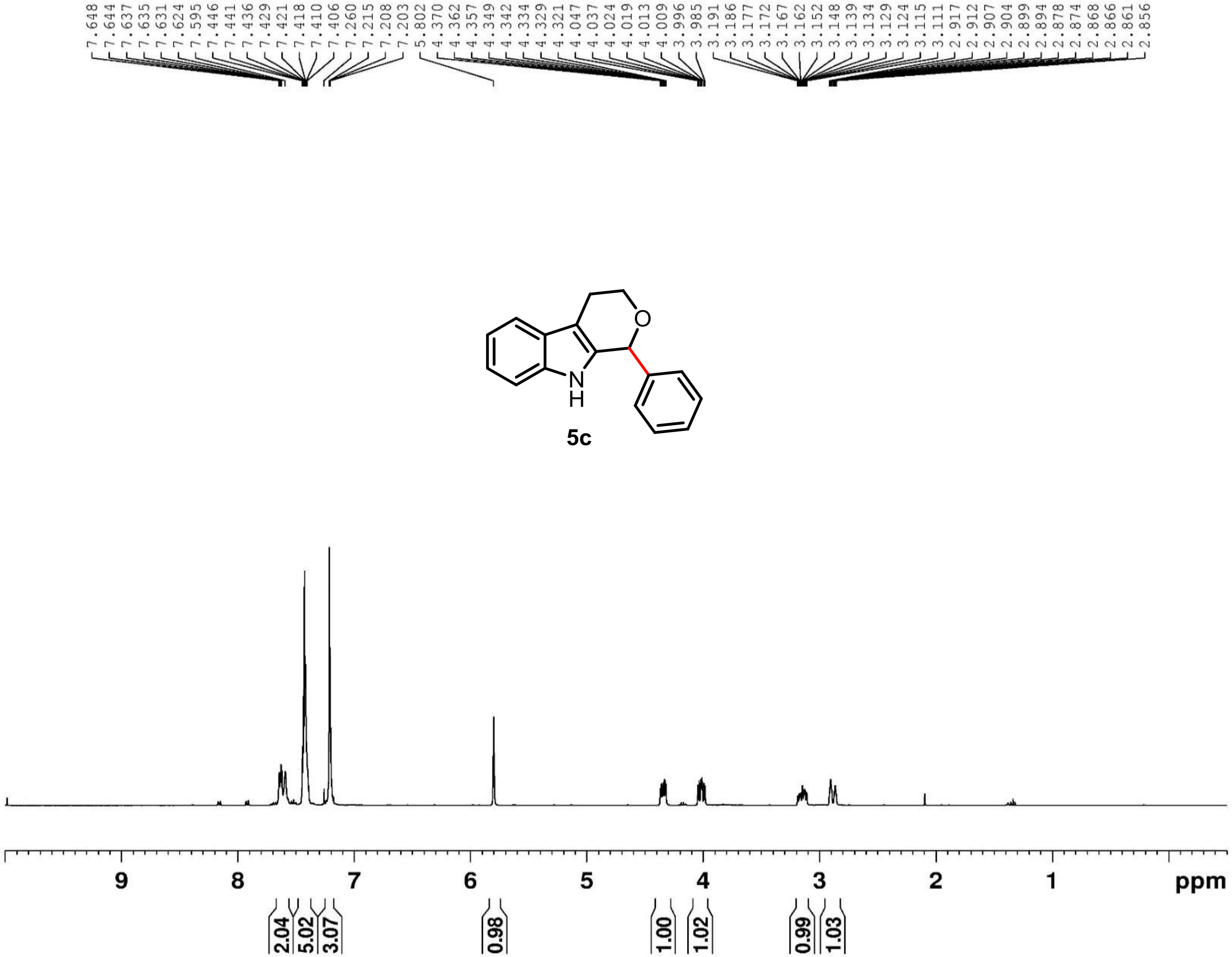


```
NAME          llx-3173-1
EXPNO                  1
PROCNO                 1
Date_           20190308
Time               15.14
INSTRUM            spect
PROBHD   5 mm PABBO BB/
PULPROG             zg30
TD                 65536
SOLVENT            CDCl3
NS                    12
DS                     2
SWH             8012.820 Hz
FIDRES          0.122266 Hz
AQ             4.0894966 sec
RG                  19.7
DW                62.400 usec
DE                  6.50 usec
TE                 294.6 K
D1            1.00000000 sec
TD0                    1

======== CHANNEL f1 ========
SFO1         400.1324710 MHz
NUC1                  1H
P1                 14.50 usec
SI                 65536
SF           400.1300099 MHz
WDW                   EM
SSB                    0
LB                  0.30 Hz
GB                     0
PC                  1.00
```

Supplementary Figure 120. ^{1}H-NMR of 5c

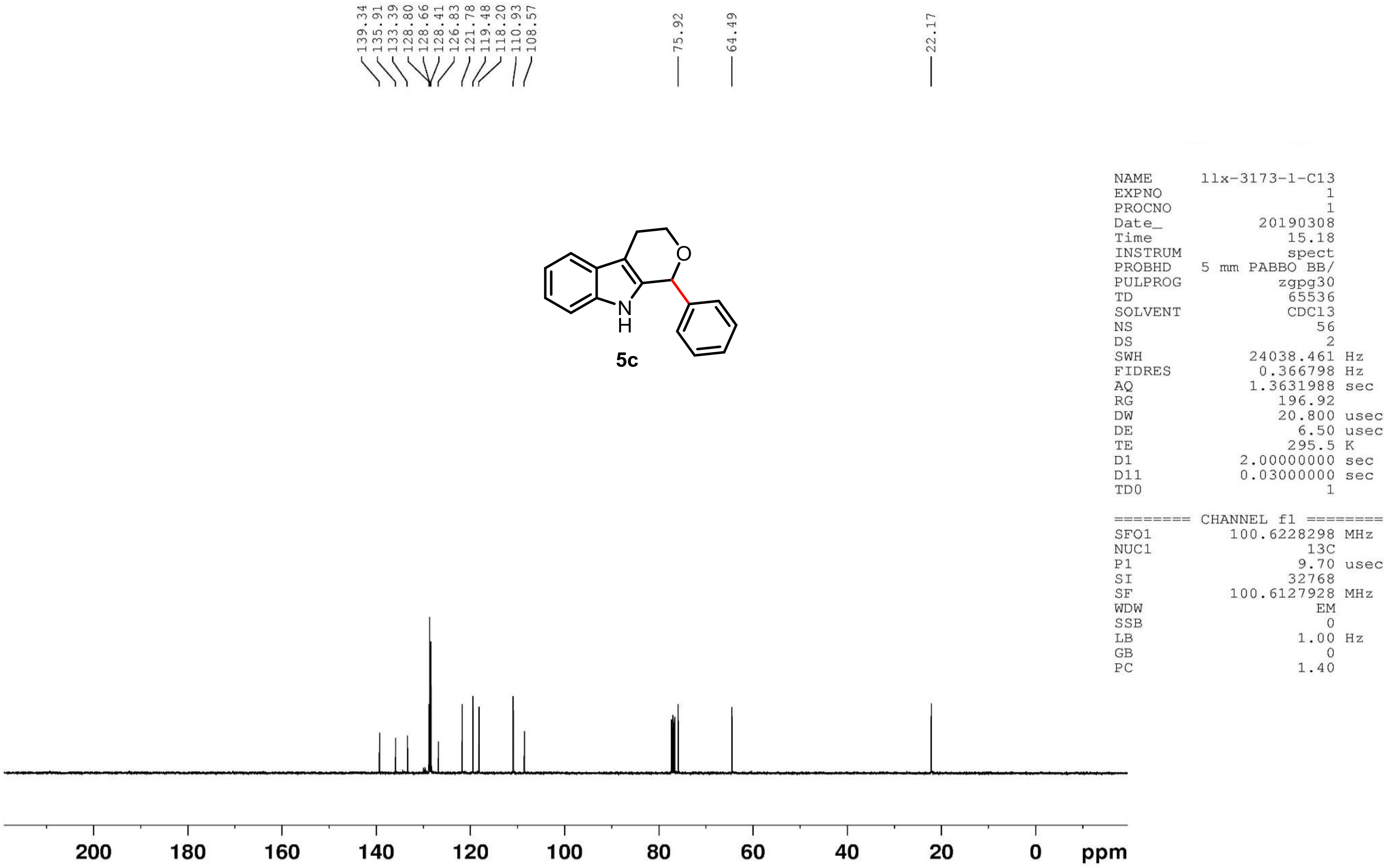

Supplementary Figure 121. ^{13}C-NMR of 5c

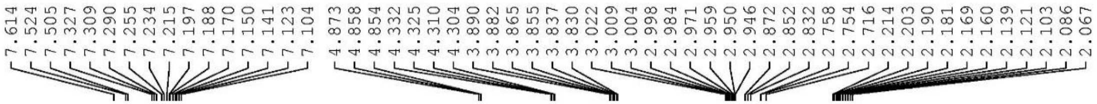

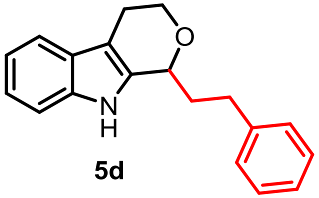

5d

```
NAME        llx-4026-1-H
EXPNO                  3
PROCNO                 1
Date_           20190309
Time               18.24
INSTRUM            spect
PROBHD   5 mm PABBO BB/
PULPROG             zg30
TD                 65536
SOLVENT            CDCl3
NS                    16
DS                     2
SWH             8012.820 Hz
FIDRES          0.122266 Hz
AQ             4.0894966 sec
RG                 82.92
DW                62.400 usec
DE                  6.50 usec
TE                 296.1 K
D1            1.00000000 sec
TD0                    1

======== CHANNEL f1 ========
SFO1         400.1324710 MHz
NUC1                  1H
P1                 14.50 usec
SI                 65536
SF           400.1300102 MHz
WDW                   EM
SSB                    0
LB                  0.30 Hz
GB                     0
PC                  1.00
```

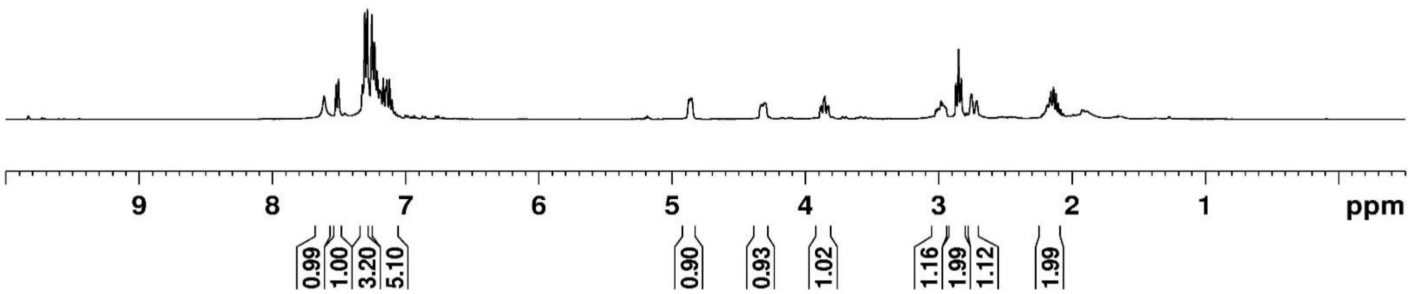

Supplementary Figure 122. ^{1}H-NMR of 5d

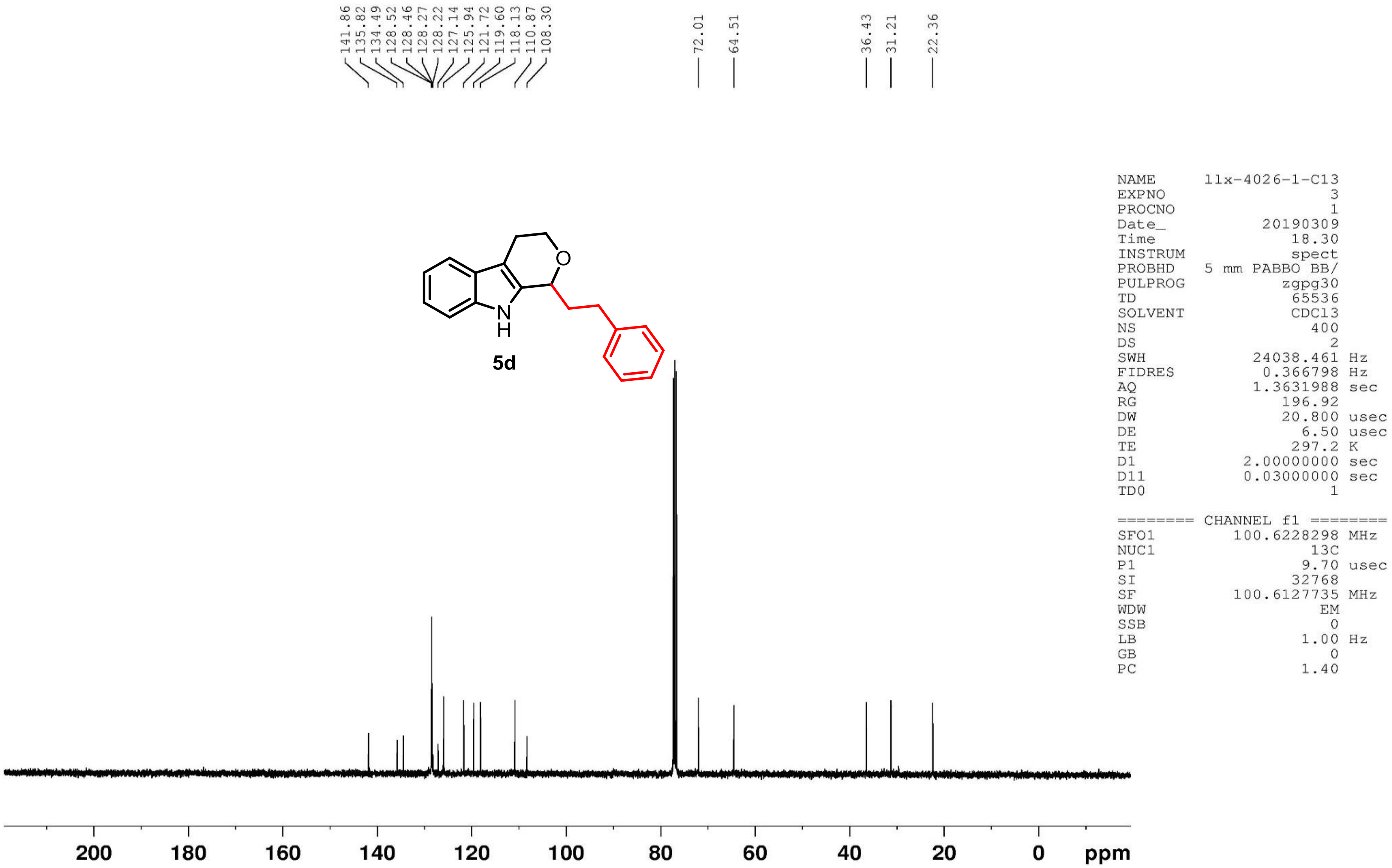

Supplementary Figure 123. ^{13}C-NMR of 5d

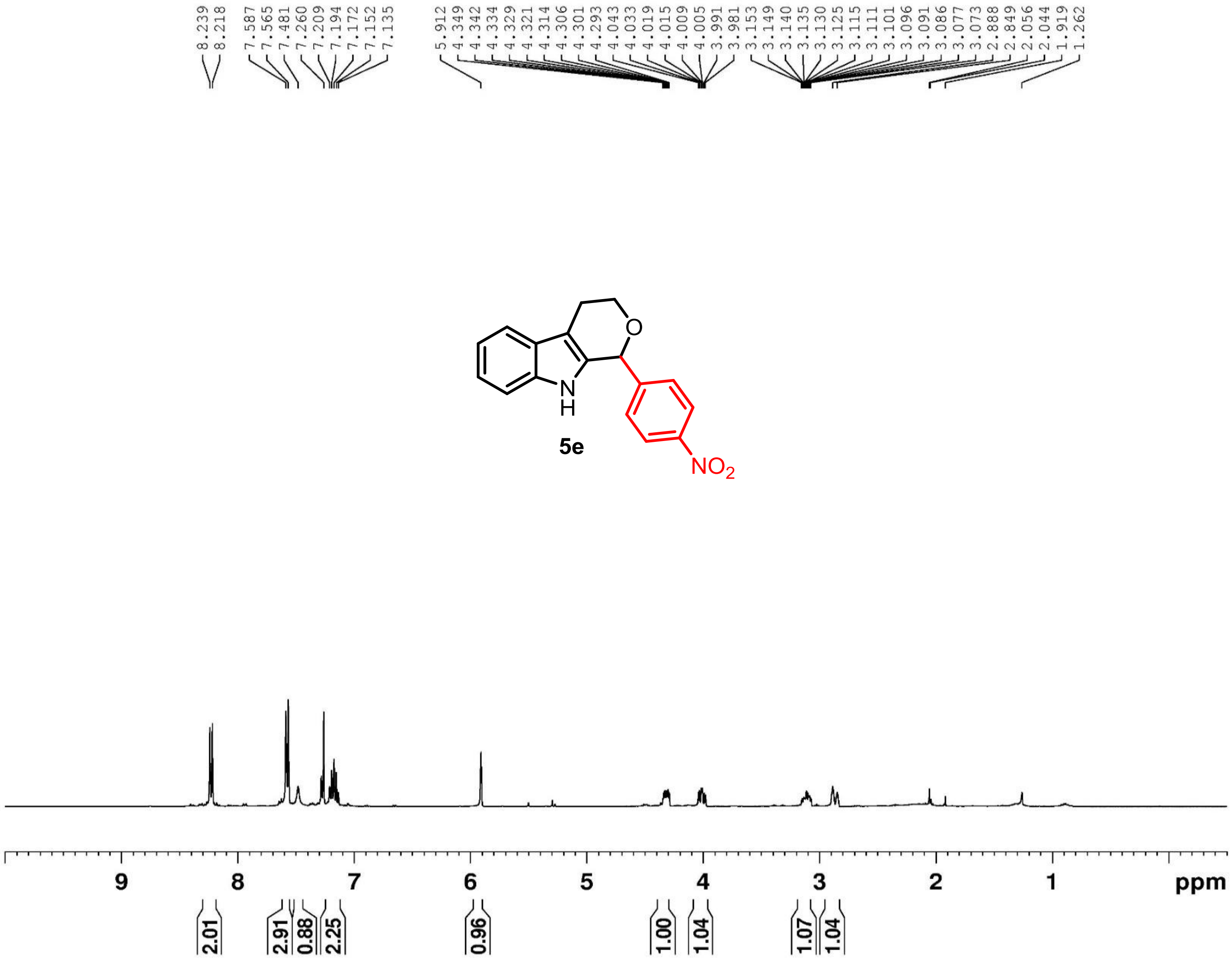


```
NAME           zz-10-135-H
EXPNO                    1
PROCNO                   1
Date_             20190308
Time                 22.17
INSTRUM              spect
PROBHD      5 mm PABBO BB-
PULPROG               zg30
TD                   65536
SOLVENT              CDCl3
NS                      16
DS                       2
SWH               8223.685 Hz
FIDRES            0.125483 Hz
AQ               3.9846387 sec
RG                     456
DW                  60.800 usec
DE                    6.00 usec
TE                   294.7 K
D1              1.00000000 sec
TD0                      1

======== CHANNEL f1 ========
NUC1                    1H
P1                   15.80 usec
PL1                  -1.00 dB
PL1W           12.17476940 W
SFO1           400.1324710 MHz
SI                   32768
SF             400.1300093 MHz
WDW                     EM
SSB                      0
LB                    0.30 Hz
GB                       0
PC                    1.00
```

Supplementary Figure 124. ^{1}H-NMR of 5e

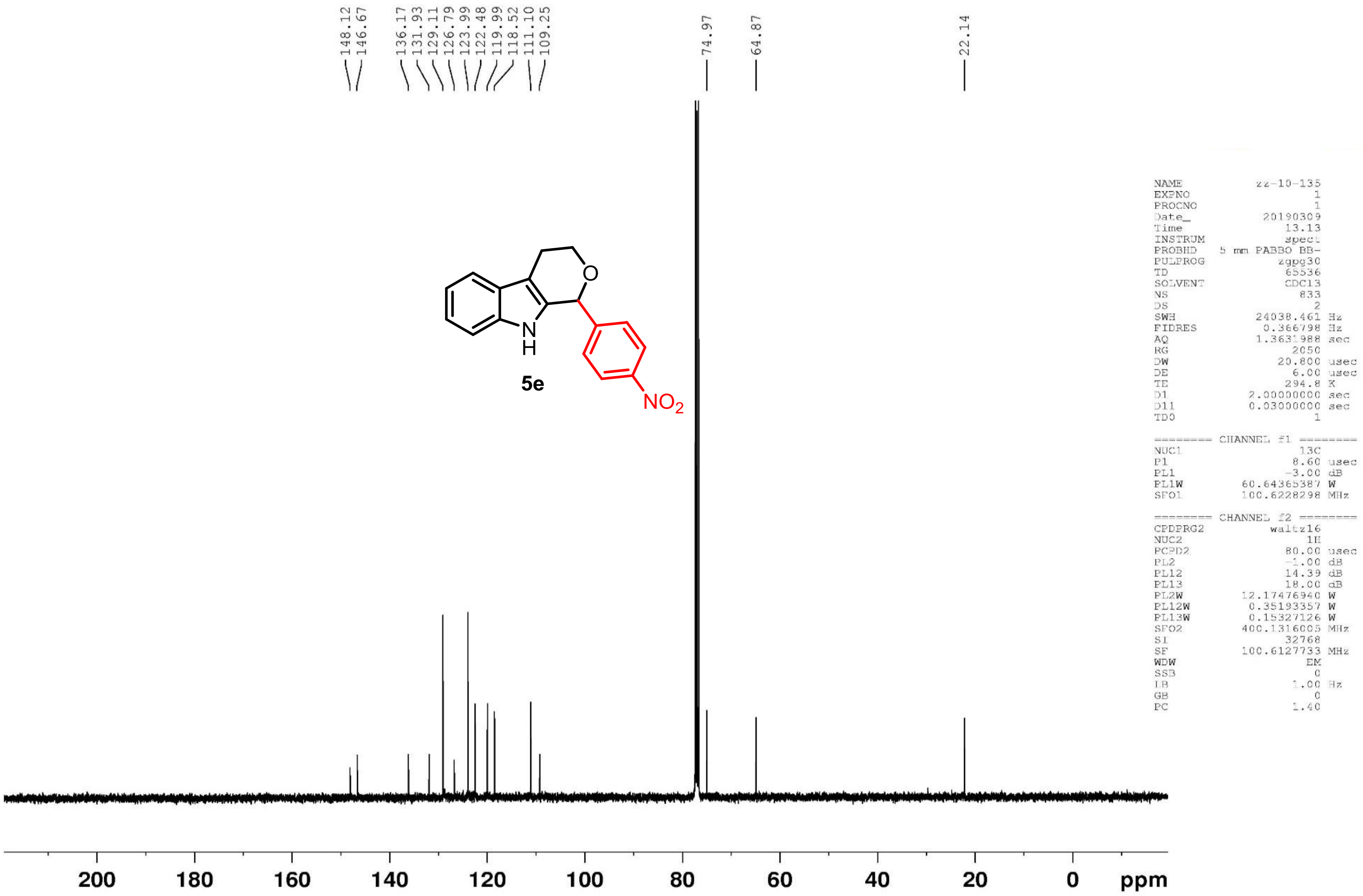

Supplementary Figure 125. ^{13}C-NMR of 5e

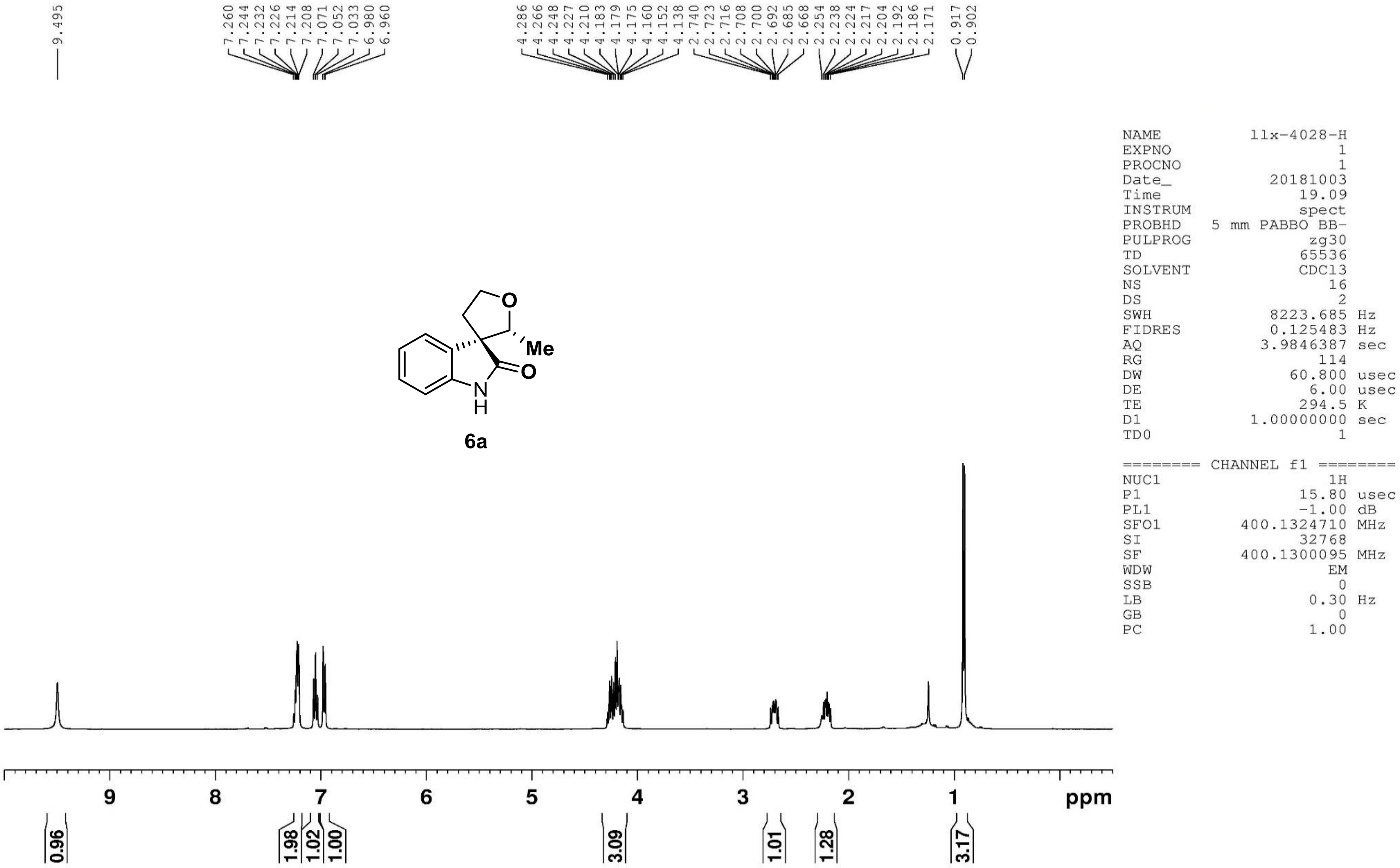

Supplementary Figure 126. ^{1}H-NMR of 6a

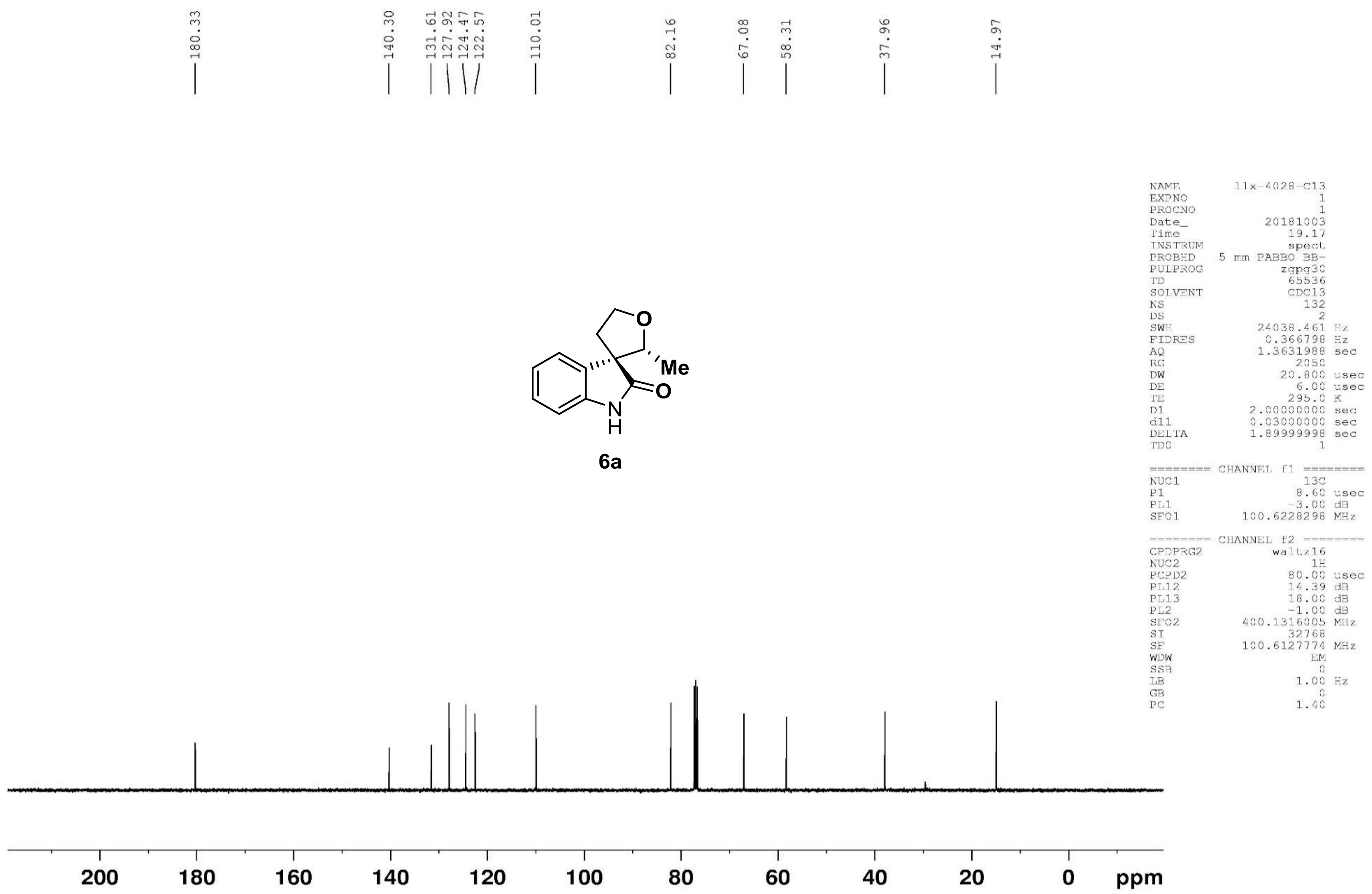

Supplementary Figure 127. ^{13}**C-NMR of 6a**

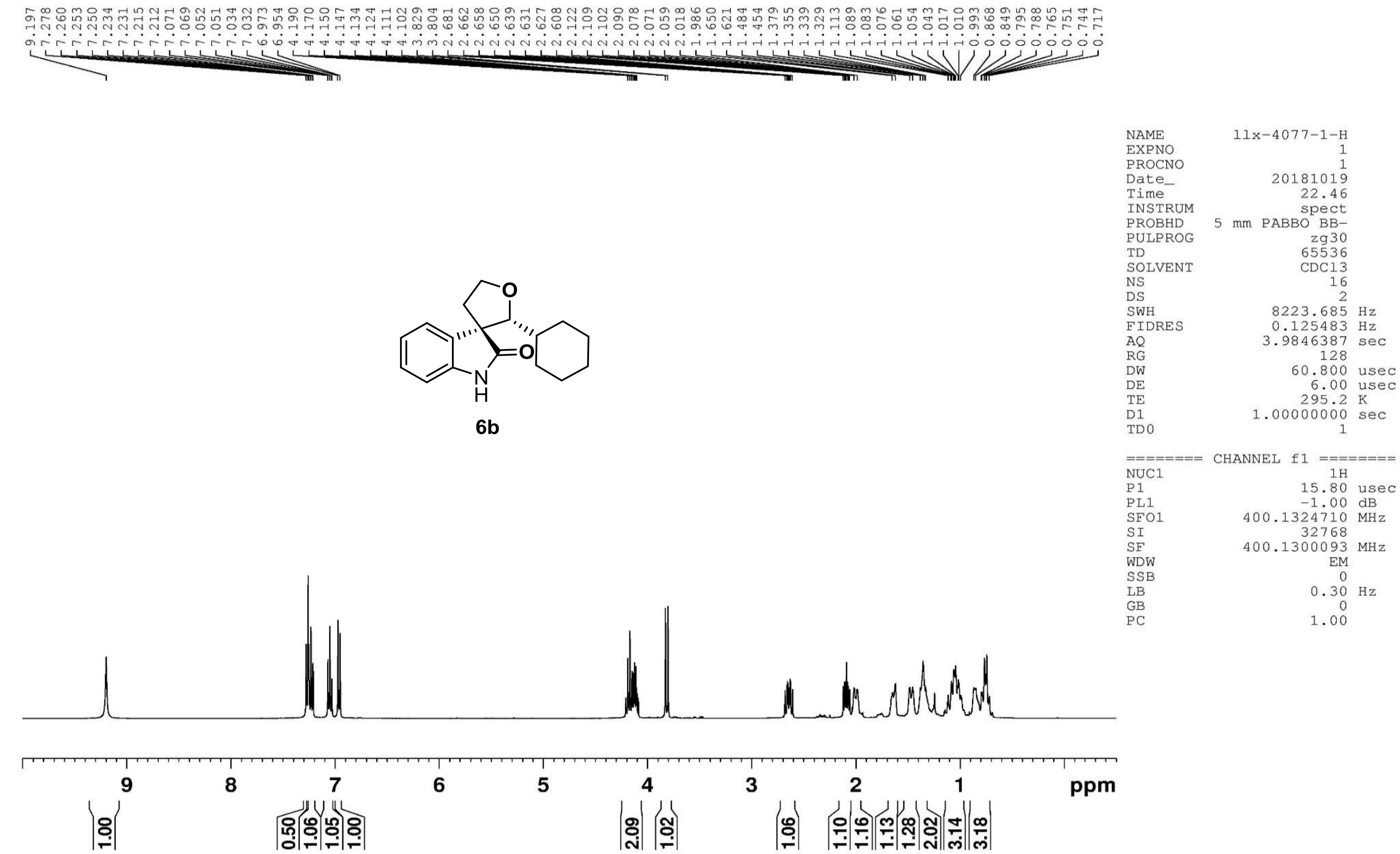

Supplementary Figure 128. ^{1}H-NMR of 6b

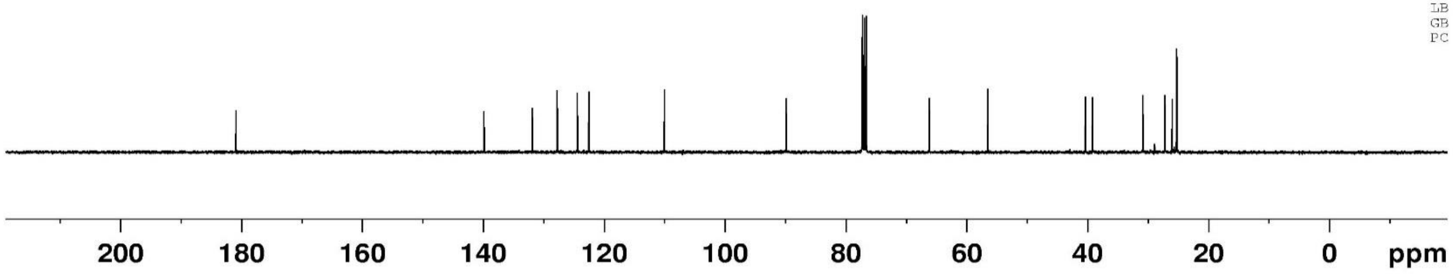

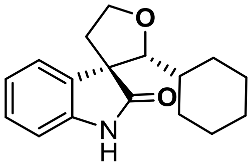

6b

```
NAME       11x-4077-1-C13
EXPNO                   1
PROCNO                  1
Date_            20181020
Time                 1.52
INSTRUM             spect
PROBHD    5 mm PABBO BB-
PULPROG           zgpg30
TD                 65536
SOLVENT            CDCl3
NS                   400
DS                     2
SWH            24038.461 Hz
FIDRES          0.366798 Hz
AQ             1.3631988 sec
RG                  2050
DW                20.800 usec
DE                  6.00 usec
TE                 297.3 K
D1            2.00000000 sec
d11           0.03000000 sec
DELTA         1.89999998 sec
TD0                    1

======== CHANNEL f1 ========
NUC1                 13C
P1                  8.60 usec
PL1                -3.00 dB
SFO1        100.6228298 MHz

-------- CHANNEL f2 --------
CPDPRG2          waltz16
NUC2                  1H
PCPD2              80.00 usec
PL12               14.39 dB
PL13               18.00 dB
PL2                -1.00 dB
SFO2        400.1316005 MHz
SI                 32768
SF          100.6127735 MHz
WDW                   EM
SSB                    0
LB                  1.00 Hz
GB                     0
PC                  1.40
```

Supplementary Figure 129. ^{13}C-NMR of 6b

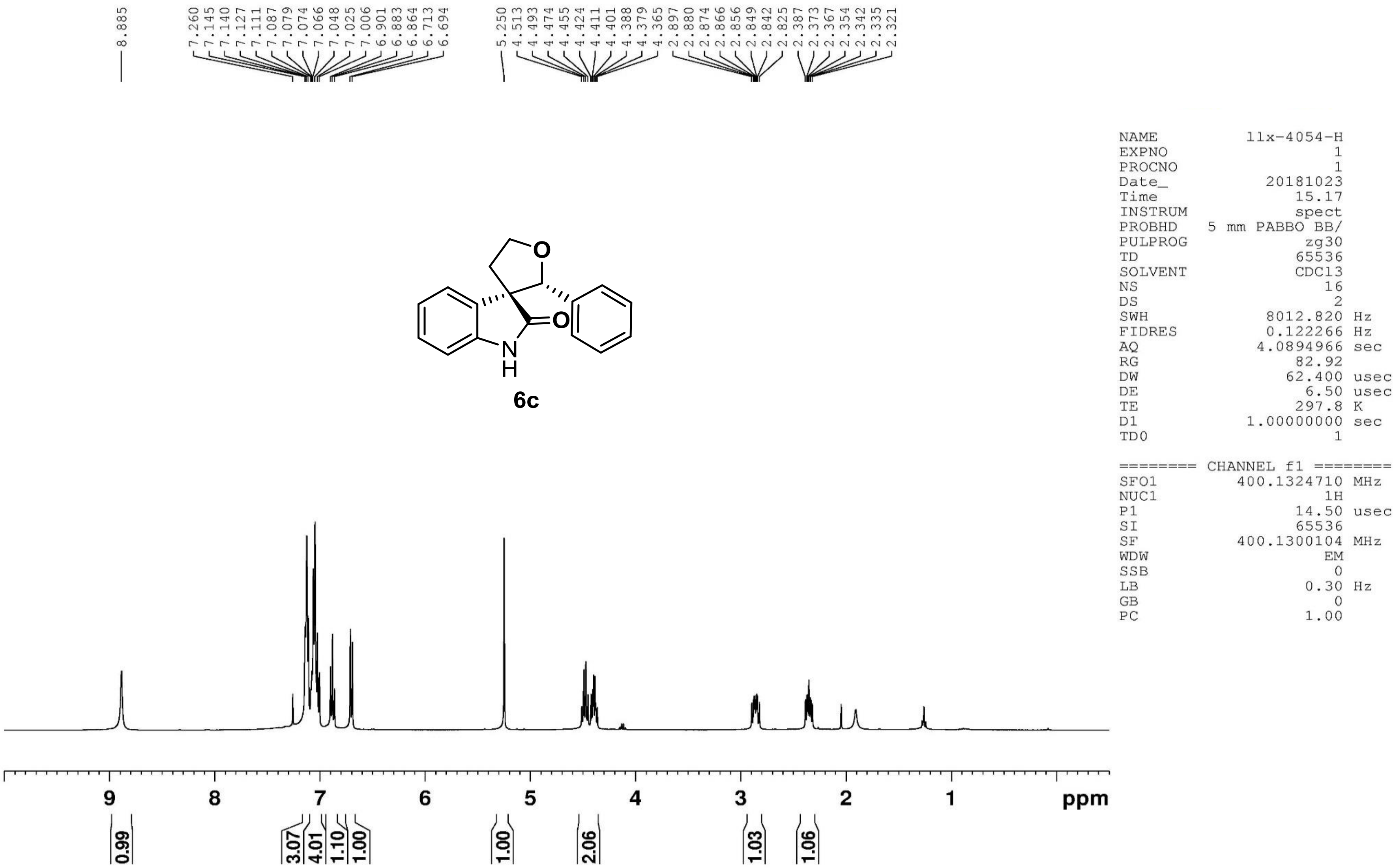

Supplementary Figure 130. ^{1}H-NMR of 6c

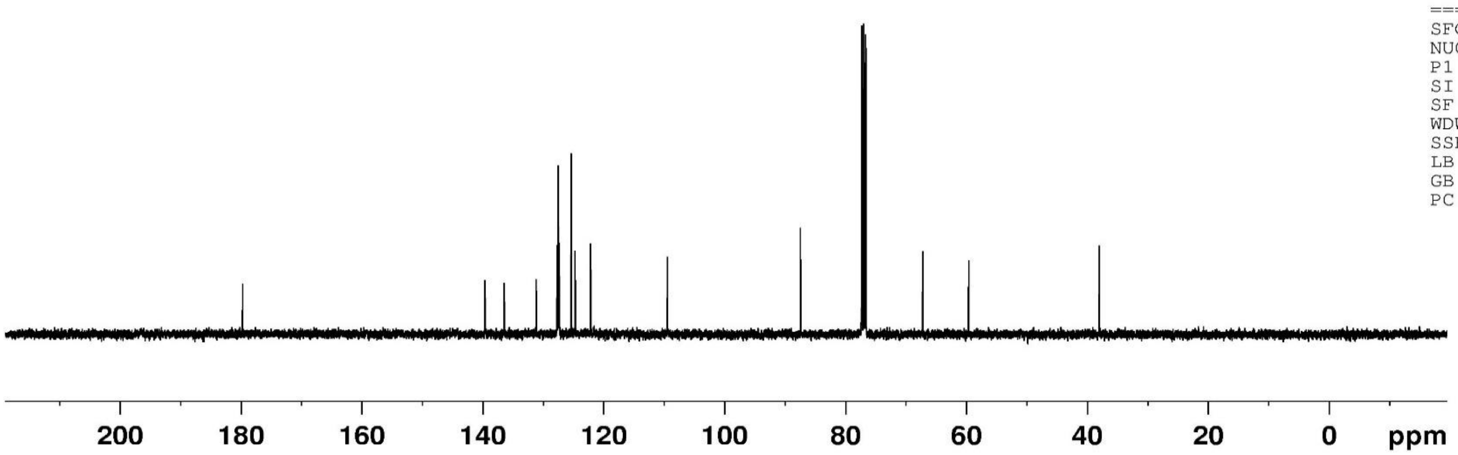

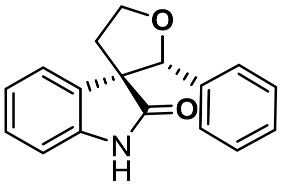

6c

NAME	llx-4054-C13
EXPNO	1
PROCNO	1
Date_	20181023
Time	15.22
INSTRUM	spect
PROBHD	5 mm PABBO BB/
PULPROG	zgpg30
TD	65536
SOLVENT	CDCl3
NS	120
DS	2
SWH	24038.461 Hz
FIDRES	0.366798 Hz
AQ	1.3631988 sec
RG	196.92
DW	20.800 usec
DE	6.50 usec
TE	298.4 K
D1	2.00000000 sec
D11	0.03000000 sec
TD0	1
======== CHANNEL f1 ========	
SFO1	100.6228298 MHz
NUC1	13C
P1	9.70 usec
SI	32768
SF	100.6127751 MHz
WDW	EM
SSB	0
LB	1.00 Hz
GB	0
PC	1.40

Supplementary Figure 131. ^{13}C-NMR of 6c

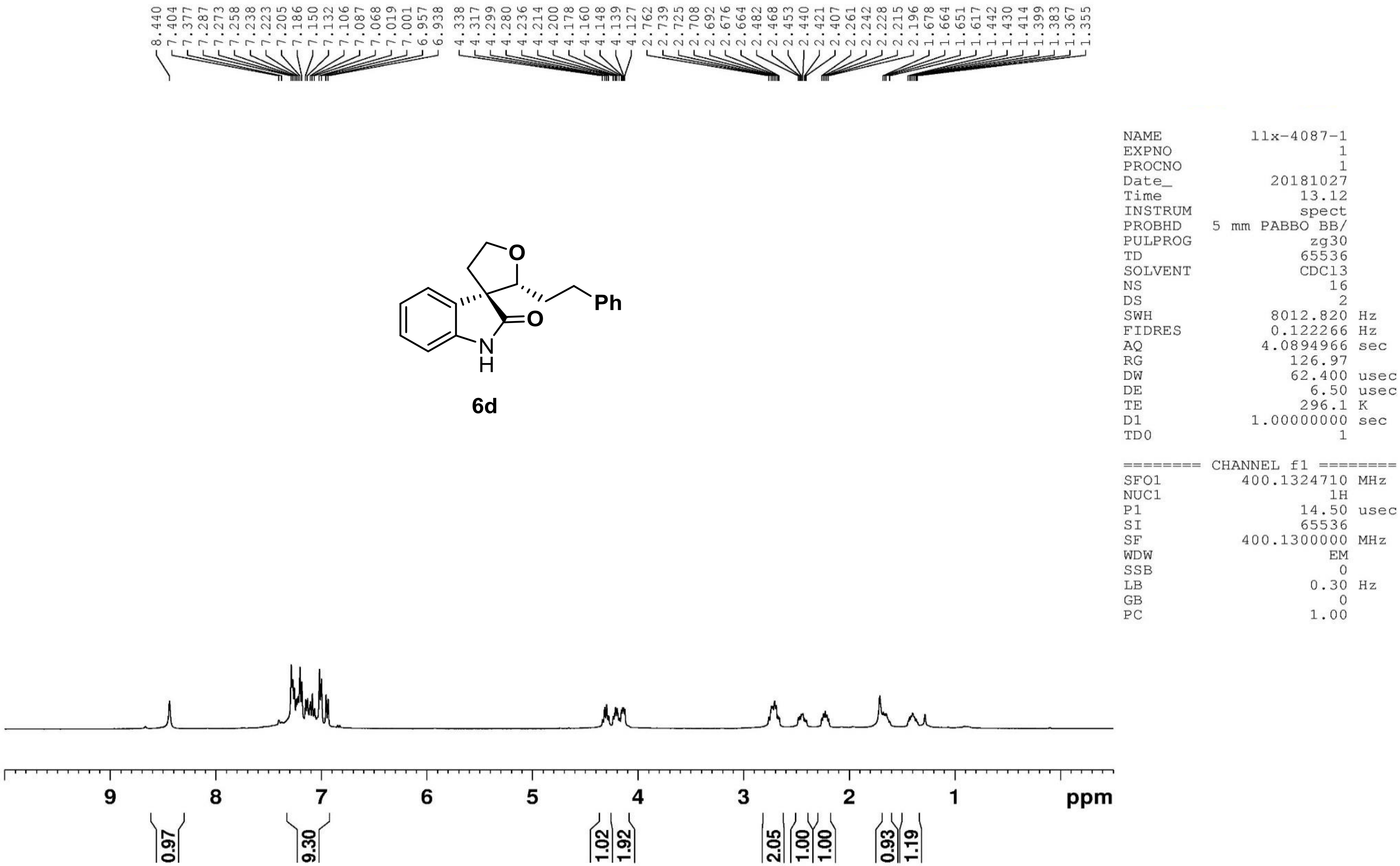

Supplementary Figure 132. ^{1}H-NMR of 6d

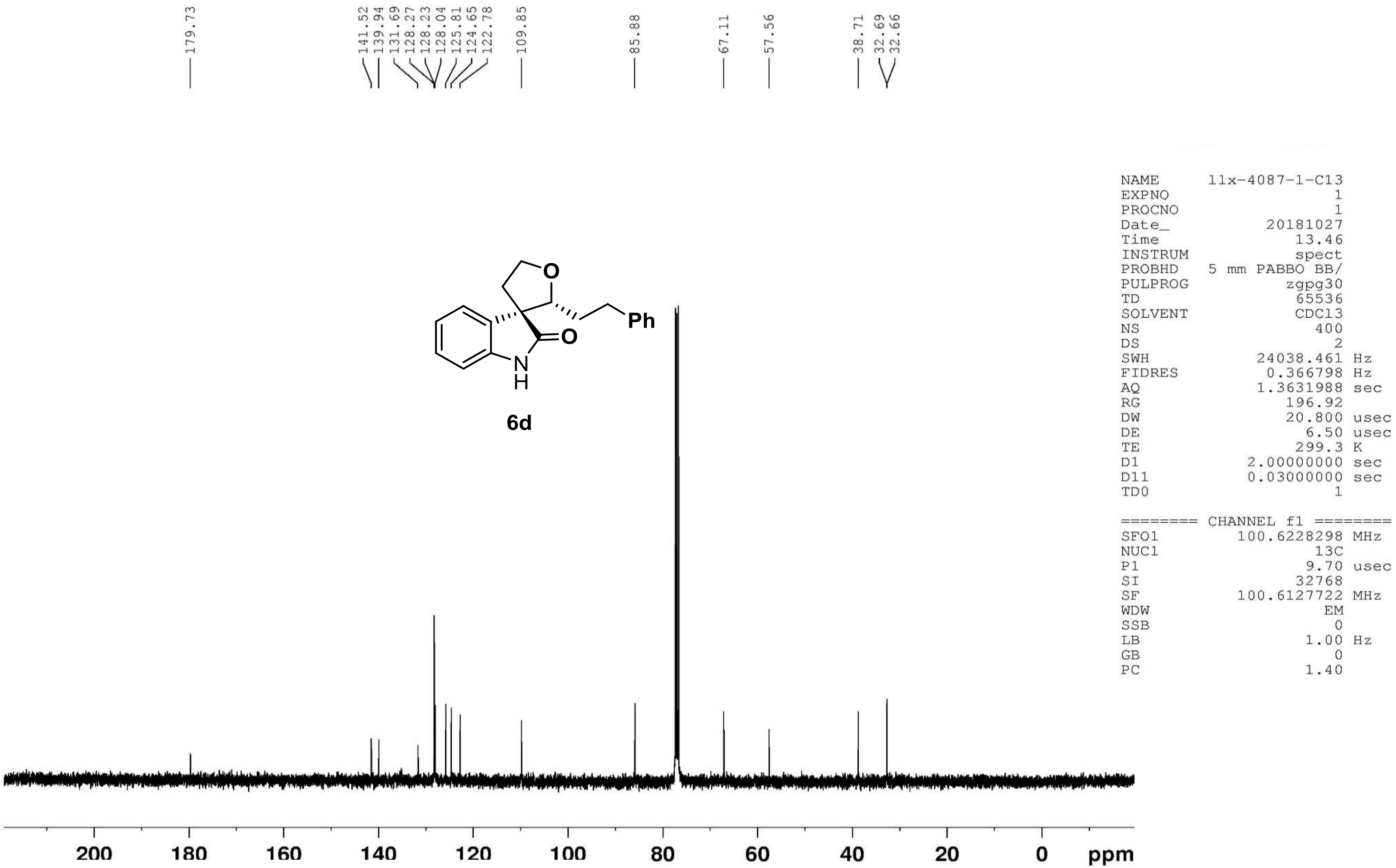

Supplementary Figure 133. ^{13}C-NMR of 6d

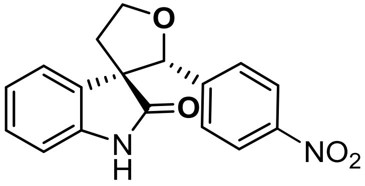

6e

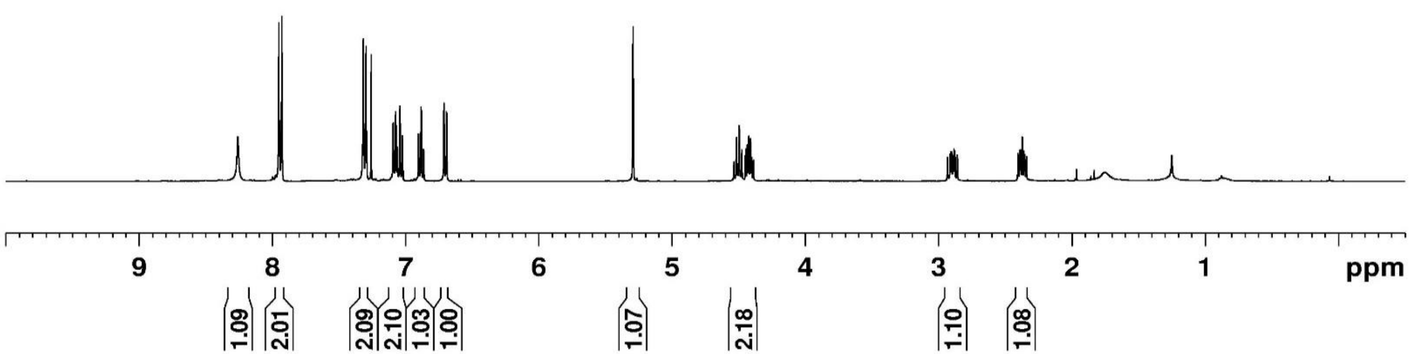


```
[illegible]
EXPNO                   2
PROCNO                  1
Date_            20190313
Time                23.38
INSTRUM             spect
PROBHD   5 mm PABBO BB/
PULPROG              zg30
TD                  65536
SOLVENT             CDCl3
NS                     16
DS                      2
SWH              8012.820 Hz
FIDRES           0.122266 Hz
AQ              4.0894966 sec
RG                 112.31
DW                 62.400 usec
DE                   6.50 usec
TE                  296.3 K
D1             1.00000000 sec
TD0                     1

======== CHANNEL f1 ========
SFO1          400.1324710 MHz
NUC1                   1H
P1                  14.50 usec
SI                  65536
SF            400.1300101 MHz
WDW                    EM
SSB                     0
LB                   0.30 Hz
GB                      0
PC                   1.00
```

Supplementary Figure 134. ^{1}H-NMR of 6e

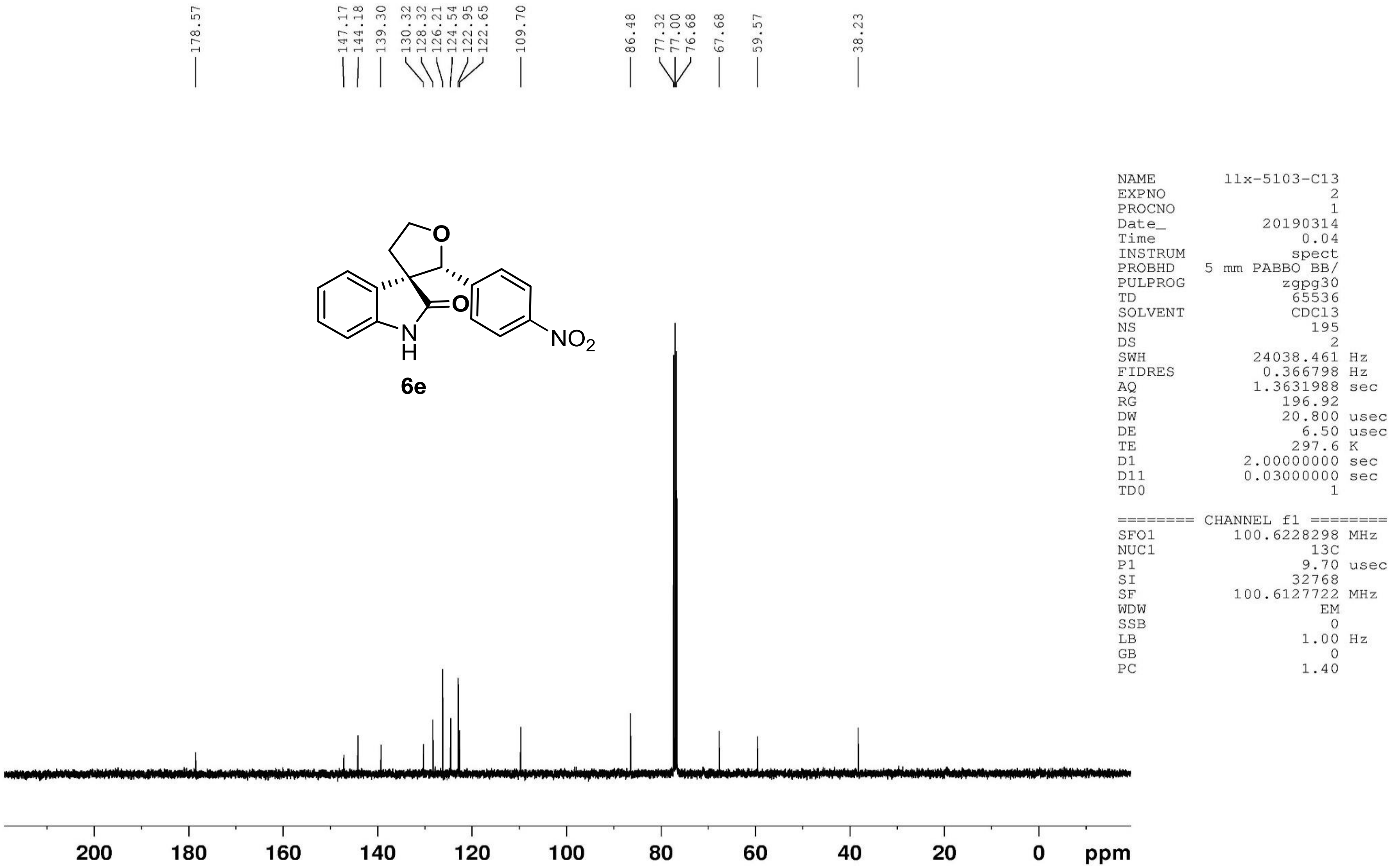

NAME	llx-5103-C13	
EXPNO	2	
PROCNO	1	
Date_	20190314	
Time	0.04	
INSTRUM	spect	
PROBHD	5 mm PABBO BB/	
PULPROG	zgpg30	
TD	65536	
SOLVENT	CDCl3	
NS	195	
DS	2	
SWH	24038.461	Hz
FIDRES	0.366798	Hz
AQ	1.3631988	sec
RG	196.92	
DW	20.800	usec
DE	6.50	usec
TE	297.6	K
D1	2.00000000	sec
D11	0.03000000	sec
TD0	1	
======== CHANNEL f1 ========		
SFO1	100.6228298	MHz
NUC1	13C	
P1	9.70	usec
SI	32768	
SF	100.6127722	MHz
WDW	EM	
SSB	0	
LB	1.00	Hz
GB	0	
PC	1.40	

Supplementary Figure 135. ^{13}C-NMR of 6e

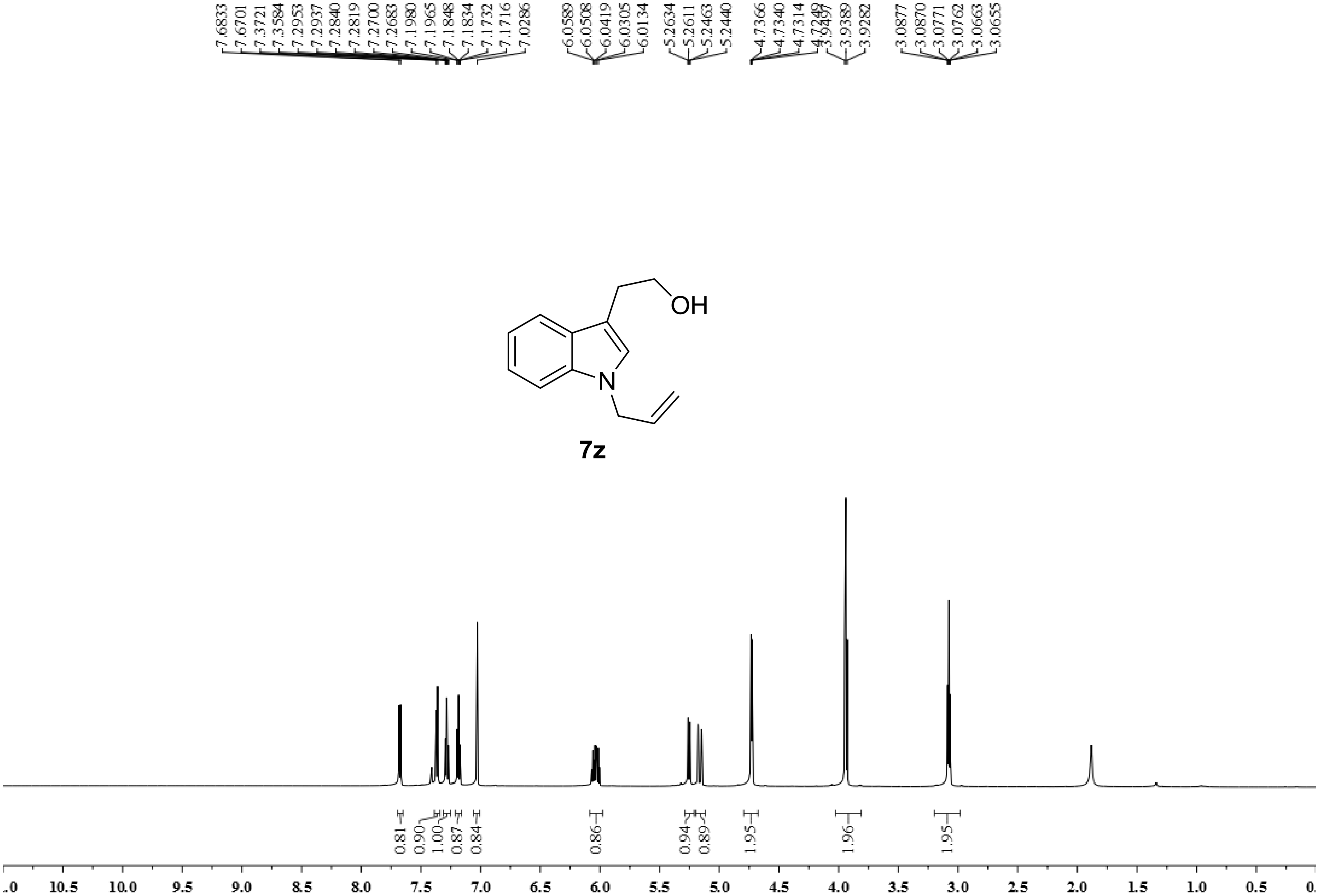

Supplementary Figure 136. ^{1}H-NMR of 7z

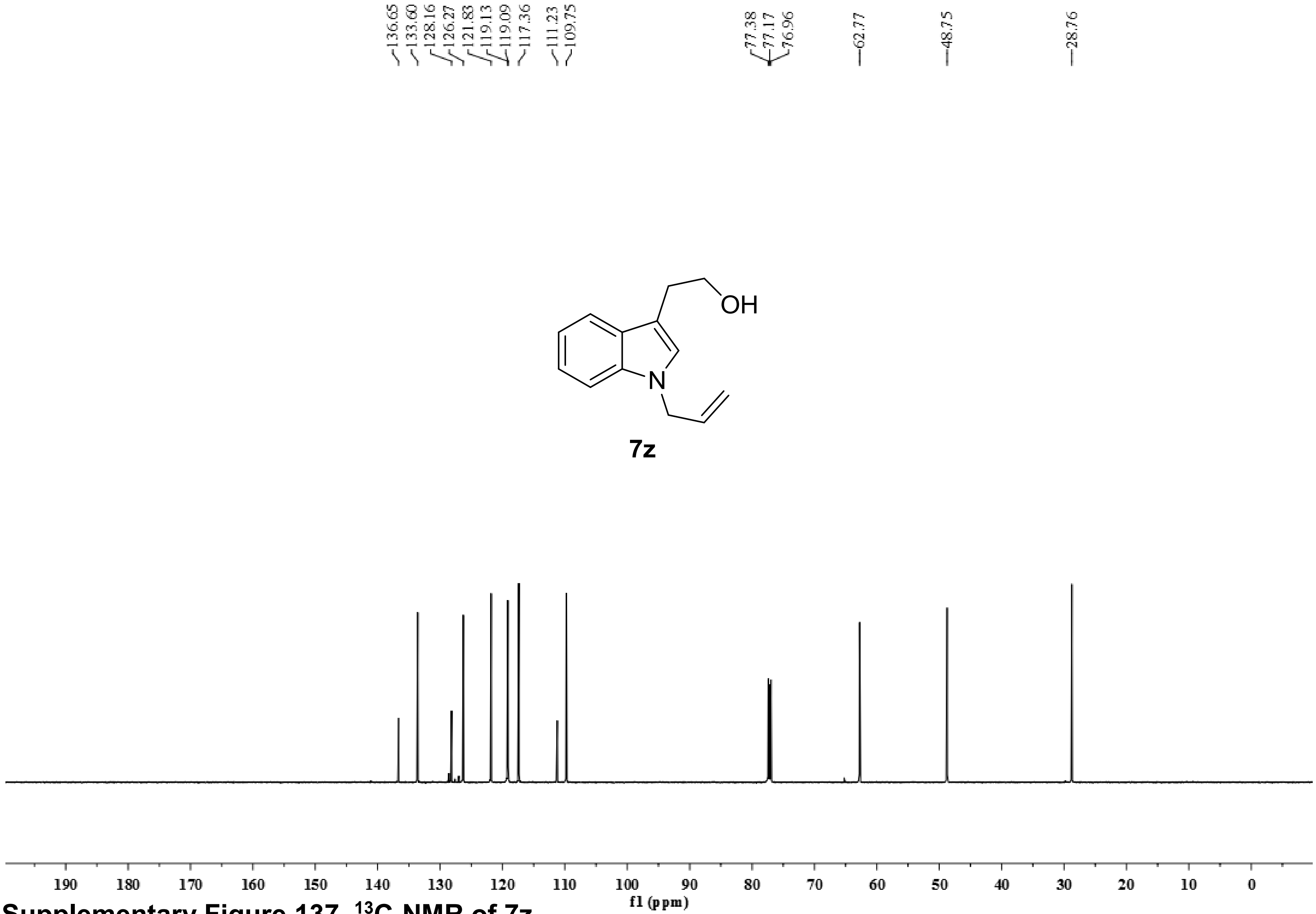

Supplementary Figure 137. ^{13}C-NMR of 7z

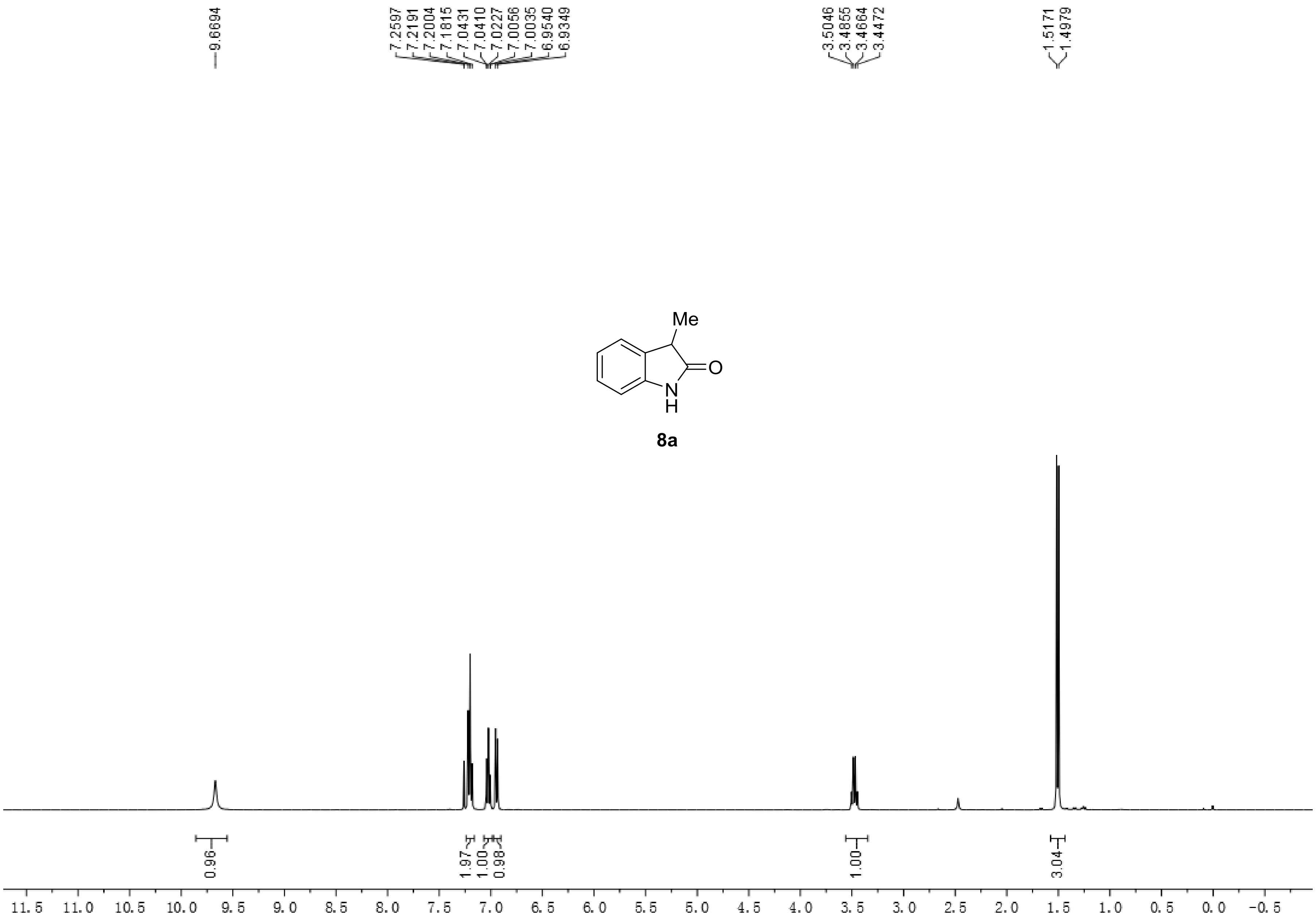

Supplementary Figure 138. ^{1}H-NMR of 8a

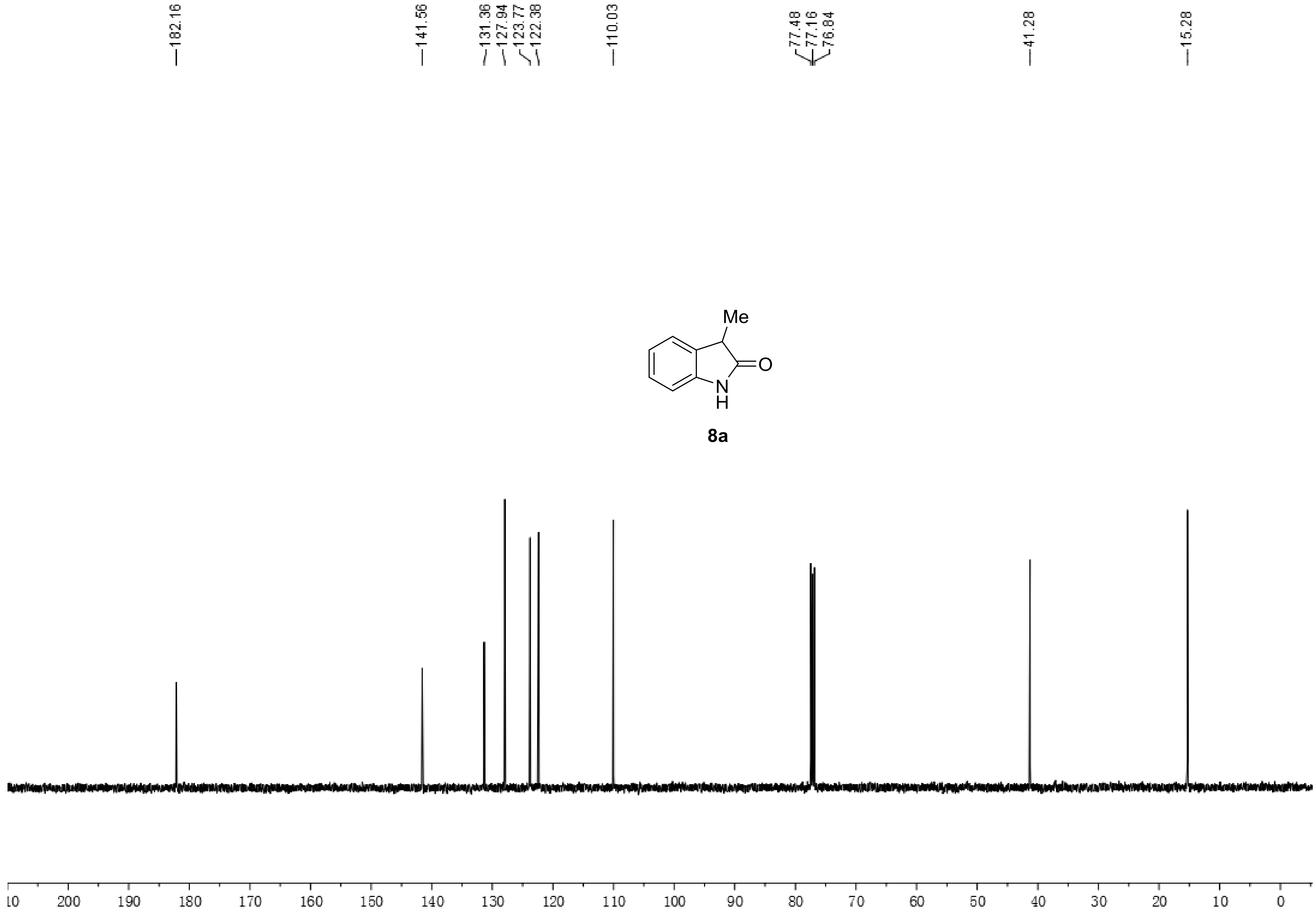

Supplementary Figure 139. ^{13}C-NMR of 8a

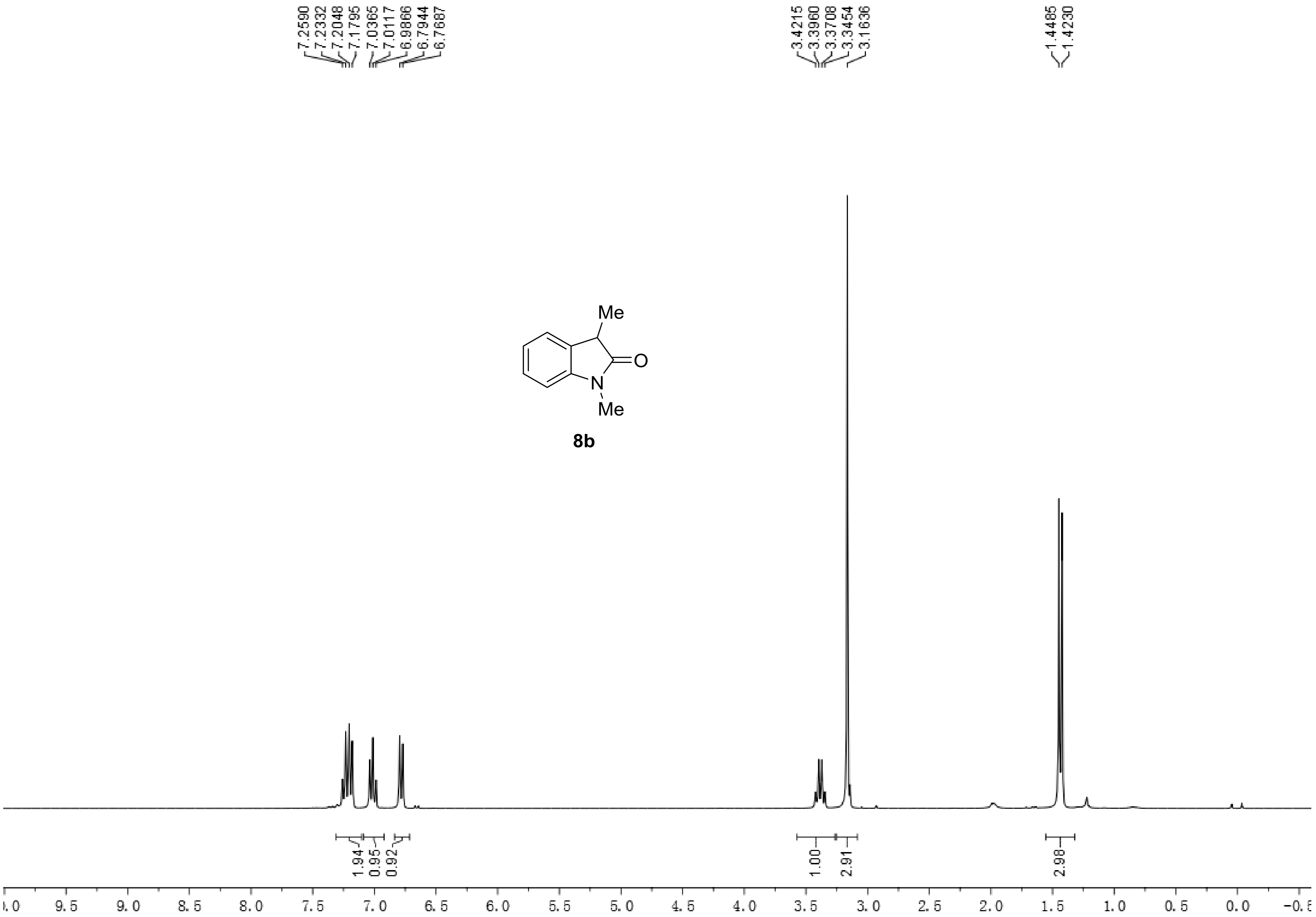

Supplementary Figure 140. ^{1}H-NMR of 8b

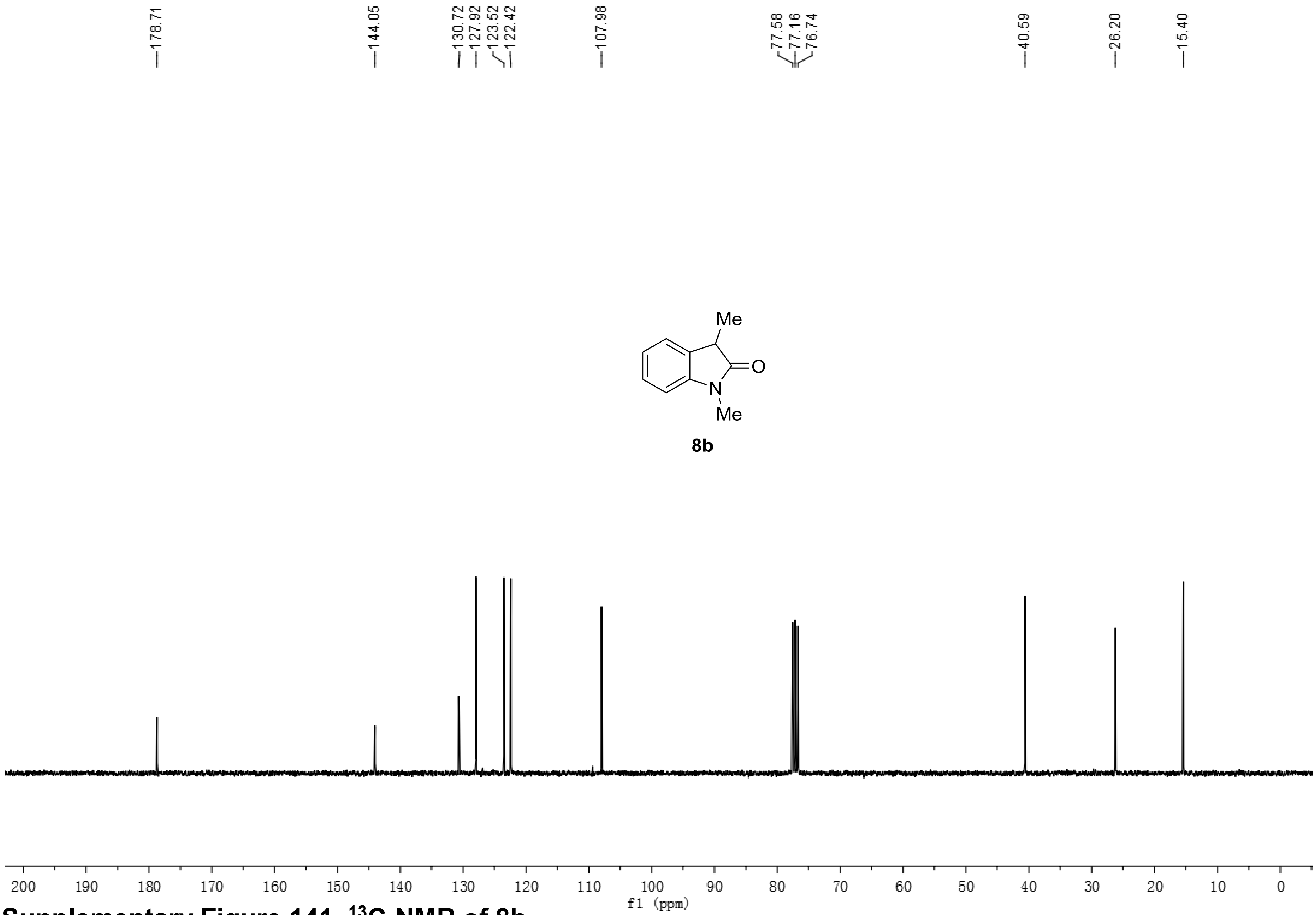

Supplementary Figure 141. ^{13}**C-NMR of 8b**

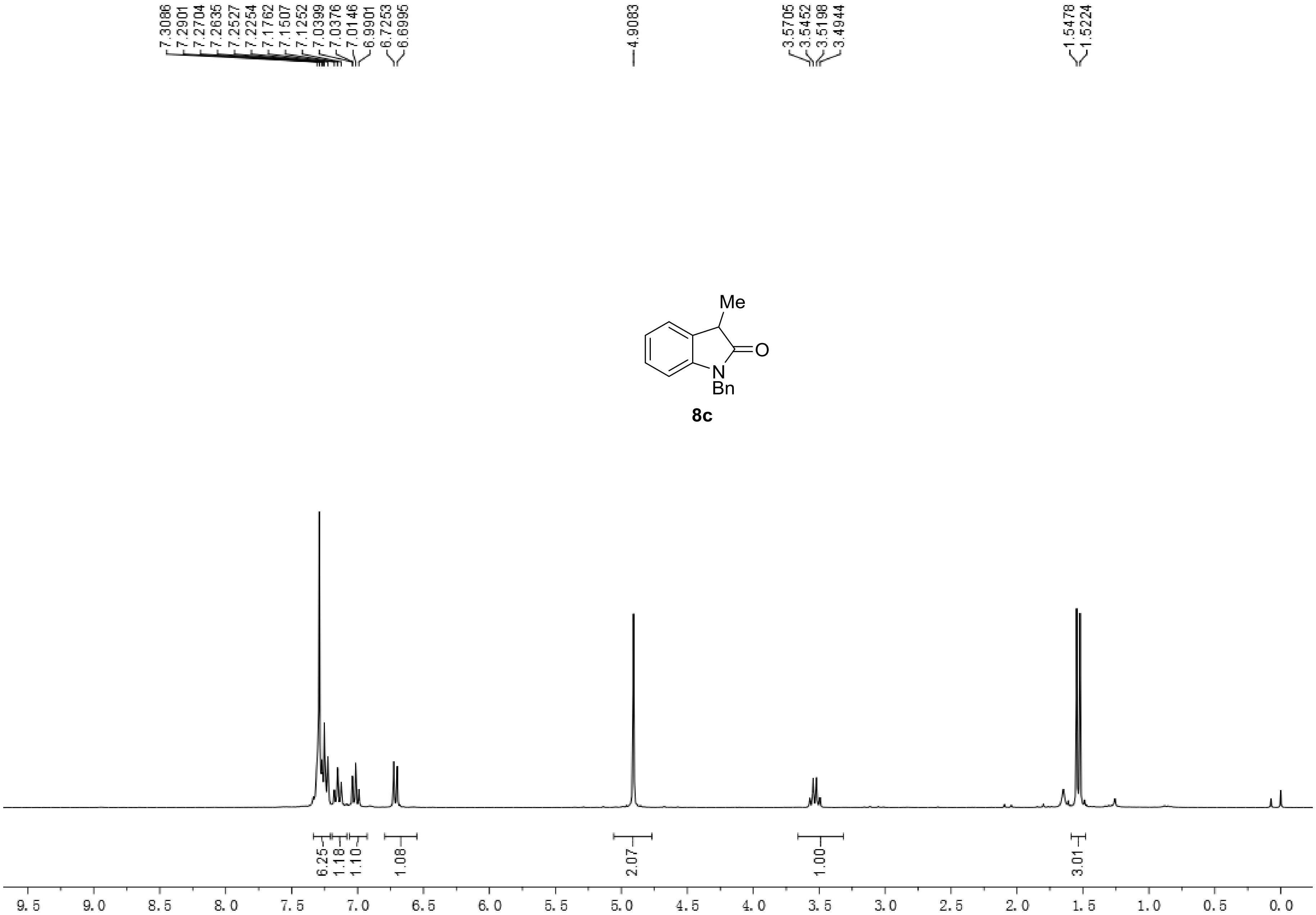

8c

Supplementary Figure 142. ^{1}H-NMR of 8c

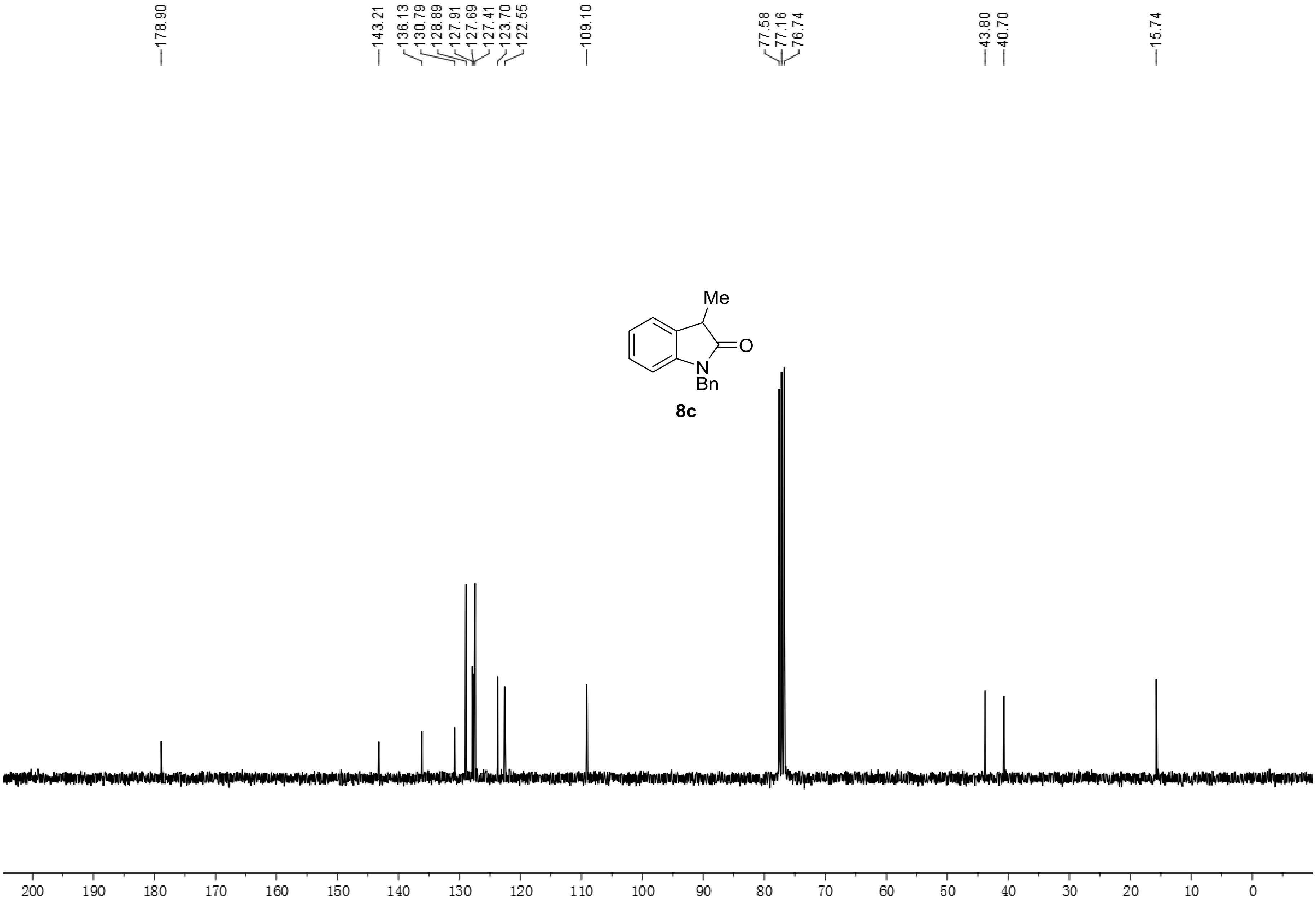

Supplementary Figure 143. ^{13}C-NMR of 8c

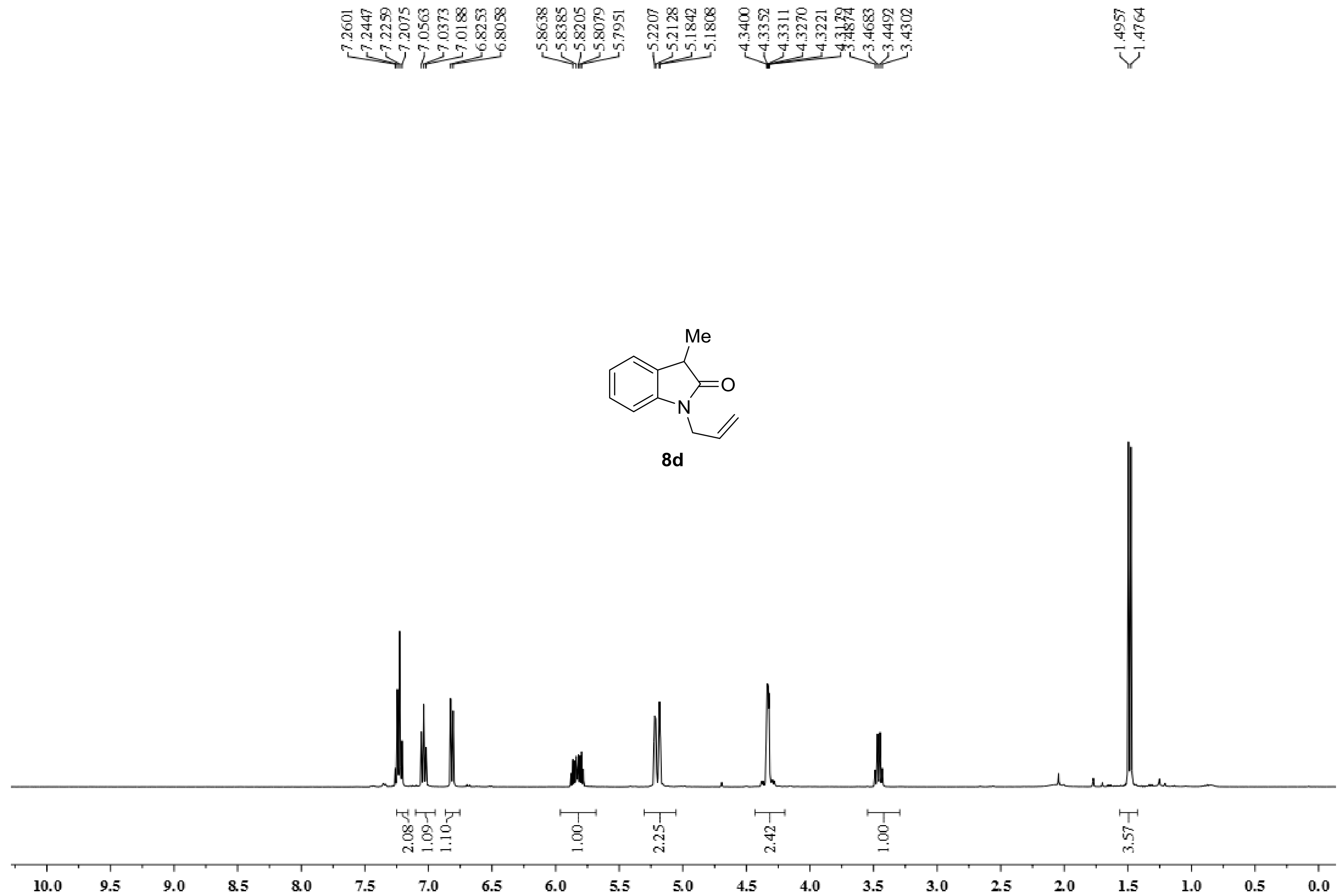

Supplementary Figure 144. ^{1}H-NMR of 8d

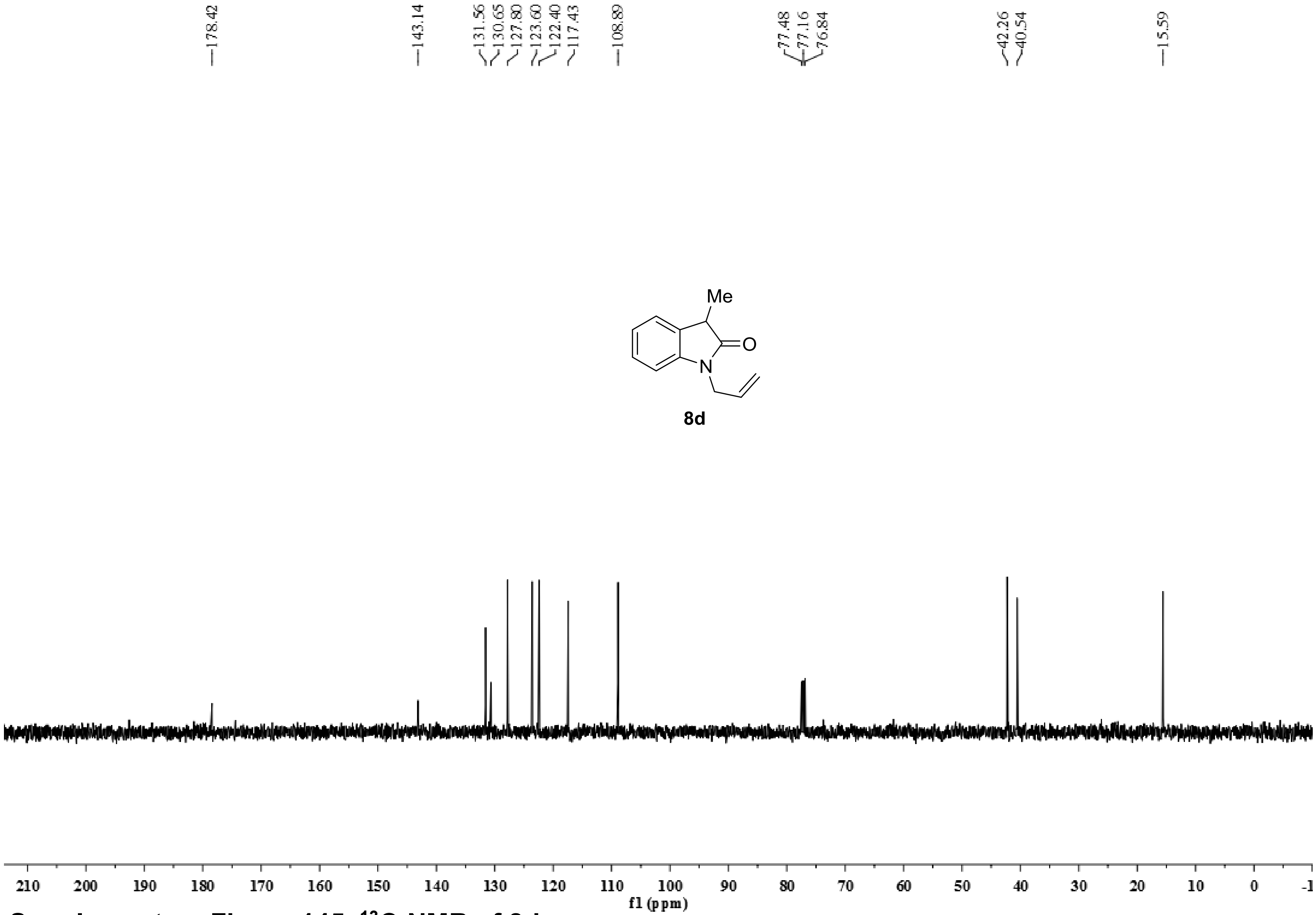

Supplementary Figure 145. ^{13}C-NMR of 8d

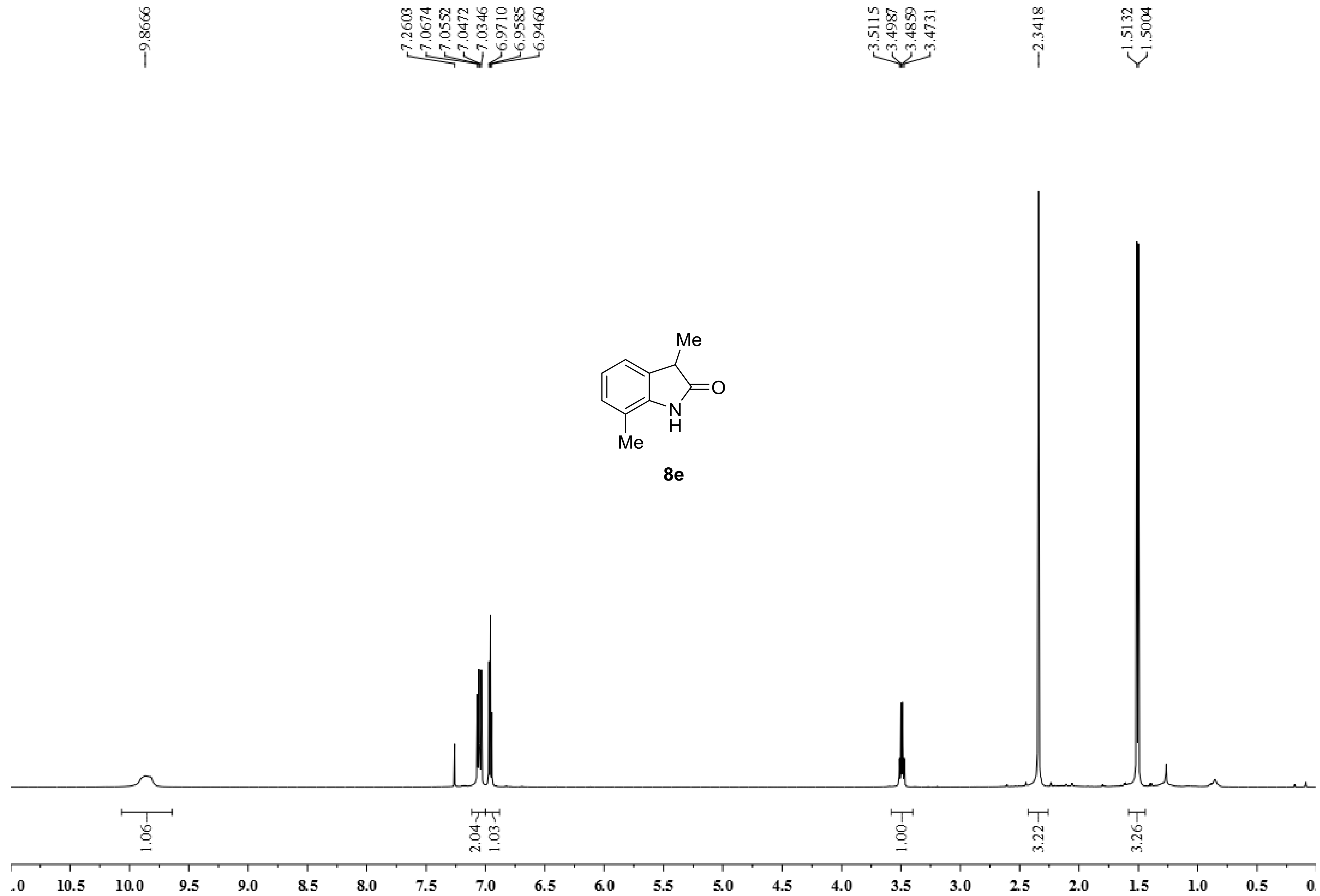

Supplementary Figure 146. ^{1}H-NMR of 8e

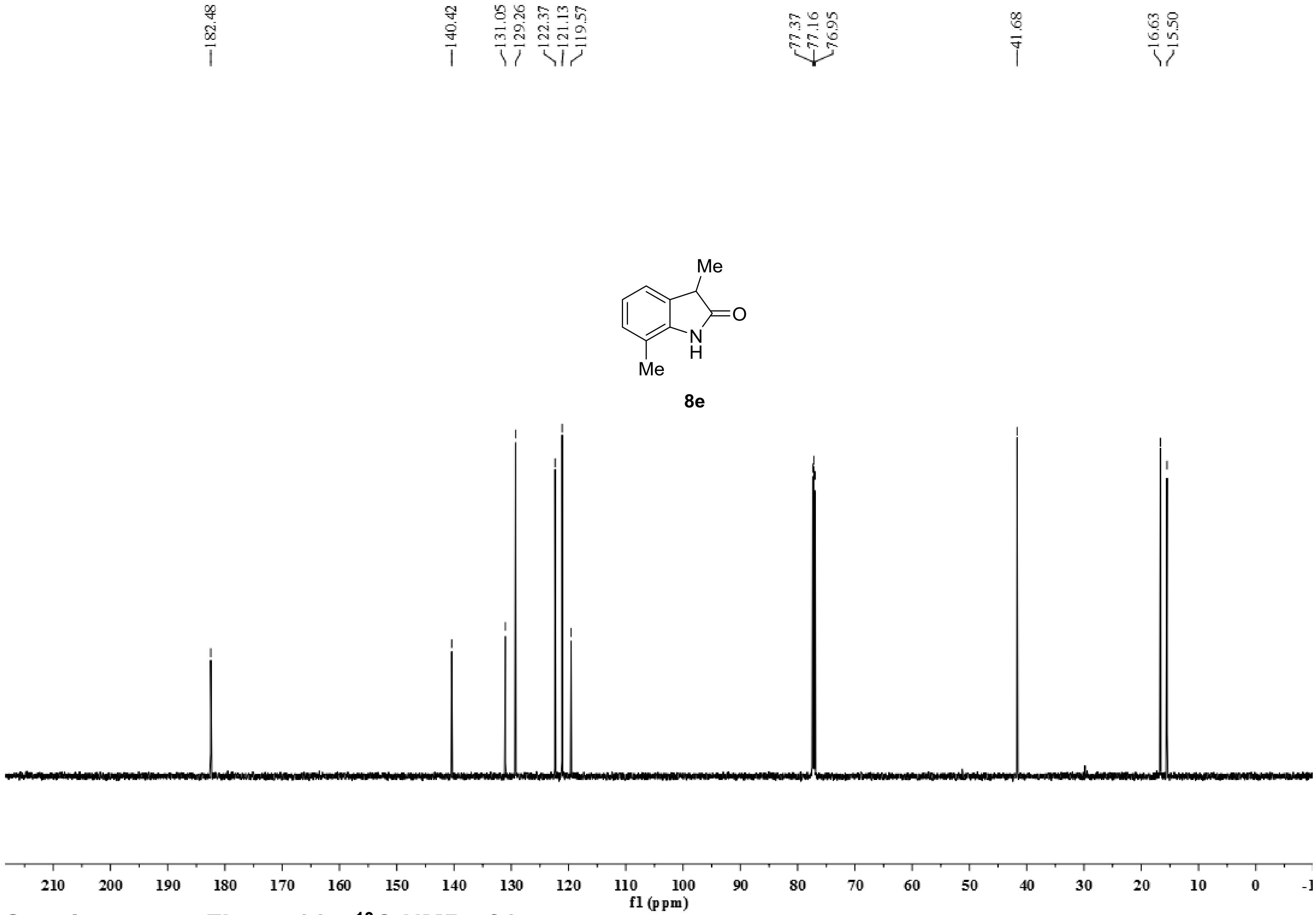

Supplementary Figure 147. ^{13}C-NMR of 8e

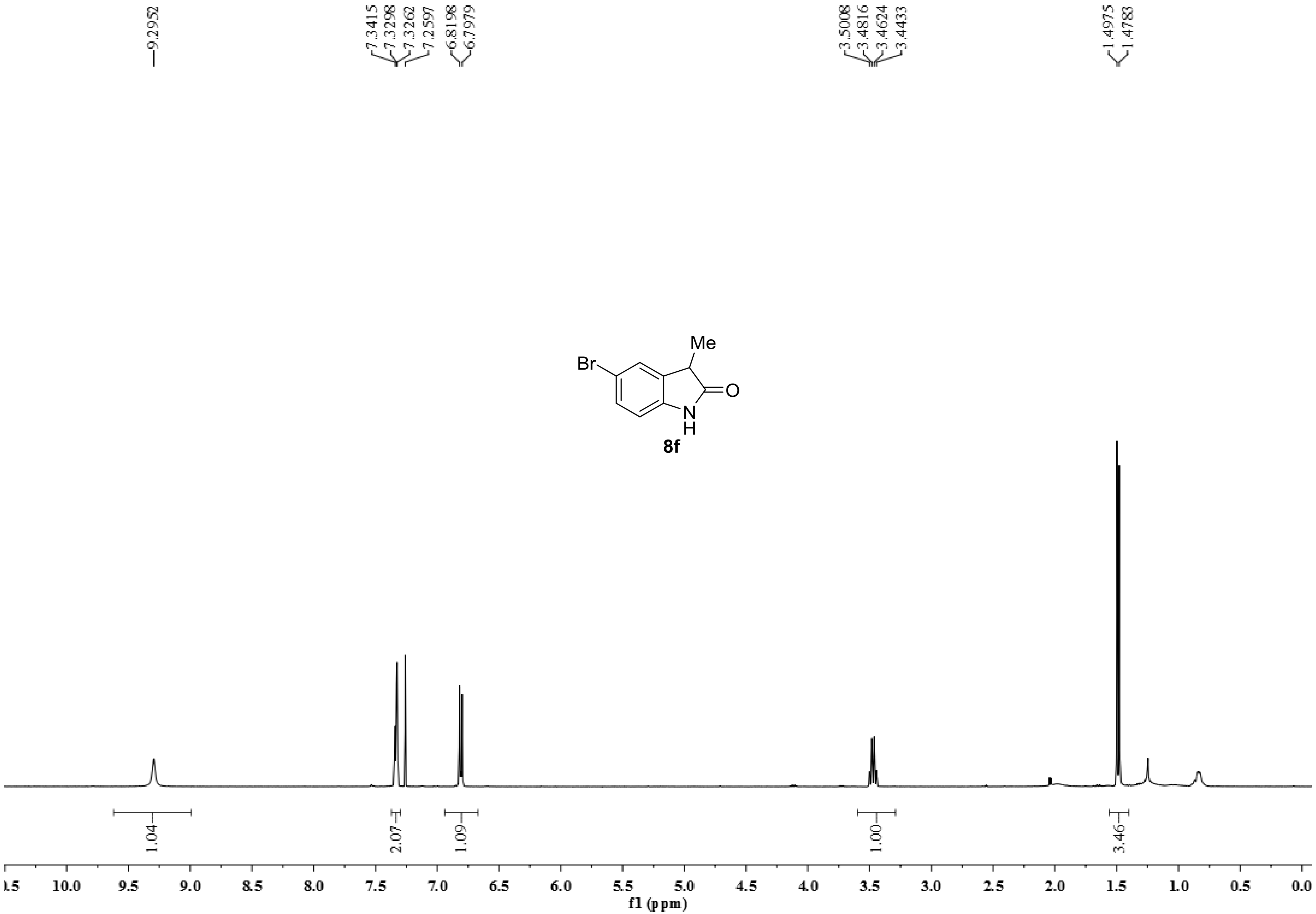

Supplementary Figure 148. ^{1}H-NMR of 8f

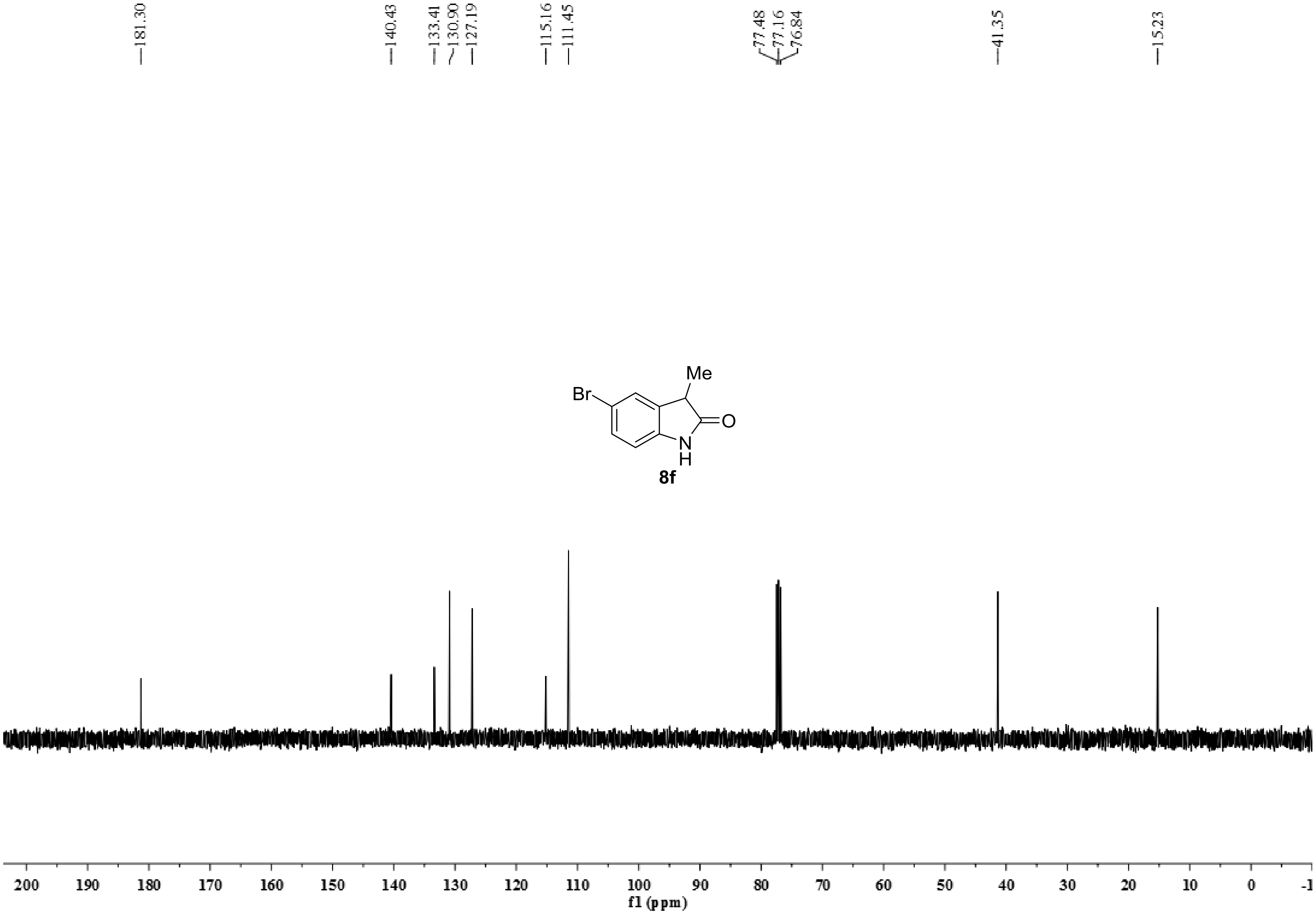

Supplementary Figure 149. ^{13}C-NMR of 8f

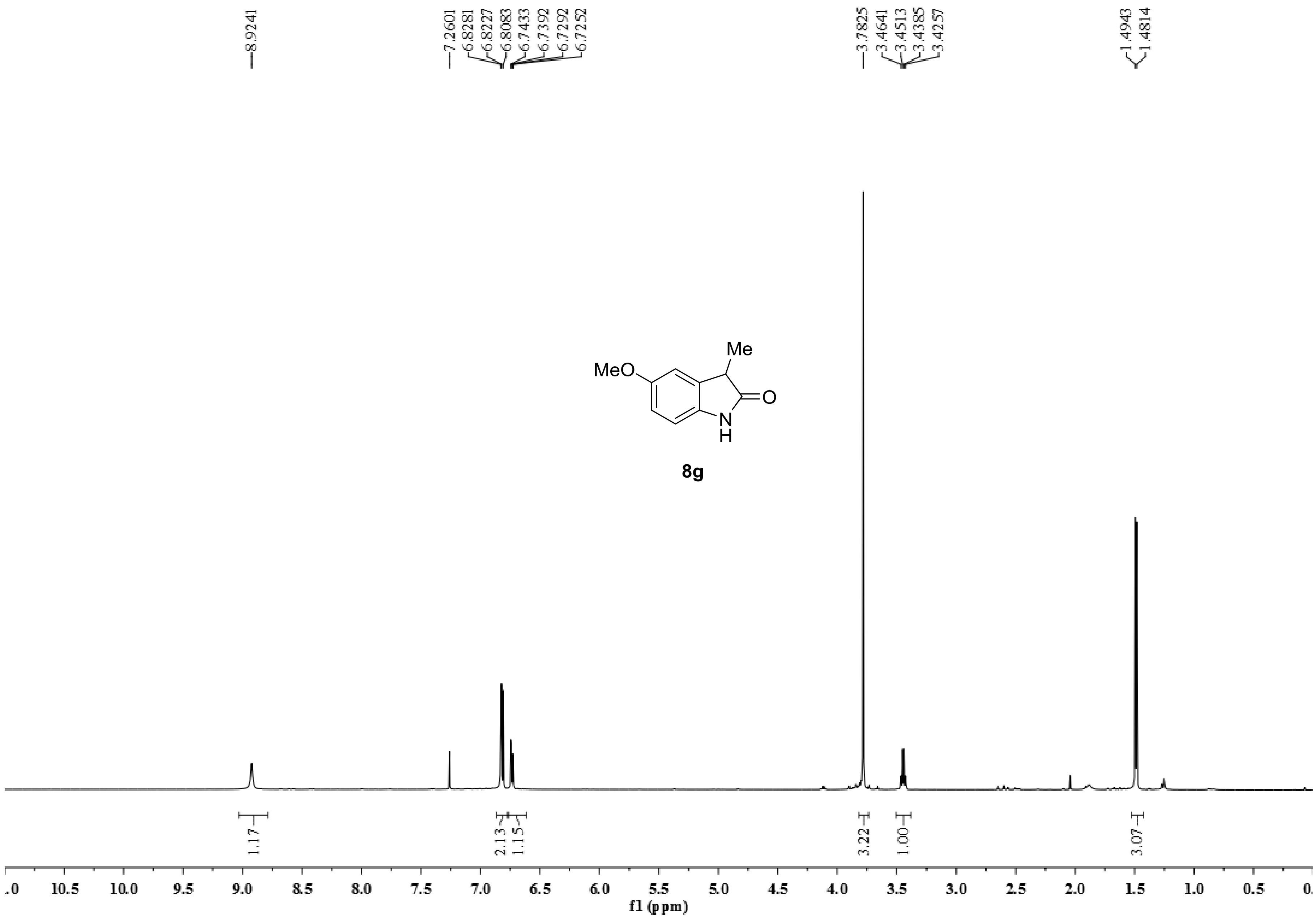

Supplementary Figure 150. ^{1}H-NMR of 8g

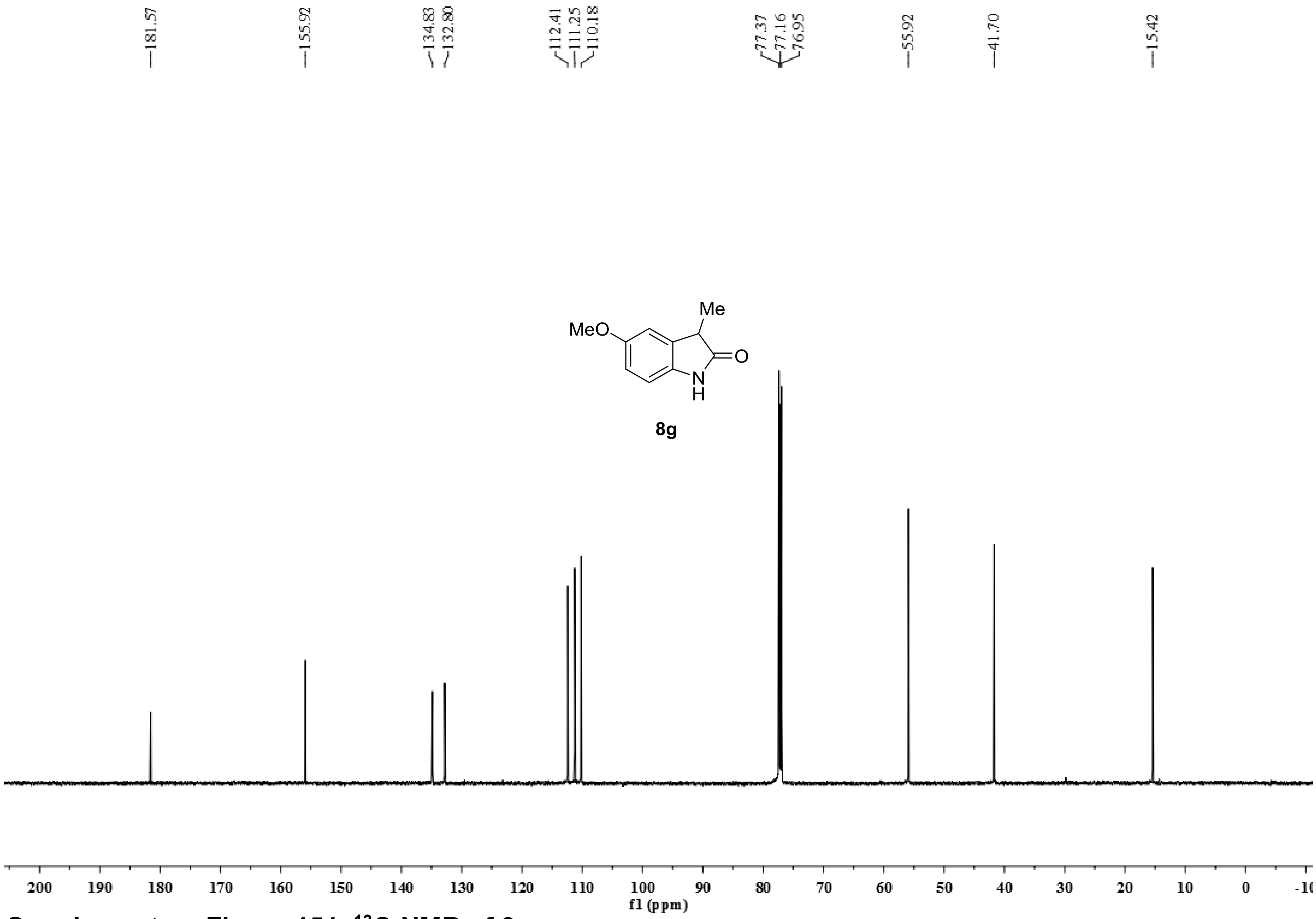

Supplementary Figure 151. ^{13}C-NMR of 8g

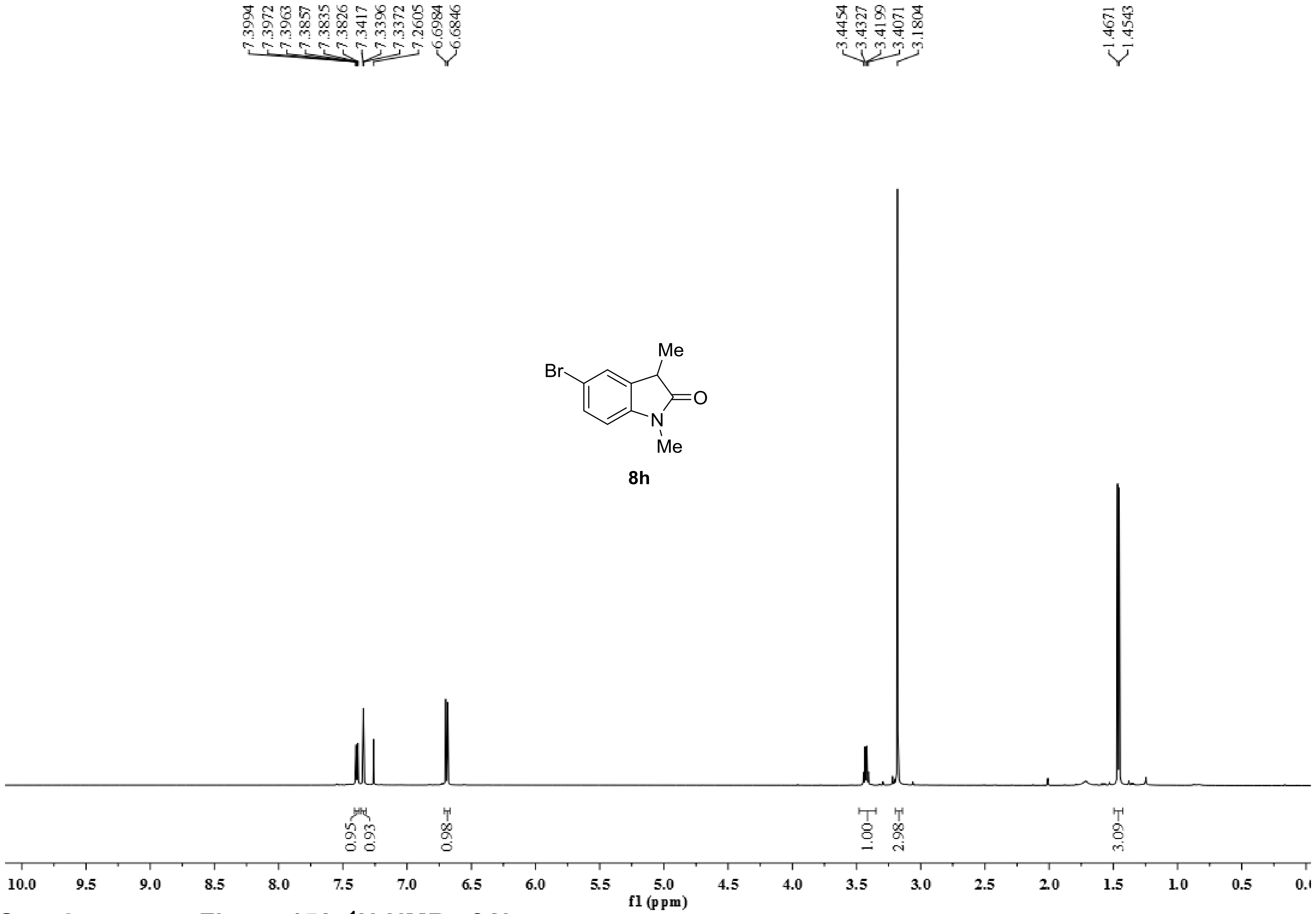

Supplementary Figure 152. ^{1}H-NMR of 8h

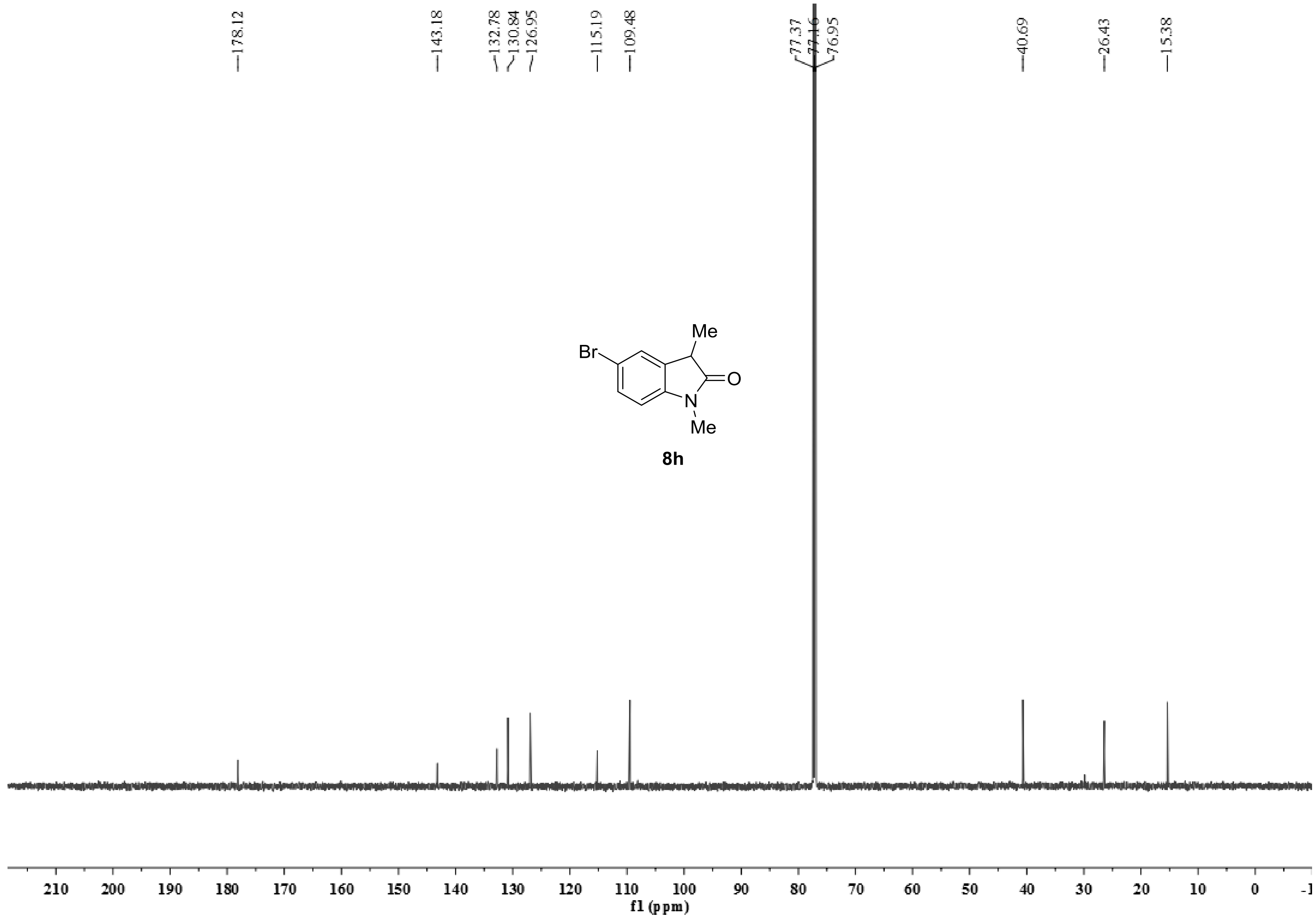

Supplementary Figure 153. ^{13}C-NMR of 8h

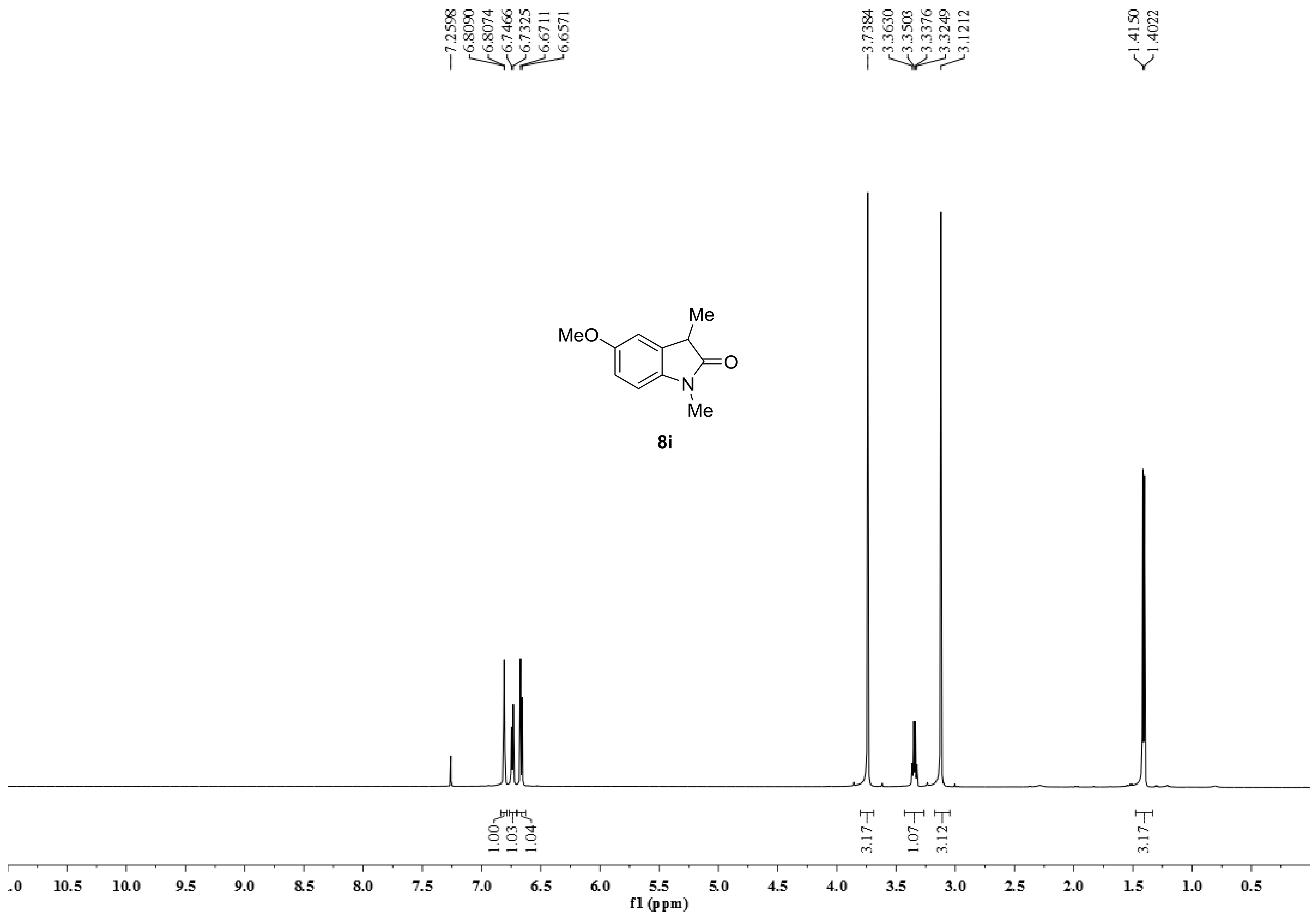

Supplementary Figure 154. ¹H-NMR of 8i

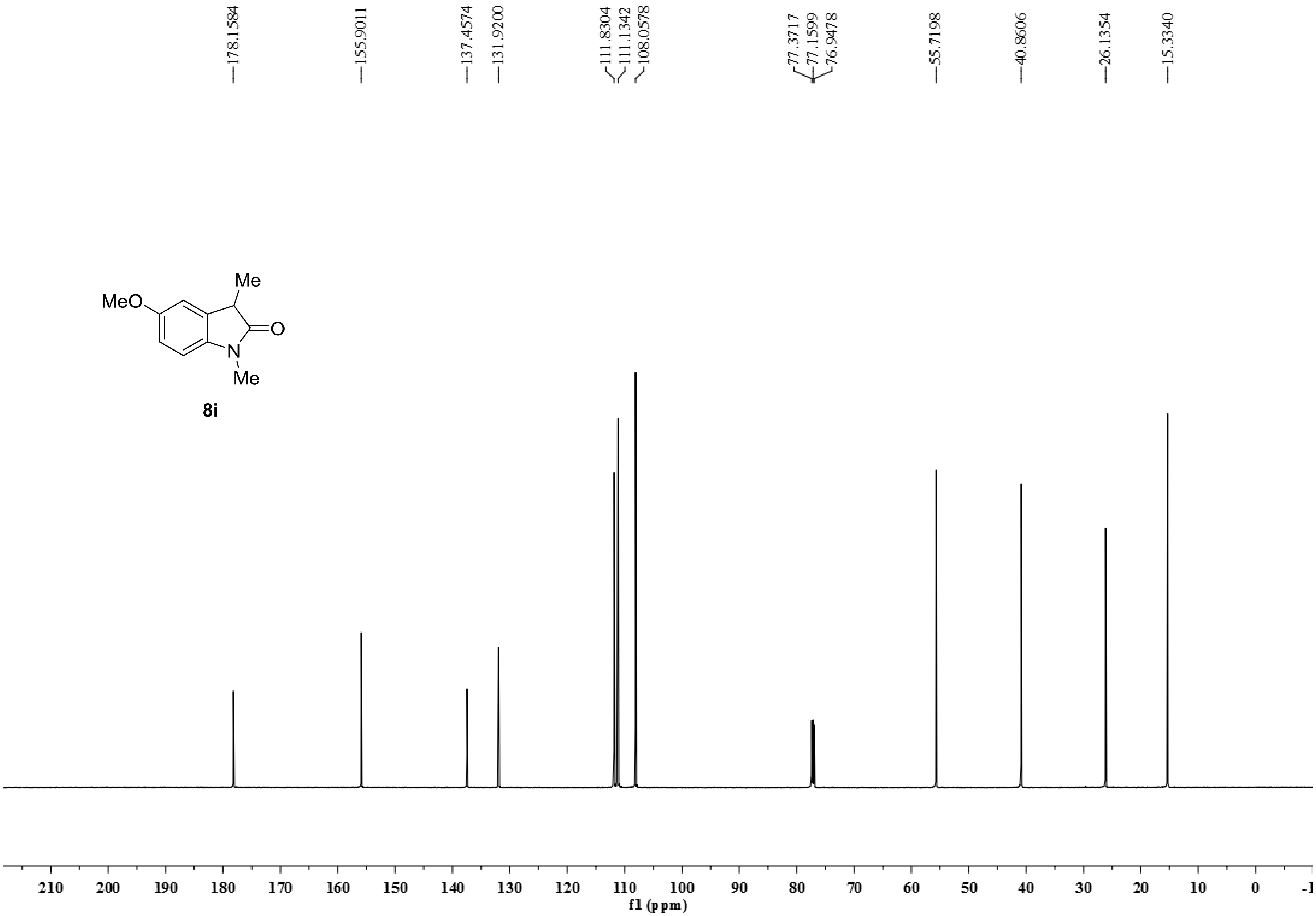

Supplementary Figure 155. ^{13}C-NMR of 8i

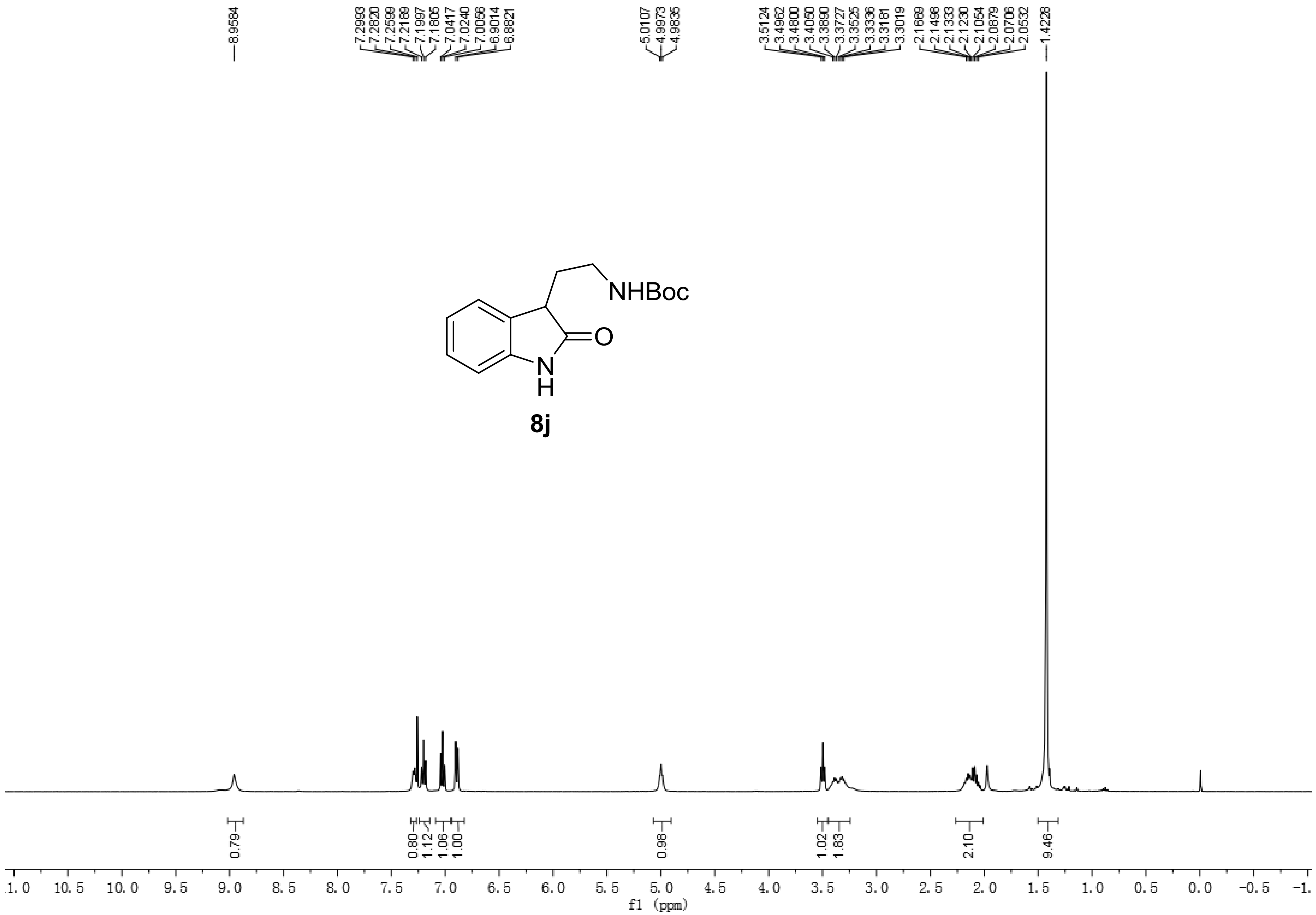

Supplementary Figure 156. ^{1}H-NMR of 8j

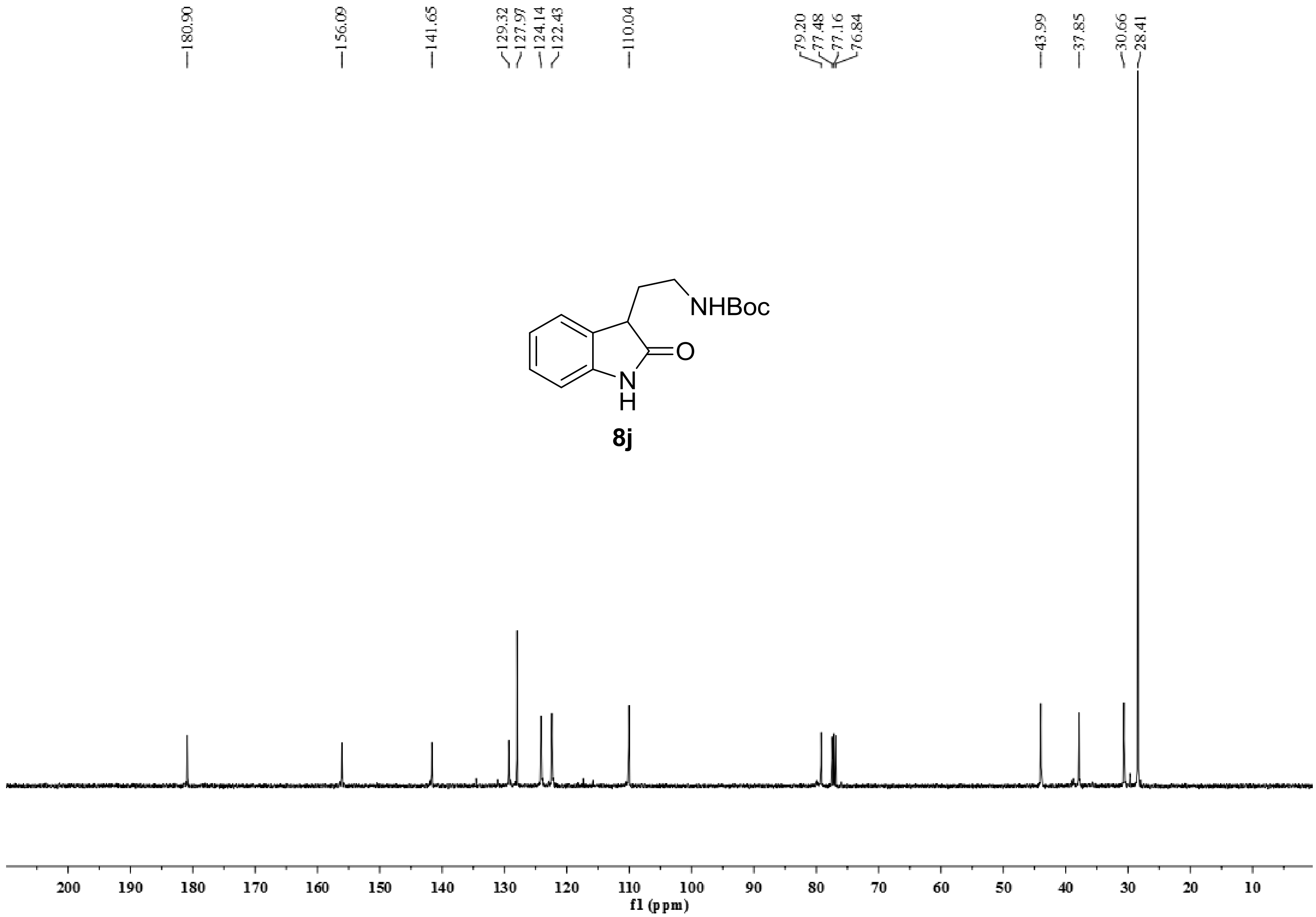

Supplementary Figure 157. ^{13}C-NMR of 8j

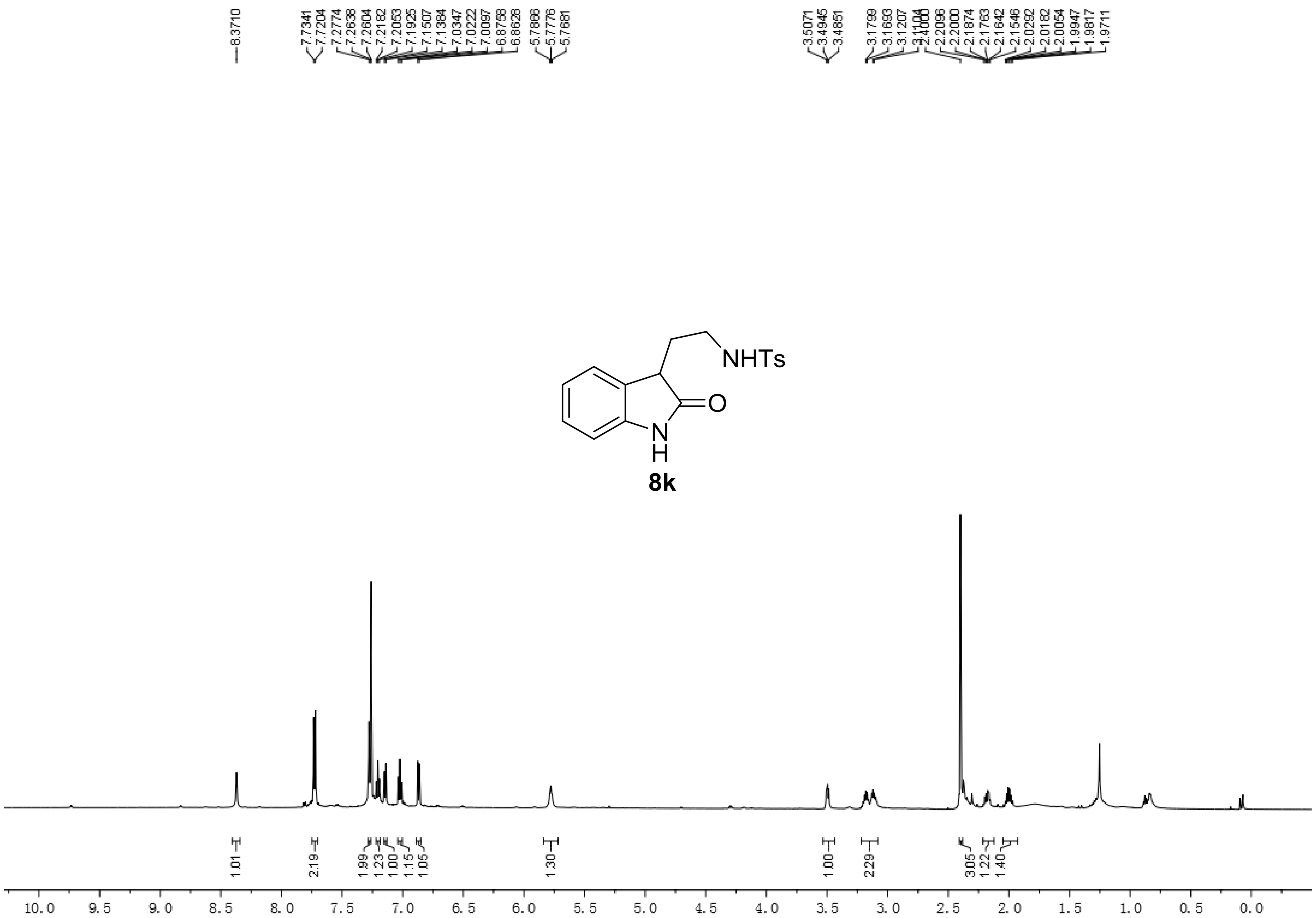

Supplementary Figure 158. ^{1}H-NMR of 8k

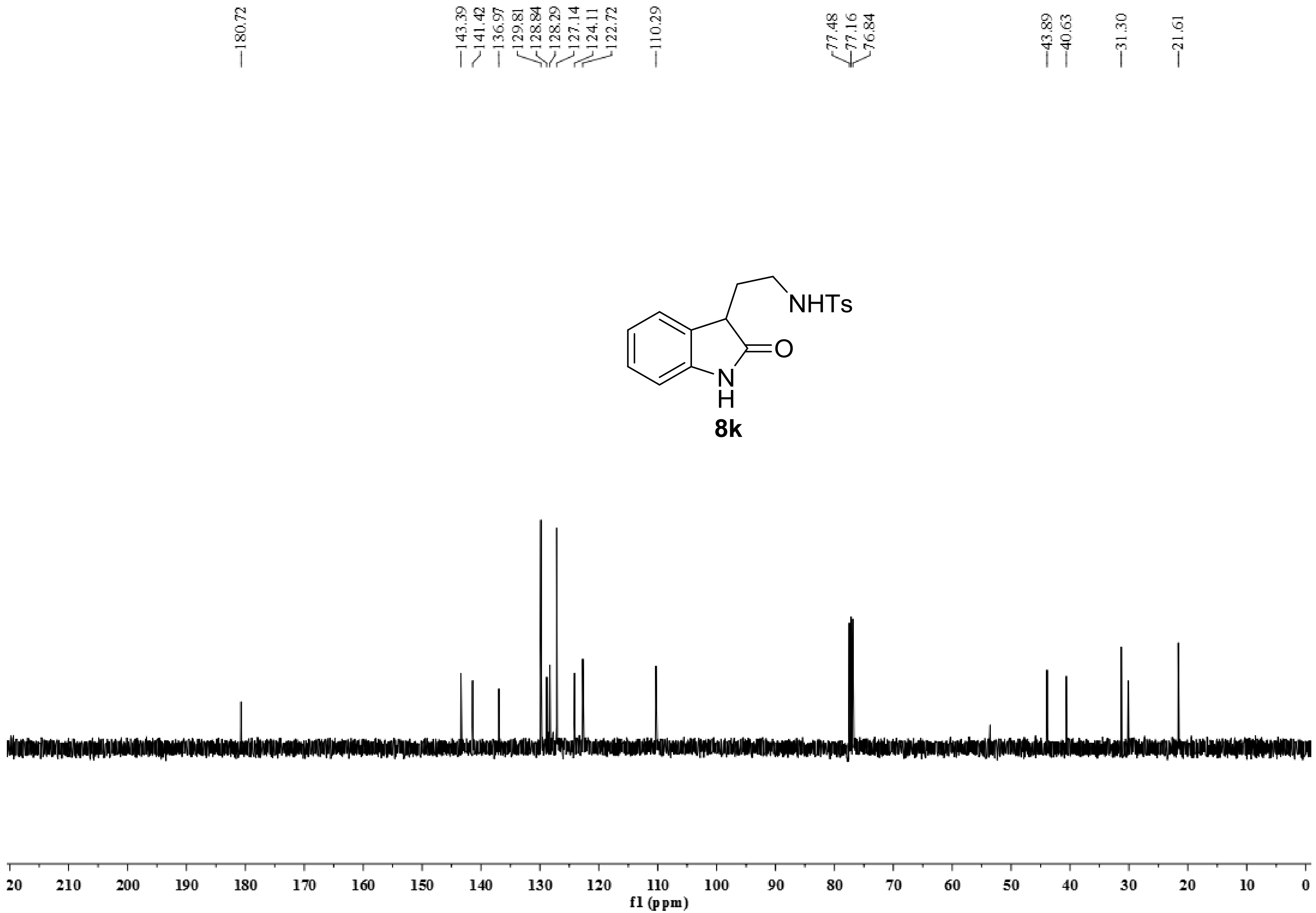

Supplementary Figure 159. ^{13}C-NMR of 8k

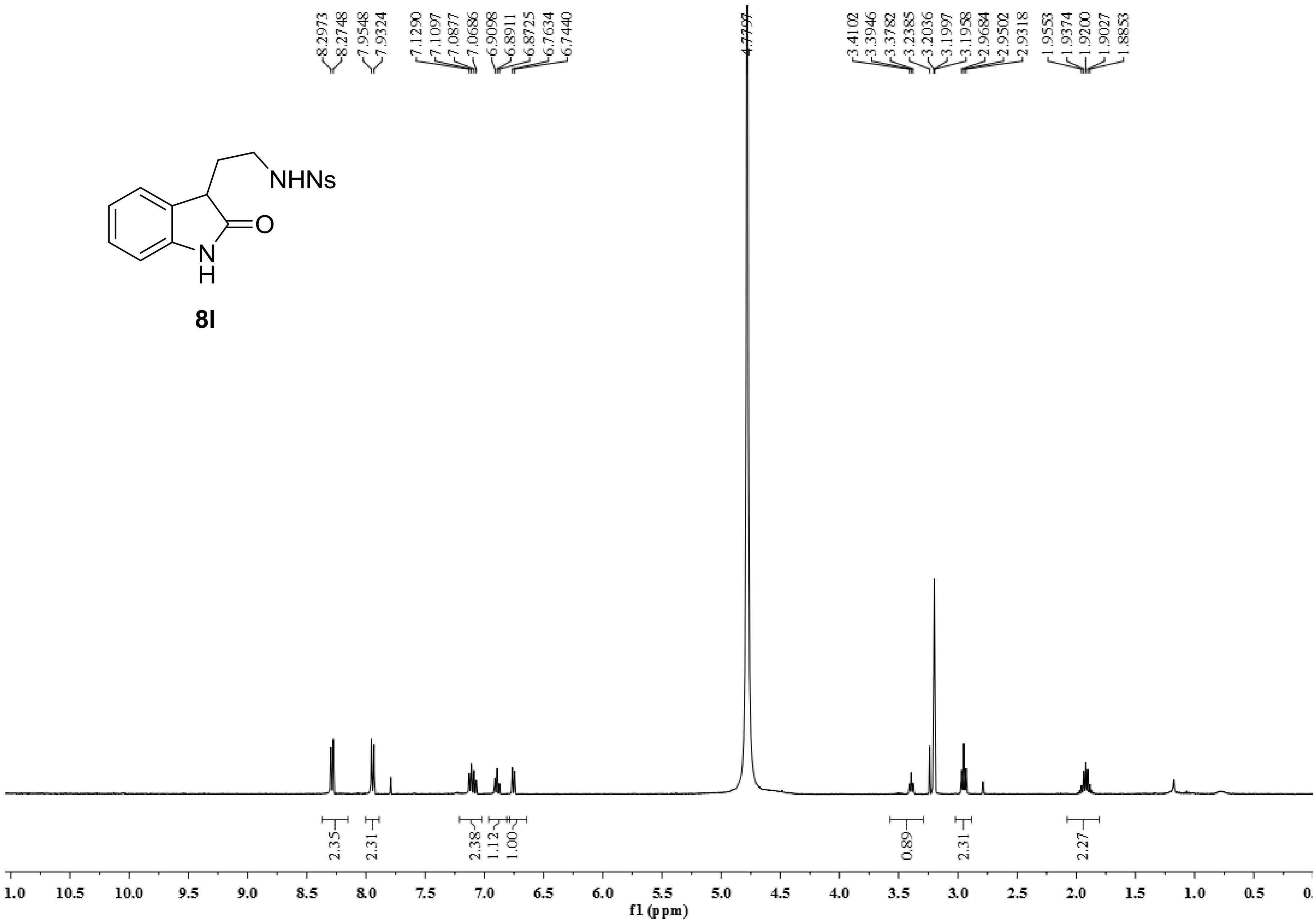

Supplementary Figure 160. ^{1}H-NMR of 8l

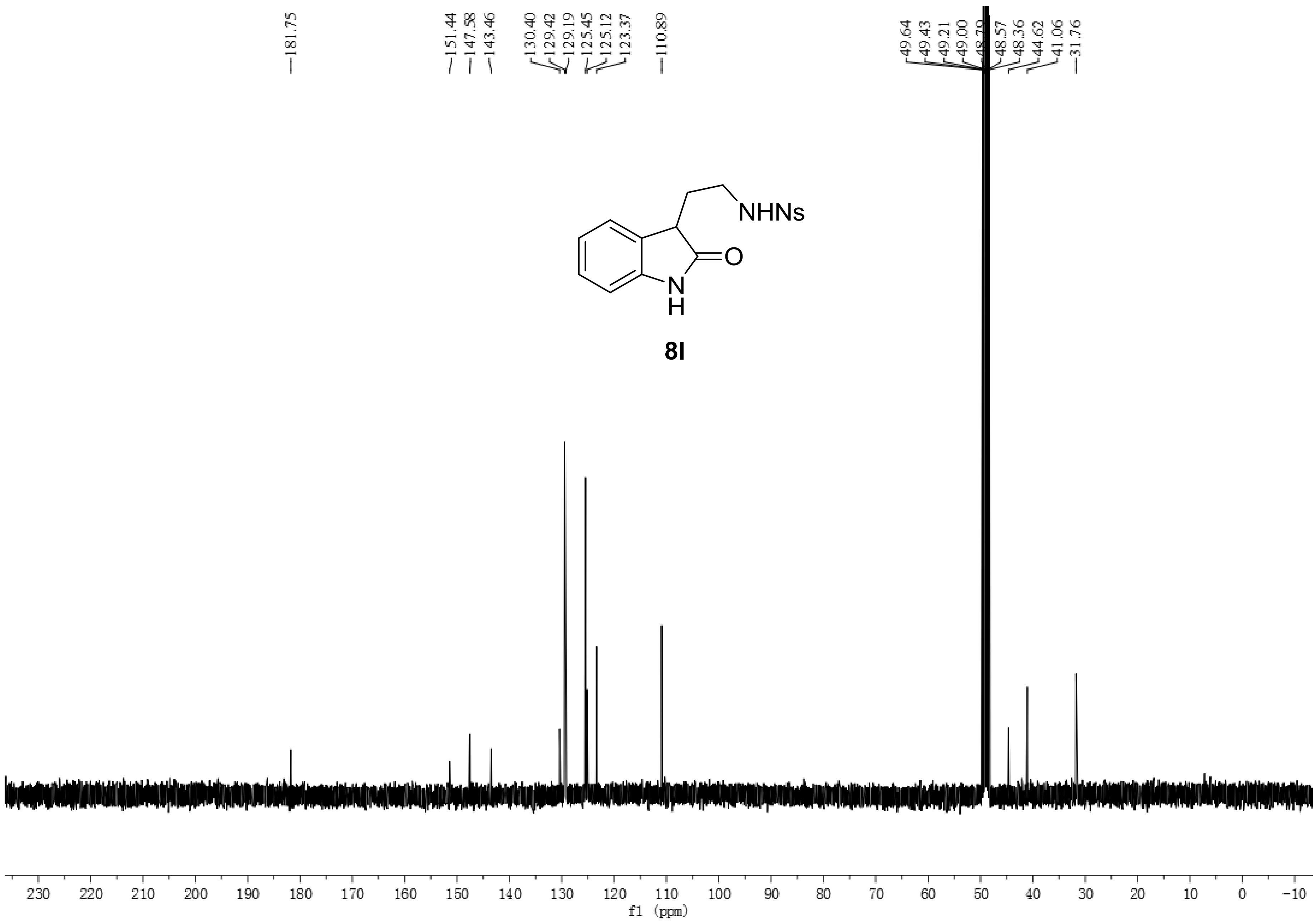

Supplementary Figure 161. ^{13}C-NMR of 8l

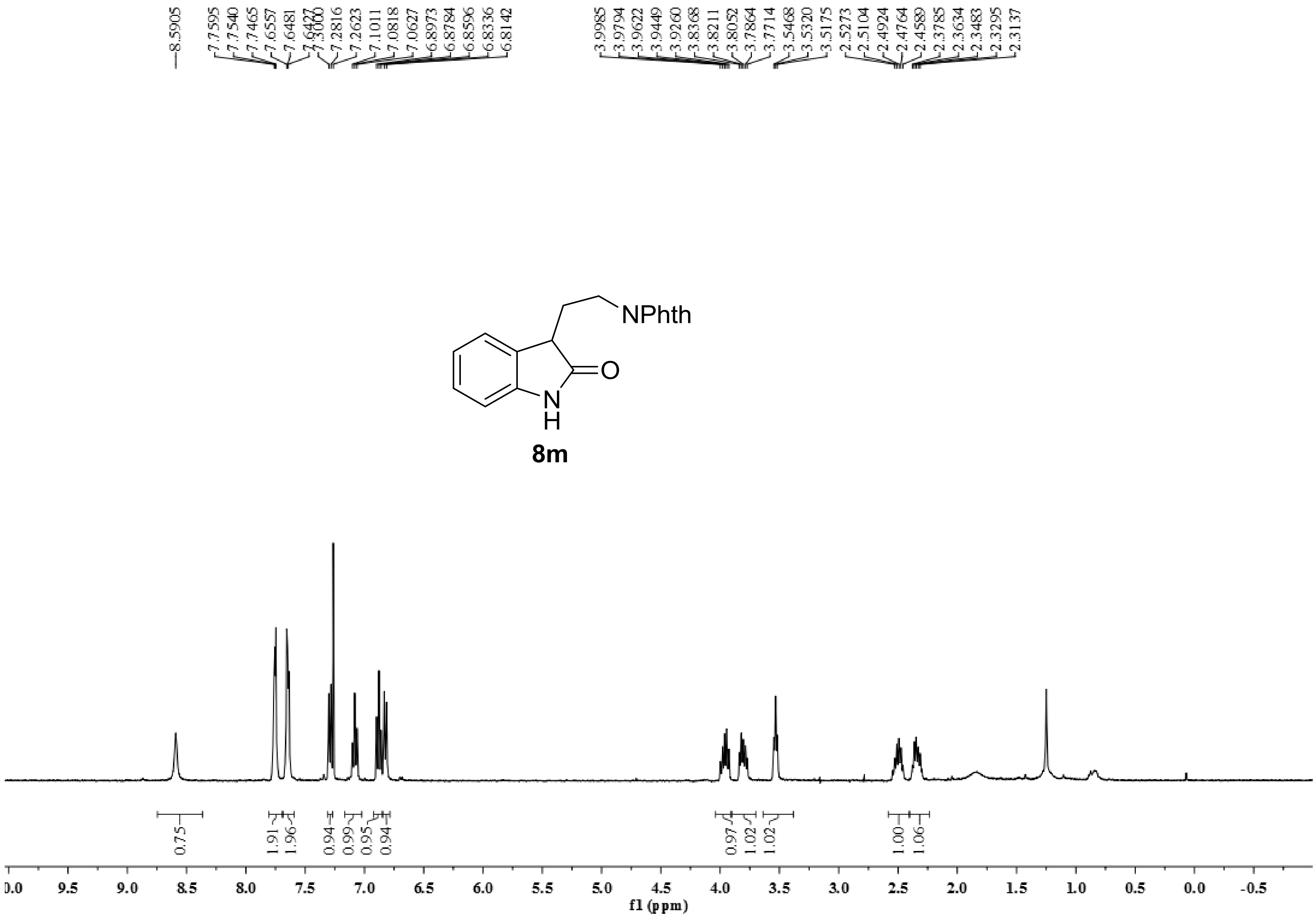

Supplementary Figure 162. ^{1}H-NMR of 8m

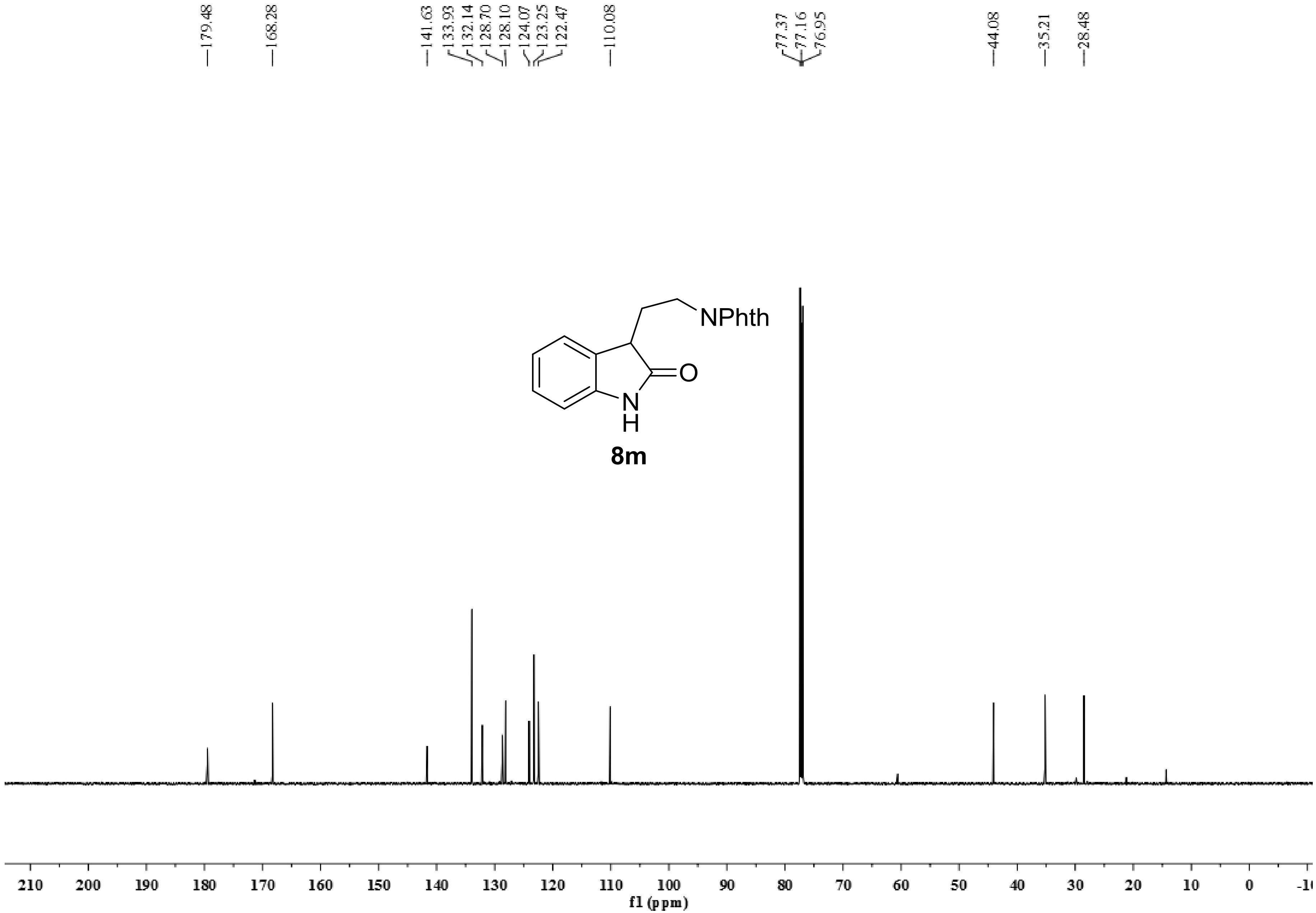

Supplementary Figure 161. ^{13}C-NMR of 8m

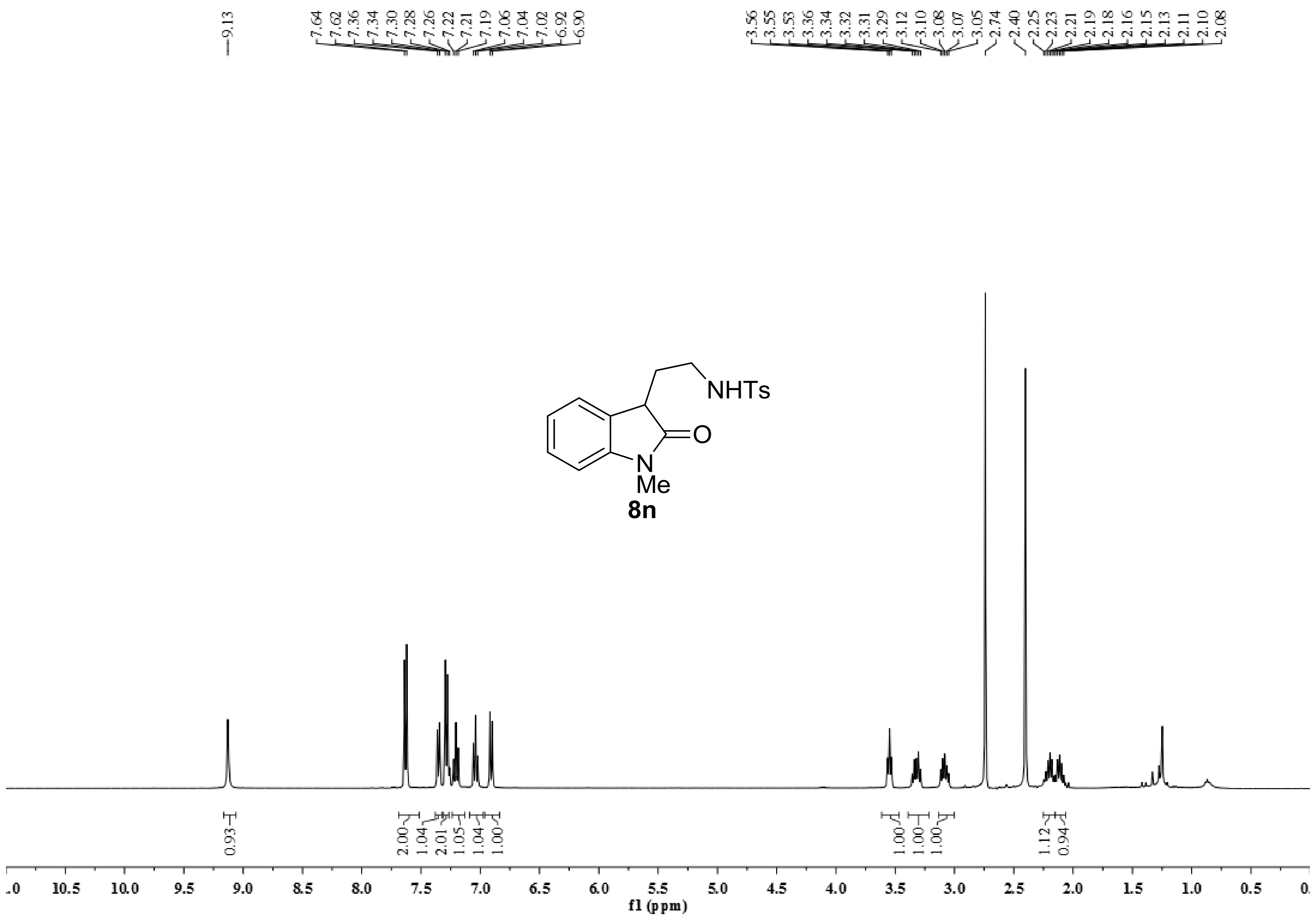

Supplementary Figure 164. ^{1}H-NMR of 8n

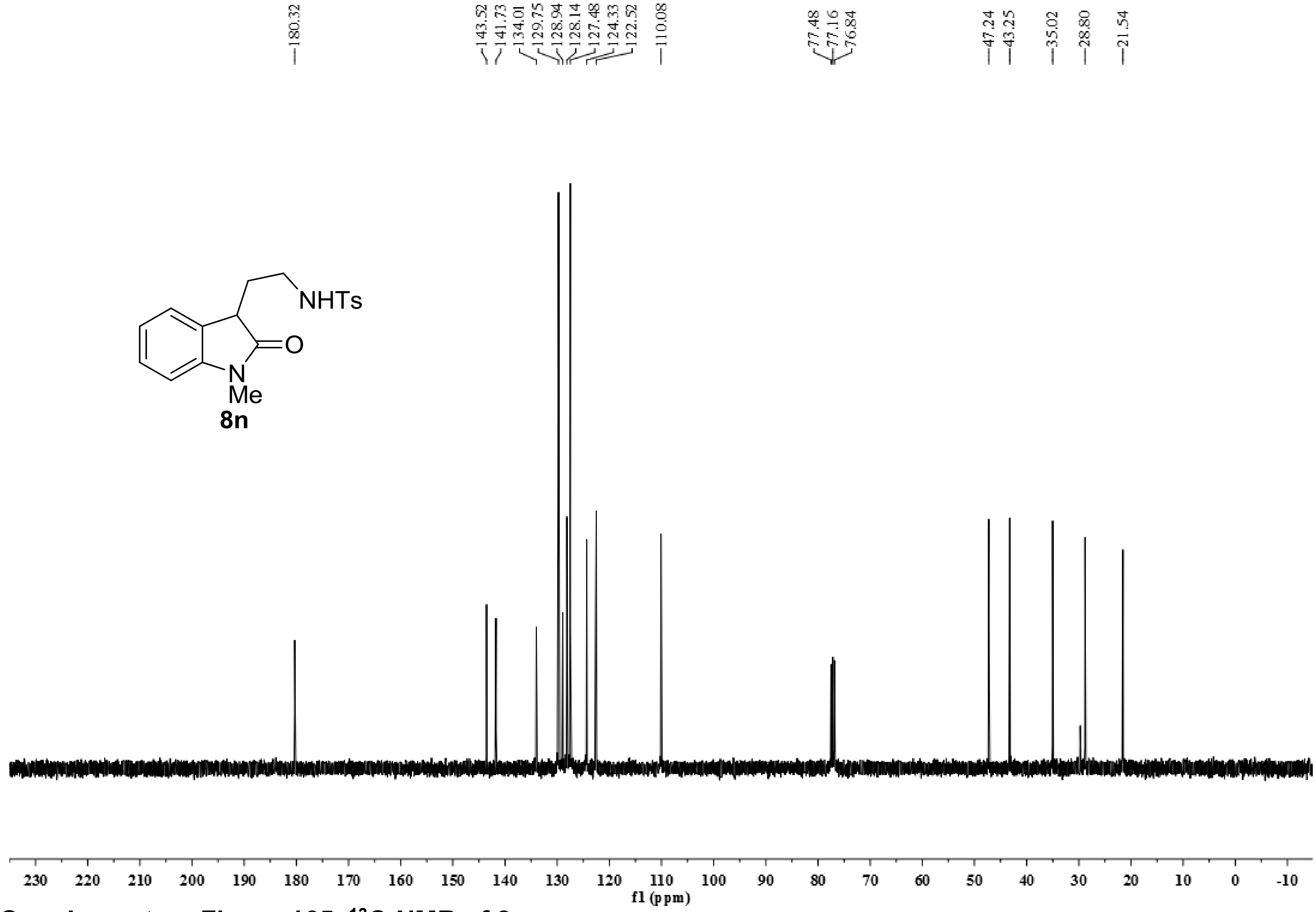

Supplementary Figure 165. ^{13}C-NMR of 8n

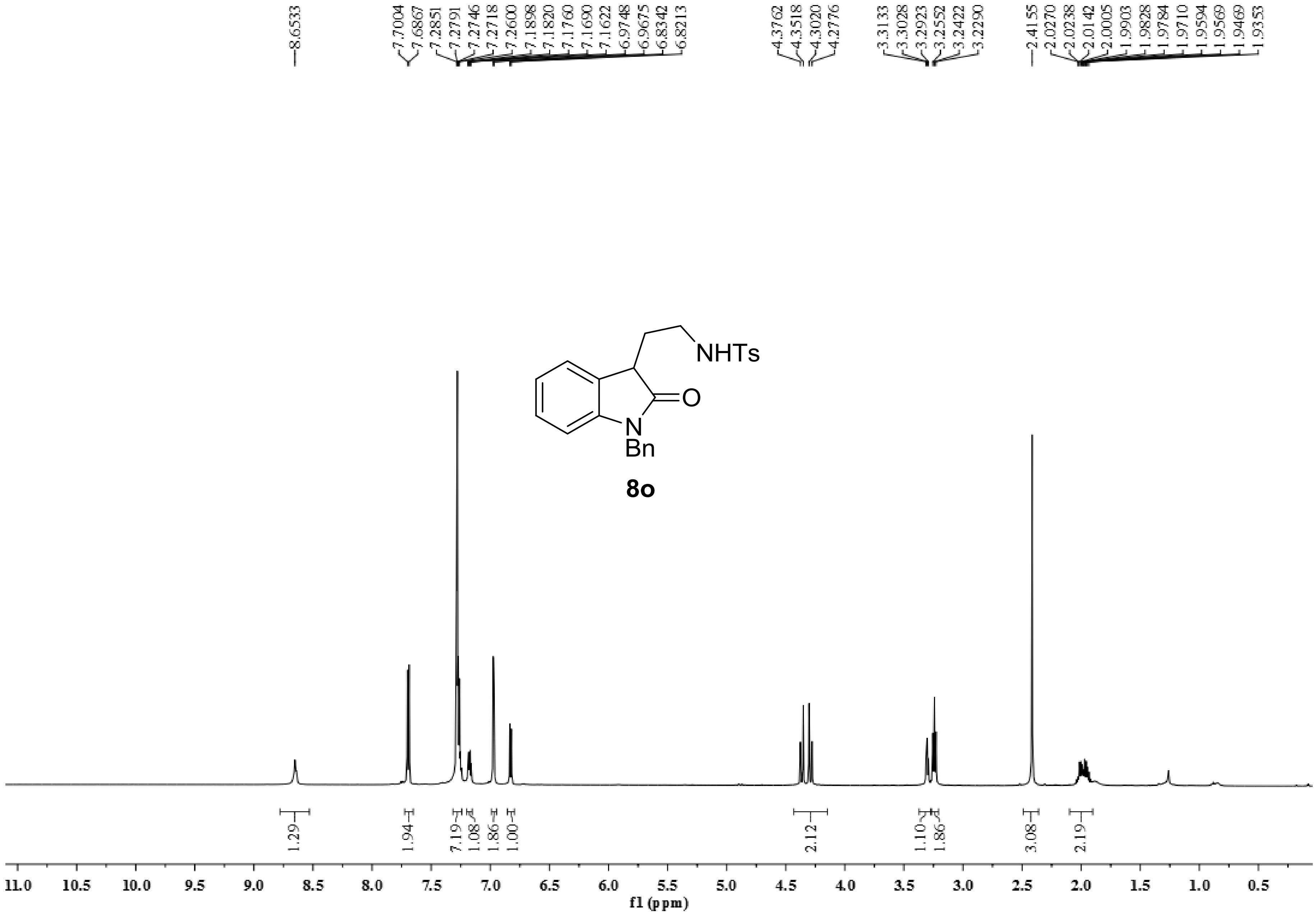

Supplementary Figure 166. ¹H-NMR of 8o

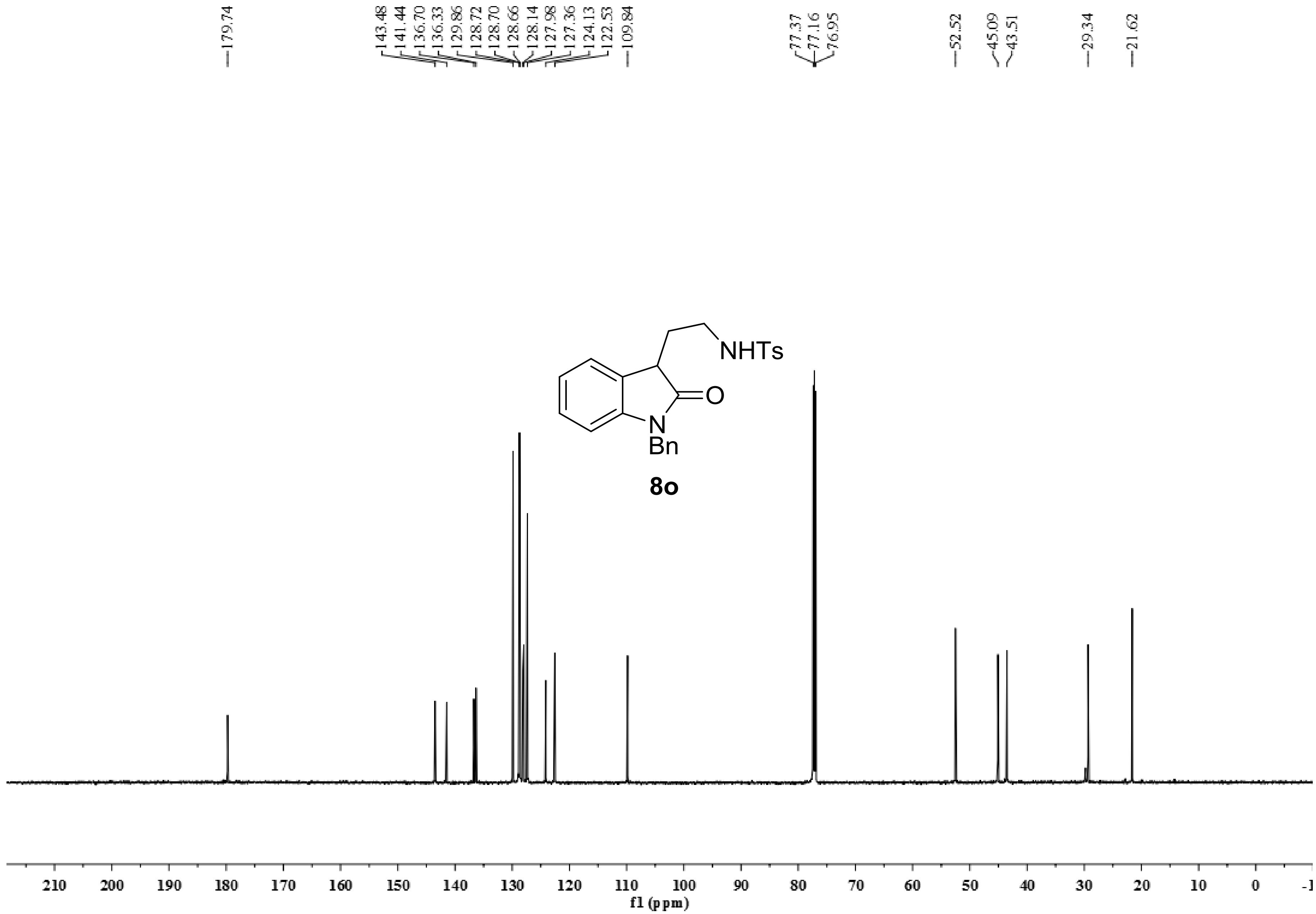

Supplementary Figure 167. ^{13}C-NMR of 8o

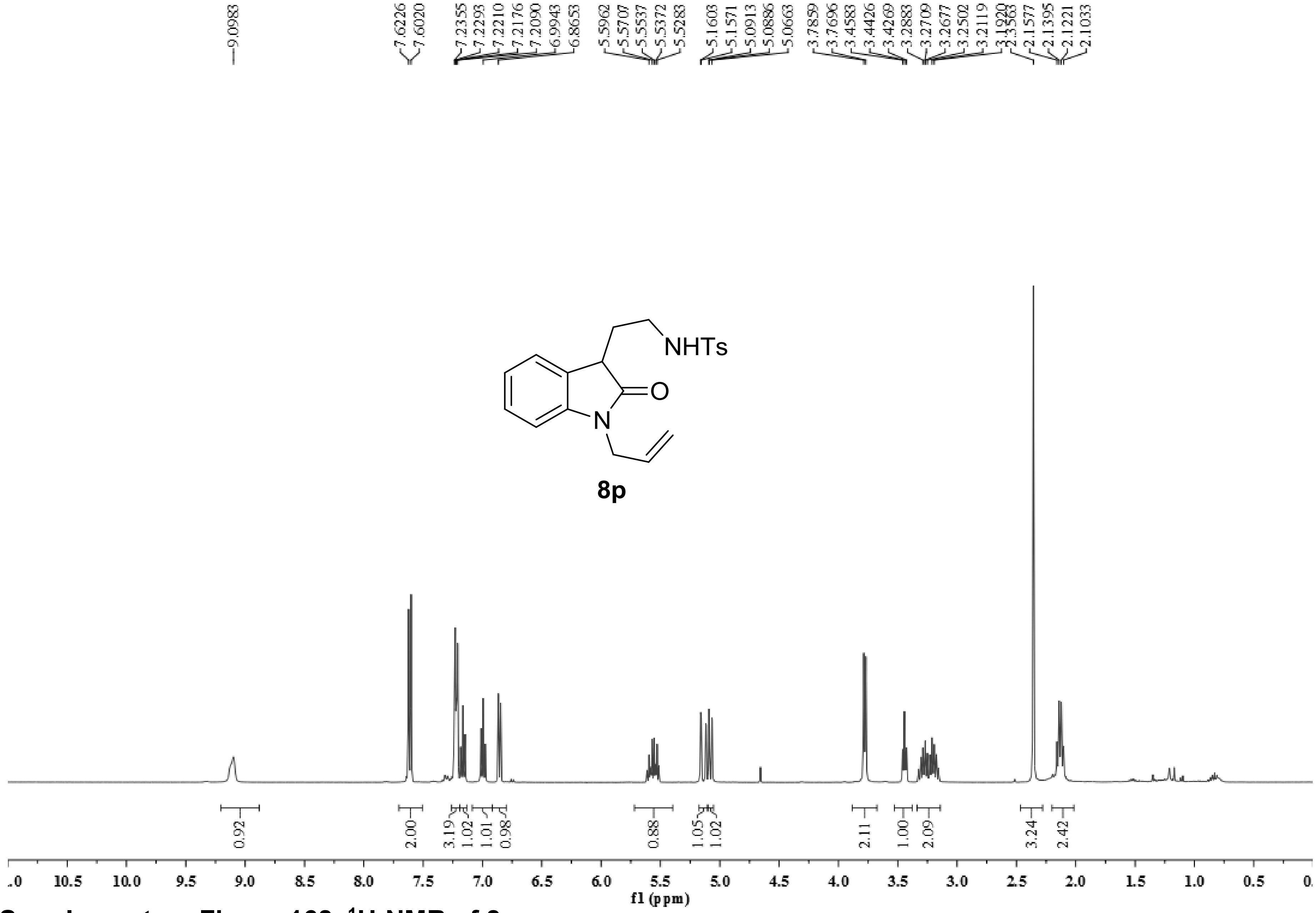

Supplementary Figure 168. ¹H-NMR of 8p

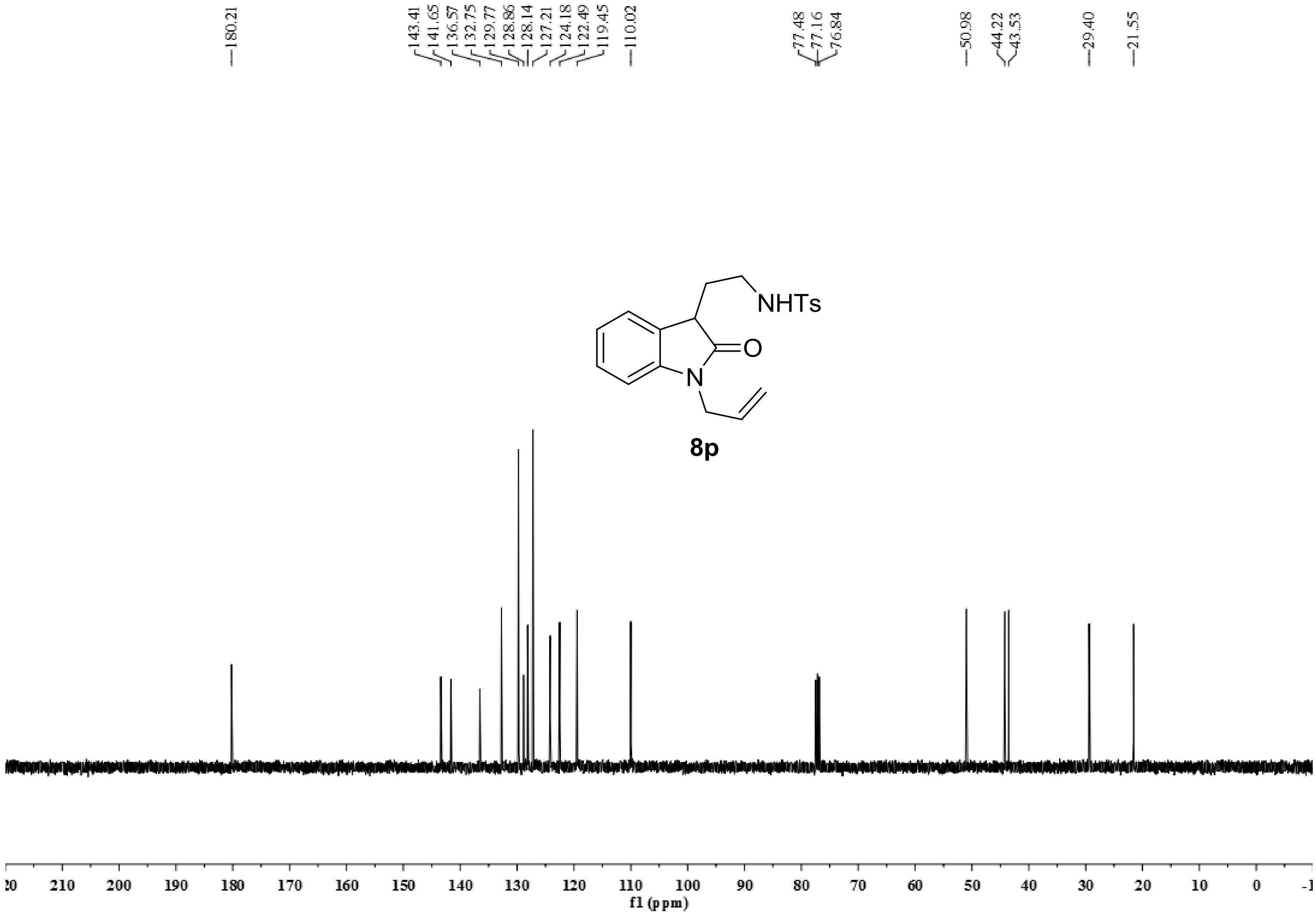

Supplementary Figure 169. ^{13}C-NMR of 8p

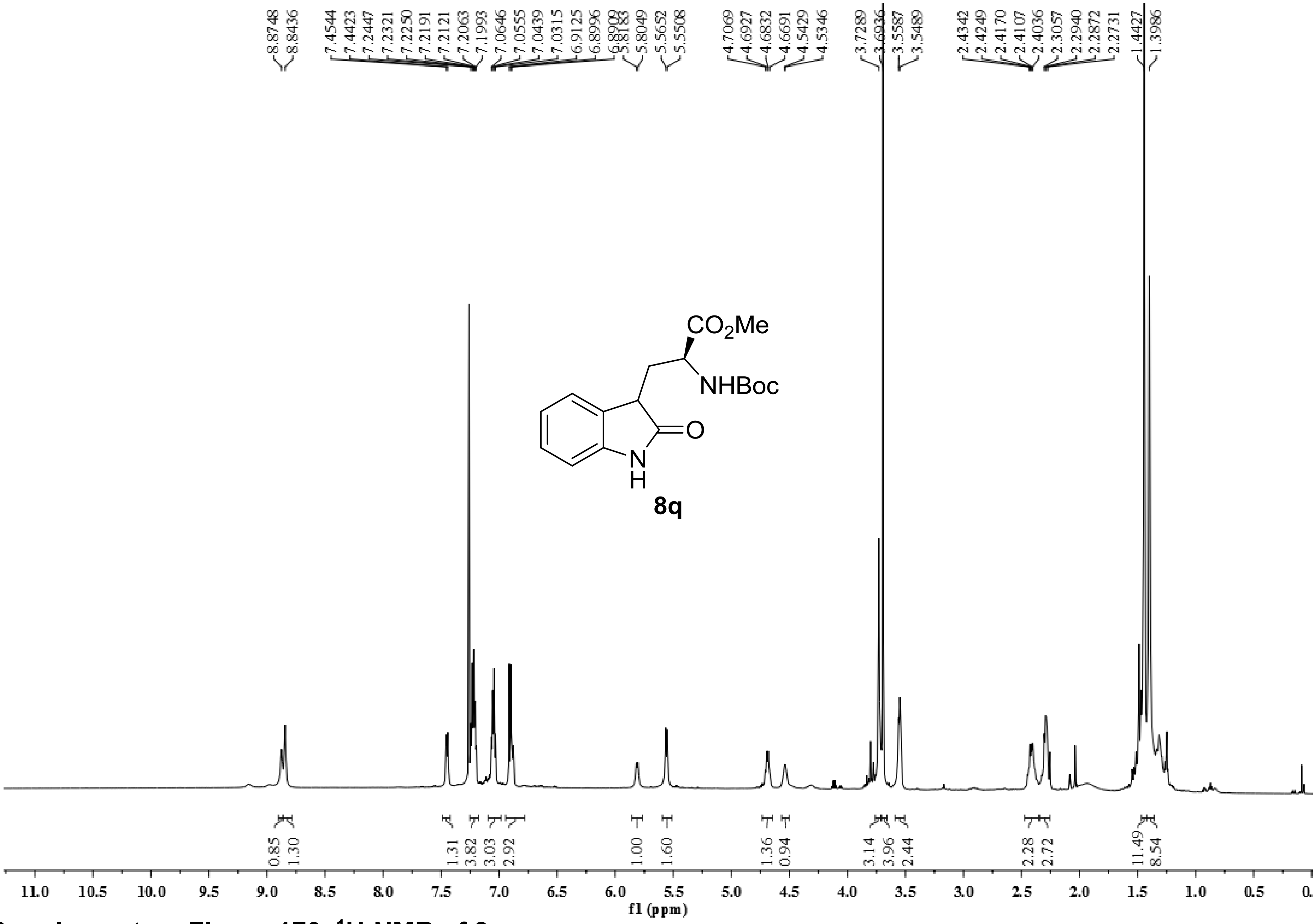

Supplementary Figure 170. ^{1}H-NMR of 8q

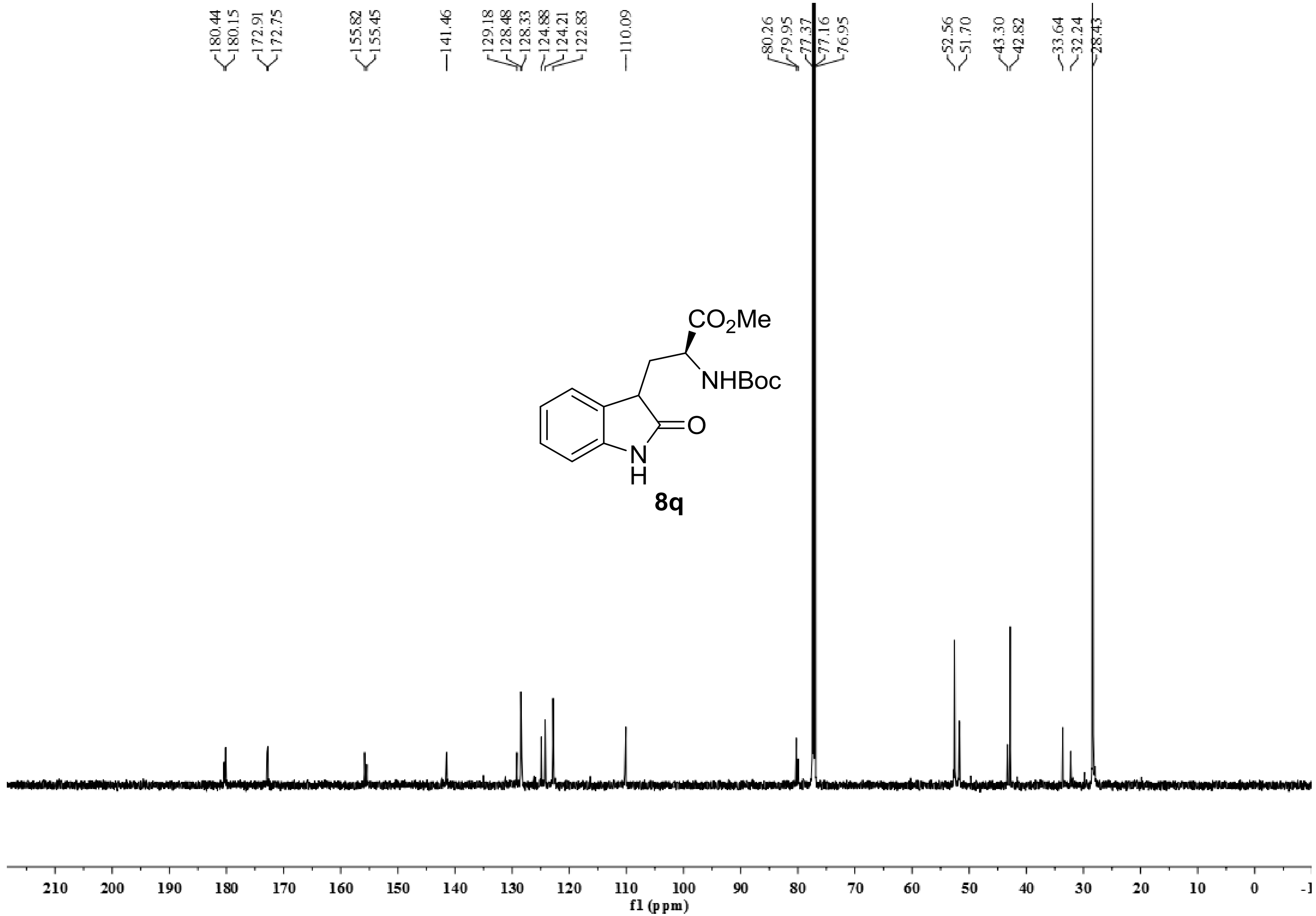

Supplementary Figure 171. ^{13}C-NMR of 8q

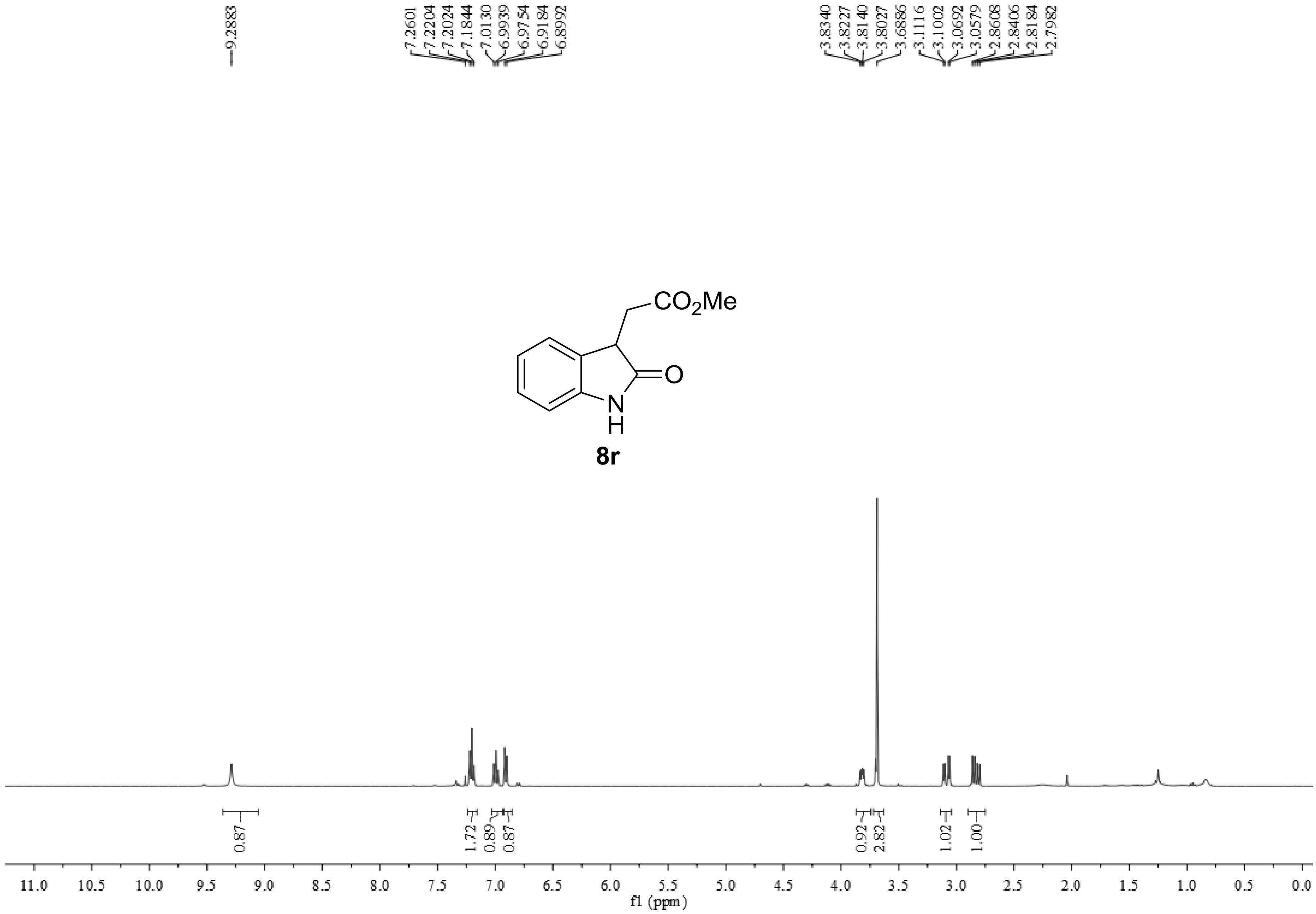

Supplementary Figure 172. ^{1}H-NMR of 8r

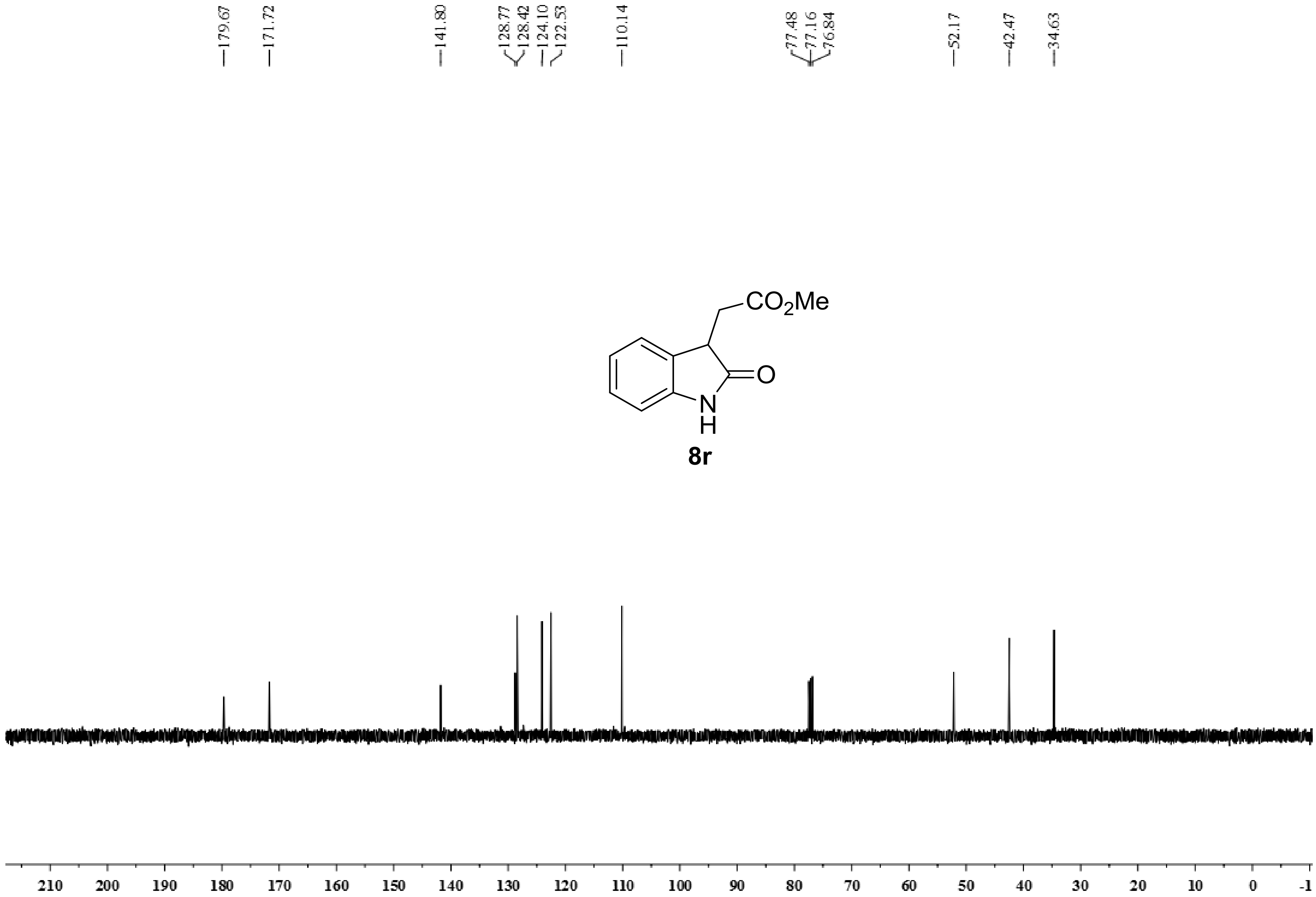

Supplementary Figure 173. ^{13}C-NMR of 8r

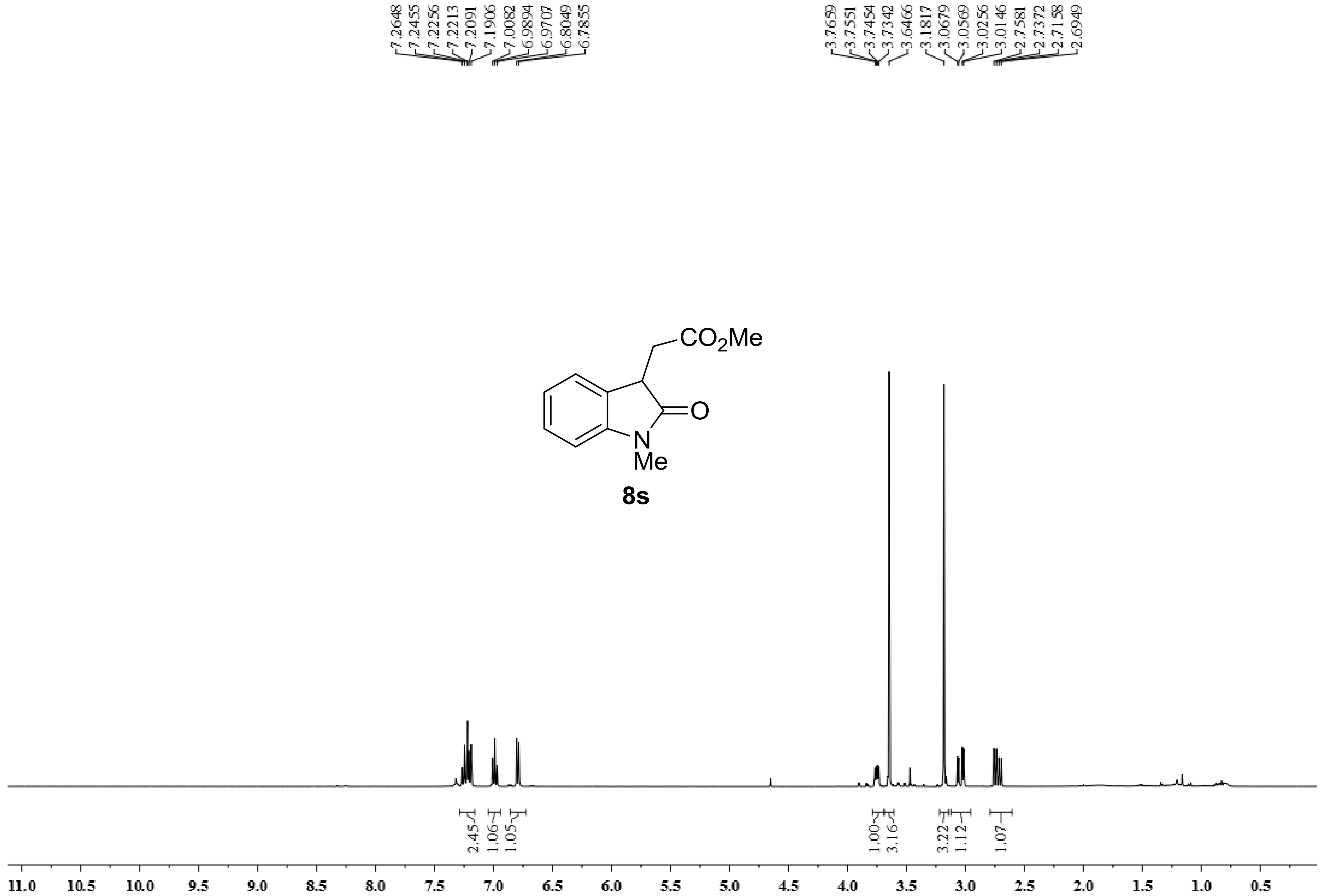

Supplementary Figure 174. ^{1}H-NMR of 8s

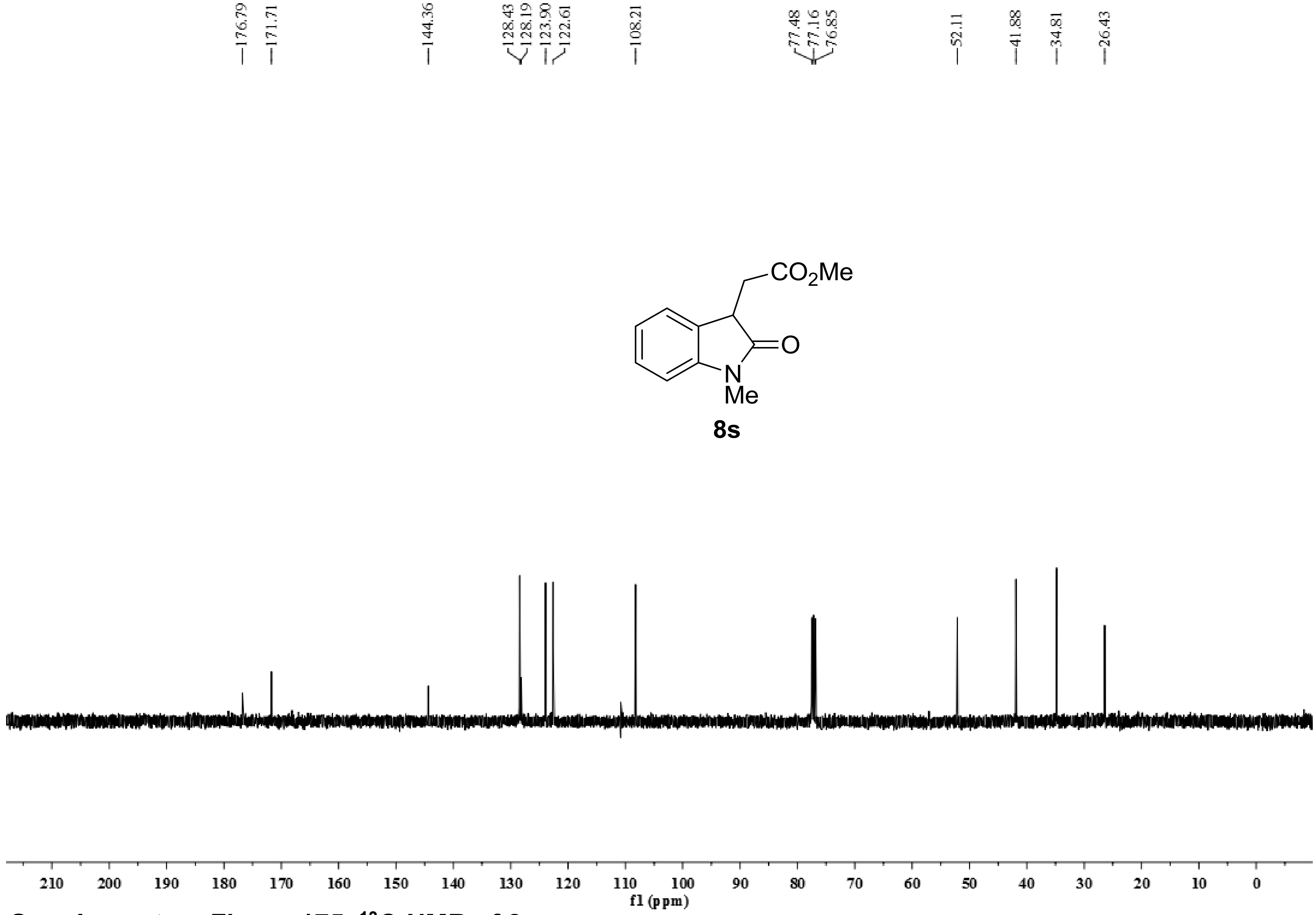

Supplementary Figure 175. ^{13}C-NMR of 8s

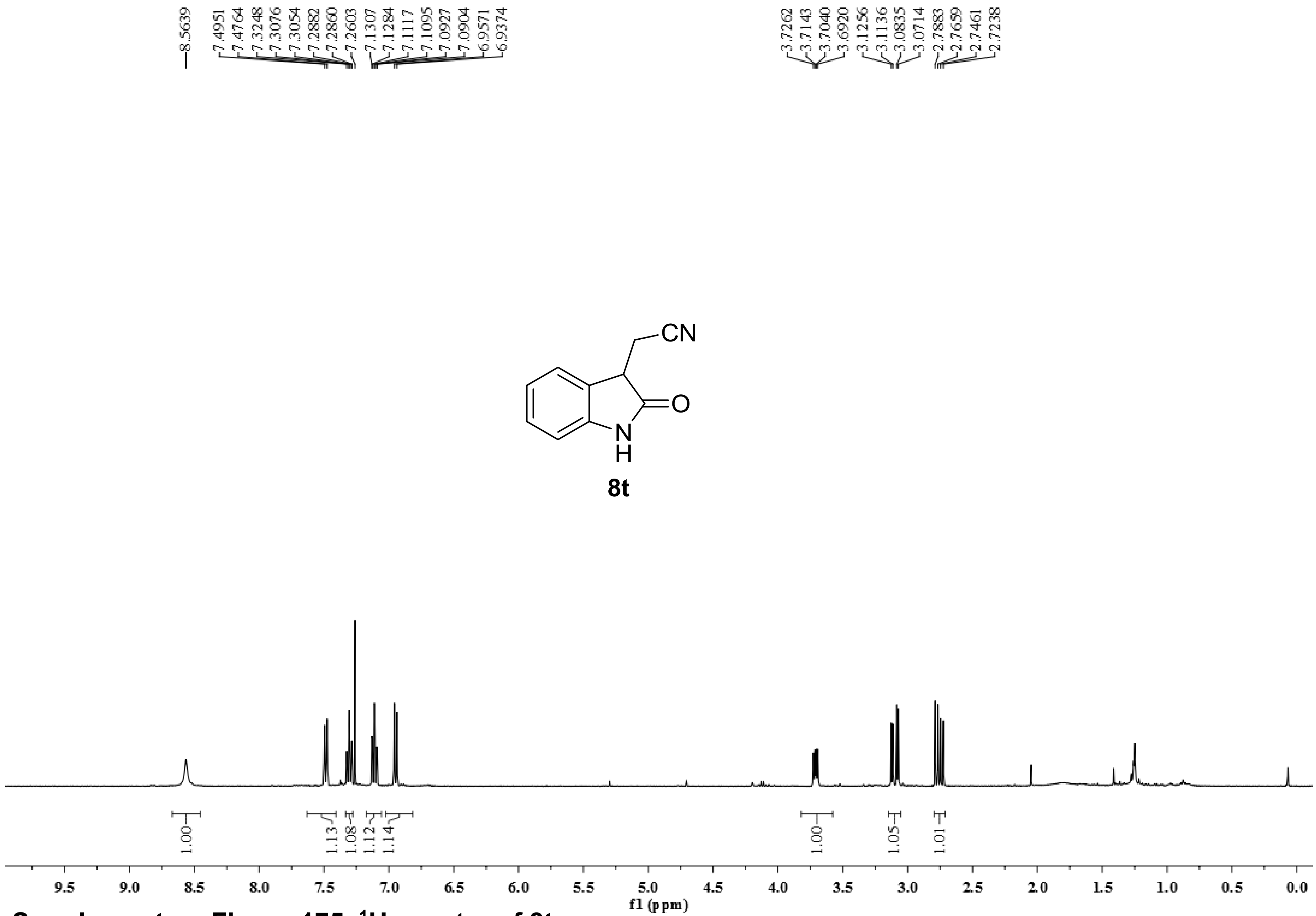

Supplementary Figure 175. ^{1}H spectra of 8t

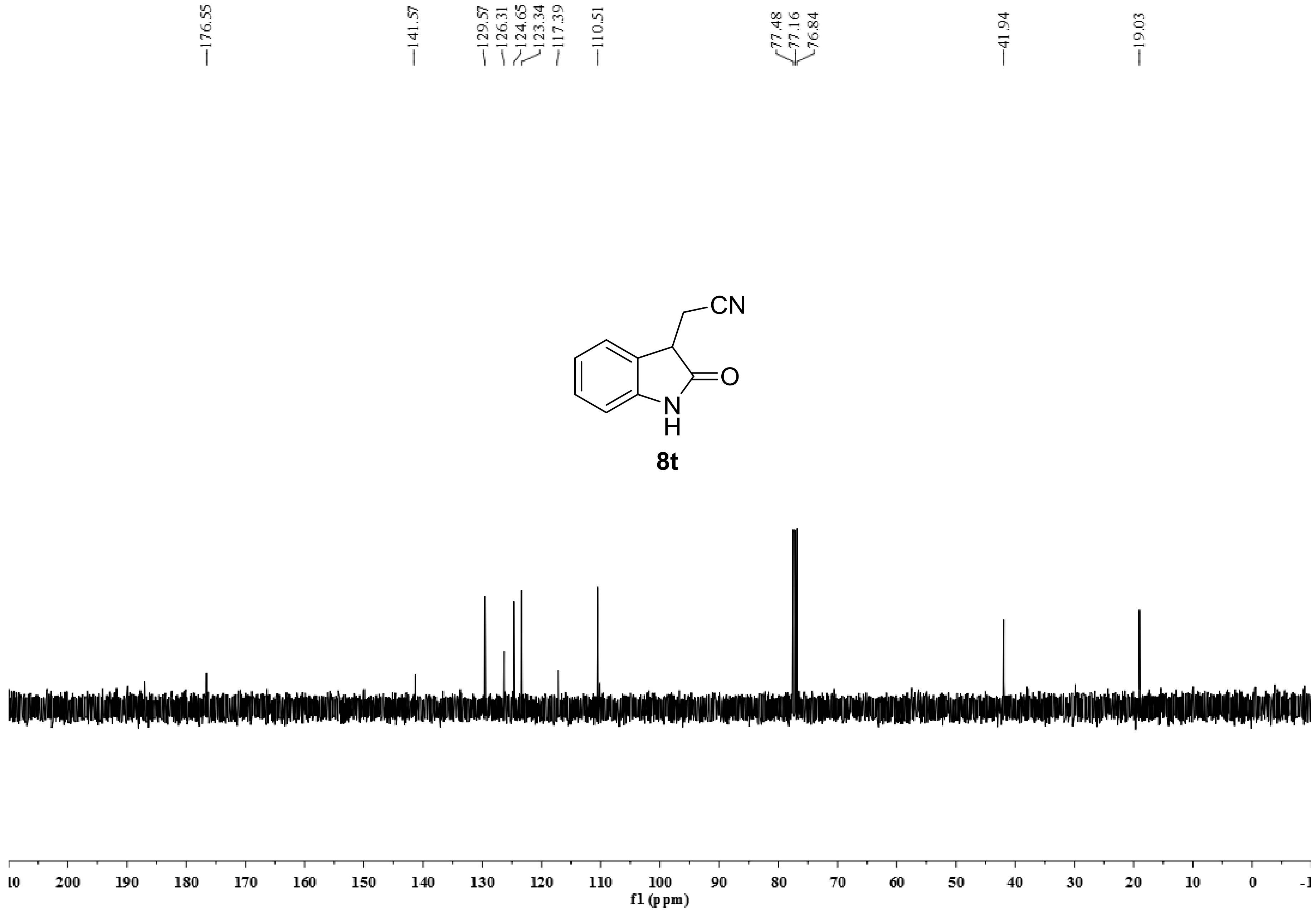

Supplementary Figure 177. ^{13}C-NMR of 8t

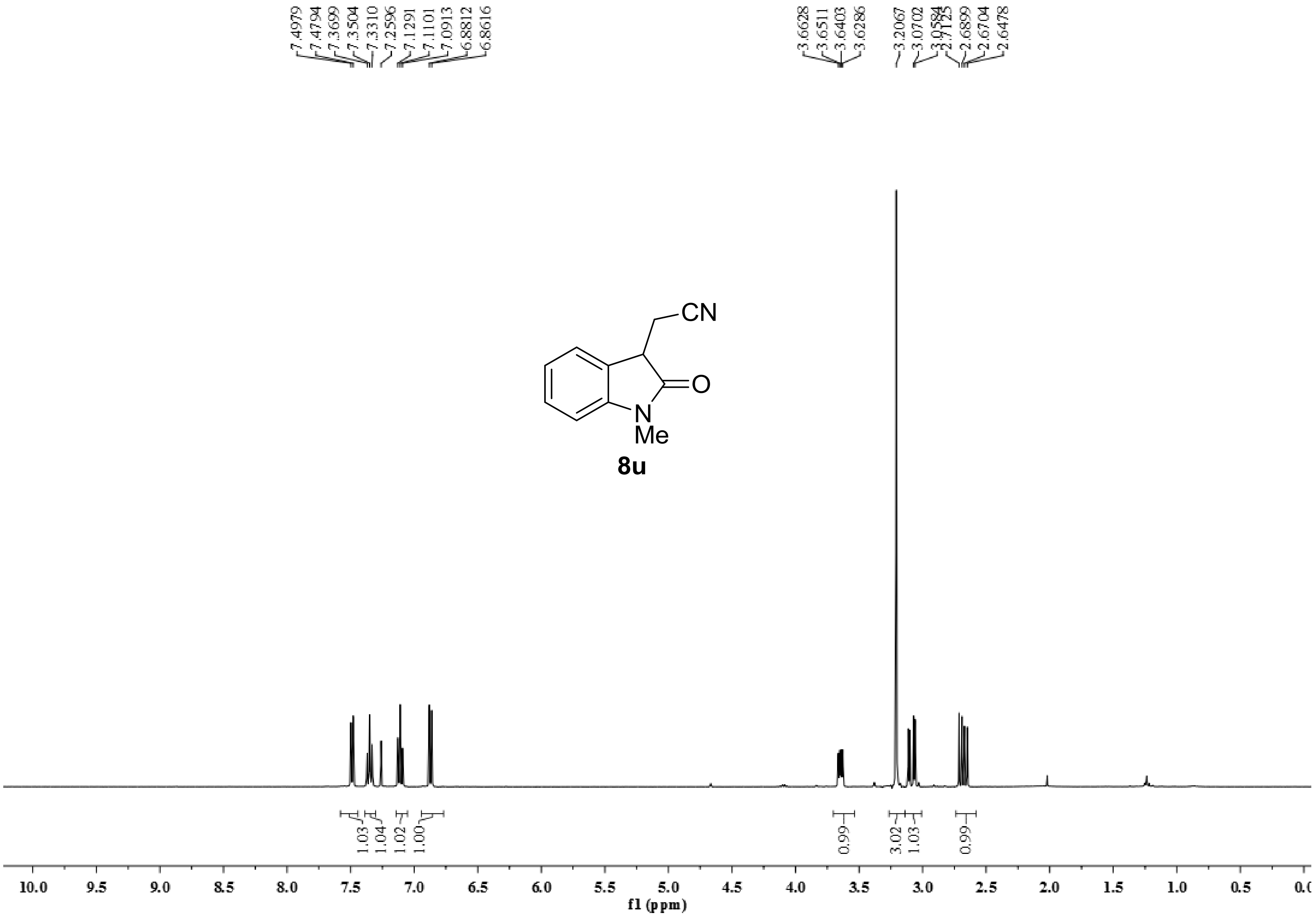

Supplementary Figure 178. ^{1}H-NMR of 8u

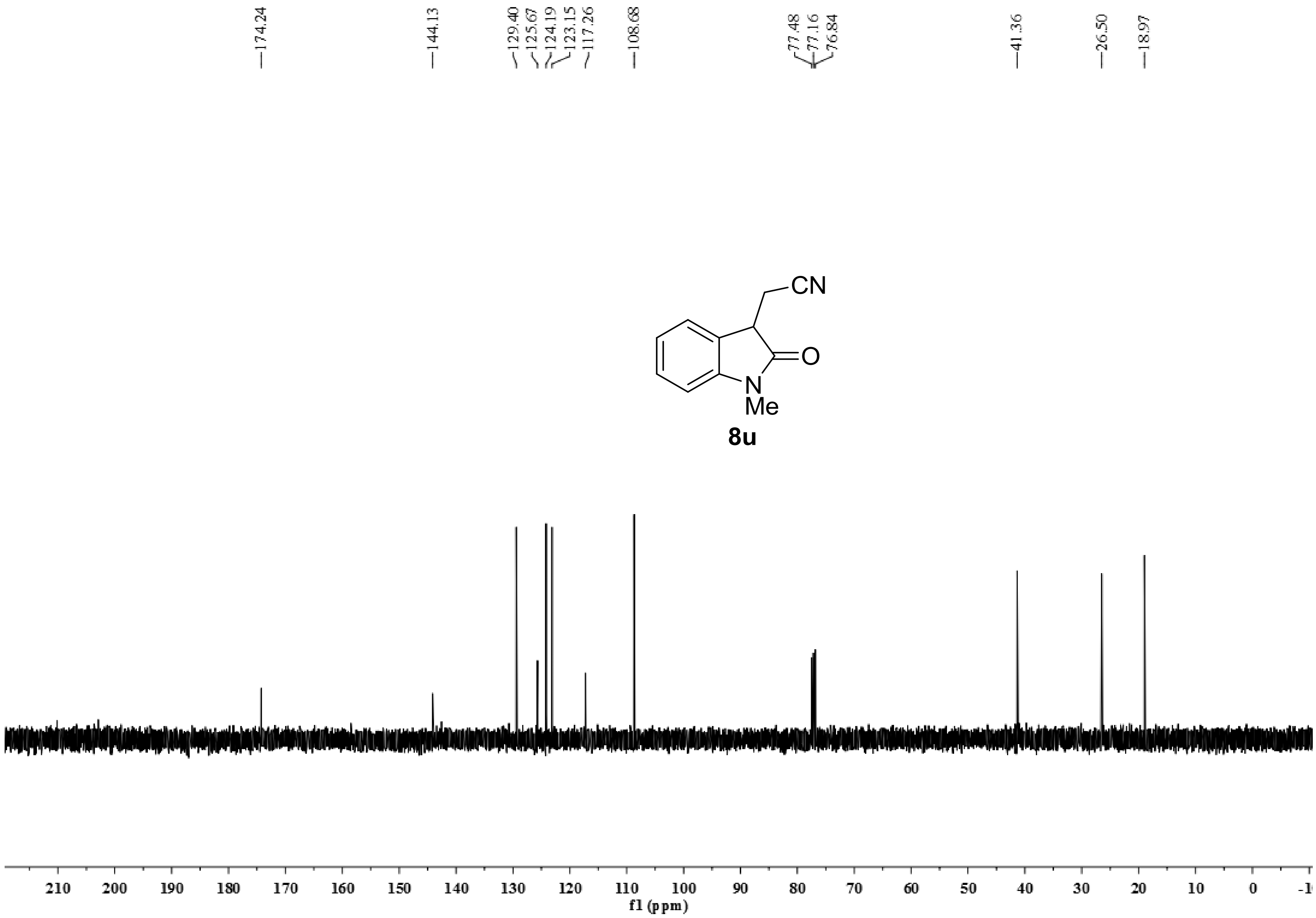

Supplementary Figure 179. ^{13}C-NMR of 8u

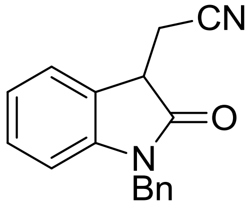

8v

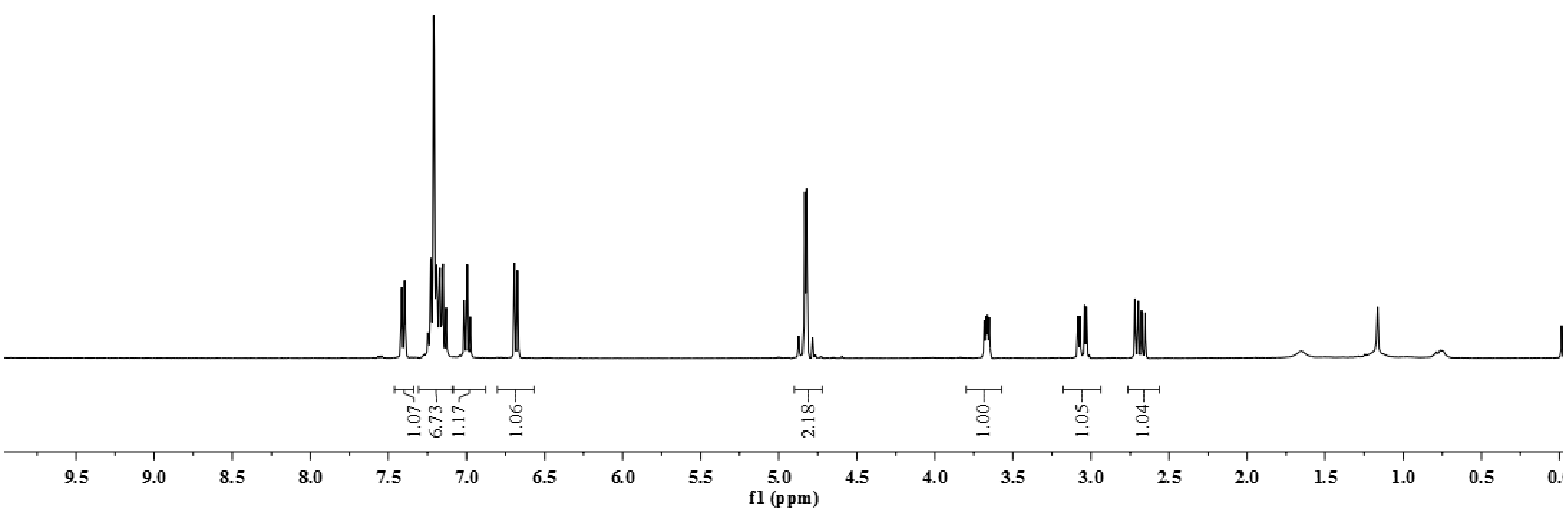

Supplementary Figure 180. ^{1}H-NMR of 8v

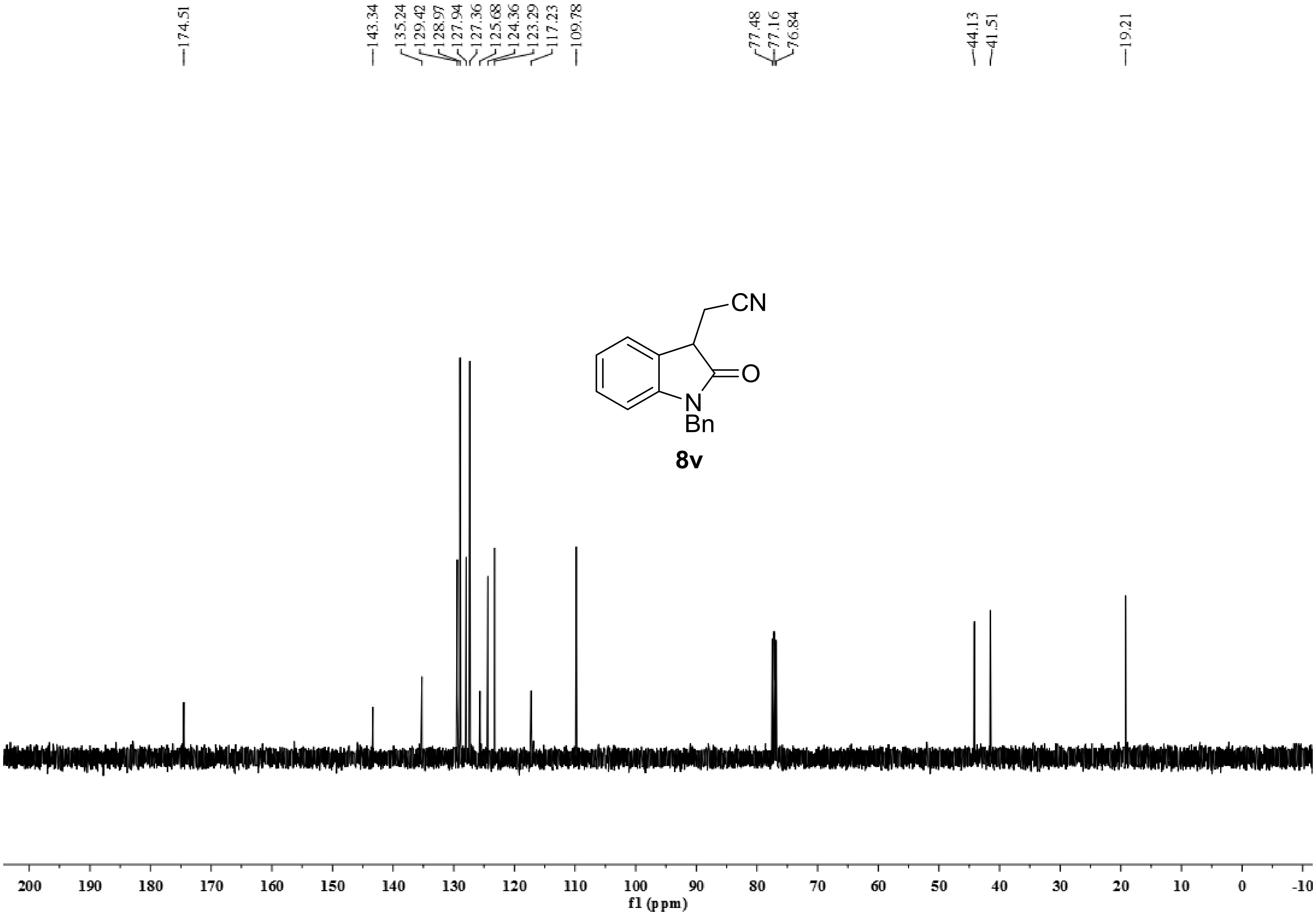

Supplementary Figure 181. ^{13}C-NMR of 8v

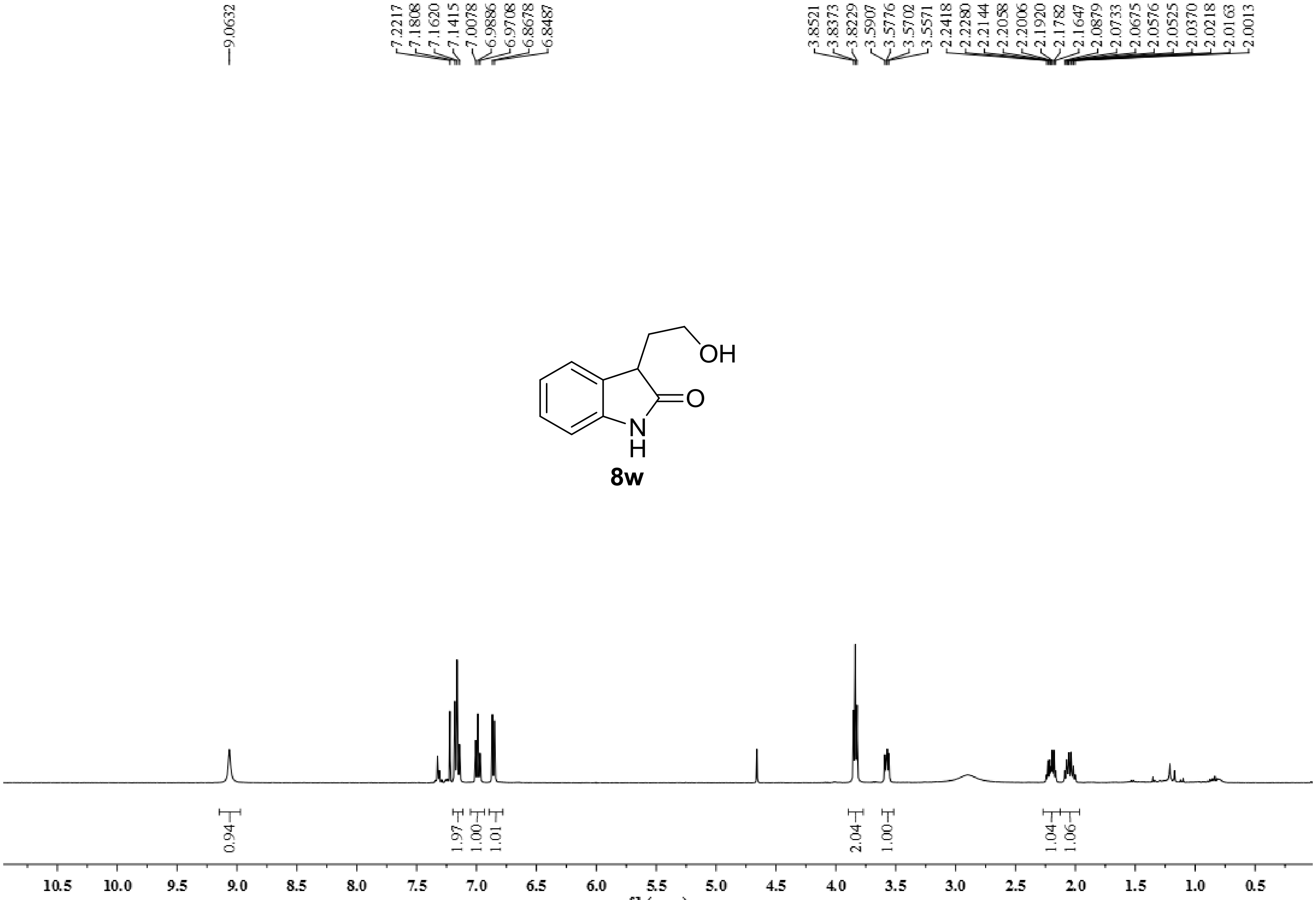

Supplementary Figure 182. ^{1}H-NMR of 8w

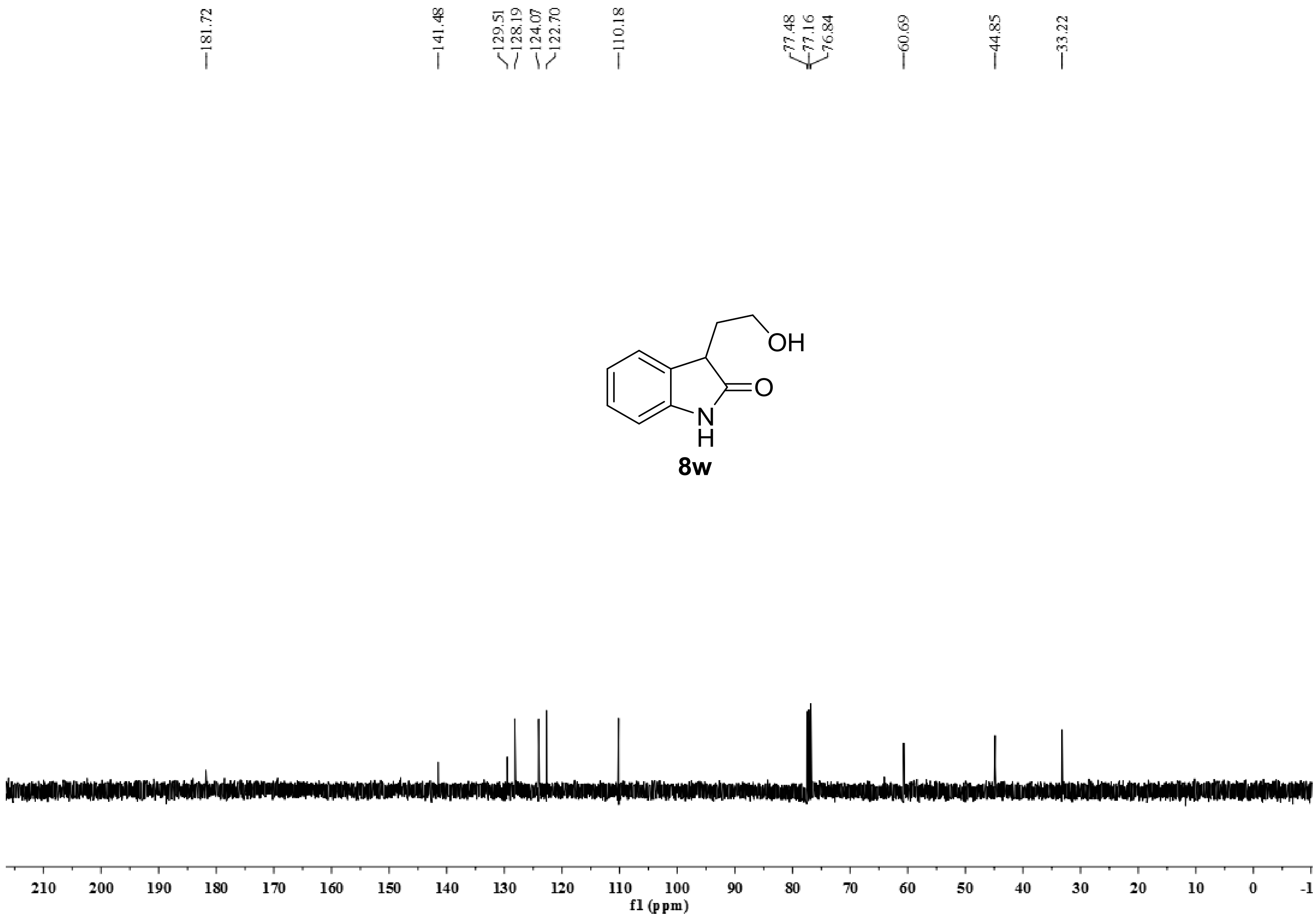

Supplementary Figure 183. ^{13}C-NMR of 8w

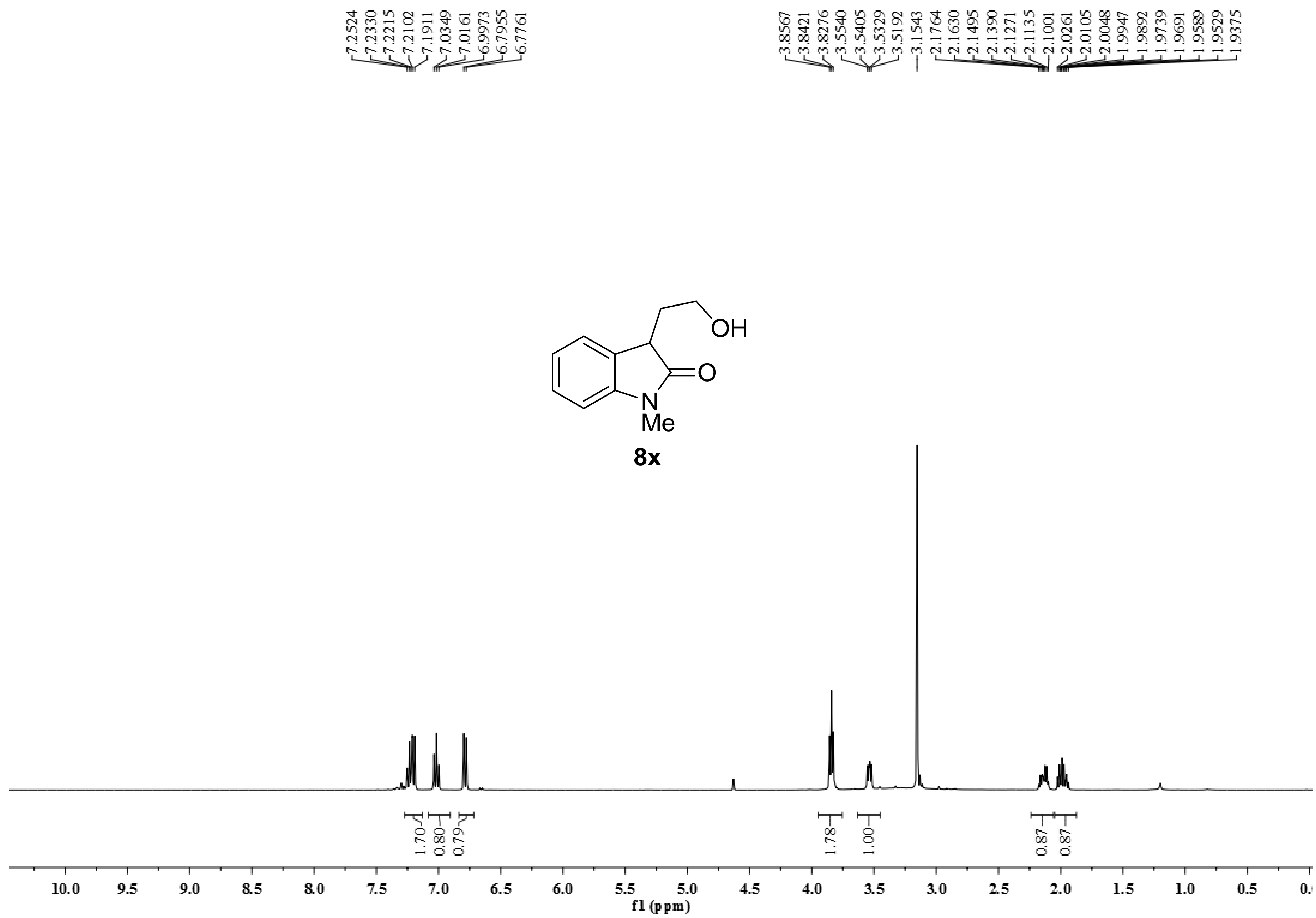

Supplementary Figure 184. ¹H-NMR of 8x

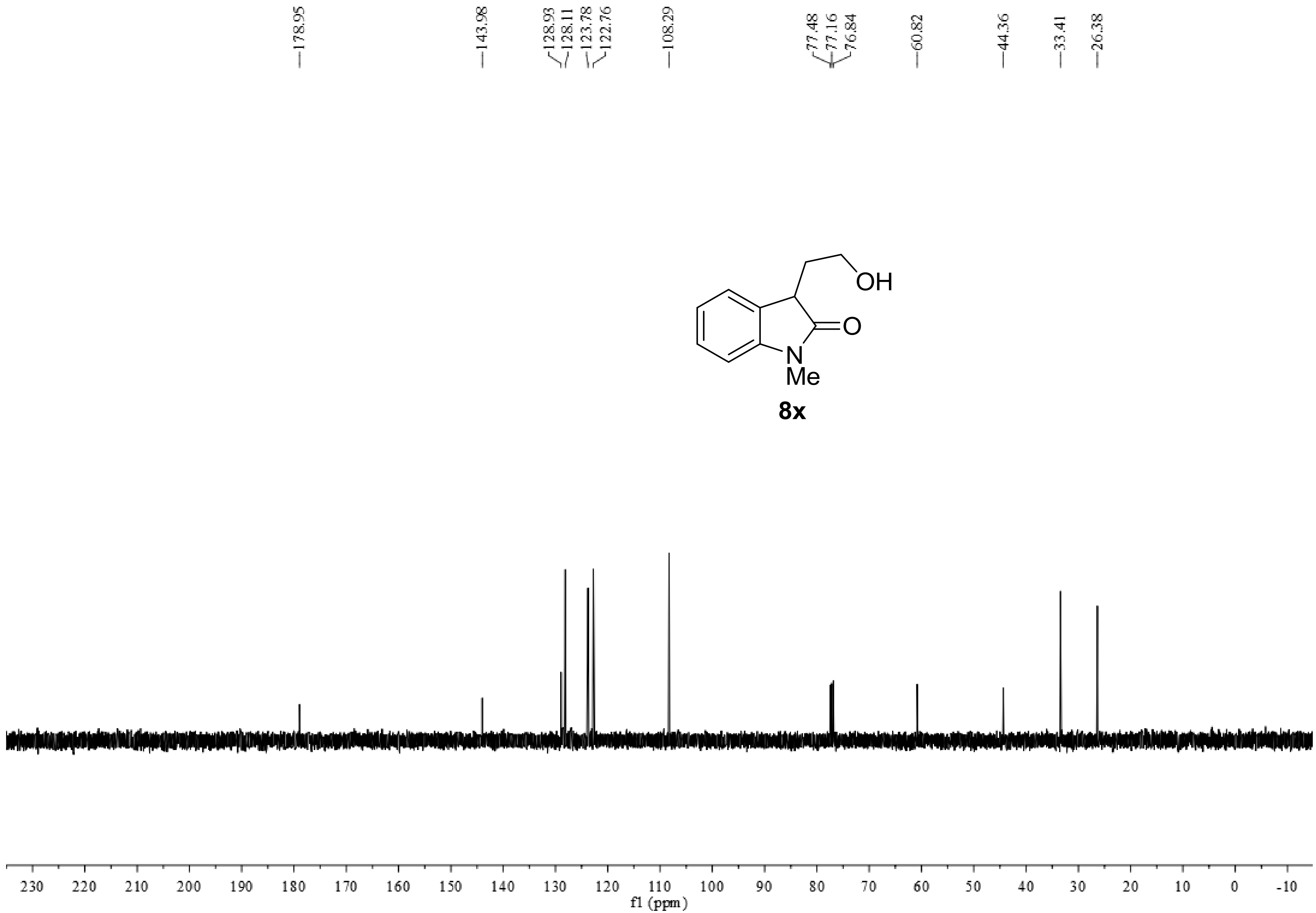

Supplementary Figure 185. ^{13}C-NMR of 8x

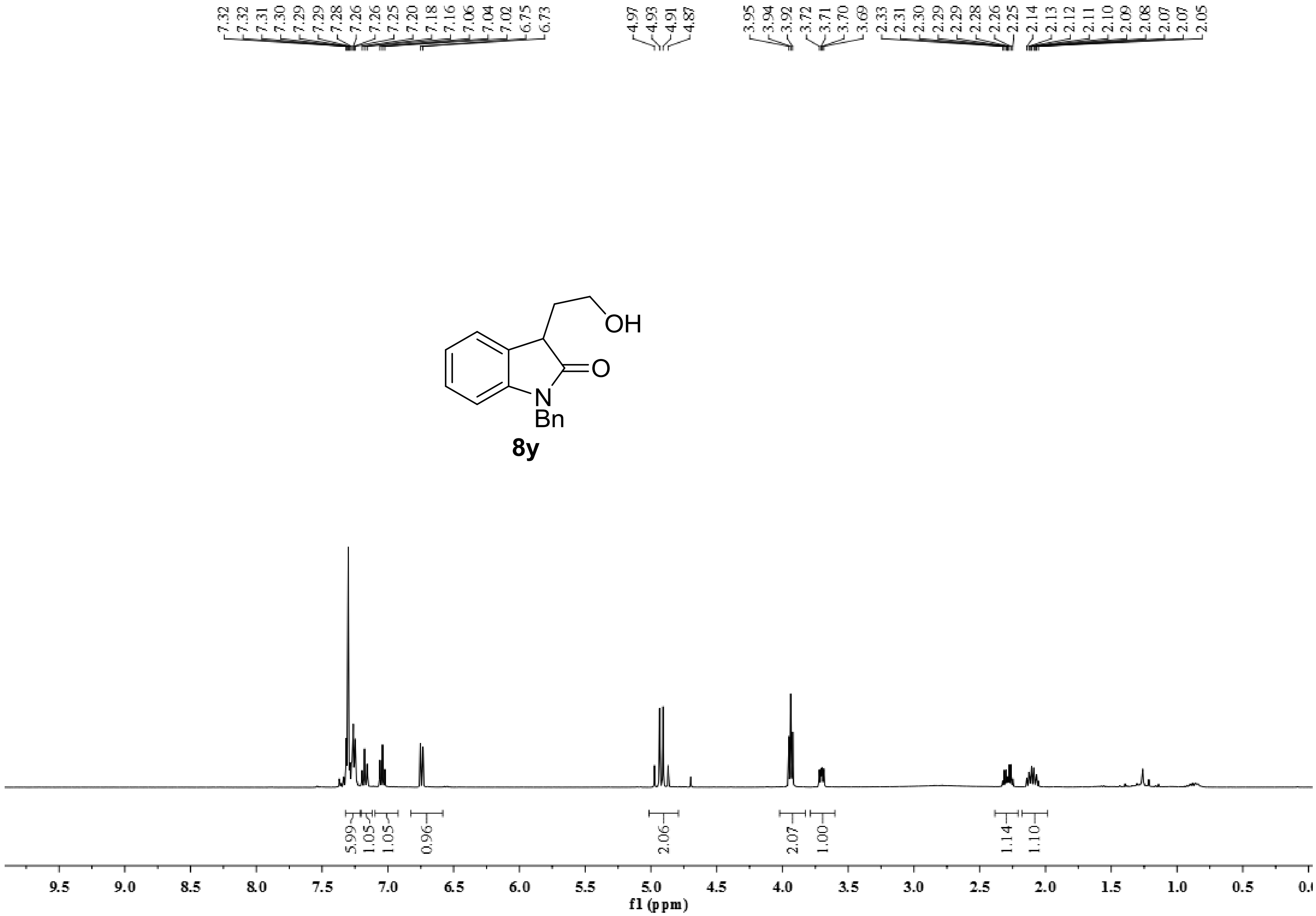

Supplementary Figure 186. ^{1}H-NMR of 8y

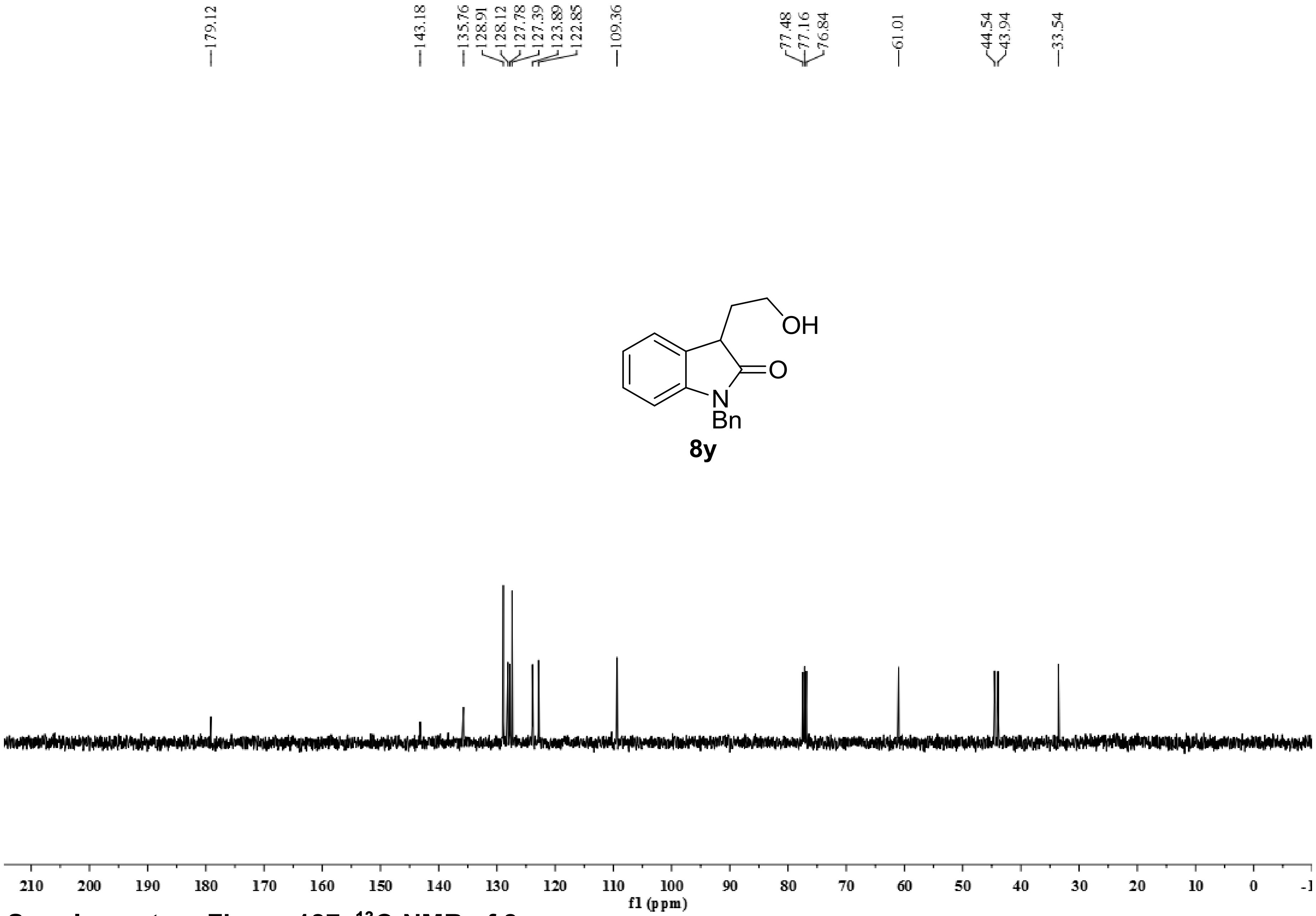

Supplementary Figure 187. ^{13}C-NMR of 8y

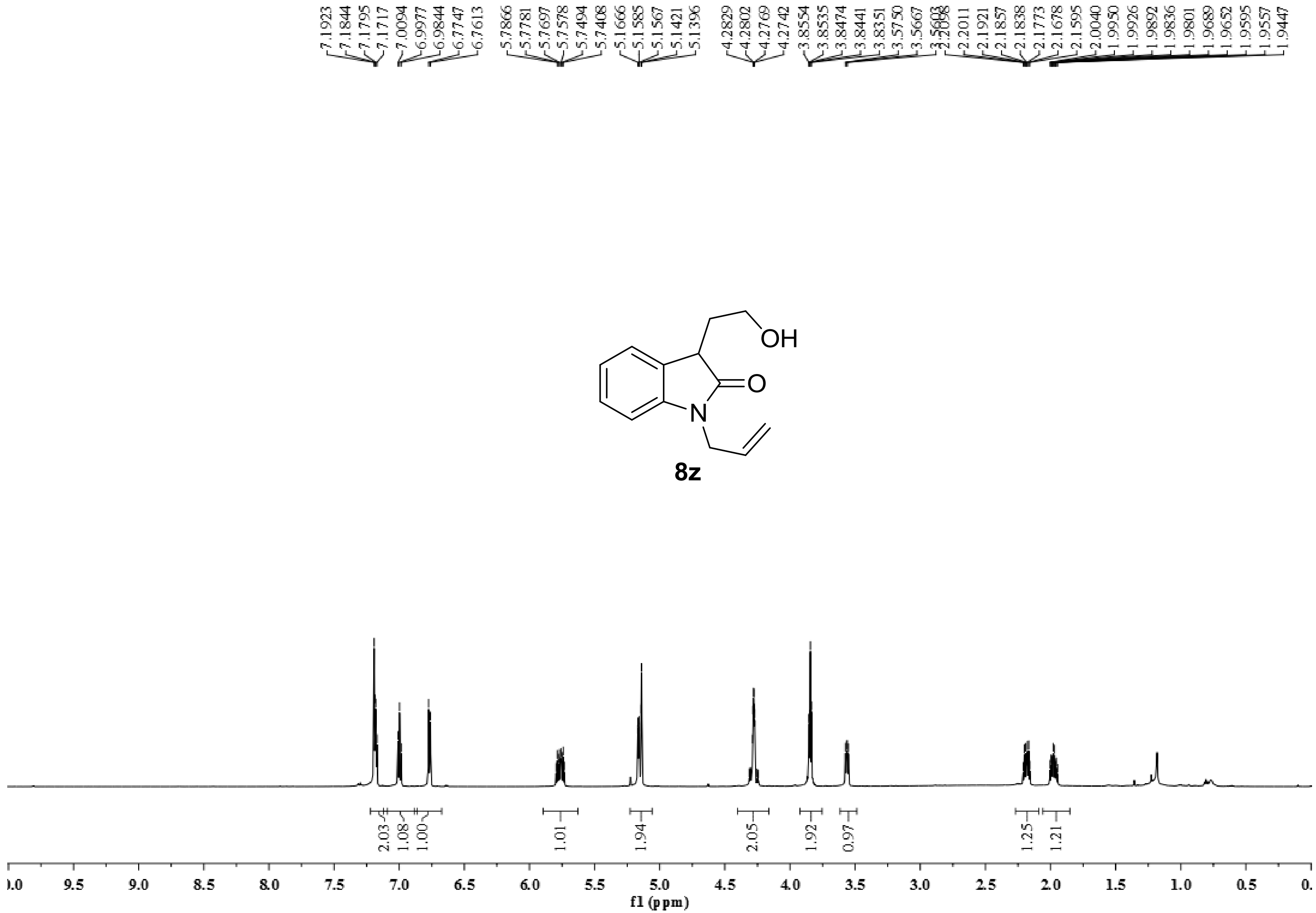

Supplementary Figure 188. ^{1}H-NMR of 8z

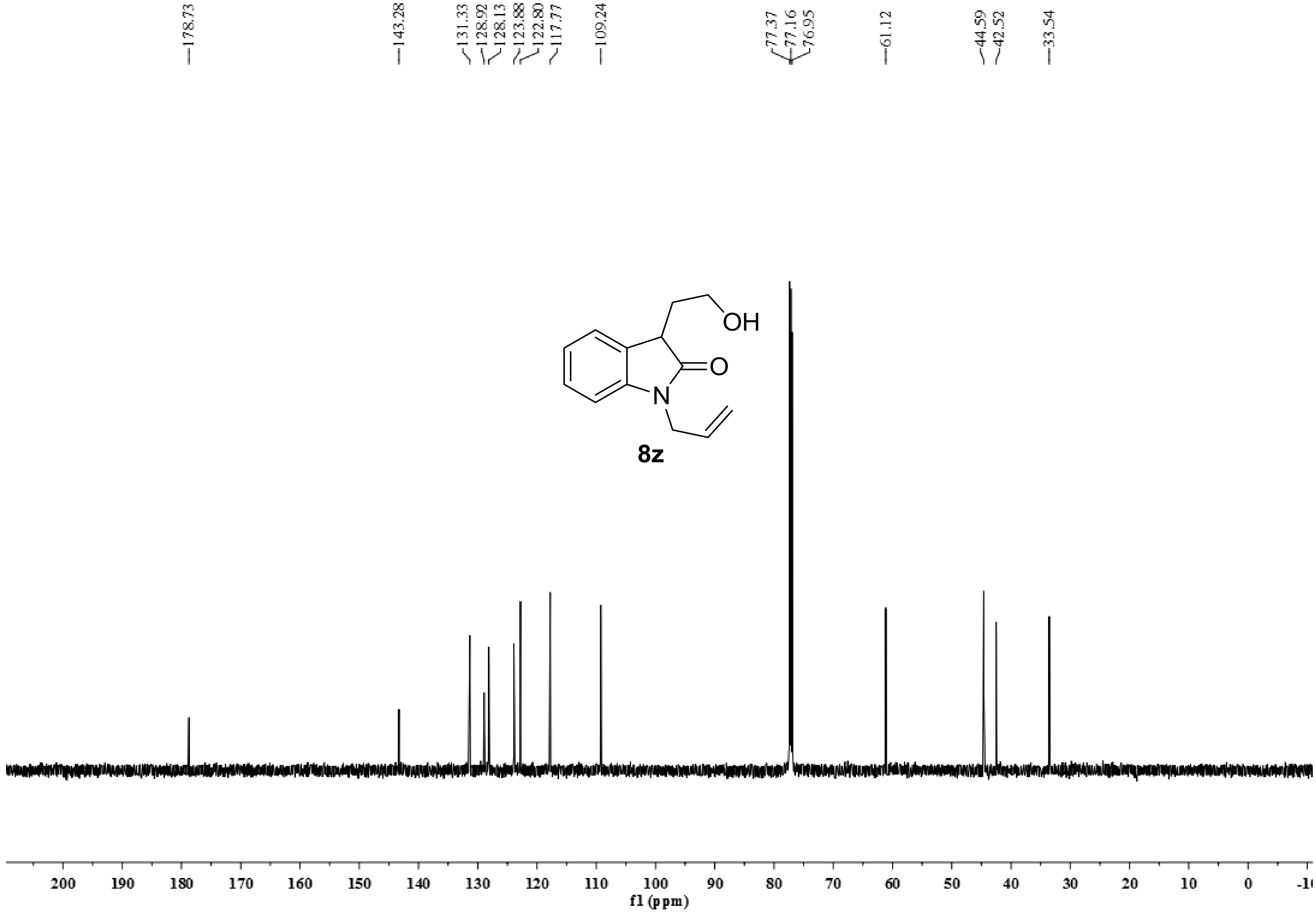

8z

Supplementary Figure 189. ^{13}C-NMR of 8z

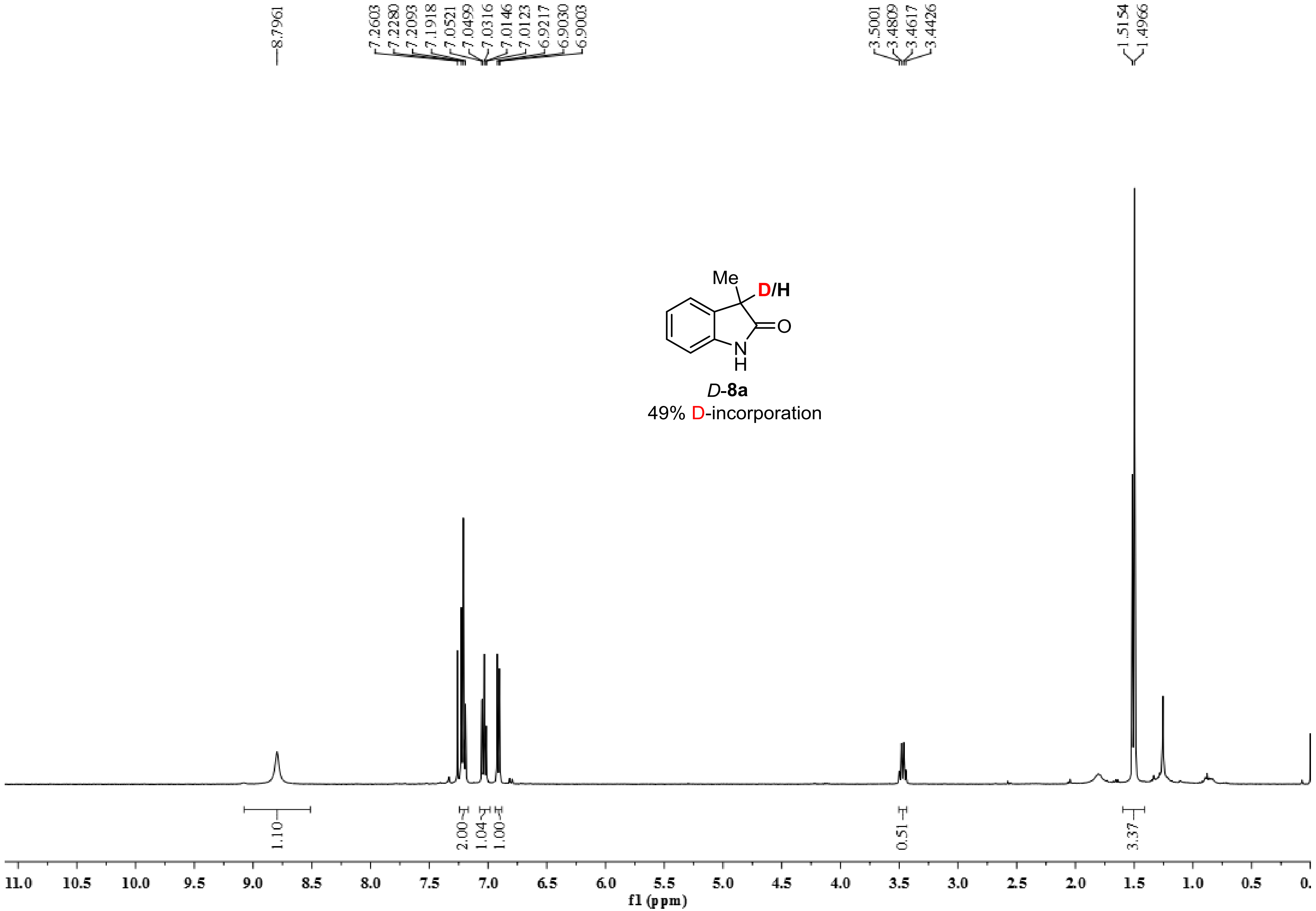

Supplementary Figure 190. ^{1}H-NMR of 49% *D*-8a

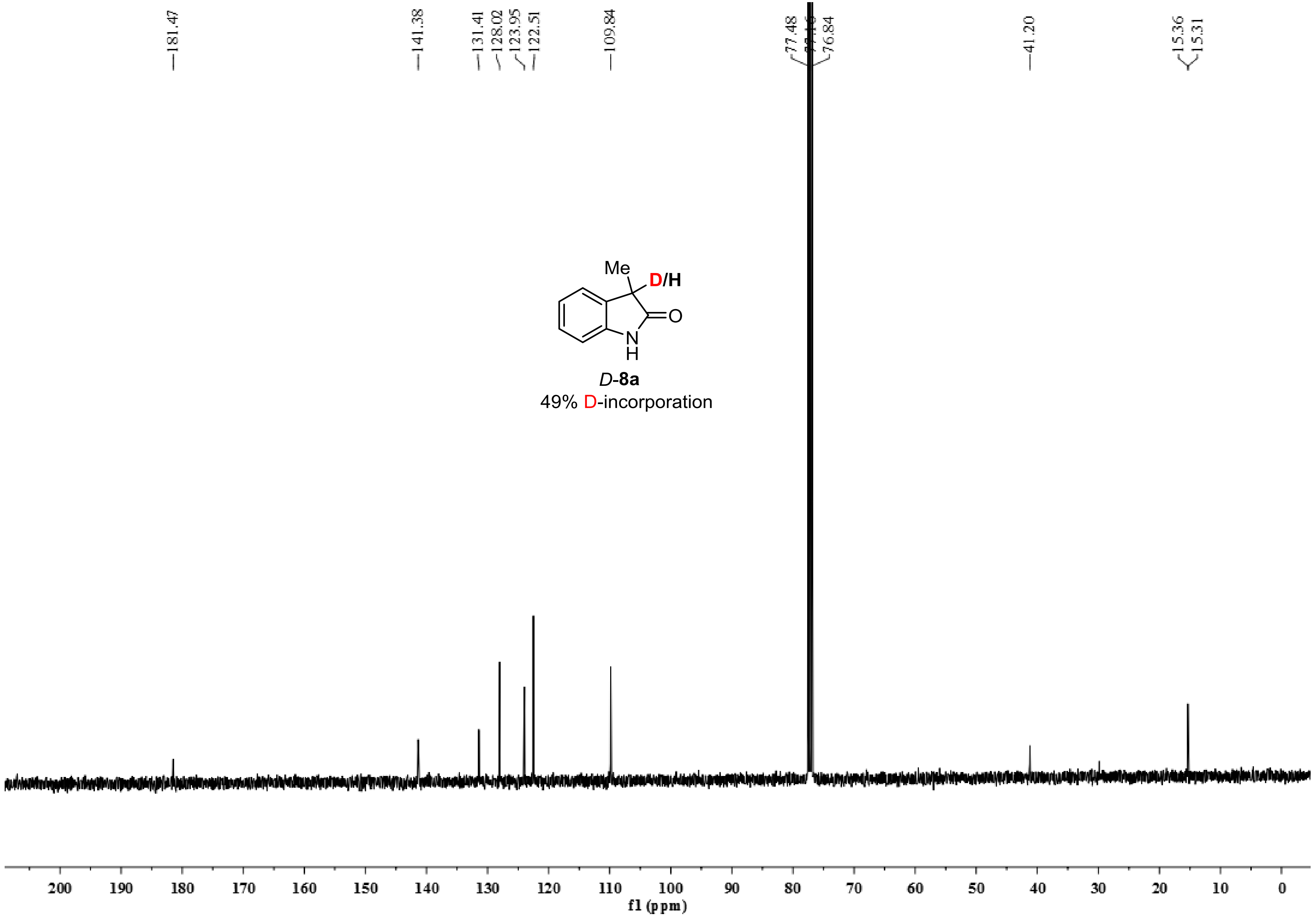

Supplementary Figure 191. ^{13}C-NMR of 49% *D*-8a

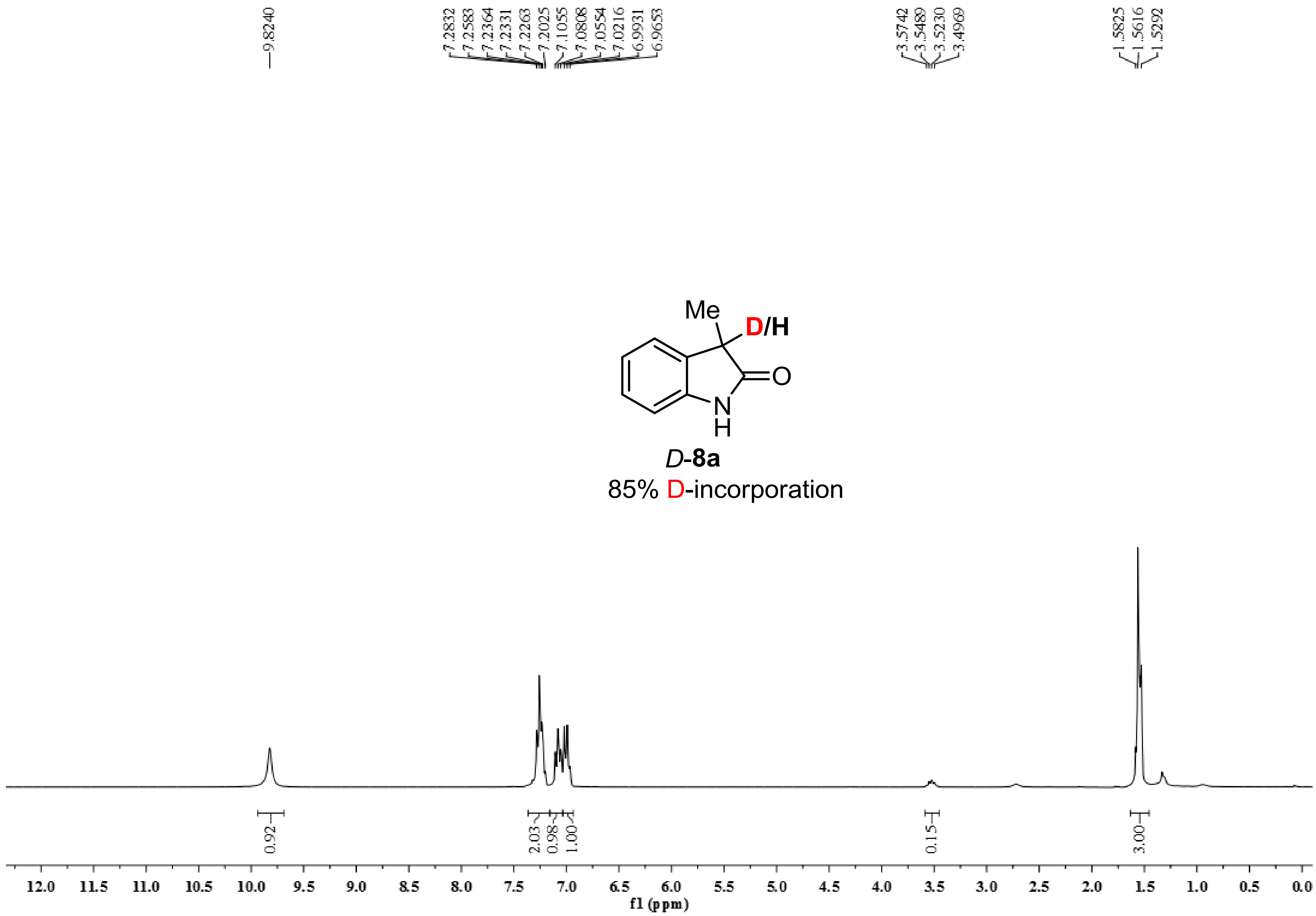

Supplementary Figure 192. ^{1}H-NMR of 85% *D*-8a

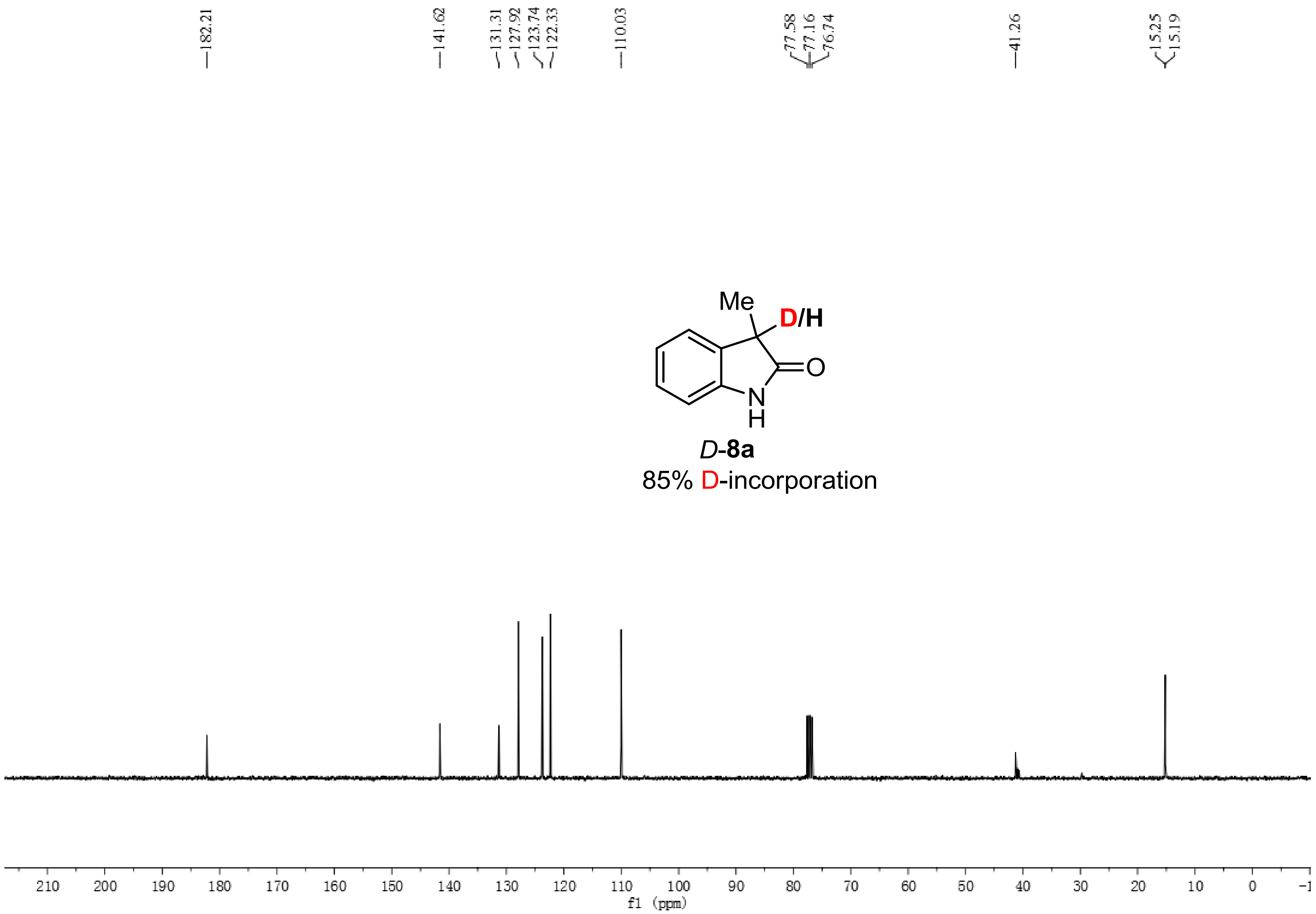

Supplementary Figure 193. ^{13}C-NMR of 85% *D*-8a

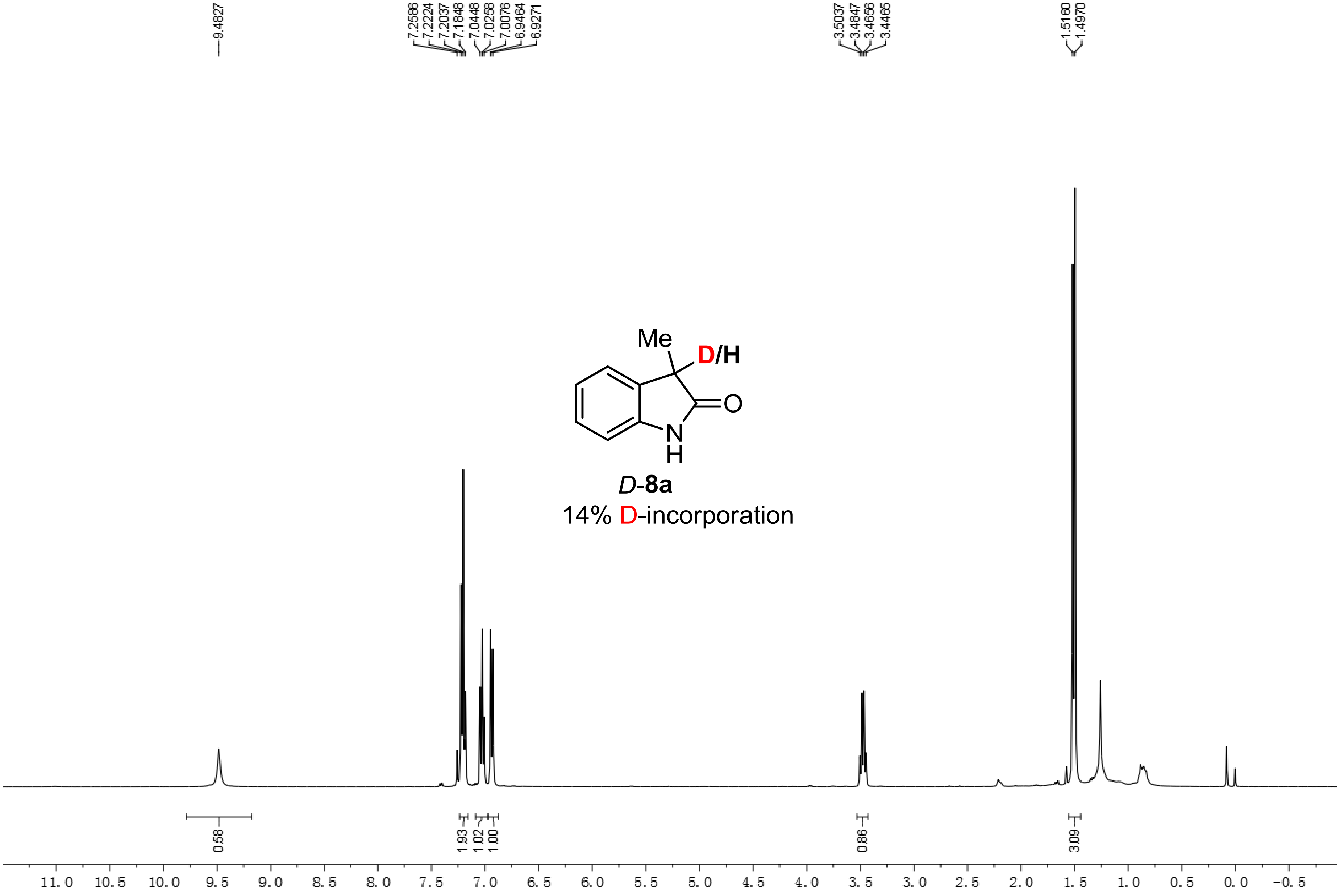

Supplementary Figure 194. ^{1}H-NMR of 14% *D*-8a

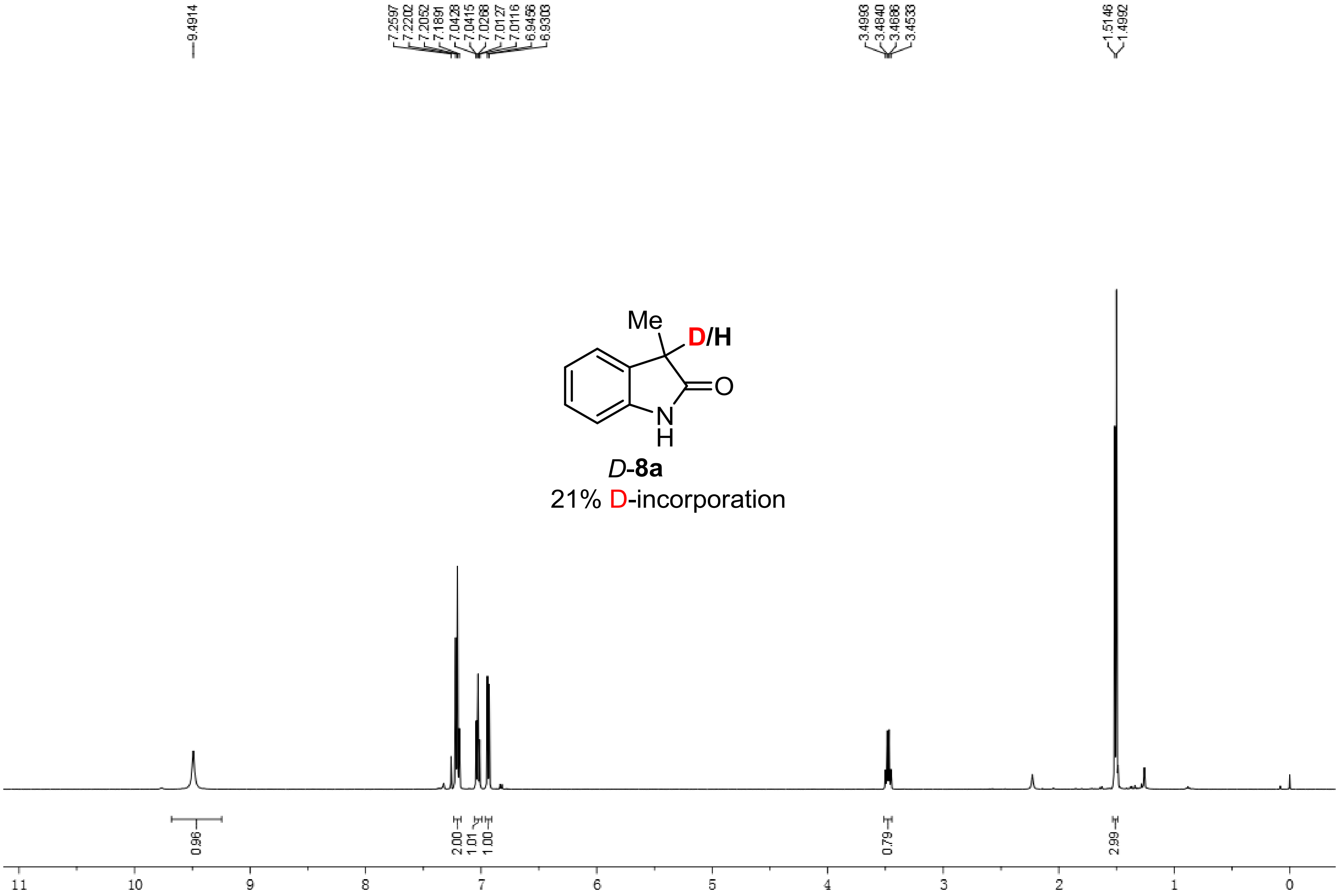

Supplementary Figure 195. ¹H-NMR of 14% *D*-8a

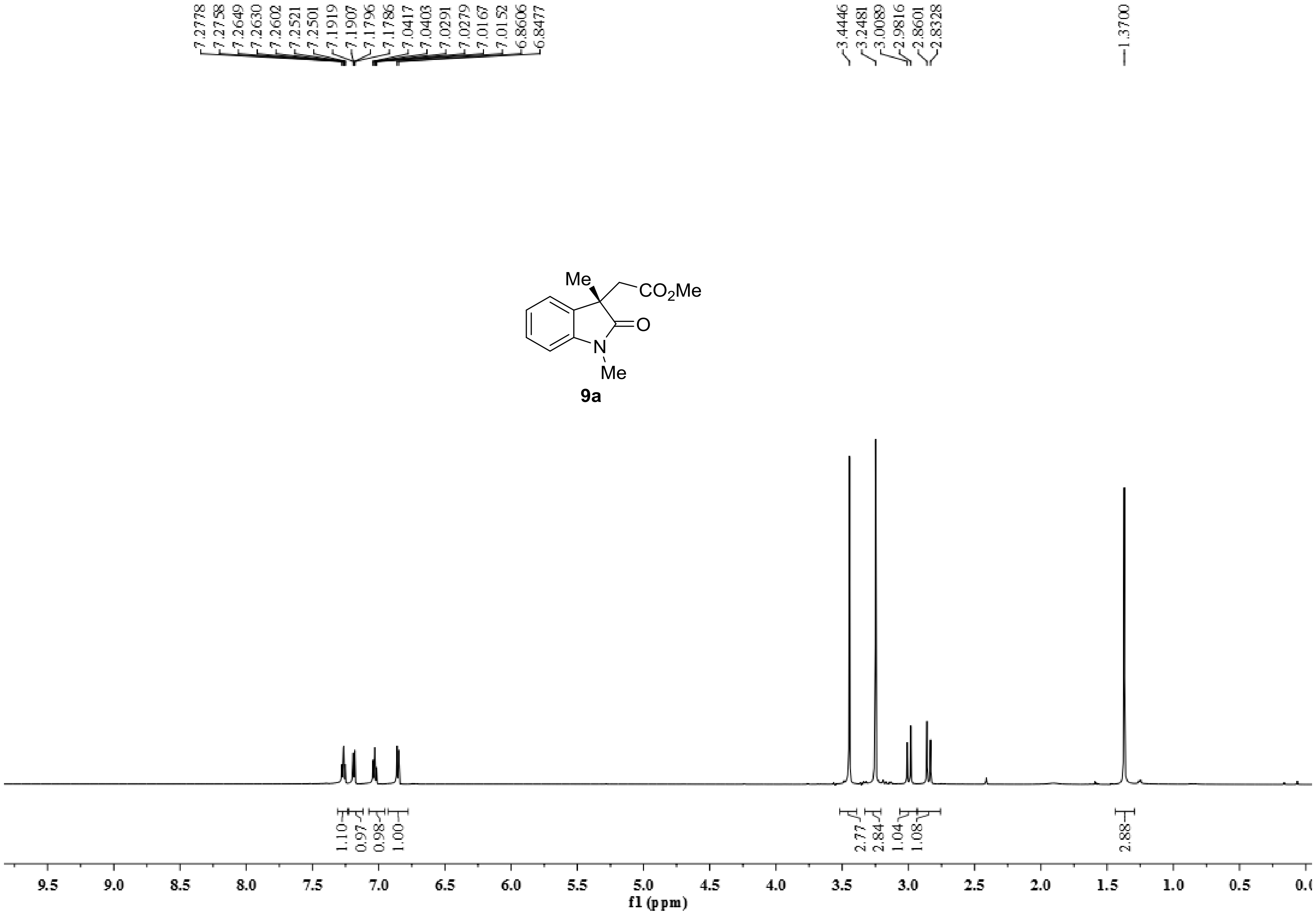

Supplementary Figure 196. ^{1}H-NMR of 9a

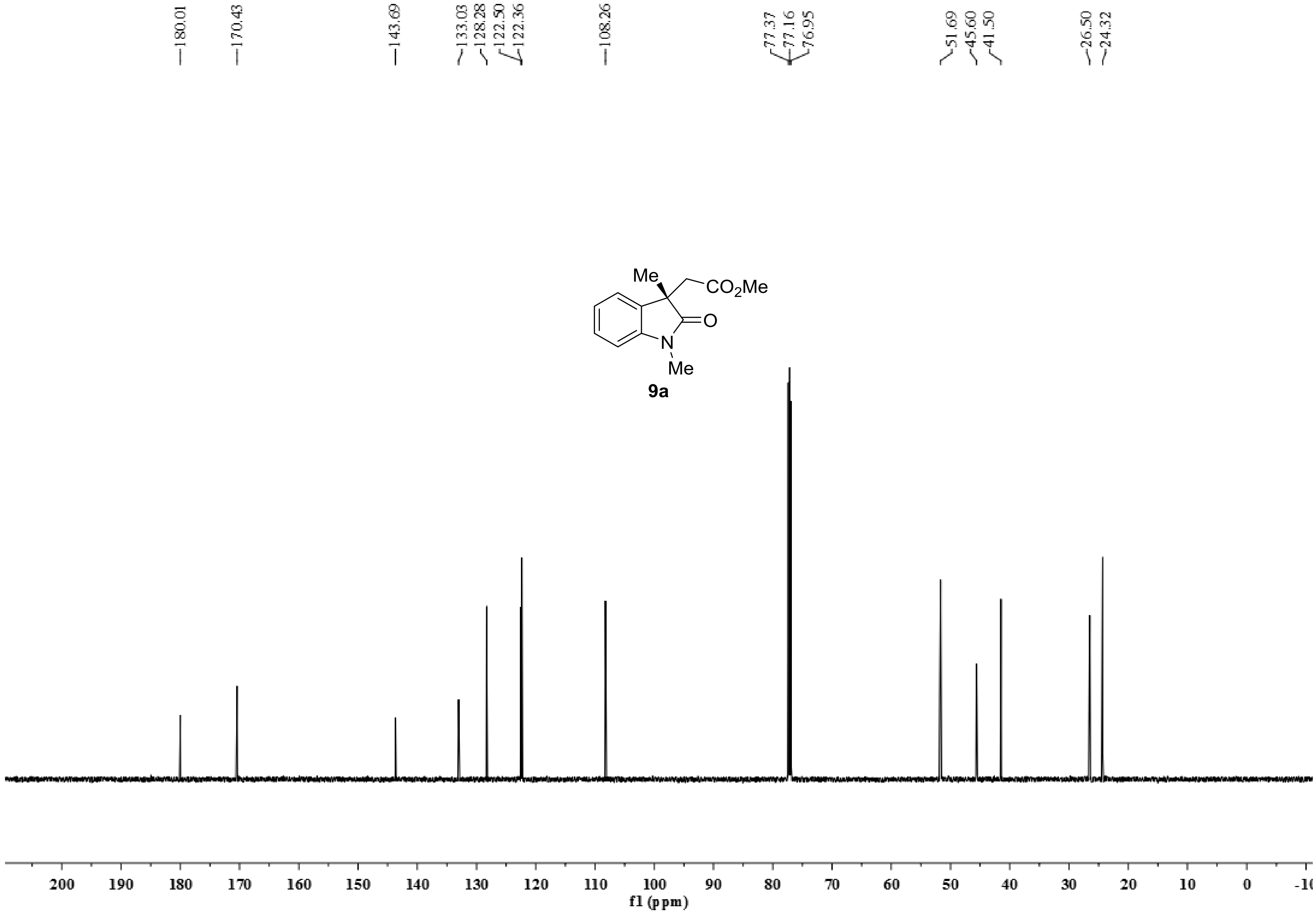

Supplementary Figure 197. ^{13}C-NMR of 9a

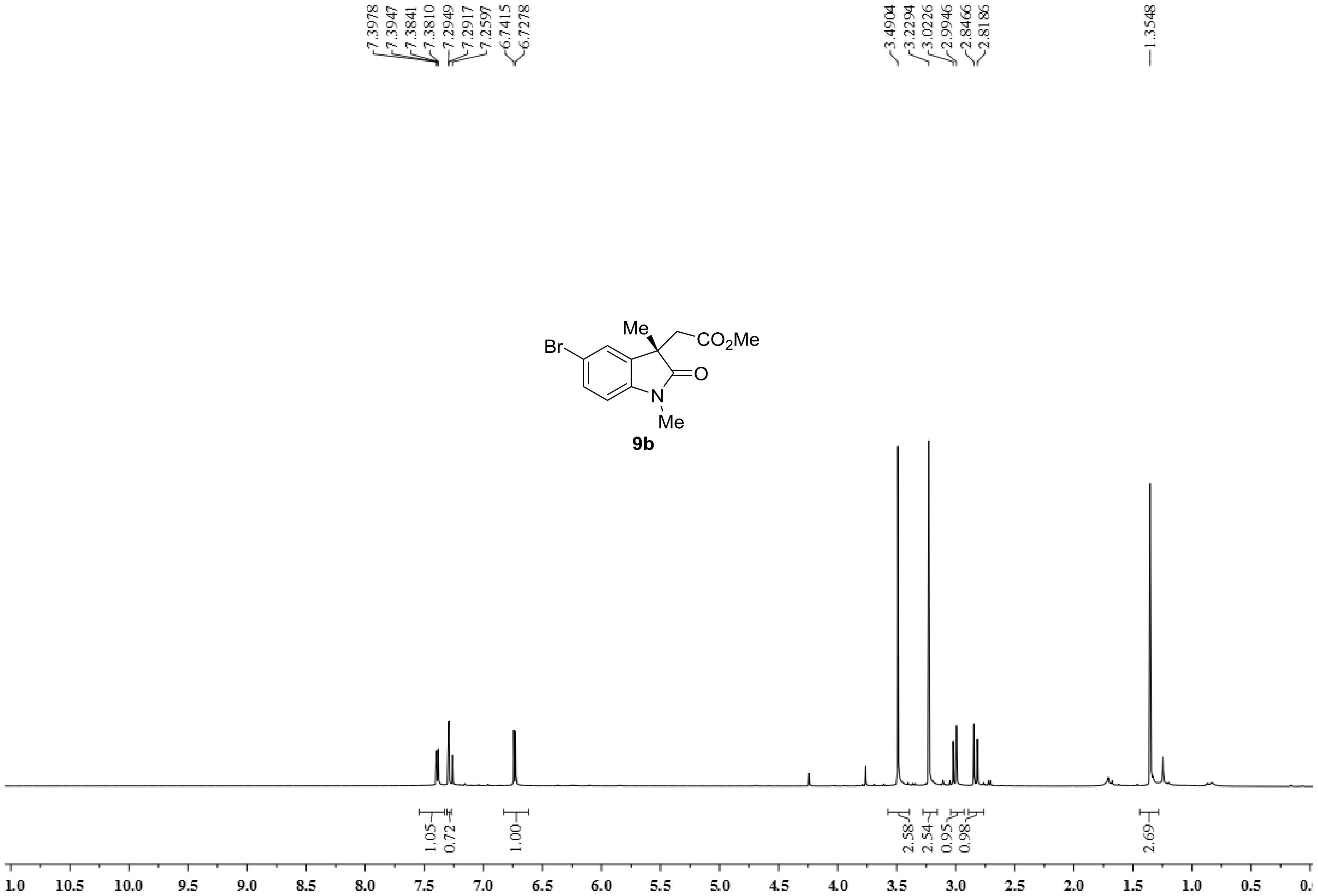

Supplementary Figure 198. ^{1}H-NMR of 9b

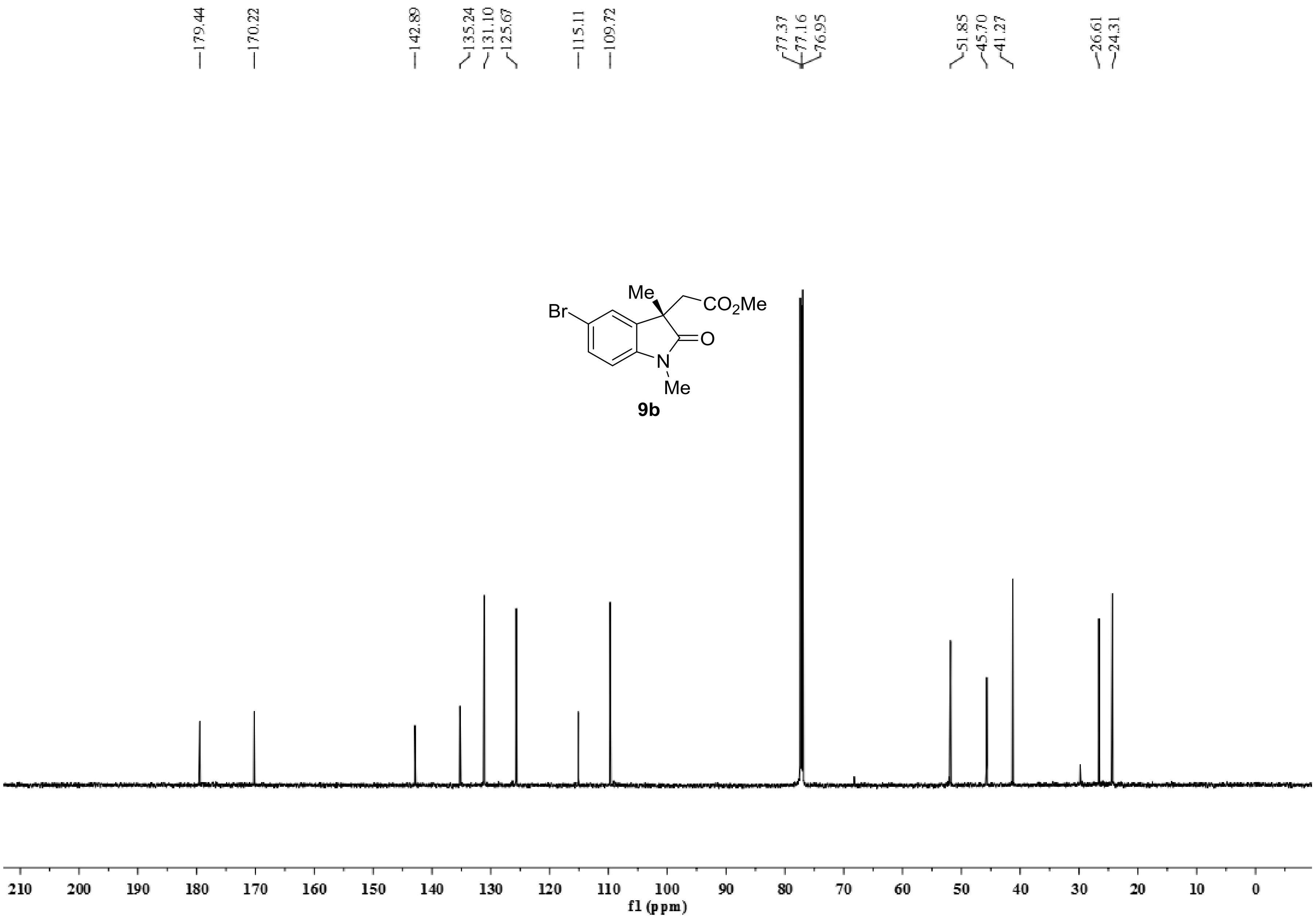

Supplementary Figure 199. ^{13}C-NMR of 9b

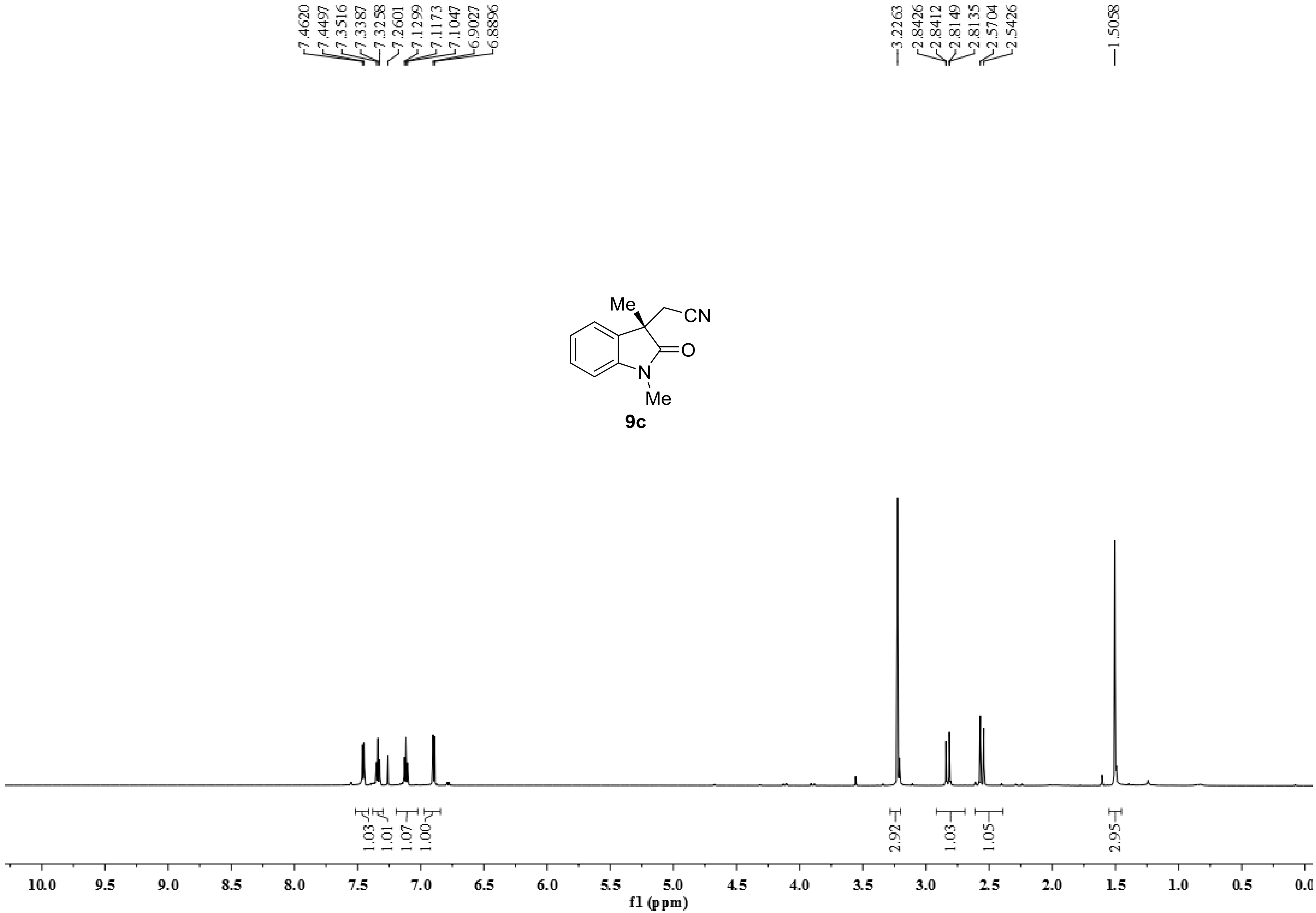

Supplementary Figure 200. ^{1}H-NMR of 9c

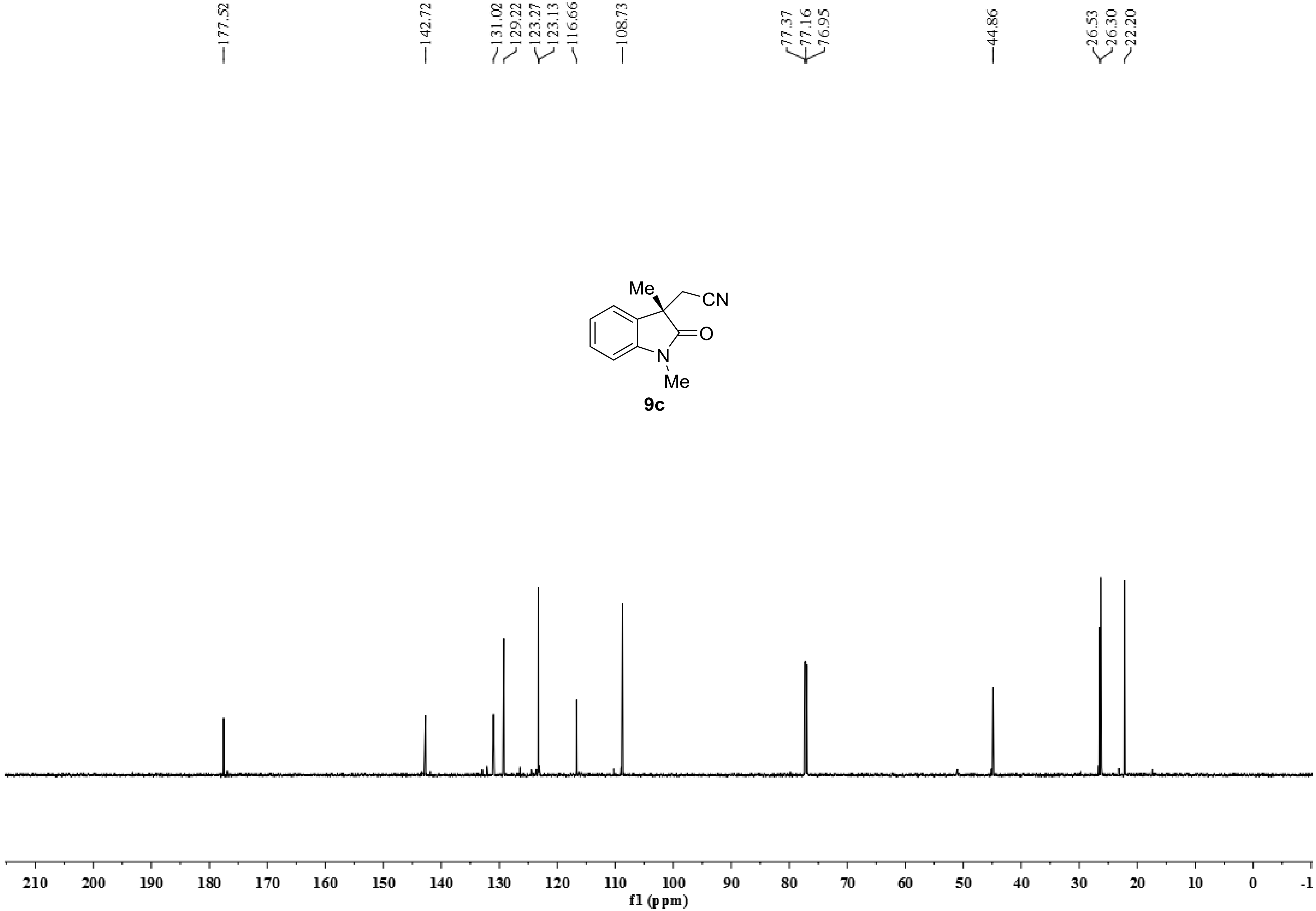

Supplementary Figure 201. ^{13}C-NMR of 9c

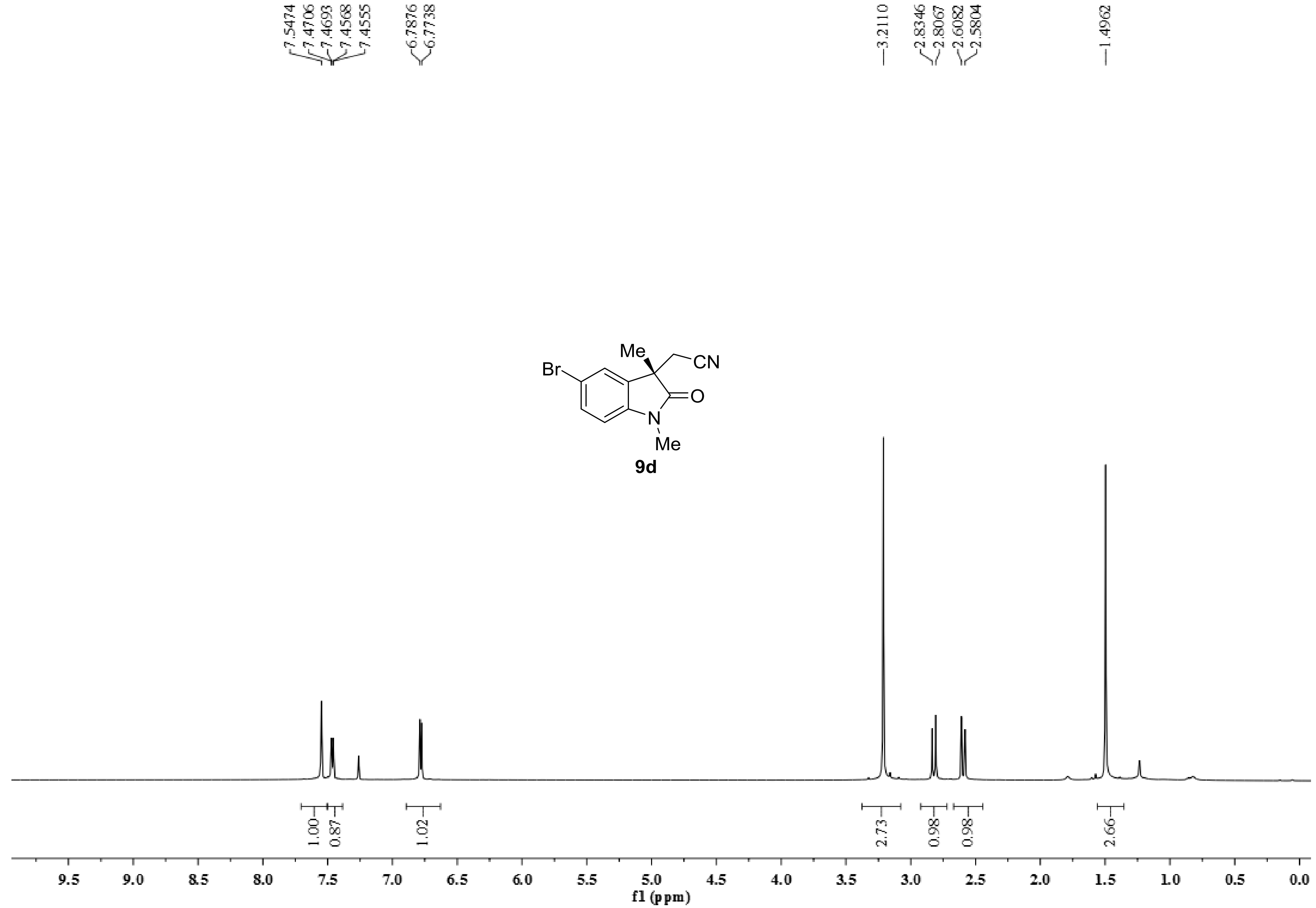

Supplementary Figure 202. ^{13}C-NMR of 9d

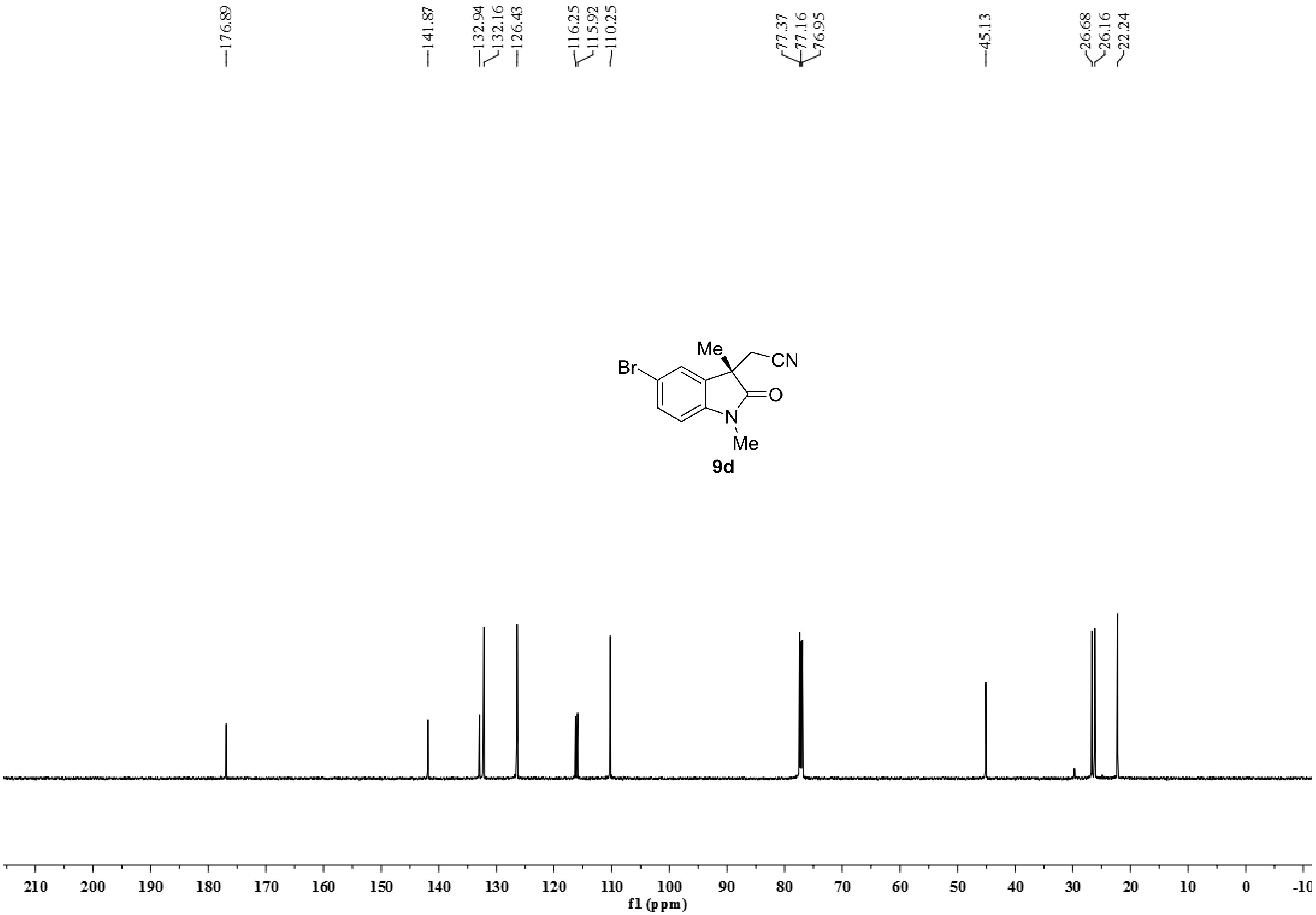

9d

Supplementary Figure 203. ^{13}C-NMR of 9d

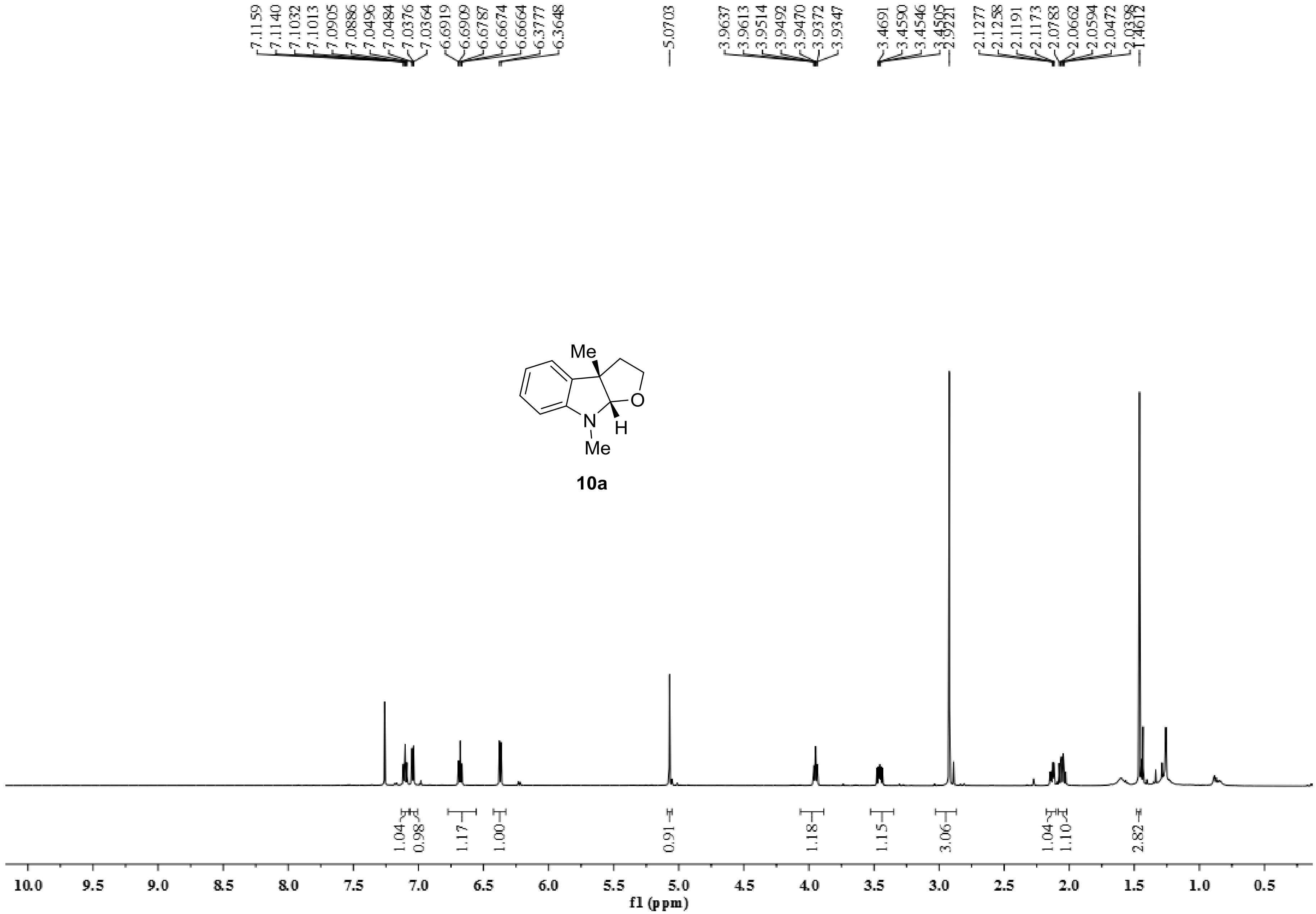

Supplementary Figure 204. ^{1}H-NMR of 10a

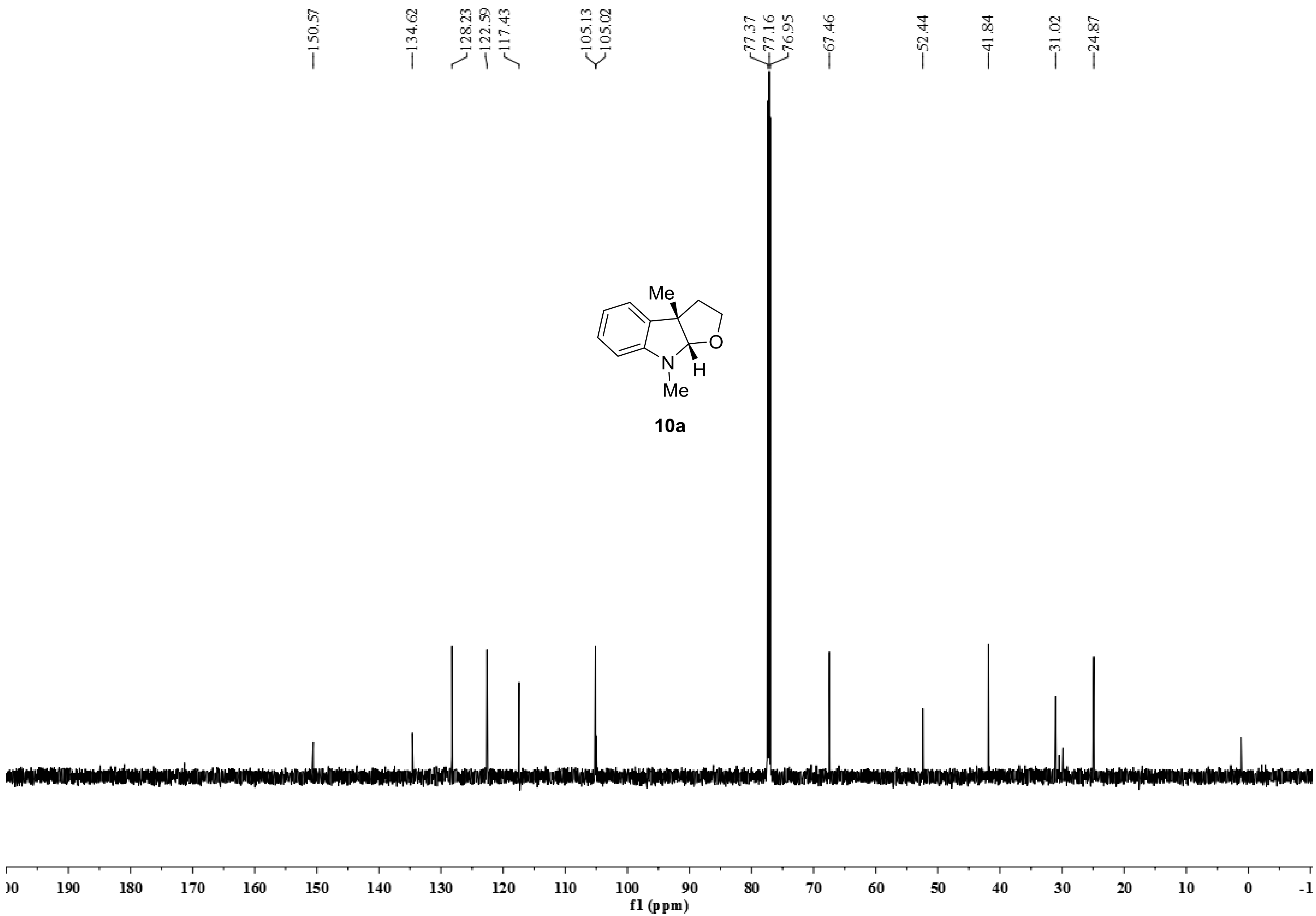

Supplementary Figure 205. ^{13}C-NMR of 10a

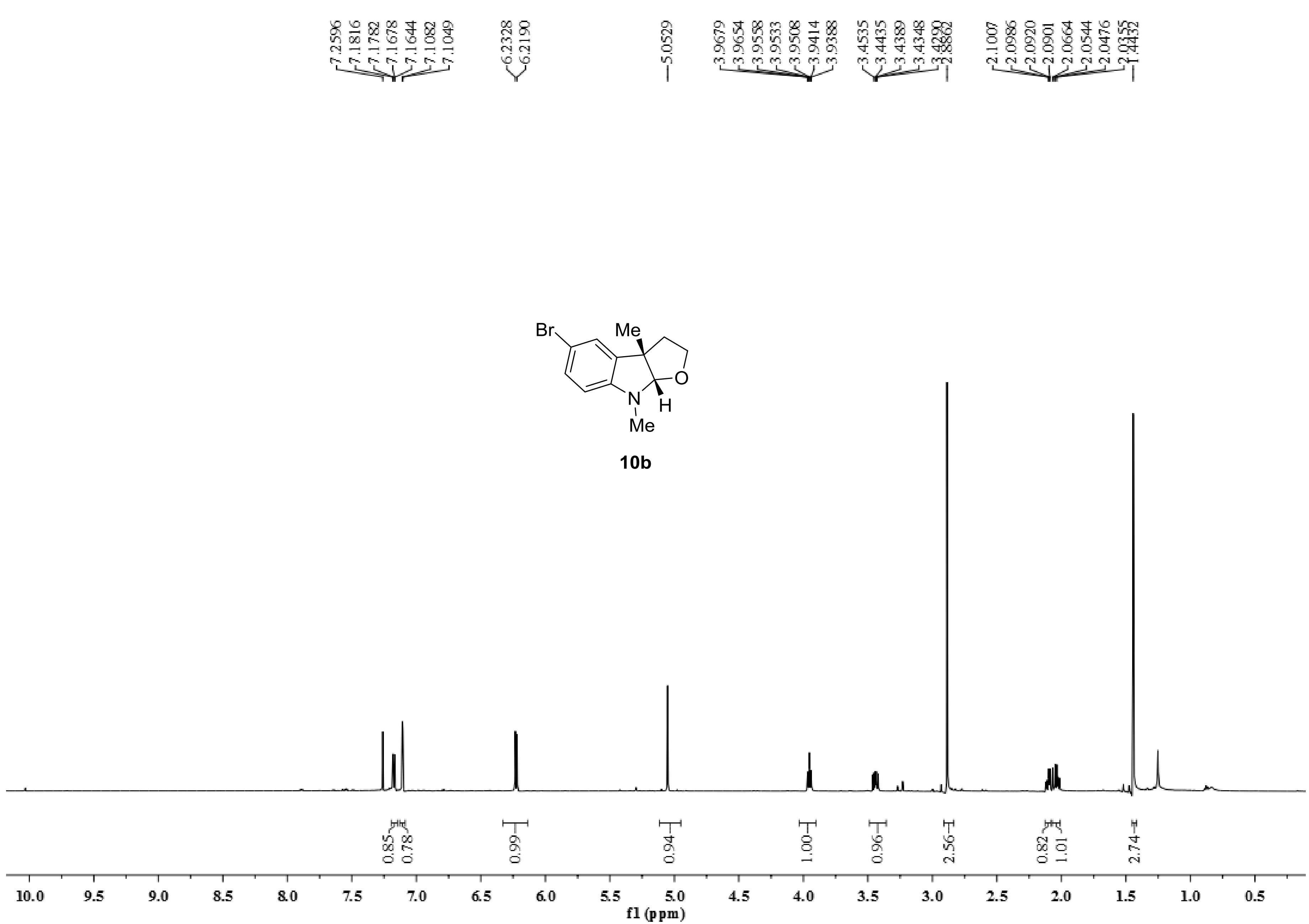

Supplementary Figure 206. ^{1}H-NMR of 10b

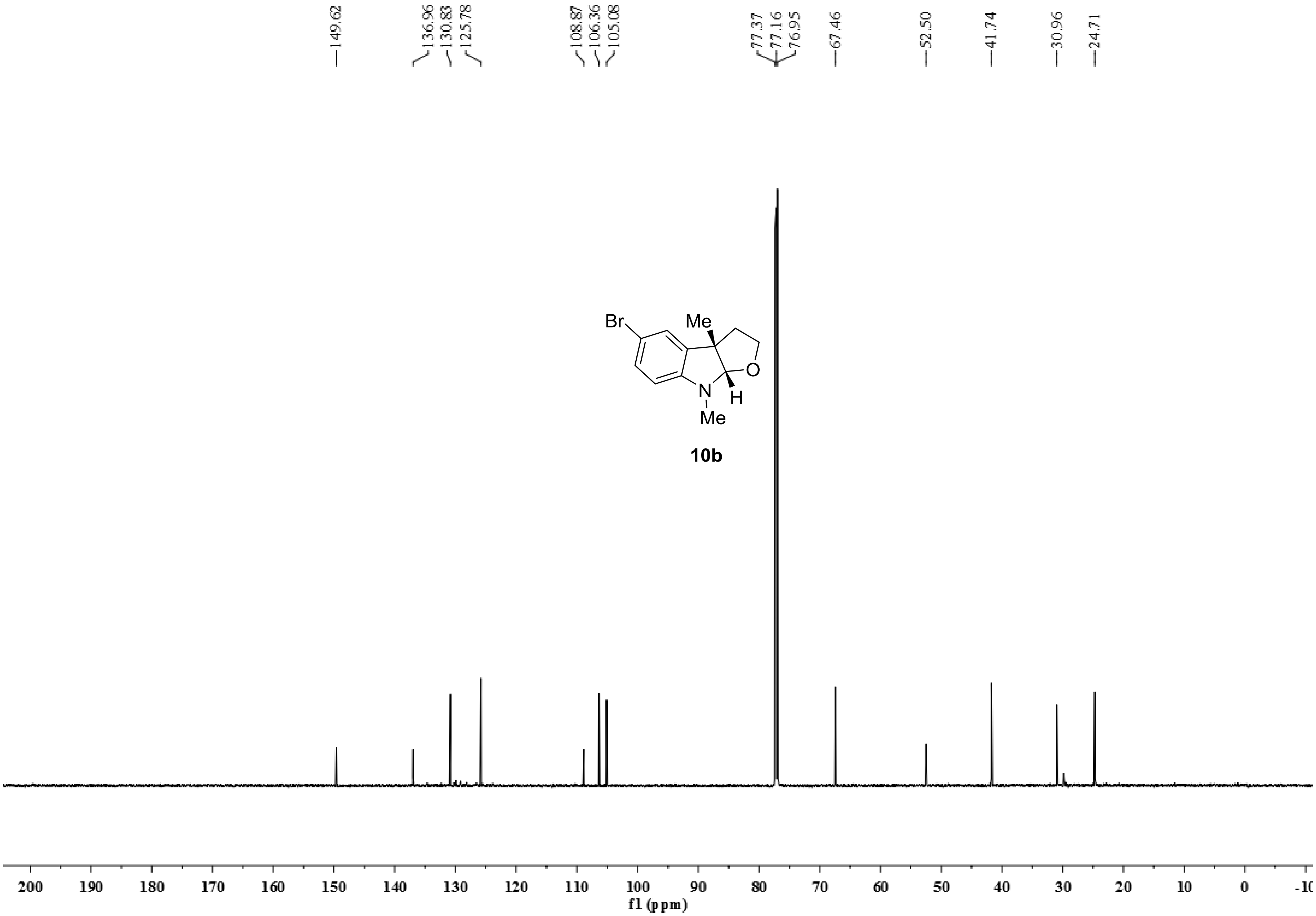

Supplementary Figure 207. ^{13}C-NMR of 10b

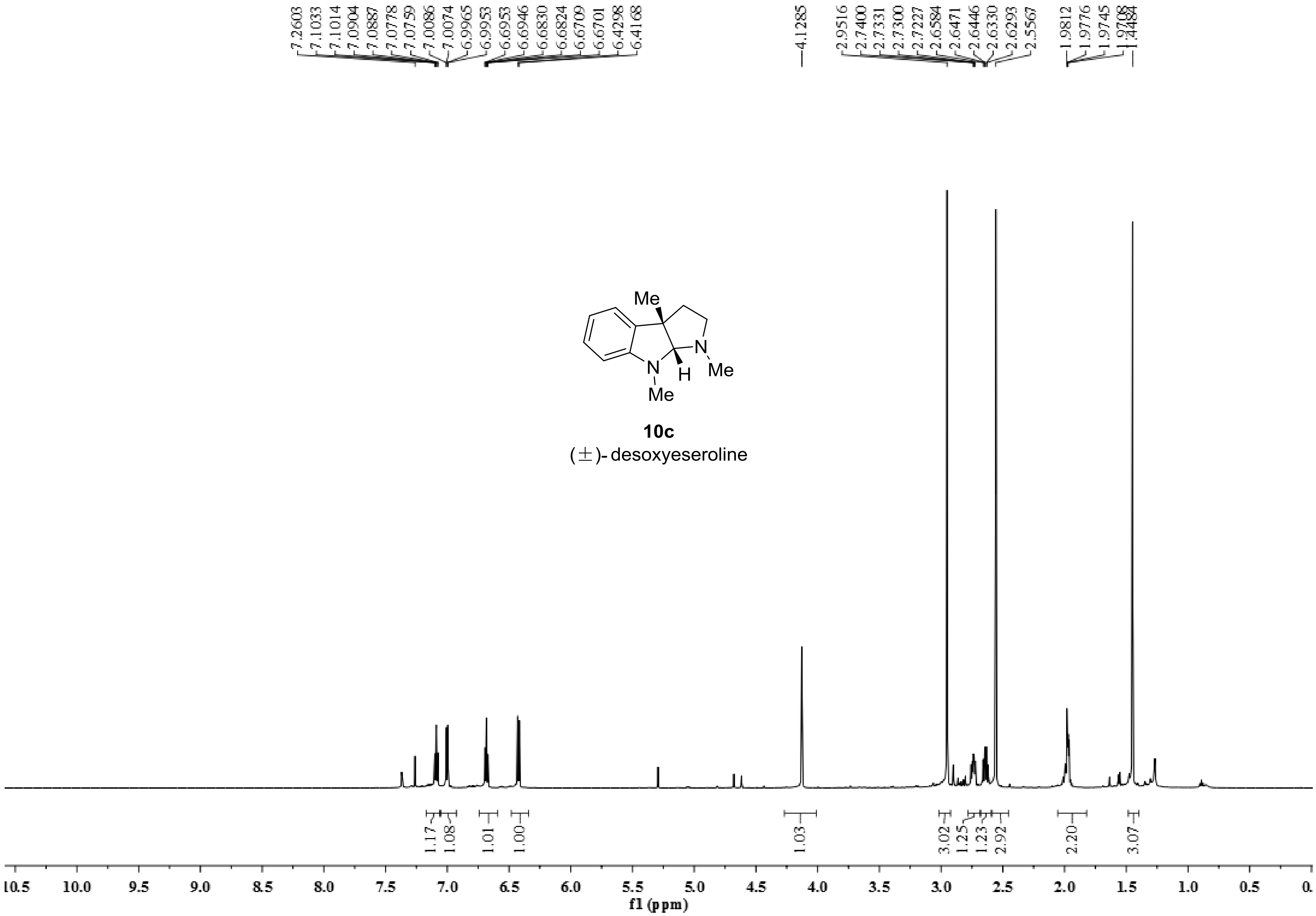

Supplementary Figure 208. ^{1}H-NMR of 10c

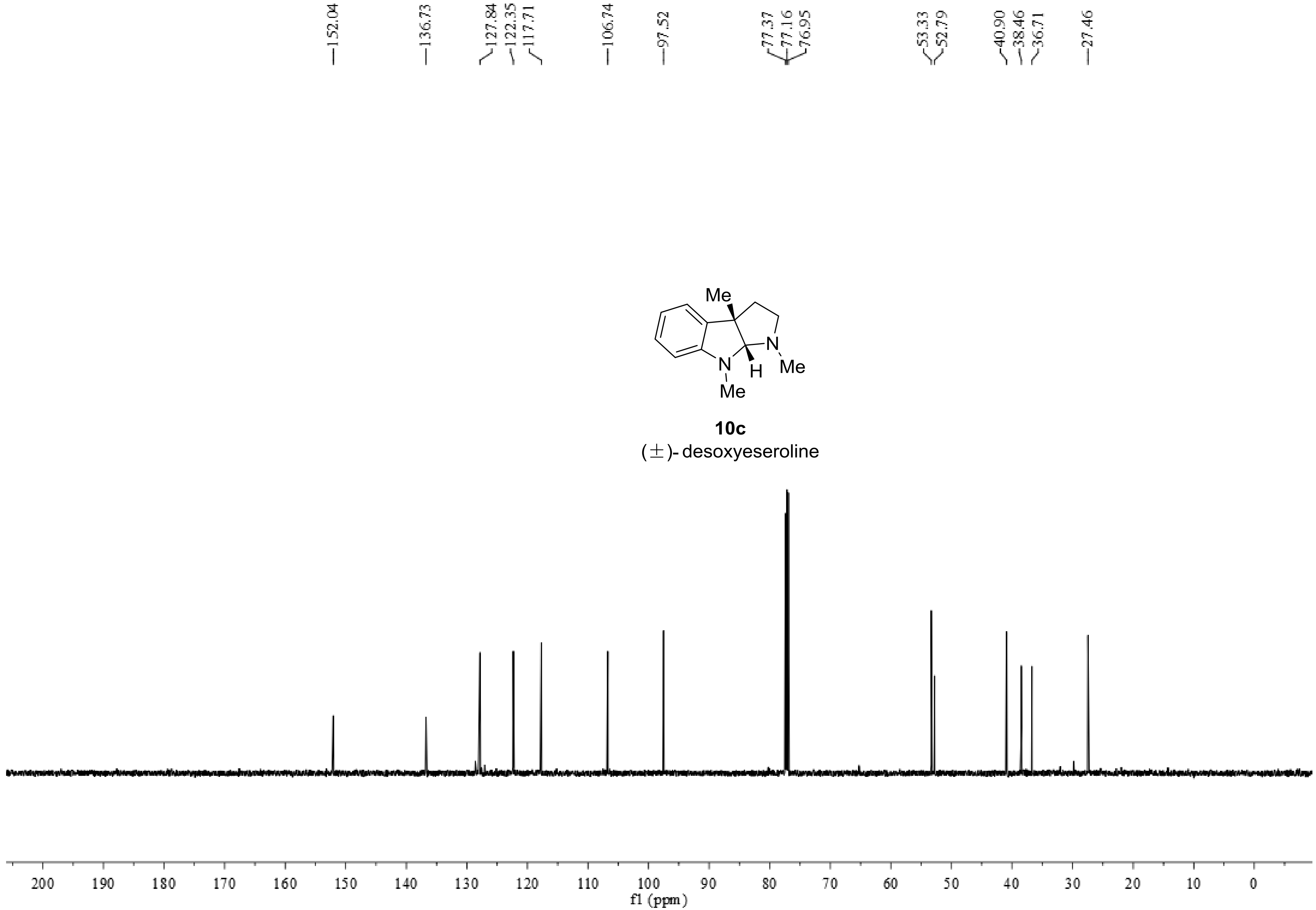

Supplementary Figure 209. ^{13}C-NMR of 10c

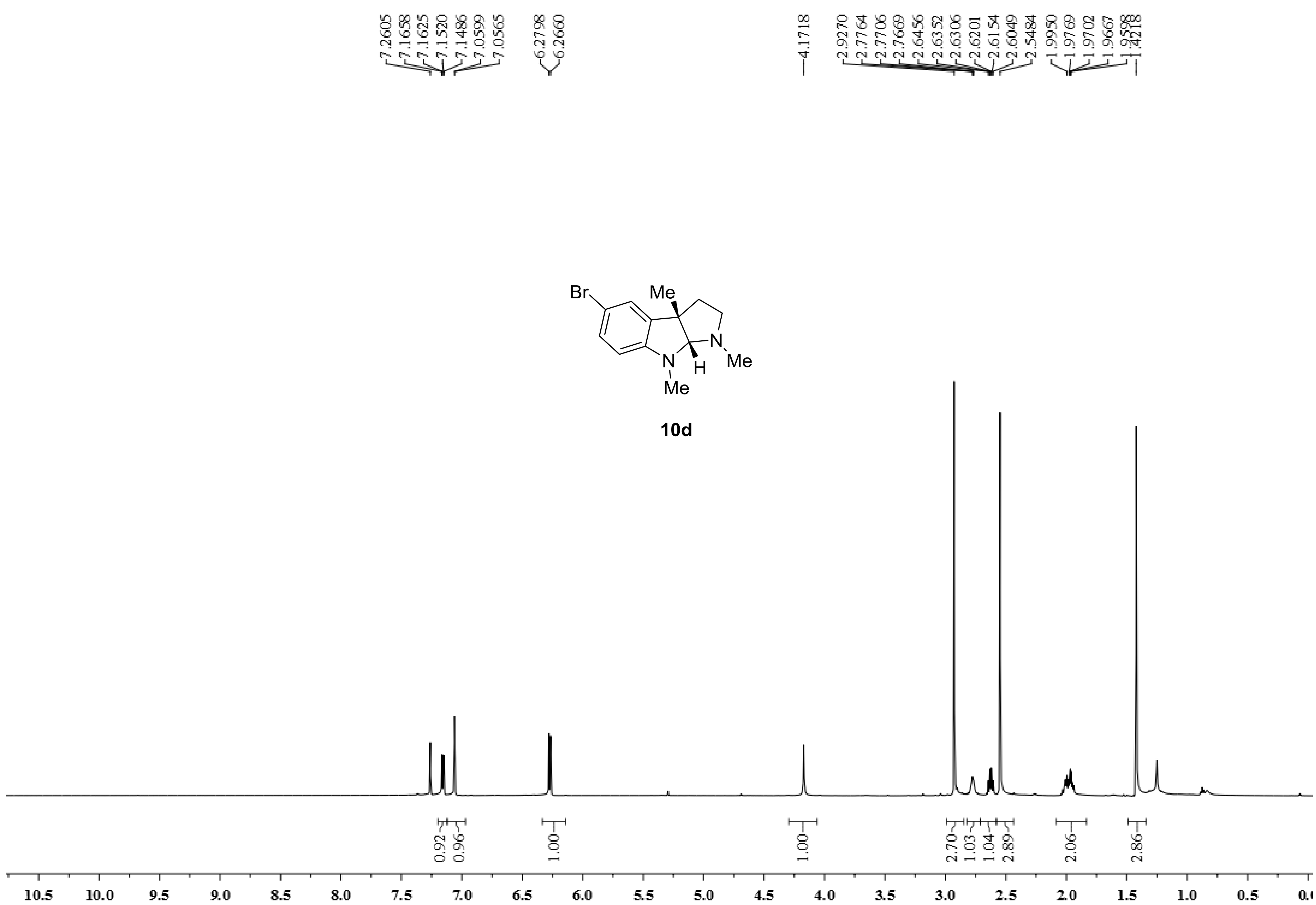

Supplementary Figure 210. ^{1}H-NMR of 10d

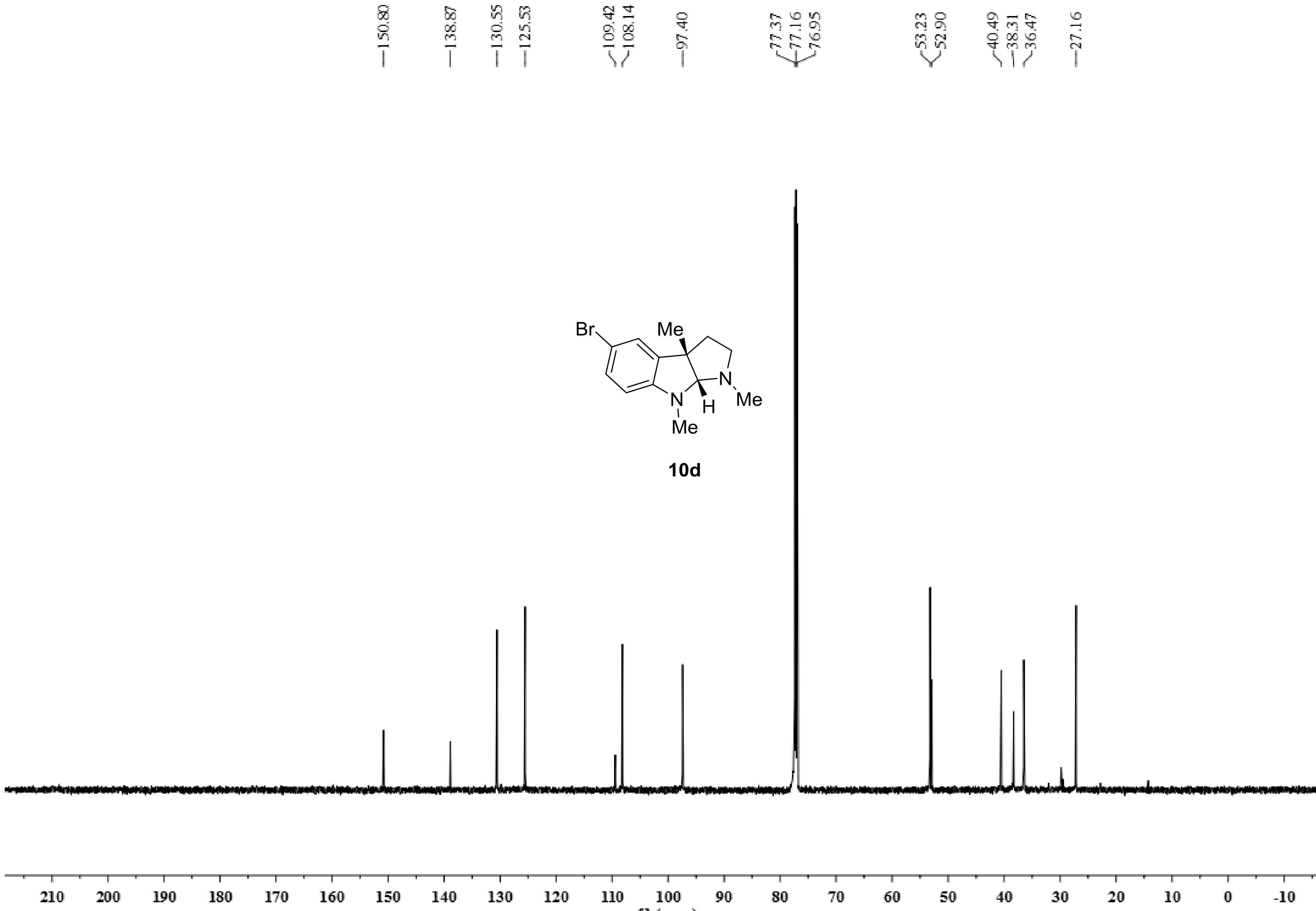

Supplementary Figure 211. ^{13}C-NMR of 10d

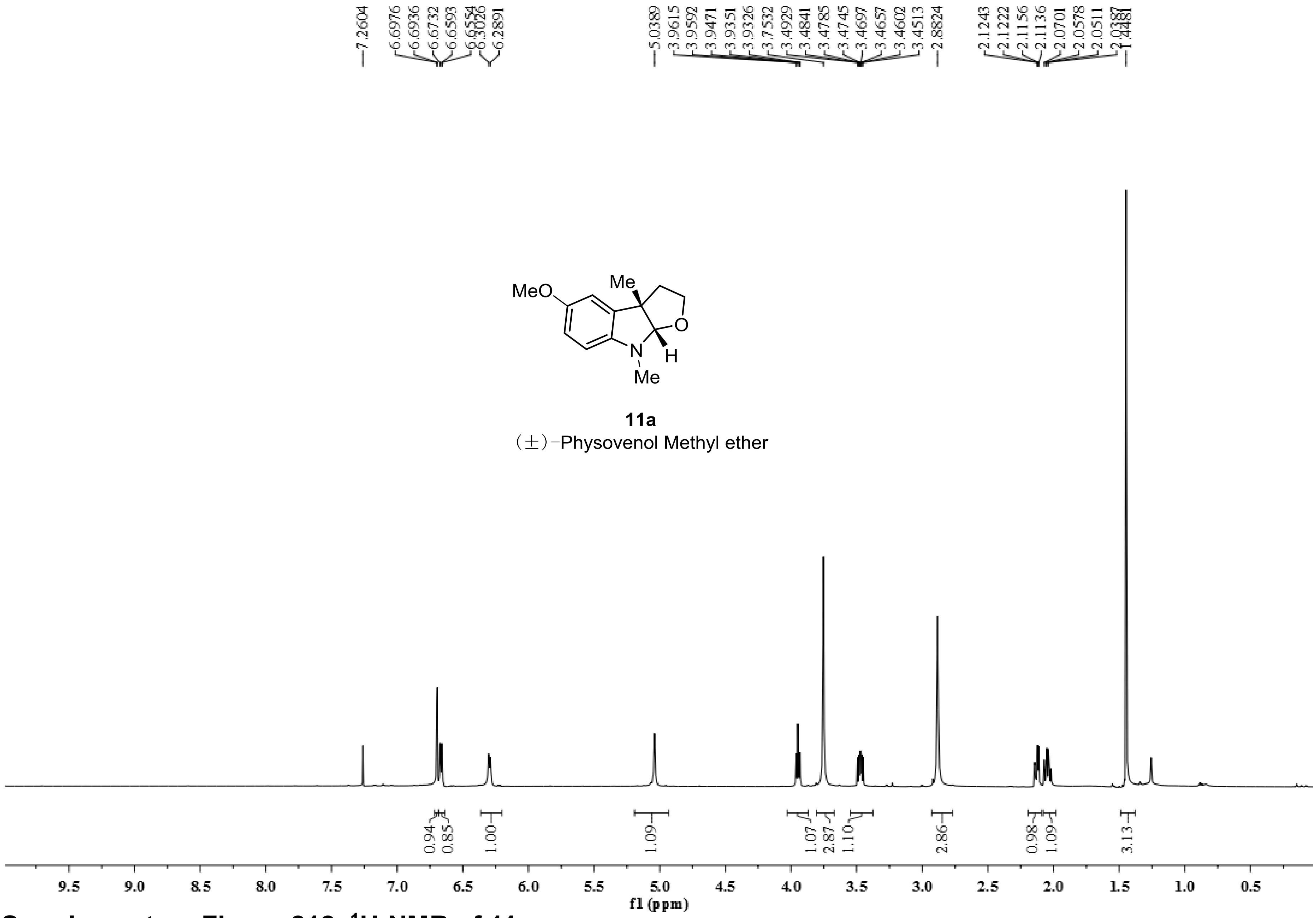

Supplementary Figure 212. ^{1}H-NMR of 11a

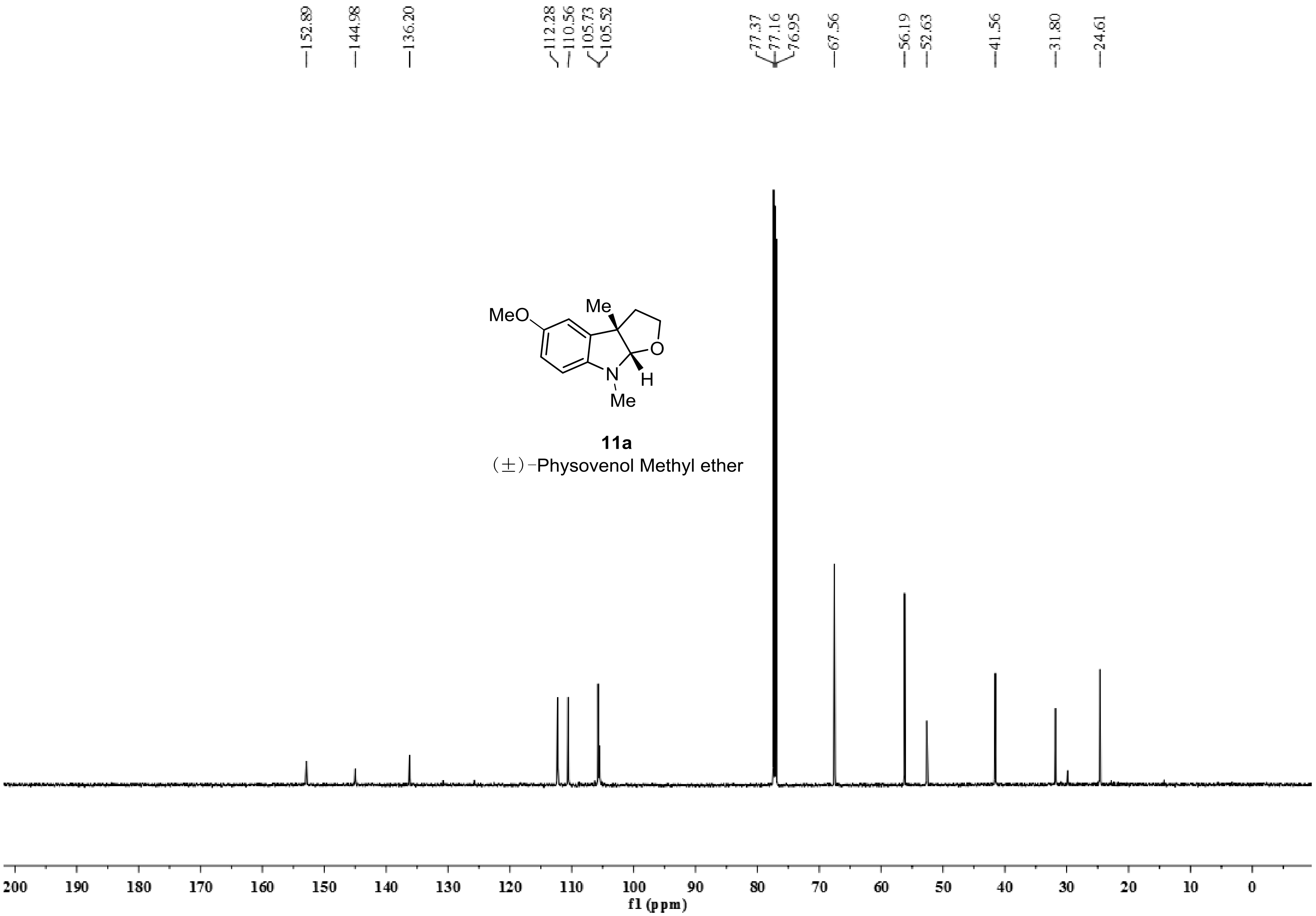

Supplementary Figure 213. ^{13}C-NMR of 11a

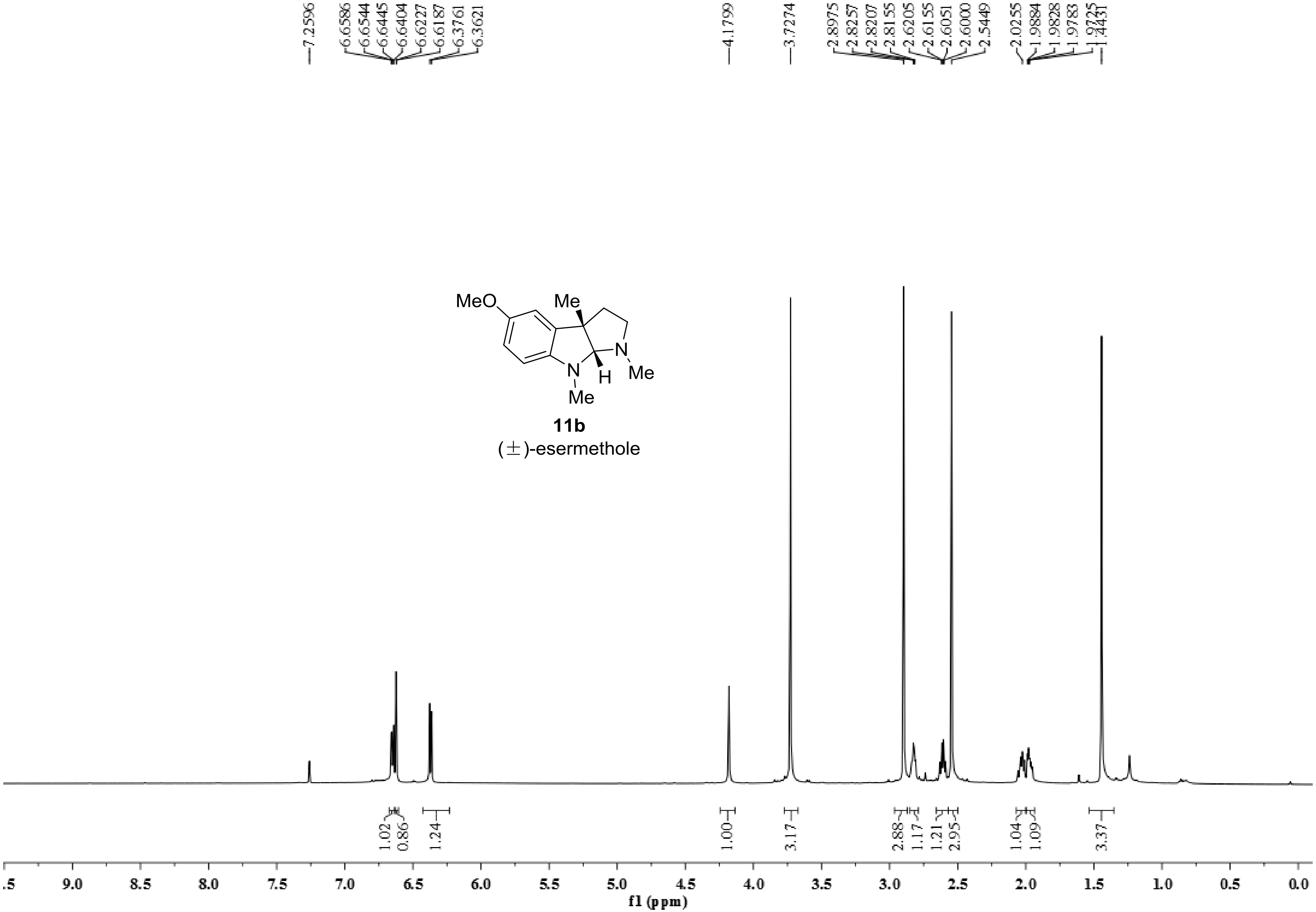

Supplementary Figure 214. ^{1}H-NMR of 11b

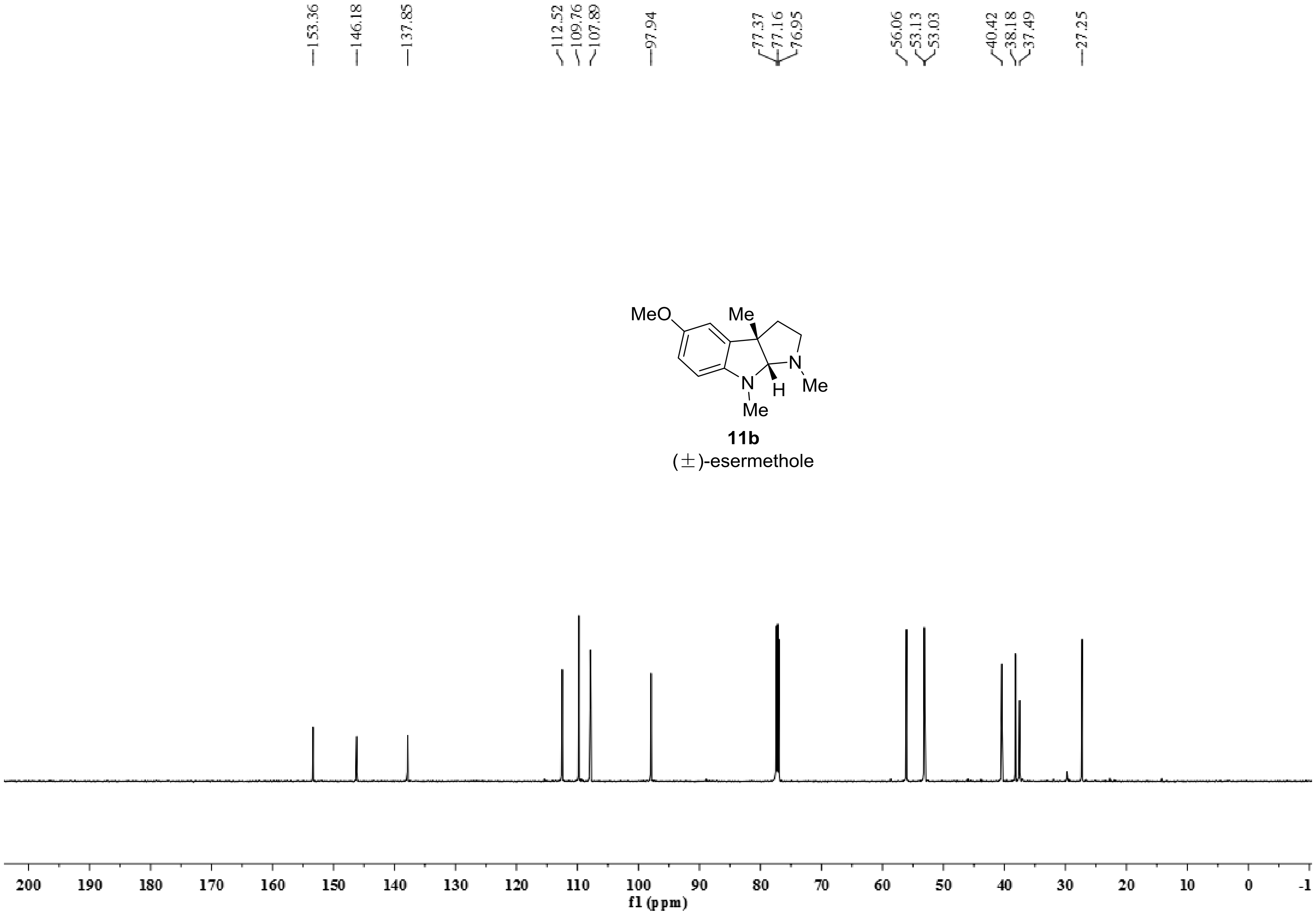

Supplementary Figure 215. ^{13}C-NMR of 11b

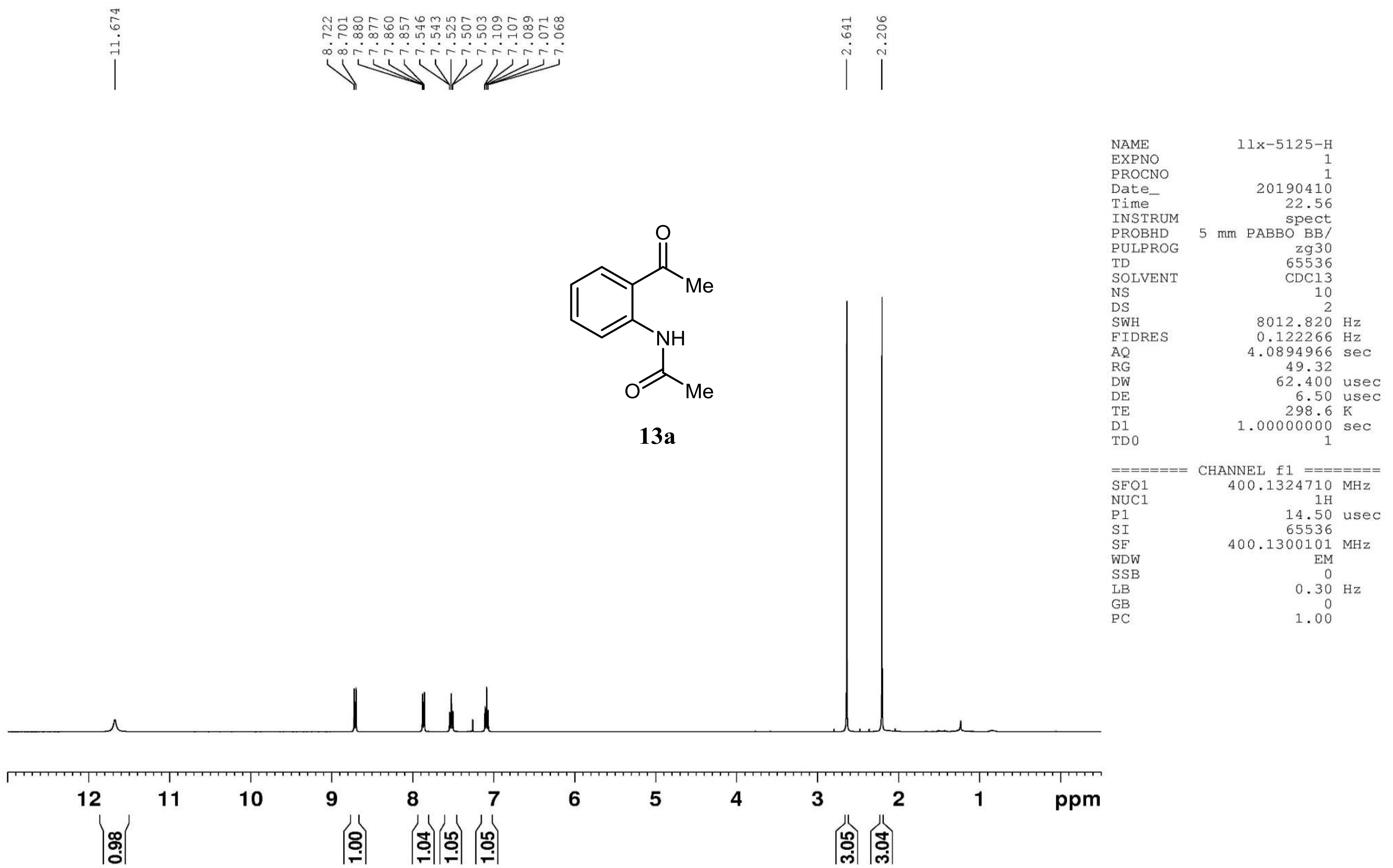

Supplementary Figure 216. ^{1}H-NMR of 13a

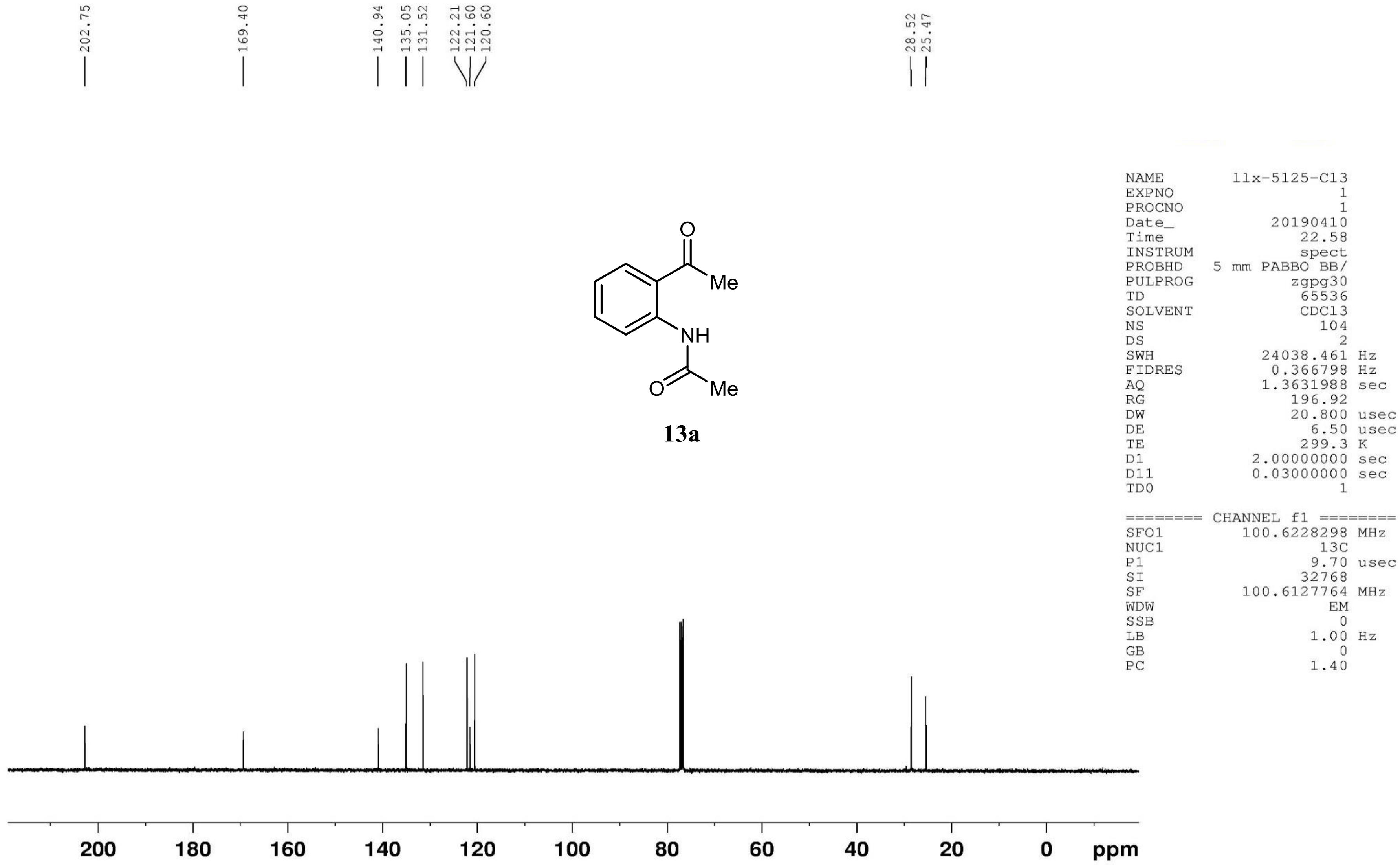

Supplementary Figure 217. ^{13}C-NMR of 13a

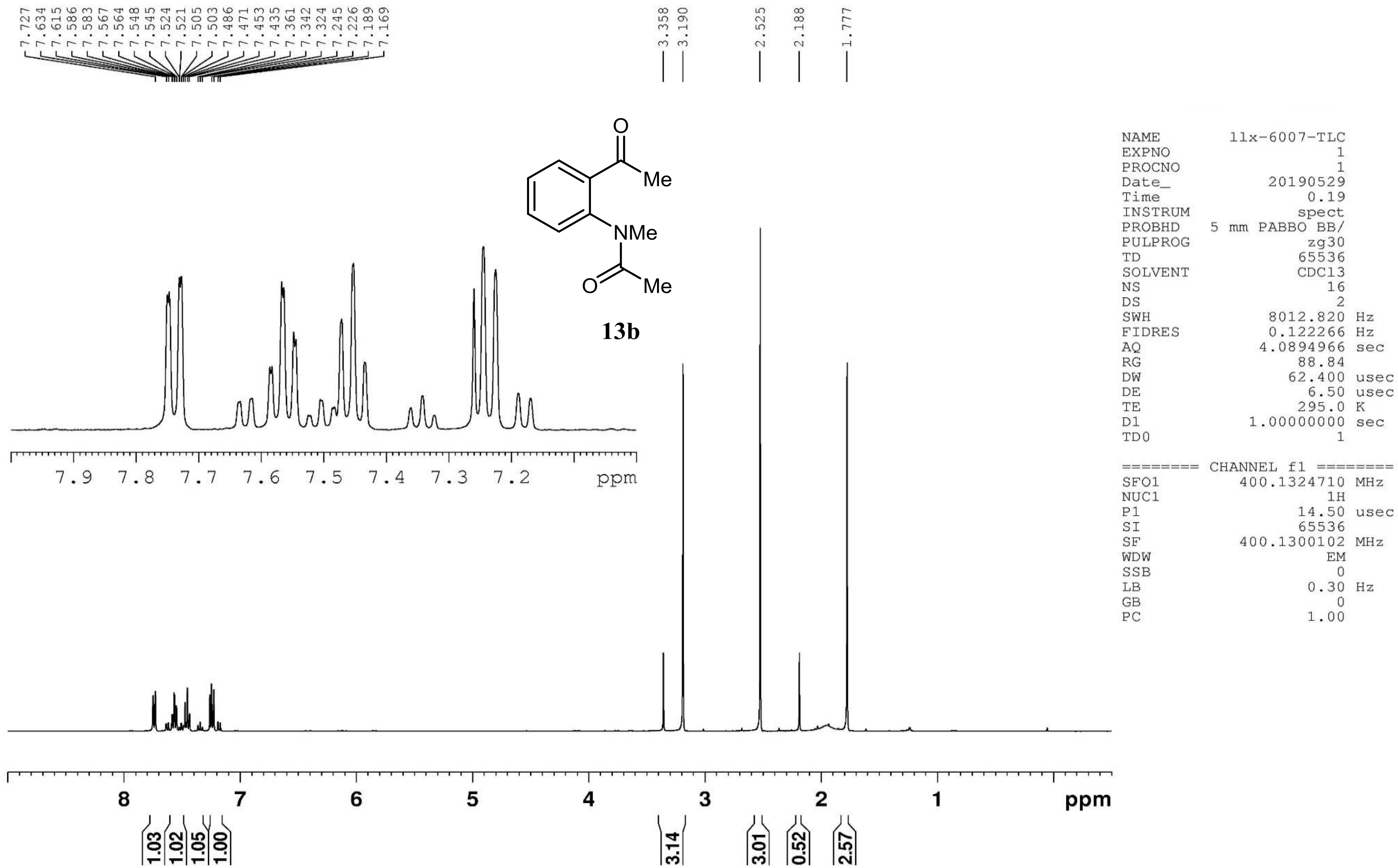

Supplementary Figure 218. ^{1}H-NMR of 13b

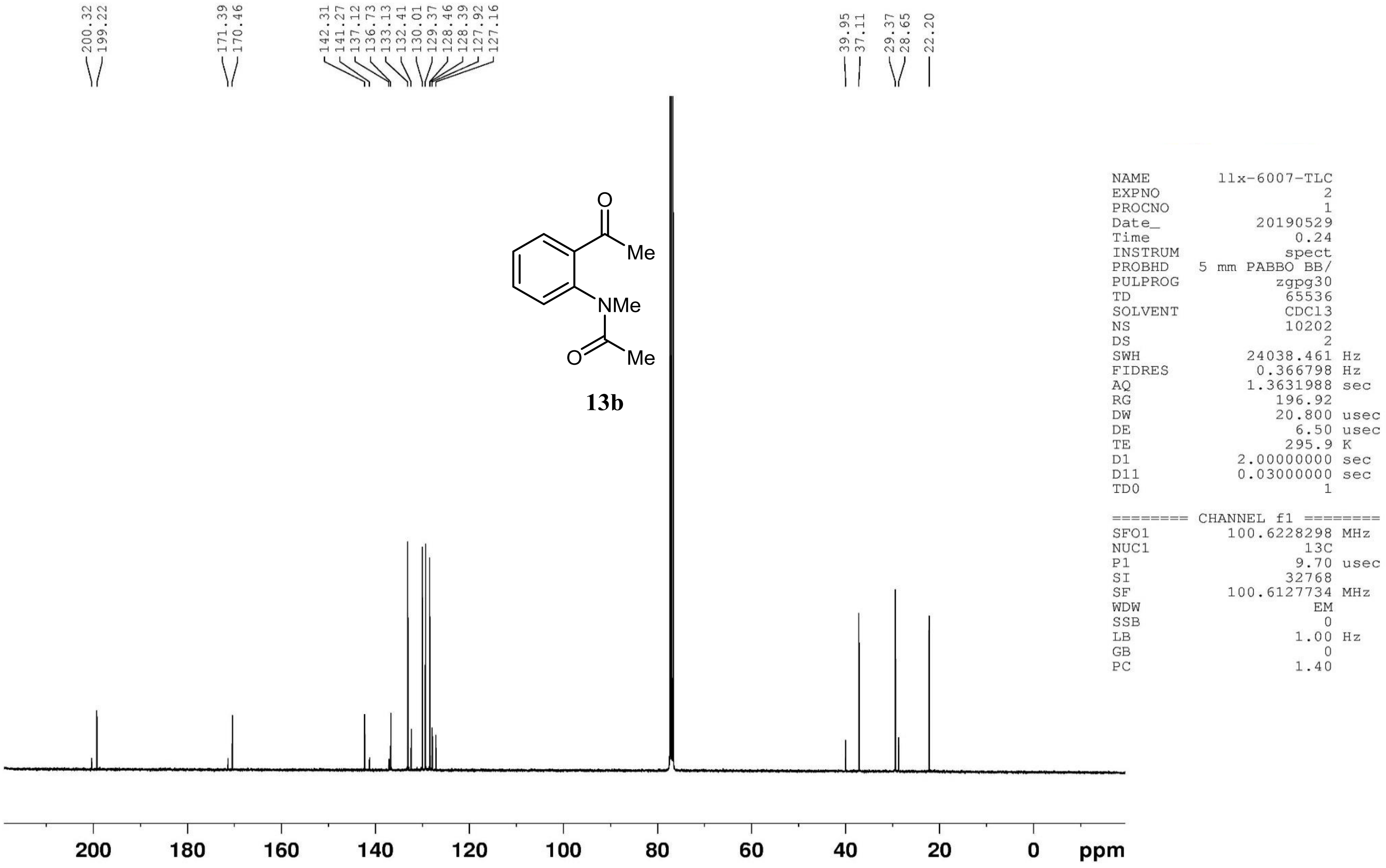

Supplementary Figure 219. ^{13}C-NMR of 13b

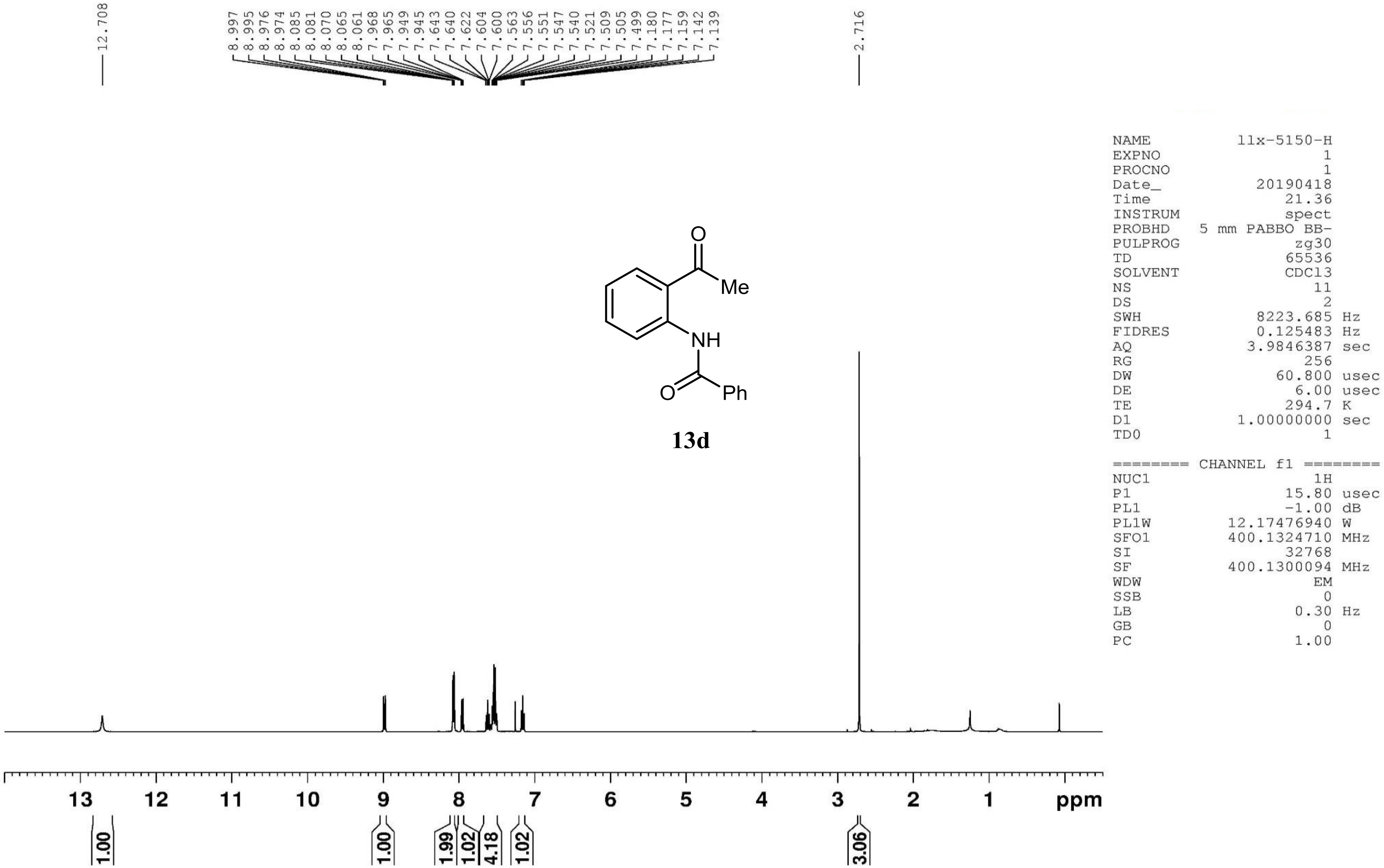

Supplementary Figure 220. ^{1}H-NMR of 13d

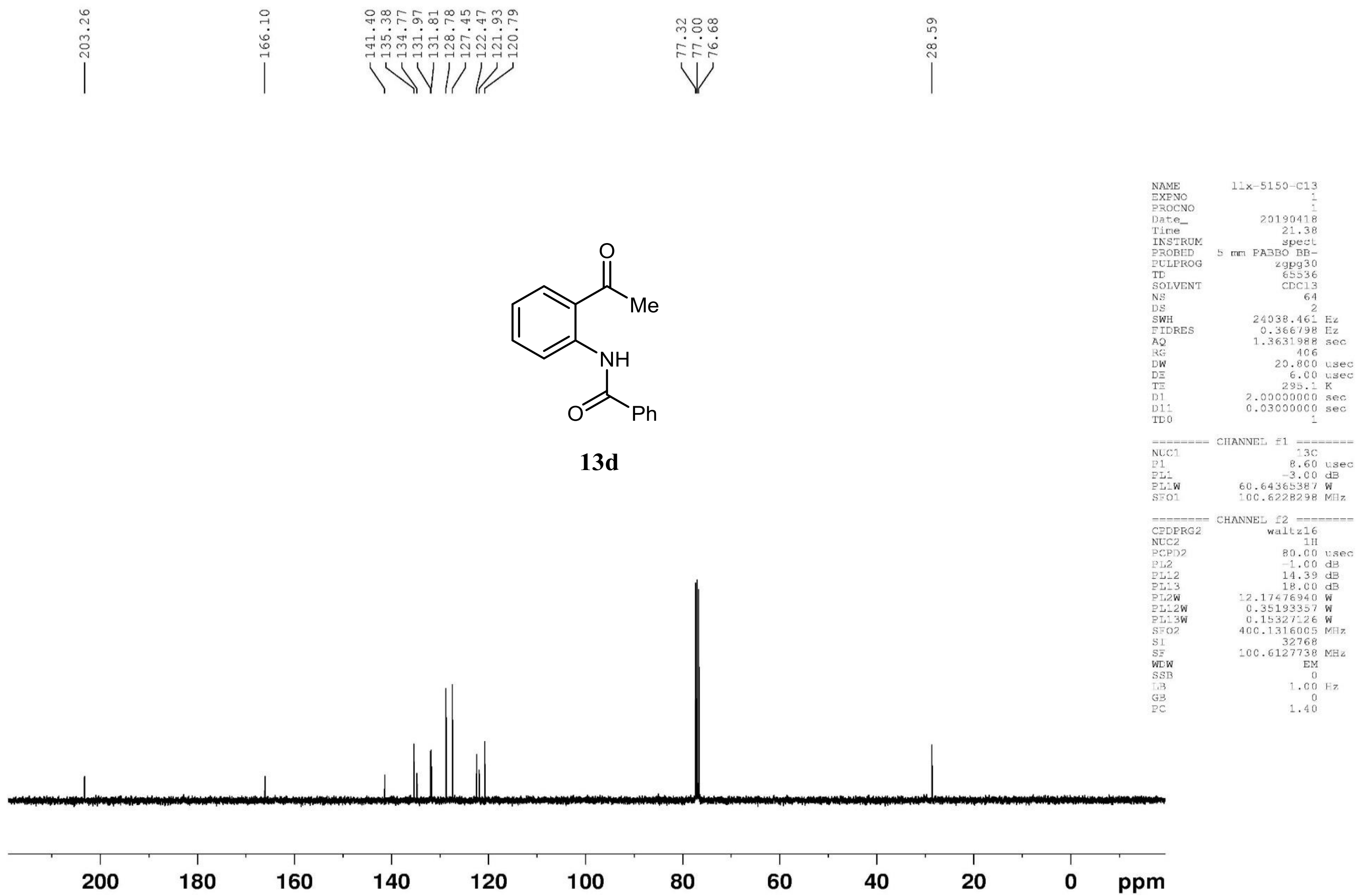

Supplementary Figure 221. ^{13}C-NMR of 13d

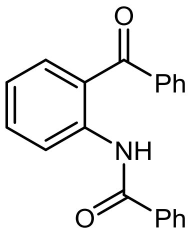

13e

NAME	llx-5154-H
EXPNO	4
PROCNO	1
Date_	20190418
Time	23.21
INSTRUM	spect
PROBHD	5 mm PABBO BB/
PULPROG	zg30
TD	65536
SOLVENT	CDCl3
NS	16
DS	2
SWH	8012.820 Hz
FIDRES	0.122266 Hz
AQ	4.0894966 sec
RG	112.31
DW	62.400 usec
DE	6.50 usec
TE	298.1 K
D1	1.00000000 sec
TD0	1
======== CHANNEL f1 ========	
SFO1	400.1324710 MHz
NUC1	1H
P1	14.50 usec
SI	65536
SF	400.1300102 MHz
WDW	EM
SSB	0
LB	0.30 Hz
GB	0
PC	1.00

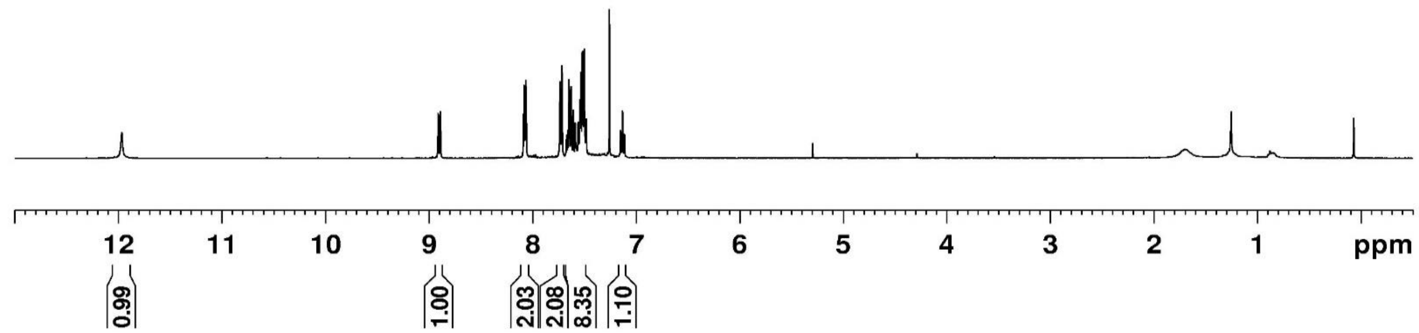

Supplementary Figure 222. ^{1}H-NMR of 13e

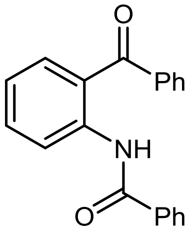

13e

NAME	llx-5154-C13
EXPNO	1
PROCNO	1
Date_	20190418
Time	22.12
INSTRUM	spect
PROBHD	5 mm PABBO BB/
PULPROG	zgpg30
TD	65536
SOLVENT	CDCl3
NS	101
DS	2
SWH	24038.461 Hz
FIDRES	0.366798 Hz
AQ	1.3631988 sec
RG	196.92
DW	20.800 usec
DE	6.50 usec
TE	298.3 K
D1	2.00000000 sec
D11	0.03000000 sec
TD0	1
======== CHANNEL f1 ========	
SFO1	100.6228298 MHz
NUC1	13C
P1	9.70 usec
SI	32768
SF	100.6127740 MHz
WDW	EM
SSB	0
LB	1.00 Hz
GB	0
PC	1.40

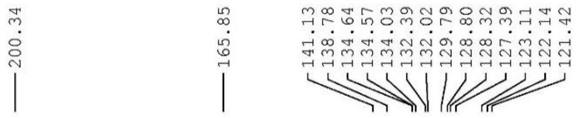
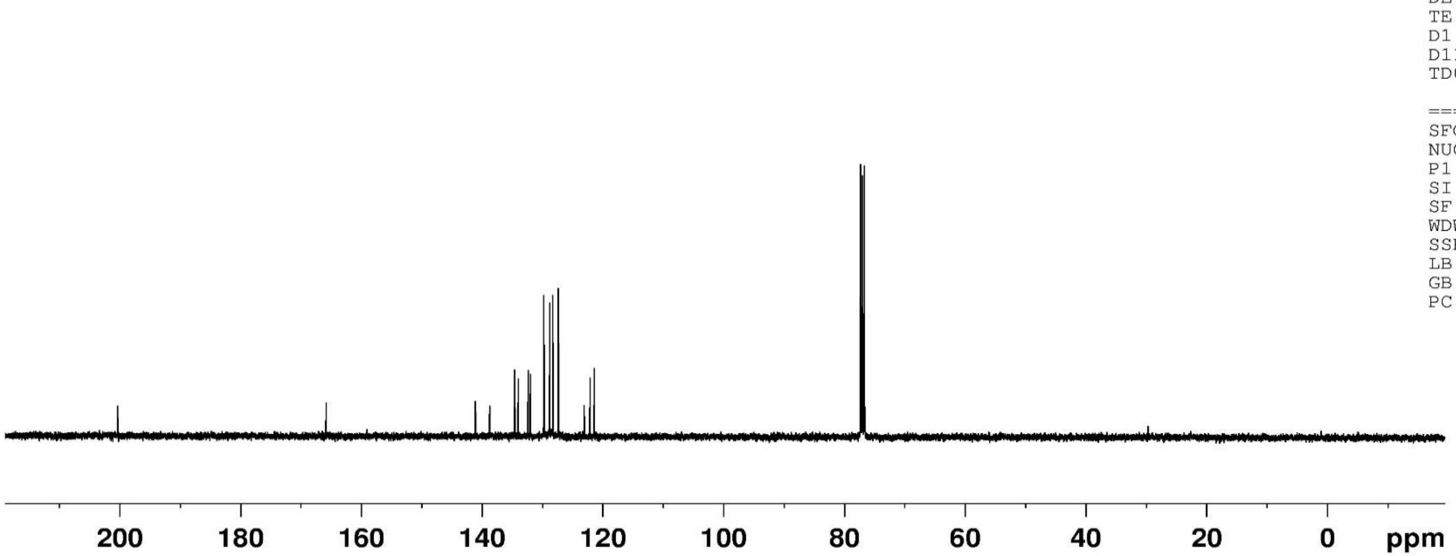

Supplementary Figure 223. ^{13}C-NMR of 13e

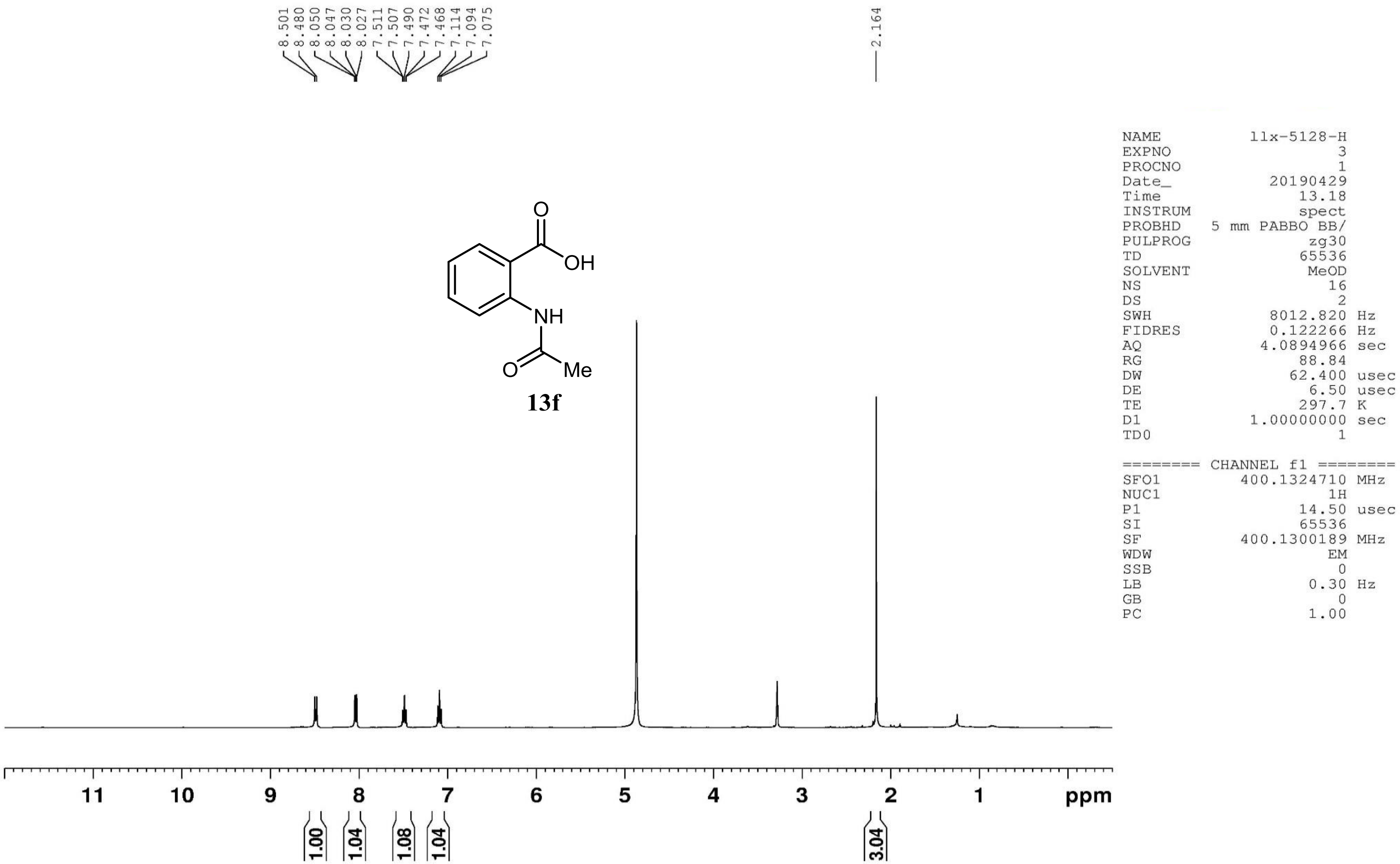

Supplementary Figure 224. ^{1}H-NMR of 13f

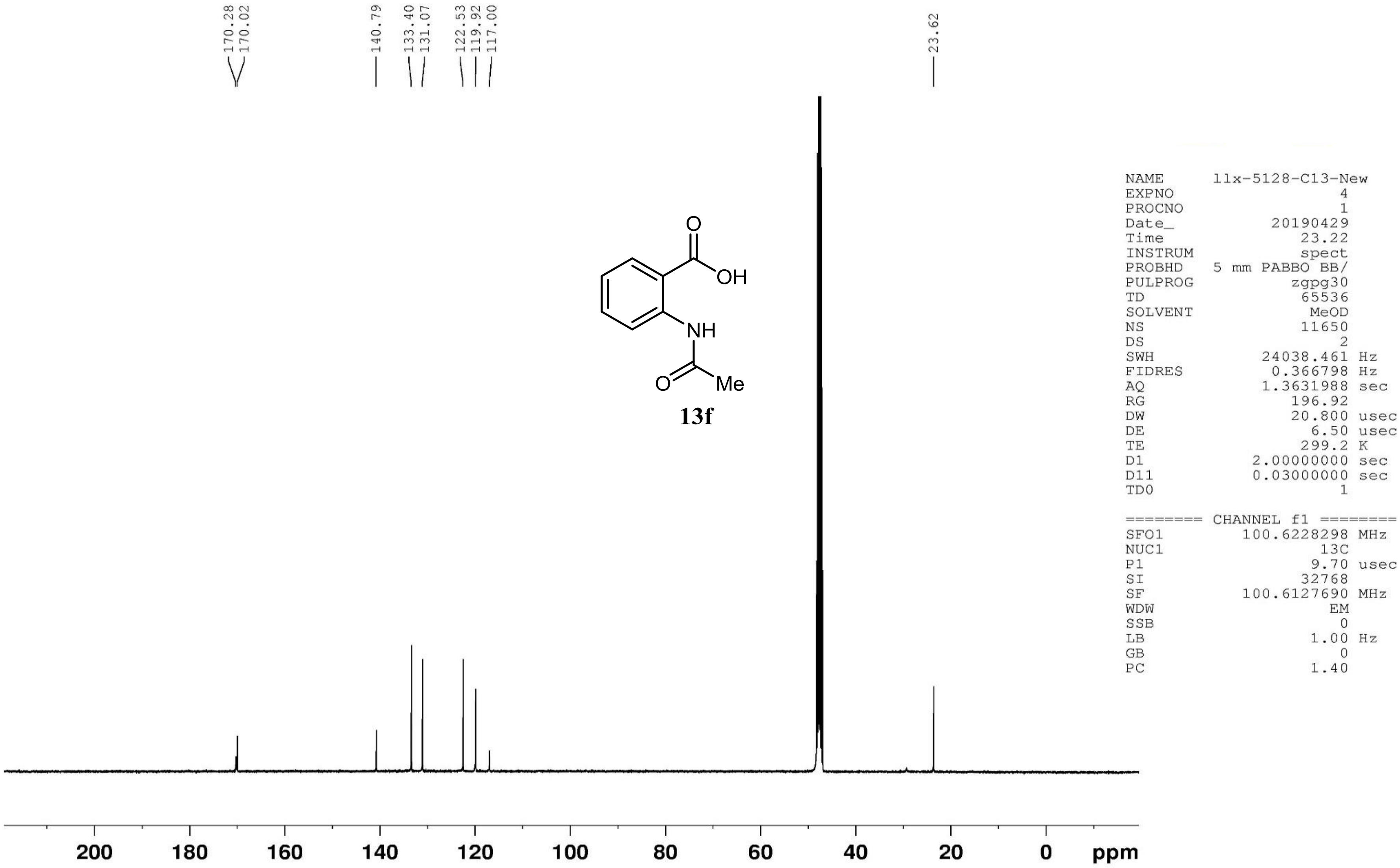

Supplementary Figure 225. ^{13}C-NMR of 13f

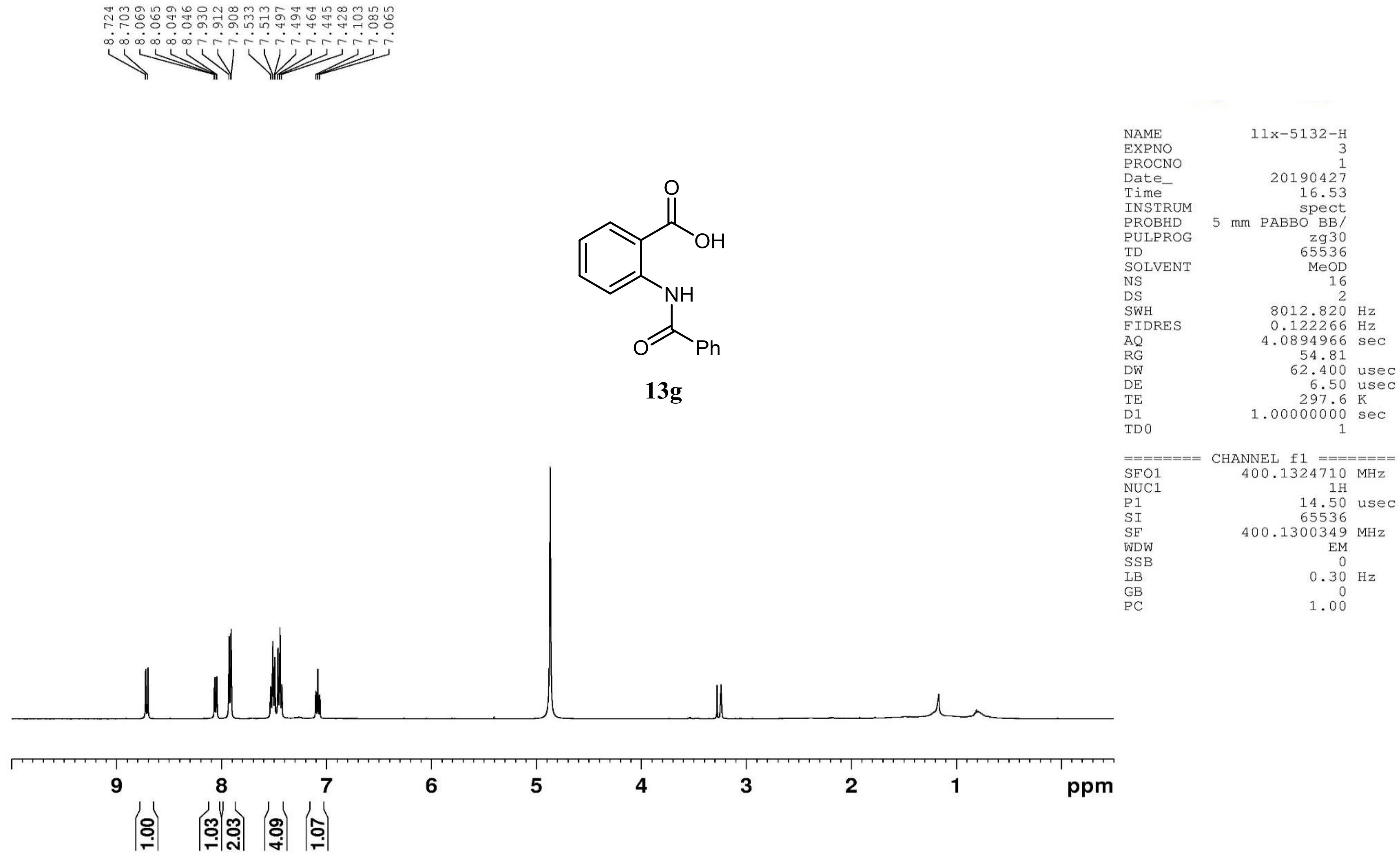

Supplementary Figure 226. ^{1}H-NMR of 13g

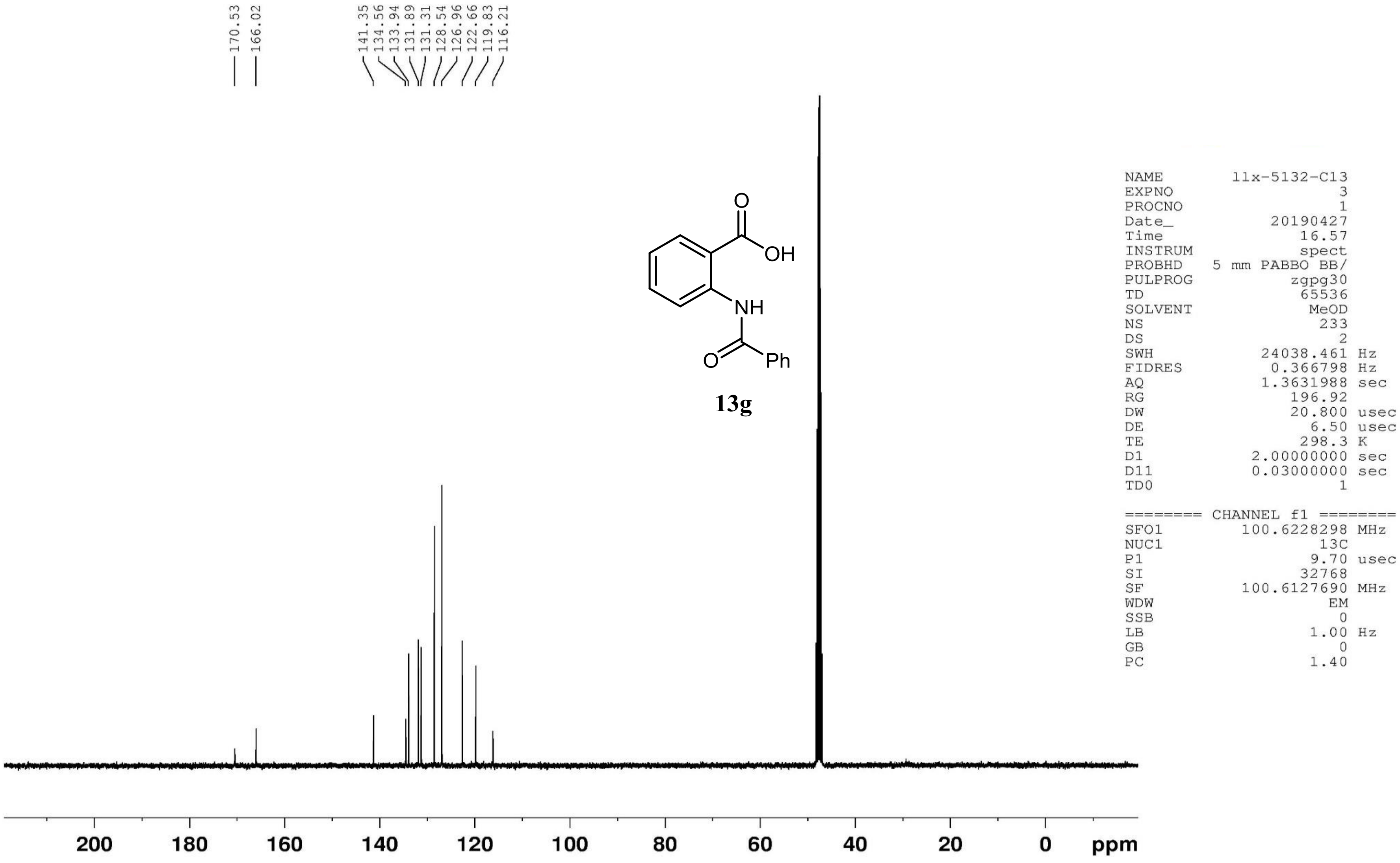

Supplementary Figure 227. ^{13}C-NMR of 13g

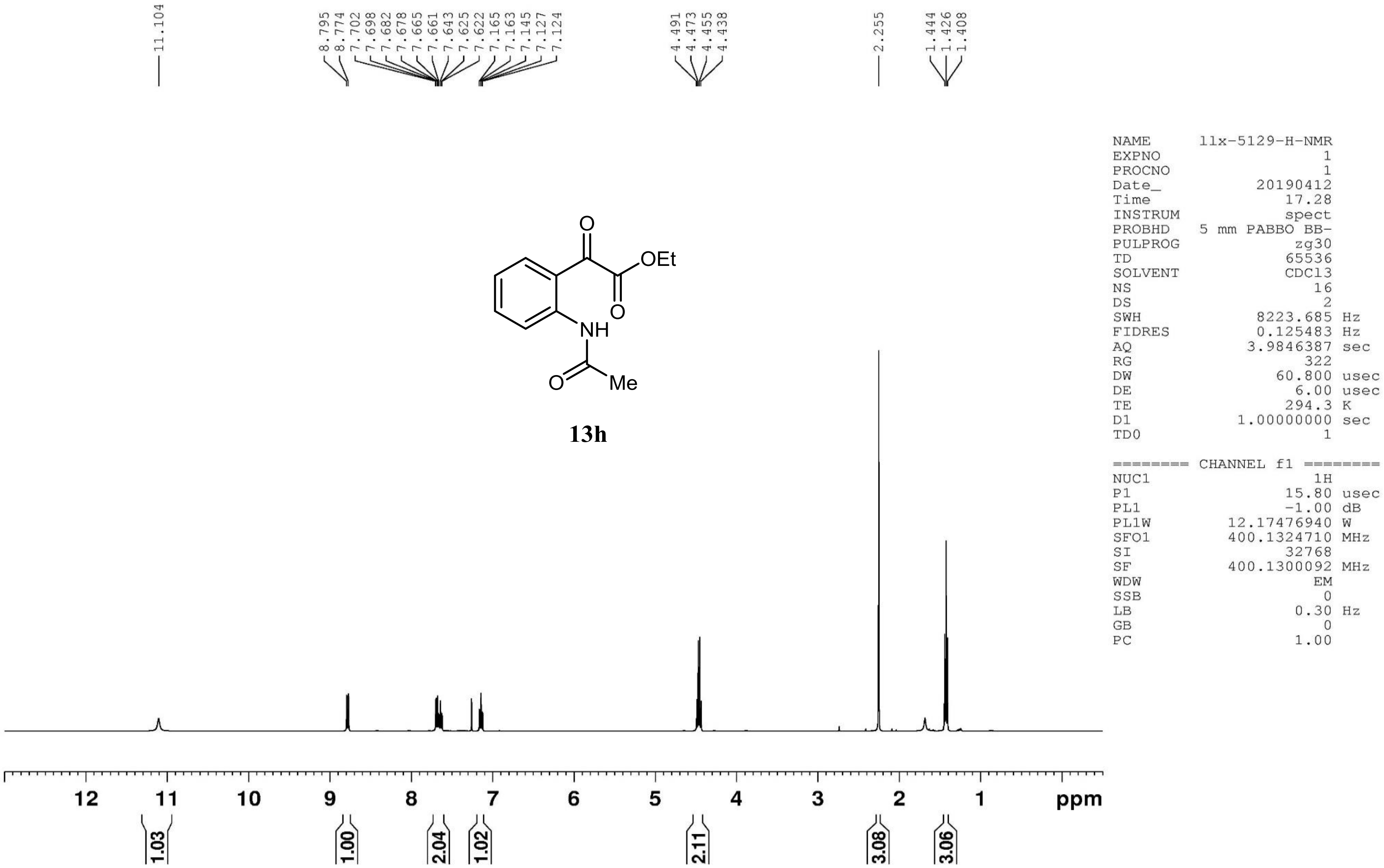

Supplementary Figure 228. ^{1}H-NMR of 13h

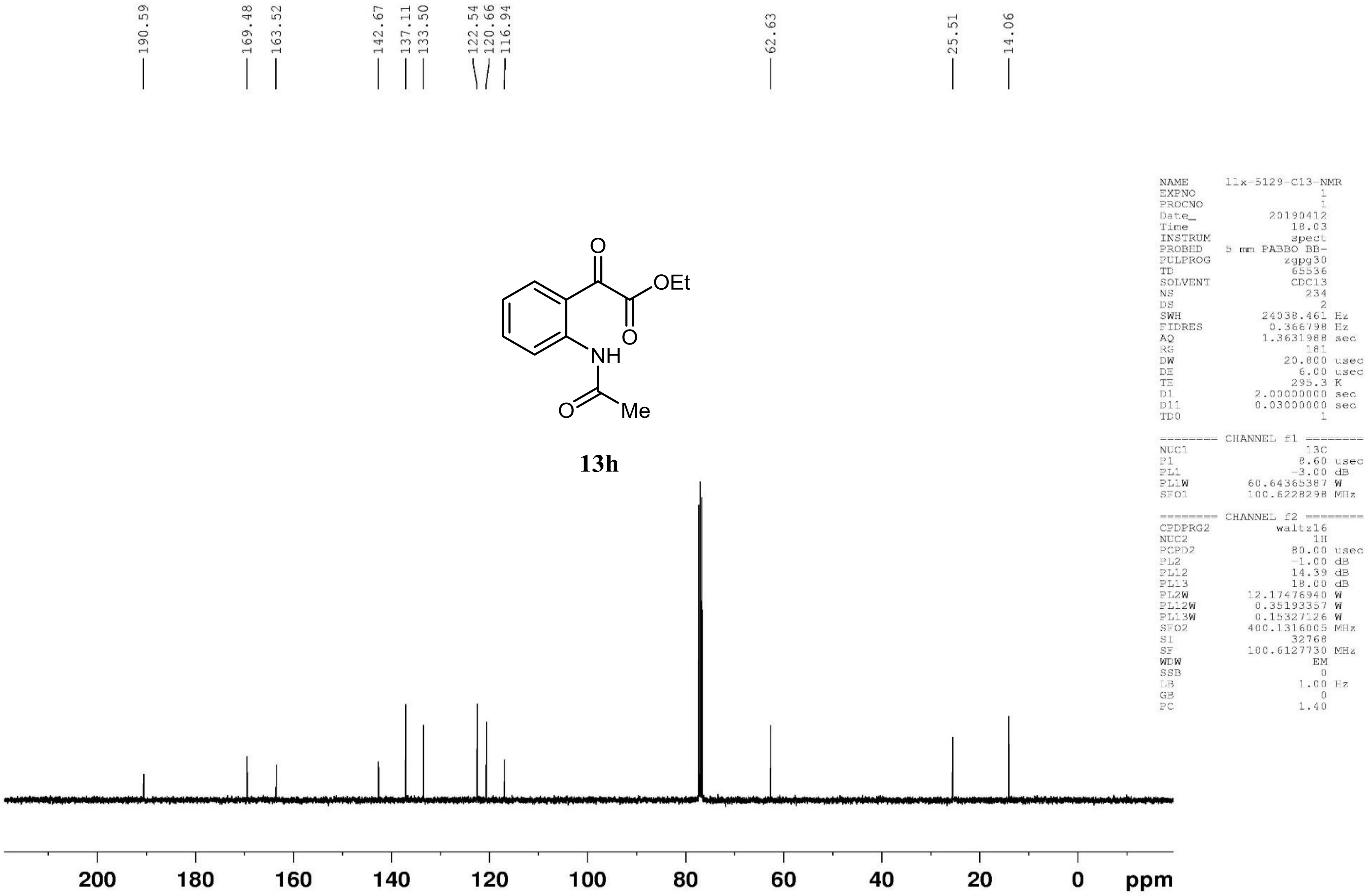

Supplementary Figure 229. ^{13}C-NMR of 13h

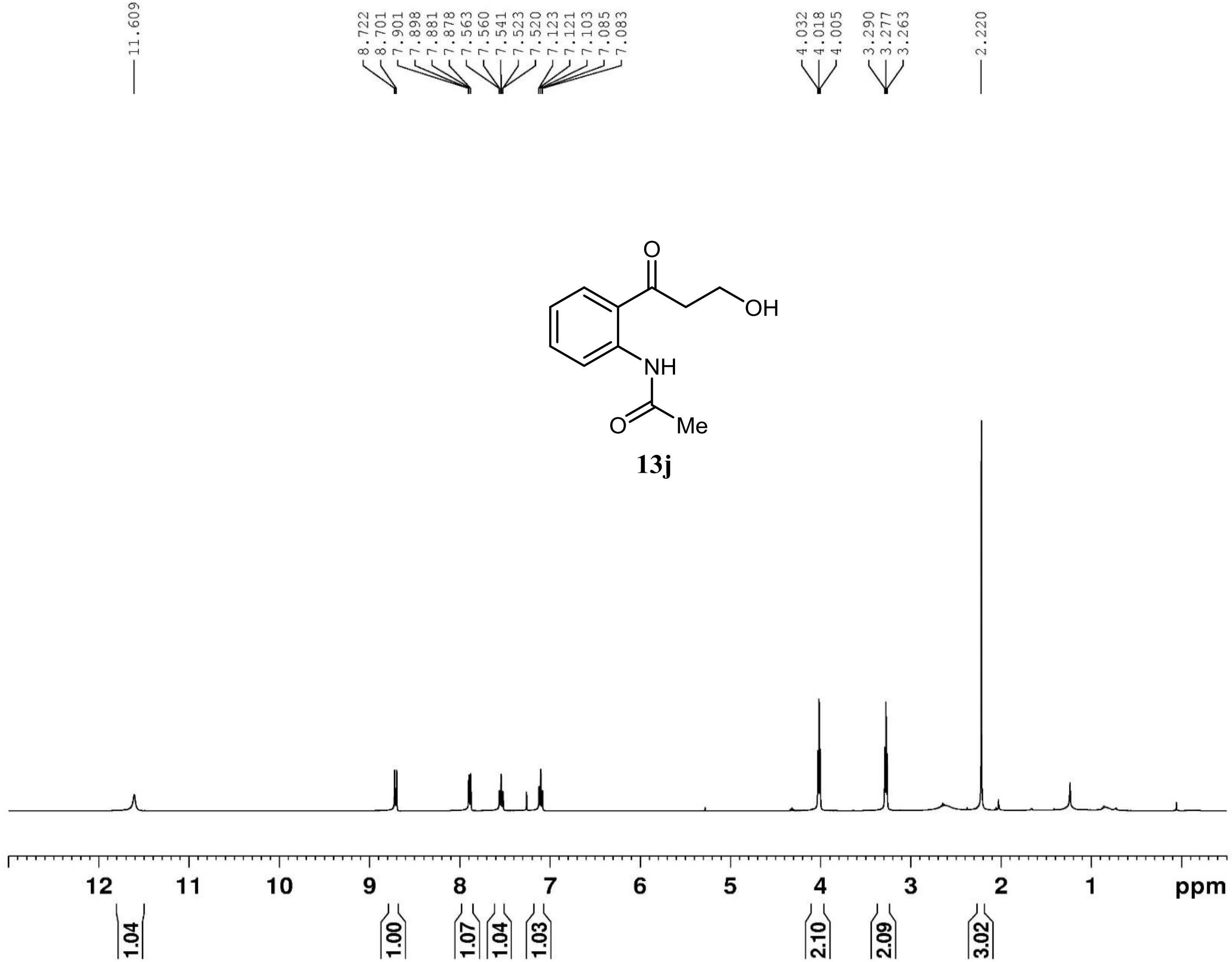

13j

NAME	llx-5149-H
EXPNO	1
PROCNO	1
Date_	20190418
Time	13.39
INSTRUM	spect
PROBHD	5 mm PABBO BB-
PULPROG	zg30
TD	65536
SOLVENT	CDCl3
NS	16
DS	2
SWH	8223.685 Hz
FIDRES	0.125483 Hz
AQ	3.9846387 sec
RG	181
DW	60.800 usec
DE	6.00 usec
TE	294.5 K
D1	1.00000000 sec
TD0	1
======== CHANNEL f1 ========	
NUC1	1H
P1	15.80 usec
PL1	-1.00 dB
PL1W	12.17476940 W
SFO1	400.1324710 MHz
SI	32768
SF	400.1300090 MHz
WDW	EM
SSB	0
LB	0.30 Hz
GB	0
PC	1.00

Supplementary Figure 230. ^{1}H-NMR of 13j

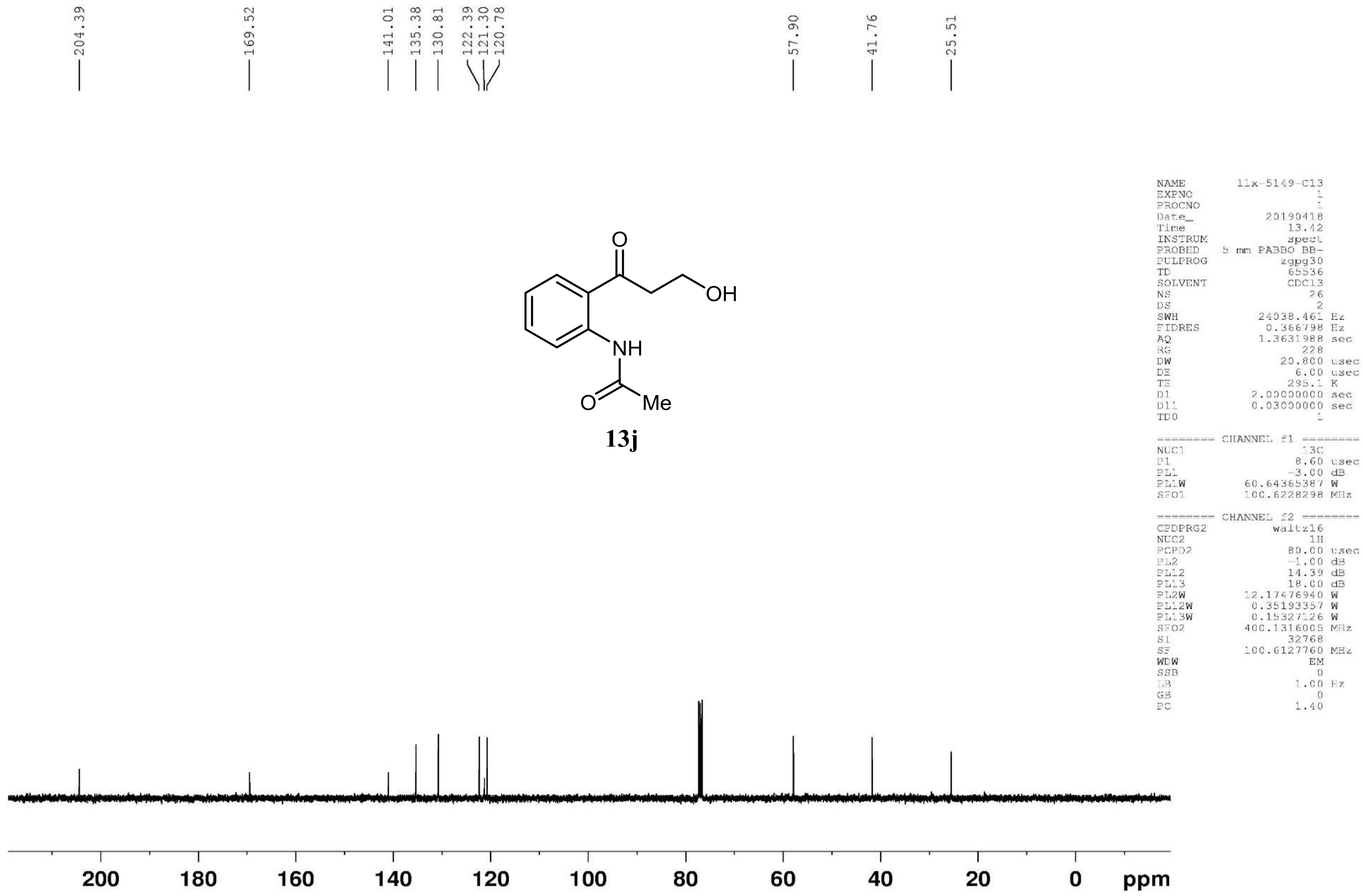

Supplementary Figure 231. ^{13}**C-NMR of 13j**

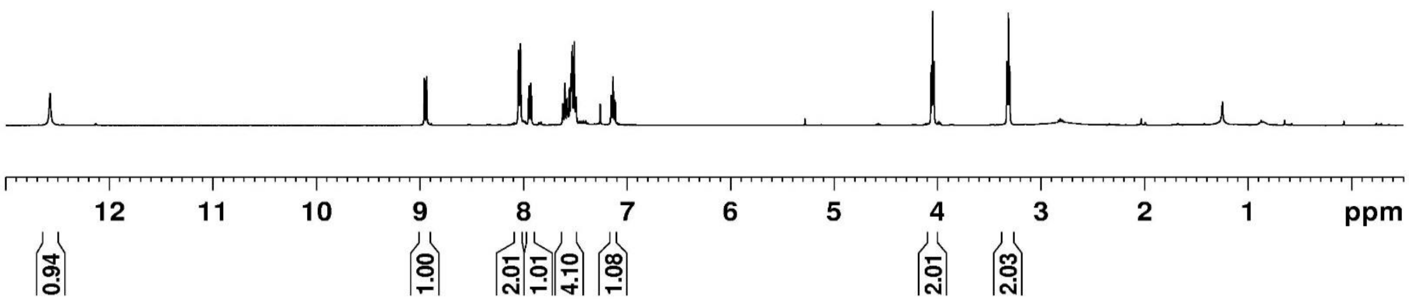

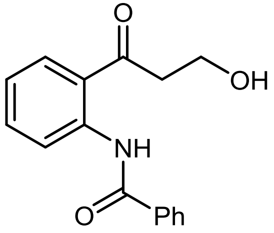

13k

NAME	llx-5151-H	
EXPNO	1	
PROCNO	1	
Date_	20190418	
Time	13.22	
INSTRUM	spect	
PROBHD	5 mm PABBO BB-	
PULPROG	zg30	
TD	65536	
SOLVENT	CDCl3	
NS	4	
DS	0	
SWH	8223.685	Hz
FIDRES	0.125483	Hz
AQ	3.9846387	sec
RG	181	
DW	60.800	usec
DE	6.00	usec
TE	294.4	K
D1	1.00000000	sec
TD0	1	
======== CHANNEL f1 ========		
NUC1	1H	
P1	15.80	usec
PL1	-1.00	dB
PL1W	12.17476940	W
SFO1	400.1324710	MHz
SI	32768	
SF	400.1300091	MHz
WDW	EM	
SSB	0	
LB	0.30	Hz
GB	0	
PC	1.00	

Supplementary Figure 232. ^{1}H-NMR of 13k

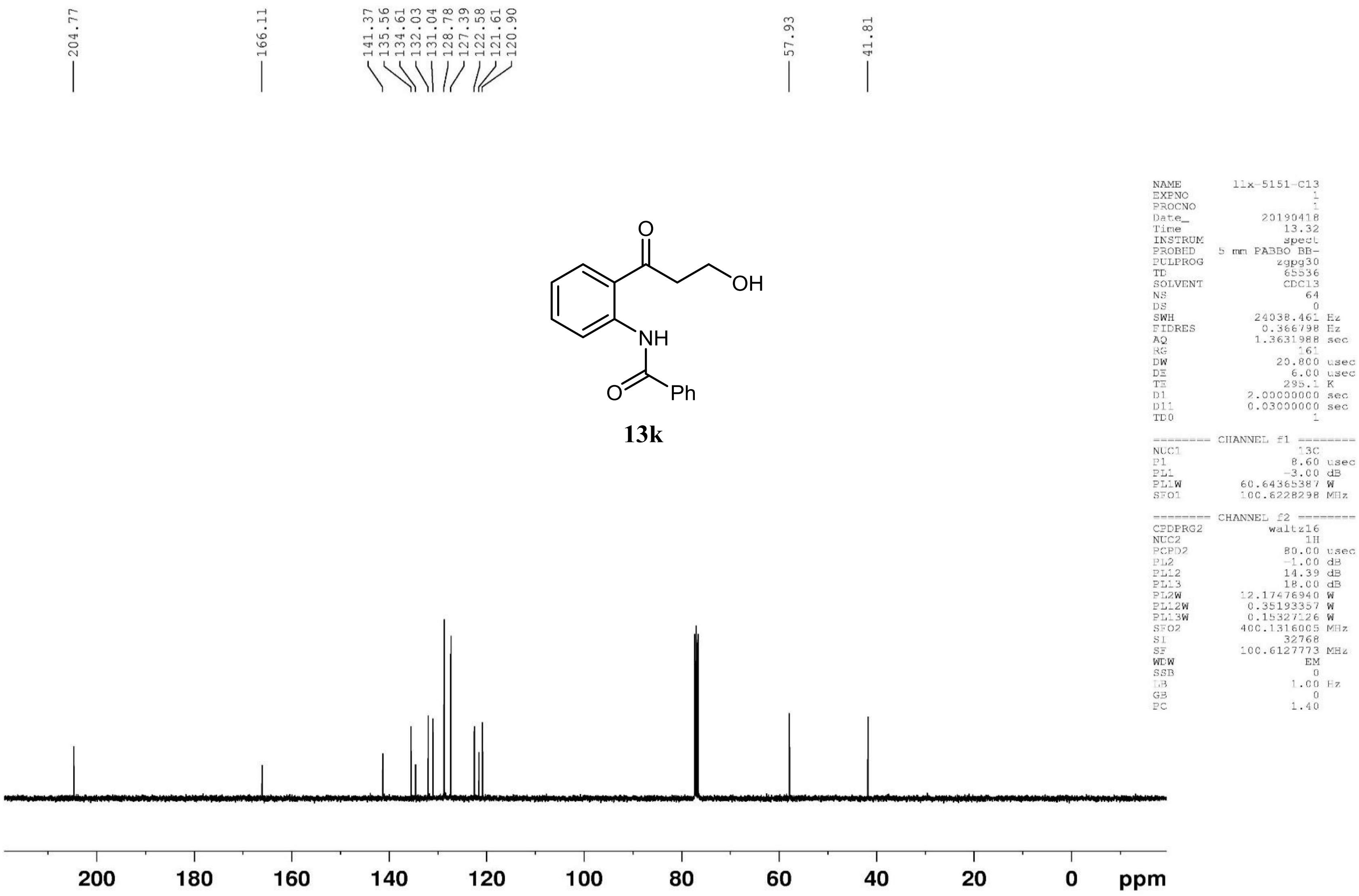

13k

Supplementary Figure 233. ^{13}C-NMR of 13k

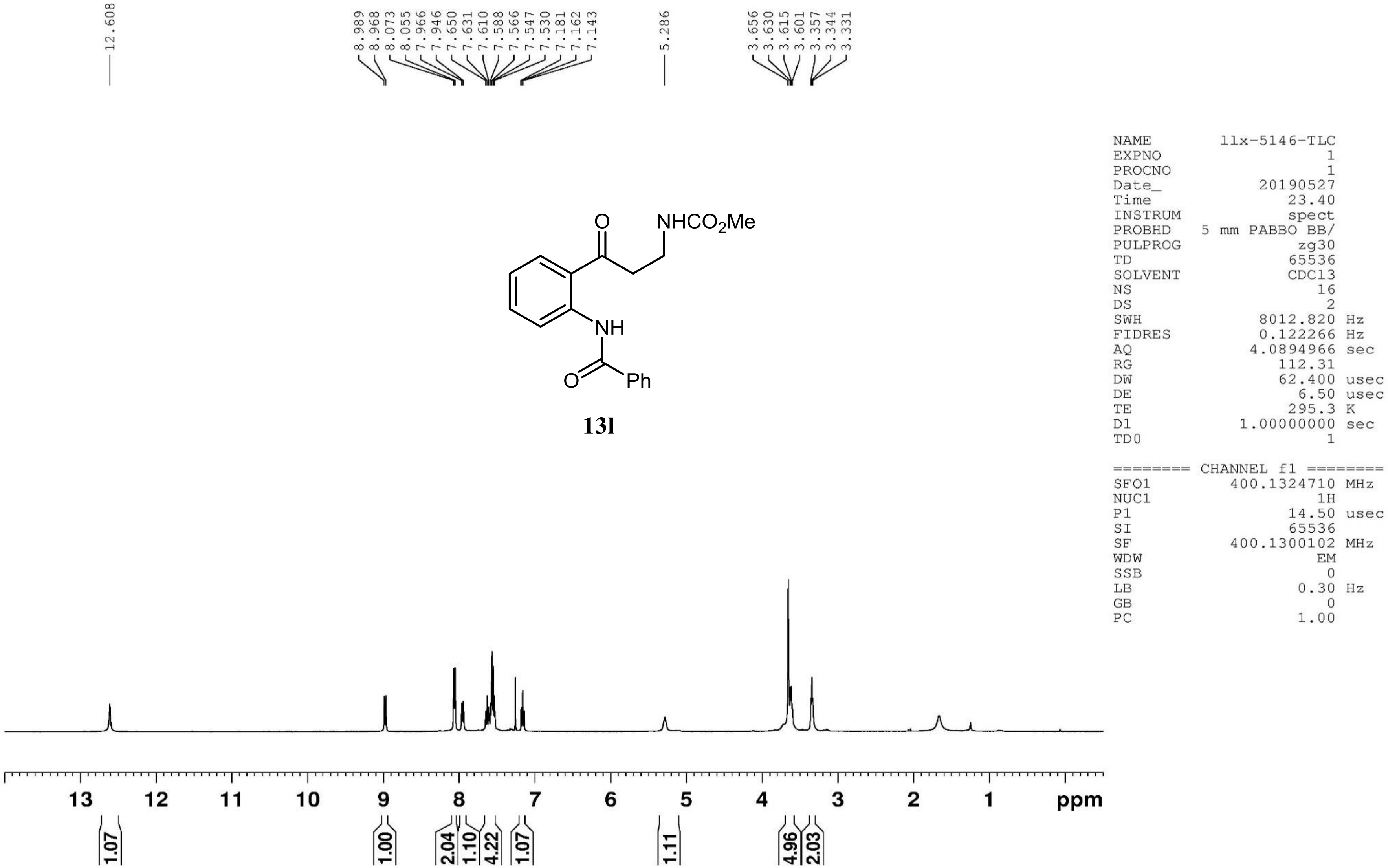

Supplementary Figure 234. ^{1}H-NMR of 13l

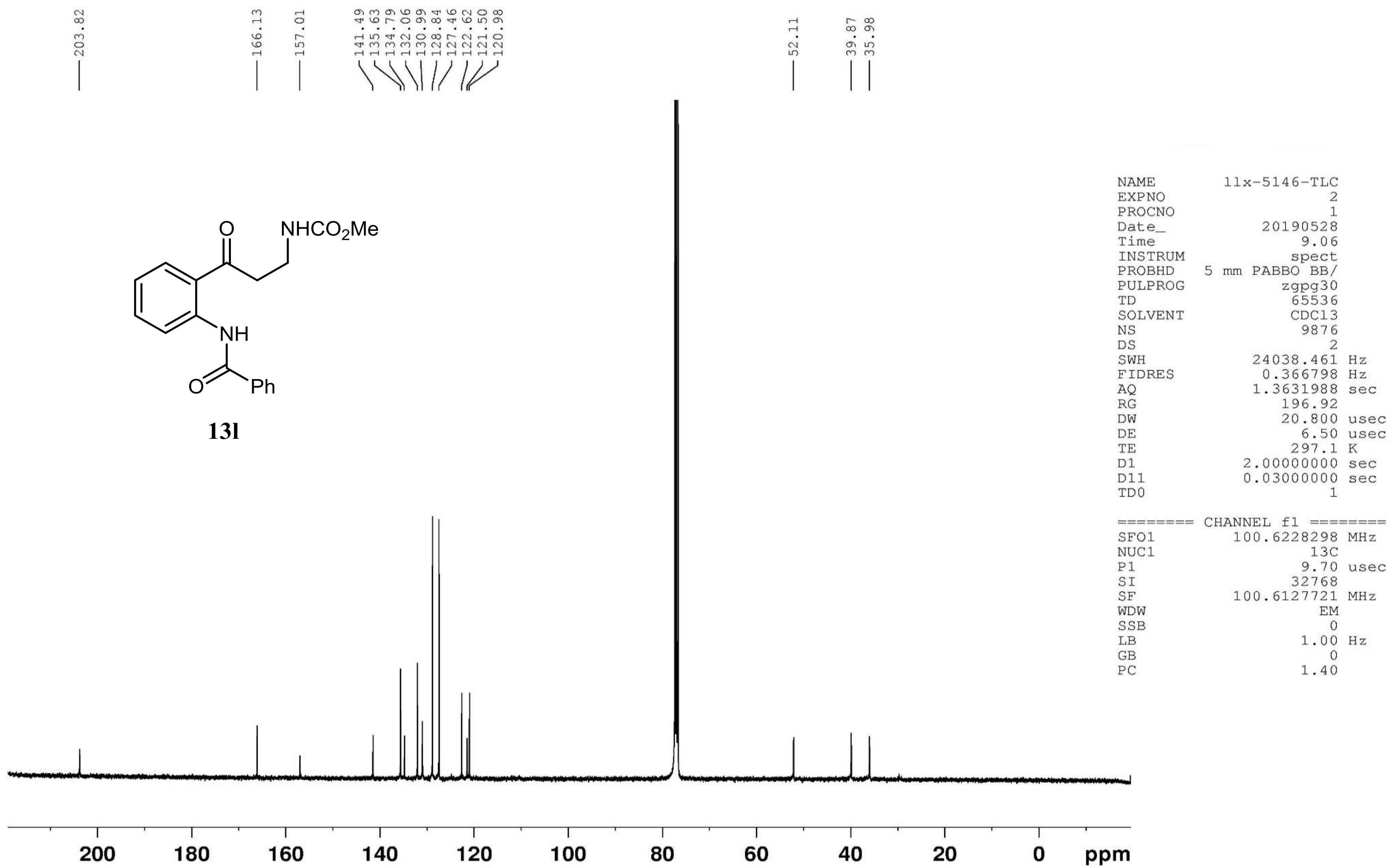

Supplementary Figure 235. ^{13}C-NMR of 13l

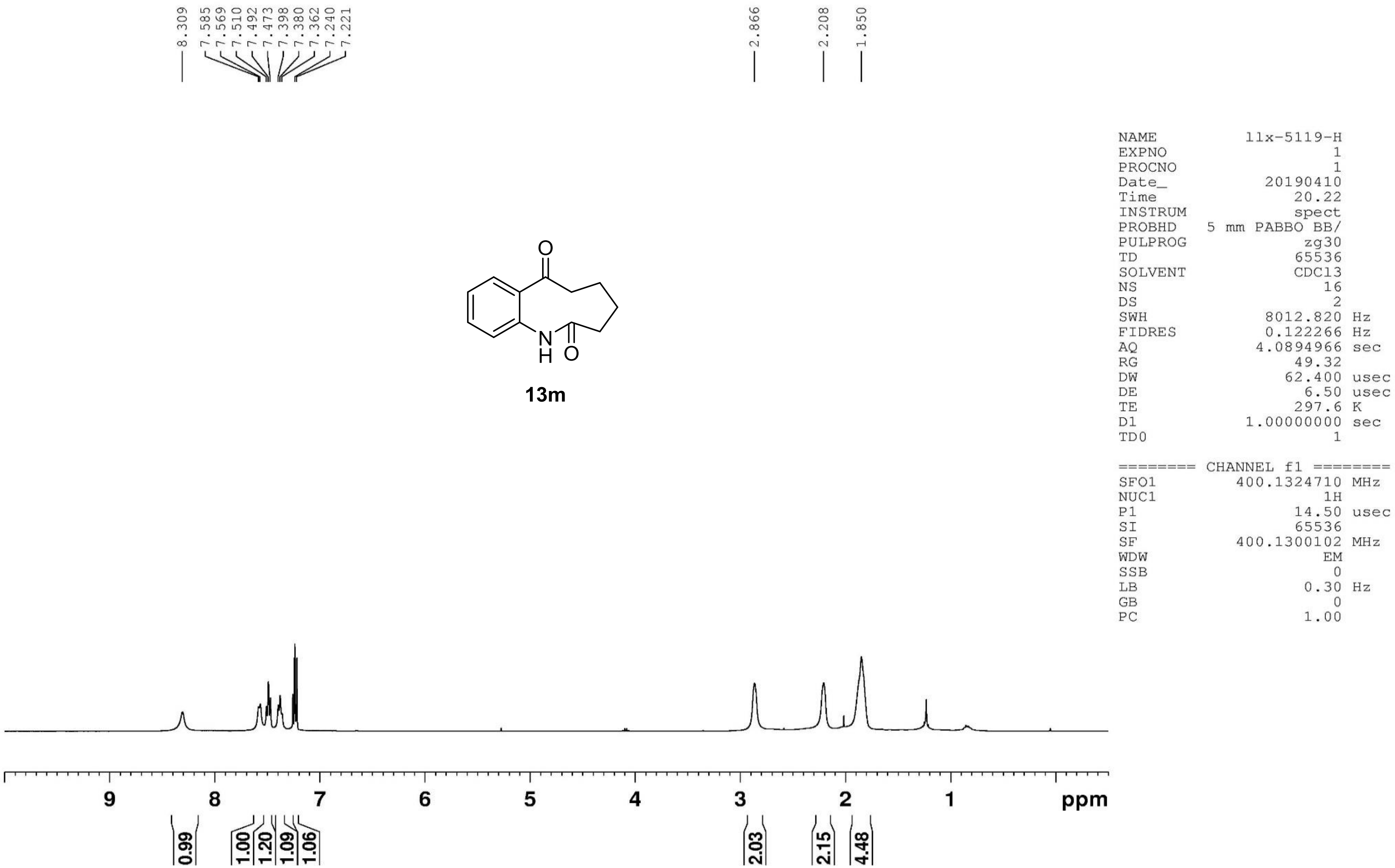

Supplementary Figure 236. ^{1}H-NMR of 13m

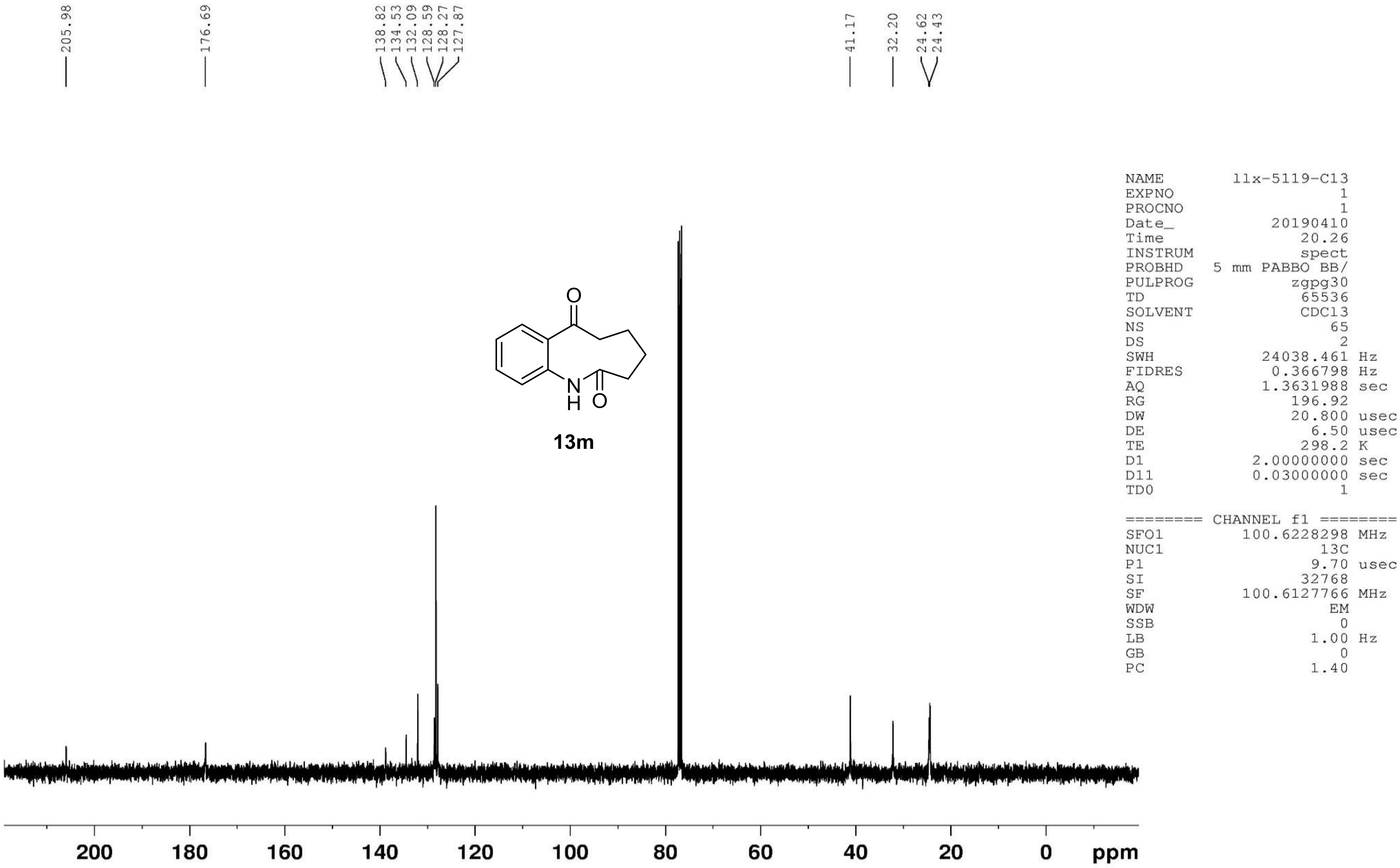

Supplementary Figure 237. ^{13}**C-NMR of 13m**

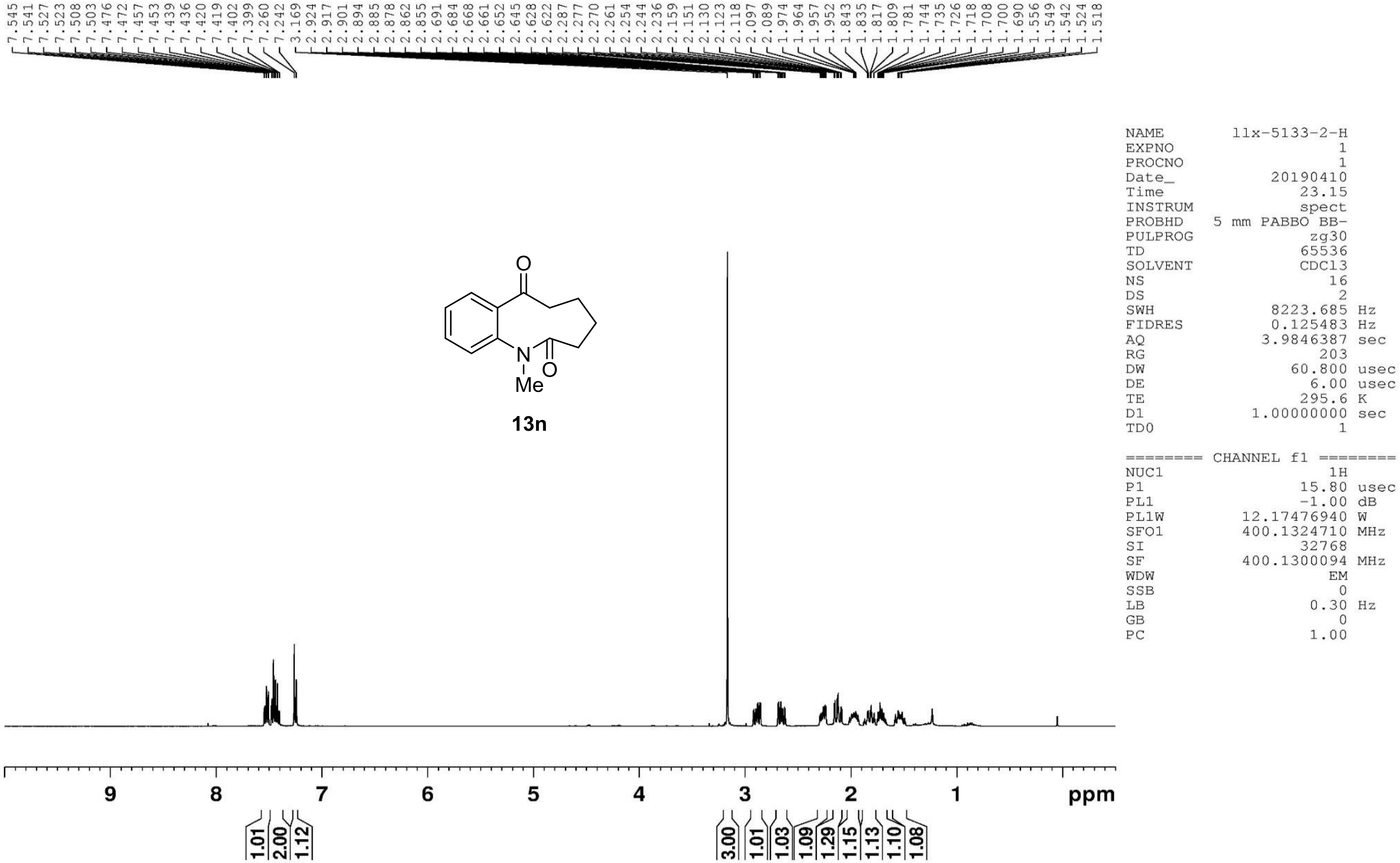

Supplementary Figure 238. ^{1}H-NMR of 13n

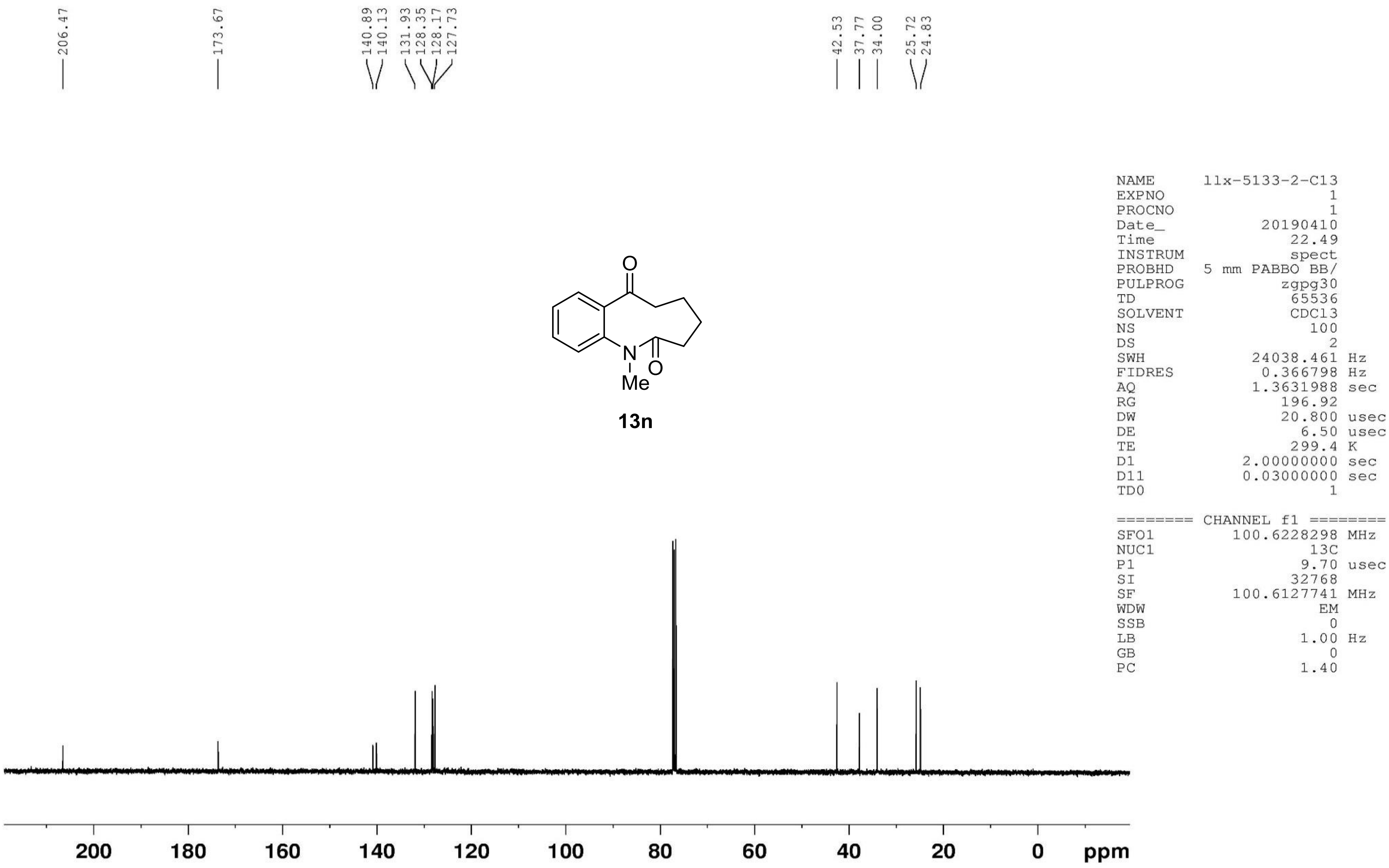

Supplementary Figure 239. ^{13}C-NMR of 13n

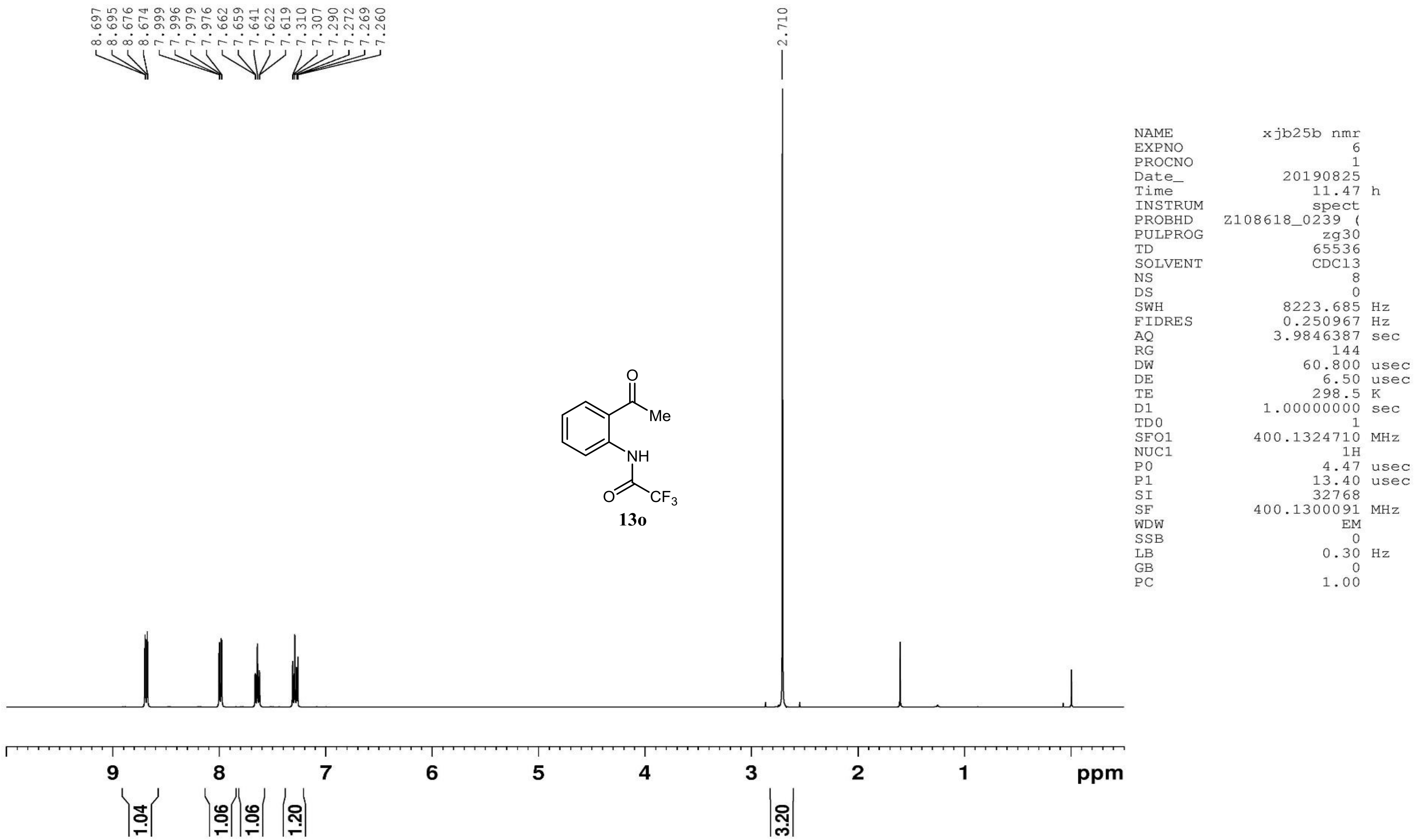

Supplementary Figure 240. ^{1}H-NMR of 13o

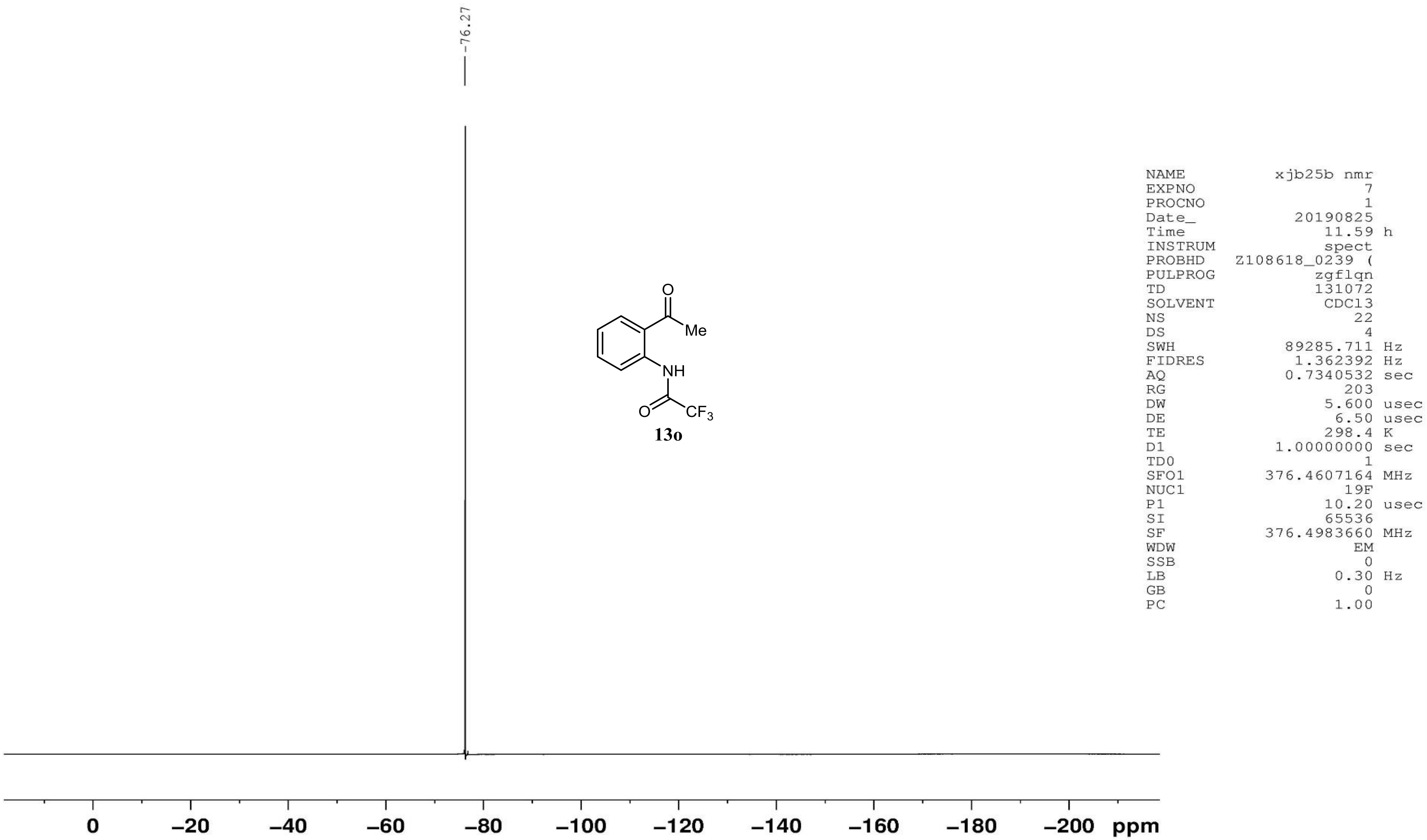

Supplementary Figure 241. ^{13}**C-NMR of 13o**

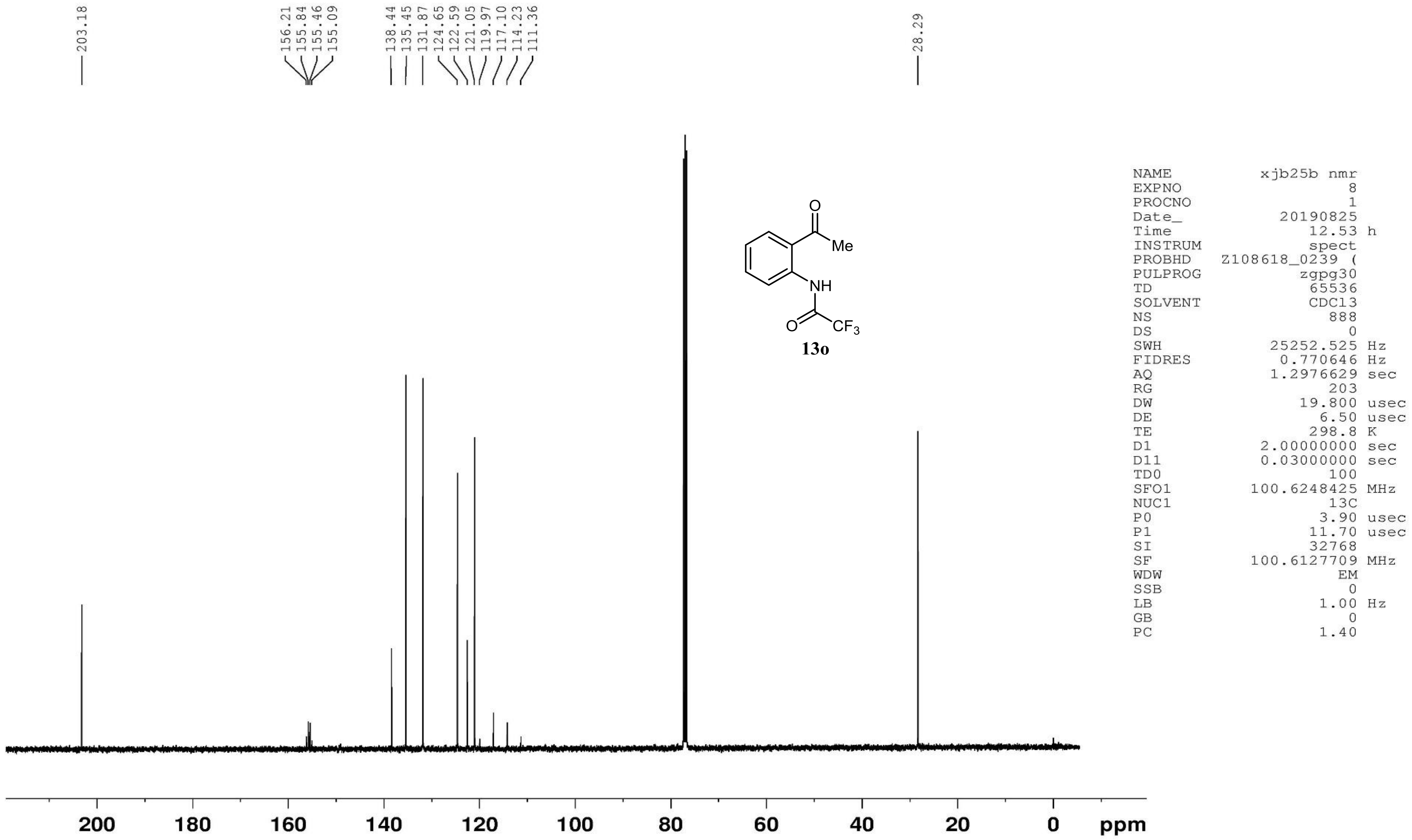

Supplementary Figure 242. ^{19}F-NMR of 13o

Supplementary Figure 243. X-Ray crystal structure of **2o**(CCDC:1935503)

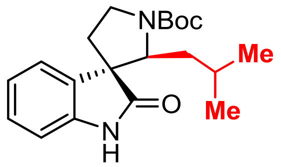

2o

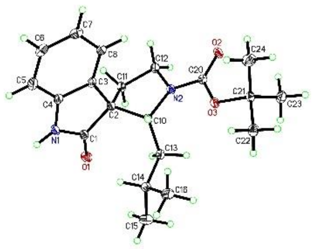

Supplementary Table 1: Crystal data and structure refinement for spirooxindole **2o.**

Identification code	lixin2CuLT
Empirical formula	$C_{20}H_{28}N_2O_3$
Formula weight	344.44
Temperature/K	100.00(10)
Crystal system	orthorhombic
Space group	$Pna2_1$
a/Å	11.4935(2)
b/Å	17.3724(2)
c/Å	9.47560(10)
α/°	90
β/°	90
γ/°	90
Volume/Å^3	1891.99(4)
Z	4
ρ_{calc}g/cm^3	1.209
μ/mm^{-1}	0.650
F(000)	744.0
Crystal size/mm^3	0.18 × 0.15 × 0.03
Radiation	CuKα (λ = 1.54184)
2Θ range for data collection/°	9.226 to 134.926
Index ranges	$-13 \leq h \leq 11, -20 \leq k \leq 17, -11 \leq l \leq 9$
Reflections collected	10098
Independent reflections	3102 [R_{int} = 0.0219, R_{sigma} = 0.0204]
Data/restraints/parameters	3102/1/231
Goodness-of-fit on F^2	1.027
Final R indexes [I>=2σ (I)]	R_1 = 0.0245, wR_2 = 0.0601
Final R indexes [all data]	R_1 = 0.0253, wR_2 = 0.0607
Largest diff. peak/hole / e Å^{-3}	0.14/-0.14
Flack parameter	-0.04(8)

Supplementary Figure 244. X-Ray crystal structure of 2 o'(CCDC:1935504)

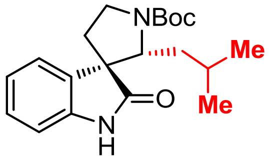

2o'

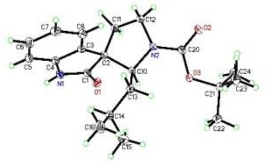

Supplementary Table 2: Crystal data and structure refinement for spirooxindole **2o'.**

Identification code	lixin3CuLT
Empirical formula	$C_{20}H_{28}N_2O_3$
Formula weight	344.44
Temperature/K	100.00(10)
Crystal system	triclinic
Space group	P-1
a/Å	8.4590(6)
b/Å	9.6113(8)
c/Å	12.0140(9)
α/°	73.358(7)
β/°	84.662(6)
γ/°	82.215(7)
Volume/Å^3	925.71(13)
Z	2
ρ_{calc}g/cm^3	1.236
μ/mm^{-1}	0.664
F(000)	372.0
Crystal size/mm^3	0.25 × 0.16 × 0.05
Radiation	CuKα (λ = 1.54184)
2Θ range for data collection/°	7.692 to 134.962
Index ranges	$-10 \leq h \leq 10$, $-11 \leq k \leq 11$, $-10 \leq l \leq 14$
Reflections collected	5085
Independent reflections	3278 [R_{int} = 0.0176, R_{sigma} = 0.0251]
Data/restraints/parameters	3278/0/231
Goodness-of-fit on F^2	1.021
Final R indexes [I>=2σ (I)]	R_1 = 0.0355, wR_2 = 0.1200
Final R indexes [all data]	R_1 = 0.0391, wR_2 = 0.1267
Largest diff. peak/hole / e Å^{-3}	0.35/-0.19

Supplementary Figure 245. X-Ray crystal structure of **6a**(CCDC:1935506)

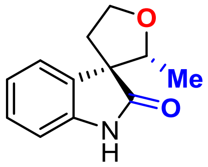

6a

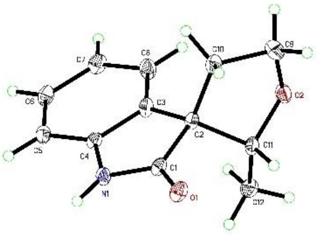

Supplementary Table 3: Crystal data and structure refinement for oxa-spirooxindole **6a.**

dentification code	lixin5CuLT
Empirical formula	$C_{12}H_{13}NO_2$
Formula weight	203.23
Temperature/K	100.00(10)
Crystal system	orthorhombic
Space group	$P2_12_12_1$
a/Å	8.64301(16)
b/Å	10.7925(2)
c/Å	10.89914(19)
α/°	90
β/°	90
γ/°	90
Volume/Å^3	1016.67(3)
Z	4
ρ_{calc}g/cm^3	1.328
μ/mm^{-1}	0.735
F(000)	432.0
Crystal size/mm^3	0.3 × 0.25 × 0.2
Radiation	CuKα (λ = 1.54184)
2Θ range for data collection/°	11.538 to 134.986
Index ranges	$-10 \leq h \leq 10$, $-9 \leq k \leq 12$, $-13 \leq l \leq 13$
Reflections collected	5455
Independent reflections	1815 [R_{int} = 0.0131, R_{sigma} = 0.0138]
Data/restraints/parameters	1815/61/202
Goodness-of-fit on F^2	1.030
Final R indexes [I>=2σ (I)]	R_1 = 0.0244, wR_2 = 0.0611
Final R indexes [all data]	R_1 = 0.0246, wR_2 = 0.0612
Largest diff. peak/hole / e Å^{-3}	0.17/-0.13
Flack parameter	0.5(2)

Supplementary Figure 246. X-Ray crystal structure of **6b**(CCDC:1935507)

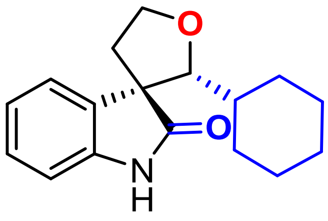

6b

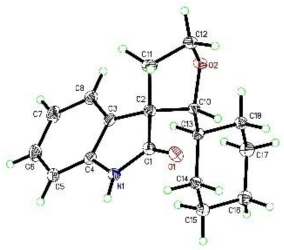

Supplementary Table 4: Crystal data and structure refinement for oxa-spirooxindole **6b.**

Identification code	lixin7CuLT
Empirical formula	$C_{17}H_{21}NO_2$
Formula weight	271.35
Temperature/K	100.00(10)
Crystal system	monoclinic
Space group	$P2_1/c$
a/Å	9.1543(2)
b/Å	19.1552(4)
c/Å	8.24510(15)
α/°	90
β/°	97.445(2)
γ/°	90
Volume/Å^3	1433.62(5)
Z	4
ρ_{calc}g/cm^3	1.257
μ/mm^{-1}	0.648
F(000)	584.0
Crystal size/mm^3	0.2 × 0.15 × 0.03
Radiation	CuKα (λ = 1.54184)
2Θ range for data collection/°	9.234 to 134.938
Index ranges	-10 ≤ h ≤ 10, -22 ≤ k ≤ 19, -9 ≤ l ≤ 9
Reflections collected	7675
Independent reflections	2574 [R_{int} = 0.0206, R_{sigma} = 0.0213]
Data/restraints/parameters	2574/0/181
Goodness-of-fit on F^2	1.043
Final R indexes [I>=2σ (I)]	R_1 = 0.0338, wR_2 = 0.0860
Final R indexes [all data]	R_1 = 0.0382, wR_2 = 0.0887
Largest diff. peak/hole / e Å^{-3}	0.23/-0.21

Supplementary Figure 247. X-Ray crystal structure of **6c**(CCDC:1935508)

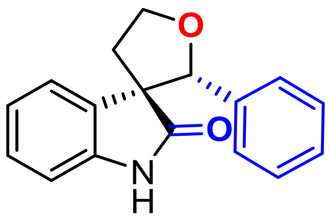

6c

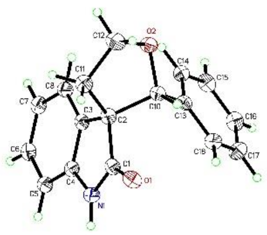

Supplementary Table 5: Crystal data and structure refinement for oxa-spirooxindole **6c.**

Identification code	lixin6CuLT
Empirical formula	$C_{17}H_{15}NO_2$
Formula weight	265.30
Temperature/K	100.00(10)
Crystal system	monoclinic
Space group	$P2_1/n$
a/Å	9.1540(6)
b/Å	12.9987(5)
c/Å	11.7253(7)
α/°	90
β/°	106.429(7)
γ/°	90
Volume/Å^3	1338.23(14)
Z	4
ρ_{calc}g/cm^3	1.317
μ/mm^{-1}	0.693
F(000)	560.0
Crystal size/mm^3	0.06 × 0.06 × 0.05
Radiation	CuKα (λ = 1.54184)
2Θ range for data collection/°	10.402 to 134.984
Index ranges	$-10 \leq h \leq 10$, $-14 \leq k \leq 15$, $-8 \leq l \leq 14$
Reflections collected	6924
Independent reflections	2393 [R_{int} = 0.0426, R_{sigma} = 0.0424]
Data/restraints/parameters	2393/0/181
Goodness-of-fit on F^2	1.029
Final R indexes [I>=2σ (I)]	R_1 = 0.0447, wR_2 = 0.1160
Final R indexes [all data]	R_1 = 0.0563, wR_2 = 0.1233
Largest diff. peak/hole / e Å^{-3}	0.23/-0.25

Supplementary References

1. Ye, J. et al. Oxidative rearrangement coupling reaction for the functionalization of tetrahydro-β-carbolines with aromatic amines. *Angew. Chem. Int. Ed.* **56,** 14968-14972 (2017).
2. Yamanaka, E., Shibata, N. & Sakai, S. A development of Pictet–Spengler reaction in aprotic media using chloroformates; a short synthesis of borrerine. *Heterocycles* **22,** 371-374 (1984).
3. Tsuji, R., Yamanaka, M., Nishida, A. & Nakagawa, M. Pictet–Spengler reaction of nitrones and imines catalyzed by $Yb(OTf)_3$–TMSCl. *Chem. Lett.* **31,** 428-429 (2002);
4. Li, C., Chan, C., Heimann, A. C. & Danishefsky, S. J. On the rearrangement of an azaspiroindolenine to a precursor to phalarine: mechanistic insights. *Angew. Chem. Int. Ed.* **46,** 1444-1447 (2007).
5. Pigge, F. C.; Fang, S. & Rath N. P. Reduction of tricarbonyl(η^6-Indole)chromium(0) complexes. *J. Organomet. Chem.* **559,** 131-140 (1998).
6. Jana. N., Nguyen, Q. & Driver, T. G. Development of a Suzuki cross-coupling reaction between 2-azidoarylboronic pinacolate esters and vinyl triflates to enable the synthesis of [2,3]-fused indole heterocycles. *J. Org. Chem.* **79,** 2781-2791 (2014).
7. Kuehne, M. E.; Roland, D. M. & Hafter, R. Studies in biomimetic alkaloid syntheses. 2. Synthesis of vincadifformine from tetrahydro-.beta.-carboline through a secodine intermediate. *J. Org. Chem.* **43,** 3705-3710 (1978).
8. Naota, T., Nakato, T. & Murahashi, S. I. Novel method for α-substitution of amines via N-methoxycarbonyl-α-*t*-butyldioxyamines.*Tetrahedron Lett.* **51,** 7475-7478 (1990).
9. Kawate, T., Nakagawa, M., Yamazaki, H., Hirayama, M. & Hino, T. Alkylation of 3, 4-Dihydro-β-carboline. *Chem. Pharm. Bull.* **41,** 287-291 (1993).
10. Skouta, R., Hayano, M., Shimada, K. & Stockwell, B. R. Design and synthesis of Pictet-Spengler condensation products that exhibit oncogenic-RAS synthetic lethality and induce non-apoptotic cell death. *Bioorg. Med. Chem. Lett.* **22,** 5707-5713 (2012).
11. Chen, L.*et al.* Design, synthesis, and biological activities of spirooxindoles containing acylhydrazone fragment derivatives based on the biosynthesis of alkaloids derived from tryptophan. *J. Agric. Food Chem.* **64,** 6508-6516 (2016).
12. Zhou, S. & Tong, R. Three-step catalytic asymmetric total syntheses of 13-methyltetrahydroprotoberberine alkaloids. *Org. Lett.* **19,** 1594-1597 (2017).
13 Diker, K., Biach, K. E., de Maindreville, M. D. & Lévy, J. Reductive Pictet−Spengler cyclization of nitriles in the presence of tryptamine: synthesis of indolo[2,3-a]quinolizidine, nazlinine, and elaeocarpidine. *J. Nat. Prod.* **60,** 791-793 (1997).
14 Chen, J.- Q., Wei, Y.- L., Xu, G.- Q., Liang, Y.- M. & Xu, P.- F. Intramolecular 1,5-H transfer reaction of aryl iodides through visible-light photoredox catalysis: a concise method for the synthesis of natural product scaffolds. *Chem. Commun.* **52,** 6455-6458 (2016).
15 Görmen, M., Goff, R. L., Lawson, A. M., Daïch, A. & Comesse, S. Tandem aza-Michael/spiro-ring closure sequence: access to a versatiles caffold and total synthesis of (±)-coerulescine.*Tetrahedron Lett.* **54,** 2174-2176 (2013).
16. Bell, S. E. V., Brown, R. F. C., Eastwood, F. W. & Horvath, J. M. An approach to some spiro oxindole alkaloids through cycloaddition reactions of 3-methylideneindolin-2-one. *Aust. J. Chem.* **53,** 183-190 (2000).
17. Miyake, F. Y., Yakushijin, K. & Horne, D. A. Preparation and synthetic applications of 2-halotryptamines: synthesis of elacomine and isoelacomine.*Org. Lett.* **6,** 711-713 (2004).
18. De, S., Das, M. K., Bhunia, S. & Bisai, A. Unified approach to the spiro(pyrrolidinyl-oxindole) and hexahydropyrrolo[2,3-b]indole alkaloids: total syntheses of pseudophrynamines 270 and 272A. *Org. Lett.* **17,** 5922-5925 (2015).
19. Mukaiyama, T., Ogata, K., Sato, I. & Hayashi, Y. Asymmetric organocatalyzed michael addition of nitromethane to a 2-oxoindoline-3-ylidene acetaldehyde and the three one-pot sequential synthesis of (−)-horsfiline and (−)-coerulescine. *Chem. Eur. J.* **20,** 13583-13588 (2014).
20. Paciaroni, N. G. *et al.* A Tryptoline ring-distortion strategy leads to complex and diverse biologically active molecules from the indole alkaloid yohimbine. *Chem. Eur. J.* **23,** 4327-4335 (2017).
21. Wilson, R. M., Farr, R. A. & Burlett, D. J. Synthesis and chemistry of a stabilized dehydrosecodine model system. *J. Org. Chem.* **46,** 3293-3302 (1981).
22. Maskeri, M. A., O'Connor, M. J., Jaworski, A. A., Davies, A. V. & Scheidt, K. A. A cooperative hydrogen bond donor-brønsted

acid system for the enantioselective synthesis of tetrahydropyrans. *Angew. Chem. Int. Ed.* **57,** 17225-17229 (2018)

23. Kang, T. *et al.* Asymmetric catalytic double michael additions for the synthesis of spirooxindoles. *Chem. Eur. J.* **24,** 3703-3706 (2018).
24. DiPoto, M. C., Hughes, R. P. & Wu, J. Dearomative indole (3 + 2) reactions with azaoxyallyl cations-new method for the synthesis of pyrroloindolines. *J. Am. Chem. Soc.* **137,** 14861-14864 (2015).
25. Senthilkumar, S. & Kumarraja, M. Regioselective N-allylation and N-cinnamylation of indoles using CuI-exchanged hierarchical nanoporous material. *Catal. Commun.* **70,** 86-89 (2015).
26. Petit, S. *et al.* Structure-activity relationship analysis of the peptide deformylase inhibitor 5-bromo-1H-indole-3-acetohydroxamic acid. *ChemMedChem.* **4,** 261-275 (2009).
27. Repka, L. M., Ni, J. & Reisman, S. E. Enantioselective synthesis of pyrroloindolines by a formal [3 + 2] cycloaddition reaction. *J. Am. Chem. Soc.* **132,** 14418-14420 (2010).
28. Trost, B. M. & Quancard, J. Palladium-catalyzed enantioselective C-3 allylation of 3-substituted-1h-indoles using trialkylboranes *J. Am. Chem. Soc.* **128,** 6314-6315 (2006).
29. Zheng, N., Chang, Y.-Y., Zhang, L.-J., Gong, J.-X. & Yang, Z. Gold-catalyzed intramolecular tandem cyclization of indole-ynamides: diastereoselective synthesis of spirocyclic pyrrolidinoindolines. *Chem. Asian J.* **11,** 371-375 (2016).
30. Liu, C. *et al*. Enantioselective synthesis of 3a-amino-pyrroloindolines by copper-catalyzed direct asymmetric dearomative amination of tryptamines. *Angew. Chem. Int. Ed.* **55,** 751-754 (2016).
31. Wanner, M. J., Claveau, E., Maarseveen, J. H. V. & Hiemstra, H. Enantioselective syntheses of corynanthe alkaloids by chiral brønsted acid and palladium catalysis. *Chem. Eur. J.* **17,** 13680-13683 (2011).
32. Rivera-Becerril, E., Joseph-Nathan, P., Pérez-Álvarez, V. M. & Morales-Ríos, M. S. Synthesis and biological evaluation of (−)- and (+)-debromoflustramine B and its analogues as selective butyrylcholinesterase inhibitors. *J. Med. Chem.* **51,** 5271-5284 (2008).
33. Stephens, D. E. & Larionov, O. V. Straightforward Access to Hexahydropyrrolo[2,3-b]indole core by a regioselective C3-azo coupling reaction of arenediazonium compounds with tryptamines. *Eur. J. Org. Chem.* **2014,** 3662-3670 (2014).
34. Han, L., Liu, C., Zhang, W., Shi, X.- X. & You, S.- L. Dearomatization of tryptophols via a vanadium-catalyzed asymmetric epoxidation and ring-opening cascade.Chem. Commun. **50,** 1231-1233 (2014).
35. Cooper, L. *et al.* Synthesis of a novel type of 2,3′-BIMs via platinum-catalysed reaction of indolylallenes with indoles. *Chem. Eur. J.* **24,** 6105-6114 (2018).
36. Liu, Y.*et al.* Palladium-catalyzed selective heck-type diarylation of allylic esters with aryl halides involving a β-OAc elimination process *Org. Lett.* **13,** 1126-1129 (2011).
37. Xing, Q., Lv, H., Xia, C. & Li, F. Intramolecular cooperative C-C bond cleavage reaction of 1,3-dicarbonyl compounds with 2-iodoanilines to give o-(n-acylamino)aryl ketones and multisubstituted indoles. *Chem. Eur. J.* **21,** 8591-8596 (2015).
38. Yagoubi, M., Cruz, A. C. F., Nichols, P. L., Elliott, R. L. & Willis, M. C. Cascade palladium-catalyzed direct intramolecular arylation/alkene isomerization sequences: synthesis of indoles and benzofurans. *Angew. Chem. Int. Ed.* **49,** 7958-7962 (2010).
39. Kuwano, R. & Kashiwabara, M. Ruthenium-catalyzed asymmetric hydrogenation of N-Boc-indoles.*Org. Lett.* **8,** 2653-2655(2006).
40. Xu, J. & Tong, R. An environmentally friendly protocol for oxidative halocyclization of tryptamine and tryptophol derivatives. *Green Chem.* **19,** 2952-2956 (2017).
41. Shen, Z. *et al.* Synthesis of naked amino-pyrroloindoline via direct aminocyclization of tryptamine. *Org. Biomol. Chem.* **13,** 5381-5384 (2015).
42. Shimizu, R.et al. Direct C2-trifluoromethylation of indole derivatives catalyzed by copper acetate. *Tetrahedron Lett.* **51,** 5947-5949 (2010).
43. Zhang, C.- P. et al. Copper-mediated trifluoromethylation of heteroaromatic compounds by trifluoromethyl sulfonium salts. *Angew. Chem. Int. Ed.* **50,** 1896-1900 (2011).

44 Kumar, M. P. & Liu, R. S. Zn(OTf)$_2$-catalyzed cyclization of proparyl alcohols with anilines, phenols, and amides for synthesis of indoles, benzofurans, and oxazoles through different annulation mechanisms. *J. Org. Chem.* **71,** 4951-4955 (2006).

45. Ackermann, L. & Althammer, A. One-pot 2-aryl/vinylindole synthesis consisting of a ruthenium-catalyzed hydroamination and

a palladium-catalyzed heck reaction using 2-chloroaniline.*Synlett*, **18,** 3125-3129 (2006).

46. Yeung, C. S., Ziegler, R. E., Porco, Jr, J. A. & Jacobsen, E. N. Thiourea-catalyzed enantioselective addition of indoles to pyrones: alkaloid cores with quaternary carbons.J. Am. Chem. Soc. **136,** 13614-13617 (2014);
47. Verma, A. & Kumar, S. Selective oxidative decarbonylative cleavage of unstrained $C(sp^3)$–$C(sp^2)$ bond: synthesis of substituted benzoxazinones.*Org. Lett.* **18,** 4388-4391 (2016).
48. Yin, Z. & Sun, P. Palladium-catalyzed direct ortho-acylation through an oxidative coupling of acetanilides with toluene derivatives. *J. Org. Chem.* **77,** 11339-11344 (2012).
49. Errede, L. A.; Oien, H. T. & Yarian, D. R. Acylanthranils. 3. The influence of ring substituents on reactivity and selectivity in the reaction of acylanthranils with amines. *J. Org. Chem.* **42,** 12-18 (1977).
50. Prashanth, M. K. & Revanasiddappa, H. D. Synthesis of some new glutamine linked 2,3- disubstituted quinazolinone derivatives as potent antimicrobial and antioxidant agents. *Med. Chem. Res*. **22,** 2665-2676 (2013).
51. Witkop, B., Patrick, J.B. & Rosenblum, M. Ring effects in autoxidation. a new type of camps reaction. *J. Am. Chem. Soc.* **73,** 2641-2647 (1951).
